# Organ-Resolved Salicylate–Phenylpropanoid Metabolite Profiling of *Echinacea purpurea*, *E. pallida*, and *E. tennesseensis* Tissues Extracted with 50% Aqueous Methanol by LC–MS/MS and LC–HRMS

**DOI:** 10.3390/molecules31172963

**Published:** 2026-08-25

**Authors:** Chuck Chang, Min Du, Yiming Zhang, Tingkwan Lau, Jan V. Slama, Robert O’Brien, Afoke Ibi, Julia Solnier

**Affiliations:** 1 Research Department, ISURA, Burnaby, BC V3N 4S9, Canada; mdu@isura.ca (M.D.); yzhang@isura.ca (Y.Z.); wlau@isura.ca (T.L.); aibi@isura.ca (A.I.); jsolnier@isura.ca (J.S.); 2 Research and Education, Factors Farms, Kelowna, BC V1Y 8P4, Canada; jslama@factorsgroup.com; 3 Biology Department, University of British Columbia, Kelowna, BC V1V 1V7, Canada; r.ob@suprarnd.ca

**Keywords:** *Echinacea purpurea*, *Echinacea pallida*, *Echinacea tennesseensis*, salicylic acid, salicylate pathway, phenylpropanoids, caffeic acid derivatives, flavonoids, LC–HRMS, plant metabolomics, plant defense, secondary metabolism

## Abstract

Salicylic acid (SA) is a central plant defense hormone, but its occurrence and metabolic context in *Echinacea* tissues have not been systematically characterized. This study aimed to characterize endogenous free SA, salicylate-associated features, and their organ-level distributions in selected specimens of *Echinacea purpurea*, *E. pallida*, and *E. tennesseensis*, and to evaluate free SA variability among individual *E. purpurea* flower heads. Targeted LC–MS/MS and retrospective LC–HRMS profiling were used to examine free SA, preservative panel analytes, salicylate-related pathway features, phenylpropanoid/caffeic acid derivatives, and flavonoid-related metabolites across roots, stems, leaves, and flowers of *Echinacea purpurea*, *E. pallida*, and *E. tennesseensis*. Free SA was detected in selected aerial and reproductive tissue extracts, whereas benzoic acid, dehydroacetic acid, and paraben esters were not detected. Confidence-qualified LC–HRMS yielded formula-level assignments and tentative candidates associated with salicylates, phenylpropanoids, caffeic-acid derivatives, and flavonoids. Free SA detection and broader salicylate-associated feature patterns were not identical, with candidate conjugated or pathway-related features also observed in roots. Analysis of 80 individual *E. purpurea* flower heads showed marked free SA variability (mean ± SD 4.72 ± 13.77 μg/g; range 0–121.48 μg/g). These findings support the endogenous occurrence of free SA in *Echinacea* within a broader, tissue-variable specialized-metabolite context. Future studies with greater biological replication, authentic standards, seasonal sampling, and mechanistic approaches should clarify salicylate biosynthesis, regulation, and biological significance in *Echinacea*.

## 1. Introduction

Salicylic acid (SA) is a small phenolic acid phytohormone with central roles in plant defense, development, and stress adaptation. In plants, SA participates in local and systemic immune signaling, regulates transcriptional programs associated with systemic acquired resistance, and interacts with other hormone networks including jasmonates and ethylene [1,2,3,4]. Although SA is also widely recognized because of its pharmacological relationship to acetylsalicylic acid, its primary relevance in plant biology is an endogenous signal that coordinates responses to biotic and abiotic stress [5,6]. Accordingly, the distribution of SA and related metabolites across plant organs can provide insight into tissue-specific defense physiology, stress status, and secondary-metabolite regulation [7].

Plant SA biosynthesis occurs principally through the isochorismate pathway [8]. In the model plant *Arabidopsis thaliana*, chorismate is converted to isochorismate by isochorismate synthase 1 (ICS1) in plastids [9,10]. Isochorismate is then exported to the cytosol by Enhanced Disease Susceptibility 5 (EDS5), where avrPphB Susceptible 3 (PBS3) catalyzes the formation of isochorismate-9-glutamate (I9G) [9]. I9G subsequently yields SA through EPS1-promoted decomposition and related chemistry [9,11]. Once produced, SA can be converted into multiple downstream forms, including salicylic acid 2-O-β-D-glucoside (SAG) and salicylic acid glucose ester (SGE), which function as reversible storage or sequestration forms, methyl salicylate (MeSA), which acts as a volatile or mobile defense signal, and gentisic acid or related dihydroxybenzoic acids, which are associated with downstream stress metabolism [12,13,14]. A phenylalanine ammonia-lyase (PAL)-linked route through *trans*-cinnamic acid and benzoic acid has also been described in some plant systems, although the relative contribution of this route appears to vary among taxa and physiological conditions [15,16].

The genus *Echinacea* provides a useful system in which to examine salicylate pathway metabolism because it is rich in phenylpropanoid-derived secondary metabolites [17,18]. Species such as *Echinacea purpurea*, *E. pallida*, and *E. tennesseensis* accumulate caffeic acid derivatives, including caftaric acid, chlorogenic acid isomers, cichoric acid, dicaffeoylquinic acids, and related phenolics, as well as flavonoids and alkamides [19,20,21,22]. These compounds are commonly used as phytochemical markers for *Echinacea* raw materials and extracts, and many are associated with plant defense chemistry [23,24,25,26]. Importantly, salicylate metabolism and caffeic acid derivative biosynthesis share an upstream aromatic amino acid and phenylpropanoid context, particularly through phenylalanine and *trans*-cinnamic acid [15,27,28]. Thus, salicylate-related metabolites should not be interpreted in isolation, but rather as part of a broader network of defense-associated secondary metabolism [29].

Despite extensive study of *Echinacea* phytochemistry, SA and related salicylate pathway metabolites have not been systematically mapped across organs and species in relation to the broader phenylpropanoid and flavonoid background. This represents a gap in understanding because free SA alone may not reflect total salicylate pathway activity [30]. Conjugated forms, biosynthetic precursors, downstream products, and co-occurring phenylpropanoid metabolites may provide a more biologically meaningful view of the pathway [1,31]. In addition, plant defense metabolites often display strong organ specificity and non-normal biological variability, especially in reproductive tissues exposed to environmental stress, microbial contact, herbivory, and developmental heterogeneity [32,33,34].

High-resolution mass spectrometry (HRMS) provides an opportunity to evaluate salicylate metabolism at the pathway level [35]. Rather than measuring free SA alone, liquid chromatography–HRMS (LC-HRMS) can retrospectively profile accurate-mass features corresponding to salicylate conjugates, isochorismate pathway features, phenylpropanoid intermediates, caffeic acid derivatives, and flavonoid-related metabolites [36,37]. When combined with targeted LC–MS/MS quantitation of free SA and related small phenolic acids, this approach can distinguish tissue-specific accumulation of free SA from broader pathway-associated metabolite signatures [38]. Such integration is particularly useful for medicinal and aromatic plants, where secondary-metabolite profiles are shaped by both genetic background and environmental conditions [39,40,41,42].

Previous *Echinacea* phytochemical studies have focused primarily on alkamides, caffeic acid derivatives, flavonoids, chemotaxonomic variation, and responses to exogenous elicitors [43,44,45]. However, the endogenous occurrence of free salicylic acid and its relationship to salicylate-associated and phenylpropanoid-related features have not been systematically examined across organs of *E. purpurea*, *E. pallida*, and *E. tennesseensis*. We therefore tested three hypotheses: (i) free SA and salicylate-associated features would show different detection patterns among the analyzed root, stem, leaf, and flower extracts; (ii) the occurrence of free SA would not necessarily parallel that of salicylate conjugates, candidate pathway intermediates, or other related LC–HRMS features; and (iii) free SA concentrations would vary substantially among individual *E. purpurea* flower heads. To test these hypotheses, free SA and preservative panel analytes were measured by targeted LC–MS/MS, and existing LC–HRMS data were screened retrospectively for confidence-qualified salicylate-associated, phenylpropanoid, caffeic acid derivative, and flavonoid-related features. Organ-level observations were evaluated descriptively in selected specimens from the three species, and free SA variability was characterized in 80 individual *E. purpurea* flower heads. The novelty of this study lies in combining targeted quantification of endogenous free SA, organ-resolved LC–HRMS screening across three *Echinacea* species, explicit feature-level annotation confidence, and individual-flower analysis within one analytical framework.

## 2. Results

### 2.1. Targeted LC–MS/MS Quantitation

Salicylic acid (SA) and preservative panel analytes in tissues of three *Echinacea* species were measured by LC–MS/MS (μg/g) (Table 1). Benzoic acid (BA), dehydroacetic acid, methyl-, ethyl-, isopropyl-, propyl-, sec-butyl-, iso-butyl- and butyl *p*-hydroxybenzoates were also measured. None were detected in the 16 analyzed samples. Free SA was detected in selected flower, leaf, and stem extracts but not in the analyzed root extracts.

### 2.2. Retrospective LC–HRMS Metabolite Profiling

Existing LC–HRMS datasets were retrospectively screened to complement the targeted LC-MS/MS results. The search included features associated with salicylate biosynthesis, salicylate conjugation, phenylpropanoid metabolism, caffeic acid derivatives and flavonoid-related families. Extracted-ion peak areas were integrated for pre-defined accurate-mass features. These included candidate salicylates, chorismite-related compounds, caffeic acid derivatives, hydroxycinnamic acids, and flavonoid glycoside families. The complete candidate list is provided in Table 2.

The expanded HRMS profile indicates that salicylate-related features occur within a metabolome dominated by phenylpropanoid-derived metabolites rather than as isolated signals. Caftaric acid was among the dominant HRMS features in select specimens from the three species, consistent with the established importance of caffeic acid derivatives in *Echinacea* phytochemistry [46,47]. Additional caffeic acid derivative features, including chlorogenic acid isomers, cichoric acid, dicaffeoylquinic acid and caffeic acid, were also detected across multiple tissues. These observations are consistent with the presence of diverse phenylpropanoid-derived metabolites and provide chemical context for the salicylate-associated candidate features [48,49].

Salicylate-associated features, including SA glucoside/ester family signals, salicyloyl-malate, methyl salicylate, gentisic acid and putative isochorismate/chorismate or I9G-related features, were detected at variable intensities among the extracts analyzed across species and tissues. Free SA was not detected in the analyzed root extracts by targeted LC–MS/MS. In contrast, LC-HRMS yielded formula-level or tentative candidate features associated with salicylate conjugates or related metabolites in some root extracts. Therefore, free SA and the broader LC-HRMS feature profile provided different analytical readouts. The analyzed root extracts may contain related or conjugated candidates despite the non-detection of free SA; however, the tentative assignments do not establish their identities or biological roles.

Because these annotations were derived from retrospective accurate-mass extraction and were not supported by authentic standards for every feature, the values in Table 2 are best interpreted as relative HRMS signal intensities rather than absolute concentrations. Within this constraint, the salicylate-associated candidates occurred alongside numerous phenylpropanoid and caffeic acid derivative-related features. This pattern places confirmed free SA within a broader specialized-metabolite profile. Compound names assigned without authentic-standard confirmation are reported as putatively annotated features or tentative candidates. Such assignments indicate consistency with the proposed molecular formula or structure but do not confirm metabolite identity, pathway activity, or metabolic flux.

Retrospective HRMS mining also revealed abundant signals corresponding to caffeic acid derivatives, especially caftaric acid, chlorogenic/neochlorogenic/cryptochlorogenic acid isomers, cichoric acid, dicaffeoylquinic acid and caffeic acid. These metabolites were detected among the analyzed extracts across species and tissues, with particularly large peak areas for caftaric acid and flavonoid glycoside families. This finding is consistent with the established phytochemical identity of *Echinacea*, in which caffeic acid derivatives are major phenylpropanoid products and commonly used quality markers. The co-occurrence of these CAD features with salicylate-related metabolites places SA within a broader phenylpropanoid/stress response network rather than as an isolated preservative-like compound.

### 2.3. Organ-Level Visualization of Metabolite Distribution

**Figure 1:**
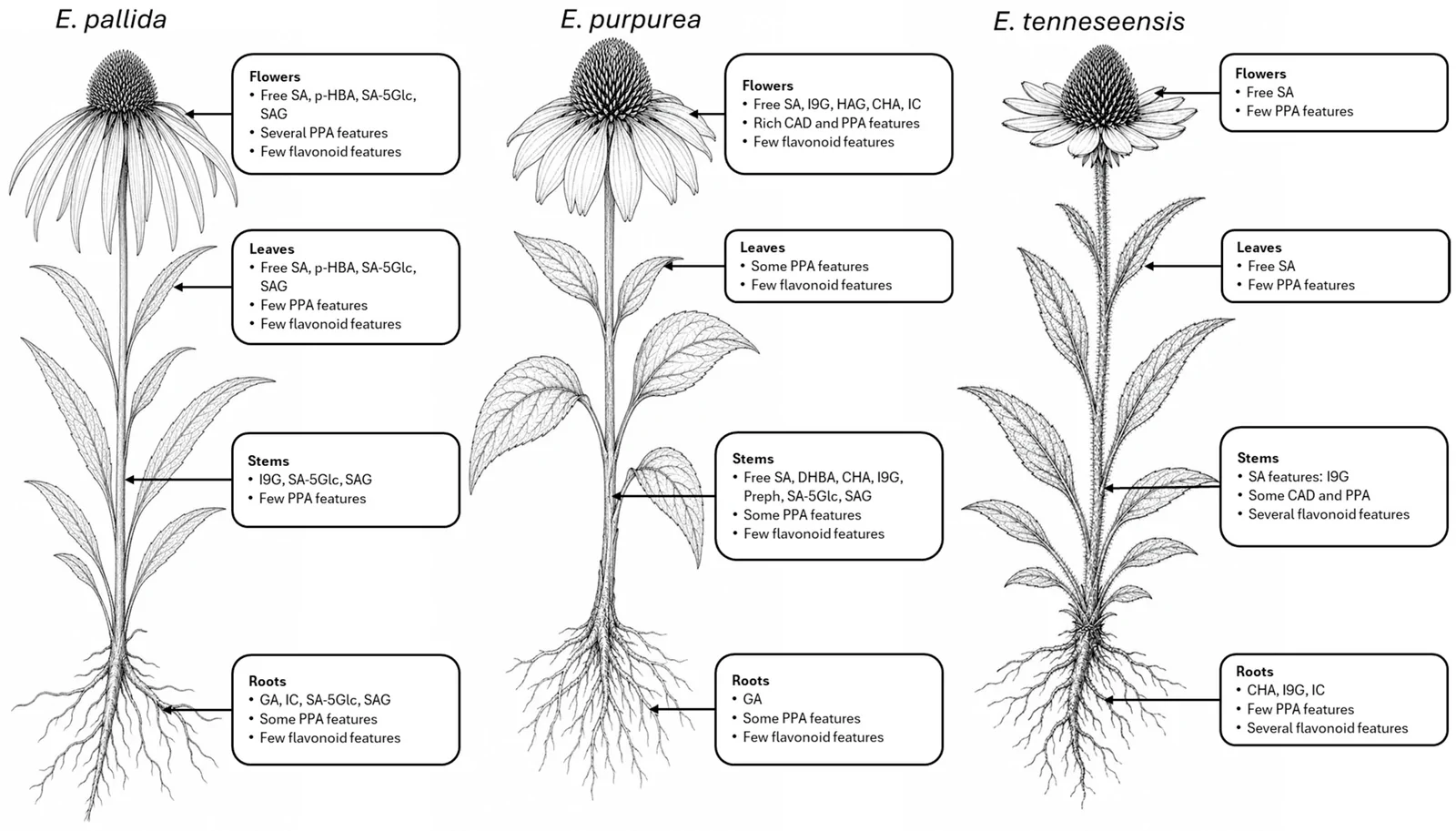
An interpretive summary of analytical features observed in selected organ extracts. Free SA and *p*-HBA were measured by targeted LC-MS/MS. Other labels represent confidence-qualified LC-HRMS formula assignments or tentative structural candidates, as specified in Table 2 and Supplementary Table S2. The schematic summarizes observations from the selected specimens and does not establish organ specificity, species-wide distributions, pathway activity, or statistically significant differences. The absence of a label indicates non-detection under the applied workflow rather than confirmed biological absence.

Figure 1 summarizes the targeted LC–MS/MS results and confidence-qualified LC–HRMS candidate features observed in the selected organ extracts. Free SA and *p*-HBA were measured by targeted LC–MS/MS, whereas the remaining labels represent formula-level assignments or tentative structural candidates. Within the limited set of specimens, free SA was detected in selected flower, leaf, and stem extracts but not in the analyzed root extracts. The schematic is descriptive and does not establish organ specificity, species-wide distributions, pathway activity, or statistically significant differences.

Within the limited set of sampled specimens, free SA was detected in selected flower, leaf, and stem extracts but was not detected in the analyzed root extracts. These observations describe the analyzed samples only and do not establish characteristic organ-level or interspecific distributions.

### 2.4. Population Variability in 80 Flowers

Free SA concentrations varied markedly among the 80 individual *Echinacea purpurea* flower heads (Table 3). The mean ± SD was 4.72 ± 13.77 μg/g fresh weight. The values ranged from not detected to 121.48 μg/g. The interquartile range was 4.46 μg/g. This indicated that the central 50% of observations occupied a much narrower interval than the overall dataset. The resulting dataset displayed a markedly right-skewed distribution (skewness = 7.95) and substantial variability among individual flower heads.

The histogram of binned concentrations showed that most flowers fell in the 0–2 μg/g range, with progressively fewer in higher bins and a small number of flowers exceeding 8 μg/g. The upper-tail values demonstrate that individual flower heads can contain substantially more free SA than the sample mean. However, no composite prepared from the same 80 flowers was analyzed. The magnitude of a pooling effect therefore remains unknown [44,50].

### 2.5. DNA Barcoding

DNA sequencing of the chloroplast *trnH–psbA* intergenic spacer generated high-quality sequences ranging from approximately 300 to 510 base pairs across all analyzed *Echinacea* leaf samples. BLASTN analysis against the NCBI Core Nucleotide database returned multiple high-scoring alignments to *Echinacea* plastid reference sequences for each lot. Sequence identities ranged from approximately 95% to 100%. E-values were zero or near zero, indicating strong similarity to database reference sequences. For each sample, the highest-scoring matches corresponded exclusively to species within the genus *Echinacea*, with no evidence of non-*Echinacea* plant contamination detected.

Based on best BLAST match scores and sequence identity, botanical assignments differed among the analyzed lots. One lot was most closely matched to *Echinacea purpurea*, with 100% sequence identity to multiple *E. purpurea* plastid genome and *psbA–trnH* reference sequences. The second lot showed highest similarity to *E. pallida*, with approximately 95% sequence identity across aligned regions. The third lot produced equivalent top matches to *E. tennesseensis* and *E. speciosa*. The amplified barcode region therefore did not uniquely resolve these taxa. Multiple *Echinacea* species produced similarly high-scoring alignments. This result illustrates the limited species-level resolution of the single *trnH–psbA* marker in this dataset. See Supplementary Figures S1–S3 for representative data.

## 3. Discussion

### 3.1. Endogenous Salicylate Metabolism in Echinacea

The present study provides evidence that salicylate-related metabolites occur endogenously in *Echinacea* tissues and are embedded within a broader secondary-metabolite network. Targeted LC–MS/MS showed that free SA was detected primarily in aerial and reproductive specimens, while root specimens were negative for free SA under the targeted method (Table 1). Even with the limited number of samples, this organ-associated observation in the sampled specimens may reflect the known biology of SA as a defense-associated plant hormone [38,51], because aerial tissues and flowers are exposed to diverse environmental challenges, including microbial contact, herbivory, mechanical damage, and developmental stress [52]. The absence of free SA in root specimens by LC–MS/MS does not imply the absence of salicylate metabolism, but it may reflect that free SA accumulation could be tissue-selective under the conditions sampled [53,54].

The LC–HRMS data extend this interpretation by showing that salicylate-associated features co-occurred with conjugated and pathway-related annotations, including SA glucoside/glucose-ester isomers, gentisic acid or related dihydroxybenzoic acids, chorismate/isochorismate-related features, and putative I9G or I9G coproduct features (Table 2). Because these HRMS features were not all confirmed with authentic standards, they are best interpreted as confidence-qualified pathway-level annotations rather than definitive structural identifications. Nevertheless, the co-detection of multiple salicylate-related features is consistent with the hypothesis that SA occurs within an endogenous metabolic context rather than as an isolated analyte [14,15,55].

### 3.2. Free SA Accumulation Differs from Broader Salicylate Pathway Signatures

One of the key biological observations is that the detected pattern for free SA showed a more restricted tissue distribution among the analyzed organ extracts than several HRMS-detected salicylate-associated features (Figure 1). Root specimens were consistently negative for free SA by targeted LC–MS/MS, yet HRMS profiling detected selected conjugated or pathway-related features in those specimens (Table 2). This suggests that the analyzed root specimens may contain salicylate-related precursors, conjugates, or downstream metabolites without accumulating free SA above the detection threshold of the targeted method. Such a pattern may reflect the dynamic regulation of plant hormone metabolism, in which biosynthesis, conjugation, storage, transport, turnover, and release are controlled separately [56].

Mechanistically, the measured free SA pool represents the net balance among biosynthesis, conjugation, deconjugation, transport, compartmentation, and degradation. The detection of free SA in selected aerial extracts but not in the analyzed roots could therefore reflect differences in any of these processes rather than differences in biosynthesis alone. For example, roots may maintain a lower free SA pool through glycosylation, sequestration, export, or rapid turnover, whereas selected aerial or reproductive tissues may accumulate free SA when synthesis or deconjugation exceeds its removal. The candidate salicylate conjugate features observed by LC–HRMS are compatible with this homeostatic model. However, because most related features were not confirmed with authentic standards and the study did not measure conjugated SA concentrations, transport, enzyme activity, gene expression, or metabolic flux, the relative contributions of these mechanisms remain unresolved.

This distinction is particularly relevant for SAG and SGE. In model systems, SAG is commonly described as a vacuolar storage form of SA [55,57], whereas SGE and related conjugates contribute to cytosolic or transport-associated pools [14,58]. Detection of these features in *Echinacea* suggests the presence of salicylate conjugation capacity, but individual SAG and SGE assignments should remain conservative because these isomers share precursor masses and related salicylate-derived product ions.

The putative detection of I9G-related features is also biologically informative but should be interpreted with appropriate caution. I9G is central to the isochorismate-derived SA pathway in *Arabidopsis*, where PBS3 converts isochorismate to I9G and EPS1 promotes its conversion to SA and a glutamate-derived coproduct [10,59,60]. Features consistent with I9G raise the hypothesis that an isochorismate-related route may occur in *Echinacea* [9,15], but direct pathway confirmation would require authentic standards, targeted MS/MS, isotope labeling, enzyme assays, or transcript analysis, which could be the focus of future studies.

### 3.3. Salicylates Occur Within a Broader Phenylpropanoid and Flavonoid Network

The expanded HRMS dataset places salicylate-related metabolism in the context of the broader *Echinacea* secondary-metabolite profile. Confidence-qualified features corresponding to caffeic acid derivatives and phenylpropanoid-related metabolites, including caftaric acid, chlorogenic acid isomers, cichoric acid, dicaffeoylquinic acids, caffeoylshikimic acid, ferulic acid, sinapic acid derivatives, and sinapoyl conjugates, were detected among the analyzed extracts across species and tissues (Table 2). Flavonoid-related features, including apigenin, kaempferol, luteolin, quercetin, and their glycosides, were also observed (Table 2). These results are consistent with the established phytochemical identity of *Echinacea*, in which caffeic acid derivatives and flavonoids are major components of the phenylpropanoid-derived metabolome [18,61].

This broader context is important because salicylate pathway metabolites are likely to reflect plant defense physiology rather than function as isolated chemical markers [14]. Phenylpropanoid metabolism contributes to structural defense, antioxidant capacity, pigmentation, UV protection, pathogen responses, and signaling interactions [62,63,64,65,66]. Salicylate metabolism intersects with this network through shared aromatic precursors and stress-responsive regulation [8]. The co-occurrence of salicylate-associated candidates with CAD- and flavonoid-related features is consistent with a broader defense-associated specialized-metabolite context [67]. One possible mechanism is shared regulation or allocation through aromatic amino acid and phenylpropanoid metabolism. However, co-detection alone does not demonstrate coordinated pathway regulation or active defense responses in the sampled plants. The organ-level visualization in Figure 1 highlights this relationship by showing that the analyzed organ extracts contained different combinations of candidate salicylate- and phenylpropanoid-related features.

### 3.4. Natural Variability of SA

**Figure 2:**
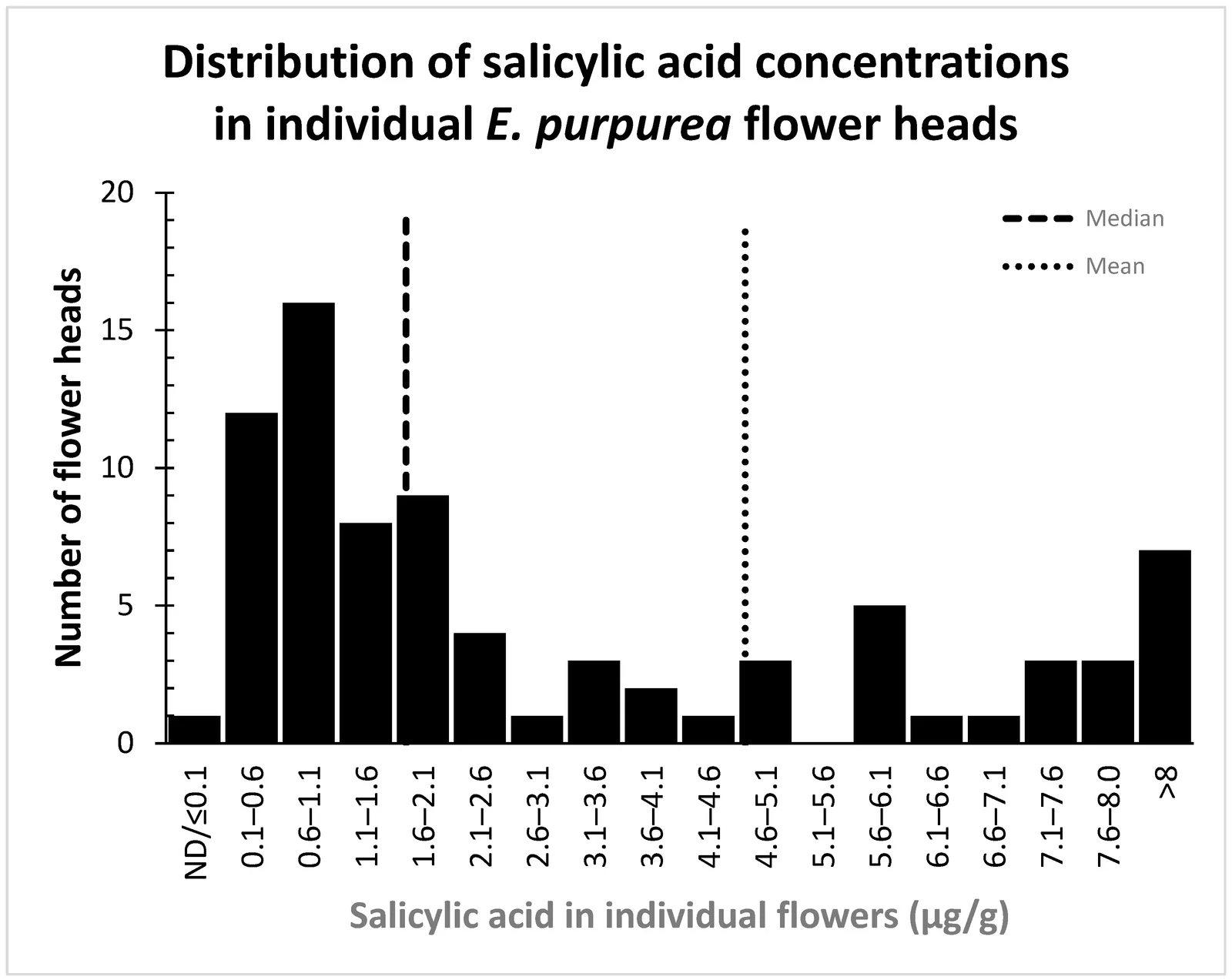
The distribution of salicylic acid concentrations in 80 individual *Echinacea purpurea* flower heads. Bars show the number of flower heads within each concentration interval. Non-detection was assigned a value of 0 μg/g for descriptive plotting and included in the ND/≤0.1 μg/g bin. The distribution was right-skewed, with most flower heads containing low salicylic acid concentrations and a smaller number showing elevated values. Mean ± SD = 4.72 ± 13.77 μg/g; median = 1.75; range = 0–121.48 μg/g.

Analysis of 80 individual *E. purpurea* flower heads revealed substantial natural variability in SA concentration. The distribution was strongly right-skewed, with most flowers containing low concentrations and a small subset showing much higher values (Figure 2). At the level of SA homeostasis, the upper-tail observations could reflect transient conditions in which local SA synthesis or deconjugation exceeded conjugation, export, and degradation. Developmental variation and localized environmental or biotic exposure are alternative explanations, but these factors were not measured. This pattern is typical of inducible plant defense metabolites, which often vary according to developmental stage, microenvironment, localized herbivory, microbial exposure, or other stress conditions [34].

The high-SA flowers are biologically informative because reproductive tissues are exposed to diverse external interactions, including pollinators, microbes, and herbivores. While pathogen exposure, defense activation, gene expression, hormone signaling, enzyme activity, and metabolic flux were not measured, elevated SA in individual flower heads may reflect developmental variation, environmental heterogeneity, differences in SA synthesis, conjugation, deconjugation, or possible localized activation of defense-associated metabolism [40,68,69]. The 80-flower dataset also illustrates potential biologically meaningful variability that may be masked in composite tissue samples.

### 3.5. Applied Implications and Limitations

Although this study is primarily framed around endogenous plant metabolism and defense physiology, the findings also have practical relevance for botanical raw material characterization. *Echinacea* materials are used in herbal and dietary products, and their chemical profiles are influenced by species, organ, harvest stage, and environmental conditions [18,70,71]. The present data confirm endogenous free SA and identify additional tentative salicylate-associated features. Therefore, detection of SA in *Echinacea* materials or extracts should be interpreted in the context of endogenous salicylate metabolism and organ-level variability.

On the other hand, several limitations of this study should be noted. Because the analytical extracts were not dried and weighed, gravimetric extraction yields could not be determined. The HRMS peak areas are relative, non-calibrated signal intensities and should not be interpreted as absolute concentrations or compared quantitatively across chemically distinct metabolite classes. Several annotations, including isomeric SA glucoside/glucose-ester features and putative I9G-related features, remain pathway-level assignments rather than fully confirmed structures. Because of limited biological replication, the organ-level comparison was exploratory, while the 80-flower dataset was collected from a single field population and harvest window. Pooled quality control samples, compound-specific recovery, matrix effect assessment, and calibrated response factors were not available for the retrospective LC-HRMS workflow. Accordingly, the results are interpreted qualitatively, and non-detection should not be considered proof of biological absence. Also, the observed patterns may reflect individual plants, developmental state, cultivation conditions, sampling, or analytical variation.

Furthermore, genotype effects were not evaluated because only a limited number of plants were analyzed and genetic variation within the cultivated population was not assessed. Since plant secondary metabolism is strongly influenced by genotype–environment interactions, future studies incorporating multiple seasons, locations, and genetic backgrounds would provide a more comprehensive understanding of the salicylate pathway variability in *Echinacea*. Accordingly, the mechanisms discussed above should be regarded as testable explanations for the observed chemical patterns rather than experimentally demonstrated pathways.

## 4. Materials and Methods

### 4.1. Plant Materials and Sampling

Fresh, whole-plant samples of *Echinacea purpurea*, *E. pallida* and *E. tennesseensis* were obtained from Factors Farms in Armstrong, British Columbia (50°23′42.1″ N, 119°16′01.6″ W) in August 2025. At sampling, the plants were fully mature, flowering-stage plants in their fourth year of cultivation. Botanical identity was assessed by Jan V. Slama, MSc, who has more than 20 years of professional experience in medicinal plant identification and botanical raw material quality assessment. Available roots, stems, leaves, and inflorescences were examined using the key and descriptions in the *Flora of North America* treatment of *Echinacea* [72]. Supplier cultivation records and *trnH–psbA* DNA barcode results provided additional supporting evidence. No herbarium voucher specimens were prepared or deposited. Consequently, the exact source plants cannot be independently re-examined.

Plants were stored at 4–8 °C until analysis. Identity was verified by macroscopic examination and DNA barcoding as described below. Roots, stems, leaves and flowers were sampled from 2 *E. purpurea*, 1 *E. pallida*, and 1 *E. tennesseensis* plant, giving a total of sixteen tissue lots with internal sample codes 119651–119666.

The organ-level survey included two individual *E. purpurea* plants and one individual each of *E. pallida* and *E. tennesseensis*. Each organ extract represented one sampled plant–organ combination. Because biological replication was insufficient for population-level inference, organ and species patterns were evaluated descriptively and should be considered exploratory. Each tissue sample was extracted once; replicate instrumental injections were technical replicates and were not treated as independent biological observations.

For the flower variability study, 80 individual *E. purpurea* flower heads were harvested at full anthesis from the cultivated fields of Factors Farms. Each flower was labeled and refrigerated at 4–8 °C until sample preparation.

### 4.2. Extraction for LC–MS/MS and LC–HRMS

Fresh plant tissues were cut up into pieces smaller than 5 mm prior to analysis. Approximately 0.2 g of tissue was accurately weighed into a 15 mL polypropylene centrifuge tube (Thermo Fisher Scientific, Mississauga, ON, Canada). The sample was extracted with 10 mL of 50% aqueous methanol (HPLC grade, Thermo Fisher Scientific, Mississauga, ON, Canada), *v*/*v*, sonicated for 30 min, and diluted to 20 mL with the same solvent. Consistent with established aqueous methanol plant metabolomics approaches, this solvent composition was used as a fit-for-purpose analytical extraction medium for free SA and broader polar-to-semipolar phytochemical screening [73,74,75].

The liquid extracts were centrifuged at 3500 rpm for 10 min and the supernatant filtered through a 0.45 μm PVDF membrane (Chromatographic Specialties, Brockville, ON, Canada) to obtain the test solution. The same samples were used for both the LC-MS/MS and LC-HRMS experiments. Since liquid extracts were not evaporated to obtain dried extracts, gravimetric extraction yields were not determined. Quantitative results were normalized to the recorded initial fresh tissue mass and expressed as μg/g fresh weight.

Samples were analyzed on a fresh-weight basis and all concentrations reported in Section 3 and the tables are also expressed accordingly unless otherwise indicated.

### 4.3. LC–MS/MS Quantitation of SA and Preservatives

Quantitative analysis of salicylic acid (SA) and preservative panel analytes was performed in accordance with the Taiwan Food and Drug Administration (TFDA) official method “Method of Test for Preservatives in Foods”, with minor adaptations for botanical matrices [76,77]. The LC-MS/MS method covers four acid preservatives (benzoic acid, dehydroacetic acid, *p*-hydroxybenzoic acid (*p*-HBA) and salicylic acid) and seven ester preservatives (methyl, ethyl, isopropyl, propyl, sec-butyl, iso-butyl and butyl *p*-hydroxybenzoate).

Chromatographic separation was performed on a Thermo Ultimate 3400 UHPLC (Thermo, Waltham, MA, USA) with an Agilent Poroshell EC-C18 column (100 mm × 2.1 mm, 2.7 μm, Mississauga, ON, Canada) operated at a flow rate of 0.4 mL/min. Mobile phase A was a 5 mM citrate buffer (Millipore Sigma, Oakville, ON, Canada) and mobile phase B was methanol–acetonitrile (LC-MS grade, Thermo Fisher Scientific, Mississauga, ON, Canada) 1:2, *v*/*v*. The gradient followed Method Condition 1 of the TFDA official method. Samples and standard solutions were kept at 10.0 °C in a Transcend LX autosampler (Thermo, Waltham, MA, USA) and injected at 5.0 μL volumes. Analytes were detected by a Quantiva triple-quadrupole LC–MS/MS system (Thermo, Waltham, MA, USA) operated in multiple reaction monitoring (MRM) mode using HESI negative mode for all analytes except dehydroacetic acid. The precursor/product ion pairs and polarity settings are specified in the TFDA method. Calibration curves for each analyte were prepared from mixed standard solutions (0.002–25.0 μg/mL) as prescribed in the method. Stable isotope internal standards were obtained from Millipore Sigma (Oakville, ON, Canada). These included 4-hydroxybenzoic-2,3,5,6-d_4_ acid, salicylic acid-d_4_, benzoic acid-d_5_, methyl *p*-hydroxybenzoate ^13^C_6_, ethyl *p*-hydroxybenzoate-d_4_, isopropyl *p*-hydroxybenzoate-d_4_, and isobutyl *p*-hydroxybenzoate-d_4_. Quantification was based on analyte-to-internal standard peak area ratios. Matrix effects were not independently quantified using post-extraction fortified matrix extracts and neat standards. Analyte-matched stable-isotope-labeled internal standards, or designated isotope-labeled surrogates where an exact analog was unavailable, were used to compensate for sample preparation and ionization response variability.

Samples were analyzed once by LC-MS/MS unless selected as a periodic replicate at a frequency of one out of every twenty samples. These duplicates monitored analytical repeatability and were not treated as independent extraction or biological replicates. Quality control for the targeted LC–MS/MS analysis included calibration standards, blank injections, stable-isotope internal standards, and one duplicate analysis after every twenty study samples. Duplicate measurements were used to monitor analytical repeatability, while internal standard responses were monitored across the analytical sequence. Analytical performance characteristics, including calibration range, linearity, accuracy, surrogate recovery, injection precision, internal standard response repeatability, and LOD/LOQ information, are reported in Supplementary Table S5.

### 4.4. LC–HRMS Profiling of SA Pathway Metabolites

SA pathway metabolites were determined according to published methods [12,14,78] with minor modifications for HRMS. High-resolution analyses were performed on a Q Exactive Orbitrap mass spectrometer (Thermo, Waltham, MA, USA) equipped with a heated electrospray ionization source and coupled to a Vanquish UHPLC (Thermo, Waltham, MA, USA) system with an Agilent EC-C18 column (100 mm × 2.1 mm, 2.6 μm). Mobile phase A was water with 0.5% formic acid; mobile phase B was acetonitrile. The gradient started at 12% B, was held for 0.5 min, ramped to 27% B at 5.6 min, to 37% B at 8.65 min and to 60% B at 13.8 min, followed by a brief wash at 67% B and re-equilibration.

Full-scan MS data from *m*/*z* 100–600 were acquired at 70,000 resolution in HESI negative mode. A targeted MS/MS inclusion list was used to trigger fragmentation at a normalized collision energy (NCE) of 35. The inclusion list contained ions corresponding to *trans*-cinnamic acid, methyl salicylate, SAG, SGE, I9G, isochorismate, chorismate, and related features. Retention time windows were set at 10% windows. Candidate features were evaluated using accurate mass, isotopic pattern, retention behavior, and diagnostic fragments where available. Formula-level or structural confidence assignments were made, as described in Section 2.5. Feature-specific extracted-ion windows of ±5–15 ppm were used for retrospective screening, as detailed in Supplementary Table S1 using FreeStyle Version 1.6 (Thermo, Waltham, MA, USA). While most features were extracted at ±5 ppm, wider windows of ±8–15 ppm were used selectively for low-*m*/*z* and highly abundant features were not consistently captured using the narrower window due to space charge effects.

The retrospective LC-HRMS workflow was used for qualitative suspect screening and confidence-qualified feature annotation rather than absolute quantification. Each extract was analyzed twice as duplicate instrumental injections. Supplementary Table S4 contains a summary of analytical replications performed in these experiments. Candidate features were retained following manual review of chromatographic peak shape, retention behavior, accurate mass, isotope pattern, plausible adduct assignment, and MS/MS evidence where available. Agreement between duplicate runs was evaluated based on feature detection, retention time, and precursor mass. Because compound-specific recovery, matrix effects, and calibration were not established for this retrospective workflow, LC-HRMS peak areas were treated as uncalibrated relative signals and were not used for absolute quantification or direct comparison among chemically different metabolites.

### 4.5. Metabolite Identification and Confidence Assignment

Annotation confidence was reported using the Metabolomics Standards Initiative (MSI) framework. Level 1 identifications required agreement with an authentic reference standard using at least two orthogonal properties (e.g., retention time and MS/MS fragmentation) under identical conditions. Putative structure-level annotations required accurate-mass and supporting MS/MS evidence but lacked complete authentic-standard confirmation. Features supported only by accurate mass, isotope pattern, and plausible adduct assignment were reported as molecular formula assignments or tentative structural candidates rather than confirmed metabolites [79]. In this study, salicylic acid (SA) measured by the targeted LC–MS/MS workflow was treated as confirmed where quantified within the validated preservative panel method (Table 1), whereas salicylate pathway intermediates and conjugates detected by LC–HRMS were treated as putatively annotated features (Table 2) because several signals were derived from retrospective accurate-mass extraction and were not supported by authentic standards for every feature. Accordingly, HRMS results are presented as relative signal intensities and co-occurrence patterns among candidate features, rather than absolute concentrations. Absolute quantification and higher-confidence confirmation for metabolites such as SAG, SGE, I9G, and MeSA would require authentic standards and isotopically labeled internal standards that were outside the scope of this work.

### 4.6. DNA Extraction, Barcoding and BLAST Analysis

Genomic DNA was extracted from fresh plant materials with a commercial plant genomic DNA purification kit that uses magnetic beads according to the manufacturer’s instructions (MagaBio Plus, Bioer Technology, Hangzhou, China). The DNA barcode region spanning the *trnH–psbA* intergenic spacer was amplified using generic *trnH–psbA* primers. PCR amplification was conducted using a SimpliAmp (Thermo, Waltham, MA, USA) thermal cycler, and sequencing was performed on a SeqStudio genetic analyzer (Thermo, Waltham, MA, USA).

Resulting nucleotide sequences (approximately 300–510 bp, depending on sample) were queried against the NCBI Core Nucleotide database using BLAST version 2.17.0+. Sequence alignment and similarity scoring were performed using the BLAST greedy alignment algorithm, and database indexing followed the MegaBLAST production search framework. Taxonomic similarity was assessed based on the highest sequence identity and alignment score among *Echinacea* reference sequences. For each lot, multiple *Echinacea* plastid genomes and *psbA–trnH* reference sequences were returned with high sequence similarity, and the best match was reported as the taxonomic call.

### 4.7. Statistical Analysis

No formal statistical tests were performed for organ or species comparisons because the design included only two *E. purpurea* plants and one plant for each of the other species. These observations were therefore summarized descriptively and were not used to infer population-level tissue or species differences.

Descriptive statistics for SA concentrations in the 80-flower dataset were calculated in Microsoft Excel Version 2606 (Build 20131.20152). The arithmetic mean, median, minimum, maximum, sample standard deviation, skewness, and interquartile range were calculated using AVERAGE, MEDIAN, MIN, MAX, STDEV.S, SKEW, and QUARTILE.INC, respectively. Shapiro–Wilk *W* and *p* were calculated with the Real Statistics Resource Pack software (Release 9.8, Charles Zaiontz, real-statistics.com), copyright (2013–2026). Single non-detection was assigned 0 μg/g for the descriptive summaries, histogram, and Shapiro–Wilk analysis. For descriptive visualization, histograms were generated using 0.5 μg/g bin widths with an open-ended > 8 μg/g category to preserve resolution among the majority of low-concentration observations while preventing the single maximum value of 121.48 μg/g from compressing the central distribution. Potential unusual values were checked against original sample identifiers, chromatograms, integration boundaries, calibration status, internal standard response, preparation records, and transcription records. No value was excluded solely because of its magnitude. The maximum value was retained only after all records were confirmed. No outlier exclusion was applied; all 80 measurements were retained to reflect true biological variability.

### 4.8. Generative Artificial Intelligence Usage

During the preparation of this manuscript, OpenAI ChatGPT was used to assist with language editing and manuscript organization. OpenAI image generation tools were also used to create schematic botanical illustrations and graphical abstract elements. These illustrations were used only as conceptual visual aids and not as taxonomic evidence, analytical data, or primary research results. All AI-assisted text and AI-generated images were reviewed, edited, and verified by the authors for scientific accuracy. The authors take full responsibility for the content of this manuscript.

## 5. Conclusions

This study provides evidence for free SA and salicylate-related features in selected *Echinacea* specimens. Targeted LC–MS/MS confirmed low-level free SA in selected aerial and reproductive tissues and showed that other preservative panel analytes were not detected. Retrospective LC–HRMS screening yielded putatively annotated salicylate-associated, phenylpropanoid/caffeic acid derivative, and flavonoid-related features in the analyzed extracts, placing confirmed free SA within a broader specialized-metabolite context. Analysis of 80 individual *E. purpurea* flower heads further showed marked natural variability in free SA concentrations. Together, these findings support the endogenous occurrence of free SA in the analyzed *Echinacea* specimens and provide an exploratory framework for future studies of salicylate biology, phenylpropanoid metabolism, and botanical raw material characterization. Because the organ-level survey included limited biological replication, the observed patterns should not be considered representative of the species or interpreted as established tissue-specific or interspecific differences. Larger studies using independently sampled plants across genotypes, developmental stages, harvest periods, and environments are required to validate these preliminary observations.

## Figures and Tables

**Table 1:** Quantitative concentrations of salicylic acid (SA) and *p*-hydroxybenzoic acid (*p*-HBA) in flowers, leaves, roots, and stems of *E. purpurea*, *E. pallida*, and *E. tennesseensis* determined by LC-MS/MS. A total of 16 tissue samples (sample IDs 119651–119666) were analyzed individually. Concentrations are reported as μg/g fresh weight (FW).

	SA	*p*-HBA
*Echinacea pallida*		
Flowers		
119666	0.240	2.098
Leaves		
119665	0.060	0.620
Roots		
119663	ND	ND
Stems		
119664	ND	ND
*Echinacea purpurea*		
Flowers		
119654	0.350	ND
119658	1.630	ND
Leaves		
119653	ND	ND
119657	ND	ND
Roots		
119651	ND	ND
119655	ND	ND
Stems		
119652	0.109	ND
119656	0.185	1.061
*Echinacea tennesseensis*		
Flowers		
119662	0.070	ND
Leaves		
119661	0.059	ND
Roots		
119659	ND	ND
Stems		
119660	ND	ND

Benzoic acid, dehydroacetic acid, and seven paraben preservatives were included in the analytical method but were not detected in any sample. Values are reported to three decimal places to reflect the analytical reporting format and should not be interpreted as equivalent biological precision. Values are shown for SA and *p*-hydroxybenzoic acid (*p*-HBA). ND indicates that the analyte response was below the applicable detection limit.

**Table 2:** Qualitative summary of retrospectively screened LC–HRMS features in selected Echinacea organ extracts.

Species/Tissue	Candidate Salicylate-Associated Features	Candidate Phenylpropanoid/CAD-Related Features	Candidate Flavonoid-Related Features	Descriptive Summary
*Echinacea pallida*				
Flowers	SA-5Glc, SAG, *p*-HBA	5-CQA, CaftA, ChicA, SinGlc, Z-SinA, Z-SinMal	Kae-diGlc	CAD/phenylpropanoid-rich profile with co-detected salicylate features.
Leaves	SA-5Glc, SAG	ChicA, SinGlc, Z-SinMal	Kae-Glc, Kae-diGlc	CAD/phenylpropanoid-rich profile with co-detected salicylate features.
Stems	I9G, SA-5Glc, SAG	3-CQA, Caff-Shik, ChicA, SinGlc, Z-SinMal	None detected	CAD/phenylpropanoid-rich profile with co-detected salicylate features.
Roots	GA, IC, SA-5Glc, SAG	3-CQA, 5-CQA, CaftA, ChicA, E-SinA, SinGlc, Z-SinMal, cis-5-CQA	Kae-Glc, Kae-diGlc	Pathway/conjugate features detected; free SA was ND by targeted LC–MS/MS.
*Echinacea purpurea*				
Flowers	CHA, I9G, IC, Preph	3,4-diCQA, 3,5-diCQA, 3-CQA, 4,5-diCQA, 5-CQA, Caff-Shik, CaftA, ChicA, E-SinMal, SinGlc, Z-SinA, cis-5-CQA	Api, Api-Glc, Api-diGlc, Kae, Kae-Glc, Kae-diGlc, Lut, Lut-Glc, Lut-diGlc, Que	Flavonoid-rich profile with accompanying salicylate and phenylpropanoid features.
Leaves	CHA, I9G, IC	3,4-diCQA, 3,5-diCQA, 3-CQA, 4,5-diCQA, 4-CQA, 5-CQA, Caff-Shik, CaftA, ChicA, FerA, SinGlc, Z-SinMal, cis-5-CQA	Api, Api-Glc, Api-diGlc, Kae, Kae-diGlc, Lut, Lut-Glc, Lut-diGlc, Que	Flavonoid-rich profile with accompanying salicylate and phenylpropanoid features.
Stems	2,3-DHBA, CHA, I9G, Preph, SA-5Glc, SAG	3,4-diCQA, 3,5-diCQA, 3-CQA, 4,5-diCQA, 4-CQA, 5-CQA, CaftA, ChicA, E-SinA, E-SinMal, SinGlc, Z-SinA, Z-SinMal, cis-5-CQA	Api-diGlc, Kae-Glc, Kae-diGlc	CAD/phenylpropanoid-rich profile with co-detected salicylate features.
Roots	GA	5-CQA, Caff-Shik, E-SinA, E-SinMal, FerA, SinGlc, Z-SinA, Z-SinMal	Kae-Glc, Kae-diGlc	Candidate salicylate-associated features observed; free SA not detected by targeted LC–MS/MS.
*Echinacea tennesseensis*				
Flowers	None detected	5-CQA, CaftA, SinGlc	None detected	Selected CAD-related features detected/no salicylate-associated features retained.
Leaves	MeSA	5-CQA, CaftA, ChicA, FerA, SinGlc, Z-SinMal	None detected	CAD/phenylpropanoid-rich profile with co-detected salicylate features.
Stems	I9G	5-CQA, CaftA, FerA, SinGlc, Z-SinA	None detected	CAD/phenylpropanoid-rich profile with co-detected salicylate features.
Roots	CHA, I9G, IC	3,4-diCQA, 3,5-diCQA, 3-CQA, 4,5-diCQA, 5-CQA, Caff-Shik, FerA, SinGlc, cis-5-CQA	Api, Api-Glc, Api-diGlc, Kae, Kae-diGlc, Lut, Lut-Glc, Lut-diGlc, Que	Candidate salicylate-associated features observed; free SA not detected by targeted LC–MS/MS.

Metabolites in this table represent tentative candidates or putatively annotated LC-HRMS features based on accurate mass, retention behavior, isotopic pattern, and MS/MS evidence where available. Feature-level evidence and annotation confidence are provided in Supplementary Table S2. Abbreviations are defined in the Abbreviations Section.

**Table 3:** Summary statistics for salicylic acid (SA) in 80 individual *Echinacea purpurea* flower heads. Samples were collected from one field population during one harvest window. Concentrations are reported as μg/g fresh weight (FW).

Metric	Value
Number of flowers (*n*)	80
Mean SA in raw flowers (μg/g, fresh weight)	4.72
Median (μg/g)	1.75
Standard deviation (μg/g)	13.77
Range in raw flowers (min–max, μg/g)	0.00–121.48
Interquartile range (μg/g)	4.46
Skewness	7.95
Shapiro–Wilk *W*	0.25
*p*	<0.001

Summary statistics are reported to two decimal places.

## Data Availability

Data are contained within the article and Supplementary Materials.

## Supplementary Materials

The following supporting information can be downloaded at https://www.mdpi.com/article/10.3390/molecules31172963/s1.

## Abbreviations

The following abbreviations are used in this manuscript:

2,3-DHBA	2,3-Dihydroxybenzoic acid
3-CQA	3-Caffeoylquinic acid
3,4-diCQA	3,4-Dicaffeoylquinic acid
3,5-diCQA	3,5-Dicaffeoylquinic acid
4-CQA	4-Caffeoylquinic acid
4,5-diCQA	4,5-Dicaffeoylquinic acid
5-CQA	5-Caffeoylquinic acid (chlorogenic acid)
Api	Apigenin
Api-Glc	Apigenin glucoside
Api-diGlc	Apigenin diglucoside
BA	Benzoic acid
BLASTN	Basic Local Alignment Search Tool (nucleotide)
CAD	Caffeic acid derivatives
Caff-Shik	Caffeoylshikimic acid
CaftA	Caftaric acid
CHA	Chorismate
ChicA	Cichoric acid
cis-5-CQA	cis-5-Caffeoylquinic acid
diGlc	Diglucoside
E-SinA	*E*-Sinapic acid
E-SinMal	*E*-Sinapoyl malate
EDS5	Enhanced Disease Susceptibility 5
EPS1	Enhanced Pseudomonas Susceptibility 1
FerA	Ferulic acid
GA	Gentisic acid (2,5-dihydroxybenzoic acid)
Glc	Glucoside
HESI	Heated electrospray ionization
HRMS	High-resolution mass spectrometry
IC	Isochorismate
ICS1	Isochorismate synthase 1
I9G	Isochorismate-9-glutamate
Kae	Kaempferol
Kae-Glc	Kaempferol glucoside
Kae-diGlc	Kaempferol diglucoside
LC–HRMS	Liquid chromatography–high-resolution mass spectrometry
LC–MS/MS	Liquid chromatography–tandem mass spectrometry
Lut	Luteolin
Lut-Glc	Luteolin glucoside
Lut-diGlc	Luteolin diglucoside
MeSA	Methyl salicylate
MRM	Multiple reaction monitoring
MSI	Metabolomics Standards Initiative
NCE	Normalized collision energy
ND	Not detected
PAL	Phenylalanine ammonia-lyase
*p*-HBA	*p*-Hydroxybenzoic acid
PBS3	AvrPphB Susceptible 3
Preph	Prephenate
Que	Quercetin
SA	Salicylic acid
SAG	Salicylic acid 2-O-β-D-glucoside
SGE	Salicylic acid glucose ester
SinGlc	Sinapic acid glucoside
TFDA	Taiwan Food and Drug Administration
*trnH–psbA*	Chloroplast *trnH–psbA* intergenic spacer
UHPLC	Ultra-high-performance liquid chromatography
Z-SinA	*Z*-Sinapic acid
Z-SinMal	*Z*-Sinapoyl malate

## References

[1] Li A., Sun X., Liu L. (2022). Action of Salicylic Acid on Plant Growth. Front. Plant Sci..

[2] Janda T., Gondor K.O., Pál M., Szalai G., Aftab T., Yusuf M. (2021). Salicylic Acid Signalling Under Stress Conditions in Plants. Jasmonates and Salicylates Signaling in Plants.

[3] Rivas-San Vicente M., Plasencia J. (2011). Salicylic Acid beyond Defence: Its Role in Plant Growth and Development. J. Exp. Bot..

[4] Caarls L., Van der Does D., Hickman R., Jansen W., Verk M.C.V., Proietti S., Lorenzo O., Solano R., Pieterse C.M.J., Van Wees S.C.M. (2017). Assessing the Role of ETHYLENE RESPONSE FACTOR Transcriptional Repressors in Salicylic Acid-Mediated Suppression of Jasmonic Acid-Responsive Genes. Plant Cell Physiol..

[5] Fuster V., Sweeny J.M. (2011). Aspirin: A Historical and Contemporary Therapeutic Overview. Circulation.

[6] Alegbeleye B.J., Akpoveso O.-O.P., Mohammed R.K., Asare B.Y.-A. (2020). Pharmacology, Pharmaceutics and Clinical Use of Aspirin: A Narrative Review. J. Drug Deliv. Ther..

[7] Klessig D.F., Tian M., Choi H.W. (2016). Multiple Targets of Salicylic Acid and Its Derivatives in Plants and Animals. Front. Immunol..

[8] Lefevere H., Bauters L., Gheysen G. (2020). Salicylic Acid Biosynthesis in Plants. Front. Plant Sci..

[9] Rekhter D., Lüdke D., Ding Y., Feussner K., Zienkiewicz K., Lipka V., Wiermer M., Zhang Y., Feussner I. (2019). Isochorismate-Derived Biosynthesis of the Plant Stress Hormone Salicylic Acid. Science.

[10] Chen J. (2024). Arabidopsis ICS1 Produces Salicylic Acid Precursor Isochorismate through a Gated Channel Mechanism. Plant Physiol..

[11] Torrens-Spence M.P., Bobokalonova A., Carballo V., Glinkerman C.M., Pluskal T., Shen A., Weng J.-K. (2019). PBS3 and EPS1 Complete Salicylic Acid Biosynthesis from Isochorismate in Arabidopsis. Mol. Plant.

[12] George Thompson A.M., Iancu C.V., Neet K.E., Dean J.V., Choe J. (2017). Differences in Salicylic Acid Glucose Conjugations by UGT74F1 and UGT74F2 from *Arabidopsis thaliana*. Sci. Rep..

[13] Allasia V., Industri B., Ponchet M., Quentin M., Favery B., Keller H. (2018). Quantification of Salicylic Acid (SA) and SA-Glucosides in *Arabidopsis thaliana*. Bio-Protocol.

[14] Berim A., Gang D.R. (2022). Accumulation of Salicylic Acid and Related Metabolites in *Selaginella moellendorffii*. Plants.

[15] Mishra A.K., Baek K.-H. (2021). Salicylic Acid Biosynthesis and Metabolism: A Divergent Pathway for Plants and Bacteria. Biomolecules.

[16] Zhang Q.-L., Liu T.-J., Wang Y.-B., Feng T., Chen L., Xiao B.-Z., Li H.-X., Tan H.-B., Huang H.-R., Ge X.-J. (2026). Evolution and Diversification of PAL-Mediated Salicylic Acid Biosynthesis in Rosaceae. Hortic. Res..

[17] Tahmasebi A., Ebrahimie E., Pakniyat H., Ebrahimi M., Mohammadi-Dehcheshmeh M. (2019). Insights from the *Echinacea purpurea* (L.) Moench Transcriptome: Global Reprogramming of Gene Expression Patterns towards Activation of Secondary Metabolism Pathways. Ind. Crops Prod..

[18] Fu R., Zhang P., Deng Z., Jin G., Guo Y., Zhang Y. (2021). Diversity of Antioxidant Ingredients among *Echinacea* Species. Ind. Crops Prod..

[19] Hou C.-C., Chen C., Yang N.-S., Chen Y.-P., Lo C.-P., Wang S.-Y., Tien Y.-J., Tsai P.-W., Shyur L.-F. (2009). Comparative Metabolomics Approach Coupled with Cell- and Gene-Based Assays for Species Classification and Anti-Inflammatory Bioactivity Validation of *Echinacea* Plants. J. Nutr. Biochem..

[20] Sabra A., Adam L., Daayf F., Renault S. (2012). Salinity-Induced Changes in Caffeic Acid Derivatives, Alkamides and Ketones in Three *Echinacea* Species. Environ. Exp. Bot..

[21] Tsai Y.-L., Chiou S.-Y., Chan K.-C., Sung J.-M., Lin S.-D. (2012). Caffeic Acid Derivatives, Total Phenols, Antioxidant and Antimutagenic Activities of *Echinacea purpurea* Flower Extracts. LWT—Food Sci. Technol..

[22] Senchina D.S., McCann D.A., Flinn G.N., Wu L., Zhai Z., Cunnick J.E., Wurtele E.S., Kohut M.L. (2009). *Echinacea tennesseensis* Ethanol Tinctures Harbor Cytokine- and Proliferation-Enhancing Capacities. Cytokine.

[23] Ahmadi F. (2024). Phytochemistry, Mechanisms, and Preclinical Studies of *Echinacea* Extracts in Modulating Immune Responses to Bacterial and Viral Infections: A Comprehensive Review. Antibiotics.

[24] Goel V., Lovlin R., Barton R., Lyon M.R., Bauer R., Lee T.D.G., Basu T.K. (2004). Efficacy of a Standardized *Echinacea* Preparation (EchinilinTM) for the Treatment of the Common Cold: A Randomized, Double-Blind, Placebo-Controlled Trial. J. Clin. Pharm. Ther..

[25] Chen H., Pan B., Zhang S., Li X., Zhang Y., Gao K., Chen D., Wang L., Jiang T., Luo C. (2025). Recent Advances in Biosynthesis and Bioactivity of Plant Caffeoylquinic Acids. Curr. Issues Mol. Biol..

[26] Ahmadi F., Kariman K., Mousavi M., Rengel Z. (2024). *Echinacea*: Bioactive Compounds and Agronomy. Plants.

[27] Zhu B., Zhang Y., Gao R., Wu Z., Zhang W., Zhang C., Zhang P., Ye C., Yao L., Jin Y. (2025). Complete Biosynthesis of Salicylic Acid from Phenylalanine in Plants. Nature.

[28] Yu J., Xie J., Sun M., Xiong S., Xu C., Zhang Z., Li M., Li C., Lin L. (2024). Plant-Derived Caffeic Acid and Its Derivatives: An Overview of Their NMR Data and Biosynthetic Pathways. Molecules.

[29] Tan L., Liu L., Liu J., Fu Y., Zhang Y., Sun C., Zhang Y. (2025). Integrated Transcriptomic and Metabolomic Analysis Reveals Biochar-Induced Enhancement of Growth and Secondary Metabolism in the Medicinal Plant *Echinacea purpurea*. Int. J. Mol. Sci..

[30] Rozhon W., Petutschnig E., Wrzaczek M., Jonak C. (2005). Quantification of Free and Total Salicylic Acid in Plants by Solid-Phase Extraction and Isocratic High-Performance Anion-Exchange Chromatography. Anal. Bioanal. Chem..

[31] Amara A., Frainay C., Jourdan F., Naake T., Neumann S., Novoa-del-Toro E.M., Salek R.M., Salzer L., Scharfenberg S., Witting M. (2022). Networks and Graphs Discovery in Metabolomics Data Analysis and Interpretation. Front. Mol. Biosci..

[32] Pang Z., Chen J., Wang T., Gao C., Li Z., Guo L., Xu J., Cheng Y. (2021). Linking Plant Secondary Metabolites and Plant Microbiomes: A Review. Front. Plant Sci..

[33] Divekar P.A., Narayana S., Divekar B.A., Kumar R., Gadratagi B.G., Ray A., Singh A.K., Rani V., Singh V., Singh A.K. (2022). Plant Secondary Metabolites as Defense Tools against Herbivores for Sustainable Crop Protection. Int. J. Mol. Sci..

[34] Palazon J., Alcalde M.A. (2025). Secondary Metabolites in Plants. Plants.

[35] Hama J.R., Laursen B.B., Fomsgaard I.S., Vestergård M. (2026). Transfer of Chemical Defence from Rye to Clover Is Associated with Enhanced Clover Resistance against Root-Knot Nematodes. New Phytol..

[36] Malm L., Palm E., Souihi A., Plassmann M., Liigand J., Kruve A. (2021). Guide to Semi-Quantitative Non-Targeted Screening Using LC/ESI/HRMS. Molecules.

[37] Reddy P., Plozza T., Ezernieks V., Stefanelli D., Scalisi A., Goodwin I., Rochfort S. (2022). Metabolic Pathways for Observed Impacts of Crop Load on Floral Induction in Apple. Int. J. Mol. Sci..

[38] Fresno D.H., Munné-Bosch S. (2021). Differential Tissue-Specific Jasmonic Acid, Salicylic Acid, and Abscisic Acid Dynamics in Sweet Cherry Development and Their Implications in Fruit-Microbe Interactions. Front. Plant Sci..

[39] Zhan X., Chen Z., Chen R., Shen C. (2022). Environmental and Genetic Factors Involved in Plant Protection-Associated Secondary Metabolite Biosynthesis Pathways. Front. Plant Sci..

[40] Salam U., Ullah S., Tang Z.-H., Elateeq A.A., Khan Y., Khan J., Khan A., Ali S. (2023). Plant Metabolomics: An Overview of the Role of Primary and Secondary Metabolites Against Different Environmental Stress Factors. Life.

[41] Zhao Y., Liu G., Yang F., Liang Y., Gao Q., Xiang C., Li X., Yang R., Zhang G., Jiang H. (2023). Multilayered Regulation of Secondary Metabolism in Medicinal Plants. Mol. Hortic..

[42] Jangpangi D., Patni B., Chandola V., Chandra S. (2025). Medicinal Plants in a Changing Climate: Understanding the Links between Environmental Stress and Secondary Metabolite Synthesis. Front. Plant Sci..

[43] Zygmunt A.E., Karoń Ł., Karoń K., Grabowski W., Pedrycz D., Drapała G., Pedrycz E. (2024). A Natural Way of Boosting Immunity: The Role of *Echinacea* in Cold Prevention and Treatment—A Literature Review. Qual. Sport.

[44] Binns S.E., Arnason J.T., Baum B.R. (2002). Phytochemical Variation within Populations of *Echinacea angustifolia* (Asteraceae). Biochem. Syst. Ecol..

[45] Jain D., Bisht S., Parvez A., Singh K., Bhaskar P., Koubouris G. (2024). Effective Biotic Elicitors for Augmentation of Secondary Metabolite Production in Medicinal Plants. Agriculture.

[46] Vlasheva M., Katsarova M., Dobreva A., Dzhurmanski A., Denev P., Dimitrova S. (2024). *Echinacea* Species Cultivated in Bulgaria as a Source of Chicoric and Caftaric Acids. Agronomy.

[47] Mei B., Xie H., Xing H., Kong D., Pan X., Li Y., Wu H. (2020). Changes of Phenolic Acids and Antioxidant Activities in Diploid and Tetraploid *Echinacea purpurea* at Different Growth Stages. Rev. Bras. Farmacogn..

[48] Jin G., Deng Z., Wang H., Zhang Y., Fu R. (2024). EpMYB2 Positively Regulates Chicoric Acid Biosynthesis by Activating Both Primary and Specialized Metabolic Genes in Purple Coneflower. Plant J..

[49] Abdoli M., Mehrpooya Z., Talebian M.R. (2021). Effect of Salicylic Acid and Yeast Extract on Caffeic Acid Derivatives Production in *Echinacea purpurea* L.. J. Med. Plants.

[50] Salinas P., Velozo S., Herrera-Vásquez A. (2025). Salicylic Acid Accumulation: Emerging Molecular Players and Novel Perspectives on Plant Development and Nutrition. J. Exp. Bot..

[51] Vigliocco A., Del Bel Z., Pérez-Chaca M.V., Molina A., Zirulnik F., Andrade A.M., Alemano S. (2020). Spatiotemporal Variations in Salicylic Acid and Hydrogen Peroxide in Sunflower Seeds During Transition from Dormancy to Germination. Physiol. Plant..

[52] Nelson A.S., Whitehead S.R. (2021). Fruit Secondary Metabolites Shape Seed Dispersal Effectiveness. Trends Ecol. Evol..

[53] Chen Z., Iyer S., Caplan A., Klessig D.F., Fan B. (1997). Differential Accumulation of Salicylic Acid and Salicylic Acid-Sensitive Catalase in Different Rice Tissues. Plant Physiol..

[54] Spoel S.H., Dong X. (2024). Salicylic Acid in Plant Immunity and Beyond. Plant Cell.

[55] Dempsey D.A., Vlot A.C., Wildermuth M.C., Klessig D.F. (2011). Salicylic Acid Biosynthesis and Metabolism. Arab. Book.

[56] Wong C., Alabadí D., Blázquez M.A. (2023). Spatial Regulation of Plant Hormone Action. J. Exp. Bot..

[57] Dempsey D.A., Klessig D.F. (2017). How Does the Multifaceted Plant Hormone Salicylic Acid Combat Disease in Plants and Are Similar Mechanisms Utilized in Humans?. BMC Biol..

[58] Saleem M., Fariduddin Q., Janda T. (2021). Multifaceted Role of Salicylic Acid in Combating Cold Stress in Plants: A Review. J. Plant Growth Regul..

[59] Chen H., Clinton M., Qi G., Wang D., Liu F., Fu Z.Q. (2019). Connecting the Dots: A New and Complete Salicylic Acid Biosynthesis Pathway. Mol. Plant.

[60] Roychowdhury R., Mishra S., Anand G., Dalal D., Gupta R., Kumar A., Gupta R. (2024). Decoding the Molecular Mechanism Underlying Salicylic Acid (SA)-Mediated Plant Immunity: An Integrated Overview from Its Biosynthesis to the Mode of Action. Physiol. Plant..

[61] Miller S.C. (2004). Echinacea: The Genus Echinacea.

[62] Ramaroson M.-L., Koutouan C., Helesbeux J.-J., Le Clerc V., Hamama L., Geoffriau E., Briard M. (2022). Role of Phenylpropanoids and Flavonoids in Plant Resistance to Pests and Diseases. Molecules.

[63] Korkina L.G. (2007). Phenylpropanoids as Naturally Occurring Antioxidants: From Plant Defense to Human Health. Cell. Mol. Biol..

[64] Nazir M., Tungmunnithum D., Bose S., Drouet S., Garros L., Giglioli-Guivarc’h N., Abbasi B.H., Hano C. (2019). Differential Production of Phenylpropanoid Metabolites in Callus Cultures of *Ocimum basilicum* L. with Distinct In Vitro Antioxidant Activities and In Vivo Protective Effects against UV Stress. J. Agric. Food Chem..

[65] Grace S.C., Logan B.A. (2000). Energy Dissipation and Radical Scavenging by the Plant Phenylpropanoid Pathway. Philos. Trans. R. Soc. B Biol. Sci..

[66] Di X., Hu R., Gao Y., Schaller H., Madhujith T., Hao G., Hemmerlin A., Liao P. (2026). Bridging Metabolic Pathways: Coordinated Regulation of Isoprenoid and Phenylpropanoid Metabolism in Plants. J. Exp. Bot..

[67] Bauters L., Stojilković B., Gheysen G. (2021). Pathogens Pulling the Strings: Effectors Manipulating Salicylic Acid and Phenylpropanoid Biosynthesis in Plants. Mol. Plant Pathol..

[68] Sarabia L.D., Boughton B.A., Rupasinghe T., Van De Meene A.M.L., Callahan D.L., Hill C.B., Roessner U. (2018). High-Mass-Resolution MALDI Mass Spectrometry Imaging Reveals Detailed Spatial Distribution of Metabolites and Lipids in Roots of Barley Seedlings in Response to Salinity Stress. Metabolomics.

[69] Obata T., Fernie A.R. (2012). The Use of Metabolomics to Dissect Plant Responses to Abiotic Stresses. Cell. Mol. Life Sci..

[70] Kocacik A., Yalcin H. (2023). The Effect of Harvest Time on the Volatile Compounds and Bioactive Properties of the Flowers, Leaves, and Stems of *Echinacea pallida* and Its Utilization to Improve the Oxidative Stability of Vegetable Oils. Grasas Aceites.

[71] Daley E. (2019). A Phytochemical and Antibacterial Analysis of *Echinacea purpurea* (L.) Moench Throughout Seasonal Development. Master’s Thesis.

[72] Urbatsch L.E., Neubig K.M., Cox P.B., Flora of North America Editorial Committee (2006). Echinacea. Flora of North America North of Mexico.

[73] De Vos R.C., Moco S., Lommen A., Keurentjes J.J., Bino R.J., Hall R.D. (2007). Untargeted Large-Scale Plant Metabolomics Using Liquid Chromatography Coupled to Mass Spectrometry. Nat. Protoc..

[74] Šimura J., Antoniadi I., Široká J., Tarkowská D., Strnad M., Ljung K., Novák O. (2018). Plant Hormonomics: Multiple Phytohormone Profiling by Targeted Metabolomics. Plant Physiol..

[75] Vinayagam V., Thirugnanasambandam A., Ragupathy S., Sneha R., Newmaster S.G. (2025). Optimization of Extraction Methods for NMR and LC-MS Metabolite Fingerprint Profiling of Botanical Ingredients in Food and Natural Health Products (NHPs). Molecules.

[76] Taiwan Food and Drugs Administration (2019). Method of Test for Preservatives in Foods MOHWA0020.03.

[77] Kuo C., Fang C., Hsu C., Tsai L., Kao Y., Lin M., Tseng S. (2024). Development of a Multiple Analytical Method for Preservatives in Foods. Ann. Rept. Food Drug Res..

[78] Wang L., Zhang Y., Fu X., Zhang T., Ma J., Zhang L., Qian H., Tang K., Li S., Zhao J. (2017). Molecular Cloning, Characterization, and Promoter Analysis of the Isochorismate Synthase (AaICS1) Gene from *Artemisia annua*. J. Zhejiang Univ. Sci. B.

[79] Alseekh S., Aharoni A., Brotman Y., Contrepois K., D’Auria J., Ewald J., Ewald J.C., Fraser P.D., Giavalisco P., Hall R.D. (2021). Mass Spectrometry-Based Metabolomics: A Guide for Annotation, Quantification and Best Reporting Practices. Nat. Methods.

