# Supplementary material for: Organ-Resolved Salicylate–Phenylpropanoid Metabolite Profiling of Echinacea purpurea, E. pallida, and E. tennesseensis Tissues Extracted with 50% Aqueous Methanol by LC–MS/MS and LC–HRMS
Source: Molecules. 2026 Aug 25;31(17):2963. doi: 10.3390/molecules31172963 (PMC13567413; doi:10.3390/molecules31172963)
Supplement: Supplementary file 1 [file molecules-31-02963-s001.zip › SupplementaryFiles/Supplementary Fig S1.pdf]

Plant Identification Report
Lot: 119657250903-1 Echinacea purpurea leaves

Date reported: 17-Sep-25

Reported by: mdu

Call: **Best matches *Echinacea purpurea*.**

Comments: This sample from dried plant leaves. *Echinacea angustifolia* was also identified with generic NS primers as the only *Echinacea* species (results not shown); however, GenBank has low amounts of full 18S accessions for other *Echinacea* species and therefore this marker cannot be relied on for ID.

Instrument name: SimpliAmp; SeqStudio

Primer(s) used/Sequenced by: Generic trnH-psbA primers/mdu

Extraction Method/Extracted by: RIDA kit/mdu

DNAseq Run File: Seq25091501

```
BLASTN 2.17.0+
Reference: Zheng Zhang, Scott Schwartz, Lukas Wagner, and
Webb Miller (2000), "A greedy algorithm for aligning DNA
sequences", J Comput Biol 2000; 7(1-2):203-14.


Reference for database indexing: Aleksandr Morgulis, George
Coulouris, Yan Raytselis, Thomas L. Madden, Richa Agarwala,
Alejandro A. Schaffer (2008), "Database Indexing for
Production MegaBLAST Searches", Bioinformatics 24:1757-1764.


RID: CPAATJC4015


Database: Core nucleotide BLAST database
           118,456,123 sequences; 925,171,125,281 total letters
Query=
Length=510
```

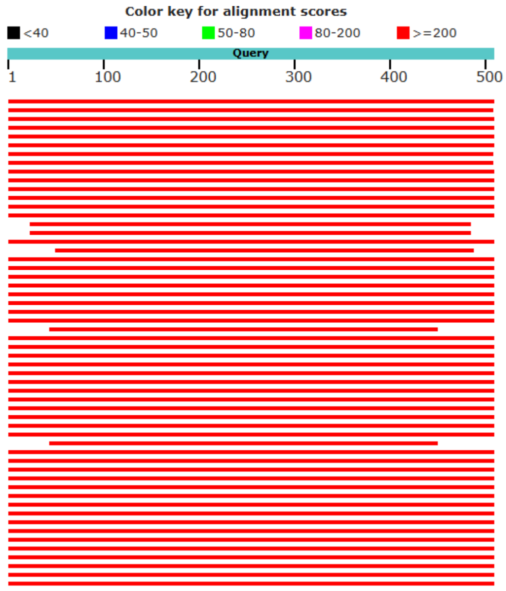

Sequences producing significant alignments:	Score (Bits)	E Value	Max Ident
**ON641367.1 Echinacea purpurea isolate BPTPS168 voucher LISE:96...**	**941**	**0.0**	**100%**
**EU785934.1 Echinacea purpurea PsbA (psbA) gene, partial cds; p...**	**939**	**0.0**	**100%**
NC_034326.1 Echinacea tennesseensis plastid, complete genome	935	0.0	100%
NC_034325.1 Echinacea speciosa plastid, complete genome	935	0.0	100%
MK055334.1 Echinacea purpurea plastid, complete genome	926	0.0	99%
NC_034321.1 Echinacea pallida plastid, complete genome	918	0.0	99%
EU785935.1 Echinacea pallida PsbA (psbA) gene, partial cds; ps...	917	0.0	99%
EU785933.1 Echinacea angustifolia PsbA (psbA) gene, partial cd...	915	0.0	99%
NC_034324.1 Echinacea angustifolia plastid, complete genome	911	0.0	99%
NC_034320.1 Echinacea paradoxa plastid, complete genome	905	0.0	99%
NC_034323.1 Echinacea atrorubens plastid, complete genome	905	0.0	99%
NC_034328.1 Echinacea sanguinea plastid, complete genome	902	0.0	99%
NC_034322.1 Echinacea laevigata plastid, complete genome	893	0.0	98%
NC_034327.1 Echinacea purpurea plastid, complete genome	870	0.0	98%
MF348644.1 Echinacea pallida isolate AD3HJ47 photosystem II pr...	813	0.0	98%
MF348389.1 Echinacea pallida isolate AD0EE57 photosystem II pr...	813	0.0	98%
NC_081940.1 Enydra sessilis chloroplast, complete genome	797	0.0	95%
LS999859.1 Echinacea angustifolia DNA containing psbA-trnH IGS...	787	0.0	99%
MZ292978.1 Acmella paniculata chloroplast, complete genome	754	0.0	93%
NC_035875.1 Ambrosia artemisiifolia chloroplast, complete genome	749	0.0	93%
NC_053299.1 Iostephane heterophylla voucher Schilling 94 chlor...	749	0.0	93%
MG019037.1 Ambrosia artemisiifolia chloroplast, complete genome	749	0.0	93%
NC_036810.2 Ambrosia trifida chloroplast, complete genome	749	0.0	93%
MT793855.1 Mikania sylvatica chloroplast, complete genome	747	0.0	93%
NC_053285.1 Aldama linearis voucher Schilling 70 chloroplast, ...	737	0.0	93%
MT793838.1 Mikania lehmannii chloroplast, complete genome	736	0.0	93%
MF037494.1 Echinacea sanguinea voucher Bengtson9 trnH-psbA int...	730	0.0	99%
NC_053296.1 Aldama canescens voucher Schilling 17 chloroplast,...	728	0.0	92%
NC_057174.1 Aldama robusta voucher Bringel 985 chloroplast, co...	723	0.0	92%
NC_053279.1 Aldama filifolia voucher Loeuille 849 chloroplast,...	723	0.0	92%
NC_053297.1 Aldama corumbensis voucher Loeuille 909 chloroplas...	723	0.0	92%
NC_057172.1 Aldama excelsa voucher Schilling H2481 chloroplast...	721	0.0	92%
MT576958.1 Tithonia diversifolia chloroplast, complete genome	721	0.0	92%
NC_053300.1 Tithonia diversifolia voucher Loeuille 678 chlorop...	721	0.0	92%
MT700543.1 Pappobolus lanatus var. lanatus chloroplast, comple...	721	0.0	92%
NC_053284.1 Aldama kunthiana voucher voucher Silva s.n. (ESA 1...	717	0.0	92%
NC_057177.1 Aldama squalida voucher Loeuille 790 chloroplast, ...	717	0.0	92%
NC_053295.1 Aldama bracteata voucher Filartiga 15 chloroplast,...	717	0.0	92%
NC_057171.1 Aldama discolor voucher Bombo 72 chloroplast, comp...	717	0.0	92%
MF037491.1 Echinacea atrorubens voucher Barnard1532 trnH-psbA ...	717	0.0	99%
NC_053282.1 Aldama goyazii voucher Magenta 716 chloroplast, co...	717	0.0	92%
NC_053294.1 Aldama bakeriana voucher Loeuille 867 chloroplast,...	717	0.0	92%
NC_053286.1 Aldama macrorhiza voucher Magenta 476 chloroplast,...	717	0.0	92%
NC_057181.1 Aldama veredensis voucher Loeuille 921 chloroplast...	717	0.0	92%
NC_057178.1 Aldama tenuifolia voucher Silva s.n. (ESA 122870) ...	717	0.0	92%
NC_053293.1 Aldama aspilioides voucher Filartiga 18 chloroplas...	717	0.0	92%
NC_053700.1 Sigesbeckia orientalis chloroplast, complete genome	713	0.0	92%
NC_057182.1 Aldama vernonioides voucher Magenta 460 chloroplas...	713	0.0	92%
NC_057180.1 Aldama tukumanensis voucher Heiden 1837 chloroplas...	713	0.0	92%
NC_057175.1 Aldama rubra voucher Magenta 388 chloroplast, comp...	713	0.0	92%
NC_053283.1 Aldama grandiflora voucher Loeuille 750 chloroplas...	713	0.0	92%
NC_053288.1 Aldama nudibasilaris voucher Filartiga 1 chloropla...	713	0.0	92%
NC_057176.1 Aldama santacatarinensis voucher Magenta 706 chlor...	712	0.0	92%

NC_053290.1 Aldama pilosa voucher Filartiga 10 chloroplast, co...	712	0.0	92%
NC_053281.1 Aldama gardneri voucher Filartiga 16 chloroplast, ...	712	0.0	92%
MN337904.1 Aldama arenaria chloroplast, complete genome	712	0.0	92%
AB427162.1 Ambrosia artemisiifolia chloroplast psbA gene for D...	710	0.0	93%
KF746370.1 Helianthus grosseserratus isolate DB25 plastid, com...	710	0.0	92%
NC_023109.1 Helianthus divaricatus isolate DB07 plastid, compl...	710	0.0	92%
NC_057179.1 Aldama tuberosa voucher Loeuille 719 chloroplast, ...	708	0.0	91%
NC_053292.1 Aldama arenaria voucher Magenta 275 chloroplast, c...	708	0.0	91%
KF746353.1 Helianthus divaricatus isolate DB08 plastid, comple...	706	0.0	91%
NC_023107.1 Helianthus giganteus isolate DB01 plastid, complet...	706	0.0	91%
NC_023113.1 Helianthus strumosus isolate DB31 plastid, complet...	706	0.0	91%
PV541157.1 Helianthus ciliaris cultivar PI 653550 chloroplast,...	706	0.0	91%
NC_023114.1 Helianthus maximiliani isolate MAX01 plastid, comp...	706	0.0	91%
NC_086876.1 Helianthus mollis plastid, complete genome	706	0.0	91%
KF241793.1 Ambrosia artemisiifolia isolate NJI-7 PsbA (psbA) g...	706	0.0	93%
NC_066996.1 Helianthus carnosus chloroplast, complete genome	706	0.0	91%
KF746377.1 Helianthus tuberosus isolate DB32 plastid, complete...	706	0.0	91%
KF746349.1 Helianthus giganteus isolate DB04 plastid, complete...	706	0.0	91%
KF746351.1 Helianthus grosseserratus isolate DB06 plastid, com...	706	0.0	91%
MF037492.1 Echinacea pallida voucher Rebman1053 trnH-psbA inte...	706	0.0	98%
KF746383.1 Helianthus maximiliani isolate MAX17 plastid, compl...	706	0.0	91%
MK341450.1 Helianthus annuus cultivar HA89(MAX1) chloroplast, ...	706	0.0	91%
MT302566.1 Helianthus hirsutus chloroplast, complete genome	706	0.0	91%
KF746368.1 Helianthus grosseserratus isolate DB23 plastid, com...	706	0.0	91%
KF746379.1 Helianthus tuberosus isolate DB34 plastid, complete...	706	0.0	91%
MF037490.1 Echinacea angustifolia voucher Rolfsmeier19250 trnH...	704	0.0	98%
NC_053298.1 Aldama trichophylla voucher Magenta 390 chloroplas...	704	0.0	91%
MT793836.1 Mikania glomerata chloroplast, complete genome	702	0.0	92%
MT793840.1 Mikania oblongifolia chloroplast, complete genome	702	0.0	92%
MN311247.1 Aldama trichophylla chloroplast, complete genome	702	0.0	91%
NC_053289.1 Aldama nudicaulis voucher Loeuille 734 chloroplast...	702	0.0	91%
MT793841.1 Mikania obtusata chloroplast, complete genome	702	0.0	92%
MT793839.1 Mikania neurocaula chloroplast, complete genome	702	0.0	92%
MT793834.1 Mikania decora chloroplast	702	0.0	92%
NC_053280.1 Aldama fusiformis voucher Siniscalchi 398 chloropl...	702	0.0	91%
MT793844.1 Mikania ternata chloroplast	702	0.0	92%
KF241798.1 Ambrosia artemisiifolia isolate AZ-1 PsbA (psbA) ge...	702	0.0	94%
MW353165.1 Helianthus laciniatus chloroplast, complete genome	701	0.0	91%
NC_058797.1 Helianthus verticillatus chloroplast, complete genome	701	0.0	91%
KF746360.1 Helianthus hirsutus isolate DB15 plastid, complete ...	701	0.0	91%
KF746363.1 Helianthus tuberosus isolate DB18 plastid, complete...	701	0.0	91%
MT302564.1 Helianthus pauciflorus subsp. pauciflorus chloropla...	701	0.0	91%
KF746378.1 Helianthus tuberosus isolate DB33 plastid, complete...	701	0.0	91%
KF746364.1 Helianthus divaricatus isolate DB19 plastid, comple...	701	0.0	91%
MG696658.1 Helianthus tuberosus chloroplast, complete genome	701	0.0	91%
MT793848.1 Litothamnus nitidus chloroplast, complete genome	701	0.0	92%
KF746373.1 Helianthus decapetalus isolate DB28 plastid, comple...	701	0.0	91%

ALIGNMENTS

>ON641367.1 Echinacea purpurea isolate BPTPS168 voucher LISE:96479 chloroplast, complete genome
Length=151924

Score = 941 bits (509), Expect = 0.0
Identities = 509/509 (100%), Gaps = 0/509 (0%)
Strand=Plus/Plus

```
Query  2      GTTATGCATGAACGTAATGCTCATAATTTCCCTCTAGACTTAGCTGCTATTGAAGCTCCA  61
              ||||||||||||||||||||||||||||||||||||||||||||||||||||||||||||
Sbjct  83080  GTTATGCATGAACGTAATGCTCATAATTTCCCTCTAGACTTAGCTGCTATTGAAGCTCCA  83139

Query  62     TATACAAATGGATAAGCCTTTGGTCTGATTGTATAGGAGTTTTTGAACTAAAAAAGGAGC  121
              ||||||||||||||||||||||||||||||||||||||||||||||||||||||||||||
Sbjct  83140  TATACAAATGGATAAGCCTTTGGTCTGATTGTATAGGAGTTTTTGAACTAAAAAAGGAGC  83199

Query  122    AATAACGCCCTCTTGATAAAACAAGAGGGAAGCTATTGCTCCttttttttATTTAGTACT  181
              ||||||||||||||||||||||||||||||||||||||||||||||||||||||||||||
Sbjct  83200  AATAACGCCCTCTTGATAAAACAAGAGGGAAGCTATTGCTCCTTTTTTTTATTTAGTACT  83259

Query  182    ATTTGCCTTACATAGTTTCTTTAAAAATAACAAGTACTTTTTATAGTTTGGTTCGATTTG  241
              ||||||||||||||||||||||||||||||||||||||||||||||||||||||||||||
Sbjct  83260  ATTTGCCTTACATAGTTTCTTTAAAAATAACAAGTACTTTTTATAGTTTGGTTCGATTTG  83319

Query  242    CGTGtttttctttgtattcatttatattataggtttctatatcatatatttttcccaat  301
              ||||||||||||||||||||||||||||||||||||||||||||||||||||||||||||
Sbjct  83320  CGTGTTTTTCTTTGTATTCATTTATATTATAGGTTTCTATATCATATATTTTTCCCAAT  83379

Query  302    cttttatgaagttttatttACAATTTAATTTCAATCGAAAATAGAGAAAAATGACAATTT  361
              ||||||||||||||||||||||||||||||||||||||||||||||||||||||||||||
Sbjct  83380  CTTTTATGAAGTTTTATTTACAATTTAATTTCAATCGAAAATAGAGAAAAATGACAATTT  83439

Query  362    TGCTTTTTGATTTATATTGATTAAATCAAAATAAGAAAGAATATGCtttttttATGTTAA  421
              ||||||||||||||||||||||||||||||||||||||||||||||||||||||||||||
Sbjct  83440  TGCTTTTTGATTTATATTGATTAAATCAAAATAAGAAAGAATATGCTTTTTTTATGTTAA  83499

Query  422    TGGAAAAATATAGTAATACTAGATAATCGTAGAGGGGCGGATGTAGCCAAGTGGATCAAG  481
              ||||||||||||||||||||||||||||||||||||||||||||||||||||||||||||
Sbjct  83500  TGGAAAAATATAGTAATACTAGATAATCGTAGAGGGGCGGATGTAGCCAAGTGGATCAAG  83559

Query  482    GCAGTGGATTGTGAATCCACCATGCGCGG  510
              |||||||||||||||||||||||||||||
Sbjct  83560  GCAGTGGATTGTGAATCCACCATGCGCGG  83588
```

>EU785934.1 Echinacea purpurea PsbA (psbA) gene, partial cds; psbA-trnH intergenic spacer, complete sequence; and tRNA-His (trnH) gene,
partial sequence; chloroplast
Length=508

Score = 939 bits (508), Expect = 0.0
Identities = 508/508 (100%), Gaps = 0/508 (0%)
Strand=Plus/Plus

```
Query  2    GTTATGCATGAACGTAATGCTCATAATTTCCCTCTAGACTTAGCTGCTATTGAAGCTCCA  61
            ||||||||||||||||||||||||||||||||||||||||||||||||||||||||||||
Sbjct  1    GTTATGCATGAACGTAATGCTCATAATTTCCCTCTAGACTTAGCTGCTATTGAAGCTCCA  60

Query  62   TATACAAATGGATAAGCCTTTGGTCTGATTGTATAGGAGTTTTTGAACTAAAAAAGGAGC  121
            ||||||||||||||||||||||||||||||||||||||||||||||||||||||||||||
Sbjct  61   TATACAAATGGATAAGCCTTTGGTCTGATTGTATAGGAGTTTTTGAACTAAAAAAGGAGC  120

Query  122  AATAACGCCCTCTTGATAAAACAAGAGGGAAGCTATTGCTCCttttttttATTTAGTACT  181
            ||||||||||||||||||||||||||||||||||||||||||||||||||||||||||||
Sbjct  121  AATAACGCCCTCTTGATAAAACAAGAGGGAAGCTATTGCTCCTTTTTTTTATTTAGTACT  180
```

```
Query  182   ATTTGCCTTACATAGTTTCTTTAAAAATAACAAGTACTTTTTATAGTTTGGTTCGATTTG  241
             ||||||||||||||||||||||||||||||||||||||||||||||||||||||||||||
Sbjct  181   ATTTGCCTTACATAGTTTCTTTAAAAATAACAAGTACTTTTTATAGTTTGGTTCGATTTG  240

Query  242   CGTGtttttctttgtattcatttatattataggtttctatatcatatatttttcccaat  301
             ||||||||||||||||||||||||||||||||||||||||||||||||||||||||||||
Sbjct  241   CGTGTTTTTTCTTTGTATTCATTTATATTATAGGTTTCTATATCATATATTTTTCCCAAT  300

Query  302   cttttatgaagttttatttACAATTTAATTTCAATCGAAAATAGAGAAAAATGACAATTT  361
             ||||||||||||||||||||||||||||||||||||||||||||||||||||||||||||
Sbjct  301   CTTTTATGAAGTTTTATTTACAATTTAATTTCAATCGAAAATAGAGAAAAATGACAATTT  360

Query  362   TGCTTTTTGATTTATATTGATTAAATCAAAATAAGAAAGAATATGCtttttttATGTTAA  421
             ||||||||||||||||||||||||||||||||||||||||||||||||||||||||||||
Sbjct  361   TGCTTTTTGATTTATATTGATTAAATCAAAATAAGAAAGAATATGCTTTTTTTATGTTAA  420

Query  422   TGGAAAAATATAGTAATACTAGATAATCGTAGAGGGGCGGATGTAGCCAAGTGGATCAAG  481
             ||||||||||||||||||||||||||||||||||||||||||||||||||||||||||||
Sbjct  421   TGGAAAAATATAGTAATACTAGATAATCGTAGAGGGGCGGATGTAGCCAAGTGGATCAAG  480

Query  482   GCAGTGGATTGTGAATCCACCATGCGCG  509
             ||||||||||||||||||||||||||||
Sbjct  481   GCAGTGGATTGTGAATCCACCATGCGCG  508


>NC_034326.1 Echinacea tennesseensis plastid, complete genome
 KX548223.1 Echinacea tennesseensis plastid, complete genome
Length=151877

 Score = 935 bits (506),  Expect = 0.0
 Identities = 508/509 (99%), Gaps = 0/509 (0%)
 Strand=Plus/Minus

Query  2     GTTATGCATGAACGTAATGCTCATAATTTCCCTCTAGACTTAGCTGCTATTGAAGCTCCA  61
             ||||||||||||||||||||||||||||||||||||||||||||||||||||||||||||
Sbjct  534   GTTATGCATGAACGTAATGCTCATAATTTCCCTCTAGACTTAGCTGCTATTGAAGCTCCA  475

Query  62    TATACAAATGGATAAGCCTTTGGTCTGATTGTATAGGAGTTTTTGAACTAAAAAAGGAGC  121
             | ||||||||||||||||||||||||||||||||||||||||||||||||||||||||||
Sbjct  474   TCTACAAATGGATAAGCCTTTGGTCTGATTGTATAGGAGTTTTTGAACTAAAAAAGGAGC  415

Query  122   AATAACGCCCTCTTGATAAAACAAGAGGGAAGCTATTGCTCCttttttttATTTAGTACT  181
             ||||||||||||||||||||||||||||||||||||||||||||||||||||||||||||
Sbjct  414   AATAACGCCCTCTTGATAAAACAAGAGGGAAGCTATTGCTCCTTTTTTTTATTTAGTACT  355

Query  182   ATTTGCCTTACATAGTTTCTTTAAAAATAACAAGTACTTTTTATAGTTTGGTTCGATTTG  241
             ||||||||||||||||||||||||||||||||||||||||||||||||||||||||||||
Sbjct  354   ATTTGCCTTACATAGTTTCTTTAAAAATAACAAGTACTTTTTATAGTTTGGTTCGATTTG  295

Query  242   CGTGtttttctttgtattcatttatattataggtttctatatcatatatttttcccaat  301
             ||||||||||||||||||||||||||||||||||||||||||||||||||||||||||||
Sbjct  294   CGTGTTTTTTCTTTGTATTCATTTATATTATAGGTTTCTATATCATATATTTTTCCCAAT  235

Query  302   cttttatgaagttttatttACAATTTAATTTCAATCGAAAATAGAGAAAAATGACAATTT  361
             ||||||||||||||||||||||||||||||||||||||||||||||||||||||||||||
Sbjct  234   CTTTTATGAAGTTTTATTTACAATTTAATTTCAATCGAAAATAGAGAAAAATGACAATTT  175
```

```
Query  362   TGCTTTTTGATTTATATTGATTAAATCAAAATAAGAAAGAATATGCtttttttATGTTAA  421
             ||||||||||||||||||||||||||||||||||||||||||||||||||||||||||||
Sbjct  174   TGCTTTTTGATTTATATTGATTAAATCAAAATAAGAAAGAATATGCTTTTTTTATGTTAA  115

Query  422   TGGAAAAATATAGTAATACTAGATAATCGTAGAGGGGCGGATGTAGCCAAGTGGATCAAG  481
             ||||||||||||||||||||||||||||||||||||||||||||||||||||||||||||
Sbjct  114   TGGAAAAATATAGTAATACTAGATAATCGTAGAGGGGCGGATGTAGCCAAGTGGATCAAG  55

Query  482   GCAGTGGATTGTGAATCCACCATGCGCGG  510
             |||||||||||||||||||||||||||||
Sbjct  54    GCAGTGGATTGTGAATCCACCATGCGCGG  26


>NC_034325.1 Echinacea speciosa plastid, complete genome
 KX548222.1 Echinacea speciosa plastid, complete genome
Length=151860

 Score = 935 bits (506),  Expect = 0.0
 Identities = 508/509 (99%), Gaps = 0/509 (0%)
 Strand=Plus/Minus

Query  2     GTTATGCATGAACGTAATGCTCATAATTTCCCTCTAGACTTAGCTGCTATTGAAGCTCCA  61
             ||||||||||||||||||||||||||||||||||||||||||||||||||||||||||||
Sbjct  534   GTTATGCATGAACGTAATGCTCATAATTTCCCTCTAGACTTAGCTGCTATTGAAGCTCCA  475

Query  62    TATACAAATGGATAAGCCTTTGGTCTGATTGTATAGGAGTTTTTGAACTAAAAAAGGAGC  121
             | ||||||||||||||||||||||||||||||||||||||||||||||||||||||||||
Sbjct  474   TCTACAAATGGATAAGCCTTTGGTCTGATTGTATAGGAGTTTTTGAACTAAAAAAGGAGC  415

Query  122   AATAACGCCCTCTTGATAAAACAAGAGGGAAGCTATTGCTCCttttttttATTTAGTACT  181
             ||||||||||||||||||||||||||||||||||||||||||||||||||||||||||||
Sbjct  414   AATAACGCCCTCTTGATAAAACAAGAGGGAAGCTATTGCTCCTTTTTTTTATTTAGTACT  355

Query  182   ATTTGCCTTACATAGTTTCTTTAAAAATAACAAGTACTTTTTATAGTTTGGTTCGATTTG  241
             ||||||||||||||||||||||||||||||||||||||||||||||||||||||||||||
Sbjct  354   ATTTGCCTTACATAGTTTCTTTAAAAATAACAAGTACTTTTTATAGTTTGGTTCGATTTG  295

Query  242   CGTGtttttctttgtattcatttatattataggtttctatatcatatatttttcccaat  301
             ||||||||||||||||||||||||||||||||||||||||||||||||||||||||||||
Sbjct  294   CGTGTTTTTCTTTGTATTCATTTATATTATAGGTTTCTATATCATATATTTTTCCCAAT  235

Query  302   cttttatgaagttttatttACAATTTAATTTCAATCGAAAATAGAGAAAAATGACAATTT  361
             ||||||||||||||||||||||||||||||||||||||||||||||||||||||||||||
Sbjct  234   CTTTTATGAAGTTTTATTTACAATTTAATTTCAATCGAAAATAGAGAAAAATGACAATTT  175

Query  362   TGCTTTTTGATTTATATTGATTAAATCAAAATAAGAAAGAATATGCtttttttATGTTAA  421
             ||||||||||||||||||||||||||||||||||||||||||||||||||||||||||||
Sbjct  174   TGCTTTTTGATTTATATTGATTAAATCAAAATAAGAAAGAATATGCTTTTTTTATGTTAA  115

Query  422   TGGAAAAATATAGTAATACTAGATAATCGTAGAGGGGCGGATGTAGCCAAGTGGATCAAG  481
             ||||||||||||||||||||||||||||||||||||||||||||||||||||||||||||
Sbjct  114   TGGAAAAATATAGTAATACTAGATAATCGTAGAGGGGCGGATGTAGCCAAGTGGATCAAG  55

Query  482   GCAGTGGATTGTGAATCCACCATGCGCGG  510
             |||||||||||||||||||||||||||||
Sbjct  54    GCAGTGGATTGTGAATCCACCATGCGCGG  26
```

```
>MK055334.1 Echinacea purpurea plastid, complete genome
Length=151948

 Score = 926 bits (501),  Expect = 0.0
 Identities = 508/511 (99%), Gaps = 2/511 (0%)
 Strand=Plus/Minus

Query  2    GTTATGCATGAACGTAATGCTCATAATTTCCCTCTAGACTTAGCTGCTATTGAAGCTCCA  61
            ||||||||||||||||||||||||||||||||||||||||||||||||||||||||||||
Sbjct  536  GTTATGCATGAACGTAATGCTCATAATTTCCCTCTAGACTTAGCTGCTATTGAAGCTCCA  477

Query  62   TATACAAATGGATAAGCCTTTGGTCTGATTGTATAGGAGTTTTTGAACTAAAAAAGGAGC  121
            | ||||||||||||||||||||||||||||||||||||||||||||||||||||||||||
Sbjct  476  TCTACAAATGGATAAGCCTTTGGTCTGATTGTATAGGAGTTTTTGAACTAAAAAAGGAGC  417

Query  122  AATAACGCCCTCTTGATAAAACAAGAGGGAAGCTATTGCTCCtttttttt-ATTTAGTAC  180
            |||||||||||||||||||||||||||||||||||||||||||||||||| |||||||||
Sbjct  416  AATAACGCCCTCTTGATAAAACAAGAGGGAAGCTATTGCTCCTTTTTTTTTATTTAGTAC  357

Query  181  TATTTGCCTTACATAGTTTCTTTAAAAATAACAAGTACTTTTTATAGTTTGGTTCGATTT  240
            ||||||||||||||||||||||||||||||||||||||||||||||||||||||||||||
Sbjct  356  TATTTGCCTTACATAGTTTCTTTAAAAATAACAAGTACTTTTTATAGTTTGGTTCGATTT  297

Query  241  GCGTGtttttt-ctttgtattcatttatattataggtttctatatcatatattttccca  299
            ||||||||||| ||||||||||||||||||||||||||||||||||||||||||||||||
Sbjct  296  GCGTGTTTTTTTCTTTGTATTCATTTATATTATAGGTTTCTATATCATATATTTTCCCA  237

Query  300  atcttttatgaagttttatttACAATTTAATTTCAATCGAAAATAGAGAAAAATGACAAT  359
            ||||||||||||||||||||||||||||||||||||||||||||||||||||||||||||
Sbjct  236  ATCTTTTATGAAGTTTTATTTACAATTTAATTTCAATCGAAAATAGAGAAAAATGACAAT  177

Query  360  TTTGCTTTTTGATTTATATTGATTAAATCAAAATAAGAAAGAATATGCtttttttATGTT  419
            ||||||||||||||||||||||||||||||||||||||||||||||||||||||||||||
Sbjct  176  TTTGCTTTTTGATTTATATTGATTAAATCAAAATAAGAAAGAATATGCTTTTTTTATGTT  117

Query  420  AATGGAAAAATATAGTAATACTAGATAATCGTAGAGGGGCGGATGTAGCCAAGTGGATCA  479
            ||||||||||||||||||||||||||||||||||||||||||||||||||||||||||||
Sbjct  116  AATGGAAAAATATAGTAATACTAGATAATCGTAGAGGGGCGGATGTAGCCAAGTGGATCA  57

Query  480  AGGCAGTGGATTGTGAATCCACCATGCGCGG  510
            |||||||||||||||||||||||||||||||
Sbjct  56   AGGCAGTGGATTGTGAATCCACCATGCGCGG  26


>NC_034321.1 Echinacea pallida plastid, complete genome
 KX548218.1 Echinacea pallida plastid, complete genome
Length=151883

 Score = 918 bits (497),  Expect = 0.0
 Identities = 506/510 (99%), Gaps = 2/510 (0%)
 Strand=Plus/Minus

Query  2    GTTATGCATGAACGTAATGCTCATAATTTCCCTCTAGACTTAGCTGCTATTGAAGCTCCA  61
            ||||||||||||||||||||||||||||||||||||||||||||||||||||||||||||
Sbjct  534  GTTATGCATGAACGTAATGCTCATAATTTCCCTCTAGACTTAGCTGCTATTGAAGCTCCA  475
```

```
Query  62   TATACAAATGGATAAGCCTTTGGTCTGATTGTATAGGAGTTTTTGAACTAAAAAAGGAGC  121
            | ||||||||||||||||||||||||||||||||||||||||||||||||||||||||||
Sbjct  474  TCTACAAATGGATAAGCCTTTGGTCTGATTGTATAGGAGTTTTTGAACTAAAAAAGGAGC  415

Query  122  AATAACGCCCTCTTGATAAAACAAGAGGGAAGCTATTGCTCCttttttttATTTAGTACT  181
            ||||||||||||||||||||||||||||||||||||||||||||||||| ||||||||||
Sbjct  414  AATAACGCCCTCTTGATAAAACAAGAGGGAAGCTATTGCTCCTTTTTTT-ATTTAGTACT  356

Query  182  ATTTGCCTTACATAGTTTCTTTAAAAATAACAAGTACTTTTTATAGTTTGGTTCGATTTG  241
            ||||||||||||||||||||||||||||||||||||||||||||||||||||||||||||
Sbjct  355  ATTTGCCTTACATAGTTTCTTTAAAAATAACAAGTACTTTTTATAGTTTGGTTCGATTTG  296

Query  242  CGTGttttttctttgtattcatttatattataggtttctatatcatatatttttcccaat  301
            |||||||||||||||||||||||||||||||||||||||||||||| |||||||||||||
Sbjct  295  CGTGTTTTTTCTTTGTATTCATTTATATTATAGGTTTCTATATCATTTATTTTTCCCAAT  236

Query  302  cttttatgaagttttatttACAATTTAATTTCAATCGAAAATAGAGAAAAATGACAATTT  361
            ||||||||||||||||||||||||||||||||||||||||||||||||||||||||||||
Sbjct  235  CTTTTATGAAGTTTTATTTACAATTTAATTTCAATCGAAAATAGAGAAAAATGACAATTT  176

Query  362  TGCTTTTTGATTTATATTGATTAAATCAAAATAAGAAAGAATATGCtttttttATGTTAA  421
            ||||||||||||||||||||||||||||||||||||||||||||||||||||||||||||
Sbjct  175  TGCTTTTTGATTTATATTGATTAAATCAAAATAAGAAAGAATATGCTTTTTTTATGTTAA  116

Query  422  TGGAAAAA-TATAGTAATACTAGATAATCGTAGAGGGGCGGATGTAGCCAAGTGGATCAA  480
            |||||||| |||||||||||||||||||||||||||||||||||||||||||||||||||
Sbjct  115  TGGAAAAAATATAGTAATACTAGATAATCGTAGAGGGGCGGATGTAGCCAAGTGGATCAA  56

Query  481  GGCAGTGGATTGTGAATCCACCATGCGCGG  510
            ||||||||||||||||||||||||||||||
Sbjct  55   GGCAGTGGATTGTGAATCCACCATGCGCGG  26


>EU785935.1 Echinacea pallida PsbA (psbA) gene, partial cds; psbA-trnH intergenic
spacer, complete sequence; and tRNA-His (trnH) gene,
partial sequence; chloroplast
Length=508

 Score = 917 bits (496),  Expect = 0.0
 Identities = 505/509 (99%), Gaps = 2/509 (0%)
 Strand=Plus/Plus

Query  2    GTTATGCATGAACGTAATGCTCATAATTTCCCTCTAGACTTAGCTGCTATTGAAGCTCCA  61
            ||||||||||||||||||||||||||||||||||||||||||||||||||||||||||||
Sbjct  1    GTTATGCATGAACGTAATGCTCATAATTTCCCTCTAGACTTAGCTGCTATTGAAGCTCCA  60

Query  62   TATACAAATGGATAAGCCTTTGGTCTGATTGTATAGGAGTTTTTGAACTAAAAAAGGAGC  121
            | ||||||||||||||||||||||||||||||||||||||||||||||||||||||||||
Sbjct  61   TCTACAAATGGATAAGCCTTTGGTCTGATTGTATAGGAGTTTTTGAACTAAAAAAGGAGC  120

Query  122  AATAACGCCCTCTTGATAAAACAAGAGGGAAGCTATTGCTCCttttttttATTTAGTACT  181
            |||||||||||||||||||||||||||||||||||||||||| |||||||||||||||||
Sbjct  121  AATAACGCCCTCTTGATAAAACAAGAGGGAAGCTATTGCTCC-TTTTTTTATTTAGTACT  179

Query  182  ATTTGCCTTACATAGTTTCTTTAAAAATAACAAGTACTTTTTATAGTTTGGTTCGATTTG  241
            ||||||||||||||||||||||||||||||||||||||||||||||||||||||||||||
```

```
Sbjct  180  ATTTGCCTTACATAGTTTCTTTAAAAATAACAAGTACTTTTTATAGTTTGGTTCGATTTG  239

Query  242  CGTGttttttctttgtattcatttatattataggtttctatatcatatatttttcccaat  301
            |||||||||||||||||||||||||||||||||||||||||||||| |||||||||||||
Sbjct  240  CGTGTTTTTTCTTTGTATTCATTTATATTATAGGTTTCTATATCATTTATTTTTCCCAAT  299

Query  302  cttttatgaagttttatttACAATTTAATTTCAATCGAAAATAGAGAAAAATGACAATTT  361
            ||||||||||||||||||||||||||||||||||||||||||||||||||||||||||||
Sbjct  300  CTTTTATGAAGTTTTATTTACAATTTAATTTCAATCGAAAATAGAGAAAAATGACAATTT  359

Query  362  TGCTTTTTGATTTATATTGATTAAATCAAAATAAGAAAGAATATGCtttttttATGTTAA  421
            ||||||||||||||||||||||||||||||||||||||||||||||||||||||||||||
Sbjct  360  TGCTTTTTGATTTATATTGATTAAATCAAAATAAGAAAGAATATGCTTTTTTTATGTTAA  419

Query  422  TGG-AAAAATATAGTAATACTAGATAATCGTAGAGGGGCGGATGTAGCCAAGTGGATCAA  480
            ||| ||||||||||||||||||||||||||||||||||||||||||||||||||||||||
Sbjct  420  TGGAAAAAATATAGTAATACTAGATAATCGTAGAGGGGCGGATGTAGCCAAGTGGATCAA  479

Query  481  GGCAGTGGATTGTGAATCCACCATGCGCG  509
            |||||||||||||||||||||||||||||
Sbjct  480  GGCAGTGGATTGTGAATCCACCATGCGCG  508


>EU785933.1 Echinacea angustifolia PsbA (psbA) gene, partial cds; psbA-trnH
intergenic spacer, complete sequence; and tRNA-His (trnH)
gene, partial sequence; chloroplast
Length=506

 Score = 915 bits (495),  Expect = 0.0
 Identities = 504/508 (99%), Gaps = 2/508 (0%)
 Strand=Plus/Plus

Query  2    GTTATGCATGAACGTAATGCTCATAATTTCCCTCTAGACTTAGCTGCTATTGAAGCTCCA  61
            ||||||||||||||||||||||||||||||||||||||||||||||||||||||||||||
Sbjct  1    GTTATGCATGAACGTAATGCTCATAATTTCCCTCTAGACTTAGCTGCTATTGAAGCTCCA  60

Query  62   TATACAAATGGATAAGCCTTTGGTCTGATTGTATAGGAGTTTTTGAACTAAAAAAGGAGC  121
            | ||||||||||||||||||||||||||||||||||||||||||||||||||||||||||
Sbjct  61   TCTACAAATGGATAAGCCTTTGGTCTGATTGTATAGGAGTTTTTGAACTAAAAAAGGAGC  120

Query  122  AATAACGCCCTCTTGATAAAACAAGAGGGAAGCTATTGCTCCttttttttATTTAGTACT  181
            |||||||||||||||||||||||||||||||||||||||||| |||||||||||||||||
Sbjct  121  AATAACGCCCTCTTGATAAAACAAGAGGGAAGCTATTGCTCC-TTTTTTTATTTAGTACT  179

Query  182  ATTTGCCTTACATAGTTTCTTTAAAAATAACAAGTACTTTTTATAGTTTGGTTCGATTTG  241
            ||||||||||||||||||||||||||||||||||||||||||||||||||||||||||||
Sbjct  180  ATTTGCCTTACATAGTTTCTTTAAAAATAACAAGTACTTTTTATAGTTTGGTTCGATTTG  239

Query  242  CGTGttttttctttgtattcatttatattataggtttctatatcatatatttttcccaat  301
            ||||||||||||||||||||||||||||||||||||||||||||||||||||||||||||
Sbjct  240  CGTGTTTTTTCTTTGTATTCATTTATATTATAGGTTTCTATATCATATATTTTTCCCAAT  299

Query  302  cttttatgaagttttatttACAATTTAATTTCAATCGAAAATAGAGAAAAATGACAATTT  361
            ||||||||||||||||||||||||||||||||||||||||||||||||||||||||||||
Sbjct  300  CTTTTATGAAGTTTTATTTACAATTTAATTTCAATCGAAAATAGAGAAAAATGACAATTT  359

Query  362  TGCTTTTTGATTTATATTGATTAAATCAAAATAAGAAAGAATATGCtttttttATGTTAA  421
```

```
              |||||||| ||||||||||||||||||||||||||||||||||||| |||||||||||||
Sbjct  360   TGCTTTTTTATTTATATTGATTAAATCAAAATAAGAAAGAATATGC-TTTTTTATGTTAA  418

Query  422   TGGAAAAATATAGTAATACTAGATAATCGTAGAGGGGCGGATGTAGCCAAGTGGATCAAG  481
             ||||||||||||||||||||||||||||||||||||||||||||||||||||||||||||
Sbjct  419   TGGAAAAATATAGTAATACTAGATAATCGTAGAGGGGCGGATGTAGCCAAGTGGATCAAG  478

Query  482   GCAGTGGATTGTGAATCCACCATGCGCG  509
             ||||||||||||||||||||||||||||
Sbjct  479   GCAGTGGATTGTGAATCCACCATGCGCG  506


>NC_034324.1 Echinacea angustifolia plastid, complete genome
 KX548221.1 Echinacea angustifolia plastid, complete genome
Length=151935

 Score = 911 bits (493),  Expect = 0.0
 Identities = 504/509 (99%), Gaps = 2/509 (0%)
 Strand=Plus/Minus

Query  2     GTTATGCATGAACGTAATGCTCATAATTTCCCTCTAGACTTAGCTGCTATTGAAGCTCCA  61
             ||||||||||||||||||||||||||||||||||||||||||||||||||||||||||||
Sbjct  532   GTTATGCATGAACGTAATGCTCATAATTTCCCTCTAGACTTAGCTGCTATTGAAGCTCCA  473

Query  62    TATACAAATGGATAAGCCTTTGGTCTGATTGTATAGGAGTTTTTGAACTAAAAAAGGAGC  121
             | ||||||||||||||||||||||||||||||||||||||||||||||||||||||||||
Sbjct  472   TCTACAAATGGATAAGCCTTTGGTCTGATTGTATAGGAGTTTTTGAACTAAAAAAGGAGC  413

Query  122   AATAACGCCCTCTTGATAAAACAAGAGGGAAGCTATTGCTCCttttttttATTTAGTACT  181
             ||||| |||||||||||||||||||||| ||||||||||||||||||| ||||||||||
Sbjct  412   AATAATGCCCTCTTGATAAAACAAGAGGAAAGCTATTGCTCCTTTTTTT-ATTTAGTACT  354

Query  182   ATTTGCCTTACATAGTTTCTTTAAAAATAACAAGTACTTTTTATAGTTTGGTTCGATTTG  241
             ||||||||||||||||||||||||||||||||||||||||||||||||||||||||||||
Sbjct  353   ATTTGCCTTACATAGTTTCTTTAAAAATAACAAGTACTTTTTATAGTTTGGTTCGATTTG  294

Query  242   CGTGtttttctttgtattcatttatattataggtttctatatcatatatttttcccaat  301
             ||||||||| |||||||||||||||||||||||||||||||||||||||||||||||||
Sbjct  293   CGTGTTTTT-CTTTGTATTCATTTATATTATAGGTTTCTATATCATATATTTTTCCCAAT  235

Query  302   cttttatgaagttttatttACAATTTAATTTCAATCGAAAATAGAGAAAAATGACAATTT  361
             ||||||||||||||||||||||||||||||||||||||||||||||||||||||||||||
Sbjct  234   CTTTTATGAAGTTTTATTTACAATTTAATTTCAATCGAAAATAGAGAAAAATGACAATTT  175

Query  362   TGCTTTTTGATTTATATTGATTAAATCAAAATAAGAAAGAATATGCtttttttATGTTAA  421
             ||||||||||||||||||||||||||||||||||||||||||||||||||||||||||||
Sbjct  174   TGCTTTTTGATTTATATTGATTAAATCAAAATAAGAAAGAATATGCTTTTTTTATGTTAA  115

Query  422   TGGAAAAATATAGTAATACTAGATAATCGTAGAGGGGCGGATGTAGCCAAGTGGATCAAG  481
             ||||||||||||||||||||||||||||||||||||||||||||||||||||||||||||
Sbjct  114   TGGAAAAATATAGTAATACTAGATAATCGTAGAGGGGCGGATGTAGCCAAGTGGATCAAG  55

Query  482   GCAGTGGATTGTGAATCCACCATGCGCGG  510
             |||||||||||||||||||||||||||||
Sbjct  54    GCAGTGGATTGTGAATCCACCATGCGCGG  26
```

```
>NC_034320.1 Echinacea paradoxa plastid, complete genome
 KX548217.1 Echinacea paradoxa plastid, complete genome
Length=151837

 Score = 905 bits (490),  Expect = 0.0
 Identities = 504/510 (99%), Gaps = 3/510 (1%)
 Strand=Plus/Minus

Query  2    GTTATGCATGAACGTAATGCTCATAATTTCCCTCTAGACTTAGCTGCTATTGAAGCTCCA  61
            ||||||||||||||||||||||||||||||||||||||||||||||||||||||||||||
Sbjct  533  GTTATGCATGAACGTAATGCTCATAATTTCCCTCTAGACTTAGCTGCTATTGAAGCTCCA  474

Query  62   TATACAAATGGATAAGCCTTTGGTCTGATTGTATAGGAGTTTTTGAACTAAAAAAGGAGC  121
            | ||||||||||||||||||||||||||||||||||||||||||||||||||||||||||
Sbjct  473  TCTACAAATGGATAAGCCTTTGGTCTGATTGTATAGGAGTTTTTGAACTAAAAAAGGAGC  414

Query  122  AATAACGCCCTCTTGATAAAACAAGAGGGAAGCTATTGCTCCttttttttATTTAGTACT  181
            ||||||| ||||||||||||||||||||||||||||||||||||||||| ||||||||||
Sbjct  413  AATAACGTCCTCTTGATAAAACAAGAGGGAAGCTATTGCTCCTTTTTTT-ATTTAGTACT  355

Query  182  ATTTGCCTTACATAGTTTCTTTAAAAATAACAAGTACTTTTTATAGTTTGGTTCGATTTG  241
            ||||||||||||||||||||||||||||||||||||||||||||||||||||||||||||
Sbjct  354  ATTTGCCTTACATAGTTTCTTTAAAAATAACAAGTACTTTTTATAGTTTGGTTCGATTTG  295

Query  242  CGTGtttttttctttgtattcatttatattataggtttctatatcatatatttttcccaat  301
            |||||||||||||||||||||||||||||||||||||||||||||| |||||||||||||
Sbjct  294  CGTGTTTTTTCTTTGTATTCATTTATATTATAGGTTTCTATATCATTTATTTTTCCCAAT  235

Query  302  cttttatgaagttttatttACAATTTAATTTCAATCGAAAATAGAGAAAAATGACAATTT  361
            ||||||||||||||||||||||||||||||||||||||||||||||||||||||||||||
Sbjct  234  CTTTTATGAAGTTTTATTTACAATTTAATTTCAATCGAAAATAGAGAAAAATGACAATTT  175

Query  362  TGCTTTTTGATTTATATTGATTAAATCAAAATAAGAAAGAATATGCttttttttATGTTAA  421
            ||||||||||||||||||||||||||||||||||||||||||||||||||||| |||||||
Sbjct  174  TGCTTTTTGATTTATATTGATTAAATCAAAATAAGAAAGAATATGCTTTTTTT-ATGTTAA  116

Query  422  TGG-AAAAATATAGTAATACTAGATAATCGTAGAGGGGCGGATGTAGCCAAGTGGATCAA  480
            ||| ||||||||||||||||||||||||||||||||||||||||||||||||||||||||
Sbjct  115  TGGAAAAAATATAGTAATACTAGATAATCGTAGAGGGGCGGATGTAGCCAAGTGGATCAA  56

Query  481  GGCAGTGGATTGTGAATCCACCATGCGCGG  510
            ||||||||||||||||||||||||||||||
Sbjct  55   GGCAGTGGATTGTGAATCCACCATGCGCGG  26


>NC_034323.1 Echinacea atrorubens plastid, complete genome
 KX548220.1 Echinacea atrorubens plastid, complete genome
Length=151912

 Score = 905 bits (490),  Expect = 0.0
 Identities = 504/510 (99%), Gaps = 3/510 (1%)
 Strand=Plus/Minus

Query  2    GTTATGCATGAACGTAATGCTCATAATTTCCCTCTAGACTTAGCTGCTATTGAAGCTCCA  61
            ||||||||||||||||||||||||||||||||||||||||||||||||||||||||||||
Sbjct  533  GTTATGCATGAACGTAATGCTCATAATTTCCCTCTAGACTTAGCTGCTATTGAAGCTCCA  474
```

```
Query  62   TATACAAATGGATAAGCCTTTGGTCTGATTGTATAGGAGTTTTTGAACTAAAAAAGGAGC  121
            | ||||||||||||||||||||||||||||||||||||||||||||||||||||||||||
Sbjct  473  TCTACAAATGGATAAGCCTTTGGTCTGATTGTATAGGAGTTTTTGAACTAAAAAAGGAGC  414

Query  122  AATAACGCCCTCTTGATAAAACAAGAGGGAAGCTATTGCTCCttttttttATTTAGTACT  181
            ||||||| ||||||||||||||||||||||||||||||||||||||||| ||||||||||
Sbjct  413  AATAACGTCCTCTTGATAAAACAAGAGGGAAGCTATTGCTCCTTTTTTT-ATTTAGTACT  355

Query  182  ATTTGCCTTACATAGTTTCTTTAAAAATAACAAGTACTTTTTATAGTTTGGTTCGATTTG  241
            ||||||||||||||||||||||||||||||||||||||||||||||||||||||||||||
Sbjct  354  ATTTGCCTTACATAGTTTCTTTAAAAATAACAAGTACTTTTTATAGTTTGGTTCGATTTG  295

Query  242  CGTGttttttctttgtattcatttatattataggtttctatatcatatatttttcccaat  301
            |||||||||||||||||||||||||||||||||||||||||||||| |||||||||||||
Sbjct  294  CGTGTTTTTTCTTTGTATTCATTTATATTATAGGTTTCTATATCATTTATTTTTCCCAAT  235

Query  302  cttttatgaagttttatttACAATTTAATTTCAATCGAAAATAGAGAAAAATGACAATTT  361
            ||||||||||||||||||||||||||||||||||||||||||||||||||||||||||||
Sbjct  234  CTTTTATGAAGTTTTATTTACAATTTAATTTCAATCGAAAATAGAGAAAAATGACAATTT  175

Query  362  TGCTTTTTGATTTATATTGATTAAATCAAAATAAGAAAGAATATGCtttttttATGTTAA  421
            |||||||||||||||||||||||||||||||||||||||||||||||||||| |||||||
Sbjct  174  TGCTTTTTGATTTATATTGATTAAATCAAAATAAGAAAGAATATGCTTTTTT-ATGTTAA  116

Query  422  TGG-AAAAATATAGTAATACTAGATAATCGTAGAGGGGCGGATGTAGCCAAGTGGATCAA  480
            ||| ||||||||||||||||||||||||||||||||||||||||||||||||||||||||
Sbjct  115  TGGAAAAAATATAGTAATACTAGATAATCGTAGAGGGGCGGATGTAGCCAAGTGGATCAA  56

Query  481  GGCAGTGGATTGTGAATCCACCATGCGCGG  510
            ||||||||||||||||||||||||||||||
Sbjct  55   GGCAGTGGATTGTGAATCCACCATGCGCGG  26


>NC_034328.1 Echinacea sanguinea plastid, complete genome
 KX548225.1 Echinacea sanguinea plastid, complete genome
Length=151926

 Score = 902 bits (488),  Expect = 0.0
 Identities = 503/510 (99%), Gaps = 2/510 (0%)
 Strand=Plus/Minus

Query  2    GTTATGCATGAACGTAATGCTCATAATTTCCCTCTAGACTTAGCTGCTATTGAAGCTCCA  61
            ||||||||||||||||||||||||||||||||||||||||||||||||||||||||||||
Sbjct  534  GTTATGCATGAACGTAATGCTCATAATTTCCCTCTAGACTTAGCTGCTATTGAAGCTCCA  475

Query  62   TATACAAATGGATAAGCCTTTGGTCTGATTGTATAGGAGTTTTTGAACTAAAAAAGGAGC  121
            | ||||||||||||||||||||||||||||||||||||||||||||||||||||||||||
Sbjct  474  TCTACAAATGGATAAGCCTTTGGTCTGATTGTATAGGAGTTTTTGAACTAAAAAAGGAGC  415

Query  122  AATAACGCCCTCTTGATAAAACAAGAGGGAAGCTATTGCTCCttttttttATTTAGTACT  181
            ||||||||||||||||||||||||||||||||||||||||||||||||| ||||||||||
Sbjct  414  AATAACGCCCTCTTGATAAAACAAGAGGGAAGCTATTGCTCCTTTTTTT-ATTTAGTACT  356

Query  182  ATTTGCCTTACATAGTTTCTTTAAAAATAACAAGTACTTTTTATAGTTTGGTTCGATTTG  241
            ||||||||||||||||||||||||||||||||||||||||||||||||||||||||||||
Sbjct  355  ATTTGCCTTACATAGTTTCTTTAAAAATAACAAGTACTTTTTATAGTTTGGTTCGATTTG  296
```

```
Query  242   CGTGttttttctttgtattcatttatattataggtttctatatcatatatttttcccaat  301
             |||||||||||||||||||||||||||||||||||||||||||||| |||||||||||||
Sbjct  295   CGTGTTTTTTCTTTGTATTCATTTATATTATAGGTTTCTATATCATTTATTTTTCCCAAT  236

Query  302   cttttatgaagttttatttACAATTTAATTTCAATCGAAAATAGAGAAAAATGACAATTT  361
              |||||||||||||||||||||||||||||||||||||||||||| ||||||||||||||
Sbjct  235   ATTTTATGAAGTTTTATTTACAATTTAATTTCAATCGAAAATAGATAAAAATGACAATTT  176

Query  362   TGCTTTTTGATTTATATTGATTAAATCAAAATAAGAAAGAATATGCtttttttATGTTAA  421
             |||||||| |||||||||||||||||||||||||||||||||||||||||||||||||||
Sbjct  175   TGCTTTTTTATTTATATTGATTAAATCAAAATAAGAAAGAATATGCTTTTTTTATGTTAA  116

Query  422   TGGAAAAA-TATAGTAATACTAGATAATCGTAGAGGGGCGGATGTAGCCAAGTGGATCAA  480
             |||||||| |||||||||||||||||||||||||||||||||||||||||||||||||||
Sbjct  115   TGGAAAAAATATAGTAATACTAGATAATCGTAGAGGGGCGGATGTAGCCAAGTGGATCAA  56

Query  481   GGCAGTGGATTGTGAATCCACCATGCGCGG  510
             ||||||||||||||||||||||||||||||
Sbjct  55    GGCAGTGGATTGTGAATCCACCATGCGCGG  26


>NC_034322.1 Echinacea laevigata plastid, complete genome
 KX548219.1 Echinacea laevigata plastid, complete genome
Length=151886

 Score = 893 bits (483),  Expect = 0.0
 Identities = 504/513 (98%), Gaps = 5/513 (1%)
 Strand=Plus/Minus

Query  2     GTTATGCATGAACGTAATGCTCATAATTTCCCTCTAGACTTAGCTGCTATTGAAGCTCCA  61
             ||||||||||||||||||||||||||||||||||||||||||||||||||||||||||||
Sbjct  537   GTTATGCATGAACGTAATGCTCATAATTTCCCTCTAGACTTAGCTGCTATTGAAGCTCCA  478

Query  62    TATACAAATGGATAAGCCTTTGGTCTGATTGTATAGGAGTTTTTGAACTAAAAAAGGAGC  121
             | ||||||||||||||||||||||||||||||||||||||||||||||||||||||||||
Sbjct  477   TCTACAAATGGATAAGCCTTTGGTCTGATTGTATAGGAGTTTTTGAACTAAAAAAGGAGC  418

Query  122   AATAACGCCCTCTTGATAAAACAAGAGGGAAGCTATTGCTCCttttttttATTTAGTACT  181
             ||||||||||||||||||||||||||||||||||||||||||||||||| ||||||||||
Sbjct  417   AATAACGCCCTCTTGATAAAACAAGAGGGAAGCTATTGCTCCTTTTTTT-ATTTAGTACT  359

Query  182   ATTTGCCTTACATAGTTTCTTAAAAATAACAAGTACTTTTTATAGTTTGGTTCGATTTG  241
             ||||||||||||||||||||||||||||| ||||||||||||||||||||||||||||||
Sbjct  358   ATTTGCCTTACATAGTTTCTTAAAAATAATAAGTACTTTTTATAGTTTGGTTCGATTTG  299

Query  242   CGTGttttttctttgtattcatttatattataggtttctatatcatatatttttcccaat  301
             ||||||||||||||||||||||||||||||||||||||||||||||||||||||||||||
Sbjct  298   CGTGTTTTTTCTTTGTATTCATTTATATTATAGGTTTCTATATCATATATTTTTCCCAAT  239

Query  302   cttttatgaagttttatttACAATTTAATTTCAATCGAAAATAGAGAAAAATGACAATTT  361
             ||||||||||||||||||||||||||||||||||||||||||||| ||||||||||||||
Sbjct  238   CTTTTATGAAGTTTTATTTACAATTTAATTTCAATCGAAAATAGATAAAAATGACAATTT  179

Query  362   TGCTTTTTGATTTATATTGATTAAATCAAAATAAGAAAGAA----TATGCtttttttATG  417
             |||||||||||||||||||||||||| ||||||||||||||    |||||||||||||||
Sbjct  178   TGCTTTTTGATTTATATTGATTAAATAAAAATAAGAAAGAAAGAATATGCTTTTTTTATG  119
```

```
Query  418  TTAATGGAAAAATATAGTAATACTAGATAATCGTAGAGGGGCGGATGTAGCCAAGTGGAT  477
            ||||||||||||||||||||||||||||||||||||||||||||||||||||||||||||
Sbjct  118  TTAATGGAAAAATATAGTAATACTAGATAATCGTAGAGGGGCGGATGTAGCCAAGTGGAT  59

Query  478  CAAGGCAGTGGATTGTGAATCCACCATGCGCGG  510
            |||||||||||||||||||||||||||||||||
Sbjct  58   CAAGGCAGTGGATTGTGAATCCACCATGCGCGG  26
```

>NC_034327.1 Echinacea purpurea plastid, complete genome
 KX548224.1 Echinacea purpurea plastid, complete genome
Length=151913

 Score = 870 bits (471), Expect = 0.0
 Identities = 498/509 (98%), Gaps = 9/509 (2%)
 Strand=Plus/Minus

```
Query  2    GTTATGCATGAACGTAATGCTCATAATTTCCCTCTAGACTTAGCTGCTATTGAAGCTCCA  61
            ||||||||||||||||||||||||||||||||||||||||||||||||||||||||||||
Sbjct  525  GTTATGCATGAACGTAATGCTCATAATTTCCCTCTAGACTTAGCTGCTATTGAAGCTCCA  466

Query  62   TATACAAATGGATAAGCCTTTGGTCTGATTGTATAGGAGTTTTTGAACTAAAAAAGGAGC  121
            | ||||||||||||||||||||||||||||||||||||||||||||||||||||||||||
Sbjct  465  TCTACAAATGGATAAGCCTTTGGTCTGATTGTATAGGAGTTTTTGAACTAAAAAAGGAGC  406

Query  122  AATAACGCCCTCTTGATAAAACAAGAGGGAAGCTATTGCTCCttttttttATTTAGTACT  181
            ||||||||||||||||||||||||||||||||||||||||||||||||| ||||||||||
Sbjct  405  AATAACGCCCTCTTGATAAAACAAGAGGGAAGCTATTGCTCCTTTTTTT-ATTTAGTACT  347

Query  182  ATTTGCCTTACATAGTTTCTTTAAAAATAACAAGTACTTTTTATAGTTTGGTTCGATTTG  241
            ||||||||||||||||||||||||||||||||||||||||||||||||||||||||||||
Sbjct  346  ATTTGCCTTACATAGTTTCTTTAAAAATAACAAGTACTTTTTATAGTTTGGTTCGATTTG  287

Query  242  CGTGtttttttctttgtattcatttatattataggtttctatatcatatatttttcccaat  301
            ||||||||||||||||||||||||||||||||||||||||||||||||||||||||||||
Sbjct  286  CGTGTTTTTTCTTTGTATTCATTTATATTATAGGTTTCTATATCATATATTTTTCCCAAT  227

Query  302  cttttatgaagttttatttACAATTTAATTTCAATCGAAAATAGAGAAAAATGACAATTT  361
            |||||||||||||||||||||||||||||||||||||||||||||||||||||| |||||
Sbjct  226  CTTTTATGAAGTTTTATTTACAATTTAATTTCAATCGAAAATAGAGAAAAATGAGAATTT  167

Query  362  TGCTTTTTGATTTATATTGATTAAATCAAAATAAGAAAGAATATGCttttttttATGTTAA  421
            ||||||||||||||||||||||||||||||||||||||||||||||||||||||||||||
Sbjct  166  TGCTTTTTGATTTATATTGATTAAATCAAAATAAGAAAGAATATGCTTTTTTTATGTTAA  107

Query  422  TGGAAAAATATAGTAATACTAGATAATCGTAGAGGGGCGGATGTAGCCAAGTGGATCAAG  481
            |||||||||||||||        |||||||||||||||||||||||||||||||||||||
Sbjct  106  TGGAAAAATATAGTA--------TAATCGTAGAGGGGCGGATGTAGCCAAGTGGATCAAG  55

Query  482  GCAGTGGATTGTGAATCCACCATGCGCGG  510
            |||||||||||||||||||||||||||||
Sbjct  54   GCAGTGGATTGTGAATCCACCATGCGCGG  26
```

>MF348644.1 Echinacea pallida isolate AD3HJ47 photosystem II protein D1 (psbA)
gene, partial cds; and psbA-trnH intergenic spacer, partial
sequence; chloroplast

```
Length=466

 Score = 813 bits (440),  Expect = 0.0
 Identities = 459/467 (98%), Gaps = 5/467 (1%)
 Strand=Plus/Plus

Query  24   ATAATTTCCCTCTAGACTTAGCTGCTATTGAAGCTCCATATACAAATGGATAAGCCTTTG  83
            ||||||||||||||||||||||||||||||||||||||| ||||||||||||||||||||
Sbjct  1    ATAATTTCCCTCTAGACTTAGCTGCTATTGAAGCTCCATCTACAAATGGATAAGCCTTTG  60

Query  84   GTCTGATTGTATAGGAGTTTTTGAACTAAAAAAGGAGCAATAACGCCCTCTTGATAAAAC  143
            ||||||||||||||||||||||||||||||||||||||||||||||||||||||||||||
Sbjct  61   GTCTGATTGTATAGGAGTTTTTGAACTAAAAAAGGAGCAATAACGCCCTCTTGATAAAAC  120

Query  144  AAGAGGGAAGCTATTGCTCCttttttttATTTAGTACTATTTGCCTTACATAGTTTCTTT  203
            |||||||||||||||||||| |||||||||||||||||||||||||||||||||||||||
Sbjct  121  AAGAGGGAAGCTATTGCTCC-TTTTTTTATTTAGTACTATTTGCCTTACATAGTTTCTTT  179

Query  204  AAAAATAACAAGTACTTTTTATAGTTTGGTTCGATTTGCGTGtttttttctttgtattcat  263
            |||||||| |||||||||||||||||||||||||||||||||||||||||||||||||||
Sbjct  180  AAAAATAATAAGTACTTTTTATAGTTTGGTTCGATTTGCGTGTTTTTTCTTTGTATTCAT  239

Query  264  ttatattataggtttctatatcatatatttttcccaatcttttatgaagttttatttACA  323
            ||||||||||||||||||||||||||||||||||||||||||||||||||||||||||||
Sbjct  240  TTATATTATAGGTTTCTATATCATATATTTTTCCCAATCTTTTATGAAGTTTTATTTACA  299

Query  324  ATTTAATTTCAATCGAAAATAGAGAAAAATGACAATTTTGCTTTTTGATTTATATTGATT  383
            ||||||||||||||||||||||||||||||||||||||||||||||||||||||||||||
Sbjct  300  ATTTAATTTCAATCGAAAATAGAGAAAAATGACAATTTTGCTTTTTGATTTATATTGATT  359

Query  384  AAATCAAAAT----AAGAAAGAATATGCtttttttATGTTAATGGAAAAATATAGTAATA  439
            |||| |||||    ||||||||||||||||||||||||||||||||||||||||||||||
Sbjct  360  AAATAAAAATAAGAAAGAAAGAATATGCTTTTTTTATGTTAATGGAAAAATATAGTAATA  419

Query  440  CTAGATAATCGTAGAGGGGCGGATGTAGCCAAGTGGATCAAGGCAGT  486
            |||||||||||||||||||||||||||||||||||||||||||||||
Sbjct  420  CTAGATAATCGTAGAGGGGCGGATGTAGCCAAGTGGATCAAGGCAGT  466


>MF348389.1 Echinacea pallida isolate AD0EE57 photosystem II protein D1 (psbA)
gene, partial cds; and psbA-trnH intergenic spacer, partial
sequence; chloroplast
Length=466

 Score = 813 bits (440),  Expect = 0.0
 Identities = 459/467 (98%), Gaps = 5/467 (1%)
 Strand=Plus/Plus

Query  24   ATAATTTCCCTCTAGACTTAGCTGCTATTGAAGCTCCATATACAAATGGATAAGCCTTTG  83
            ||||||||||||||||||||||||||||||||||||||| ||||||||||||||||||||
Sbjct  1    ATAATTTCCCTCTAGACTTAGCTGCTATTGAAGCTCCATCTACAAATGGATAAGCCTTTG  60

Query  84   GTCTGATTGTATAGGAGTTTTTGAACTAAAAAAGGAGCAATAACGCCCTCTTGATAAAAC  143
            ||||||||||||||||||||||||||||||||||||||||||||||||||||||||||||
Sbjct  61   GTCTGATTGTATAGGAGTTTTTGAACTAAAAAAGGAGCAATAACGCCCTCTTGATAAAAC  120

Query  144  AAGAGGGAAGCTATTGCTCCttttttttATTTAGTACTATTTGCCTTACATAGTTTCTTT  203
```

```
                ||||||||||||||||||||| ||||||||||||||||||||||||||||||||||||||||
Sbjct  121   AAGAGGGAAGCTATTGCTCC-TTTTTTTATTTAGTACTATTTGCCTTACATAGTTTCTTT  179

Query  204   AAAAATAACAAGTACTTTTTATAGTTTGGTTCGATTTGCGTGtttttctttgtattcat  263
             |||||||| |||||||||||||||||||||||||||||||||||||||||||||||||||
Sbjct  180   AAAAATAATAAGTACTTTTTATAGTTTGGTTCGATTTGCGTGTTTTTCTTTGTATTCAT  239

Query  264   ttatattataggtttctatatcatatatttttcccaatcttttatgaagttttatttACA  323
             ||||||||||||||||||||||||||||||||||||||||||||||||||||||||||||
Sbjct  240   TTATATTATAGGTTTCTATATCATATATTTTTCCCAATCTTTTATGAAGTTTTATTTACA  299

Query  324   ATTTAATTTCAATCGAAAATAGAGAAAAATGACAATTTTGCTTTTTGATTTATATTGATT  383
             ||||||||||||||||||||||||||||||||||||||||||||||||||||||||||||
Sbjct  300   ATTTAATTTCAATCGAAAATAGAGAAAAATGACAATTTTGCTTTTTGATTTATATTGATT  359

Query  384   AAATCAAAAT----AAGAAAGAATATGCtttttttATGTTAATGGAAAAATATAGTAATA  439
             |||| |||||    ||||||||||||||||||||||||||||||||||||||||||||||
Sbjct  360   AAATAAAAATAAGAAAGAAAGAATATGCTTTTTTTATGTTAATGGAAAAATATAGTAATA  419

Query  440   CTAGATAATCGTAGAGGGGCGGATGTAGCCAAGTGGATCAAGGCAGT  486
             |||||||||||||||||||||||||||||||||||||||||||||||
Sbjct  420   CTAGATAATCGTAGAGGGGCGGATGTAGCCAAGTGGATCAAGGCAGT  466


>NC_081940.1 Enydra sessilis chloroplast, complete genome
 OR069728.1 Enydra sessilis chloroplast, complete genome
Length=151340

 Score = 797 bits (431),  Expect = 0.0
 Identities = 486/512 (95%), Gaps = 6/512 (1%)
 Strand=Plus/Minus

Query  2     GTTATGCATGAACGTAATGCTCATAATTTCCCTCTAGACTTAGCTGCTATTGAAGCTCCA  61
             ||||||||||||||||||||||||||||||||||||||||||||||||||||||||||||
Sbjct  548   GTTATGCATGAACGTAATGCTCATAATTTCCCTCTAGACTTAGCTGCTATTGAAGCTCCA  489

Query  62    TATACAAATGGATAAGCCTTTGGTCTGATTGTATAGGAGTTTTTGAACTAAAAAAGGAGC  121
             | |||||||||||||| |||||||||||||||||||||||||||||||||||||||||||
Sbjct  488   TCTACAAATGGATAAGACTTTGGTCTGATTGTATAGGAGTTTTTGAACTAAAAAAGGAGC  429

Query  122   AATAACGCCCTCTTGATAAAACAAGAGGGAAGCTATTGCTCCtttttttATTTAGTACT  181
             ||||||||||||||||||||||||||||||||||||||||||||||||||||||||||||
Sbjct  428   AATAACGCCCTCTTGATAAAACAAGAGGGAAGCTATTGCTCCTTTTTTTTATTTAGTACT  369

Query  182   ATTTGCCTTACATAGTTTCTTTAAAAATAACAAGTACTTTTTATAGTTTGGTTCGATTTG  241
             |||||||||||||||||||||||||||||||||| ||||||| |||||||||||||||||
Sbjct  368   ATTTGCCTTACATAGTTTCTTTAAAAATAACAAGGACTTTTTCTAGTTTGGTTCGATTTG  309

Query  242   CGTGtttttctttgtattcatttatattataggtttctatatcatatatttttccc-aa  300
             |||||||| ||||||||||||||||||||||||||||||||||||| || ||||||| ||
Sbjct  308   CGTGTTTTATCTTTGTATTCATTTATATTATAGGTTTCTATATCATTTACTTTTCCCCAA  249

Query  301   tcttt-tatgaagttttatttACAATTTAATTTCAATCGAAAATAGAGAAAAATGACAAT  359
             | ||| ||||||||||||||| ||||| ||||||||||||||||||| |||||||| |||
Sbjct  248   TATTTGTATGAAGTTTTATTTCCAATTCAATTTCAATCGAAAATAGATAAAAATGAAAAT  189

Query  360   TTTGCTTTTTGATTTATATTGATTAAATCAAAATAAGAAAGAATATGCttttttt-ATGT  418
```

```
               ||||||| || ||| |  |||||| | | |||| ||||||||||||||||||||| ||||
Sbjct  188    TTTGCTTATTTATT-ACTTTGATTTATT-AAAA-AAGAAAGAATATGCTTTTTTTATGT  132

Query  419    TAATGGAAAAATATAGTAATACTAGATAATCGTAGAGGGGCGGATGTAGCCAAGTGGATC  478
              |||||||||||||||||||||||||| ||| |||||||||||||||||||||||||||||
Sbjct  131    TAATGGAAAAATATAGTAATACTAGAGAATAGTAGAGGGGCGGATGTAGCCAAGTGGATC  72

Query  479    AAGGCAGTGGATTGTGAATCCACCATGCGCGG  510
              ||||||||||||||||||||||||||||||||
Sbjct  71     AAGGCAGTGGATTGTGAATCCACCATGCGCGG  40


>LS999859.1 Echinacea angustifolia DNA containing psbA-trnH IGS, specimen
voucher FEM_DBE_015
Length=437

 Score = 787 bits (426),  Expect = 0.0
 Identities = 435/439 (99%), Gaps = 2/439 (0%)
 Strand=Plus/Plus

Query  51     TTGAAGCTCCATATACAAATGGATAAGCCTTTGGTCTGATTGTATAGGAGTTTTTGAACT  110
              |||||||||||| |||||||||||||||||||||||||||||||||||||||||||||||
Sbjct  1      TTGAAGCTCCATCTACAAATGGATAAGCCTTTGGTCTGATTGTATAGGAGTTTTTGAACT  60

Query  111    AAAAAAGGAGCAATAACGCCCTCTTGATAAAACAAGAGGGAAGCTATTGCTCCttttttt  170
              ||||||||||||||||||||||||||||||||||||||||||||||||||||| ||||||
Sbjct  61     AAAAAAGGAGCAATAACGCCCTCTTGATAAAACAAGAGGGAAGCTATTGCTCC-TTTTTT  119

Query  171    tATTTAGTACTATTTGCCTTACATAGTTTCTTTAAAAATAACAAGTACTTTTTATAGTTT  230
              ||||||||||||||||||||||||||||||||||||||||||||||||||||||||||||
Sbjct  120    TATTTAGTACTATTTGCCTTACATAGTTTCTTTAAAAATAACAAGTACTTTTTATAGTTT  179

Query  231    GGTTCGATTTGCGTGtttttctttgtattcatttatattataggtttctatatcatata  290
              ||||||||||||||||||||||||||||||||||||||||||||||||||||||||||||
Sbjct  180    GGTTCGATTTGCGTGTTTTTCTTTGTATTCATTTATATTATAGGTTTCTATATCATATA  239

Query  291    tttttcccaatcttttatgaagttttatttACAATTTAATTTCAATCGAAAATAGAGAAA  350
              ||||||||||||||||||||||||||||||||||||||||||||||||||||||||||||
Sbjct  240    TTTTTCCCAATCTTTTATGAAGTTTTATTTACAATTTAATTTCAATCGAAAATAGAGAAA  299

Query  351    AATGACAATTTTGCTTTTTGATTTATATTGATTAAATCAAAATAAGAAAGAATATGCttt  410
              ||||||||||||||||||| ||||||||||||||||||||||||||||||||||||| ||
Sbjct  300    AATGACAATTTTGCTTTTTTATTTATATTGATTAAATCAAAATAAGAAAGAATATGC-TT  358

Query  411    ttttATGTTAATGGAAAAATATAGTAATACTAGATAATCGTAGAGGGGCGGATGTAGCCA  470
              ||||||||||||||||||||||||||||||||||||||||||||||||||||||||||||
Sbjct  359    TTTTATGTTAATGGAAAAATATAGTAATACTAGATAATCGTAGAGGGGCGGATGTAGCCA  418

Query  471    AGTGGATCAAGGCAGTGGA  489
              |||||||||||||||||||
Sbjct  419    AGTGGATCAAGGCAGTGGA  437


>MZ292978.1 Acmella paniculata chloroplast, complete genome
Length=152117

 Score = 754 bits (408),  Expect = 0.0
```

```
 Identities = 481/515 (93%), Gaps = 10/515 (2%)
 Strand=Plus/Minus

Query  2    GTTATGCATGAACGTAATGCTCATAATTTCCCTCTAGACTTAGCTGCTATTGAAGCTCCA  61
            ||||||||||||||||||||||||||||||||||||||||||||||||||||||||||||
Sbjct  531  GTTATGCATGAACGTAATGCTCATAATTTCCCTCTAGACTTAGCTGCTATTGAAGCTCCA  472

Query  62   TATACAAATGGATAAGCCTTTGGTCTGATTGTATAGGAGTTTTTGAACTAAAAAAGGAGC  121
            | ||| |||||||||| |||||||||||||||||||||||||||||||||||||||||||
Sbjct  471  TCTACCAATGGATAAGACTTTGGTCTGATTGTATAGGAGTTTTTGAACTAAAAAAGGAGC  412

Query  122  AATAAC-GCCCTCTTGATAAAACAAGAGGGAAGCTATTGCTCCtttttttATTTAGTAC  180
            |||| |  |||||||| |  | ||||||||  | |||||||||||||||| |||||||||
Sbjct  411  AATAGCTTCCCTCTTGTTTTATCAAGAGGG-CGTTATTGCTCCTTTTTTT-ATTTAGTAC  354

Query  181  TATTTGCCTTACATAGTTTCTTTAAAAATAACAAGTACTTTTTATAGTTTGGTTCGATTT  240
            ||||||||||||||||||||||||||||||||||||||||||||||||||||||||||||
Sbjct  353  TATTTGCCTTACATAGTTTCTTTAAAAATAACAAGTACTTTTTATAGTTTGGTTCGATTT  294

Query  241  GCGTGttttttctttgtattcatttatattataggtttctatatcatatatttttcccaa  300
            |||||||||||||||||||||||||||||||||||||| ||||||||||| |||||||||
Sbjct  293  GCGTGTTTTTTCTTTGTATTCATTTATATTATAGGTTTATATATCATATACTTTTCCCAA  234

Query  301  tcttttatgaagttttatttACAATTTAATTTCAATCGAAAATAGAGAAAAATGACAATT  360
            ||||||||||||| |||||||||||| ||||||||||||||||||| |||||||||||||
Sbjct  233  TCTTTTATGAAGTGTTATTTACAATTCAATTTCAATCGAAAATAGATAAAAATGACAATT  174

Query  361  TTGCTTTTTGATTTATATTGATTAAATCAAAATAAGAAAGAA-TA-T--GC-tttttttA  415
            |||||| ||  ||||| || |  |||| ||||||||||| || || |  || ||||||||
Sbjct  173  TTGCTTATTTTTTTAT-TTAAG-AAATAAAAATAAGAAATAAATAATATGCTTTTTTTTA  116

Query  416  TGTTAATGGAAAAATATAGTAATACTAGATAATCGTAGAGGGGCGGATGTAGCCAAGTGG  475
            ||||||||||||||||||||||||||||||||| ||||||||||||||||||||||||||
Sbjct  115  TGTTAATGGAAAAATATAGTAATACTAGATAATAGTAGAGGGGCGGATGTAGCCAAGTGG  56

Query  476  ATCAAGGCAGTGGATTGTGAATCCACCATGCGCGG  510
            |||||||||||||||||||||||||||||||||||
Sbjct  55   ATCAAGGCAGTGGATTGTGAATCCACCATGCGCGG  21


 Score = 110 bits (59),  Expect = 3e-19
 Identities = 59/59 (100%), Gaps = 0/59 (0%)
 Strand=Plus/Plus

Query  111  AAAAAAGGAGCAATAACGCCCTCTTGATAAAACAAGAGGGAAGCTATTGCTCCtttttt  169
            |||||||||||||||||||||||||||||||||||||||||||||||||||||||||||
Sbjct  364  AAAAAAGGAGCAATAACGCCCTCTTGATAAAACAAGAGGGAAGCTATTGCTCCTTTTTT  422


>NC_035875.1 Ambrosia artemisiifolia chloroplast, complete genome
 MF362689.1 Ambrosia artemisiifolia chloroplast, complete genome
Length=152215

 Score = 749 bits (405),  Expect = 0.0
 Identities = 482/516 (93%), Gaps = 18/516 (3%)
 Strand=Plus/Minus
```

```
Query  2    GTTATGCATGAACGTAATGCTCATAATTTCCCTCTAGACTTAGCTGCTATTGAAGCTCCA  61
            ||||||||||||||||||||||||||||||||||||||||||||||||||||||||||||
Sbjct  707  GTTATGCATGAACGTAATGCTCATAATTTCCCTCTAGACTTAGCTGCTATTGAAGCTCCA  648

Query  62   TATACAAATGGATAAGCCTTTGGTCTGATTGTATAGGAGTTTTTGAACTAAAAAAGGAGC  121
            | |||||||||||||| |||||||||||||||||||||||||||||||||||||||||||
Sbjct  647  TCTACAAATGGATAAGACTTTGGTCTGATTGTATAGGAGTTTTTGAACTAAAAAAGGAGC  588

Query  122  AATAACGCCCTCTTGATAAAACAAGAGGGAAGCTATTGCTCCttttttttATTTAGTACT  181
            ||||||| ||||||||||||||||||||||||||||||||||||||||||||||||||||
Sbjct  587  AATAACGTCCTCTTGATAAAACAAGAGGGAAGCTATTGCTCCTTTTTTTTATTTAGTACT  528

Query  182  ATTTGCCTTACATAGTTTCTTTAAAAATAACAAGTACTTTTATAGTTTGGTTCGATTTG  241
            |||||||||||||||||||||||||||||         ||||||||||||||||||||||
Sbjct  527  ATTTGCCTTACATAGTTTCTTTAAAAATA---------TTTTATAGTTTGGTTCGATTTG  477

Query  242  CGTGttttttctttgtattcatttatattataggtttctatatcatatatttttcccaat  301
            ||||||||||||||||||||||||||||||||||||||||||||||||| ||||||||||
Sbjct  476  CGTGTTTTTTCTTTGTATTCATTTATATTATAGGTTTCTATATCATATACTTTTCCCAAT  417

Query  302  cttttatgaagttttatttACAATTTAATTTCAATCGAAAATAGAGAAAAATGACAATTT  361
            ||||||||||||||||||||||||| ||||||||||||||||||| ||||||||||||||
Sbjct  416  CTTTTATGAAGTTTTATTTACAATTCAATTTCAATCGAAAATAGACAAAAATGACAATTT  357

Query  362  TGCTTTTTGATT--TA-TATTGATTAAATCAAAA---T-AAGAAAGAATATGCttttttt  414
            | ||| || |||  |  |||| ||||||| ||||   | |||||| ||||||||||||
Sbjct  356  TTCTTATTTATTACTTTTATTTATTAAATAAAAAGAATAAAGAAATAATATGCTTTTTG-  298

Query  415  ATGTTAATGGAAAAATATAGTAATACTAGATAATCGTAGAGGGGCGGATGTAGCCAAGTG  474
            || ||||||||||||||||||||||||||||||  |||||||||||||||||||||||||
Sbjct  297  AT-TTAATGGAAAAATATAGTAATACTAGATAACAGTAGAGGGGCGGATGTAGCCAAGTG  239

Query  475  GATCAAGGCAGTGGATTGTGAATCCACCATGCGCGG  510
            ||||||||||||||||||||||||||||||||||||
Sbjct  238  GATCAAGGCAGTGGATTGTGAATCCACCATGCGCGG  203


>NC_053299.1 Iostephane heterophylla voucher Schilling 94 chloroplast, complete
genome
 MT700542.1 Iostephane heterophylla chloroplast, complete genome
Length=151495

 Score = 749 bits (405),  Expect = 0.0
 Identities = 485/521 (93%), Gaps = 16/521 (3%)
 Strand=Plus/Minus

Query  2    GTTATGCATGAACGTAATGCTCATAATTTCCCTCTAGACTTAGCTGCTATTGAAGCTCCA  61
            ||||||||||||||||||||||||||||||||||||||||||||||||||||||||||||
Sbjct  537  GTTATGCATGAACGTAATGCTCATAATTTCCCTCTAGACTTAGCTGCTATTGAAGCTCCA  478

Query  62   TATACAAATGGATAAGCCTTTGGTCTGATTGTATAGGAGTTTTTGAACTAAAAAAGGAGC  121
            | |||||||||||||| |||||||||||||||||||||||||||||||||||||||||||
Sbjct  477  TCTACAAATGGATAAGACTTTGGTCTGATTGTATAGGAGTTTTTGAACTAAAAAAGGAGC  418

Query  122  AATAAC-GCCCTCTTGATAAAACAAGAGGGAAGCTATTGCTCCttttttttATTTAGTAC  180
            |||| |  ||||||||||||||||||||||  | |||||||||||||||| |||||||||
Sbjct  417  AATAGCTTCCCTCTTGATAAAACAAGAGGG-CGTTATTGCTCCTTTTTTT-ATTTAGTAC  360
```

```
Query  181   TATTTGCCTTACATAGTTTCTTTAAA-------AATAACAAGTACTTTTTATAGTTTGGT  233
             ||||||||||||||||||||||||||       |||||||||||||||||||||||||||
Sbjct  359   TATTTGCCTTACATAGTTTCTTTAAACTTTAAAAATAACAAGTACTTTTTATAGTTTGGT  300

Query  234   TCGATTTGCGTGttttttctttgtattcatttatattataggtttctatatcatatattt  293
             ||||||||| |||||||| |||||||||||||||||||||||||||||||||||||||||
Sbjct  299   TCGATTTGCTTGTTTTTTATTTGTATTCATTTATATTATAGGTTTCTATATCATATATTT  240

Query  294   ttcccaatcttttatgaagtttt-atttACAATTTAATTTCAATCGAAAATAGAGAAAAA  352
             |||| | ||||||||||||||||  |||||||||||||||||||||||||||||  ||||
Sbjct  239   TTCCTACTCTTTTATGAAGTTTTTTTTTACAATTTAATTTCAATCGAAAATAGA-TAAAA  181

Query  353   TGACAATTTTGCTT--TT-TGATTTATATTGATTAAATCAAAATAAGAAAGAATATGCtt  409
             ||||||||||||||  || | | || |||| ||| ||| ||||| |||||||||||||||
Sbjct  180   TGACAATTTTGCTTATTTATTACTTTTATTTATTCAATAAAAATGAGAAAGAATATGCTT  121

Query  410   ttttATGTTAATGGAAAAATATAGTAATACTAGATAATCGTAGAGGGGCGGATGTAGCC  469
             |||| ||||||||||||||||||||||||||||||||| |||||||||||||||||||||
Sbjct  120   TTTT-ATGTTAATGGAAAAATATAGTAATACTAGATAATAGTAGAGGGGCGGATGTAGCC  62

Query  470   AAGTGGATCAAGGCAGTGGATTGTGAATCCACCATGCGCGG  510
             |||||||||||||||||||||||||||||||||||||||||
Sbjct  61    AAGTGGATCAAGGCAGTGGATTGTGAATCCACCATGCGCGG  21


>MG019037.1 Ambrosia artemisiifolia chloroplast, complete genome
Length=152223

 Score = 749 bits (405),  Expect = 0.0
 Identities = 483/517 (93%), Gaps = 19/517 (4%)
 Strand=Plus/Minus

Query  2     GTTATGCATGAACGTAATGCTCATAATTTCCCTCTAGACTTAGCTGCTATTGAAGCTCCA  61
             ||||||||||||||||||||||||||||||||||||||||||||||||||||||||||||
Sbjct  535   GTTATGCATGAACGTAATGCTCATAATTTCCCTCTAGACTTAGCTGCTATTGAAGCTCCA  476

Query  62    TATACAAATGGATAAGCCTTTGGTCTGATTGTATAGGAGTTTTTGAACTAAAAAAGGAGC  121
             | |||||||||||||| |||||||||||||||||||||||||||||||||||||||||||
Sbjct  475   TCTACAAATGGATAAGACTTTGGTCTGATTGTATAGGAGTTTTTGAACTAAAAAAGGAGC  416

Query  122   AATAACG-CCCTCTTGATAAAACAAGAGGGAAGCTATTGCTCCttttttttATTTAGTAC  180
             ||||||| ||||||||||||||||||||||||||||||||||||||||||||||||||||
Sbjct  415   AATAACGTCCCTCTTGATAAAACAAGAGGGAAGCTATTGCTCCTTTTTTTTATTTAGTAC  356

Query  181   TATTTGCCTTACATAGTTTCTTTAAAAATAACAAGTACTTTTTATAGTTTGGTTCGATTT  240
             ||||||||||||||||||||||||||||||         |||||||||||||||||||||
Sbjct  355   TATTTGCCTTACATAGTTTCTTTAAAAATA---------TTTTATAGTTTGGTTCGATTT  305

Query  241   GCGTGttttttctttgtattcatttatattataggtttctatatcatatatttttcccaa  300
             |||||||||||||||||||||||||||||||||||||||||||||||||| |||||||||
Sbjct  304   GCGTGTTTTTTCTTTGTATTCATTTATATTATAGGTTTCTATATCATATACTTTTCCCAA  245

Query  301   tcttttatgaagttttatttACAATTTAATTTCAATCGAAAATAGAGAAAAATGACAATT  360
             |||||||||||||||||||||||||| ||||||||||||||||||| |||||||||||||
Sbjct  244   TCTTTTATGAAGTTTTATTTACAATTCAATTTCAATCGAAAATAGACAAAAATGACAATT  185
```

```
Query  361   TTGCTTTTTGATT--TA-TATTGATTAAATCAAAA---T-AAGAAAGAATATGCtttttt  413
             || ||| || |||  |  |||| ||||||| ||||   | |||||| ||||||||||||
Sbjct  184   TTTCTTATTTATTACTTTTATTTATTAAATAAAAAGAATAAAGAAATAATATGCTTTTTG  125

Query  414   tATGTTAATGGAAAAATATAGTAATACTAGATAATCGTAGAGGGGCGGATGTAGCCAAGT  473
              || ||||||||||||||||||||||||||||||  ||||||||||||||||||||||||
Sbjct  124   -AT-TTAATGGAAAAATATAGTAATACTAGATAACAGTAGAGGGGCGGATGTAGCCAAGT  67

Query  474   GGATCAAGGCAGTGGATTGTGAATCCACCATGCGCGG  510
             |||||||||||||||||||||||||||||||||||||
Sbjct  66    GGATCAAGGCAGTGGATTGTGAATCCACCATGCGCGG  30


>NC_036810.2 Ambrosia trifida chloroplast, complete genome
 MG029118.2 Ambrosia trifida chloroplast, complete genome
Length=152040

 Score = 749 bits (405),  Expect = 0.0
 Identities = 482/516 (93%), Gaps = 18/516 (3%)
 Strand=Plus/Minus

Query  2     GTTATGCATGAACGTAATGCTCATAATTTCCCTCTAGACTTAGCTGCTATTGAAGCTCCA  61
             ||||||||||||||||||||||||||||||||||||||||||||||||||||||||||||
Sbjct  534   GTTATGCATGAACGTAATGCTCATAATTTCCCTCTAGACTTAGCTGCTATTGAAGCTCCA  475

Query  62    TATACAAATGGATAAGCCTTTGGTCTGATTGTATAGGAGTTTTTGAACTAAAAAAGGAGC  121
             | |||||||||||||| |||||||||||||||||||||||||||||||||||||||||||
Sbjct  474   TCTACAAATGGATAAGACTTTGGTCTGATTGTATAGGAGTTTTTGAACTAAAAAAGGAGC  415

Query  122   AATAACGCCCTCTTGATAAAACAAGAGGGAAGCTATTGCTCCttttttttATTTAGTACT  181
             ||||||| ||||||||||||||||||||||||||||||||||||||||||||||||||||
Sbjct  414   AATAACGTCCTCTTGATAAAACAAGAGGGAAGCTATTGCTCCTTTTTTTTATTTAGTACT  355

Query  182   ATTTGCCTTACATAGTTTCTTTAAAAATAACAAGTACTTTTATAGTTTGGTTCGATTTG  241
             |||||||||||||||||||||||||||||         ||||||||||||||||||||||
Sbjct  354   ATTTGCCTTACATAGTTTCTTTAAAAATA---------TTTTATAGTTTGGTTCGATTTG  304

Query  242   CGTGtttttctttgtattcatttatattataggtttctatatcatatattttcccaat  301
             ||||||||||||||||||||||||||||||||||||||||||||||||| ||||||||||
Sbjct  303   CGTGTTTTTCTTTGTATTCATTTATATTATAGGTTTCTATATCATATACTTTTCCCAAT  244

Query  302   cttttatgaagttttatttACAATTTAATTTCAATCGAAAATAGAGAAAAATGACAATTT  361
             ||||||||||||||||||||||||| ||||||||||||||||||| ||||||||||||||
Sbjct  243   CTTTTATGAAGTTTTATTTACAATTCAATTTCAATCGAAAATAGACAAAAATGACAATTT  184

Query  362   TGCTTTTTGATT--TA-TATTGATTAAATCAAAA---T-AAGAAAGAATATGCttttttt  414
             | ||| || |||  |  |||| ||||||| ||||   | |||||| ||||||||||||
Sbjct  183   TTCTTATTTATTACTTTTATTTATTAAATAAAAAGAATAAAGAAATAATATGCTTTTTG-  125

Query  415   ATGTTAATGGAAAAATATAGTAATACTAGATAATCGTAGAGGGGCGGATGTAGCCAAGTG  474
             || ||||||||||||||||||||||||||||||  |||||||||||||||||||||||||
Sbjct  124   AT-TTAATGGAAAAATATAGTAATACTAGATAACAGTAGAGGGGCGGATGTAGCCAAGTG  66

Query  475   GATCAAGGCAGTGGATTGTGAATCCACCATGCGCGG  510
             ||||||||||||||||||||||||||||||||||||
Sbjct  65    GATCAAGGCAGTGGATTGTGAATCCACCATGCGCGG  30
```

```
>MT793855.1 Mikania sylvatica chloroplast, complete genome
Length=152099

 Score = 747 bits (404),  Expect = 0.0
 Identities = 484/520 (93%), Gaps = 16/520 (3%)
 Strand=Plus/Minus

Query  2    GTTATGCATGAACGTAATGCTCATAATTTCCCTCTAGACTTAGCTGCTATTGAAGCTCCA  61
            ||||||||||||||||||||||||||||||||||||||||||||||||||||||||||||
Sbjct  535  GTTATGCATGAACGTAATGCTCATAATTTCCCTCTAGACTTAGCTGCTATTGAAGCTCCA  476

Query  62   TATACAAATGGATAAGCCTTTGGTCTGATTGTATAGGAGTTTTTGAACTAAAAAAGGAGC  121
            | |||||||||||||| |||||||||||||||||||||||||||||||||||||||||||
Sbjct  475  TCTACAAATGGATAAGACTTTGGTCTGATTGTATAGGAGTTTTTGAACTAAAAAAGGAGC  416

Query  122  AATAACGCCCTCTTGATAAAACAAGAGGGAAGCTATTGCTCCttttttttATTTAGTACT  181
            |||||||||||||||||| |||||||||||||||||||||||||||||| ||||||||||
Sbjct  415  AATAACGCCCTCTTGATACAACAAGAGGGAAGCTATTGCTCCTTTTTTT-ATTTAGTACT  357

Query  182  ATTTGCCTTACATAGTTTCTTTAAAAATAAC-----------AAGTACTTTTTATAGTTT  230
            |||||||||||||||||||||||||||||||           ||| ||||||||||||||
Sbjct  356  ATTTGCCTTACATAGTTTCTTTAAAAATAACTTAAAAAAAACAAGGACTTTTTATAGTTT  297

Query  231  GGTTCGATTTGCGTGttttttctttgtattcatttatattataggtttctatatcatata  290
            |||||||||||||||||||||||||||||||||||||||||||||||||||||| |||||
Sbjct  296  GGTTCGATTTGCGTGTTTTTTCTTTGTATTCATTTATATTATAGGTTTCTATATAATATA  237

Query  291  tttttcccaatcttttatgaagttttatttACAATTTAATTTCAATCGAAAATAGAGAAA  350
            ||||||||||||||||||||||||| |||| ||||| |||||||||||||||||||  ||
Sbjct  236  TTTTTCCCAATCTTTTATGAAGTTTGATTTCCAATTCAATTTCAATCGAAAATAGATCAA  177

Query  351  AATGACAATTTTGCTTTTTGATTTATATTGATTAAATCAAAATAAGAAAGAATATGCttt  410
            ||||| |||||||||| || ||| |  || ||| | |  |||||||||| |||||| |||
Sbjct  176  AATGAAAATTTTGCTTATTTATT-ACTTTTATTTATT--AAATAAGAAATAATATG-TTT  121

Query  411  ttttATGTTAATGGAAAAATATAGTAATACTAGATAATCGTAGAGGGGCGGATGTAGCCA  470
            |||||||||||||||||||||||||||||||||||||| |||||||||||||||||||||
Sbjct  120  TTTTATGTTAATGGAAAAATATAGTAATACTAGATAATAGTAGAGGGGCGGATGTAGCCA  61

Query  471  AGTGGATCAAGGCAGTGGATTGTGAATCCACCATGCGCGG  510
            ||||||||||||||||||||||||||||||||||||||||
Sbjct  60   AGTGGATCAAGGCAGTGGATTGTGAATCCACCATGCGCGG  21


>NC_053285.1 Aldama linearis voucher Schilling 70 chloroplast, complete genome
 MN337896.1 Aldama linearis chloroplast, complete genome
Length=151314

 Score = 737 bits (399),  Expect = 0.0
 Identities = 486/525 (93%), Gaps = 17/525 (3%)
 Strand=Plus/Minus

Query  2    GTTATGCATGAACGTAATGCTCATAATTTCCCTCTAGACTTAGCTGCTATTGAAGCTCCA  61
            ||||||||||||||||||||||||||||||||||||||||||||||||||||||||||||
Sbjct  547  GTTATGCATGAACGTAATGCTCATAATTTCCCTCTAGACTTAGCTGCTATTGAAGCTCCA  488
```

```
Query  62   TATACAAATGGATAAGCCTTTGGTCTGATTGTATAGGAGTTTTTGAACTAAAAAAGGAGC  121
            | |||||||||||||| |||||||||||||||||||||||||||||||||||||||||||
Sbjct  487  TCTACAAATGGATAAGACTTTGGTCTGATTGTATAGGAGTTTTTGAACTAAAAAAGGAGC  428

Query  122  AATAACGCCCTCTTGATAAAACAAGAGGGAAGCTATTGCTCCttttttttATTTAGTACT  181
            ||||||||||||||| |  | |||||||||||||||||||||||||||||||||||||||
Sbjct  427  AATAACGCCCTCTTGTTTTATCAAGAGGGAAGCTATTGCTCCTTTTTTTTATTTAGTACT  368

Query  182  ATTTGCCTTACATAGTTTCTTTAAA-------AATAACAAGTACTTTTTATAGTTTGGTT  234
            |||||||||||||||||||||||||       ||||||||||||||||||||||||||||
Sbjct  367  ATTTGCCTTACATAGTTTCTTTAAACTTTAAAAATAACAAGTACTTTTTATAGTTTGGTT  308

Query  235  CGATTTGCGTGttttttctttgtattcatttatattataggtttctatatcatatatttt  294
            |||||||  |||||||| |||||||||||||||||||||||||||||||||||||| |||
Sbjct  307  CGATTTGTTTGTTTTTTATTTGTATTCATTTATATTATAGGTTTCTATATCATATACTTT  248

Query  295  t-c----cc-aatcttttatgaagttttatttACAATTTAATTTCAATCGAAAATAGAGA  348
            | |    || |||||||||||||||||| |||||||||| | |||||||||||||||| |
Sbjct  247  TACTTTTCCTAATCTTTTATGAAGTTTTTTTTACAATTTCAATTCAATCGAAAATAGATA  188

Query  349  AAAATGACAATTTTGCTTTTTGATT--TA-TATTGATTAAATCAAAATAAGAAAGAATAT  405
            |||||||||||||||||| || |||  |  |||| ||| ||| ||||| |||||||||||
Sbjct  187  AAAATGACAATTTTGCTTATTTATTACTTTATTTATTCAATAAAAATGAGAAAGAATAT  128

Query  406  GCtttttttATGTTAATGGAAAAATATAGTAATACTAGATAATCGTAGAGGGGCGGATGT  465
            |||||||| |||||||||||||||||||||||||||||||||| ||||||||||||||||
Sbjct  127  GCTTTTTT-ATGTTAATGGAAAAATATAGTAATACTAGATAATAGTAGAGGGGCGGATGT  69

Query  466  AGCCAAGTGGATCAAGGCAGTGGATTGTGAATCCACCATGCGCGG  510
            |||||||||||||||||||||||||||||||||||||||||||||
Sbjct  68   AGCCAAGTGGATCAAGGCAGTGGATTGTGAATCCACCATGCGCGG  24


>MT793838.1 Mikania lehmannii chloroplast, complete genome
Length=152062

 Score = 736 bits (398),  Expect = 0.0
 Identities = 482/520 (93%), Gaps = 15/520 (3%)
 Strand=Plus/Minus

Query  2    GTTATGCATGAACGTAATGCTCATAATTTCCCTCTAGACTTAGCTGCTATTGAAGCTCCA  61
            ||||||||||||||||||||||||||||||||||||||||||||||||||||||||||||
Sbjct  536  GTTATGCATGAACGTAATGCTCATAATTTCCCTCTAGACTTAGCTGCTATTGAAGCTCCA  477

Query  62   TATACAAATGGATAAGCCTTTGGTCTGATTGTATAGGAGTTTTTGAACTAAAAAAGGAGC  121
            | |||||||||||||| |||||||||||||||||||||||||||||||||||||||||||
Sbjct  476  TCTACAAATGGATAAGACTTTGGTCTGATTGTATAGGAGTTTTTGAACTAAAAAAGGAGC  417

Query  122  AATAACGCCCTCTTGATAAAACAAGAGGGAAGCTATTGCTCCttttttttATTTAGTACT  181
            |||||||||||||||||| |||||||||||||||||||||||||||||| ||||||||||
Sbjct  416  AATAACGCCCTCTTGATACAACAAGAGGGAAGCTATTGCTCCTTTTTTT-ATTTAGTACT  358

Query  182  ATTTGCCTTACATAGTTTCTTTAAAAATAAC-----------AAGTACTTTTTATAGTTT  230
            |||||||||||||||||||||||||||||||           ||| ||||||||||||||
Sbjct  357  ATTTGCCTTACATAGTTTCTTTAAAAATAACTTAAAAATAACAAGGACTTTTTATAGTTT  298

Query  231  GGTTCGATTTGCGTGttttttctttgtattcatttatattataggtttctatatcatata  290
```

```
            |||||||||||||||||||||||||||||||||||||||||||||||||||||| |||||
Sbjct  297  GGTTCGATTTGCGTGTTTTTTCTTTGTATTCATTTATATTATAGGTTTCTATATAATATA  238

Query  291  tttttcccaatcttttatgaagttttatttACAATTTAATTTCAATCGAAAATAGAGAAA  350
            ||||||||||||||||||||||||| |||| ||||| |||||||||||||||||||  ||
Sbjct  237  TTTTTCCCAATCTTTTATGAAGTTTGATTTCCAATTCAATTTCAATCGAAAATAGATCAA  178

Query  351  AATGACAATTTTGCTTTTTGATTTATATTGATTAAATCAAAATAAGAAAGAATATGCttt  410
            ||||| |||||||||| || ||| |  || |||   |  |||||||||| |||||| |||
Sbjct  177  AATGAAAATTTTGCTTATTTATT-ACTTTTATTTCTT--AAATAAGAAATAATATGTTTT  121

Query  411  ttttATGTTAATGGAAAAATATAGTAATACTAGATAATCGTAGAGGGGCGGATGTAGCCA  470
            ||| |||||||||||||||||||||||||||||||||| |||||||||||||||||||||
Sbjct  120  TTTGATGTTAATGGAAAAATATAGTAATACTAGATAATAGTAGAGGGGCGGATGTAGCCA  61

Query  471  AGTGGATCAAGGCAGTGGATTGTGAATCCACCATGCGCGG  510
            ||||||||||||||||||||||||||||||||||||||||
Sbjct  60   AGTGGATCAAGGCAGTGGATTGTGAATCCACCATGCGCGG  21
```

>MF037494.1 Echinacea sanguinea voucher Bengtson9 trnH-psbA intergenic spacer region, partial sequence; chloroplast
Length=407

Score = 730 bits (395), Expect = 0.0
Identities = 404/408 (99%), Gaps = 2/408 (0%)
Strand=Plus/Plus

```
Query  45   CTGCTATTGAAGCTCCATATACAAATGGATAAGCCTTTGGTCTGATTGTATAGGAGTTTT  104
            |||||||||||||||||| |||||||||||||||||||||||||||||||||||||||||
Sbjct  1    CTGCTATTGAAGCTCCATCTACAAATGGATAAGCCTTTGGTCTGATTGTATAGGAGTTTT  60

Query  105  TGAACTAAAAAAGGAGCAATAACGCCCTCTTGATAAAACAAGAGGGAAGCTATTGCTCCt  164
            |||||||||||||||||||||||||||||||||||||||||||||||||||||||||||
Sbjct  61   TGAACTAAAAAAGGAGCAATAACGCCCTCTTGATAAAACAAGAGGGAAGCTATTGCTCC-  119

Query  165  tttttttATTTAGTACTATTTGCCTTACATAGTTTCTTTAAAAATAACAAGTACTTTTTA  224
            ||||||||||||||||||||||||||||||||||||||||||||||||||||||||||||
Sbjct  120  TTTTTTTATTTAGTACTATTTGCCTTACATAGTTTCTTTAAAAATAACAAGTACTTTTTA  179

Query  225  TAGTTTGGTTCGATTTGCGTGttttttctttgtattcatttatattataggtttctatat  284
            ||||||||||||||||||||||||||||||||||||||||||||||||||||||||||||
Sbjct  180  TAGTTTGGTTCGATTTGCGTGTTTTTTCTTTGTATTCATTTATATTATAGGTTTCTATAT  239

Query  285  catatatttttcccaatcttttatgaagttttatttACAATTTAATTTCAATCGAAAATA  344
            ||| ||||||||||||||||||||||||||||||||||||||||||||||||||||||||
Sbjct  240  CATTTATTTTTCCCAATCTTTTATGAAGTTTTATTTACAATTTAATTTCAATCGAAAATA  299

Query  345  GAGAAAAATGACAATTTTGCTTTTTGATTTATATTGATTAAATCAAAATAAGAAAGAATA  404
            ||||||||||||||||||||||||||||||||||||||||||||||||||||||||||||
Sbjct  300  GAGAAAAATGACAATTTTGCTTTTTGATTTATATTGATTAAATCAAAATAAGAAAGAATA  359

Query  405  TGCtttttttATGTTAATGG-AAAAATATAGTAATACTAGATAATCGT  451
            |||||||||||||||||||| |||||||||||||||||||||||||||
Sbjct  360  TGCTTTTTTTATGTTAATGGAAAAAATATAGTAATACTAGATAATCGT  407
```

```
>NC_053296.1 Aldama canescens voucher Schilling 17 chloroplast, complete genome
 MN337908.1 Aldama canescens chloroplast, complete genome
Length=151386

 Score = 728 bits (394),  Expect = 0.0
 Identities = 486/527 (92%), Gaps = 19/527 (4%)
 Strand=Plus/Minus

Query  2    GTTATGCATGAACGTAATGCTCATAATTTCCCTCTAGACTTAGCTGCTATTGAAGCTCCA  61
            ||||||||||||||||||||||||||||||||||||||||||||||||||||||||||||
Sbjct  546  GTTATGCATGAACGTAATGCTCATAATTTCCCTCTAGACTTAGCTGCTATTGAAGCTCCA  487

Query  62   TATACAAATGGATAAGCCTTTGGTCTGATTGTATAGGAGTTTTTGAACTAAAAAAGGAGC  121
            | |||||||||||||| |||||||||||||||||||||||||||||||||||||||||||
Sbjct  486  TCTACAAATGGATAAGACTTTGGTCTGATTGTATAGGAGTTTTTGAACTAAAAAAGGAGC  427

Query  122  AATAACGCCCTCTTGATAAAACAAGAGGGAAGCTATTGCTCCttttttttATTTAGTACT  181
            ||||||||||||||| |  | |||||||||||||||||||||||||||||||||||||||
Sbjct  426  AATAACGCCCTCTTGTTTTATCAAGAGGGAAGCTATTGCTCCTTTTTTTTATTTAGTACT  367

Query  182  ATTTGCCTTACATAGTTTCTTTAAA-------AATAACAAGTACTTTTTATAGTTTGGTT  234
            |||||||||||||||||||||||||       ||||||||||||||||||||||||||||
Sbjct  366  ATTTGCCTTACATAGTTTCTTTAAACTTTAAAAATAACAAGTACTTTTTATAGTTTGGTT  307

Query  235  CGATTTGCGTGtttttctttgtattcatttatattataggtttctatatcatatatttt  294
            |||||||  |||||||| |||||||||||||||||||||||||||||||||||||| |||
Sbjct  306  CGATTTGTTTGTTTTTTATTTGTATTCATTTATATTATAGGTTTCTATATCATATACTTT  247

Query  295  t-c----cc-aatcttttatgaagttttattt--ACAATTTAATTTCAATCGAAAATAGA  346
            | |    || |||||||||||||||||| |||   ||||| ||| |||||||||||||||
Sbjct  246  TACTTTTCCTAATCTTTTATGAAGTTTTTTTTTACAATCTAAATTCAATCGAAAATAGA  187

Query  347  GAAAAATGACAATTTTGCTTTTTGATT--TA-TATTGATTAAATCAAAATAAGAAAGAAT  403
             ||||||||||||||||||| || |||  |  |||| ||| ||| ||||| |||||||||
Sbjct  186  TAAAAATGACAATTTTGCTTATTTATTACTTTATTTATTCAATAAAAATGAGAAAGAAT  127

Query  404  ATGCtttttttATGTTAATGGAAAAATATAGTAATACTAGATAATCGTAGAGGGGCGGAT  463
            |||||||||| |||||||||||||||||||||||||||||||||| ||||||||||||||
Sbjct  126  ATGCTTTTTT-ATGTTAATGGAAAAATATAGTAATACTAGATAATAGTAGAGGGGCGGAT  68

Query  464  GTAGCCAAGTGGATCAAGGCAGTGGATTGTGAATCCACCATGCGCGG  510
            |||||||||||||||||||||||||||||||||||||||||||||||
Sbjct  67   GTAGCCAAGTGGATCAAGGCAGTGGATTGTGAATCCACCATGCGCGG  21


>NC_057174.1 Aldama robusta voucher Bringel 985 chloroplast, complete genome
 MN356028.1 Aldama robusta chloroplast, complete genome
Length=151343

 Score = 723 bits (391),  Expect = 0.0
 Identities = 484/526 (92%), Gaps = 18/526 (3%)
 Strand=Plus/Minus

Query  2    GTTATGCATGAACGTAATGCTCATAATTTCCCTCTAGACTTAGCTGCTATTGAAGCTCCA  61
            ||||||||||||||||||||||||||||||||||||||||||||||||||||||||||||
Sbjct  554  GTTATGCATGAACGTAATGCTCATAATTTCCCTCTAGACTTAGCTGCTATTGAAGCTCCA  495
```

```
Query  62   TATACAAATGGATAAGCCTTTGGTCTGATTGTATAGGAGTTTTTGAACTAAAAAAGGAGC  121
            | |||||||||||||| |||||||||||||||||||||||||||||||||||||||||||
Sbjct  494  TCTACAAATGGATAAGACTTTGGTCTGATTGTATAGGAGTTTTTGAACTAAAAAAGGAGC  435

Query  122  AATAACGCCCTCTTGATAAAACAAGAGGGAAGCTATTGCTCCttttttttATTTAGTACT  181
            ||||||||||||||| |  | |||||||||||||||||||||||||||||||||||||||
Sbjct  434  AATAACGCCCTCTTGTTTTATCAAGAGGGAAGCTATTGCTCCTTTTTTTTATTTAGTACT  375

Query  182  ATTTGCCTTACATAGTTTCTTTAAA-------AATAACAAGTACTTTTTATAGTTTGGTT  234
            |||||||||||||||||||||||||       ||||||||||||||||||||||||||||
Sbjct  374  ATTTGCCTTACATAGTTTCTTTAAACTTTAAAAATAACAAGTACTTTTTATAGTTTGGTT  315

Query  235  CGATTTGCGTGtttttctttgtattcatttatattataggtttctatatcatatatttt  294
            |||||||  ||||||| ||||||||||||||||||||||||||||||||||||||| |||
Sbjct  314  CGATTTGTTTGTTTTTATTTGTATTCATTTATATTATAGGTTTCTATATCATATACTTT  255

Query  295  t-c----cc-aatcttttatgaagttttattt-ACAATTTAATTTCAATCGAAAATAGAG  347
            | |    || |||||||||||||||||| ||| ||||| ||| |||||||||||||||| 
Sbjct  254  TACTTTTCCTAATCTTTTATGAAGTTTTTTTTTACAATCTAAATTCAATCGAAAATAGAT  195

Query  348  AAAAATGACAATTTTGCTTTTTGATT--TA-TATTGATTAAATCAAAATAAGAAAGAATA  404
            |||||||||||||||| || || |||   |  |||| ||| ||| ||||| ||||||||||
Sbjct  194  AAAAATGACAATTTTGTTTATTTATTACTTTTATTTATTCAATAAAAATGAGAAAGAATA  135

Query  405  TGCtttttttATGTTAATGGAAAAATATAGTAATACTAGATAATCGTAGAGGGGCGGATG  464
            ||||||||| |||||||||| ||||||||||||||||||||||| |||||||||||||||
Sbjct  134  TGCTTTTTT-ATGTTAATGGGAAAATATAGTAATACTAGATAATAGTAGAGGGGCGGATG  76

Query  465  TAGCCAAGTGGATCAAGGCAGTGGATTGTGAATCCACCATGCGCGG  510
            ||||||||||||||||||||||||||||||||||||||||||||||
Sbjct  75   TAGCCAAGTGGATCAAGGCAGTGGATTGTGAATCCACCATGCGCGG  30


>NC_053279.1 Aldama filifolia voucher Loeuille 849 chloroplast, complete genome
 MN337890.1 Aldama filifolia chloroplast, complete genome
Length=151333

 Score = 723 bits (391),  Expect = 0.0
 Identities = 484/526 (92%), Gaps = 18/526 (3%)
 Strand=Plus/Minus

Query  2    GTTATGCATGAACGTAATGCTCATAATTTCCCTCTAGACTTAGCTGCTATTGAAGCTCCA  61
            ||||||||||||||||||||||||||||||||||||||||||||||||||||||||||||
Sbjct  554  GTTATGCATGAACGTAATGCTCATAATTTCCCTCTAGACTTAGCTGCTATTGAAGCTCCA  495

Query  62   TATACAAATGGATAAGCCTTTGGTCTGATTGTATAGGAGTTTTTGAACTAAAAAAGGAGC  121
            | |||||||||||||| |||||||||||||||||||||||||||||||||||||||||||
Sbjct  494  TCTACAAATGGATAAGACTTTGGTCTGATTGTATAGGAGTTTTTGAACTAAAAAAGGAGC  435

Query  122  AATAACGCCCTCTTGATAAAACAAGAGGGAAGCTATTGCTCCttttttttATTTAGTACT  181
            ||||||||||||||| |  | |||||||||||||||||||||||||||||||||||||||
Sbjct  434  AATAACGCCCTCTTGTTTTATCAAGAGGGAAGCTATTGCTCCTTTTTTTTATTTAGTACT  375

Query  182  ATTTGCCTTACATAGTTTCTTTAAA-------AATAACAAGTACTTTTTATAGTTTGGTT  234
            |||||||||||||||||||||||||       ||||||||||||||||||||||||||||
Sbjct  374  ATTTGCCTTACATAGTTTCTTTAAACTTTAAAAATAACAAGTACTTTTTATAGTTTGGTT  315
```

```
Query  235  CGATTTGCGTGtttttctttgtattcatttatattataggtttctatatcatatatttt  294
            |||||||  |||||||| ||||||||||||||||||||||||||||||||||||||| |||
Sbjct  314  CGATTTGTTTGTTTTTTATTTGTATTCATTTATATTATAGGTTTCTATATCATATACTTT  255

Query  295  t-c----cc-aatcttttatgaagttttattt-ACAATTTAATTTCAATCGAAAATAGAG  347
            | |    || |||||||||||||||||| ||| ||||| ||| ||||||||||||||||
Sbjct  254  TACTTTTCCTAATCTTTTATGAAGTTTTTTTTTACAATCTAAATTCAATCGAAAATAGAT  195

Query  348  AAAAATGACAATTTTGCTTTTTGATT--TA-TATTGATTAAATCAAAATAAGAAAGAATA  404
            |||||||||||||||| || || |||  |  |||| ||| ||| ||||| ||||||||||
Sbjct  194  AAAAATGACAATTTTGTTTATTTATTACTTTATTTATTCAATAAAAATGAGAAAGAATA  135

Query  405  TGCttttttATGTTAATGGAAAAATATAGTAATACTAGATAATCGTAGAGGGGCGGATG  464
            ||||||||| |||||||||| |||||||||||||||||||||| ||||||||||||||||
Sbjct  134  TGCTTTTTT-ATGTTAATGGGAAAATATAGTAATACTAGATAATAGTAGAGGGGCGGATG  76

Query  465  TAGCCAAGTGGATCAAGGCAGTGGATTGTGAATCCACCATGCGCGG  510
            ||||||||||||||||||||||||||||||||||||||||||||||
Sbjct  75   TAGCCAAGTGGATCAAGGCAGTGGATTGTGAATCCACCATGCGCGG  30


>NC_053297.1 Aldama corumbensis voucher Loeuille 909 chloroplast, complete
genome
 MN337909.1 Aldama corumbensis chloroplast, complete genome
Length=151327

 Score = 723 bits (391),  Expect = 0.0
 Identities = 484/526 (92%), Gaps = 18/526 (3%)
 Strand=Plus/Minus

Query  2    GTTATGCATGAACGTAATGCTCATAATTTCCCTCTAGACTTAGCTGCTATTGAAGCTCCA  61
            ||||||||||||||||||||||||||||||||||||||||||||||||||||||||||||
Sbjct  545  GTTATGCATGAACGTAATGCTCATAATTTCCCTCTAGACTTAGCTGCTATTGAAGCTCCA  486

Query  62   TATACAAATGGATAAGCCTTTGGTCTGATTGTATAGGAGTTTTTGAACTAAAAAAGGAGC  121
            | |||||||||||||| |||||||||||||||||||||||||||||||||||||||||||
Sbjct  485  TCTACAAATGGATAAGACTTTGGTCTGATTGTATAGGAGTTTTTGAACTAAAAAAGGAGC  426

Query  122  AATAACGCCCTCTTGATAAAACAAGAGGGAAGCTATTGCTCCttttttttATTTAGTACT  181
            ||||||||||||||| |  | |||||||||||||||||||||||||||||||||||||||
Sbjct  425  AATAACGCCCTCTTGTTTTATCAAGAGGGAAGCTATTGCTCCTTTTTTTTATTTAGTACT  366

Query  182  ATTTGCCTTACATAGTTTCTTTAAA-------AATAACAAGTACTTTTTATAGTTTGGTT  234
            |||||||||||||||||||||||||       ||||||||||||||||||||||||||||
Sbjct  365  ATTTGCCTTACATAGTTTCTTTAAACTTTAAAAATAACAAGTACTTTTTATAGTTTGGTT  306

Query  235  CGATTTGCGTGtttttctttgtattcatttatattataggtttctatatcatatatttt  294
            |||||||  |||||||| ||||||||||||||||||||||||||||||||||||||| |||
Sbjct  305  CGATTTGTTTGTTTTTTATTTGTATTCATTTATATTATAGGTTTCTATATCATATACTTT  246

Query  295  t-c----cc-aatcttttatgaagttttattt-ACAATTTAATTTCAATCGAAAATAGAG  347
            | |    || |||||||||||||||||| ||| ||||| ||| ||||||||||||||||
Sbjct  245  TACTTTTCCTAATCTTTTATGAAGTTTTTTTTTACAATCTAAATTCAATCGAAAATAGAT  186

Query  348  AAAAATGACAATTTTGCTTTTTGATT--TA-TATTGATTAAATCAAAATAAGAAAGAATA  404
            |||||||||||||||| || || |||  |  |||| ||| ||| ||||| ||||||||||
Sbjct  185  AAAAATGACAATTTTGTTTATTTATTACTTTATTTATTCAATAAAAATGAGAAAGAATA  126
```

```
Query  405   TGCtttttttATGTTAATGGAAAAATATAGTAATACTAGATAATCGTAGAGGGGCGGATG  464
             ||||||||| |||||||||| ||||||||||||||||||||||| |||||||||||||||
Sbjct  125   TGCTTTTTT-ATGTTAATGGGAAAATATAGTAATACTAGATAATAGTAGAGGGGCGGATG  67

Query  465   TAGCCAAGTGGATCAAGGCAGTGGATTGTGAATCCACCATGCGCGG  510
             ||||||||||||||||||||||||||||||||||||||||||||||
Sbjct  66    TAGCCAAGTGGATCAAGGCAGTGGATTGTGAATCCACCATGCGCGG  21
```

>NC_057172.1 Aldama excelsa voucher Schilling H2481 chloroplast, complete genome
MN356026.1 Aldama excelsa chloroplast, complete genome
Length=151312

Score = 721 bits (390), Expect = 0.0
Identities = 487/530 (92%), Gaps = 22/530 (4%)
Strand=Plus/Minus

```
Query  2     GTTATGCATGAACGTAATGCTCATAATTTCCCTCTAGACTTAGCTGCTATTGAAGCTCCA  61
             ||||||||||||||||||||||||||||||||||||||||||||||||||||||||||||
Sbjct  558   GTTATGCATGAACGTAATGCTCATAATTTCCCTCTAGACTTAGCTGCTATTGAAGCTCCA  499

Query  62    TATACAAATGGATAAGCCTTTGGTCTGATTGTATAGGAGTTTTTGAACTAAAAAAGGAGC  121
             | |||||||||||||| |||||||||||||||||||||||||||||||||||||||||||
Sbjct  498   TCTACAAATGGATAAGACTTTGGTCTGATTGTATAGGAGTTTTTGAACTAAAAAAGGAGC  439

Query  122   AATAACGCCCTCTTGATAAAACAAGAGGGAAGCTATTGCTCCtttttttt-ATTTAGTAC  180
             ||||||||||||||| |  | ||||||||||||||||||||||||||||| |||||||||
Sbjct  438   AATAACGCCCTCTTGTTTTATCAAGAGGGAAGCTATTGCTCCTTTTTTTTTATTTAGTAC  379

Query  181   TATTTGCCTTACATAGTTTCTTTAAA-------AATAACAAGTACTTTTTATAGTTTGGT  233
             ||||||||||||||||||||||||||       |||||||||||||||||||||||||||
Sbjct  378   TATTTGCCTTACATAGTTTCTTTAAACTTAAAAATAACAAGTACTTTTTATAGTTTGGT  319

Query  234   TCGATTTGCGTGttttttctttgtattcatttatattataggtttctatatcatatattt  293
             ||||||||  |||||||| |||||||||||||||||||||||||||||||||||||| ||
Sbjct  318   TCGATTTGTTTGTTTTTTATTTGTATTCATTTATATTATAGGTTTCTATATCATATACTT  259

Query  294   tt-c----cc-aatcttttatgaagttttatttACAATTTAATTTCAATCGAAAATAGAG  347
             || |    || |||||||||||||||||| |||||||||| | |||||||||||||||| 
Sbjct  258   TTACTTTTCCTAATCTTTTATGAAGTTTTTTTTACAATTTCAATTCAATCGAAAATAGAT  199

Query  348   AAAAATGACAATTTTGCTT-TTTG--A-TTT-A--TATTGATTAAATCAAAATAAGAAAG  400
             ||||||||||||||||||| |||   | ||| |  |||| ||| ||| ||||| ||||||
Sbjct  198   AAAAATGACAATTTTGCTTATTTATTACTTTTATTTATTTATTCAATAAAAATGAGAAAG  139

Query  401   AATATGCtttttttATGTTAATGGAAAAATATAGTAATACTAGATAATCGTAGAGGGGCG  460
             ||||||||||||| |||||||||||||||||||||||||||||||||| ||||| |||||
Sbjct  138   AATATGCTTTTTT-ATGTTAATGGAAAAATATAGTAATACTAGATAATAGTAGAAGGGCG  80

Query  461   GATGTAGCCAAGTGGATCAAGGCAGTGGATTGTGAATCCACCATGCGCGG  510
             ||||||||||||||||||||||||||||||||||||||||||||||||||
Sbjct  79    GATGTAGCCAAGTGGATCAAGGCAGTGGATTGTGAATCCACCATGCGCGG  30
```

>MT576958.1 Tithonia diversifolia chloroplast, complete genome

```
Length=151161

 Score = 721 bits (390),  Expect = 0.0
 Identities = 483/525 (92%), Gaps = 18/525 (3%)
 Strand=Plus/Minus

Query  2    GTTATGCATGAACGTAATGCTCATAATTTCCCTCTAGACTTAGCTGCTATTGAAGCTCCA  61
            ||||||||||||||||||||||||||||||||||||||||||||||||||||||||||||
Sbjct  543  GTTATGCATGAACGTAATGCTCATAATTTCCCTCTAGACTTAGCTGCTATTGAAGCTCCA  484

Query  62   TATACAAATGGATAAGCCTTTGGTCTGATTGTATAGGAGTTTTTGAACTAAAAAAGGAGC  121
            | |||||||||||||| |||||||||||||||||||||||||||||||||||||||||||
Sbjct  483  TCTACAAATGGATAAGACTTTGGTCTGATTGTATAGGAGTTTTTGAACTAAAAAAGGAGC  424

Query  122  AATAACGCCCTCTTGATAAAACAAGAGGGAAGCTATTGCTCCttttttttATTTAGTACT  181
            ||||||||||||||| |  | |||||||||||||||||||||||||||||||||||||||
Sbjct  423  AATAACGCCCTCTTGTTTTATCAAGAGGGAAGCTATTGCTCCTTTTTTTTATTTAGTACT  364

Query  182  ATTTGCCTTACATAGTTTCTTTAAA-------AATAACAAGTACTTTTTATAGTTTGGTT  234
            |||||||||||||||||||||||||       ||||||||||||||||| ||||||||||
Sbjct  363  ATTTGCCTTACATAGTTTCTTTAAACTTTAAAAATAACAAGTACTTTTTCTAGTTTGGTT  304

Query  235  CGATTTGCGTGttttttctttgtattcatttatattataggtttctatatcatatatttt  294
            |||||||  |||||||| |||||||||||||||||||||||||||||||||||||| |||
Sbjct  303  CGATTTGTTTGTTTTTTATTTGTATTCATTTATATTATAGGTTTCTATATCATATACTTT  244

Query  295  t-c----cc-aatcttttatgaagttttatttACAATTTAATTTCAATCGAAAATAGAGA  348
            | |    || |||||||||||||||||| |||||||||| | ||||||||||| |||| |
Sbjct  243  TACTTTTCCTAATCTTTTATGAAGTTTT-TTTACAATTTCAATTCAATCGAAACTAGATA  185

Query  349  AAAATGACAATTTTGCTTTTTGATT--TA-TATTGATTAAATCAAAATAAGAAAGAATAT  405
            ||||||||||||||| || || |||  |  |||| ||| ||| ||||| |||||||||||
Sbjct  184  AAAATGACAATTTTGTTTATTTATTACTTTTATTTATTCAATAAAAATGAGAAAGAATAT  125

Query  406  GCtttttttATGTTAATGGAAAAATATAGTAATACTAGATAATCGTAGAGGGGCGGATGT  465
            |||||||| |||||||||||||||||||||||||||||||||| ||||||||||||||||
Sbjct  124  GCTTTTTT-ATGTTAATGGAAAAATATAGTAATACTAGATAATAGTAGAGGGGCGGATGT  66

Query  466  AGCCAAGTGGATCAAGGCAGTGGATTGTGAATCCACCATGCGCGG  510
            |||||||||||||||||||||||||||||||||||||||||||||
Sbjct  65   AGCCAAGTGGATCAAGGCAGTGGATTGTGAATCCACCATGCGCGG  21


>NC_053300.1 Tithonia diversifolia voucher Loeuille 678 chloroplast, complete
genome
 MT700544.1 Tithonia diversifolia chloroplast, complete genome
Length=151356

 Score = 721 bits (390),  Expect = 0.0
 Identities = 483/525 (92%), Gaps = 18/525 (3%)
 Strand=Plus/Minus

Query  2    GTTATGCATGAACGTAATGCTCATAATTTCCCTCTAGACTTAGCTGCTATTGAAGCTCCA  61
            ||||||||||||||||||||||||||||||||||||||||||||||||||||||||||||
Sbjct  543  GTTATGCATGAACGTAATGCTCATAATTTCCCTCTAGACTTAGCTGCTATTGAAGCTCCA  484

Query  62   TATACAAATGGATAAGCCTTTGGTCTGATTGTATAGGAGTTTTTGAACTAAAAAAGGAGC  121
```

```
            | |||||||||||||| |||||||||||||||||||||||||||||||||||||||||||
Sbjct  483  TCTACAAATGGATAAGACTTTGGTCTGATTGTATAGGAGTTTTTGAACTAAAAAAGGAGC  424

Query  122  AATAACGCCCTCTTGATAAAACAAGAGGGAAGCTATTGCTCCttttttttATTTAGTACT  181
            ||||||||||||||| |  | |||||||||||||||||||||||||||||||||||||||
Sbjct  423  AATAACGCCCTCTTGTTTTATCAAGAGGGAAGCTATTGCTCCTTTTTTTTATTTAGTACT  364

Query  182  ATTTGCCTTACATAGTTTCTTTAAA-------AATAACAAGTACTTTTTATAGTTTGGTT  234
            |||||||||||||||||||||||||       ||||||||||||||||| ||||||||||
Sbjct  363  ATTTGCCTTACATAGTTTCTTTAAACTTTAAAAATAACAAGTACTTTTTCTAGTTTGGTT  304

Query  235  CGATTTGCGTGttttttctttgtattcatttatattataggtttctatatcatatatttt  294
            |||||||  |||||||| |||||||||||||||||||||||||||||||||||||| |||
Sbjct  303  CGATTTGTTTGTTTTTTATTTGTATTCATTTATATTATAGGTTTCTATATCATATACTTT  244

Query  295  t-c----cc-aatcttttatgaagttttatttACAATTTAATTTCAATCGAAAATAGAGA  348
            | |    || |||||||||||||||||| |||||||||| | ||||||||||| |||| |
Sbjct  243  TACTTTTCCTAATCTTTTATGAAGTTTT-TTTACAATTTCAATTCAATCGAAACTAGATA  185

Query  349  AAAATGACAATTTTGCTTTTTGATT--TA-TATTGATTAAATCAAAATAAGAAAGAATAT  405
            ||||||||||||||| || || |||  |  |||| ||| ||| ||||| |||||||||||
Sbjct  184  AAAATGACAATTTTGTTTATTTATTACTTTTATTTATTCAATAAAAATGAGAAAGAATAT  125

Query  406  GCtttttttATGTTAATGGAAAAATATAGTAATACTAGATAATCGTAGAGGGGCGGATGT  465
            |||||||| |||||||||||||||||||||||||||||||||| ||||||||||||||||
Sbjct  124  GCTTTTTT-ATGTTAATGGAAAAATATAGTAATACTAGATAATAGTAGAGGGGCGGATGT  66

Query  466  AGCCAAGTGGATCAAGGCAGTGGATTGTGAATCCACCATGCGCGG  510
            |||||||||||||||||||||||||||||||||||||||||||||
Sbjct  65   AGCCAAGTGGATCAAGGCAGTGGATTGTGAATCCACCATGCGCGG  21


>MT700543.1 Pappobolus lanatus var. lanatus chloroplast, complete genome
Length=151358

 Score = 721 bits (390),  Expect = 0.0
 Identities = 483/525 (92%), Gaps = 17/525 (3%)
 Strand=Plus/Minus

Query  2    GTTATGCATGAACGTAATGCTCATAATTTCCCTCTAGACTTAGCTGCTATTGAAGCTCCA  61
            ||||||||||||||||||||||||||||||||||||||||||||||||||||||||||||
Sbjct  544  GTTATGCATGAACGTAATGCTCATAATTTCCCTCTAGACTTAGCTGCTATTGAAGCTCCA  485

Query  62   TATACAAATGGATAAGCCTTTGGTCTGATTGTATAGGAGTTTTTGAACTAAAAAAGGAGC  121
            | |||||||||||||| |||||||||||||||||||||||||||||||||||||||||||
Sbjct  484  TCTACAAATGGATAAGACTTTGGTCTGATTGTATAGGAGTTTTTGAACTAAAAAAGGAGC  425

Query  122  AATAACGCCCTCTTGATAAAACAAGAGGGAAGCTATTGCTCCttttttttATTTAGTACT  181
            ||||||| ||||||| |  | |||||||||||||||||||||||||||||||||||||||
Sbjct  424  AATAACGTCCTCTTGTTTTATCAAGAGGGAAGCTATTGCTCCTTTTTTTTATTTAGTACT  365

Query  182  ATTTGCCTTACATAGTTTCTTTAAA-------AATAACAAGTACTTTTTATAGTTTGGTT  234
            |||||||||||||||||||||||||       ||||||||||||||||||||||||||||
Sbjct  364  ATTTGCCTTACATAGTTTCTTTAAACTTTAAAAATAACAAGTACTTTTTATAGTTTGGTT  305

Query  235  CGATTTGCGTGttttttctttgtattcatttatattataggt------ttctatatcata  288
            |||||||  |||||||| ||||||||||||||||||||||||      ||||||||||||
```

```
Sbjct  304  CGATTTGTTTGTTTTTTATTTGTATTCATTTATATTATAGGTATAGGTTTCTATATCATA  245

Query  289  tatttttcccaatcttttatgaagttttatttACAATTTAATTTCAATCGAAAATAGAGA  348
            || |||||| |||||||||||||||||| |||||||||||| |||||||||||||||| |
Sbjct  244  TACTTTTCCTAATCTTTTATGAAGTTTTTTTTACAATTTAAATTCAATCGAAAATAGATA  185

Query  349  AAAATGACAATTTTGCTTTTTGATT--TA-TATTGATTAAATCAAAATAAGAAAGAATAT  405
            ||||||||||||||| || || |||  |  |||| ||| ||| ||||| |||||||||||
Sbjct  184  AAAATGACAATTTTGTTTATTTATTACTTTTATTTATTCAATAAAAATGAGAAAGAATAT  125

Query  406  GCtttttttATGTTAATGGAAAAATATAGTAATACTAGATAATCGTAGAGGGGCGGATGT  465
            |||||||| |||||||||||||||||||||||||||| ||||| |||||||||||||||
Sbjct  124  GCTTTTTT-ATGTTAATGGAAAAATATAGTAATACTATATAATAGTAGAGGGGCGGATGT  66

Query  466  AGCCAAGTGGATCAAGGCAGTGGATTGTGAATCCACCATGCGCGG  510
            |||||||||||||||||||||||||||||||||||||||||||||
Sbjct  65   AGCCAAGTGGATCAAGGCAGTGGATTGTGAATCCACCATGCGCGG  21
```

>NC_053284.1 Aldama kunthiana voucher voucher Silva s.n. (ESA 122873) chloroplast, complete genome
 MN337895.1 Aldama kunthiana chloroplast, complete genome
Length=151327

 Score = 717 bits (388), Expect = 0.0
 Identities = 484/527 (92%), Gaps = 19/527 (4%)
 Strand=Plus/Minus

```
Query  2    GTTATGCATGAACGTAATGCTCATAATTTCCCTCTAGACTTAGCTGCTATTGAAGCTCCA  61
            ||||||||||||||||||||||||||||||||||||||||||||||||||||||||||||
Sbjct  548  GTTATGCATGAACGTAATGCTCATAATTTCCCTCTAGACTTAGCTGCTATTGAAGCTCCA  489

Query  62   TATACAAATGGATAAGCCTTTGGTCTGATTGTATAGGAGTTTTTGAACTAAAAAAGGAGC  121
            | |||||||||||||| |||||||||||||||||||||||||||||||||||||||||||
Sbjct  488  TCTACAAATGGATAAGACTTTGGTCTGATTGTATAGGAGTTTTTGAACTAAAAAAGGAGC  429

Query  122  AATAACGCCCTCTTGATAAAACAAGAGGGAAGCTATTGCTCCttttttttATTTAGTACT  181
            ||||||||||||||| |  | |||||||||||||||||||||||||||||||||||||||
Sbjct  428  AATAACGCCCTCTTGTTTTATCAAGAGGGAAGCTATTGCTCCTTTTTTTTATTTAGTACT  369

Query  182  ATTTGCCTTACATAGTTTCTTTAAA-------AATAACAAGTACTTTTTATAGTTTGGTT  234
            ||||||||||||||||||||||||       |||||||||||||||||||||||||||
Sbjct  368  ATTTGCCTTACATAGTTTCTTTAAACTTTAAAAATAACAAGTACTTTTTATAGTTTGGTT  309

Query  235  CGATTTGCGTGtttttctttgtattcatttatattataggtttctatatcatatatttt  294
            |||||||  |||||||| ||||||||||||||||||||||||||||||||||||||| |||
Sbjct  308  CGATTTGTTTGTTTTTTATTTGTATTCATTTATATTATAGGTTTCTATATCATATACTTT  249

Query  295  t-c----cc-aatcttttatgaagttttattt--ACAATTTAATTTCAATCGAAAATAGA  346
            | |    || |||||||||||||||||| |||  ||||| ||| ||||||||||||||||
Sbjct  248  TACTTTTCCTAATCTTTTATGAAGTTTTTTTTTTACAATCTAAATTCAATCGAAAATAGA  189

Query  347  GAAAAATGACAATTTTGCTTTTTGATT--TA-TATTGATTAAATCAAAATAAGAAAGAAT  403
             |||||||||||||||| || || |||  |  |||| ||| ||| ||||| |||||||||
Sbjct  188  TAAAAATGACAATTTTGTTTATTTATTACTTTTATTTATTCAATAAAAATGAGAAAGAAT  129

Query  404  ATGCttttttttATGTTAATGGAAAAATATAGTAATACTAGATAATCGTAGAGGGGCGGAT  463
```

```
             |||||||||| |||||||||| |||||||||||||||||||||| ||||||||||||||
Sbjct  128   ATGCTTTTTT-ATGTTAATGGGAAAATATAGTAATACTAGATAATAGTAGAGGGGCGGAT  70

Query  464   GTAGCCAAGTGGATCAAGGCAGTGGATTGTGAATCCACCATGCGCGG  510
             |||||||||||||||||||||||||||||||||||||||||||||||
Sbjct  69    GTAGCCAAGTGGATCAAGGCAGTGGATTGTGAATCCACCATGCGCGG  23


>NC_057177.1 Aldama squalida voucher Loeuille 790 chloroplast, complete genome
 MN356031.1 Aldama squalida chloroplast, complete genome
Length=151399

 Score = 717 bits (388),  Expect = 0.0
 Identities = 484/527 (92%), Gaps = 19/527 (4%)
 Strand=Plus/Minus

Query  2     GTTATGCATGAACGTAATGCTCATAATTTCCCTCTAGACTTAGCTGCTATTGAAGCTCCA  61
             ||||||||||||||||||||||||||||||||||||||||||||||||||||||||||||
Sbjct  555   GTTATGCATGAACGTAATGCTCATAATTTCCCTCTAGACTTAGCTGCTATTGAAGCTCCA  496

Query  62    TATACAAATGGATAAGCCTTTGGTCTGATTGTATAGGAGTTTTTGAACTAAAAAAGGAGC  121
             | |||||||||||||| |||||||||||||||||||||||||||||||||||||||||||
Sbjct  495   TCTACAAATGGATAAGACTTTGGTCTGATTGTATAGGAGTTTTTGAACTAAAAAAGGAGC  436

Query  122   AATAACGCCCTCTTGATAAAACAAGAGGGAAGCTATTGCTCCtttttttt-ATTTAGTAC  180
             ||||||||||||||| |  | ||||||||||||||||||||||||||||| |||||||||
Sbjct  435   AATAACGCCCTCTTGTTTTATCAAGAGGGAAGCTATTGCTCCTTTTTTTTTATTTAGTAC  376

Query  181   TATTTGCCTTACATAGTTCTTTAAA-------AATAACAAGTACTTTTTATAGTTTGGT  233
             ||||||||||||||||||||||||||       ||||||||||||||||||||||||||
Sbjct  375   TATTTGCCTTACATAGTTCTTTAAACTTTAAAAATAACAAGTACTTTTTATAGTTTGGT  316

Query  234   TCGATTTGCGTGtttttctttgtattcatttatattataggtttctatatcatata---  290
             ||||||||  |||||||| ||||||||||||||||||||||||||||||||||||||
Sbjct  315   TCGATTTGTTTGTTTTTATTTGTATTCATTTATATTATAGGTTTCTATATCATATACTT  256

Query  291   -t--ttttcccaatcttttatgaagtttt-atttACAATTTAATTTCAATCGAAAATAGA  346
              |  |||||| ||||||||||||||||||  |||||||| ||| ||||||||||||||||
Sbjct  255   TTACTTTTCCTAATCTTTTATGAAGTTTTTTTTTACAATCTAAATTCAATCGAAAATAGA  196

Query  347   GAAAAATGACAATTTTGCTTTTTGA-T--TTATATTGATTAAATCAAAATAAGAAAGAAT  403
              ||||||||||||||| || || | |  || |||| ||| ||| ||||| |||||||||
Sbjct  195   TAAAAATGACAATTTTGTTTATTTATTACTTTTATTTATTCAATAAAAATGAGAAAGAAT  136

Query  404   ATGCttttttttATGTTAATGGAAAAATATAGTAATACTAGATAATCGTAGAGGGGCGGAT  463
             |||||||||| |||||||||| |||||||||||||||||||||| ||||||||||||||
Sbjct  135   ATGCTTTTTT-ATGTTAATGGGAAAATATAGTAATACTAGATAATAGTAGAGGGGCGGAT  77

Query  464   GTAGCCAAGTGGATCAAGGCAGTGGATTGTGAATCCACCATGCGCGG  510
             |||||||||||||||||||||||||||||||||||||||||||||||
Sbjct  76    GTAGCCAAGTGGATCAAGGCAGTGGATTGTGAATCCACCATGCGCGG  30


>NC_053295.1 Aldama bracteata voucher Filartiga 15 chloroplast, complete genome
 MN337907.1 Aldama bracteata chloroplast, complete genome
Length=151339
```

```
 Score = 717 bits (388),  Expect = 0.0
 Identities = 484/527 (92%), Gaps = 19/527 (4%)
 Strand=Plus/Minus

Query  2    GTTATGCATGAACGTAATGCTCATAATTTCCCTCTAGACTTAGCTGCTATTGAAGCTCCA  61
            ||||||||||||||||||||||||||||||||||||||||||||||||||||||||||||
Sbjct  546  GTTATGCATGAACGTAATGCTCATAATTTCCCTCTAGACTTAGCTGCTATTGAAGCTCCA  487

Query  62   TATACAAATGGATAAGCCTTTGGTCTGATTGTATAGGAGTTTTTGAACTAAAAAAGGAGC  121
            | |||||||||||||| |||||||||||||||||||||||||||||||||||||||||||
Sbjct  486  TCTACAAATGGATAAGACTTTGGTCTGATTGTATAGGAGTTTTTGAACTAAAAAAGGAGC  427

Query  122  AATAACGCCCTCTTGATAAAACAAGAGGGAAGCTATTGCTCCtttttttATTTAGTACT  181
            ||||||||||||||| |  | |||||||||||||||||||||||||||||||||||||||
Sbjct  426  AATAACGCCCTCTTGTTTTATCAAGAGGGAAGCTATTGCTCCTTTTTTTTATTTAGTACT  367

Query  182  ATTTGCCTTACATAGTTTCTTTAAA-------AATAACAAGTACTTTTTATAGTTTGGTT  234
            |||||||||||||||||||||||||       ||||||||||||||||||||||||||||
Sbjct  366  ATTTGCCTTACATAGTTTCTTTAAACTTTAAAAATAACAAGTACTTTTTATAGTTTGGTT  307

Query  235  CGATTTGCGTGttttttctttgtattcatttatattataggtttctatatcatatatttt  294
            |||||||  |||||||| |||||||||||||||||||||||||||||||||||||| |||
Sbjct  306  CGATTTGTTTGTTTTTTATTTGTATTCATTTATATTATAGGTTTCTATATCATATACTTT  247

Query  295  t-c----cc-aatcttttatgaagttttattt--ACAATTTAATTTCAATCGAAAATAGA  346
            | |    || |||||||||||||||||| |||  ||||| ||| ||||||||||||||||
Sbjct  246  TACTTTTCCTAATCTTTTATGAAGTTTTTTTTTTACAATCTAAATTCAATCGAAAATAGA  187

Query  347  GAAAAATGACAATTTTGCTTTTTGATT--TA-TATTGATTAAATCAAAATAAGAAAGAAT  403
             |||||||||||||||| || || |||  |   |||| ||| ||| ||||| |||||||||
Sbjct  186  TAAAAATGACAATTTTGTTTATTTATTACTTTTATTTATTCAATAAAAATGAGAAAGAAT  127

Query  404  ATGCtttttttATGTTAATGGAAAAATATAGTAATACTAGATAATCGTAGAGGGGCGGAT  463
            |||||||||| |||||||||| ||||||||||||||||||||||| ||||||||||||||
Sbjct  126  ATGCTTTTTT-ATGTTAATGGGAAAATATAGTAATACTAGATAATAGTAGAGGGGCGGAT  68

Query  464  GTAGCCAAGTGGATCAAGGCAGTGGATTGTGAATCCACCATGCGCGG  510
            |||||||||||||||||||||||||||||||||||||||||||||||
Sbjct  67   GTAGCCAAGTGGATCAAGGCAGTGGATTGTGAATCCACCATGCGCGG  21


>NC_057171.1 Aldama discolor voucher Bombo 72 chloroplast, complete genome
 MN356025.1 Aldama discolor chloroplast, complete genome
Length=151360

 Score = 717 bits (388),  Expect = 0.0
 Identities = 484/527 (92%), Gaps = 19/527 (4%)
 Strand=Plus/Minus

Query  2    GTTATGCATGAACGTAATGCTCATAATTTCCCTCTAGACTTAGCTGCTATTGAAGCTCCA  61
            ||||||||||||||||||||||||||||||||||||||||||||||||||||||||||||
Sbjct  548  GTTATGCATGAACGTAATGCTCATAATTTCCCTCTAGACTTAGCTGCTATTGAAGCTCCA  489

Query  62   TATACAAATGGATAAGCCTTTGGTCTGATTGTATAGGAGTTTTTGAACTAAAAAAGGAGC  121
            | |||||||||||||| |||||||||||||||||||||||||||||||||||||||||||
Sbjct  488  TCTACAAATGGATAAGACTTTGGTCTGATTGTATAGGAGTTTTTGAACTAAAAAAGGAGC  429
```

```
Query  122  AATAACGCCCTCTTGATAAAACAAGAGGGAAGCTATTGCTCCtttttttt-ATTTAGTAC  180
            ||||||||||||||| |  | ||||||||||||||||||||||||||||| |||||||||
Sbjct  428  AATAACGCCCTCTTGTTTTATCAAGAGGGAAGCTATTGCTCCTTTTTTTTTATTTAGTAC  369

Query  181  TATTTGCCTTACATAGTTTCTTTAAA-------AATAACAAGTACTTTTTATAGTTTGGT  233
            ||||||||||||||||||||||||||       |||||||||||||||||||||||||||
Sbjct  368  TATTTGCCTTACATAGTTTCTTTAAACTTTAAAAATAACAAGTACTTTTTATAGTTTGGT  309

Query  234  TCGATTTGCGTGtttttctttgtattcatttatattataggtttctatatcatata---  290
            ||||||||  ||||||| |||||||||||||||||||||||||||||||||||||||   
Sbjct  308  TCGATTTGTTTGTTTTTATTTGTATTCATTTATATTATAGGTTTCTATATCATATACTT  249

Query  291  -t--ttttcccaatcttttatgaagtttt-atttACAATTTAATTTCAATCGAAAATAGA  346
             |  |||||| ||||||||||||||||||  |||||||| ||| ||||||||||||||||
Sbjct  248  TTACTTTTCCTAATCTTTTATGAAGTTTTTTTTTACAATCTAAATTCAATCGAAAATAGA  189

Query  347  GAAAAATGACAATTTTGCTTTTTGA-T--TTATATTGATTAAATCAAAATAAGAAAGAAT  403
             |||||||||||||||| || || | |  || |||| ||| ||| ||||| |||||||||
Sbjct  188  TAAAAATGACAATTTTGTTTATTTATTACTTTTATTTATTCAATAAAAATGAGAAAGAAT  129

Query  404  ATGCtttttttATGTTAATGGAAAAATATAGTAATACTAGATAATCGTAGAGGGGCGGAT  463
            |||||||||| |||||||||| ||||||||||||||||||||||| ||||||||||||||
Sbjct  128  ATGCTTTTTT-ATGTTAATGGGAAAATATAGTAATACTAGATAATAGTAGAGGGGCGGAT  70

Query  464  GTAGCCAAGTGGATCAAGGCAGTGGATTGTGAATCCACCATGCGCGG  510
            |||||||||||||||||||||||||||||||||||||||||||||||
Sbjct  69   GTAGCCAAGTGGATCAAGGCAGTGGATTGTGAATCCACCATGCGCGG  23
```

>MF037491.1 Echinacea atrorubens voucher Barnard1532 trnH-psbA intergenic spacer region, partial sequence; chloroplast
Length=406

Score = 717 bits (388), Expect = 0.0
Identities = 402/408 (99%), Gaps = 3/408 (1%)
Strand=Plus/Plus

```
Query  45   CTGCTATTGAAGCTCCATATACAAATGGATAAGCCTTTGGTCTGATTGTATAGGAGTTTT  104
            |||||||||||||||||| |||||||||||||||||||||||||||||||||||||||||
Sbjct  1    CTGCTATTGAAGCTCCATCTACAAATGGATAAGCCTTTGGTCTGATTGTATAGGAGTTTT  60

Query  105  TGAACTAAAAAAGGAGCAATAACGCCCTCTTGATAAAACAAGAGGGAAGCTATTGCTCCt  164
            |||||||||||||||||||||||| |||||||||||||||||||||||||||||||||| 
Sbjct  61   TGAACTAAAAAAGGAGCAATAACGTCCTCTTGATAAAACAAGAGGGAAGCTATTGCTCC-  119

Query  165  tttttttATTTAGTACTATTTGCCTTACATAGTTTCTTTAAAAATAACAAGTACTTTTTA  224
            ||||||||||||||||||||||||||||||||||||||||||||||||||||||||||||
Sbjct  120  TTTTTTTATTTAGTACTATTTGCCTTACATAGTTTCTTTAAAAATAACAAGTACTTTTTA  179

Query  225  TAGTTTGGTTCGATTTGCGTGtttttctttgtattcatttatattataggtttctatat  284
            ||||||||||||||||||||||||||||||||||||||||||||||||||||||||||||
Sbjct  180  TAGTTTGGTTCGATTTGCGTGTTTTTCTTTGTATTCATTTATATTATAGGTTTCTATAT  239

Query  285  catatatttttcccaatcttttatgaagttttatttACAATTTAATTTCAATCGAAAATA  344
            ||| ||||||||||||||||||||||||||||||||||||||||||||||||||||||||
Sbjct  240  CATTTATTTTTCCCAATCTTTTATGAAGTTTTATTTACAATTTAATTTCAATCGAAAATA  299
```

```
Query  345  GAGAAAAATGACAATTTTGCTTTTTGATTTATATTGATTAAATCAAAATAAGAAAGAATA  404
            ||||||||||||||||||||||||||||||||||||||||||||||||||||||||||||
Sbjct  300  GAGAAAAATGACAATTTTGCTTTTTGATTTATATTGATTAAATCAAAATAAGAAAGAATA  359

Query  405  TGCtttttttATGTTAATGG-AAAAATATAGTAATACTAGATAATCGT  451
            ||| |||||||||||||||| |||||||||||||||||||||||||||
Sbjct  360  TGC-TTTTTTATGTTAATGGAAAAAATATAGTAATACTAGATAATCGT  406


>NC_053282.1 Aldama goyazii voucher Magenta 716 chloroplast, complete genome
 MN337893.1 Aldama goyazii chloroplast, complete genome
Length=151279

 Score = 717 bits (388),  Expect = 0.0
 Identities = 484/527 (92%), Gaps = 19/527 (4%)
 Strand=Plus/Minus

Query  2    GTTATGCATGAACGTAATGCTCATAATTTCCCTCTAGACTTAGCTGCTATTGAAGCTCCA  61
            ||||||||||||||||||||||||||||||||||||||||||||||||||||||||||||
Sbjct  548  GTTATGCATGAACGTAATGCTCATAATTTCCCTCTAGACTTAGCTGCTATTGAAGCTCCA  489

Query  62   TATACAAATGGATAAGCCTTTGGTCTGATTGTATAGGAGTTTTTGAACTAAAAAAGGAGC  121
            | |||||||||||||| |||||||||||||||||||||||||||||||||||||||||||
Sbjct  488  TCTACAAATGGATAAGACTTTGGTCTGATTGTATAGGAGTTTTTGAACTAAAAAAGGAGC  429

Query  122  AATAACGCCCTCTTGATAAAACAAGAGGGAAGCTATTGCTCCttttttttATTTAGTACT  181
            ||||||||||||||| |  | |||||||||||||||||||||||||||||||||||||||
Sbjct  428  AATAACGCCCTCTTGTTTTATCAAGAGGGAAGCTATTGCTCCTTTTTTTTATTTAGTACT  369

Query  182  ATTTGCCTTACATAGTTTCTTTAAA-------AATAACAAGTACTTTTTATAGTTTGGTT  234
            |||||||||||||||||||||||||       ||||||||||||||||||||||||||||
Sbjct  368  ATTTGCCTTACATAGTTTCTTTAAACTTTAAAAATAACAAGTACTTTTTATAGTTTGGTT  309

Query  235  CGATTTGCGTGttttttctttgtattcatttatattataggtttctatatcatatatttt  294
            |||||||  |||||||| |||||||||||||||||||||||||||||||||||||| |||
Sbjct  308  CGATTTGTTTGTTTTTTATTTGTATTCATTTATATTATAGGTTTCTATATCATATACTTT  249

Query  295  t-c----cc-aatcttttatgaagttttattt--ACAATTTAATTTCAATCGAAAATAGA  346
            | |    || |||||||||||||||||| |||  ||||| ||| ||||||||||||||||
Sbjct  248  TACTTTTCCTAATCTTTTATGAAGTTTTTTTTTTACAATCTAAATTCAATCGAAAATAGA  189

Query  347  GAAAAATGACAATTTTGCTTTTTGATT--TA-TATTGATTAAATCAAAATAAGAAAGAAT  403
             |||||||||||||||| || || |||  |  |||| ||| ||| ||||| |||||||||
Sbjct  188  TAAAAATGACAATTTTGTTTATTTATTACTTTTATTTATTCAATAAAAATGAGAAAGAAT  129

Query  404  ATGCtttttttATGTTAATGGAAAAATATAGTAATACTAGATAATCGTAGAGGGGCGGAT  463
            |||||||||| |||||||||| ||||||||||||||||||||||| ||||||||||||||
Sbjct  128  ATGCTTTTTT-ATGTTAATGGGAAAATATAGTAATACTAGATAATAGTAGAGGGGCGGAT  70

Query  464  GTAGCCAAGTGGATCAAGGCAGTGGATTGTGAATCCACCATGCGCGG  510
            |||||||||||||||||||||||||||||||||||||||||||||||
Sbjct  69   GTAGCCAAGTGGATCAAGGCAGTGGATTGTGAATCCACCATGCGCGG  23


>NC_053294.1 Aldama bakeriana voucher Loeuille 867 chloroplast, complete genome
 MN337906.1 Aldama bakeriana chloroplast, complete genome
Length=151376
```

```
 Score = 717 bits (388),  Expect = 0.0
 Identities = 484/527 (92%), Gaps = 19/527 (4%)
 Strand=Plus/Minus

Query  2    GTTATGCATGAACGTAATGCTCATAATTTCCCTCTAGACTTAGCTGCTATTGAAGCTCCA  61
            ||||||||||||||||||||||||||||||||||||||||||||||||||||||||||||
Sbjct  546  GTTATGCATGAACGTAATGCTCATAATTTCCCTCTAGACTTAGCTGCTATTGAAGCTCCA  487

Query  62   TATACAAATGGATAAGCCTTTGGTCTGATTGTATAGGAGTTTTTGAACTAAAAAAGGAGC  121
            | |||||||||||||| |||||||||||||||||||||||||||||||||||||||||||
Sbjct  486  TCTACAAATGGATAAGACTTTGGTCTGATTGTATAGGAGTTTTTGAACTAAAAAAGGAGC  427

Query  122  AATAACGCCCTCTTGATAAAACAAGAGGGAAGCTATTGCTCCtttttttt-ATTTAGTAC  180
            ||||||||||||||| |  | ||||||||||||||||||||||||||||| |||||||||
Sbjct  426  AATAACGCCCTCTTGTTTTATCAAGAGGGAAGCTATTGCTCCTTTTTTTTTATTTAGTAC  367

Query  181  TATTTGCCTTACATAGTTTCTTTAAA-------AATAACAAGTACTTTTTATAGTTTGGT  233
            ||||||||||||||||||||||||||       |||||||||||||||||||||||||||
Sbjct  366  TATTTGCCTTACATAGTTTCTTTAAACTTTAAAAATAACAAGTACTTTTTATAGTTTGGT  307

Query  234  TCGATTTGCGTGttttttctttgtattcatttatattataggtttctatatcatata---  290
            ||||||||  |||||||| ||||||||||||||||||||||||||||||||||||||   
Sbjct  306  TCGATTTGTTTGTTTTTTATTTGTATTCATTTATATTATAGGTTTCTATATCATATACTT  247

Query  291  -t--ttttcccaatcttttatgaagtttt-atttACAATTTAATTTCAATCGAAAATAGA  346
             |  |||||| ||||||||||||||||||  |||||||| ||| ||||||||||||||||
Sbjct  246  TTACTTTTCCTAATCTTTTATGAAGTTTTTTTTTACAATCTAAATTCAATCGAAAATAGA  187

Query  347  GAAAAATGACAATTTTGCTTTTTGA-T--TTATATTGATTAAATCAAAATAAGAAAGAAT  403
             |||||||||||||||| || || | |  || |||| ||| ||| ||||| |||||||||
Sbjct  186  TAAAAATGACAATTTTGTTTATTTATTACTTTTATTTATTCAATAAAAATGAGAAAGAAT  127

Query  404  ATGCtttttttATGTTAATGGAAAAATATAGTAATACTAGATAATCGTAGAGGGGCGGAT  463
            |||||||||| |||||||||| ||||||||||||||||||||||| ||||||||||||||
Sbjct  126  ATGCTTTTTT-ATGTTAATGGGAAAATATAGTAATACTAGATAATAGTAGAGGGGCGGAT  68

Query  464  GTAGCCAAGTGGATCAAGGCAGTGGATTGTGAATCCACCATGCGCGG  510
            |||||||||||||||||||||||||||||||||||||||||||||||
Sbjct  67   GTAGCCAAGTGGATCAAGGCAGTGGATTGTGAATCCACCATGCGCGG  21


>NC_053286.1 Aldama macrorhiza voucher Magenta 476 chloroplast, complete genome
 MN337897.1 Aldama macrorhiza voucher Magenta 476 chloroplast, complete genome
Length=151314

 Score = 717 bits (388),  Expect = 0.0
 Identities = 484/527 (92%), Gaps = 19/527 (4%)
 Strand=Plus/Minus

Query  2    GTTATGCATGAACGTAATGCTCATAATTTCCCTCTAGACTTAGCTGCTATTGAAGCTCCA  61
            ||||||||||||||||||||||||||||||||||||||||||||||||||||||||||||
Sbjct  548  GTTATGCATGAACGTAATGCTCATAATTTCCCTCTAGACTTAGCTGCTATTGAAGCTCCA  489

Query  62   TATACAAATGGATAAGCCTTTGGTCTGATTGTATAGGAGTTTTTGAACTAAAAAAGGAGC  121
            | |||||||||||||| |||||||||||||||||||||||||||||||||||||||||||
Sbjct  488  TCTACAAATGGATAAGACTTTGGTCTGATTGTATAGGAGTTTTTGAACTAAAAAAGGAGC  429
```

```
Query  122  AATAACGCCCTCTTGATAAAACAAGAGGGAAGCTATTGCTCCttttttttATTTAGTACT  181
            ||||||||||||||| |  | |||||||||||||||||||||||||||||||||||||||
Sbjct  428  AATAACGCCCTCTTGTTTTATCAAGAGGGAAGCTATTGCTCCTTTTTTTTATTTAGTACT  369

Query  182  ATTTGCCTTACATAGTTTCTTTAAA-------AATAACAAGTACTTTTTATAGTTTGGTT  234
            |||||||||||||||||||||||||       ||||||||||||||||||||||||||||
Sbjct  368  ATTTGCCTTACATAGTTTCTTTAAACTTTAAAAATAACAAGTACTTTTTATAGTTTGGTT  309

Query  235  CGATTTGCGTGttttttctttgtattcatttatattataggtttctatatcatatatttt  294
            |||||||  |||||||| |||||||||||||||||||||||||||||||||||||| |||
Sbjct  308  CGATTTGTTTGTTTTTTATTTGTATTCATTTATATTATAGGTTTCTATATCATATACTTT  249

Query  295  t-c----cc-aatcttttatgaagttttattt--ACAATTTAATTTCAATCGAAAATAGA  346
            | |    || |||||||||||||||||| |||  ||||| ||| ||||||||||||||||
Sbjct  248  TACTTTTCCTAATCTTTTATGAAGTTTTTTTTTTACAATCTAAATTCAATCGAAAATAGA  189

Query  347  GAAAAATGACAATTTTGCTTTTTGATT--TA-TATTGATTAAATCAAAATAAGAAAGAAT  403
             |||||||||||||||| || || |||  |  |||| ||| ||| |||||  |||||||||
Sbjct  188  TAAAAATGACAATTTTGTTTATTTATTACTTTTATTTATTCAATAAAAATGAGAAAGAAT  129

Query  404  ATGCtttttttATGTTAATGGAAAAATATAGTAATACTAGATAATCGTAGAGGGGCGGAT  463
            ||||||||||| |||||||||| ||||||||||||||||||||||| ||||||||||||||
Sbjct  128  ATGCTTTTTT-ATGTTAATGGGAAAATATAGTAATACTAGATAATAGTAGAGGGGCGGAT  70

Query  464  GTAGCCAAGTGGATCAAGGCAGTGGATTGTGAATCCACCATGCGCGG  510
            |||||||||||||||||||||||||||||||||||||||||||||||
Sbjct  69   GTAGCCAAGTGGATCAAGGCAGTGGATTGTGAATCCACCATGCGCGG  23


>NC_057181.1 Aldama veredensis voucher Loeuille 921 chloroplast, complete
genome
 MN356035.1 Aldama veredensis chloroplast, complete genome
Length=151331

 Score = 717 bits (388),  Expect = 0.0
 Identities = 484/527 (92%), Gaps = 19/527 (4%)
 Strand=Plus/Minus

Query  2    GTTATGCATGAACGTAATGCTCATAATTTCCCTCTAGACTTAGCTGCTATTGAAGCTCCA  61
            ||||||||||||||||||||||||||||||||||||||||||||||||||||||||||||
Sbjct  548  GTTATGCATGAACGTAATGCTCATAATTTCCCTCTAGACTTAGCTGCTATTGAAGCTCCA  489

Query  62   TATACAAATGGATAAGCCTTTGGTCTGATTGTATAGGAGTTTTTGAACTAAAAAAGGAGC  121
            | |||||||||||||| |||||||||||||||||||||||||||||||||||||||||||
Sbjct  488  TCTACAAATGGATAAGACTTTGGTCTGATTGTATAGGAGTTTTTGAACTAAAAAAGGAGC  429

Query  122  AATAACG-CCCTCTTGATAAAACAAGAGGGAAGCTATTGCTCCttttttttATTTAGTAC  180
            ||||||| |||||||| |  | ||||||||||||||||||||||||||||||||||||||
Sbjct  428  AATAACGNCCCTCTTGTTTTATCAAGAGGGAAGCTATTGCTCCTTTTTTTTATTTAGTAC  369

Query  181  TATTTGCCTTACATAGTTTCTTTAAA-------AATAACAAGTACTTTTTATAGTTTGGT  233
            ||||||||||||||||||||||||||       |||||||||||||||||||||||||||
Sbjct  368  TATTTGCCTTACATAGTTTCTTTAAACTTTAAAAATAACAAGTACTTTTTATAGTTTGGT  309

Query  234  TCGATTTGCGTGttttttctttgtattcatttatattataggtttctatatcatatattt  293
            ||||||||  |||||||| |||||||||||||||||||||||||||||||||||||| ||
```

```
Sbjct  308   TCGATTTGTTTGTTTTTTATTTGTATTCATTTATATTATAGGTTTCTATATCATATACTT  249

Query  294   tt-c----cc-aatcttttatgaagttttattt-ACAATTTAATTTCAATCGAAAATAGA  346
             || |    || |||||||||||||||||| ||| ||||| ||| ||||||||||||||||
Sbjct  248   TTACTTTTCCTAATCTTTTATGAAGTTTTTTTTACAATCTAAATTCAATCGAAAATAGA  189

Query  347   GAAAAATGACAATTTTGCTTTTTGATT--TA-TATTGATTAAATCAAAATAAGAAAGAAT  403
              |||||||||||||||| || || |||  |  |||| ||| ||| ||||| |||||||||
Sbjct  188   TAAAAATGACAATTTTGTTTATTTATTACTTTTATTTATTCAATAAAAATGAGAAAGAAT  129

Query  404   ATGCtttttttATGTTAATGGAAAAATATAGTAATACTAGATAATCGTAGAGGGGCGGAT  463
             |||||||||| |||||||||| ||||||||||||||||||||||| ||||||||||||||
Sbjct  128   ATGCTTTTTT-ATGTTAATGGGAAAATATAGTAATACTAGATAATAGTAGAGGGGCGGAT  70

Query  464   GTAGCCAAGTGGATCAAGGCAGTGGATTGTGAATCCACCATGCGCGG  510
             |||||||||||||||||||||||||||||||||||||||||||||||
Sbjct  69    GTAGCCAAGTGGATCAAGGCAGTGGATTGTGAATCCACCATGCGCGG  23


>NC_057178.1 Aldama tenuifolia voucher Silva s.n. (ESA 122870) chloroplast,
complete genome
 MN356032.1 Aldama tenuifolia chloroplast, complete genome
Length=151372

 Score = 717 bits (388),  Expect = 0.0
 Identities = 484/527 (92%), Gaps = 19/527 (4%)
 Strand=Plus/Minus

Query  2     GTTATGCATGAACGTAATGCTCATAATTTCCCTCTAGACTTAGCTGCTATTGAAGCTCCA  61
             ||||||||||||||||||||||||||||||||||||||||||||||||||||||||||||
Sbjct  548   GTTATGCATGAACGTAATGCTCATAATTTCCCTCTAGACTTAGCTGCTATTGAAGCTCCA  489

Query  62    TATACAAATGGATAAGCCTTTGGTCTGATTGTATAGGAGTTTTTGAACTAAAAAAGGAGC  121
             | |||||||||||||| |||||||||||||||||||||||||||||||||||||||||||
Sbjct  488   TCTACAAATGGATAAGACTTTGGTCTGATTGTATAGGAGTTTTTGAACTAAAAAAGGAGC  429

Query  122   AATAACG-CCCTCTTGATAAAACAAGAGGGAAGCTATTGCTCCttttttttATTTAGTAC  180
             ||||||| |||||||| |  | ||||||||||||||||||||||||||||||||||||||
Sbjct  428   AATAACGNCCCTCTTGTTTTATCAAGAGGGAAGCTATTGCTCCTTTTTTTTATTTAGTAC  369

Query  181   TATTTGCCTTACATAGTTTCTTTAAA-------AATAACAAGTACTTTTTATAGTTTGGT  233
             ||||||||||||||||||||||||||       |||||||||||||||||||||||||||
Sbjct  368   TATTTGCCTTACATAGTTTCTTTAAACTTTAAAAATAACAAGTACTTTTTATAGTTTGGT  309

Query  234   TCGATTTGCGTGtttttctttgtattcatttatattataggtttctatatcatatattt  293
             ||||||||  |||||||| |||||||||||||||||||||||||||||||||||||| ||
Sbjct  308   TCGATTTGTTTGTTTTTTATTTGTATTCATTTATATTATAGGTTTCTATATCATATACTT  249

Query  294   tt-c----cc-aatcttttatgaagttttattt-ACAATTTAATTTCAATCGAAAATAGA  346
             || |    || |||||||||||||||||| ||| ||||| ||| ||||||||||||||||
Sbjct  248   TTACTTTTCCTAATCTTTTATGAAGTTTTTTTTACAATCTAAATTCAATCGAAAATAGA  189

Query  347   GAAAAATGACAATTTTGCTTTTTGATT--TA-TATTGATTAAATCAAAATAAGAAAGAAT  403
              |||||||||||||||| || || |||  |  |||| ||| ||| ||||| |||||||||
Sbjct  188   TAAAAATGACAATTTTGTTTATTTATTACTTTTATTTATTCAATAAAAATGAGAAAGAAT  129

Query  404   ATGCtttttttATGTTAATGGAAAAATATAGTAATACTAGATAATCGTAGAGGGGCGGAT  463
```

```
            |||||||||| |||||||||| ||||||||||||||||||||||| ||||||||||||||
Sbjct  128  ATGCTTTTTT-ATGTTAATGGGAAAATATAGTAATACTAGATAATAGTAGAGGGGCGGAT  70

Query  464  GTAGCCAAGTGGATCAAGGCAGTGGATTGTGAATCCACCATGCGCGG  510
            |||||||||||||||||||||||||||||||||||||||||||||||
Sbjct  69   GTAGCCAAGTGGATCAAGGCAGTGGATTGTGAATCCACCATGCGCGG  23


>NC_053293.1 Aldama aspilioides voucher Filartiga 18 chloroplast, complete
genome
 MN337905.1 Viguiera aspilioides voucher Filartiga 18 chloroplast, complete
genome
Length=151362

 Score = 717 bits (388),  Expect = 0.0
 Identities = 484/527 (92%), Gaps = 19/527 (4%)
 Strand=Plus/Minus

Query  2    GTTATGCATGAACGTAATGCTCATAATTTCCCTCTAGACTTAGCTGCTATTGAAGCTCCA  61
            ||||||||||||||||||||||||||||||||||||||||||||||||||||||||||||
Sbjct  546  GTTATGCATGAACGTAATGCTCATAATTTCCCTCTAGACTTAGCTGCTATTGAAGCTCCA  487

Query  62   TATACAAATGGATAAGCCTTTGGTCTGATTGTATAGGAGTTTTTGAACTAAAAAAGGAGC  121
            | |||||||||||||| |||||||||||||||||||||||||||||||||||||||||||
Sbjct  486  TCTACAAATGGATAAGACTTTGGTCTGATTGTATAGGAGTTTTTGAACTAAAAAAGGAGC  427

Query  122  AATAACGCCCTCTTGATAAAACAAGAGGGAAGCTATTGCTCCtttttttATTTAGTACT  181
            ||||||||||||||| |  | |||||||||||||||||||||||||||||||||||||||
Sbjct  426  AATAACGCCCTCTTGTTTTATCAAGAGGGAAGCTATTGCTCCTTTTTTTTATTTAGTACT  367

Query  182  ATTTGCCTTACATAGTTTCTTTAAA-------AATAACAAGTACTTTTTATAGTTTGGTT  234
            |||||||||||||||||||||||||       ||||||||||||||||||||||||||||
Sbjct  366  ATTTGCCTTACATAGTTTCTTTAAACTTTAAAAATAACAAGTACTTTTTATAGTTTGGTT  307

Query  235  CGATTTGCGTGtttttctttgtattcatttatattataggtttctatatcatatatttt  294
            |||||||  |||||||| |||||||||||||||||||||||||||||||||||||| |||
Sbjct  306  CGATTTGTTTGTTTTTTATTTGTATTCATTTATATTATAGGTTTCTATATCATATACTTT  247

Query  295  t-c----cc-aatcttttatgaagttttattt--ACAATTTAATTTCAATCGAAAATAGA  346
            | |    || |||||||||||||||||| |||  ||||| ||| ||||||||||||||||
Sbjct  246  TACTTTTCCTAATCTTTTATGAAGTTTTTTTTTACAATCTAAATTCAATCGAAAATAGA  187

Query  347  GAAAAATGACAATTTTGCTTTTTGATT--TA-TATTGATTAAATCAAAATAAGAAAGAAT  403
             |||||||||||||||| || || |||  |  |||| ||| ||| ||||| |||||||||
Sbjct  186  TAAAAATGACAATTTTGTTTATTTATTACTTTTATTTATTCAATAAAAATGAGAAAGAAT  127

Query  404  ATGCtttttttATGTTAATGGAAAAATATAGTAATACTAGATAATCGTAGAGGGGCGGAT  463
            |||||||||| |||||||||| ||||||||||||||||||||||| ||||||||||||||
Sbjct  126  ATGCTTTTTT-ATGTTAATGGGAAAATATAGTAATACTAGATAATAGTAGAGGGGCGGAT  68

Query  464  GTAGCCAAGTGGATCAAGGCAGTGGATTGTGAATCCACCATGCGCGG  510
            |||||||||||||||||||||||||||||||||||||||||||||||
Sbjct  67   GTAGCCAAGTGGATCAAGGCAGTGGATTGTGAATCCACCATGCGCGG  21


>NC_053700.1 Sigesbeckia orientalis chloroplast, complete genome
 MN240004.1 Sigesbeckia orientalis chloroplast, complete genome
```

```
Length=151821

 Score = 713 bits (386),  Expect = 0.0
 Identities = 474/515 (92%), Gaps = 12/515 (2%)
 Strand=Plus/Minus

Query  2    GTTATGCATGAACGTAATGCTCATAATTTCCCTCTAGACTTAGCTGCTATTGAAGCTCCA  61
            ||||||||||||||||||||||||||||||||||||||||||||||||||||||||||||
Sbjct  537  GTTATGCATGAACGTAATGCTCATAATTTCCCTCTAGACTTAGCTGCTATTGAAGCTCCA  478

Query  62   TATACAAATGGATAAGCCTTTGGTCTGATTGTATAGGAGTTTTTGAACTAAAAAAGGAGC  121
            | ||||||||||||| ||||||||||||||||||||||||||||||||||||||||||||
Sbjct  477  TCTACAAATGGATAAGACTTTGGTCTGATTGTATAGGAGTTTTTGAACTAAAAAAGGAGC  418

Query  122  AATAAC-GCCCTCTTGATAAAACAAGAGGGAAGCTATTGCTCCttttttttATTTAGTAC  180
            |||| |   ||||||| |  |  |||||||  | ||||||||||||||||||||||||||
Sbjct  417  AATAGCTTACCTCTTGTTTTATTAAGAGGG-CGTTATTGCTCCTTTTTTTTATTTAGTAC  359

Query  181  TATTTGCCTTACATAGTTTCTTTAAAAATAACAAGTACTTTTTATAGTTTGGTTCGATTT  240
            ||| |||||| |||||||||||||||||||||||| ||||||||||||||||||||||||
Sbjct  358  TATGTGCCTTCCATAGTTTCTTTAAAAATAACAAGGACTTTTTATAGTTTGGTTCGATTT  299

Query  241  GCGTGttttttctttgtattcatttatattataggtttctatatca-t-atat--ttttc  296
            |||||||||||||||||| ||||||||||||||||||||||||||| | ||||  |||||
Sbjct  298  GCGTGTTTTTTCTTTGTAGTCATTTATATTATAGGTTTCTATATCAATCATATACTTTTC  239

Query  297  ccaatcttttatgaagttttatttACAATTTAATTTCAATCGAAAATAGAGAAAAAT-GA  355
            |||||||||||||||||||||||| ||||| ||||||||||||||||||| |||||| ||
Sbjct  238  CCAATCTTTTATGAAGTTTTATTTCCAATTCAATTTCAATCGAAAATAGATAAAAATTGA  179

Query  356  CAATTTTGCTTTTTGATTTATATTGATTAAATCAAAATAAGAAAGAATATGCtttttttA  415
             |||||||||| || |||  | || |||  ||  |||||| ||||||||||||||||| |
Sbjct  178  -AATTTTGCTTATTTATTCCT-TTTATT-TAT-TAAATAATAAAGAATATGCTTTTTT-A  124

Query  416  TGTTAATGGAAAAATATAGTAATACTAGATAATCGTAGAGGGGCGGATGTAGCCAAGTGG  475
            |||||||||||||||||| |||||||||||||| ||||||||||||||||||||||||||
Sbjct  123  TGTTAATGGAAAAATATACTAATACTAGATAATAGTAGAGGGGCGGATGTAGCCAAGTGG  64

Query  476  ATCAAGGCAGTGGATTGTGAATCCACCATGCGCGG  510
            |||||||||||||||||||||||||||||||||||
Sbjct  63   ATCAAGGCAGTGGATTGTGAATCCACCATGCGCGG  29


>NC_057182.1 Aldama vernonioides voucher Magenta 460 chloroplast, complete
genome
 MN356036.1 Aldama vernonioides chloroplast, complete genome
Length=151221

 Score = 713 bits (386),  Expect = 0.0
 Identities = 484/528 (92%), Gaps = 20/528 (4%)
 Strand=Plus/Minus

Query  2    GTTATGCATGAACGTAATGCTCATAATTTCCCTCTAGACTTAGCTGCTATTGAAGCTCCA  61
            ||||||||||||||||||||||||||||||||||||||||||||||||||||||||||||
Sbjct  549  GTTATGCATGAACGTAATGCTCATAATTTCCCTCTAGACTTAGCTGCTATTGAAGCTCCA  490

Query  62   TATACAAATGGATAAGCCTTTGGTCTGATTGTATAGGAGTTTTTGAACTAAAAAAGGAGC  121
```

```
           | |||||||||||||| |||||||||||||||||||||||||||||||||||||||||||
Sbjct  489  TCTACAAATGGATAAGACTTTGGTCTGATTGTATAGGAGTTTTTGAACTAAAAAAGGAGC  430

Query  122  AATAACGCCCTCTTGATAAAACAAGAGGGAAGCTATTGCTCCtttttttATTTAGTACT  181
            ||||||||||||||| |  | |||||||||||||||||||||||||||||||||||||||
Sbjct  429  AATAACGCCCTCTTGTTTTATCAAGAGGGAAGCTATTGCTCCTTTTTTTTATTTAGTACT  370

Query  182  ATTTGCCTTACATAGTTTCTTTAAA-------AATAACAAGTACTTTTTATAGTTTGGTT  234
            |||||||||||||||||||||||||       ||||||||||||||||||||||||||||
Sbjct  369  ATTTGCCTTACATAGTTTCTTTAAACTTTAAAAATAACAAGTACTTTTTATAGTTTGGTT  310

Query  235  CGATTTGCGTGtttttctttgtattcatttatattataggtttctatatcatatatttt  294
            |||||||  ||||||| ||||||||||||||||||||||||||||||||||||||| |||
Sbjct  309  CGATTTGTTTGTTTTTATTTGTATTCATTTATATTATAGGTTTCTATATCATATACTTT  250

Query  295  t-c----cc-aatcttttatgaagttttattt---ACAATTTAATTTCAATCGAAAATAG  345
            | |    || |||||||||||||||||| |||   ||||| ||| |||||||||||||||
Sbjct  249  TACTTTTCCTAATCTTTTATGAAGTTTTTTTTTTACAATCTAAATTCAATCGAAAATAG  190

Query  346  AGAAAAATGACAATTTTGCTTTTTGATT--TAT-ATTGATTAAATCAAAATAAGAAAGAA  402
            | |||||||||||||||| || || |||  | | ||| ||| ||| ||||| ||||||||
Sbjct  189  ATAAAAATGACAATTTTGTTTATTTATTACTTTGATTTATTCAATAAAAATGAGAAAGAA  130

Query  403  TATGCtttttttATGTTAATGGAAAAATATAGTAATACTAGATAATCGTAGAGGGGCGGA  462
            ||||||||||| |||||||||| ||||||||||||||||||||||| |||||||||||||
Sbjct  129  TATGCTTTTTT-ATGTTAATGGGAAAATATAGTAATACTAGATAATAGTAGAGGGGCGGA  71

Query  463  TGTAGCCAAGTGGATCAAGGCAGTGGATTGTGAATCCACCATGCGCGG  510
            ||||||||||||||||||||||||||||||||||||||||||||||||
Sbjct  70   TGTAGCCAAGTGGATCAAGGCAGTGGATTGTGAATCCACCATGCGCGG  23


>NC_057180.1 Aldama tukumanensis voucher Heiden 1837 chloroplast, complete
genome
 MN356034.1 Aldama tukumanensis chloroplast, complete genome
Length=151336

 Score = 713 bits (386),  Expect = 0.0
 Identities = 484/528 (92%), Gaps = 20/528 (4%)
 Strand=Plus/Minus

Query  2    GTTATGCATGAACGTAATGCTCATAATTTCCCTCTAGACTTAGCTGCTATTGAAGCTCCA  61
            ||||||||||||||||||||||||||||||||||||||||||||||||||||||||||||
Sbjct  549  GTTATGCATGAACGTAATGCTCATAATTTCCCTCTAGACTTAGCTGCTATTGAAGCTCCA  490

Query  62   TATACAAATGGATAAGCCTTTGGTCTGATTGTATAGGAGTTTTTGAACTAAAAAAGGAGC  121
            | |||||||||||||| |||||||||||||||||||||||||||||||||||||||||||
Sbjct  489  TCTACAAATGGATAAGACTTTGGTCTGATTGTATAGGAGTTTTTGAACTAAAAAAGGAGC  430

Query  122  AATAACGCCCTCTTGATAAAACAAGAGGGAAGCTATTGCTCCtttttttATTTAGTACT  181
            ||||||||||||||| |  | |||||||||||||||||||||||||||||||||||||||
Sbjct  429  AATAACGCCCTCTTGTTTTATCAAGAGGGAAGCTATTGCTCCTTTTTTTTATTTAGTACT  370

Query  182  ATTTGCCTTACATAGTTTCTTTAAA-------AATAACAAGTACTTTTTATAGTTTGGTT  234
            |||||||||||||||||||||||||       ||||||||||||||||||||||||||||
Sbjct  369  ATTTGCCTTACATAGTTTCTTTAAACTTTAAAAATAACAAGTACTTTTTATAGTTTGGTT  310
```

```
Query  235  CGATTTGCGTGtttttctttgtattcatttatattataggtttctatatcatatatttt  294
            |||||||  |||||||| |||||||||||||||||||||||||||||||||||||| |||
Sbjct  309  CGATTTGTTTGTTTTTTATTTGTATTCATTTATATTATAGGTTTCTATATCATATACTTT  250

Query  295  t-c----cc-aatcttttatgaagttttattt---ACAATTTAATTTCAATCGAAAATAG  345
            | |    || ||||||||||||||||| |||   ||||| ||| ||||||||||||||||
Sbjct  249  TACTTTTCCTAATCTTTTATGAAGTTTTTTTTTTACAATCTAAATTCAATCGAAAATAG  190

Query  346  AGAAAAATGACAATTTTGCTTTTTGATT--TA-TATTGATTAAATCAAAATAAGAAAGAA  402
            | |||||||||||||||| || || |||  |  |||| ||| ||| ||||| ||||||||
Sbjct  189  ATAAAAATGACAATTTTGTTTATTTATTACTTTTATTTATTCAATAAAAATGAGAAAGAA  130

Query  403  TATGCtttttttATGTTAATGGAAAAATATAGTAATACTAGATAATCGTAGAGGGGCGGA  462
            ||||||||||| |||||||||| ||||||||||||||||||||||| |||||||||||||
Sbjct  129  TATGCTTTTTT-ATGTTAATGGGAAAATATAGTAATACTAGATAATAGTAGAGGGGCGGA  71

Query  463  TGTAGCCAAGTGGATCAAGGCAGTGGATTGTGAATCCACCATGCGCGG  510
            ||||||||||||||||||||||||||||||||||||||||||||||||
Sbjct  70   TGTAGCCAAGTGGATCAAGGCAGTGGATTGTGAATCCACCATGCGCGG  23


>NC_057175.1 Aldama rubra voucher Magenta 388 chloroplast, complete genome
 MN356029.1 Aldama rubra chloroplast, complete genome
Length=151322

 Score = 713 bits (386),  Expect = 0.0
 Identities = 484/528 (92%), Gaps = 20/528 (4%)
 Strand=Plus/Minus

Query  2    GTTATGCATGAACGTAATGCTCATAATTTCCCTCTAGACTTAGCTGCTATTGAAGCTCCA  61
            ||||||||||||||||||||||||||||||||||||||||||||||||||||||||||||
Sbjct  549  GTTATGCATGAACGTAATGCTCATAATTTCCCTCTAGACTTAGCTGCTATTGAAGCTCCA  490

Query  62   TATACAAATGGATAAGCCTTTGGTCTGATTGTATAGGAGTTTTTGAACTAAAAAAGGAGC  121
            | |||||||||||||| |||||||||||||||||||||||||||||||||||||||||||
Sbjct  489  TCTACAAATGGATAAGACTTTGGTCTGATTGTATAGGAGTTTTTGAACTAAAAAAGGAGC  430

Query  122  AATAACGCCCTCTTGATAAAACAAGAGGGAAGCTATTGCTCCttttttttATTTAGTACT  181
            ||||||||||||||| |  | ||||||||||||||||||||||||||||||||||||||||
Sbjct  429  AATAACGCCCTCTTGTTTTATCAAGAGGGAAGCTATTGCTCCTTTTTTTTATTTAGTACT  370

Query  182  ATTTGCCTTACATAGTTTCTTTAAA-------AATAACAAGTACTTTTTATAGTTTGGTT  234
            |||||||||||||||||||||||||       ||||||||||||||||||||||||||||
Sbjct  369  ATTTGCCTTACATAGTTTCTTTAAACTTTAAAAATAACAAGTACTTTTTATAGTTTGGTT  310

Query  235  CGATTTGCGTGtttttctttgtattcatttatattataggtttctatatcatatatttt  294
            |||||||  |||||||| |||||||||||||||||||||||||||||||||||||| |||
Sbjct  309  CGATTTGTTTGTTTTTTATTTGTATTCATTTATATTATAGGTTTCTATATCATATACTTT  250

Query  295  t-c----cc-aatcttttatgaagttttattt---ACAATTTAATTTCAATCGAAAATAG  345
            | |    || ||||||||||||||||| |||   ||||| ||| ||||||||||||||||
Sbjct  249  TACTTTTCCTAATCTTTTATGAAGTTTTTTTTTTACAATCTAAATTCAATCGAAAATAG  190

Query  346  AGAAAAATGACAATTTTGCTTTTTGATT--TA-TATTGATTAAATCAAAATAAGAAAGAA  402
            | |||||||||||||||| || || |||  |  |||| ||| ||| ||||| ||||||||
Sbjct  189  ATAAAAATGACAATTTTGTTTATTTATTACTTTTATTTATTCAATAAAAATGAGAAAGAA  130
```

```
Query  403  TATGCtttttttATGTTAATGGAAAAATATAGTAATACTAGATAATCGTAGAGGGGCGGA  462
            ||||||||||| |||||||||| ||||||||||||||||||||||| |||||||||||||
Sbjct  129  TATGCTTTTTT-ATGTTAATGGGAAAATATAGTAATACTAGATAATAGTAGAGGGGCGGA  71

Query  463  TGTAGCCAAGTGGATCAAGGCAGTGGATTGTGAATCCACCATGCGCGG  510
            ||||||||||||||||||||||||||||||||||||||||||||||||
Sbjct  70   TGTAGCCAAGTGGATCAAGGCAGTGGATTGTGAATCCACCATGCGCGG  23


>NC_053283.1 Aldama grandiflora voucher Loeuille 750 chloroplast, complete
genome
 MN337894.1 Aldama grandiflora chloroplast, complete genome
Length=151353

 Score = 713 bits (386),  Expect = 0.0
 Identities = 484/528 (92%), Gaps = 20/528 (4%)
 Strand=Plus/Minus

Query  2    GTTATGCATGAACGTAATGCTCATAATTTCCCTCTAGACTTAGCTGCTATTGAAGCTCCA  61
            ||||||||||||||||||||||||||||||||||||||||||||||||||||||||||||
Sbjct  549  GTTATGCATGAACGTAATGCTCATAATTTCCCTCTAGACTTAGCTGCTATTGAAGCTCCA  490

Query  62   TATACAAATGGATAAGCCTTTGGTCTGATTGTATAGGAGTTTTTGAACTAAAAAAGGAGC  121
            | |||||||||||||| |||||||||||||||||||||||||||||||||||||||||||
Sbjct  489  TCTACAAATGGATAAGACTTTGGTCTGATTGTATAGGAGTTTTTGAACTAAAAAAGGAGC  430

Query  122  AATAACGCCCTCTTGATAAAACAAGAGGGAAGCTATTGCTCCttttttttATTTAGTACT  181
            ||||||||||||||| |  | |||||||||||||||||||||||||||||||||||||||
Sbjct  429  AATAACGCCCTCTTGTTTTATCAAGAGGGAAGCTATTGCTCCTTTTTTTTATTTAGTACT  370

Query  182  ATTTGCCTTACATAGTTTCTTTAAA-------AATAACAAGTACTTTTTATAGTTTGGTT  234
            |||||||||||||||||||||||||       ||||||||||||||||||||||||||||
Sbjct  369  ATTTGCCTTACATAGTTTCTTTAAACTTTAAAAATAACAAGTACTTTTTATAGTTTGGTT  310

Query  235  CGATTTGCGTGttttttctttgtattcatttatattataggtttctatatcatatatttt  294
            |||||||  |||||||| |||||||||||||||||||||||||||||||||||||| |||
Sbjct  309  CGATTTGTTTGTTTTTTATTTGTATTCATTTATATTATAGGTTTCTATATCATATACTTT  250

Query  295  t-c----cc-aatcttttatgaagttttattt---ACAATTTAATTTCAATCGAAAATAG  345
            | |    || |||||||||||||||||| |||   ||||| ||| |||||||||||||||
Sbjct  249  TACTTTTCCTAATCTTTTATGAAGTTTTTTTTTTTACAATCTAAATTCAATCGAAAATAG  190

Query  346  AGAAAAATGACAATTTTGCTTTTTGATT--TA-TATTGATTAAATCAAAATAAGAAAGAA  402
            | |||||||||||||||| || || |||  |  |||| ||| ||| ||||| ||||||||
Sbjct  189  ATAAAAATGACAATTTTGTTTATTTATTACTTTTATTTATTCAATAAAAATGAGAAAGAA  130

Query  403  TATGCtttttttATGTTAATGGAAAAATATAGTAATACTAGATAATCGTAGAGGGGCGGA  462
            ||||||||||| |||||||||| ||||||||||||||||||||||| |||||||||||||
Sbjct  129  TATGCTTTTTT-ATGTTAATGGGAAAATATAGTAATACTAGATAATAGTAGAGGGGCGGA  71

Query  463  TGTAGCCAAGTGGATCAAGGCAGTGGATTGTGAATCCACCATGCGCGG  510
            ||||||||||||||||||||||||||||||||||||||||||||||||
Sbjct  70   TGTAGCCAAGTGGATCAAGGCAGTGGATTGTGAATCCACCATGCGCGG  23


>NC_053288.1 Aldama nudibasilaris voucher Filartiga 1 chloroplast, complete
genome
```

```
 MN337899.1 Aldama nudibasilaris chloroplast, complete genome
Length=151316

 Score = 713 bits (386),  Expect = 0.0
 Identities = 484/528 (92%), Gaps = 20/528 (4%)
 Strand=Plus/Minus

Query  2    GTTATGCATGAACGTAATGCTCATAATTTCCCTCTAGACTTAGCTGCTATTGAAGCTCCA  61
            ||||||||||||||||||||||||||||||||||||||||||||||||||||||||||||
Sbjct  549  GTTATGCATGAACGTAATGCTCATAATTTCCCTCTAGACTTAGCTGCTATTGAAGCTCCA  490

Query  62   TATACAAATGGATAAGCCTTTGGTCTGATTGTATAGGAGTTTTTGAACTAAAAAAGGAGC  121
            | |||||||||||||| |||||||||||||||||||||||||||||||||||||||||||
Sbjct  489  TCTACAAATGGATAAGACTTTGGTCTGATTGTATAGGAGTTTTTGAACTAAAAAAGGAGC  430

Query  122  AATAACGCCCTCTTGATAAAACAAGAGGGAAGCTATTGCTCCttttttttATTTAGTACT  181
            ||||||||||||||| |  | |||||||||||||||||||||||||||||||||||||||
Sbjct  429  AATAACGCCCTCTTGTTTTATCAAGAGGGAAGCTATTGCTCCTTTTTTTTATTTAGTACT  370

Query  182  ATTTGCCTTACATAGTTTCTTTAAA-------AATAACAAGTACTTTTTATAGTTTGGTT  234
            |||||||||||||||||||||||||       ||||||||||||||||||||||||||||
Sbjct  369  ATTTGCCTTACATAGTTTCTTTAAACTTTAAAAATAACAAGTACTTTTTATAGTTTGGTT  310

Query  235  CGATTTGCGTGtttttctttgtattcatttatattataggtttctatatcatatatttt  294
            |||||||  ||||||| ||||||||||||||||||||||||||||||||||||||| |||
Sbjct  309  CGATTTGTTTGTTTTTATTTGTATTCATTTATATTATAGGTTTCTATATCATATACTTT  250

Query  295  t-c----cc-aatcttttatgaagttttattt---ACAATTTAATTTCAATCGAAAATAG  345
            | |    || |||||||||||||||||| |||   ||||| ||| |||||||||||||||
Sbjct  249  TACTTTTCCTAATCTTTTATGAAGTTTTTTTTTTACAATCTAAATTCAATCGAAAATAG  190

Query  346  AGAAAAATGACAATTTTGCTTTTTGATT--TA-TATTGATTAAATCAAAATAAGAAAGAA  402
            | |||||||||||||||| || || |||  |  |||| ||| ||| ||||| ||||||||
Sbjct  189  ATAAAAATGACAATTTTGTTTATTTATTACTTTTATTTATTCAATAAAAATGAGAAAGAA  130

Query  403  TATGCtttttttATGTTAATGGAAAAATATAGTAATACTAGATAATCGTAGAGGGGCGGA  462
            ||||||||||| |||||||||| ||||||||||||||||||||||| |||||||||||||
Sbjct  129  TATGCTTTTTT-ATGTTAATGGGAAAATATAGTAATACTAGATAATAGTAGAGGGGCGGA  71

Query  463  TGTAGCCAAGTGGATCAAGGCAGTGGATTGTGAATCCACCATGCGCGG  510
            ||||||||||||||||||||||||||||||||||||||||||||||||
Sbjct  70   TGTAGCCAAGTGGATCAAGGCAGTGGATTGTGAATCCACCATGCGCGG  23


>NC_057176.1 Aldama santacatarinensis voucher Magenta 706 chloroplast, complete
genome
 MN356030.1 Aldama santacatarinensis chloroplast, complete genome
Length=151362

 Score = 712 bits (385),  Expect = 0.0
 Identities = 483/527 (92%), Gaps = 20/527 (4%)
 Strand=Plus/Minus

Query  2    GTTATGCATGAACGTAATGCTCATAATTTCCCTCTAGACTTAGCTGCTATTGAAGCTCCA  61
            ||||||||||||||||||||||||||||||||||||||||||||||||||||||||||||
Sbjct  554  GTTATGCATGAACGTAATGCTCATAATTTCCCTCTAGACTTAGCTGCTATTGAAGCTCCA  495
```

```
Query  62   TATACAAATGGATAAGCCTTTGGTCTGATTGTATAGGAGTTTTTGAACTAAAAAAGGAGC  121
            | |||||||||||||| |||||||||||||||||||||||||||||||||||||||||||
Sbjct  494  TCTACAAATGGATAAGACTTTGGTCTGATTGTATAGGAGTTTTTGAACTAAAAAAGGAGC  435

Query  122  AATAACGCCCTCTTGATAAAACAAGAGGGAAGCTATTGCTCCttttttttATTTAGTACT  181
            ||||||||||||||| |  | |||||||||||||||||||||||||||| ||||||||||
Sbjct  434  AATAACGCCCTCTTGTTTTATCAAGAGGGAAGCTATTGCTCCTTTTTTT-ATTTAGTACT  376

Query  182  ATTTGCCTTACATAGTTTCTTTAAA-------AATAACAAGTACTTTTTATAGTTTGGTT  234
            |||||||||||||||||||||||||       ||||||||||||||||||||||||||||
Sbjct  375  ATTTGCCTTACATAGTTTCTTTAAACTTTAAAAATAACAAGTACTTTTTATAGTTTGGTT  316

Query  235  CGATTTGCGTGttttttctttgtattcatttatattataggtttctatatcatata----  290
            |||||||  |||||||| ||||||||||||||||||||||||||||||||||||||
Sbjct  315  CGATTTGTTTGTTTTTTATTTGTATTCATTTATATTATAGGTTTCTATATCATATACTTT  256

Query  291  t--ttttcccaatcttttatgaagtttt--atttACAATTTAATTTCAATCGAAAATAGA  346
            |  |||||| ||||||||||||||||||   |||||||| ||| ||||||||||||||||
Sbjct  255  TACTTTTCCTAATCTTTTATGAAGTTTTTTTTTTACAATCTAAATTCAATCGAAAATAGA  196

Query  347  GAAAAATGACAATTTTGCTTTTTGA-T--TTATATTGATTAAATCAAAATAAGAAAGAAT  403
             |||||||||||||||| || || | |  || |||| ||| ||| ||||| |||||||||
Sbjct  195  TAAAAATGACAATTTTGTTTATTTATTACTTTTATTTATTCAATAAAAATGAGAAAGAAT  136

Query  404  ATGCtttttttATGTTAATGGAAAAATATAGTAATACTAGATAATCGTAGAGGGGCGGAT  463
            |||||||||| |||||||||| ||||||||||||||||||||||| ||||||||||||||
Sbjct  135  ATGCTTTTTT-ATGTTAATGGGAAAATATAGTAATACTAGATAATAGTAGAGGGGCGGAT  77

Query  464  GTAGCCAAGTGGATCAAGGCAGTGGATTGTGAATCCACCATGCGCGG  510
            |||||||||||||||||||||||||||||||||||||||||||||||
Sbjct  76   GTAGCCAAGTGGATCAAGGCAGTGGATTGTGAATCCACCATGCGCGG  30


>NC_053290.1 Aldama pilosa voucher Filartiga 10 chloroplast, complete genome
 MN337901.1 Aldama pilosa chloroplast, complete genome
Length=151354

 Score = 712 bits (385),  Expect = 0.0
 Identities = 483/527 (92%), Gaps = 20/527 (4%)
 Strand=Plus/Minus

Query  2    GTTATGCATGAACGTAATGCTCATAATTTCCCTCTAGACTTAGCTGCTATTGAAGCTCCA  61
            ||||||||||||||||||||||||||||||||||||||||||||||||||||||||||||
Sbjct  556  GTTATGCATGAACGTAATGCTCATAATTTCCCTCTAGACTTAGCTGCTATTGAAGCTCCA  497

Query  62   TATACAAATGGATAAGCCTTTGGTCTGATTGTATAGGAGTTTTTGAACTAAAAAAGGAGC  121
            | |||||||||||||| |||||||||||||||||||||||||||||||||||||||||||
Sbjct  496  TCTACAAATGGATAAGACTTTGGTCTGATTGTATAGGAGTTTTTGAACTAAAAAAGGAGC  437

Query  122  AATAACGCCCTCTTGATAAAACAAGAGGGAAGCTATTGCTCCttttttttATTTAGTACT  181
            ||||||||||||||| |  | |||||||||||||||||||||||||||| ||||||||||
Sbjct  436  AATAACGCCCTCTTGTTTTATCAAGAGGGAAGCTATTGCTCCTTTTTTT-ATTTAGTACT  378

Query  182  ATTTGCCTTACATAGTTTCTTTAAA-------AATAACAAGTACTTTTTATAGTTTGGTT  234
            |||||||||||||||||||||||||       ||||||||||||||||||||||||||||
Sbjct  377  ATTTGCCTTACATAGTTTCTTTAAACTTTAAAAATAACAAGTACTTTTTATAGTTTGGTT  318
```

```
Query  235  CGATTTGCGTGttttttctttgtattcatttatattataggtttctatatcatata----  290
            |||||||  |||||||| ||||||||||||||||||||||||||||||||||
Sbjct  317  CGATTTGTTTGTTTTTTATTTGTATTCATTTATATTATAGGTTTCTATATCATATACTTT  258

Query  291  t--ttttcccaatcttttatgaagtttt--atttACAATTTAATTTCAATCGAAAATAGA  346
            |  |||||| ||||||||||||||||||   |||||||| ||| ||||||||||||||||
Sbjct  257  TACTTTTCCTAATCTTTTATGAAGTTTTTTTTTTACAATCTAAATTCAATCGAAAATAGA  198

Query  347  GAAAAATGACAATTTTGCTTTTTGA-T--TTATATTGATTAAATCAAAATAAGAAAGAAT  403
             |||||||||||||||| || || | |  || |||| ||| ||| ||||| |||||||||
Sbjct  197  TAAAAATGACAATTTTGTTTATTTATTACTTTTATTTATTCAATAAAAATGAGAAAGAAT  138

Query  404  ATGCtttttttATGTTAATGGAAAAATATAGTAATACTAGATAATCGTAGAGGGGCGGAT  463
            |||||||||| |||||||||| ||||||||||||||||||||||| ||||||||||||||
Sbjct  137  ATGCTTTTTT-ATGTTAATGGGAAAATATAGTAATACTAGATAATAGTAGAGGGGCGGAT  79

Query  464  GTAGCCAAGTGGATCAAGGCAGTGGATTGTGAATCCACCATGCGCGG  510
            |||||||||||||||||||||||||||||||||||||||||||||||
Sbjct  78   GTAGCCAAGTGGATCAAGGCAGTGGATTGTGAATCCACCATGCGCGG  32


>NC_053281.1 Aldama gardneri voucher Filartiga 16 chloroplast, complete genome
 MN337892.1 Aldama gardneri chloroplast, complete genome
Length=151312

 Score = 712 bits (385),  Expect = 0.0
 Identities = 483/527 (92%), Gaps = 19/527 (4%)
 Strand=Plus/Minus

Query  2    GTTATGCATGAACGTAATGCTCATAATTTCCCTCTAGACTTAGCTGCTATTGAAGCTCCA  61
            ||||||||||||||||||||||||||||||||||||||||||||||||||||||||||||
Sbjct  555  GTTATGCATGAACGTAATGCTCATAATTTCCCTCTAGACTTAGCTGCTATTGAAGCTCCA  496

Query  62   TATACAAATGGATAAGCCTTTGGTCTGATTGTATAGGAGTTTTTGAACTAAAAAAGGAGC  121
            | |||||||||||||| |||||||||||||||||||||||||||||||||||||||||||
Sbjct  495  TCTACAAATGGATAAGACTTTGGTCTGATTGTATAGGAGTTTTTGAACTAAAAAAGGAGC  436

Query  122  AATAACGCCCTCTTGATAAAACAAGAGGGAAGCTATTGCTCCttttttttATTTAGTACT  181
            ||||||||||||||| |  | |||||||||||||||||||||||||||||||||||||||
Sbjct  435  AATAACGCCCTCTTGTTTTATCAAGAGGGAAGCTATTGCTCCTTTTTTTTATTTAGTACT  376

Query  182  ATTTGCCTTACATAGTTTCTTTAAA-------AATAACAAGTACTTTTTATAGTTTGGTT  234
            |||||||||||||||||||||||||       ||||||||||||||||||||||||||||
Sbjct  375  ATTTGCCTTACATAGTTTCTTTAAACTTTAAAAATAACAAGTACTTTTTATAGTTTGGTT  316

Query  235  CGATTTGCGTGttttttctttgtattcatttatattataggtttctatatcatatatttt  294
            |||||||  |||||||| |||||||||||||||||||||||||||||||||| |||
Sbjct  315  CGATTTGTTTGTTTTTTATTTGTATTCATTTATATTATAGGTTTCTATATCATATACTTT  256

Query  295  t-c----cc-aatcttttatgaagttttattt--ACAATTTAATTTCAATCGAAAATAGA  346
            | |    || |||||||||||||||||| |||  ||||| ||| ||||||||||||||||
Sbjct  255  TACTTTTCCTAATCTTTTATGAAGTTTTTTTTTTACAATCTAAATTCAATCGAAAATAGA  196

Query  347  GAAAAATGACAATTTTGCTTTTTGATT--TA-TATTGATTAAATCAAAATAAGAAAGAAT  403
             |||||||||||||||| || || |||  |  |||| ||| ||| ||||| |||||||||
Sbjct  195  TAAAAATGACAATTTTGTTTATTTATTACTTTTATTTATTCAATAAAAATGAGAAAGAAT  136
```

```
Query  404  ATGCtttttttATGTTAATGGAAAAATATAGTAATACTAGATAATCGTAGAGGGGCGGAT  463
            |||||||||| || ||||||| ||||||||||||||||||||||| ||||||||||||||
Sbjct  135  ATGCTTTTTT-ATATTAATGGGAAAATATAGTAATACTAGATAATAGTAGAGGGGCGGAT  77

Query  464  GTAGCCAAGTGGATCAAGGCAGTGGATTGTGAATCCACCATGCGCGG  510
            |||||||||||||||||||||||||||||||||||||||||||||||
Sbjct  76   GTAGCCAAGTGGATCAAGGCAGTGGATTGTGAATCCACCATGCGCGG  30


>MN337904.1 Aldama arenaria chloroplast, complete genome
Length=151346

 Score = 712 bits (385),  Expect = 0.0
 Identities = 483/527 (92%), Gaps = 19/527 (4%)
 Strand=Plus/Minus

Query  2    GTTATGCATGAACGTAATGCTCATAATTTCCCTCTAGACTTAGCTGCTATTGAAGCTCCA  61
            ||||||||||||||||||||||||||||||||||||||||||||||||||||||||||||
Sbjct  546  GTTATGCATGAACGTAATGCTCATAATTTCCCTCTAGACTTAGCTGCTATTGAAGCTCCA  487

Query  62   TATACAAATGGATAAGCCTTTGGTCTGATTGTATAGGAGTTTTTGAACTAAAAAAGGAGC  121
            | |||||||||||||| |||||||||||||||||||||||||||||||||||||||||||
Sbjct  486  TCTACAAATGGATAAGACTTTGGTCTGATTGTATAGGAGTTTTTGAACTAAAAAAGGAGC  427

Query  122  AATAACGCCCTCTTGATAAAACAAGAGGGAAGCTATTGCTCCtttttttt-ATTTAGTAC  180
            ||||||||||||||| |  | ||||||||||||||||||||||||||||| |||||||||
Sbjct  426  AATAACGCCCTCTTGTTTTATCAAGAGGGAAGCTATTGCTCCTTTTTTTTTATTTAGTAC  367

Query  181  TATTTGCCTTACATAGTTTCTTTAAA-------AATAACAAGTACTTTTTATAGTTTGGT  233
            ||||||||||||||||||||||||||       |||||||||||||||||||||||||||
Sbjct  366  TATTTGCCTTACATAGTTTCTTTAAACTTTAAAAATAACAAGTACTTTTTATAGTTTGGT  307

Query  234  TCGATTTGCGTGtttttctttgtattcatttatattataggtttctatatcatata---  290
            ||||||||  |||||||  ||||||||||||||||||||||||||||||||||||||
Sbjct  306  TCGATTTGTTTGTTTTTGATTTGTATTCATTTATATTATAGGTTTCTATATCATATACTT  247

Query  291  -t--ttttcccaatcttttatgaagtttt-atttACAATTTAATTTCAATCGAAAATAGA  346
             |  |||||| ||||||||||||||||||  |||||||| ||| ||||||||||||||||
Sbjct  246  TTACTTTTCCTAATCTTTTATGAAGTTTTTTTTTACAATCTAAATTCAATCGAAAATAGA  187

Query  347  GAAAAATGACAATTTTGCTTTTTGA-T--TTATATTGATTAAATCAAAATAAGAAAGAAT  403
             |||||||||||||||| || || | |  || |||| ||| ||| ||||| |||||||||
Sbjct  186  TAAAAATGACAATTTTGTTTATTTATTACTTTTATTTATTCAATAAAAATGAGAAAGAAT  127

Query  404  ATGCtttttttATGTTAATGGAAAAATATAGTAATACTAGATAATCGTAGAGGGGCGGAT  463
            |||||||||| |||||||||| ||||||||||||||||||||||| ||||||||||||||
Sbjct  126  ATGCTTTTTT-ATGTTAATGGGAAAATATAGTAATACTAGATAATAGTAGAGGGGCGGAT  68

Query  464  GTAGCCAAGTGGATCAAGGCAGTGGATTGTGAATCCACCATGCGCGG  510
            |||||||||||||||||||||||||||||||||||||||||||||||
Sbjct  67   GTAGCCAAGTGGATCAAGGCAGTGGATTGTGAATCCACCATGCGCGG  21


>AB427162.1 Ambrosia artemisiifolia chloroplast psbA gene for D1 protein,
complete cds
Length=1693
```

```
 Score = 710 bits (384),  Expect = 0.0
 Identities = 464/499 (93%), Gaps = 19/499 (4%)
 Strand=Plus/Plus

Query  2     GTTATGCATGAACGTAATGCTCATAATTTCCCTCTAGACTTAGCTGCTATTGAAGCTCCA  61
             ||||||||||||||||||||||||||||||||||||||||||||||||||||||||||||
Sbjct  1206  GTTATGCATGAACGTAATGCTCATAATTTCCCTCTAGACTTAGCTGCTATTGAAGCTCCA  1265

Query  62    TATACAAATGGATAAGCCTTTGGTCTGATTGTATAGGAGTTTTTGAACTAAAAAAGGAGC  121
             | |||||||||||||| |||||||||||||||||||||||||||||||||||||||||||
Sbjct  1266  TCTACAAATGGATAAGACTTTGGTCTGATTGTATAGGAGTTTTTGAACTAAAAAAGGAGC  1325

Query  122   AATAACGCCCTCTTGATAAAACAAGAGGGAAGCTATTGCTCCttttttttATTTAGTACT  181
             ||||||| ||||||||||||||||||||||||||||||||||||||||||||||||||||
Sbjct  1326  AATAACGTCCTCTTGATAAAACAAGAGGGAAGCTATTGCTCCTTTTTTTTATTTAGTACT  1385

Query  182   ATTTGCCTTACATAGTTTCTTTAAAAATAACAAGTACTTTTTATAGTTTGGTTCGATTTG  241
             |||||||||||||||||||||||||||||         ||||||||||||||||||||||
Sbjct  1386  ATTTGCCTTACATAGTTTCTTTAAAAATA---------TTTTATAGTTTGGTTCGATTTG  1436

Query  242   CGTGtttttctttgtattcatttatattataggtttctatatcatatatttttcccaat  301
             |||||||||||||||||||||||||||||||||||||||||||||||| |||||||||||
Sbjct  1437  CGTGTTTTTCTTTGTATTCATTTATATTATAGGTTTCTATATCATATACTTTTCCCAAT  1496

Query  302   cttttatgaagttttatttACAATTTAATTTCAATCGAAAATAGAGAAAAATGACAATTT  361
             ||||||||||||||||||||||||| ||||||||||||||||||| ||||||||||||||
Sbjct  1497  CTTTTATGAAGTTTTATTTACAATTCAATTTCAATCGAAAATAGACAAAAATGACAATTT  1556

Query  362   TGCTTTTTGATT--TA-TATTGATTAAATCAAAA---T-AAGAAAGAATATGCttttttt  414
             | ||| || |||  |  |||| ||||||| ||||   | |||||| ||||||||||||
Sbjct  1557  TTCTTATTTATTACTTTTATTTATTAAATAAAAAGAATAAAGAAATAATATGCTTTTTG-  1615

Query  415   ATGTTAAT-GGAAAAATATAGTAATACTAGATAATCGTAGAGGGGCGGATGTAGCCAAGT  473
             || ||||| |||||||||||||||||||||||||  ||||||||||||||||||||||||
Sbjct  1616  AT-TTAATGGGAAAAATATAGTAATACTAGATAACAGTAGAGGGGCGGATGTAGCCAAGT  1674

Query  474   GGATCAAGGCAGTGGATTG  492
             |||||||||||||||||||
Sbjct  1675  GGATCAAGGCAGTGGATTG  1693


>KF746370.1 Helianthus grosseserratus isolate DB25 plastid, complete genome
Length=151045

 Score = 710 bits (384),  Expect = 0.0
 Identities = 483/527 (92%), Gaps = 21/527 (4%)
 Strand=Plus/Minus

Query  2    GTTATGCATGAACGTAATGCTCATAATTTCCCTCTAGACTTAGCTGCTATTGAAGCTCCA  61
            ||||||||||||||||||||||||||||||||||||||||||||||||||||||||||||
Sbjct  545  GTTATGCATGAACGTAATGCTCATAATTTCCCTCTAGACTTAGCTGCTATTGAAGCTCCA  486

Query  62   TATACAAATGGATAAGCCTTTGGTCTGATTGTATAGGAGTTTTTGAACTAAAAAAGGAGC  121
            | |||||||||||||| |||||||||||||||||||||||||||||||||||||||||||
Sbjct  485  TCTACAAATGGATAAGACTTTGGTCTGATTGTATAGGAGTTTTTGAACTAAAAAAGGAGC  426

Query  122  AATAAC-GCCCTCTTGATAAAACAAGAGGGAAGCTATTGCTCCttttttttATTTAGTAC  180
```

```
               |||| |  ||||||||||||||||||||||  | ||||||||||||||||||||||||||
Sbjct  425  AATAGCTTCCCTCTTGATAAAACAAGAGGG-CGTTATTGCTCCTTTTTTTTATTTAGTAC  367

Query  181  TATTTGCCTTACATAGTTTCTTTAAA-------AATAACAAGTACTTTTTATAGTTTGGT  233
            ||||||||||||||||||||||||||       |||||||||||||||||||||||||||
Sbjct  366  TATTTGCCTTACATAGTTTCTTTAAACTTTAAAAATAACAAGTACTTTTTATAGTTTGGT  307

Query  234  TCGATTTGCGTGtttttctttgtattcatttatattataggtttctatatcatata---  290
            ||||||||  |||||||  ||||||||||||||||||||||||||||||||||||||
Sbjct  306  TCGATTTGTTTGTTTTT-ATTTGTATTCATTTATATTATAGGTTTCTATATCATATACTT  248

Query  291  -t--ttttcccaatcttttatgaagtttt-atttACAATTTAATTTCAATCGAAAATAGA  346
             |  |||||| ||||||||||||||||||  |||||||||||| |||||| |||||||||
Sbjct  247  TTACTTTTCCTAATCTTTTATGAAGTTTTTTTTACAATTTAAATTCAATTGAAAATAGA  188

Query  347  GAAAAATGACAATTTTGCTT--TT-TGATTTATATTGATTAAATCAAAATAAGAAAGAAT  403
             |||||||| ||||||||||  || | | || |||| ||| ||| ||||| |||||||||
Sbjct  187  TAAAAATGAAAATTTTGCTTATTTATTACTTTTATTTATTCAATAAAAATGAGAAAGAAT  128

Query  404  ATGCtttttttATGTTAATGGAAAAATATAGTAATACTAGATAATCGTAGAGGGGCGGAT  463
            |||||||||| |||||||||||||||||||||||||||||||||| ||||||||||||||
Sbjct  127  ATGCTTTTTT-ATGTTAATGGAAAAATATAGTAATACTAGATAATAGTAGAGGGGCGGAT  69

Query  464  GTAGCCAAGTGGATCAAGGCAGTGGATTGTGAATCCACCATGCGCGG  510
            |||||||||||||||||||||||||||||||||||||||||||||||
Sbjct  68   GTAGCCAAGTGGATCAAGGCAGTGGATTGTGAATCCACCATGCGCGG  22


>NC_023109.1 Helianthus divaricatus isolate DB07 plastid, complete genome
 KF746352.1 Helianthus divaricatus isolate DB07 plastid, complete genome
Length=151045

 Score = 710 bits (384),  Expect = 0.0
 Identities = 483/527 (92%), Gaps = 21/527 (4%)
 Strand=Plus/Minus

Query  2    GTTATGCATGAACGTAATGCTCATAATTTCCCTCTAGACTTAGCTGCTATTGAAGCTCCA  61
            ||||||||||||||||||||||||||||||||||||||||||||||||||||||||||||
Sbjct  545  GTTATGCATGAACGTAATGCTCATAATTTCCCTCTAGACTTAGCTGCTATTGAAGCTCCA  486

Query  62   TATACAAATGGATAAGCCTTTGGTCTGATTGTATAGGAGTTTTTGAACTAAAAAAGGAGC  121
            | |||||||||||||| |||||||||||||||||||||||||||||||||||||||||||
Sbjct  485  TCTACAAATGGATAAGACTTTGGTCTGATTGTATAGGAGTTTTTGAACTAAAAAAGGAGC  426

Query  122  AATAAC-GCCCTCTTGATAAAACAAGAGGGAAGCTATTGCTCCttttttttATTTAGTAC  180
            |||| |  ||||||||||||||||||||||  | ||||||||||||||||||||||||||
Sbjct  425  AATAGCTTCCCTCTTGATAAAACAAGAGGG-CGTTATTGCTCCTTTTTTTTATTTAGTAC  367

Query  181  TATTTGCCTTACATAGTTTCTTTAAA-------AATAACAAGTACTTTTTATAGTTTGGT  233
            ||||||||||||||||||||||||||       |||||||||||||||||||||||||||
Sbjct  366  TATTTGCCTTACATAGTTTCTTTAAACTTTAAAAATAACAAGTACTTTTTATAGTTTGGT  307

Query  234  TCGATTTGCGTGtttttctttgtattcatttatattataggtttctatatcatata---  290
            ||||||||  |||||||  ||||||||||||||||||||||||||||||||||||||
Sbjct  306  TCGATTTGTTTGTTTTT-ATTTGTATTCATTTATATTATAGGTTTCTATATCATATACTT  248

Query  291  -t--ttttcccaatcttttatgaagtttt-atttACAATTTAATTTCAATCGAAAATAGA  346
```

```
            |  |||||| ||||||||||||||||||  |||||||||||| |||||| |||||||||
Sbjct  247  TTACTTTTCCTAATCTTTTATGAAGTTTTTTTTACAATTTAAATTCAATTGAAAATAGA  188

Query  347  GAAAAATGACAATTTTGCTT--TT-TGATTTATATTGATTAAATCAAAATAAGAAAGAAT  403
             |||||||| ||||||||||  || | | || |||| ||| ||| ||||| |||||||||
Sbjct  187  TAAAAATGAAAATTTTGCTTATTTATTACTTTTATTTATTCAATAAAAATGAGAAAGAAT  128

Query  404  ATGCtttttttATGTTAATGGAAAAATATAGTAATACTAGATAATCGTAGAGGGGCGGAT  463
            |||||||||| |||||||||||||||||||||||||||||||||| ||||||||||||||
Sbjct  127  ATGCTTTTTT-ATGTTAATGGAAAAATATAGTAATACTAGATAATAGTAGAGGGGCGGAT  69

Query  464  GTAGCCAAGTGGATCAAGGCAGTGGATTGTGAATCCACCATGCGCGG  510
            |||||||||||||||||||||||||||||||||||||||||||||||
Sbjct  68   GTAGCCAAGTGGATCAAGGCAGTGGATTGTGAATCCACCATGCGCGG  22


>NC_057179.1 Aldama tuberosa voucher Loeuille 719 chloroplast, complete genome
 MN356033.1 Aldama tuberosa chloroplast, complete genome
Length=151323

 Score = 708 bits (383),  Expect = 0.0
 Identities = 484/529 (91%), Gaps = 21/529 (4%)
 Strand=Plus/Minus

Query  2    GTTATGCATGAACGTAATGCTCATAATTTCCCTCTAGACTTAGCTGCTATTGAAGCTCCA  61
            ||||||||||||||||||||||||||||||||||||||||||||||||||||||||||||
Sbjct  550  GTTATGCATGAACGTAATGCTCATAATTTCCCTCTAGACTTAGCTGCTATTGAAGCTCCA  491

Query  62   TATACAAATGGATAAGCCTTTGGTCTGATTGTATAGGAGTTTTTGAACTAAAAAAGGAGC  121
            | |||||||||||||| |||||||||||||||||||||||||||||||||||||||||||
Sbjct  490  TCTACAAATGGATAAGACTTTGGTCTGATTGTATAGGAGTTTTTGAACTAAAAAAGGAGC  431

Query  122  AATAACGCCCTCTTGATAAAACAAGAGGGAAGCTATTGCTCCttttttttATTTAGTACT  181
            ||||||||||||||| |  | |||||||||||||||||||||||||||||||||||||||
Sbjct  430  AATAACGCCCTCTTGTTTTATCAAGAGGGAAGCTATTGCTCCTTTTTTTTATTTAGTACT  371

Query  182  ATTTGCCTTACATAGTTTCTTTAAA-------AATAACAAGTACTTTTTATAGTTTGGTT  234
            |||||||||||||||||||||||||       ||||||||||||||||||||||||||||
Sbjct  370  ATTTGCCTTACATAGTTTCTTTAAACTTTAAAAATAACAAGTACTTTTTATAGTTTGGTT  311

Query  235  CGATTTGCGTGtttttctttgtattcatttatattataggtttctatatcatatatttt  294
            |||||||  ||||||| ||||||||||||||||||||||||||||||||||||||| |||
Sbjct  310  CGATTTGTTTGTTTTTATTTGTATTCATTTATATTATAGGTTTCTATATCATATACTTT  251

Query  295  t-c----cc-aatcttttatgaagttttattt----ACAATTTAATTTCAATCGAAAATA  344
            | |    || |||||||||||||||||| |||    ||||| ||| ||||||||||||||
Sbjct  250  TACTTTTCCTAATCTTTTATGAAGTTTTTTTTTTTTACAATCTAAATTCAATCGAAAATA  191

Query  345  GAGAAAAATGACAATTTTGCTTTTTGATT--TA-TATTGATTAAATCAAAATAAGAAAGA  401
            || |||||||||||||||| || || |||  |  |||| ||| ||| ||||| |||||||
Sbjct  190  GATAAAAATGACAATTTTGTTTATTTATTACTTTTATTTATTCAATAAAAATGAGAAAGA  131

Query  402  ATATGCtttttttATGTTAATGGAAAAATATAGTAATACTAGATAATCGTAGAGGGGCGG  461
            |||||||||||| |||||||||| ||||||||||||||||||||||| ||||||||||||
Sbjct  130  ATATGCTTTTTT-ATGTTAATGGGAAAATATAGTAATACTAGATAATAGTAGAGGGGCGG  72

Query  462  ATGTAGCCAAGTGGATCAAGGCAGTGGATTGTGAATCCACCATGCGCGG  510
```

```
            |||||||||||||||||||||||||||||||||||||||||||||||||
Sbjct  71   ATGTAGCCAAGTGGATCAAGGCAGTGGATTGTGAATCCACCATGCGCGG  23
```

>NC_053292.1 Aldama arenaria voucher Magenta 275 chloroplast, complete genome
MN337903.1 Aldama arenaria chloroplast, complete genome
Length=151345

Score = 708 bits (383), Expect = 0.0
Identities = 483/528 (91%), Gaps = 20/528 (4%)
Strand=Plus/Minus

```
Query  2    GTTATGCATGAACGTAATGCTCATAATTTCCCTCTAGACTTAGCTGCTATTGAAGCTCCA  61
            ||||||||||||||||||||||||||||||||||||||||||||||||||||||||||||
Sbjct  547  GTTATGCATGAACGTAATGCTCATAATTTCCCTCTAGACTTAGCTGCTATTGAAGCTCCA  488

Query  62   TATACAAATGGATAAGCCTTTGGTCTGATTGTATAGGAGTTTTTGAACTAAAAAAGGAGC  121
            | |||||||||||||| |||||||||||||||||||||||||||||||||||||||||||
Sbjct  487  TCTACAAATGGATAAGACTTTGGTCTGATTGTATAGGAGTTTTTGAACTAAAAAAGGAGC  428

Query  122  AATAACGCCCTCTTGATAAAACAAGAGGGAAGCTATTGCTCCttttttt-ATTTAGTAC  180
            ||||||||||||||| |  | |||||||||||||||||||||||||||| |||||||||
Sbjct  427  AATAACGCCCTCTTGTTTTATCAAGAGGGAAGCTATTGCTCCTTTTTTTTTATTTAGTAC  368

Query  181  TATTTGCCTTACATAGTTTCTTTAAA-------AATAACAAGTACTTTTTATAGTTTGGT  233
            ||||||||||||||||||||||||||       |||||||||||||||||||||||||||
Sbjct  367  TATTTGCCTTACATAGTTTCTTTAAACTTTAAAAATAACAAGTACTTTTTATAGTTTGGT  308

Query  234  TCGATTTGCGTGttttttctttgtattcatttatattataggtttctatatcatata---  290
            ||||||||  |||||||  ||||||||||||||||||||||||||||||||||||||
Sbjct  307  TCGATTTGTTTGTTTTTGATTTGTATTCATTTATATTATAGGTTTCTATATCATATACTT  248

Query  291  -t--ttttcccaatcttttatgaagtttt--atttACAATTTAATTTCAATCGAAAATAG  345
             |  |||||| ||||||||||||||||||   |||||||| ||| |||||||||||||||
Sbjct  247  TTACTTTTCCTAATCTTTTATGAAGTTTTTTTTTTACAATCTAAATTCAATCGAAAATAG  188

Query  346  AGAAAAATGACAATTTTGCTTTTTGA-T--TTATATTGATTAAATCAAAATAAGAAAGAA  402
            | |||||||||||||||| || || | |  || |||| ||| ||| ||||| ||||||||
Sbjct  187  ATAAAAATGACAATTTTGTTTATTTATTACTTTTATTTATTCAATAAAAATGAGAAAGAA  128

Query  403  TATGCtttttttATGTTAATGGAAAAATATAGTAATACTAGATAATCGTAGAGGGGCGGA  462
            ||||||||||| |||||||||| ||||||||||||||||||||||| |||||||||||||
Sbjct  127  TATGCTTTTTT-ATGTTAATGGGAAAATATAGTAATACTAGATAATAGTAGAGGGGCGGA  69

Query  463  TGTAGCCAAGTGGATCAAGGCAGTGGATTGTGAATCCACCATGCGCGG  510
            ||||||||||||||||||||||||||||||||||||||||||||||||
Sbjct  68   TGTAGCCAAGTGGATCAAGGCAGTGGATTGTGAATCCACCATGCGCGG  21
```

>KF746353.1 Helianthus divaricatus isolate DB08 plastid, complete genome
Length=151037

Score = 706 bits (382), Expect = 0.0
Identities = 483/528 (91%), Gaps = 22/528 (4%)
Strand=Plus/Minus

```
Query  2    GTTATGCATGAACGTAATGCTCATAATTTCCCTCTAGACTTAGCTGCTATTGAAGCTCCA  61
```

```
                ||||||||||||||||||||||||||||||||||||||||||||||||||||||||||||
Sbjct  546   GTTATGCATGAACGTAATGCTCATAATTTCCCTCTAGACTTAGCTGCTATTGAAGCTCCA  487

Query  62    TATACAAATGGATAAGCCTTTGGTCTGATTGTATAGGAGTTTTTGAACTAAAAAAGGAGC  121
             | |||||||||||||| |||||||||||||||||||||||||||||||||||||||||||
Sbjct  486   TCTACAAATGGATAAGACTTTGGTCTGATTGTATAGGAGTTTTTGAACTAAAAAAGGAGC  427

Query  122   AATAAC-GCCCTCTTGATAAAACAAGAGGGAAGCTATTGCTCCttttttttATTTAGTAC  180
             |||| |  ||||||||||||||||||||||  | ||||||||||||||||||||||||||
Sbjct  426   AATAGCTTCCCTCTTGATAAAACAAGAGGG-CGTTATTGCTCCTTTTTTTTATTTAGTAC  368

Query  181   TATTTGCCTTACATAGTTTCTTTAAA-------AATAACAAGTACTTTTTATAGTTTGGT  233
             ||||||||||||||||||||||||||       |||||||||||||||||||||||||||
Sbjct  367   TATTTGCCTTACATAGTTTCTTTAAACTTTAAAAATAACAAGTACTTTTTATAGTTTGGT  308

Query  234   TCGATTTGCGTGtttttctttgtattcatttatattataggtttctatatcatata---  290
             ||||||||  |||||||  ||||||||||||||||||||||||||||||||||||||
Sbjct  307   TCGATTTGTTTGTTTTT-ATTTGTATTCATTTATATTATAGGTTTCTATATCATATACTT  249

Query  291   -t--ttttcccaatcttttatgaagtttt--atttACAATTTAATTTCAATCGAAAATAG  345
              |  |||||| ||||||||||||||||||   |||||||||||| |||||| ||||||||
Sbjct  248   TTACTTTTCCTAATCTTTTATGAAGTTTTTTTTTTACAATTTAAATTCAATTGAAAATAG  189

Query  346   AGAAAAATGACAATTTTGCTT--TT-TGATTTATATTGATTAAATCAAAATAAGAAAGAA  402
             | |||||||| ||||||||||  || | | || |||| ||| ||| ||||| ||||||||
Sbjct  188   ATAAAAATGAAAATTTTGCTTATTTATTACTTTTATTTATTCAATAAAAATGAGAAAGAA  129

Query  403   TATGCtttttttATGTTAATGGAAAAATATAGTAATACTAGATAATCGTAGAGGGGCGGA  462
             ||||||||||| |||||||||||||||||||||||||||||||||| |||||||||||||
Sbjct  128   TATGCTTTTTT-ATGTTAATGGAAAAATATAGTAATACTAGATAATAGTAGAGGGGCGGA  70

Query  463   TGTAGCCAAGTGGATCAAGGCAGTGGATTGTGAATCCACCATGCGCGG  510
             ||||||||||||||||||||||||||||||||||||||||||||||||
Sbjct  69    TGTAGCCAAGTGGATCAAGGCAGTGGATTGTGAATCCACCATGCGCGG  22


>NC_023107.1 Helianthus giganteus isolate DB01 plastid, complete genome
 KF746346.1 Helianthus giganteus isolate DB01 plastid, complete genome
Length=151066

 Score = 706 bits (382),  Expect = 0.0
 Identities = 483/528 (91%), Gaps = 22/528 (4%)
 Strand=Plus/Minus

Query  2     GTTATGCATGAACGTAATGCTCATAATTTCCCTCTAGACTTAGCTGCTATTGAAGCTCCA  61
             ||||||||||||||||||||||||||||||||||||||||||||||||||||||||||||
Sbjct  546   GTTATGCATGAACGTAATGCTCATAATTTCCCTCTAGACTTAGCTGCTATTGAAGCTCCA  487

Query  62    TATACAAATGGATAAGCCTTTGGTCTGATTGTATAGGAGTTTTTGAACTAAAAAAGGAGC  121
             | |||||||||||||| |||||||||||||||||||||||||||||||||||||||||||
Sbjct  486   TCTACAAATGGATAAGACTTTGGTCTGATTGTATAGGAGTTTTTGAACTAAAAAAGGAGC  427

Query  122   AATAAC-GCCCTCTTGATAAAACAAGAGGGAAGCTATTGCTCCttttttttATTTAGTAC  180
             |||| |  ||||||||||||||||||||||  | ||||||||||||||||||||||||||
Sbjct  426   AATAGCTTCCCTCTTGATAAAACAAGAGGG-CGTTATTGCTCCTTTTTTTTATTTAGTAC  368

Query  181   TATTTGCCTTACATAGTTTCTTTAAA-------AATAACAAGTACTTTTTATAGTTTGGT  233
```

```
                ||||||||||||||||||||||||||       |||||||||||||||||||||||||||
Sbjct  367   TATTTGCCTTACATAGTTTCTTTAAACTTTAAAAATAACAAGTACTTTTTATAGTTTGGT  308

Query  234   TCGATTTGCGTGtttttctttgtattcatttatattataggtttctatatcatata---  290
             ||||||||  |||||||  ||||||||||||||||||||||||||||||||||||||
Sbjct  307   TCGATTTGTTTGTTTTT-ATTTGTATTCATTTATATTATAGGTTTCTATATCATATACTT  249

Query  291   -t--ttttcccaatcttttatgaagtttt--atttACAATTTAATTTCAATCGAAAATAG  345
              |  |||||| ||||||||||||||||||   |||||||||||| |||||| ||||||||
Sbjct  248   TTACTTTTCCTAATCTTTTATGAAGTTTTTTTTTTACAATTTAAATTCAATTGAAAATAG  189

Query  346   AGAAAAATGACAATTTTGCTT--TT-TGATTTATATTGATTAAATCAAAATAAGAAAGAA  402
             | |||||||| ||||||||||  || | | || |||| ||| ||| ||||| ||||||||
Sbjct  188   ATAAAAATGAAAATTTTGCTTATTTATTACTTTATTTATTCAATAAAAATGAGAAAGAA  129

Query  403   TATGCtttttttATGTTAATGGAAAAATATAGTAATACTAGATAATCGTAGAGGGGCGGA  462
             ||||||||||| |||||||||||||||||||||||||||||||||||| |||||||||||||
Sbjct  128   TATGCTTTTTT-ATGTTAATGGAAAAATATAGTAATACTAGATAATAGTAGAGGGGCGGA  70

Query  463   TGTAGCCAAGTGGATCAAGGCAGTGGATTGTGAATCCACCATGCGCGG  510
             ||||||||||||||||||||||||||||||||||||||||||||||||
Sbjct  69    TGTAGCCAAGTGGATCAAGGCAGTGGATTGTGAATCCACCATGCGCGG  22


>NC_023113.1 Helianthus strumosus isolate DB31 plastid, complete genome
 KF746376.1 Helianthus strumosus isolate DB31 plastid, complete genome
Length=151044

 Score = 706 bits (382),  Expect = 0.0
 Identities = 483/528 (91%), Gaps = 22/528 (4%)
 Strand=Plus/Minus

Query  2     GTTATGCATGAACGTAATGCTCATAATTTCCCTCTAGACTTAGCTGCTATTGAAGCTCCA  61
             ||||||||||||||||||||||||||||||||||||||||||||||||||||||||||||
Sbjct  546   GTTATGCATGAACGTAATGCTCATAATTTCCCTCTAGACTTAGCTGCTATTGAAGCTCCA  487

Query  62    TATACAAATGGATAAGCCTTTGGTCTGATTGTATAGGAGTTTTTGAACTAAAAAAGGAGC  121
             | |||||||||||||| |||||||||||||||||||||||||||||||||||||||||||
Sbjct  486   TCTACAAATGGATAAGACTTTGGTCTGATTGTATAGGAGTTTTTGAACTAAAAAAGGAGC  427

Query  122   AATAAC-GCCCTCTTGATAAAACAAGAGGGAAGCTATTGCTCCtttttttATTTAGTAC  180
             |||| |  ||||||||||||||||||||||  | ||||||||||||||||||||||||||
Sbjct  426   AATAGCTTCCCTCTTGATAAAACAAGAGGG-CGTTATTGCTCCTTTTTTTATTTAGTAC  368

Query  181   TATTTGCCTTACATAGTTTCTTTAAA-------AATAACAAGTACTTTTTATAGTTTGGT  233
             ||||||||||||||||||||||||||       |||||||||||||||||||||||||||
Sbjct  367   TATTTGCCTTACATAGTTTCTTTAAACTTTAAAAATAACAAGTACTTTTTATAGTTTGGT  308

Query  234   TCGATTTGCGTGtttttctttgtattcatttatattataggtttctatatcatata---  290
             ||||||||  |||||||  ||||||||||||||||||||||||||||||||||||||
Sbjct  307   TCGATTTGTTTGTTTTT-ATTTGTATTCATTTATATTATAGGTTTCTATATCATATACTT  249

Query  291   -t--ttttcccaatcttttatgaagtttt--atttACAATTTAATTTCAATCGAAAATAG  345
              |  |||||| ||||||||||||||||||   |||||||||||| |||||| ||||||||
Sbjct  248   TTACTTTTCCTAATCTTTTATGAAGTTTTTTTTTTACAATTTAAATTCAATTGAAAATAG  189

Query  346   AGAAAAATGACAATTTTGCTT--TT-TGATTTATATTGATTAAATCAAAATAAGAAAGAA  402
```

```
            | |||||||| ||||||||||  || | | || |||| ||| ||| ||||| ||||||||
Sbjct  188  ATAAAAATGAAAATTTTGCTTATTTATTACTTTTATTTATTCAATAAAAATGAGAAAGAA  129

Query  403  TATGCtttttttATGTTAATGGAAAAATATAGTAATACTAGATAATCGTAGAGGGGCGGA  462
            ||||||||||| |||||||||||||||||||||||||||||||||| |||||||||||||
Sbjct  128  TATGCTTTTTT-ATGTTAATGGAAAAATATAGTAATACTAGATAATAGTAGAGGGGCGGA  70

Query  463  TGTAGCCAAGTGGATCAAGGCAGTGGATTGTGAATCCACCATGCGCGG  510
            ||||||||||||||||||||||||||||||||||||||||||||||||
Sbjct  69   TGTAGCCAAGTGGATCAAGGCAGTGGATTGTGAATCCACCATGCGCGG  22
```

>PV541157.1 Helianthus ciliaris cultivar PI 653550 chloroplast, complete genome
Length=151147

Score = 706 bits (382), Expect = 0.0
Identities = 483/528 (91%), Gaps = 22/528 (4%)
Strand=Plus/Minus

```
Query  2    GTTATGCATGAACGTAATGCTCATAATTTCCCTCTAGACTTAGCTGCTATTGAAGCTCCA  61
            ||||||||||||||||||||||||||||||||||||||||||||||||||||||||||||
Sbjct  546  GTTATGCATGAACGTAATGCTCATAATTTCCCTCTAGACTTAGCTGCTATTGAAGCTCCA  487

Query  62   TATACAAATGGATAAGCCTTTGGTCTGATTGTATAGGAGTTTTTGAACTAAAAAAGGAGC  121
            | |||||||||||||| |||||||||||||||||||||||||||||||||||||||||||
Sbjct  486  TCTACAAATGGATAAGACTTTGGTCTGATTGTATAGGAGTTTTTGAACTAAAAAAGGAGC  427

Query  122  AATAAC-GCCCTCTTGATAAAACAAGAGGGAAGCTATTGCTCCttttttttATTTAGTAC  180
            |||| |  ||||||||||||||||||||||  | ||||||||||||||||||||||||||
Sbjct  426  AATAGCTTCCCTCTTGATAAAACAAGAGGG-CGTTATTGCTCCTTTTTTTTATTTAGTAC  368

Query  181  TATTTGCCTTACATAGTTTCTTTAAA-------AATAACAAGTACTTTTTATAGTTTGGT  233
            ||||||||||||||||||||||||||       |||||||||||||||||||||||||||
Sbjct  367  TATTTGCCTTACATAGTTTCTTTAAACTTTAAAAATAACAAGTACTTTTTATAGTTTGGT  308

Query  234  TCGATTTGCGTGttttttctttgtattcatttatattataggtttctatatcatata---  290
            ||||||||  |||||||  ||||||||||||||||||||||||||||||||||||||
Sbjct  307  TCGATTTGTTTGTTTTT-ATTTGTATTCATTTATATTATAGGTTTCTATATCATATACTT  249

Query  291  -t--ttttcccaatcttttatgaagtttt--atttACAATTTAATTTCAATCGAAAATAG  345
             |  |||||| ||||||||||||||||||   |||||||||||| |||||| ||||||||
Sbjct  248  TTACTTTTCCTAATCTTTTATGAAGTTTTTTTTTTACAATTTAAATTCAATTGAAAATAG  189

Query  346  AGAAAAATGACAATTTTGCTT--TT-TGATTTATATTGATTAAATCAAAATAAGAAAGAA  402
            | |||||||| ||||||||||  || | | || |||| ||| ||| ||||| ||||||||
Sbjct  188  ATAAAAATGAAAATTTTGCTTATTTATTACTTTTATTTATTCAATAAAAATGAGAAAGAA  129

Query  403  TATGCtttttttATGTTAATGGAAAAATATAGTAATACTAGATAATCGTAGAGGGGCGGA  462
            ||||||||||| |||||||||||||||||||||||||||||||||| |||||||||||||
Sbjct  128  TATGCTTTTTT-ATGTTAATGGAAAAATATAGTAATACTAGATAATAGTAGAGGGGCGGA  70

Query  463  TGTAGCCAAGTGGATCAAGGCAGTGGATTGTGAATCCACCATGCGCGG  510
            ||||||||||||||||||||||||||||||||||||||||||||||||
Sbjct  69   TGTAGCCAAGTGGATCAAGGCAGTGGATTGTGAATCCACCATGCGCGG  22
```

```
>NC_023114.1 Helianthus maximiliani isolate MAX01 plastid, complete genome
 KF746380.1 Helianthus maximiliani isolate MAX01 plastid, complete genome
Length=151007

 Score = 706 bits (382),  Expect = 0.0
 Identities = 483/528 (91%), Gaps = 22/528 (4%)
 Strand=Plus/Minus

Query  2    GTTATGCATGAACGTAATGCTCATAATTTCCCTCTAGACTTAGCTGCTATTGAAGCTCCA  61
            ||||||||||||||||||||||||||||||||||||||||||||||||||||||||||||
Sbjct  546  GTTATGCATGAACGTAATGCTCATAATTTCCCTCTAGACTTAGCTGCTATTGAAGCTCCA  487

Query  62   TATACAAATGGATAAGCCTTTGGTCTGATTGTATAGGAGTTTTTGAACTAAAAAAGGAGC  121
            | |||||||||||||| |||||||||||||||||||||||||||||||||||||||||||
Sbjct  486  TCTACAAATGGATAAGACTTTGGTCTGATTGTATAGGAGTTTTTGAACTAAAAAAGGAGC  427

Query  122  AATAAC-GCCCTCTTGATAAAACAAGAGGGAAGCTATTGCTCCtttttttt-ATTTAGTA  179
            |||| |  ||||||||||||||||||||||  | ||||||||||||||||| ||||||||
Sbjct  426  AATAGCTTCCCTCTTGATAAAACAAGAGGG-CGTTATTGCTCCTTTTTTTTTATTTAGTA  368

Query  180  CTATTTGCCTTACATAGTTTCTTTAAA-------AATAACAAGTACTTTTTATAGTTTGG  232
            |||||||||||||||||||||||||||       ||||||||||||||||||||||||||
Sbjct  367  CTATTTGCCTTACATAGTTTCTTTAAACTTTAAAAATAACAAGTACTTTTTATAGTTTGG  308

Query  233  TTCGATTTGCGTGttttttctttgtattcatttatattataggtttctatatcatata--  290
            |||||||||  |||||||  ||||||||||||||||||||||||||||||||||||||
Sbjct  307  TTCGATTTGTTTGTTTTT-ATTTGTATTCATTTATATTATAGGTTTCTATATCATATACT  249

Query  291  --t--ttttcccaatcttttatgaagtttt-atttACAATTTAATTTCAATCGAAAATAG  345
              |  |||||| ||||||||||||||||||  |||||||||||| |||||| ||||||||
Sbjct  248  TTTACTTTTCCTAATCTTTTATGAAGTTTTTTTTTACAATTTAAATTCAATTGAAAATAG  189

Query  346  AGAAAAATGACAATTTTGCTT--TT-TGATTTATATTGATTAAATCAAAATAAGAAAGAA  402
            | |||||||| ||||||||||  || | | || |||| ||| ||| ||||| ||||||||
Sbjct  188  ATAAAAATGAAAATTTTGCTTATTTATTACTTTTATTTATTCAATAAAAATGAGAAAGAA  129

Query  403  TATGCtttttttATGTTAATGGAAAAATATAGTAATACTAGATAATCGTAGAGGGGCGGA  462
            ||||||||||| |||||||||||||||||||||||||||||||||| |||||||||||||
Sbjct  128  TATGCTTTTTT-ATGTTAATGGAAAAATATAGTAATACTAGATAATAGTAGAGGGGCGGA  70

Query  463  TGTAGCCAAGTGGATCAAGGCAGTGGATTGTGAATCCACCATGCGCGG  510
            ||||||||||||||||||||||||||||||||||||||||||||||||
Sbjct  69   TGTAGCCAAGTGGATCAAGGCAGTGGATTGTGAATCCACCATGCGCGG  22


>NC_086876.1 Helianthus mollis plastid, complete genome
 PP266439.1 Helianthus mollis plastid, complete genome
Length=151060

 Score = 706 bits (382),  Expect = 0.0
 Identities = 483/528 (91%), Gaps = 22/528 (4%)
 Strand=Plus/Minus

Query  2    GTTATGCATGAACGTAATGCTCATAATTTCCCTCTAGACTTAGCTGCTATTGAAGCTCCA  61
            ||||||||||||||||||||||||||||||||||||||||||||||||||||||||||||
Sbjct  614  GTTATGCATGAACGTAATGCTCATAATTTCCCTCTAGACTTAGCTGCTATTGAAGCTCCA  555
```

```
Query  62   TATACAAATGGATAAGCCTTTGGTCTGATTGTATAGGAGTTTTTGAACTAAAAAAGGAGC  121
            | |||||||||||||| |||||||||||||||||||||||||||||||||||||||||||
Sbjct  554  TCTACAAATGGATAAGACTTTGGTCTGATTGTATAGGAGTTTTTGAACTAAAAAAGGAGC  495

Query  122  AATAAC-GCCCTCTTGATAAAACAAGAGGGAAGCTATTGCTCCttttttttATTTAGTAC  180
            |||| |  ||||||||||||||||||||||  | ||||||||||||||||||||||||||
Sbjct  494  AATAGCTTCCCTCTTGATAAAACAAGAGGG-CGTTATTGCTCCTTTTTTTTATTTAGTAC  436

Query  181  TATTTGCCTTACATAGTTTCTTTAAA-------AATAACAAGTACTTTTTATAGTTTGGT  233
            ||||||||||||||||||||||||||       |||||||||||||||||||||||||||
Sbjct  435  TATTTGCCTTACATAGTTTCTTTAAACTTTAAAAATAACAAGTACTTTTTATAGTTTGGT  376

Query  234  TCGATTTGCGTGttttttctttgtattcatttatattataggtttctatatcatata---  290
            ||||||||  |||||||  ||||||||||||||||||||||||||||||||||||||
Sbjct  375  TCGATTTGTTTGTTTTT-ATTTGTATTCATTTATATTATAGGTTTCTATATCATATACTT  317

Query  291  -t--ttttcccaatcttttatgaagtttt--atttACAATTTAATTTCAATCGAAAATAG  345
             |  |||||| ||||||||||||||||||   |||||||||||| |||||| ||||||||
Sbjct  316  TTACTTTTCCTAATCTTTTATGAAGTTTTTTTTTTACAATTTAAATTCAATTGAAAATAG  257

Query  346  AGAAAAATGACAATTTTGCTT--TT-TGATTTATATTGATTAAATCAAAATAAGAAAGAA  402
            | |||||||| ||||||||||  || | | || |||| ||| ||| ||||| ||||||||
Sbjct  256  ATAAAAATGAAAATTTTGCTTATTTATTACTTTTATTTATTCAATAAAAATGAGAAAGAA  197

Query  403  TATGCtttttttATGTTAATGGAAAAATATAGTAATACTAGATAATCGTAGAGGGGCGGA  462
            ||||||||||| |||||||||||||||||||||||||||||||||| |||||||||||||
Sbjct  196  TATGCTTTTTT-ATGTTAATGGAAAAATATAGTAATACTAGATAATAGTAGAGGGGCGGA  138

Query  463  TGTAGCCAAGTGGATCAAGGCAGTGGATTGTGAATCCACCATGCGCGG  510
            ||||||||||||||||||||||||||||||||||||||||||||||||
Sbjct  137  TGTAGCCAAGTGGATCAAGGCAGTGGATTGTGAATCCACCATGCGCGG  90


>KF241793.1 Ambrosia artemisiifolia isolate NJI-7 PsbA (psbA) gene, partial
cds; and psbA-trnH intergenic spacer, partial sequence; chloroplast
Length=485

 Score = 706 bits (382),  Expect = 0.0
 Identities = 461/496 (93%), Gaps = 18/496 (4%)
 Strand=Plus/Plus

Query  8    CATGAACGTAATGCTCATAATTTCCCTCTAGACTTAGCTGCTATTGAAGCTCCATATACA  67
            ||||||||||||||||||||||||||||||||||||||||||||||||||||||| ||||
Sbjct  1    CATGAACGTAATGCTCATAATTTCCCTCTAGACTTAGCTGCTATTGAAGCTCCATCTACA  60

Query  68   AATGGATAAGCCTTTGGTCTGATTGTATAGGAGTTTTTGAACTAAAAAAGGAGCAATAAC  127
            |||||||||| |||||||||||||||||||||||||||||||||||||||||||||||||
Sbjct  61   AATGGATAAGACTTTGGTCTGATTGTATAGGAGTTTTTGAACTAAAAAAGGAGCAATAAC  120

Query  128  GCCCTCTTGATAAAACAAGAGGGAAGCTATTGCTCCttttttttATTTAGTACTATTTGC  187
            | ||||||||||||||||||||||||||||||||||||||||||||||||||||||||||
Sbjct  121  GTCCTCTTGATAAAACAAGAGGGAAGCTATTGCTCCTTTTTTTTATTTAGTACTATTTGC  180

Query  188  CTTACATAGTTTCTTTAAAAATAACAAGTACTTTTTATAGTTTGGTTCGATTTGCGTGtt  247
            |||||||||||||||||||||||         ||||||||||||||||||||||||||||
Sbjct  181  CTTACATAGTTTCTTTAAAAATA---------TTTTATAGTTTGGTTCGATTTGCGTGTT  231
```

```
Query  248  ttttctttgtattcatttatattataggtttctatatcatatatttttcccaatctttta  307
            ||||||||||||||||||||||||||||||||||||||||||| ||||||||||||||||
Sbjct  232  TTTTCTTTGTATTCATTTATATTATAGGTTTCTATATCATATACTTTTCCCAATCTTTTA  291

Query  308  tgaagttttatttACAATTTAATTTCAATCGAAAATAGAGAAAAATGACAATTTTGCTTT  367
            ||||||||||||||||||| ||||| ||||||||||||| ||||||||||||||| ||| 
Sbjct  292  TGAAGTTTTATTTACAATTCAATTTAAATCGAAAATAGACAAAAATGACAATTTTTCTTA  351

Query  368  TTGATT--TA-TATTGATTAAATCAAAA---T-AAGAAAGAATATGCtttttttATGTTA  420
            || |||  |  |||| ||||||| ||||   | |||||| ||||||||||||  || |||
Sbjct  352  TTTATTACTTTTATTTATTAAATAAAAAGAATAAAGAAATAATATGCTTTTTG-AT-TTA  409

Query  421  ATGGAAAAATATAGTAATACTAGATAATCGTAGAGGGGCGGATGTAGCCAAGTGGATCAA  480
            |||||||||||||||||||||||||||  |||||||||||||||||||||||||||||||
Sbjct  410  ATGGAAAAATATAGTAATACTAGATAACAGTAGAGGGGCGGATGTAGCCAAGTGGATCAA  469

Query  481  GGCAGTGGATTGTGAA  496
            ||||||||||||||||
Sbjct  470  GGCAGTGGATTGTGAA  485


>NC_066996.1 Helianthus carnosus chloroplast, complete genome
 OK216126.1 Helianthus carnosus chloroplast, complete genome
Length=151241

 Score = 706 bits (382),  Expect = 0.0
 Identities = 483/528 (91%), Gaps = 22/528 (4%)
 Strand=Plus/Minus

Query  2    GTTATGCATGAACGTAATGCTCATAATTTCCCTCTAGACTTAGCTGCTATTGAAGCTCCA  61
            ||||||||||||||||||||||||||||||||||||||||||||||||||||||||||||
Sbjct  546  GTTATGCATGAACGTAATGCTCATAATTTCCCTCTAGACTTAGCTGCTATTGAAGCTCCA  487

Query  62   TATACAAATGGATAAGCCTTTGGTCTGATTGTATAGGAGTTTTTGAACTAAAAAAGGAGC  121
            | |||||||||||||| |||||||||||||||||||||||||||||||||||||||||||
Sbjct  486  TCTACAAATGGATAAGACTTTGGTCTGATTGTATAGGAGTTTTTGAACTAAAAAAGGAGC  427

Query  122  AATAAC-GCCCTCTTGATAAAACAAGAGGGAAGCTATTGCTCCttttttttATTTAGTAC  180
            |||| |  ||||||||||||||||||||||  | ||||||||||||||||||||||||||
Sbjct  426  AATAGCTTCCCTCTTGATAAAACAAGAGGG-CGTTATTGCTCCTTTTTTTTATTTAGTAC  368

Query  181  TATTTGCCTTACATAGTTTCTTTAAA-------AATAACAAGTACTTTTTATAGTTTGGT  233
            ||||||||||||||||||||||||||       |||||||||||||||||||||||||||
Sbjct  367  TATTTGCCTTACATAGTTTCTTTAAACTTTAAAAATAACAAGTACTTTTTATAGTTTGGT  308

Query  234  TCGATTTGCGTGttttttctttgtattcatttatattataggtttctatatcatata---  290
            ||||||||  |||||||  ||||||||||||||||||||||||||||||||||||||   
Sbjct  307  TCGATTTGTTTGTTTTT-ATTTGTATTCATTTATATTATAGGTTTCTATATCATATACTT  249

Query  291  -t--ttttcccaatcttttatgaagtttt--atttACAATTTAATTTCAATCGAAAATAG  345
             |  |||||| ||||||||||||||||||   |||||||||||| |||||| ||||||||
Sbjct  248  TTACTTTTCCTAATCTTTTATGAAGTTTTTTTTTTACAATTTAAATTCAATTGAAAATAG  189

Query  346  AGAAAAATGACAATTTTGCTT--TT-TGATTTATATTGATTAAATCAAAATAAGAAAGAA  402
            | |||||||| ||||||||||  || | | || |||| ||| ||| ||||| ||||||||
Sbjct  188  ATAAAAATGAAAATTTTGCTTATTTATTACTTTTATTTATTCAATAAAAATGAGAAAGAA  129
```

```
Query  403  TATGCtttttttATGTTAATGGAAAAATATAGTAATACTAGATAATCGTAGAGGGGCGGA  462
            ||||||||||| |||||||||||||||||||||||||||||||||| |||||||||||||
Sbjct  128  TATGCTTTTTT-ATGTTAATGGAAAAATATAGTAATACTAGATAATAGTAGAGGGGCGGA  70

Query  463  TGTAGCCAAGTGGATCAAGGCAGTGGATTGTGAATCCACCATGCGCGG  510
            ||||||||||||||||||||||||||||||||||||||||||||||||
Sbjct  69   TGTAGCCAAGTGGATCAAGGCAGTGGATTGTGAATCCACCATGCGCGG  22
```

>KF746377.1 Helianthus tuberosus isolate DB32 plastid, complete genome
Length=151065

Score = 706 bits (382), Expect = 0.0
Identities = 483/528 (91%), Gaps = 22/528 (4%)
Strand=Plus/Minus

```
Query  2    GTTATGCATGAACGTAATGCTCATAATTTCCCTCTAGACTTAGCTGCTATTGAAGCTCCA  61
            ||||||||||||||||||||||||||||||||||||||||||||||||||||||||||||
Sbjct  546  GTTATGCATGAACGTAATGCTCATAATTTCCCTCTAGACTTAGCTGCTATTGAAGCTCCA  487

Query  62   TATACAAATGGATAAGCCTTTGGTCTGATTGTATAGGAGTTTTTGAACTAAAAAAGGAGC  121
            | |||||||||||||| |||||||||||||||||||||||||||||||||||||||||||
Sbjct  486  TCTACAAATGGATAAGACTTTGGTCTGATTGTATAGGAGTTTTTGAACTAAAAAAGGAGC  427

Query  122  AATAAC-GCCCTCTTGATAAAACAAGAGGGAAGCTATTGCTCCtttttttt-ATTTAGTA  179
            |||| |  ||||||||||||||||||||||  | ||||||||||||||||| ||||||||
Sbjct  426  AATAGCTTCCCTCTTGATAAAACAAGAGGG-CGTTATTGCTCCTTTTTTTTTATTTAGTA  368

Query  180  CTATTTGCCTTACATAGTTTCTTTAAA-------AATAACAAGTACTTTTTATAGTTTGG  232
            |||||||||||||||||||||||||||       ||||||||||||||||||||||||||
Sbjct  367  CTATTTGCCTTACATAGTTTCTTTAAACTTTAAAAATAACAAGTACTTTTTATAGTTTGG  308

Query  233  TTCGATTTGCGTGttttttctttgtattcatttatattataggtttctatatcatata--  290
            |||||||||  |||||||  ||||||||||||||||||||||||||||||||||||||
Sbjct  307  TTCGATTTGTTTGTTTTT-ATTTGTATTCATTTATATTATAGGTTTCTATATCATATACT  249

Query  291  --t--ttttcccaatcttttatgaagtttt-atttACAATTTAATTTCAATCGAAAATAG  345
              |  |||||| ||||||||||||||||||  |||||||||||| |||||| ||||||||
Sbjct  248  TTTACTTTTCCTAATCTTTTATGAAGTTTTTTTTTACAATTTAAATTCAATTGAAAATAG  189

Query  346  AGAAAAATGACAATTTTGCTT--TT-TGATTTATATTGATTAAATCAAAATAAGAAAGAA  402
            | |||||||| ||||||||||  || | | || |||| ||| ||| ||||| ||||||||
Sbjct  188  ATAAAAATGAAAATTTTGCTTATTTATTACTTTATTTATTCAATAAAAATGAGAAAGAA  129

Query  403  TATGCtttttttATGTTAATGGAAAAATATAGTAATACTAGATAATCGTAGAGGGGCGGA  462
            ||||||||||| |||||||||||||||||||||||||||||||||| |||||||||||||
Sbjct  128  TATGCTTTTTT-ATGTTAATGGAAAAATATAGTAATACTAGATAATAGTAGAGGGGCGGA  70

Query  463  TGTAGCCAAGTGGATCAAGGCAGTGGATTGTGAATCCACCATGCGCGG  510
            ||||||||||||||||||||||||||||||||||||||||||||||||
Sbjct  69   TGTAGCCAAGTGGATCAAGGCAGTGGATTGTGAATCCACCATGCGCGG  22
```

>KF746349.1 Helianthus giganteus isolate DB04 plastid, complete genome
Length=151027

Score = 706 bits (382), Expect = 0.0

```
 Identities = 483/528 (91%), Gaps = 22/528 (4%)
 Strand=Plus/Minus

Query  2    GTTATGCATGAACGTAATGCTCATAATTTCCCTCTAGACTTAGCTGCTATTGAAGCTCCA  61
            ||||||||||||||||||||||||||||||||||||||||||||||||||||||||||||
Sbjct  546  GTTATGCATGAACGTAATGCTCATAATTTCCCTCTAGACTTAGCTGCTATTGAAGCTCCA  487

Query  62   TATACAAATGGATAAGCCTTTGGTCTGATTGTATAGGAGTTTTTGAACTAAAAAAGGAGC  121
            | |||||||||||||| |||||||||||||||||||||||||||||||||||||||||||
Sbjct  486  TCTACAAATGGATAAGACTTTGGTCTGATTGTATAGGAGTTTTTGAACTAAAAAAGGAGC  427

Query  122  AATAAC-GCCCTCTTGATAAAACAAGAGGGAAGCTATTGCTCCttttttttt-ATTTAGTA  179
            |||| |  ||||||||||||||||||||||  | |||||||||||||||||| ||||||||
Sbjct  426  AATAGCTTCCCTCTTGATAAAACAAGAGGG-CGTTATTGCTCCTTTTTTTTTATTTAGTA  368

Query  180  CTATTTGCCTTACATAGTTTCTTTAAA-------AATAACAAGTACTTTTTATAGTTTGG  232
            |||||||||||||||||||||||||||       |||||||||||||||||||||||||
Sbjct  367  CTATTTGCCTTACATAGTTTCTTTAAACTTTAAAAATAACAAGTACTTTTTATAGTTTGG  308

Query  233  TTCGATTTGCGTGtttttctttgtattcatttatattataggtttctatatcatata--  290
            |||||||||  |||||||  ||||||||||||||||||||||||||||||||||||||
Sbjct  307  TTCGATTTGTTTGTTTTT-ATTTGTATTCATTTATATTATAGGTTTCTATATCATATACT  249

Query  291  --t--ttttcccaatcttttatgaagtttt-atttACAATTTAATTTCAATCGAAAATAG  345
              |  |||||| |||||||||||||||||  |||||||||||| |||||| ||||||||
Sbjct  248  TTTACTTTTCCTAATCTTTTATGAAGTTTTTTTTACAATTTAAATTCAATTGAAAATAG  189

Query  346  AGAAAAATGACAATTTTGCTT--TT-TGATTTATATTGATTAAATCAAAATAAGAAAGAA  402
            | |||||||| ||||||||||  || | | || |||| ||| ||| ||||| ||||||||
Sbjct  188  ATAAAAATGAAAATTTTGCTTATTTATTACTTTTATTTATTCAATAAAAATGAGAAAGAA  129

Query  403  TATGCtttttttATGTTAATGGAAAAATATAGTAATACTAGATAATCGTAGAGGGGCGGA  462
            ||||||||||| |||||||||||||||||||||||||||||||||||| |||||||||||||
Sbjct  128  TATGCTTTTTT-ATGTTAATGGAAAAATATAGTAATACTAGATAATAGTAGAGGGGCGGA  70

Query  463  TGTAGCCAAGTGGATCAAGGCAGTGGATTGTGAATCCACCATGCGCGG  510
            ||||||||||||||||||||||||||||||||||||||||||||||||
Sbjct  69   TGTAGCCAAGTGGATCAAGGCAGTGGATTGTGAATCCACCATGCGCGG  22


>KF746351.1 Helianthus grosseserratus isolate DB06 plastid, complete genome
Length=151052

 Score = 706 bits (382),  Expect = 0.0
 Identities = 483/528 (91%), Gaps = 22/528 (4%)
 Strand=Plus/Minus

Query  2    GTTATGCATGAACGTAATGCTCATAATTTCCCTCTAGACTTAGCTGCTATTGAAGCTCCA  61
            ||||||||||||||||||||||||||||||||||||||||||||||||||||||||||||
Sbjct  546  GTTATGCATGAACGTAATGCTCATAATTTCCCTCTAGACTTAGCTGCTATTGAAGCTCCA  487

Query  62   TATACAAATGGATAAGCCTTTGGTCTGATTGTATAGGAGTTTTTGAACTAAAAAAGGAGC  121
            | |||||||||||||| |||||||||||||||||||||||||||||||||||||||||||
Sbjct  486  TCTACAAATGGATAAGACTTTGGTCTGATTGTATAGGAGTTTTTGAACTAAAAAAGGAGC  427

Query  122  AATAAC-GCCCTCTTGATAAAACAAGAGGGAAGCTATTGCTCCtttttttttATTTAGTAC  180
            |||| |  ||||||||||||||||||||||  | |||||||||||||||||||||||||||
```

```
Sbjct  426   AATAGCTTCCCTCTTGATAAAACAAGAGGG-CGTTATTGCTCCTTTTTTTTATTTAGTAC  368

Query  181   TATTTGCCTTACATAGTTTCTTTAAA-------AATAACAAGTACTTTTTATAGTTTGGT  233
             ||||||||||||||||||||||||||       |||||||||||||||||||||||||||
Sbjct  367   TATTTGCCTTACATAGTTTCTTTAAACTTTAAAAATAACAAGTACTTTTTATAGTTTGGT  308

Query  234   TCGATTTGCGTGtttttctttgtattcatttatattataggtttctatatcatata---  290
             ||||||||  |||||||  ||||||||||||||||||||||||||||||||||||||
Sbjct  307   TCGATTTGTTTGTTTTT-ATTTGTATTCATTTATATTATAGGTTTCTATATCATATACTT  249

Query  291   -t--ttttcccaatcttttatgaagtttt--atttACAATTTAATTTCAATCGAAAATAG  345
              |  |||||| ||||||||||||||||||   |||||||||||| |||||| ||||||||
Sbjct  248   TTACTTTTCCTAATCTTTTATGAAGTTTTTTTTTACAATTTAAATTCAATTGAAAATAG  189

Query  346   AGAAAAATGACAATTTTGCTT--TT-TGATTTATATTGATTAAATCAAAATAAGAAAGAA  402
             | |||||||| ||||||||||  || | | || |||| ||| ||| ||||| ||||||||
Sbjct  188   ATAAAAATGAAAATTTTGCTTATTTATTACTTTTATTTATTCAATAAAAATGAGAAAGAA  129

Query  403   TATGCtttttttATGTTAATGGAAAAATATAGTAATACTAGATAATCGTAGAGGGGCGGA  462
             ||||||||||| |||||||||||||||||||||||||||||||||| |||||||||||||
Sbjct  128   TATGCTTTTTT-ATGTTAATGGAAAAATATAGTAATACTAGATAATAGTAGAGGGGCGGA  70

Query  463   TGTAGCCAAGTGGATCAAGGCAGTGGATTGTGAATCCACCATGCGCGG  510
             ||||||||||||||||||||||||||||||||||||||||||||||||
Sbjct  69    TGTAGCCAAGTGGATCAAGGCAGTGGATTGTGAATCCACCATGCGCGG  22


>MF037492.1 Echinacea pallida voucher Rebman1053 trnH-psbA intergenic spacer
region, partial sequence; chloroplast
Length=405

 Score = 706 bits (382),  Expect = 0.0
 Identities = 399/407 (98%), Gaps = 2/407 (0%)
 Strand=Plus/Plus

Query  45    CTGCTATTGAAGCTCCATATACAAATGGATAAGCCTTTGGTCTGATTGTATAGGAGTTTT  104
             |||||||||||||||||| |||||||||||||||||||||||||||||||||||||||||
Sbjct  1     CTGCTATTGAAGCTCCATCTACAAATGGATAAGCCTTTGGTCTGATTGTATAGGAGTTTT  60

Query  105   TGAACTAAAAAAGGAGCAATAACGCCCTCTTGATAAAACAAGAGGGAAGCTATTGCTCCt  164
             |||||||||||||||||||||||||||||||| |  | |||||||||||||||||||||
Sbjct  61    TGAACTAAAAAAGGAGCAATAACGCCCTCTTGTTTTATCAAGAGGGAAGCTATTGCTCC-  119

Query  165   tttttttATTTAGTACTATTTGCCTTACATAGTTTCTTTAAAAATAACAAGTACTTTTTA  224
             ||||||||||||||||||||||||||||||||||||||||||||||||||||||||||||
Sbjct  120   TTTTTTTATTTAGTACTATTTGCCTTACATAGTTTCTTTAAAAATAACAAGTACTTTTTA  179

Query  225   TAGTTTGGTTCGATTTGCGTGtttttctttgtattcatttatattataggtttctatat  284
             ||||||||||||||||||||||||||||||||||||||||||||||||||||||||||||
Sbjct  180   TAGTTTGGTTCGATTTGCGTGTTTTTTCTTTGTATTCATTTATATTATAGGTTTCTATAT  239

Query  285   catatatttttcccaatcttttatgaagttttatttACAATTTAATTTCAATCGAAAATA  344
             ||||||||||||||||||||||||||||||||||||||||||||||||||||||||||||
Sbjct  240   CATATATTTTTCCCAATCTTTTATGAAGTTTTATTTACAATTTAATTTCAATCGAAAATA  299

Query  345   GAGAAAAATGACAATTTTGCTTTTTGATTTATATTGATTAAATCAAAATAAGAAAGAATA  404
             |||||||||||||||||||||||| |||||||||||||||||||||||||||||||||||
```

```
Sbjct  300  GAGAAAAATGACAATTTTGCTTTTTTATTTATATTGATTAAATCAAAATAAGAAAGAATA  359

Query  405  TGCtttttttATGTTAATGGAAAAATATAGTAATACTAGATAATCGT  451
            ||| |||||||||||||||||||||||||||||||||||||||||||
Sbjct  360  TGC-TTTTTTATGTTAATGGAAAAATATAGTAATACTAGATAATCGT  405
```

>KF746383.1 Helianthus maximiliani isolate MAX17 plastid, complete genome
Length=151076

Score = 706 bits (382), Expect = 0.0
Identities = 483/528 (91%), Gaps = 22/528 (4%)
Strand=Plus/Minus

```
Query  2    GTTATGCATGAACGTAATGCTCATAATTTCCCTCTAGACTTAGCTGCTATTGAAGCTCCA  61
            ||||||||||||||||||||||||||||||||||||||||||||||||||||||||||||
Sbjct  546  GTTATGCATGAACGTAATGCTCATAATTTCCCTCTAGACTTAGCTGCTATTGAAGCTCCA  487

Query  62   TATACAAATGGATAAGCCTTTGGTCTGATTGTATAGGAGTTTTTGAACTAAAAAAGGAGC  121
            | |||||||||||||| |||||||||||||||||||||||||||||||||||||||||||
Sbjct  486  TCTACAAATGGATAAGACTTTGGTCTGATTGTATAGGAGTTTTTGAACTAAAAAAGGAGC  427

Query  122  AATAAC-GCCCTCTTGATAAAACAAGAGGGAAGCTATTGCTCCttttttttATTTAGTAC  180
            |||| |  ||||||||||||||||||||||  | ||||||||||||||||||||||||||
Sbjct  426  AATAGCTTCCCTCTTGATAAAACAAGAGGG-CGTTATTGCTCCTTTTTTTTATTTAGTAC  368

Query  181  TATTTGCCTTACATAGTTTCTTTAAA-------AATAACAAGTACTTTTTATAGTTTGGT  233
            ||||||||||||||||||||||||||       |||||||||||||||||||||||||||
Sbjct  367  TATTTGCCTTACATAGTTTCTTTAAACTTTAAAAATAACAAGTACTTTTTATAGTTTGGT  308

Query  234  TCGATTTGCGTGttttttctttgtattcatttatattataggtttctatatcatata---  290
            ||||||||  |||||||  ||||||||||||||||||||||||||||||||||||||
Sbjct  307  TCGATTTGTTTGTTTTT-ATTTGTATTCATTTATATTATAGGTTTCTATATCATATACTT  249

Query  291  -t--ttttcccaatcttttatgaagtttt--atttACAATTTAATTTCAATCGAAAATAG  345
             |  |||||| ||||||||||||||||||   |||||||||||| |||||| ||||||||
Sbjct  248  TTACTTTTCCTAATCTTTTATGAAGTTTTTTTTTTACAATTTAAATTCAATTGAAAATAG  189

Query  346  AGAAAAATGACAATTTTGCTT--TT-TGATTTATATTGATTAAATCAAAATAAGAAAGAA  402
            | |||||||| ||||||||||  || | | || |||| ||| ||| ||||| ||||||||
Sbjct  188  ATAAAAATGAAAATTTTGCTTATTTATTACTTTATTTATTCAATAAAAATGAGAAAGAA  129

Query  403  TATGCtttttttATGTTAATGGAAAAATATAGTAATACTAGATAATCGTAGAGGGGCGGA  462
            ||||||||||| |||||||||||||||||||||||||||||||||| |||||||||||||
Sbjct  128  TATGCTTTTTT-ATGTTAATGGAAAAATATAGTAATACTAGATAATAGTAGAGGGGCGGA  70

Query  463  TGTAGCCAAGTGGATCAAGGCAGTGGATTGTGAATCCACCATGCGCGG  510
            ||||||||||||||||||||||||||||||||||||||||||||||||
Sbjct  69   TGTAGCCAAGTGGATCAAGGCAGTGGATTGTGAATCCACCATGCGCGG  22
```

>MK341450.1 Helianthus annuus cultivar HA89(MAX1) chloroplast, complete genome
Length=151255

Score = 706 bits (382), Expect = 0.0
Identities = 483/528 (91%), Gaps = 22/528 (4%)
Strand=Plus/Minus

```
Query  2    GTTATGCATGAACGTAATGCTCATAATTTCCCTCTAGACTTAGCTGCTATTGAAGCTCCA  61
            ||||||||||||||||||||||||||||||||||||||||||||||||||||||||||||
Sbjct  545  GTTATGCATGAACGTAATGCTCATAATTTCCCTCTAGACTTAGCTGCTATTGAAGCTCCA  486

Query  62   TATACAAATGGATAAGCCTTTGGTCTGATTGTATAGGAGTTTTTGAACTAAAAAAGGAGC  121
            | |||||||||||||| |||||||||||||||||||||||||||||||||||||||||||
Sbjct  485  TCTACAAATGGATAAGACTTTGGTCTGATTGTATAGGAGTTTTTGAACTAAAAAAGGAGC  426

Query  122  AATAAC-GCCCTCTTGATAAAACAAGAGGGAAGCTATTGCTCCtttttttt-ATTTAGTA  179
            |||| |  ||||||||||||||||||||||  | ||||||||||||||||| ||||||||
Sbjct  425  AATAGCTTCCCTCTTGATAAAACAAGAGGG-CGTTATTGCTCCTTTTTTTTTATTTAGTA  367

Query  180  CTATTTGCCTTACATAGTTTCTTTAAA-------AATAACAAGTACTTTTTATAGTTTGG  232
            |||||||||||||||||||||||||||       ||||||||||||||||||||||||||
Sbjct  366  CTATTTGCCTTACATAGTTTCTTTAAACTTTAAAAATAACAAGTACTTTTTATAGTTTGG  307

Query  233  TTCGATTTGCGTGttttttctttgtattcatttatattataggtttctatatcatata--  290
            |||||||||  |||||||  ||||||||||||||||||||||||||||||||||||||
Sbjct  306  TTCGATTTGTTTGTTTTT-ATTTGTATTCATTTATATTATAGGTTTCTATATCATATACT  248

Query  291  --t--ttttcccaatcttttatgaagtttt-atttACAATTTAATTTCAATCGAAAATAG  345
              |  |||||| ||||||||||||||||||  |||||||||||| |||||| ||||||||
Sbjct  247  TTTACTTTTCCTAATCTTTTATGAAGTTTTTTTTTACAATTTAAATTCAATTGAAAATAG  188

Query  346  AGAAAAATGACAATTTTGCTT--TT-TGATTTATATTGATTAAATCAAAATAAGAAAGAA  402
            | |||||||| ||||||||||  || | | || |||| ||| ||| ||||| ||||||||
Sbjct  187  ATAAAAATGAAAATTTTGCTTATTTATTACTTTTATTTATTCAATAAAAATGAGAAAGAA  128

Query  403  TATGCtttttttATGTTAATGGAAAAATATAGTAATACTAGATAATCGTAGAGGGGCGGA  462
            ||||||||||| |||||||||||||||||||||||||||||||||| |||||||||||||
Sbjct  127  TATGCTTTTTT-ATGTTAATGGAAAAATATAGTAATACTAGATAATAGTAGAGGGGCGGA  69

Query  463  TGTAGCCAAGTGGATCAAGGCAGTGGATTGTGAATCCACCATGCGCGG  510
            ||||||||||||||||||||||||||||||||||||||||||||||||
Sbjct  68   TGTAGCCAAGTGGATCAAGGCAGTGGATTGTGAATCCACCATGCGCGG  21


>MT302566.1 Helianthus hirsutus chloroplast, complete genome
Length=151272

 Score = 706 bits (382),  Expect = 0.0
 Identities = 483/528 (91%), Gaps = 22/528 (4%)
 Strand=Plus/Minus

Query  2    GTTATGCATGAACGTAATGCTCATAATTTCCCTCTAGACTTAGCTGCTATTGAAGCTCCA  61
            ||||||||||||||||||||||||||||||||||||||||||||||||||||||||||||
Sbjct  545  GTTATGCATGAACGTAATGCTCATAATTTCCCTCTAGACTTAGCTGCTATTGAAGCTCCA  486

Query  62   TATACAAATGGATAAGCCTTTGGTCTGATTGTATAGGAGTTTTTGAACTAAAAAAGGAGC  121
            | |||||||||||||| |||||||||||||||||||||||||||||||||||||||||||
Sbjct  485  TCTACAAATGGATAAGACTTTGGTCTGATTGTATAGGAGTTTTTGAACTAAAAAAGGAGC  426

Query  122  AATAAC-GCCCTCTTGATAAAACAAGAGGGAAGCTATTGCTCCtttttttt-ATTTAGTA  179
            |||| |  ||||||||||||||||||||||  | ||||||||||||||||| ||||||||
Sbjct  425  AATAGCTTCCCTCTTGATAAAACAAGAGGG-CGTTATTGCTCCTTTTTTTTTATTTAGTA  367
```

```
Query  180  CTATTTGCCTTACATAGTTTCTTTAAA-------AATAACAAGTACTTTTTATAGTTTGG  232
            |||||||||||||||||||||||||||       ||||||||||||||||||||||||||
Sbjct  366  CTATTTGCCTTACATAGTTTCTTTAAACTTTAAAAATAACAAGTACTTTTTATAGTTTGG  307

Query  233  TTCGATTTGCGTGtttttctttgtattcatttatattataggtttctatatcatata--  290
            |||||||||  |||||||  ||||||||||||||||||||||||||||||||||||||
Sbjct  306  TTCGATTTGTTTGTTTTT-ATTTGTATTCATTTATATTATAGGTTTCTATATCATATACT  248

Query  291  --t--ttttcccaatcttttatgaagtttt-atttACAATTTAATTTCAATCGAAAATAG  345
              |  |||||| ||||||||||||||||||  |||||||||||| |||||| ||||||||
Sbjct  247  TTTACTTTTCCTAATCTTTTATGAAGTTTTTTTTTACAATTTAAATTCAATTGAAAATAG  188

Query  346  AGAAAAATGACAATTTTGCTT--TT-TGATTTATATTGATTAAATCAAAATAAGAAAGAA  402
            | |||||||| ||||||||||  || | | || |||| ||| ||| ||||| ||||||||
Sbjct  187  ATAAAAATGAAAATTTTGCTTATTTATTACTTTTATTTATTCAATAAAAATGAGAAAGAA  128

Query  403  TATGCtttttttATGTTAATGGAAAAATATAGTAATACTAGATAATCGTAGAGGGGCGGA  462
            ||||||||||| |||||||||||||||||||||||||||||||||| |||||||||||||
Sbjct  127  TATGCTTTTTT-ATGTTAATGGAAAAATATAGTAATACTAGATAATAGTAGAGGGGCGGA  69

Query  463  TGTAGCCAAGTGGATCAAGGCAGTGGATTGTGAATCCACCATGCGCGG  510
            ||||||||||||||||||||||||||||||||||||||||||||||||
Sbjct  68   TGTAGCCAAGTGGATCAAGGCAGTGGATTGTGAATCCACCATGCGCGG  21


>KF746368.1 Helianthus grosseserratus isolate DB23 plastid, complete genome
Length=151010

 Score = 706 bits (382),  Expect = 0.0
 Identities = 483/528 (91%), Gaps = 22/528 (4%)
 Strand=Plus/Minus

Query  2    GTTATGCATGAACGTAATGCTCATAATTTCCCTCTAGACTTAGCTGCTATTGAAGCTCCA  61
            ||||||||||||||||||||||||||||||||||||||||||||||||||||||||||||
Sbjct  546  GTTATGCATGAACGTAATGCTCATAATTTCCCTCTAGACTTAGCTGCTATTGAAGCTCCA  487

Query  62   TATACAAATGGATAAGCCTTTGGTCTGATTGTATAGGAGTTTTTGAACTAAAAAAGGAGC  121
            | |||||||||||||| |||||||||||||||||||||||||||||||||||||||||||
Sbjct  486  TCTACAAATGGATAAGACTTTGGTCTGATTGTATAGGAGTTTTTGAACTAAAAAAGGAGC  427

Query  122  AATAAC-GCCCTCTTGATAAAACAAGAGGGAAGCTATTGCTCCtttttttt-ATTTAGTA  179
            |||| |  ||||||||||||||||||||||  | ||||||||||||||||| ||||||||
Sbjct  426  AATAGCTTCCCTCTTGATAAAACAAGAGGG-CGTTATTGCTCCTTTTTTTTTATTTAGTA  368

Query  180  CTATTTGCCTTACATAGTTTCTTTAAA-------AATAACAAGTACTTTTTATAGTTTGG  232
            |||||||||||||||||||||||||||       ||||||||||||||||||||||||||
Sbjct  367  CTATTTGCCTTACATAGTTTCTTTAAACTTTAAAAATAACAAGTACTTTTTATAGTTTGG  308

Query  233  TTCGATTTGCGTGtttttctttgtattcatttatattataggtttctatatcatata--  290
            |||||||||  |||||||  ||||||||||||||||||||||||||||||||||||||
Sbjct  307  TTCGATTTGTTTGTTTTT-ATTTGTATTCATTTATATTATAGGTTTCTATATCATATACT  249

Query  291  --t--ttttcccaatcttttatgaagtttt-atttACAATTTAATTTCAATCGAAAATAG  345
              |  |||||| ||||||||||||||||||  |||||||||||| |||||| ||||||||
Sbjct  248  TTTACTTTTCCTAATCTTTTATGAAGTTTTTTTTTACAATTTAAATTCAATTGAAAATAG  189

Query  346  AGAAAAATGACAATTTTGCTT--TT-TGATTTATATTGATTAAATCAAAATAAGAAAGAA  402
```

```
            | |||||||| ||||||||||  || | | || |||| ||| ||| ||||| ||||||||
Sbjct  188  ATAAAAATGAAAATTTTGCTTATTTATTACTTTTATTTATTCAATAAAAATGAGAAAGAA  129

Query  403  TATGCtttttttATGTTAATGGAAAAATATAGTAATACTAGATAATCGTAGAGGGGCGGA  462
            ||||||||||| |||||||||||||||||||||||||||||||||| |||||||||||||
Sbjct  128  TATGCTTTTTT-ATGTTAATGGAAAAATATAGTAATACTAGATAATAGTAGAGGGGCGGA  70

Query  463  TGTAGCCAAGTGGATCAAGGCAGTGGATTGTGAATCCACCATGCGCGG  510
            ||||||||||||||||||||||||||||||||||||||||||||||||
Sbjct  69   TGTAGCCAAGTGGATCAAGGCAGTGGATTGTGAATCCACCATGCGCGG  22


>KF746379.1 Helianthus tuberosus isolate DB34 plastid, complete genome
Length=151045

 Score = 706 bits (382),  Expect = 0.0
 Identities = 483/528 (91%), Gaps = 22/528 (4%)
 Strand=Plus/Minus

Query  2    GTTATGCATGAACGTAATGCTCATAATTTCCCTCTAGACTTAGCTGCTATTGAAGCTCCA  61
            ||||||||||||||||||||||||||||||||||||||||||||||||||||||||||||
Sbjct  546  GTTATGCATGAACGTAATGCTCATAATTTCCCTCTAGACTTAGCTGCTATTGAAGCTCCA  487

Query  62   TATACAAATGGATAAGCCTTTGGTCTGATTGTATAGGAGTTTTTGAACTAAAAAAGGAGC  121
            | |||||||||||||| |||||||||||||||||||||||||||||||||||||||||||
Sbjct  486  TCTACAAATGGATAAGACTTTGGTCTGATTGTATAGGAGTTTTTGAACTAAAAAAGGAGC  427

Query  122  AATAAC-GCCCTCTTGATAAAACAAGAGGGAAGCTATTGCTCCtttttttt-ATTTAGTA  179
            |||| |  ||||||||||||||||||||||  | ||||||||||||||||| ||||||||
Sbjct  426  AATAGCTTCCCTCTTGATAAAACAAGAGGG-CGTTATTGCTCCTTTTTTTTTATTTAGTA  368

Query  180  CTATTTGCCTTACATAGTTTCTTTAAA-------AATAACAAGTACTTTTTATAGTTTGG  232
            |||||||||||||||||||||||||||       ||||||||||||||||||||||||||
Sbjct  367  CTATTTGCCTTACATAGTTTCTTTAAACTTTAAAAATAACAAGTACTTTTTATAGTTTGG  308

Query  233  TTCGATTTGCGTGttttttctttgtattcatttatattataggtttctatatcatata--  290
            |||||||||  |||||||  ||||||||||||||||||||||||||||||||||||||
Sbjct  307  TTCGATTTGTTTGTTTTT-ATTTGTATTCATTTATATTATAGGTTTCTATATCATATACT  249

Query  291  --t--ttttcccaatcttttatgaagtttt-atttACAATTTAATTTCAATCGAAAATAG  345
              |  |||||| ||||||||||||||||||  |||||||||||| |||||| ||||||||
Sbjct  248  TTTACTTTTCCTAATCTTTTATGAAGTTTTTTTTTACAATTTAAATTCAATTGAAAATAG  189

Query  346  AGAAAAATGACAATTTTGCTT--TT-TGATTTATATTGATTAAATCAAAATAAGAAAGAA  402
            | |||||||| ||||||||||  || | | || |||| ||| ||| ||||| ||||||||
Sbjct  188  ATAAAAATGAAAATTTTGCTTATTTATTACTTTTATTTATTCAATAAAAATGAGAAAGAA  129

Query  403  TATGCtttttttATGTTAATGGAAAAATATAGTAATACTAGATAATCGTAGAGGGGCGGA  462
            ||||||||||| |||||||||||||||||||||||||||||||||| |||||||||||||
Sbjct  128  TATGCTTTTTT-ATGTTAATGGAAAAATATAGTAATACTAGATAATAGTAGAGGGGCGGA  70

Query  463  TGTAGCCAAGTGGATCAAGGCAGTGGATTGTGAATCCACCATGCGCGG  510
            ||||||||||||||||||||||||||||||||||||||||||||||||
Sbjct  69   TGTAGCCAAGTGGATCAAGGCAGTGGATTGTGAATCCACCATGCGCGG  22


>MF037490.1 Echinacea angustifolia voucher Rolfsmeier19250 trnH-psbA intergenic
```

```
spacer region, partial sequence; chloroplast
Length=409

 Score = 704 bits (381),  Expect = 0.0
 Identities = 402/411 (98%), Gaps = 6/411 (1%)
 Strand=Plus/Plus

Query  45   CTGCTATTGAAGCTCCATATACAAATGGATAAGCCTTTGGTCTGATTGTATAGGAGTTTT  104
            |||||||||||||||||| |||||||||||||||||||||||||||||||||||||||||
Sbjct  1    CTGCTATTGAAGCTCCATCTACAAATGGATAAGCCTTTGGTCTGATTGTATAGGAGTTTT  60

Query  105  TGAACTAAAAAAGGAGCAATAACGCCCTCTTGATAAAACAAGAGGGAAGCTATTGCTCCt  164
            ||||||||||||||||||||||||||||||||||||||||||||||||||||||||||| 
Sbjct  61   TGAACTAAAAAAGGAGCAATAACGCCCTCTTGATAAAACAAGAGGGAAGCTATTGCTCC-  119

Query  165  tttttttATTTAGTACTATTTGCCTTACATAGTTTCTTTAAAAATAACAAGTACTTTTTA  224
            ||||||||||||||||||||||||||||||||||||||||||||||||||||||||||||
Sbjct  120  TTTTTTTATTTAGTACTATTTGCCTTACATAGTTTCTTTAAAAATAACAAGTACTTTTTA  179

Query  225  TAGTTTGGTTCGATTTGCGTGtttttctttgtattcatttatattataggtttctatat  284
            ||||||||||||||||||||||||||||||||||||||||||||||||||||||||||||
Sbjct  180  TAGTTTGGTTCGATTTGCGTGTTTTTTCTTTGTATTCATTTATATTATAGGTTTCTATAT  239

Query  285  catatatttttcccaatctttatgaagttttatttAC-AATTTAATTTCAATCGAAAAT  343
            ||||||||||||||||||||||||||||||||||||| ||||||||||||||||||||||
Sbjct  240  CATATATTTTTCCCAATCTTTATGAAGTTTTATTTACGAATTTAATTTCAATCGAAAAT  299

Query  344  AGAGAAAAATGAC-AATTTTGCTTTTTGATTTATATTGATTAAATCAAAA-TAAG-AAAG  400
            ||||||||||||| ||||||||||||| |||||||||||||||||||||| |||| ||||
Sbjct  300  AGAGAAAAATGACGAATTTTGCTTTTTTATTTATATTGATTAAATCAAAAATAAGAAAAG  359

Query  401  AATATGCtttttttATGTTAATGGAAAAATATAGTAATACTAGATAATCGT  451
            ||||||| |||||||||| ||||||||||||||||||||||||||||||||
Sbjct  360  AATATGC-TTTTTTATGTGAATGGAAAAATATAGTAATACTAGATAATCGT  409


>NC_053298.1 Aldama trichophylla voucher Magenta 390 chloroplast, complete
genome
 MN337911.1 Aldama trichophylla chloroplast, complete genome
Length=151353

 Score = 704 bits (381),  Expect = 0.0
 Identities = 484/530 (91%), Gaps = 22/530 (4%)
 Strand=Plus/Minus

Query  2    GTTATGCATGAACGTAATGCTCATAATTTCCCTCTAGACTTAGCTGCTATTGAAGCTCCA  61
            ||||||||||||||||||||||||||||||||||||||||||||||||||||||||||||
Sbjct  549  GTTATGCATGAACGTAATGCTCATAATTTCCCTCTAGACTTAGCTGCTATTGAAGCTCCA  490

Query  62   TATACAAATGGATAAGCCTTTGGTCTGATTGTATAGGAGTTTTTGAACTAAAAAAGGAGC  121
            | |||||||||||||| |||||||||||||||||||||||||||||||||||||||||||
Sbjct  489  TCTACAAATGGATAAGACTTTGGTCTGATTGTATAGGAGTTTTTGAACTAAAAAAGGAGC  430

Query  122  AATAACGCCCTCTTGATAAAACAAGAGGGAAGCTATTGCTCCttttttttATTTAGTACT  181
            ||||||||||||||| |  | |||||||||||||||||||||||||||||||||||||||
Sbjct  429  AATAACGCCCTCTTGTTTTATCAAGAGGGAAGCTATTGCTCCTTTTTTTTATTTAGTACT  370
```

```
Query  182   ATTTGCCTTACATAGTTTCTTTAAA-------AATAACAAGTACTTTTTATAGTTTGGTT  234
             |||||||||||||||||||||||||       ||||||||||||||||||||||||||||
Sbjct  369   ATTTGCCTTACATAGTTTCTTTAAACTTTAAAAATAACAAGTACTTTTTATAGTTTGGTT  310

Query  235   CGATTTGCGTGtttttctttgtattcatttatattataggtttctatatcatatatttt  294
             |||||||  |||||||| |||||||||||||||||||||||||||||||||||||||| |||
Sbjct  309   CGATTTGTTTGTTTTTTATTTGTATTCATTTATATTATAGGTTTCTATATCATATACTTT  250

Query  295   t-c----cc-aatcttttatgaagttttattt-----ACAATTTAATTTCAATCGAAAAT  343
             | |    || |||||||||||||||||| |||     ||||| ||| |||||||||||||
Sbjct  249   TACTTTTCCTAATCTTTTATGAAGTTTTTTTTTTTTTACAATCTAAATTCAATCGAAAAT  190

Query  344   AGAGAAAAATGACAATTTTGCTTTTTGATT--TA-TATTGATTAAATCAAAATAAGAAAG  400
             ||| |||||||||||||||| || || |||  |  |||| ||| ||| ||||| ||||||
Sbjct  189   AGATAAAAATGACAATTTTGTTTATTTATTACTTTTATTTATTCAATAAAAATGAGAAAG  130

Query  401   AATATGCtttttttATGTTAATGGAAAAATATAGTAATACTAGATAATCGTAGAGGGGCG  460
             ||||||||||||| |||||||||| |||||||||||||||||||||| |||||||||||
Sbjct  129   AATATGCTTTTTT-ATGTTAATGGGAAAATATAGTAATACTAGATAATAGTAGAGGGGCG  71

Query  461   GATGTAGCCAAGTGGATCAAGGCAGTGGATTGTGAATCCACCATGCGCGG  510
             ||||||||||||||||||||||||||||||||||||||||||||||||||
Sbjct  70    GATGTAGCCAAGTGGATCAAGGCAGTGGATTGTGAATCCACCATGCGCGG  21


>MT793836.1 Mikania glomerata chloroplast, complete genome
Length=151773

 Score = 702 bits (380),  Expect = 0.0
 Identities = 477/521 (92%), Gaps = 17/521 (3%)
 Strand=Plus/Minus

Query  2     GTTATGCATGAACGTAATGCTCATAATTTCCCTCTAGACTTAGCTGCTATTGAAGCTCCA  61
             ||||||||||||||||||||||||||||||||||||||||||||||||||||||||||||
Sbjct  536   GTTATGCATGAACGTAATGCTCATAATTTCCCTCTAGACTTAGCTGCTATTGAAGCTCCA  477

Query  62    TATACAAATGGATAAGCCTTTGGTCTGATTGTATAGGAGTTTTTGAACTAAAAAAGGAGC  121
             | |||||||||||||| |||||||||||||||||||||||||||||||||||||||||||
Sbjct  476   TCTACAAATGGATAAGACTTTGGTCTGATTGTATAGGAGTTTTTGAACTAAAAAAGGAGC  417

Query  122   AATAAC-GCCCTCTTGATAAAACAAGAGGGAAGCTATTGCTCCttttttttATTTAGTAC  180
             |||| |  |||||||| |  | ||||||||  | ||||||||||||||| |||||||||
Sbjct  416   AATAGCTTCCCTCTTGTTTTATCAAGAGGG-CGTTATTGCTCCTTTTTTT-ATTTAGTAC  359

Query  181   TATTTGCCTTACATAGTTTCTTTAAAAATAAC-----------AAGTACTTTTTATAGTT  229
             ||||||||||||||||||||||||||||||||           ||| |||||||||||||
Sbjct  358   TATTTGCCTTACATAGTTTCTTTAAAAATAACTTAAAAATAACAAGGACTTTTTATAGTT  299

Query  230   TGGTTCGATTTGCGTGtttttctttgtattcatttatattataggtttctatatcatat  289
             ||||||||||||||||||||||||||||||||||||||||||||||||||||||| ||||
Sbjct  298   TGGTTCGATTTGCGTGTTTTTTCTTTGTATTCATTTATATTATAGGTTTCTATATAATAT  239

Query  290   atttttcccaatcttttatgaagttttatttACAATTTAATTTCAATCGAAAATAGAGAA  349
             |||||||||||||||||||||||||| |||| ||||| ||||||||||||||||||  |
Sbjct  238   ATTTTTCCCAATCTTTTATGAAGTTTGATTTCCAATTCAATTTCAATCGAAAATAGATCA  179

Query  350   AAATGACAATTTTGCTTTTTGATTTATATTGATTAAATCAAAATAAGAAAGAATATGCtt  409
```

```
            ||||||||||||||||| || ||| |  || ||| | |  |||||||||| |||||||||
Sbjct  178  AAATGACAATTTTGCTTATTTATT-ACTTTTATTTATT--AAATAAGAAATAATATGCTT  122

Query  410  tttttATGTTAATGGAAAAATATAGTAATACTAGATAATCGTAGAGGGGCGGATGTAGCC  469
            |||| |||||||||||||||||||||||||||||||||| ||||||||||||||||||||
Sbjct  121  TTTTGATGTTAATGGAAAAATATAGTAATACTAGATAATAGTAGAGGGGCGGATGTAGCC  62

Query  470  AAGTGGATCAAGGCAGTGGATTGTGAATCCACCATGCGCGG  510
            |||||||||||||||||||||||||||||||||||||||||
Sbjct  61   AAGTGGATCAAGGCAGTGGATTGTGAATCCACCATGCGCGG  21


 Score = 110 bits (59),  Expect = 3e-19
 Identities = 59/59 (100%), Gaps = 0/59 (0%)
 Strand=Plus/Plus

Query  111  AAAAAAGGAGCAATAACGCCCTCTTGATAAAACAAGAGGGAAGCTATTGCTCCtttttt  169
            |||||||||||||||||||||||||||||||||||||||||||||||||||||||||||
Sbjct  369  AAAAAAGGAGCAATAACGCCCTCTTGATAAAACAAGAGGGAAGCTATTGCTCCTTTTTT  427


>MT793840.1 Mikania oblongifolia chloroplast, complete genome
Length=151845

 Score = 702 bits (380),  Expect = 0.0
 Identities = 477/521 (92%), Gaps = 17/521 (3%)
 Strand=Plus/Minus

Query  2    GTTATGCATGAACGTAATGCTCATAATTTCCCTCTAGACTTAGCTGCTATTGAAGCTCCA  61
            ||||||||||||||||||||||||||||||||||||||||||||||||||||||||||||
Sbjct  536  GTTATGCATGAACGTAATGCTCATAATTTCCCTCTAGACTTAGCTGCTATTGAAGCTCCA  477

Query  62   TATACAAATGGATAAGCCTTTGGTCTGATTGTATAGGAGTTTTTGAACTAAAAAAGGAGC  121
            | |||||||||||||| |||||||||||||||||||||||||||||||||||||||||||
Sbjct  476  TCTACAAATGGATAAGACTTTGGTCTGATTGTATAGGAGTTTTTGAACTAAAAAAGGAGC  417

Query  122  AATAAC-GCCCTCTTGATAAAACAAGAGGGAAGCTATTGCTCCttttttttATTTAGTAC  180
            |||| |  |||||||| |  | ||||||||  | |||||||||||||||| |||||||||
Sbjct  416  AATAGCTTCCCTCTTGTTTTATCAAGAGGG-CGTTATTGCTCCTTTTTTT-ATTTAGTAC  359

Query  181  TATTTGCCTTACATAGTTTCTTTAAAAATAAC-----------AAGTACTTTTTATAGTT  229
            ||||||||||||||||||||||||||||||||           ||| |||||||||||||
Sbjct  358  TATTTGCCTTACATAGTTTCTTTAAAAATAACTTAAAAATAACAAGGACTTTTTATAGTT  299

Query  230  TGGTTCGATTTGCGTGttttttctttgtattcatttatattataggtttctatatcatat  289
            ||||||||||||||||||||||||||||||||||||||||||||||||||||||| ||||
Sbjct  298  TGGTTCGATTTGCGTGTTTTTTCTTTGTATTCATTTATATTATAGGTTTCTATATAATAT  239

Query  290  atttttcccaatcttttatgaagttttatttACAATTTAATTTCAATCGAAAATAGAGAA  349
            |||||||||||||||||||||||||| |||| ||||| |||||||||||||||||||  |
Sbjct  238  ATTTTTCCCAATCTTTTATGAAGTTTGATTTCCAATTCAATTTCAATCGAAAATAGATCA  179

Query  350  AAATGACAATTTTGCTTTTTGATTTATATTGATTAAATCAAAATAAGAAAGAATATGCtt  409
            ||||||||||||||||| || ||| |  || ||| | |  |||||||||| |||||||||
Sbjct  178  AAATGACAATTTTGCTTATTTATT-ACTTTTATTTATT--AAATAAGAAATAATATGCTT  122

Query  410  tttttATGTTAATGGAAAAATATAGTAATACTAGATAATCGTAGAGGGGCGGATGTAGCC  469
```

```
               |||| |||||||||||||||||||||||||||||||||| ||||||||||||||||||||
Sbjct  121  TTTTGATGTTAATGGAAAAATATAGTAATACTAGATAATAGTAGAGGGGCGGATGTAGCC  62

Query  470  AAGTGGATCAAGGCAGTGGATTGTGAATCCACCATGCGCGG  510
            |||||||||||||||||||||||||||||||||||||||||
Sbjct  61   AAGTGGATCAAGGCAGTGGATTGTGAATCCACCATGCGCGG  21


 Score = 110 bits (59),  Expect = 3e-19
 Identities = 59/59 (100%), Gaps = 0/59 (0%)
 Strand=Plus/Plus

Query  111  AAAAAAGGAGCAATAACGCCCTCTTGATAAAACAAGAGGGAAGCTATTGCTCCtttttt  169
            |||||||||||||||||||||||||||||||||||||||||||||||||||||||||||
Sbjct  369  AAAAAAGGAGCAATAACGCCCTCTTGATAAAACAAGAGGGAAGCTATTGCTCCTTTTTT  427


>MN311247.1 Aldama trichophylla chloroplast, complete genome
Length=151299

 Score = 702 bits (380),  Expect = 0.0
 Identities = 483/529 (91%), Gaps = 22/529 (4%)
 Strand=Plus/Minus

Query  2    GTTATGCATGAACGTAATGCTCATAATTTCCCTCTAGACTTAGCTGCTATTGAAGCTCCA  61
            ||||||||||||||||||||||||||||||||||||||||||||||||||||||||||||
Sbjct  545  GTTATGCATGAACGTAATGCTCATAATTTCCCTCTAGACTTAGCTGCTATTGAAGCTCCA  486

Query  62   TATACAAATGGATAAGCCTTTGGTCTGATTGTATAGGAGTTTTTGAACTAAAAAAGGAGC  121
            | |||||||||||||| |||||||||||||||||||||||||||||||||||||||||||
Sbjct  485  TCTACAAATGGATAAGACTTTGGTCTGATTGTATAGGAGTTTTTGAACTAAAAAAGGAGC  426

Query  122  AATAACGCCCTCTTGATAAAACAAGAGGGAAGCTATTGCTCCttttttttATTTAGTACT  181
            ||||||||||||||| |  | |||||||||||||||||||||||||||| ||||||||||
Sbjct  425  AATAACGCCCTCTTGTTTTATCAAGAGGGAAGCTATTGCTCCTTTTTTT-ATTTAGTACT  367

Query  182  ATTTGCCTTACATAGTTTCTTTAAA-------AATAACAAGTACTTTTTATAGTTTGGTT  234
            |||||||||||||||||||||||||       ||||||||||||||||||||||||||||
Sbjct  366  ATTTGCCTTACATAGTTTCTTTAAACTTAAAAAATAACAAGTACTTTTTATAGTTTGGTT  307

Query  235  CGATTTGCGTGtttttctttgtattcatttatattataggtttctatatcatata----  290
            |||||||  |||||||| ||||||||||||||||||||||||||||||||||||||
Sbjct  306  CGATTTGTTTGTTTTTTATTTGTATTCATTTATATTATAGGTTTCTATATCATATACTTT  247

Query  291  t--ttttcccaatcttttatgaagtttt----atttACAATTTAATTTCAATCGAAAATA  344
            |  |||||| ||||||||||||||||||     |||||||| ||| ||||||||||||||
Sbjct  246  TACTTTTCCTAATCTTTTATGAAGTTTTTTTTTTTTACAATCTAAATTCAATCGAAAATA  187

Query  345  GAGAAAAATGACAATTTTGCTTTTTGA-T--TTATATTGATTAAATCAAAATAAGAAAGA  401
            || |||||||||||||||| || || | |  || |||| ||| ||| ||||| |||||||
Sbjct  186  GATAAAAATGACAATTTTGTTTATTTATTACTTTTATTTATTCAATAAAAATGAGAAAGA  127

Query  402  ATATGCtttttttATGTTAATGGAAAAATATAGTAATACTAGATAATCGTAGAGGGGCGG  461
            |||||||||||| |||||||||| ||||||||||||||||||||||| ||||||||||||
Sbjct  126  ATATGCTTTTTT-ATGTTAATGGGAAAATATAGTAATACTAGATAATAGTAGAGGGGCGG  68

Query  462  ATGTAGCCAAGTGGATCAAGGCAGTGGATTGTGAATCCACCATGCGCGG  510
```

```
            |||||||||||||||||||||||||||||||||||||||||||||||||
Sbjct  67   ATGTAGCCAAGTGGATCAAGGCAGTGGATTGTGAATCCACCATGCGCGG  19
```

>NC_053289.1 Aldama nudicaulis voucher Loeuille 734 chloroplast, complete genome
MN337900.1 Aldama nudicaulis chloroplast, complete genome
Length=151333

Score = 702 bits (380), Expect = 0.0
Identities = 483/529 (91%), Gaps = 22/529 (4%)
Strand=Plus/Minus

```
Query  2    GTTATGCATGAACGTAATGCTCATAATTTCCCTCTAGACTTAGCTGCTATTGAAGCTCCA  61
            ||||||||||||||||||||||||||||||||||||||||||||||||||||||||||||
Sbjct  549  GTTATGCATGAACGTAATGCTCATAATTTCCCTCTAGACTTAGCTGCTATTGAAGCTCCA  490

Query  62   TATACAAATGGATAAGCCTTTGGTCTGATTGTATAGGAGTTTTTGAACTAAAAAAGGAGC  121
            | |||||||||||||| |||||||||||||||||||||||||||||||||||||||||||
Sbjct  489  TCTACAAATGGATAAGACTTTGGTCTGATTGTATAGGAGTTTTTGAACTAAAAAAGGAGC  430

Query  122  AATAACGCCCTCTTGATAAAACAAGAGGGAAGCTATTGCTCCttttttttATTTAGTACT  181
            ||||||||||||||| |  | |||||||||||||||||||||||||||| ||||||||||
Sbjct  429  AATAACGCCCTCTTGTTTTATCAAGAGGGAAGCTATTGCTCCTTTTTTT-ATTTAGTACT  371

Query  182  ATTTGCCTTACATAGTTTCTTTAAA-------AATAACAAGTACTTTTTATAGTTTGGTT  234
            |||||||||||||||||||||||||       ||||||||||||||||||||||||||||
Sbjct  370  ATTTGCCTTACATAGTTTCTTTAAACTTTAAAAATAACAAGTACTTTTTATAGTTTGGTT  311

Query  235  CGATTTGCGTGttttttctttgtattcatttatattataggtttctatatcatata----  290
            |||||||  |||||||| ||||||||||||||||||||||||||||||||||||||
Sbjct  310  CGATTTGTTTGTTTTTTATTTGTATTCATTTATATTATAGGTTTCTATATCATATACTTT  251

Query  291  t--ttttcccaatcttttatgaagtttt----atttACAATTTAATTTCAATCGAAAATA  344
            |  |||||| ||||||||||||||||||     |||||||| ||| ||||||||||||||
Sbjct  250  TACTTTTCCTAATCTTTTATGAAGTTTTTTTTTTTTACAATCTAAATTCAATCGAAAATA  191

Query  345  GAGAAAAATGACAATTTTGCTTTTTGA-T--TTATATTGATTAAATCAAAATAAGAAAGA  401
            || |||||||||||||||| || || | |  || |||| ||| ||| ||||| |||||||
Sbjct  190  GATAAAAATGACAATTTTGTTTATTTATTACTTTTATTTATTCAATAAAAATGAGAAAGA  131

Query  402  ATATGCtttttttATGTTAATGGAAAAATATAGTAATACTAGATAATCGTAGAGGGGCGG  461
            |||||||||||| |||||||||| ||||||||||||||||||||||| ||||||||||||
Sbjct  130  ATATGCTTTTTT-ATGTTAATGGGAAAATATAGTAATACTAGATAATAGTAGAGGGGCGG  72

Query  462  ATGTAGCCAAGTGGATCAAGGCAGTGGATTGTGAATCCACCATGCGCGG  510
            |||||||||||||||||||||||||||||||||||||||||||||||||
Sbjct  71   ATGTAGCCAAGTGGATCAAGGCAGTGGATTGTGAATCCACCATGCGCGG  23
```

>MT793841.1 Mikania obtusata chloroplast, complete genome
Length=152106

Score = 702 bits (380), Expect = 0.0
Identities = 477/521 (92%), Gaps = 17/521 (3%)
Strand=Plus/Minus

```
Query  2    GTTATGCATGAACGTAATGCTCATAATTTCCCTCTAGACTTAGCTGCTATTGAAGCTCCA  61
            ||||||||||||||||||||||||||||||||||||||||||||||||||||||||||||
Sbjct  536  GTTATGCATGAACGTAATGCTCATAATTTCCCTCTAGACTTAGCTGCTATTGAAGCTCCA  477

Query  62   TATACAAATGGATAAGCCTTTGGTCTGATTGTATAGGAGTTTTTGAACTAAAAAAGGAGC  121
            | |||||||||||| |||||||||||||||||||||||||||||||||||||||||||||
Sbjct  476  TCTACAAATGGATAAGACTTTGGTCTGATTGTATAGGAGTTTTTGAACTAAAAAAGGAGC  417

Query  122  AATAAC-GCCCTCTTGATAAAACAAGAGGGAAGCTATTGCTCCttttttttATTTAGTAC  180
            |||| |  |||||||| |  | ||||||||  | |||||||||||||||| |||||||||
Sbjct  416  AATAGCTTCCCTCTTGTTTTATCAAGAGGG-CGTTATTGCTCCTTTTTTT-ATTTAGTAC  359

Query  181  TATTTGCCTTACATAGTTTCTTTAAAAATAAC-----------AAGTACTTTTTATAGTT  229
            ||||||||||||||||||||||||||||||||           ||| |||||||||||||
Sbjct  358  TATTTGCCTTACATAGTTTCTTTAAAAATAACTTAAAAATAACAAGGACTTTTTATAGTT  299

Query  230  TGGTTCGATTTGCGTGtttttctttgtattcatttatattataggtttctatatcatat  289
            ||||||||||||||||||||||||||||||||||||||||||||||||||||||| ||||
Sbjct  298  TGGTTCGATTTGCGTGTTTTTCTTTGTATTCATTTATATTATAGGTTTCTATATAATAT  239

Query  290  atttttcccaatcttttatgaagttttatttACAATTTAATTTCAATCGAAAATAGAGAA  349
            |||||||||||||||||||||||||| |||| ||||| |||||||||||||||||||  |
Sbjct  238  ATTTTTCCCAATCTTTTATGAAGTTTGATTTCCAATTCAATTTCAATCGAAAATAGATCA  179

Query  350  AAATGACAATTTTGCTTTTTGATTTATATTGATTAAATCAAAATAAGAAAGAATATGCtt  409
            ||||||||||||||||| || ||| |  || ||| | |  |||||||||| |||||||||
Sbjct  178  AAATGACAATTTTGCTTATTTATT-ACTTTTATTTATT--AAATAAGAAATAATATGCTT  122

Query  410  tttttATGTTAATGGAAAAATATAGTAATACTAGATAATCGTAGAGGGGCGGATGTAGCC  469
            |||| |||||||||||||||||||||||||||||||||| ||||||||||||||||||||
Sbjct  121  TTTTGATGTTAATGGAAAAATATAGTAATACTAGATAATAGTAGAGGGGCGGATGTAGCC  62

Query  470  AAGTGGATCAAGGCAGTGGATTGTGAATCCACCATGCGCGG  510
            |||||||||||||||||||||||||||||||||||||||||
Sbjct  61   AAGTGGATCAAGGCAGTGGATTGTGAATCCACCATGCGCGG  21
```

```
 Score = 110 bits (59),  Expect = 3e-19
 Identities = 59/59 (100%), Gaps = 0/59 (0%)
 Strand=Plus/Plus

Query  111  AAAAAAGGAGCAATAACGCCCTCTTGATAAAACAAGAGGGAAGCTATTGCTCCtttttt  169
            |||||||||||||||||||||||||||||||||||||||||||||||||||||||||||
Sbjct  369  AAAAAAGGAGCAATAACGCCCTCTTGATAAAACAAGAGGGAAGCTATTGCTCCTTTTTT  427
```

>MT793839.1 Mikania neurocaula chloroplast, complete genome
Length=152045

```
 Score = 702 bits (380),  Expect = 0.0
 Identities = 477/521 (92%), Gaps = 17/521 (3%)
 Strand=Plus/Minus

Query  2    GTTATGCATGAACGTAATGCTCATAATTTCCCTCTAGACTTAGCTGCTATTGAAGCTCCA  61
            ||||||||||||||||||||||||||||||||||||||||||||||||||||||||||||
Sbjct  536  GTTATGCATGAACGTAATGCTCATAATTTCCCTCTAGACTTAGCTGCTATTGAAGCTCCA  477
```

```
Query  62   TATACAAATGGATAAGCCTTTGGTCTGATTGTATAGGAGTTTTTGAACTAAAAAAGGAGC  121
            | |||||||||||||| |||||||||||||||||||||||||||||||||||||||||||
Sbjct  476  TCTACAAATGGATAAGACTTTGGTCTGATTGTATAGGAGTTTTTGAACTAAAAAAGGAGC  417

Query  122  AATAAC-GCCCTCTTGATAAAACAAGAGGGAAGCTATTGCTCCttttttttATTTAGTAC  180
            |||| |  |||||||| |  | ||||||||  | |||||||||||||||| |||||||||
Sbjct  416  AATAGCTTCCCTCTTGTTTTATCAAGAGGG-CGTTATTGCTCCTTTTTTT-ATTTAGTAC  359

Query  181  TATTTGCCTTACATAGTTTCTTTAAAAA-----------TAACAAGTACTTTTTATAGTT  229
            ||||||||||||||||||||||||||||           ||||||| |||||||||||||
Sbjct  358  TATTTGCCTTACATAGTTTCTTTAAAAAGAACTTAAAAATAACAAGGACTTTTTATAGTT  299

Query  230  TGGTTCGATTTGCGTGtttttctttgtattcatttatattataggtttctatatcatat  289
            ||||||||||||||||||||||||||||||||||||||||||||||||||||||| ||||
Sbjct  298  TGGTTCGATTTGCGTGTTTTTCTTTGTATTCATTTATATTATAGGTTTCTATATAATAT  239

Query  290  atttttcccaatcttttatgaagttttatttACAATTTAATTTCAATCGAAAATAGAGAA  349
            |||||||||||||||||||||||||| |||| ||||| |||||||||||||||||||  |
Sbjct  238  ATTTTTCCCAATCTTTTATGAAGTTTGATTTCCAATTCAATTTCAATCGAAAATAGATCA  179

Query  350  AAATGACAATTTTGCTTTTTGATTTATATTGATTAAATCAAAATAAGAAAGAATATGCtt  409
            ||||||||||||||||| || |||  || ||| | |  |||||||||| |||||||||
Sbjct  178  AAATGACAATTTTGCTTATTTATT-ACTTTTATTTATT--AAATAAGAAATAATATGCTT  122

Query  410  tttttATGTTAATGGAAAAATATAGTAATACTAGATAATCGTAGAGGGGCGGATGTAGCC  469
            |||| |||||||||||||||||||||||||||||||||| ||||||||||||||||||||
Sbjct  121  TTTTGATGTTAATGGAAAAATATAGTAATACTAGATAATAGTAGAGGGGCGGATGTAGCC  62

Query  470  AAGTGGATCAAGGCAGTGGATTGTGAATCCACCATGCGCGG  510
            |||||||||||||||||||||||||||||||||||||||||
Sbjct  61   AAGTGGATCAAGGCAGTGGATTGTGAATCCACCATGCGCGG  21


 Score = 110 bits (59),  Expect = 3e-19
 Identities = 59/59 (100%), Gaps = 0/59 (0%)
 Strand=Plus/Plus

Query  111  AAAAAAGGAGCAATAACGCCCTCTTGATAAAACAAGAGGGAAGCTATTGCTCCtttttt  169
            |||||||||||||||||||||||||||||||||||||||||||||||||||||||||||
Sbjct  369  AAAAAAGGAGCAATAACGCCCTCTTGATAAAACAAGAGGGAAGCTATTGCTCCTTTTTT  427


>MT793834.1 Mikania decora chloroplast
Length=151914

 Score = 702 bits (380),  Expect = 0.0
 Identities = 477/521 (92%), Gaps = 17/521 (3%)
 Strand=Plus/Minus

Query  2    GTTATGCATGAACGTAATGCTCATAATTTCCCTCTAGACTTAGCTGCTATTGAAGCTCCA  61
            ||||||||||||||||||||||||||||||||||||||||||||||||||||||||||||
Sbjct  536  GTTATGCATGAACGTAATGCTCATAATTTCCCTCTAGACTTAGCTGCTATTGAAGCTCCA  477

Query  62   TATACAAATGGATAAGCCTTTGGTCTGATTGTATAGGAGTTTTTGAACTAAAAAAGGAGC  121
            | |||||||||||||| |||||||||||||||||||||||||||||||||||||||||||
Sbjct  476  TCTACAAATGGATAAGACTTTGGTCTGATTGTATAGGAGTTTTTGAACTAAAAAAGGAGC  417
```

```
Query  122   AATAAC-GCCCTCTTGATAAAACAAGAGGGAAGCTATTGCTCCttttttttATTTAGTAC  180
             |||| |  |||||||| |  | ||||||||  | |||||||||||||||| |||||||||
Sbjct  416   AATAGCTTCCCTCTTGTTTTATCAAGAGGG-CGTTATTGCTCCTTTTTTT-ATTTAGTAC  359

Query  181   TATTTGCCTTACATAGTTTCTTAAAAATAAC-----------AAGTACTTTTTATAGTT  229
             |||||||||||||||||||||||||||||||           ||| |||||||||||||
Sbjct  358   TATTTGCCTTACATAGTTTCTTAAAAATAACTTAAAAATAACAAGGACTTTTTATAGTT  299

Query  230   TGGTTCGATTTGCGTGtttttctttgtattcatttatattataggtttctatatcatat  289
             ||||||||||||||||||||||||||||||||||||||||||||||||||||||| ||||
Sbjct  298   TGGTTCGATTTGCGTGTTTTTCTTTGTATTCATTTATATTATAGGTTTCTATATAATAT  239

Query  290   atttttcccaatcttttatgaagttttatttACAATTTAATTTCAATCGAAAATAGAGAA  349
             |||||||||||||||||||||||||| |||| ||||| |||||||||||||||||||  |
Sbjct  238   ATTTTTCCCAATCTTTTATGAAGTTTGATTTCCAATTCAATTTCAATCGAAAATAGATCA  179

Query  350   AAATGACAATTTTGCTTTTTGATTTATATTGATTAAATCAAAATAAGAAAGAATATGCtt  409
             ||||||||||||||||| || ||| |  || ||| | |  |||||||||| |||||||||
Sbjct  178   AAATGACAATTTTGCTTATTTATT-ACTTTTATTTATT--AAATAAGAAATAATATGCTT  122

Query  410   tttttATGTTAATGGAAAAATATAGTAATACTAGATAATCGTAGAGGGGCGGATGTAGCC  469
             |||| |||||||||||||||||||||||||||||||||| ||||||||||||||||||||
Sbjct  121   TTTTGATGTTAATGGAAAAATATAGTAATACTAGATAATAGTAGAGGGGCGGATGTAGCC  62

Query  470   AAGTGGATCAAGGCAGTGGATTGTGAATCCACCATGCGCGG  510
             |||||||||||||||||||||||||||||||||||||||||
Sbjct  61    AAGTGGATCAAGGCAGTGGATTGTGAATCCACCATGCGCGG  21


 Score = 110 bits (59),  Expect = 3e-19
 Identities = 59/59 (100%), Gaps = 0/59 (0%)
 Strand=Plus/Plus

Query  111   AAAAAAGGAGCAATAACGCCCTCTTGATAAAACAAGAGGGAAGCTATTGCTCCtttttt  169
             |||||||||||||||||||||||||||||||||||||||||||||||||||||||||||
Sbjct  369   AAAAAAGGAGCAATAACGCCCTCTTGATAAAACAAGAGGGAAGCTATTGCTCCTTTTTT  427


>NC_053280.1 Aldama fusiformis voucher Siniscalchi 398 chloroplast, complete
genome
 MN337891.1 Aldama fusiformis chloroplast, complete genome
Length=151314

 Score = 702 bits (380),  Expect = 0.0
 Identities = 482/528 (91%), Gaps = 20/528 (4%)
 Strand=Plus/Minus

Query  2     GTTATGCATGAACGTAATGCTCATAATTTCCCTCTAGACTTAGCTGCTATTGAAGCTCCA  61
             ||||||||||||||||||||||||||||||||||||||||||||||||||||||||||||
Sbjct  549   GTTATGCATGAACGTAATGCTCATAATTTCCCTCTAGACTTAGCTGCTATTGAAGCTCCA  490

Query  62    TATACAAATGGATAAGCCTTTGGTCTGATTGTATAGGAGTTTTTGAACTAAAAAAGGAGC  121
             | |||||||||||||| |||||||||||||||||||||||||||||||||||||||||||
Sbjct  489   TCTACAAATGGATAAGACTTTGGTCTGATTGTATAGGAGTTTTTGAACTAAAAAAGGAGC  430

Query  122   AATAACG-CCCTCTTGATAAAACAAGAGGGAAGCTATTGCTCCttttttttATTTAGTAC  180
             ||||||| |||||||| |  | |||||||||||||||||||||||||||||||||||||||
```

```
Sbjct  429   AATAACGNCCCTCTTGTTTTATCAAGAGGGAAGCTATTGCTCCTTTTTTTTATTTAGTAC  370

Query  181   TATTTGCCTTACATAGTTTCTTTAAA----AATA---ACAAGTACTTTTTATAGTTTGGT  233
             ||||||||||||||||||||||||||    || |   |||||||||||||||||||||||
Sbjct  369   TATTTGCCTTACATAGTTTCTTTAAACTTTAAAAATCACAAGTACTTTTTATAGTTTGGT  310

Query  234   TCGATTTGCGTGttttttctttgtattcatttatattataggtttctatatcatatattt  293
             ||||||||  |||||||| ||||||||||||| |||||||||||||||||||||||| ||
Sbjct  309   TCGATTTGTTTGTTTTTTATTTGTATTCATTTCTATTATAGGTTTCTATATCATATACTT  250

Query  294   tt-c----cc-aatcttttatgaagttttattt--ACAATTTAATTTCAATCGAAAATAG  345
             || |    || |||||||||||||||||| |||  ||||| ||| |||||||||||||||
Sbjct  249   TTACTTTTCCTAATCTTTTATGAAGTTTTTTTTTTACAATCTAAATTCAATCGAAAATAG  190

Query  346   AGAAAAATGACAATTTTGCTTTTTGATT--TA-TATTGATTAAATCAAAATAAGAAAGAA  402
             | |||||||||||||||| || || |||  |  |||| ||| ||| ||||| ||||||||
Sbjct  189   ATAAAAATGACAATTTTGTTTATTTATTACTTTTATTTATTCAATAAAAATGAGAAAGAA  130

Query  403   TATGCtttttttATGTTAATGGAAAAATATAGTAATACTAGATAATCGTAGAGGGGCGGA  462
             ||||||||||| |||||||||| ||||||||||||||||||||||| |||||||||||||
Sbjct  129   TATGCTTTTTT-ATGTTAATGGGAAAATATAGTAATACTAGATAATAGTAGAGGGGCGGA  71

Query  463   TGTAGCCAAGTGGATCAAGGCAGTGGATTGTGAATCCACCATGCGCGG  510
             ||||||||||||||||||||||||||||||||||||||||||||||||
Sbjct  70    TGTAGCCAAGTGGATCAAGGCAGTGGATTGTGAATCCACCATGCGCGG  23


>MT793844.1 Mikania ternata chloroplast
Length=151957

 Score = 702 bits (380),  Expect = 0.0
 Identities = 477/521 (92%), Gaps = 17/521 (3%)
 Strand=Plus/Minus

Query  2     GTTATGCATGAACGTAATGCTCATAATTTCCCTCTAGACTTAGCTGCTATTGAAGCTCCA  61
             ||||||||||||||||||||||||||||||||||||||||||||||||||||||||||||
Sbjct  536   GTTATGCATGAACGTAATGCTCATAATTTCCCTCTAGACTTAGCTGCTATTGAAGCTCCA  477

Query  62    TATACAAATGGATAAGCCTTTGGTCTGATTGTATAGGAGTTTTTGAACTAAAAAAGGAGC  121
             | |||||||||||||| |||||||||||||||||||||||||||||||||||||||||||
Sbjct  476   TCTACAAATGGATAAGACTTTGGTCTGATTGTATAGGAGTTTTTGAACTAAAAAAGGAGC  417

Query  122   AATAAC-GCCCTCTTGATAAAACAAGAGGGAAGCTATTGCTCCttttttttATTTAGTAC  180
             |||| |  |||||||| |  | ||||||||  | |||||||||||||||| |||||||||
Sbjct  416   AATAGCTTCCCTCTTGTTTTATCAAGAGGG-CGTTATTGCTCCTTTTTTT-ATTTAGTAC  359

Query  181   TATTTGCCTTACATAGTTTCTTTAAAAATAAC-----------AAGTACTTTTTATAGTT  229
             ||||||||||||||||||||||||||||||||           ||| |||||||||||||
Sbjct  358   TATTTGCCTTACATAGTTTCTTTAAAAATAACTTAAAAATAACAAGGACTTTTTATAGTT  299

Query  230   TGGTTCGATTTGCGTGttttttctttgtattcatttatattataggtttctatatcatat  289
             ||||||||||||||||||||||||||||||||||||||||||||||||||||||| ||||
Sbjct  298   TGGTTCGATTTGCGTGTTTTTTCTTTGTATTCATTTATATTATAGGTTTCTATATAATAT  239

Query  290   atttttcccaatcttttatgaagttttatttACAATTTAATTTCAATCGAAAATAGAGAA  349
             |||||||||||||||||||||||||| |||| ||||| |||||||||||||||||||  |
Sbjct  238   ATTTTTCCCAATCTTTTATGAAGTTTGATTTCCAATTCAATTTCAATCGAAAATAGATCA  179
```

```
Query  350  AAATGACAATTTTGCTTTTTGATTTATATTGATTAAATCAAAATAAGAAAGAATATGCtt  409
            ||||||||||||||||| || ||| |  || ||| | |  |||||||||| |||||||||
Sbjct  178  AAATGACAATTTTGCTTATTTATT-ACTTTTATTTATT--AAATAAGAAATAATATGCTT  122

Query  410  tttttATGTTAATGGAAAAATATAGTAATACTAGATAATCGTAGAGGGGCGGATGTAGCC  469
            |||| |||||||||||||||||||||||||||||||||| ||||||||||||||||||||
Sbjct  121  TTTTGATGTTAATGGAAAAATATAGTAATACTAGATAATAGTAGAGGGGCGGATGTAGCC  62

Query  470  AAGTGGATCAAGGCAGTGGATTGTGAATCCACCATGCGCGG  510
            |||||||||||||||||||||||||||||||||||||||||
Sbjct  61   AAGTGGATCAAGGCAGTGGATTGTGAATCCACCATGCGCGG  21


 Score = 110 bits (59),  Expect = 3e-19
 Identities = 59/59 (100%), Gaps = 0/59 (0%)
 Strand=Plus/Plus

Query  111  AAAAAAGGAGCAATAACGCCCTCTTGATAAAACAAGAGGGAAGCTATTGCTCCtttttt  169
            |||||||||||||||||||||||||||||||||||||||||||||||||||||||||||
Sbjct  369  AAAAAAGGAGCAATAACGCCCTCTTGATAAAACAAGAGGGAAGCTATTGCTCCTTTTTT  427


>KF241798.1 Ambrosia artemisiifolia isolate AZ-1 PsbA (psbA) gene, partial
cds; and psbA-trnH intergenic spacer, partial sequence; chloroplast
Length=469

 Score = 702 bits (380),  Expect = 0.0
 Identities = 445/474 (94%), Gaps = 13/474 (3%)
 Strand=Plus/Plus

Query  9    ATGAACGTAATGCTCATAATTTCCCTCTAGACTTAGCTGCTATTGAAGCTCCATATACAA  68
            |||||||||||||||||||||||||||||||||||||||||||||||||||||| |||||
Sbjct  1    ATGAACGTAATGCTCATAATTTCCCTCTAGACTTAGCTGCTATTGAAGCTCCATCTACAA  60

Query  69   ATGGATAAGCCTTTGGTCTGATTGTATAGGAGTTTTTGAACTAAAAAAGGAGCAATAACG  128
            ||||||||| ||||||||||||||||||||||||||||||||||||||||||||||||||
Sbjct  61   ATGGATAAGACTTTGGTCTGATTGTATAGGAGTTTTTGAACTAAAAAAGGAGCAATAACG  120

Query  129  CCCTCTTGATAAAACAAGAGGGAAGCTATTGCTCCttttttttATTTAGTACTATTTGCC  188
            ||||||||||||||||||||||||||||||||||||||||||||||||||| ||||||||
Sbjct  121  CCCTCTTGATAAAACAAGAGGGAAGCTATTGCTCCTTTTTTTTATTTAGTATTATTTGCC  180

Query  189  TTACATAGTTTCTTTAAAAATAACAAGTACTTTTTATAGTTTGGTTCGATTTGCGTGttt  248
            |||||||||||||||||||||||||||||||||||||||||||||||||||||| |||||
Sbjct  181  TTACATAGTTTCTTTAAAAATAACAAGTACTTTTTATAGTTTGGTTCGATTTGCATGTTT  240

Query  249  tttctttgtattcatttatattataggtttctatatcatatatttttcccaatcttttat  308
            ||| ||||||||||||||||||||||||||||| || |||   |||||||||||||||||
Sbjct  241  TTTATTTGTATTCATTTATATTATAGGTTTCTA-AT-ATAC--TTTTCCCAATCTTTTAT  296

Query  309  gaagttttatttACAATTTAATTTCAATCGAAAATAGAGAAAAATGACAATTTTGCTTTT  368
            |||||||||||||||||| ||||||||||||||||||| ||||||||||||||||||| |
Sbjct  297  GAAGTTTTATTTACAATTCAATTTCAATCGAAAATAGATAAAAATGACAATTTTGCTTAT  356

Query  369  TGATT--TA-TATTGATTAAATCAAAATAAGAAAGAA-TA-T--GCttttttt-ATGTTA  420
            | |||  |  |||| ||||||| ||||| |||||||| || |  ||||||||| || |||
```

```
Sbjct  357  TTATTACTTTTATTTATTAAATAAAAATCAGAAAGAAATAATATGCTTTTTTTTAT-TTA  415

Query  421  ATGGAAAAATATAGTAATACTAGATAATCGTAGAGGGGCGGATGTAGCCAAGTG  474
            ||||||||||||| |||||||||||||| |||||||||||||||||||||||||
Sbjct  416  ATGGAAAAATATATTAATACTAGATAATAGTAGAGGGGCGGATGTAGCCAAGTG  469


>MW353165.1 Helianthus laciniatus chloroplast, complete genome
Length=151113

 Score = 701 bits (379),  Expect = 0.0
 Identities = 482/528 (91%), Gaps = 22/528 (4%)
 Strand=Plus/Minus

Query  2    GTTATGCATGAACGTAATGCTCATAATTTCCCTCTAGACTTAGCTGCTATTGAAGCTCCA  61
            ||||||||||||||||||||||||||||||||||||||||||||||||||||||||||||
Sbjct  546  GTTATGCATGAACGTAATGCTCATAATTTCCCTCTAGACTTAGCTGCTATTGAAGCTCCA  487

Query  62   TATACAAATGGATAAGCCTTTGGTCTGATTGTATAGGAGTTTTTGAACTAAAAAAGGAGC  121
            | |||||||||||||| |||||||||||||||||||||||||||||||||||||||||||
Sbjct  486  TCTACAAATGGATAAGACTTTGGTCTGATTGTATAGGAGTTTTTGAACTAAAAAAGGAGC  427

Query  122  AATAAC-GCCCTCTTGATAAAACAAGAGGGAAGCTATTGCTCCttttttttATTTAGTAC  180
            |||| |  ||||||||||||||||||||||  | ||||||||||||||||||||||||||
Sbjct  426  AATAGCTTCCCTCTTGATAAAACAAGAGGG-CGTTATTGCTCCTTTTTTTTATTTAGTAC  368

Query  181  TATTTGCCTTACATAGTTTCTTTAAA-------AATAACAAGTACTTTTTATAGTTTGGT  233
            ||||||||||||||||||||||||||       |||||||||||||||||||||||||||
Sbjct  367  TATTTGCCTTACATAGTTTCTTTAAACTTTAAAAATAACAAGTACTTTTTATAGTTTGGT  308

Query  234  TCGATTTGCGTGttttttctttgtattcatttatattataggtttctatatcatata---  290
            | ||||||  |||||||  ||||||||||||||||||||||||||||| ||||||||
Sbjct  307  TTGATTTGTTTGTTTTT-ATTTGTATTCATTTATATTATAGGTTTCTAGATCATATACTT  249

Query  291  -t--ttttcccaatcttttatgaagtttt--atttACAATTTAATTTCAATCGAAAATAG  345
             |  |||||| ||||||||||||||||||   |||||||||||| |||||| ||||||||
Sbjct  248  TTACTTTTCCTAATCTTTTATGAAGTTTTTTTTTTACAATTTAAATTCAATTGAAAATAG  189

Query  346  AGAAAAATGACAATTTTGCTT--TT-TGATTTATATTGATTAAATCAAAATAAGAAAGAA  402
            | |||||||| ||||||||||  || | |||| |||| ||| ||| ||||| ||||||||
Sbjct  188  ATAAAAATGAAAATTTTGCTTATTTATTATTTTTATTTATTCAATAAAAATGAGAAAGAA  129

Query  403  TATGCtttttttATGTTAATGGAAAAATATAGTAATACTAGATAATCGTAGAGGGGCGGA  462
            ||||||||||| |||||||||||||||||||||||||||||||||| |||||||||||||
Sbjct  128  TATGCTTTTTT-ATGTTAATGGAAAAATATAGTAATACTAGATAATAGTAGAGGGGCGGA  70

Query  463  TGTAGCCAAGTGGATCAAGGCAGTGGATTGTGAATCCACCATGCGCGG  510
            ||||||||||||||||||||||||||||||||||||||||||||||||
Sbjct  69   TGTAGCCAAGTGGATCAAGGCAGTGGATTGTGAATCCACCATGCGCGG  22


>NC_058797.1 Helianthus verticillatus chloroplast, complete genome
 MW375414.1 Helianthus verticillatus chloroplast, complete genome
Length=151190

 Score = 701 bits (379),  Expect = 0.0
 Identities = 483/529 (91%), Gaps = 23/529 (4%)
```

```
 Strand=Plus/Minus

Query  2    GTTATGCATGAACGTAATGCTCATAATTTCCCTCTAGACTTAGCTGCTATTGAAGCTCCA  61
            ||||||||||||||||||||||||||||||||||||||||||||||||||||||||||||
Sbjct  615  GTTATGCATGAACGTAATGCTCATAATTTCCCTCTAGACTTAGCTGCTATTGAAGCTCCA  556

Query  62   TATACAAATGGATAAGCCTTTGGTCTGATTGTATAGGAGTTTTTGAACTAAAAAAGGAGC  121
            | |||||||||||||| |||||||||||||||||||||||||||||||||||||||||||
Sbjct  555  TCTACAAATGGATAAGACTTTGGTCTGATTGTATAGGAGTTTTTGAACTAAAAAAGGAGC  496

Query  122  AATAAC-GCCCTCTTGATAAAACAAGAGGGAAGCTATTGCTCCtttttttt-ATTTAGTA  179
            |||| |  ||||||||||||||||||||||  | ||||||||||||||||| ||||||||
Sbjct  495  AATAGCTTCCCTCTTGATAAAACAAGAGGG-CGTTATTGCTCCTTTTTTTTTATTTAGTA  437

Query  180  CTATTTGCCTTACATAGTTTCTTTAAA-------AATAACAAGTACTTTTTATAGTTTGG  232
            |||||||||||||||||||||||||||       ||||||||||||||||||||||||||
Sbjct  436  CTATTTGCCTTACATAGTTTCTTTAAACTTTAAAAATAACAAGTACTTTTTATAGTTTGG  377

Query  233  TTCGATTTGCGTGttttttctttgtattcatttatattataggtttctatatcatata--  290
            |||||||||  |||||||  ||||||||||||||||||||||||||||||||||||||
Sbjct  376  TTCGATTTGTTTGTTTTT-ATTTGTATTCATTTATATTATAGGTTTCTATATCATATACT  318

Query  291  --t--ttttcccaatcttttatgaagtttt--atttACAATTTAATTTCAATCGAAAATA  344
              |  |||||| ||||||||||||||||||   |||||||||||| |||||| |||||||
Sbjct  317  TTTACTTTTCCTAATCTTTTATGAAGTTTTTTTTTTACAATTTAAATTCAATTGAAAATA  258

Query  345  GAGAAAAATGACAATTTTGCTT--TT-TGATTTATATTGATTAAATCAAAATAAGAAAGA  401
            || |||||||| ||||||||||  || | | || |||| ||| ||| ||||| |||||||
Sbjct  257  GATAAAAATGAAAATTTTGCTTATTTATTACTTTTATTTATTCAATAAAAATGAGAAAGA  198

Query  402  ATATGCtttttttATGTTAATGGAAAAATATAGTAATACTAGATAATCGTAGAGGGGCGG  461
            |||||||||||| |||||||||||||||||||||||||||||||||||| ||||||||||||
Sbjct  197  ATATGCTTTTTT-ATGTTAATGGAAAAATATAGTAATACTAGATAATAGTAGAGGGGCGG  139

Query  462  ATGTAGCCAAGTGGATCAAGGCAGTGGATTGTGAATCCACCATGCGCGG  510
            |||||||||||||||||||||||||||||||||||||||||||||||||
Sbjct  138  ATGTAGCCAAGTGGATCAAGGCAGTGGATTGTGAATCCACCATGCGCGG  90


>KF746360.1 Helianthus hirsutus isolate DB15 plastid, complete genome
Length=151046

 Score = 701 bits (379),  Expect = 0.0
 Identities = 483/529 (91%), Gaps = 23/529 (4%)
 Strand=Plus/Minus

Query  2    GTTATGCATGAACGTAATGCTCATAATTTCCCTCTAGACTTAGCTGCTATTGAAGCTCCA  61
            ||||||||||||||||||||||||||||||||||||||||||||||||||||||||||||
Sbjct  547  GTTATGCATGAACGTAATGCTCATAATTTCCCTCTAGACTTAGCTGCTATTGAAGCTCCA  488

Query  62   TATACAAATGGATAAGCCTTTGGTCTGATTGTATAGGAGTTTTTGAACTAAAAAAGGAGC  121
            | |||||||||||||| |||||||||||||||||||||||||||||||||||||||||||
Sbjct  487  TCTACAAATGGATAAGACTTTGGTCTGATTGTATAGGAGTTTTTGAACTAAAAAAGGAGC  428

Query  122  AATAAC-GCCCTCTTGATAAAACAAGAGGGAAGCTATTGCTCCtttttttt-ATTTAGTA  179
            |||| |  ||||||||||||||||||||||  | ||||||||||||||||| ||||||||
Sbjct  427  AATAGCTTCCCTCTTGATAAAACAAGAGGG-CGTTATTGCTCCTTTTTTTTTATTTAGTA  369
```

```
Query  180   CTATTTGCCTTACATAGTTTCTTTAAA-------AATAACAAGTACTTTTTATAGTTTGG  232
             |||||||||||||||||||||||||||       ||||||||||||||||||||||||||
Sbjct  368   CTATTTGCCTTACATAGTTTCTTTAAACTTTAAAAATAACAAGTACTTTTTATAGTTTGG  309

Query  233   TTCGATTTGCGTGtttttctttgtattcatttatattataggtttctatatcatata--  290
             |||||||||  |||||||  ||||||||||||||||||||||||||||||||||||||
Sbjct  308   TTCGATTTGTTTGTTTTT-ATTTGTATTCATTTATATTATAGGTTTCTATATCATATACT  250

Query  291   --t--ttttcccaatcttttatgaagtttt--atttACAATTTAATTTCAATCGAAAATA  344
               |  |||||| ||||||||||||||||||   |||||||||||| |||||| |||||||
Sbjct  249   TTTACTTTTCCTAATCTTTTATGAAGTTTTTTTTTTACAATTTAAATTCAATTGAAAATA  190

Query  345   GAGAAAAATGACAATTTTGCTT--TT-TGATTTATATTGATTAAATCAAAATAAGAAAGA  401
             || |||||||| ||||||||||  || | | || |||| ||| ||| ||||| |||||||
Sbjct  189   GATAAAAATGAAAATTTTGCTTATTTATTACTTTTATTTATTCAATAAAAATGAGAAAGA  130

Query  402   ATATGCttttttttATGTTAATGGAAAAATATAGTAATACTAGATAATCGTAGAGGGGCGG  461
             |||||||||||| |||||||||||||||||||||||||||||||||||||| ||||||||||||
Sbjct  129   ATATGCTTTTTT-ATGTTAATGGAAAAATATAGTAATACTAGATAATAGTAGAGGGGCGG  71

Query  462   ATGTAGCCAAGTGGATCAAGGCAGTGGATTGTGAATCCACCATGCGCGG  510
             |||||||||||||||||||||||||||||||||||||||||||||||||
Sbjct  70    ATGTAGCCAAGTGGATCAAGGCAGTGGATTGTGAATCCACCATGCGCGG  22


>KF746363.1 Helianthus tuberosus isolate DB18 plastid, complete genome
Length=151047

 Score = 701 bits (379),  Expect = 0.0
 Identities = 483/529 (91%), Gaps = 23/529 (4%)
 Strand=Plus/Minus

Query  2     GTTATGCATGAACGTAATGCTCATAATTTCCCTCTAGACTTAGCTGCTATTGAAGCTCCA  61
             ||||||||||||||||||||||||||||||||||||||||||||||||||||||||||||
Sbjct  547   GTTATGCATGAACGTAATGCTCATAATTTCCCTCTAGACTTAGCTGCTATTGAAGCTCCA  488

Query  62    TATACAAATGGATAAGCCTTTGGTCTGATTGTATAGGAGTTTTTGAACTAAAAAAGGAGC  121
             | |||||||||||||| |||||||||||||||||||||||||||||||||||||||||||
Sbjct  487   TCTACAAATGGATAAGACTTTGGTCTGATTGTATAGGAGTTTTTGAACTAAAAAAGGAGC  428

Query  122   AATAAC-GCCCTCTTGATAAAACAAGAGGGAAGCTATTGCTCCttttttttATTTAGTAC  180
             |||| |  ||||||||||||||||||||||  | ||||||||||||||||||||||||||
Sbjct  427   AATAGCTTCCCTCTTGATAAAACAAGAGGG-CGTTATTGCTCCTTTTTTTTATTTAGTAC  369

Query  181   TATTTGCCTTACATAGTTTCTTTAAA-------AATAACAAGTACTTTTTATAGTTTGGT  233
             ||||||||||||||||||||||||||       |||||||||||||||||||||||||||
Sbjct  368   TATTTGCCTTACATAGTTTCTTTAAACTTTAAAAATAACAAGTACTTTTTATAGTTTGGT  309

Query  234   TCGATTTGCGTGtttttctttgtattcatttatattataggtttctatatcatata---  290
             ||||||||  |||||||  ||||||||||||||||||||||||||||||||||||||
Sbjct  308   TCGATTTGTTTGTTTTT-ATTTGTATTCATTTATATTATAGGTTTCTATATCATATACTT  250

Query  291   -t--ttttcccaatcttttatgaagtttt---atttACAATTTAATTTCAATCGAAAATA  344
              |  |||||| ||||||||||||||||||    |||||||||||| |||||| |||||||
Sbjct  249   TTACTTTTCCTAATCTTTTATGAAGTTTTTTTTTTTACAATTTAAATTCAATTGAAAATA  190
```

```
Query  345   GAGAAAAATGACAATTTTGCTT--TT-TGATTTATATTGATTAAATCAAAATAAGAAAGA  401
             || |||||||| ||||||||||  || | | || |||| ||| ||| ||||| |||||||
Sbjct  189   GATAAAAATGAAAATTTTGCTTATTTATTACTTTTATTTATTCAATAAAAATGAGAAAGA  130

Query  402   ATATGCtttttttATGTTAATGGAAAAATATAGTAATACTAGATAATCGTAGAGGGGCGG  461
             |||||||||||| ||||||||||||||||||||||||||||||||||| ||||||||||||
Sbjct  129   ATATGCTTTTTT-ATGTTAATGGAAAAATATAGTAATACTAGATAATAGTAGAGGGGCGG  71

Query  462   ATGTAGCCAAGTGGATCAAGGCAGTGGATTGTGAATCCACCATGCGCGG  510
             |||||||||||||||||||||||||||||||||||||||||||||||||
Sbjct  70    ATGTAGCCAAGTGGATCAAGGCAGTGGATTGTGAATCCACCATGCGCGG  22
```

>MT302564.1 Helianthus pauciflorus subsp. pauciflorus chloroplast, complete genome
Length=151210

Score = 701 bits (379), Expect = 0.0
Identities = 483/529 (91%), Gaps = 23/529 (4%)
Strand=Plus/Minus

```
Query  2     GTTATGCATGAACGTAATGCTCATAATTTCCCTCTAGACTTAGCTGCTATTGAAGCTCCA  61
             ||||||||||||||||||||||||||||||||||||||||||||||||||||||||||||
Sbjct  546   GTTATGCATGAACGTAATGCTCATAATTTCCCTCTAGACTTAGCTGCTATTGAAGCTCCA  487

Query  62    TATACAAATGGATAAGCCTTTGGTCTGATTGTATAGGAGTTTTTGAACTAAAAAAGGAGC  121
             | |||||||||||||| |||||||||||||||||||||||||||||||||||||||||||
Sbjct  486   TCTACAAATGGATAAGACTTTGGTCTGATTGTATAGGAGTTTTTGAACTAAAAAAGGAGC  427

Query  122   AATAAC-GCCCTCTTGATAAAACAAGAGGGAAGCTATTGCTCCttttttttt-ATTTAGTA  179
             |||| |  |||||||||||||||||||||  | |||||||||||||||| ||||||||
Sbjct  426   AATAGCTTCCCTCTTGATAAAACAAGAGGG-CGTTATTGCTCCTTTTTTTTTATTTAGTA  368

Query  180   CTATTTGCCTTACATAGTTTCTTTAAA-------AATAACAAGTACTTTTTATAGTTTGG  232
             |||||||||||||||||||||||||||       ||||||||||||||||||||||||||
Sbjct  367   CTATTTGCCTTACATAGTTTCTTTAAACTTTAAAAATAACAAGTACTTTTTATAGTTTGG  308

Query  233   TTCGATTTGCGTGtttttttctttgtattcatttatattataggtttctatatcatata--  290
             |||||||||  |||||||  ||||||||||||||||||||||||||||||||||||||
Sbjct  307   TTCGATTTGTTTGTTTTT-ATTTGTATTCATTTATATTATAGGTTTCTATATCATATACT  249

Query  291   --t--ttttcccaatcttttatgaagtttt--atttACAATTTAATTTCAATCGAAAATA  344
               |  |||||| ||||||||||||||||||   |||||||||||| |||||| |||||||
Sbjct  248   TTTACTTTTCCTAATCTTTTATGAAGTTTTTTTTTACAATTTAAATTCAATTGAAAATA  189

Query  345   GAGAAAAATGACAATTTTGCTT--TT-TGATTTATATTGATTAAATCAAAATAAGAAAGA  401
             || |||||||| ||||||||||  || | | || |||| ||| ||| ||||| |||||||
Sbjct  188   GATAAAAATGAAAATTTTGCTTATTTATTACTTTTATTTATTCAATAAAAATGAGAAAGA  129

Query  402   ATATGCtttttttATGTTAATGGAAAAATATAGTAATACTAGATAATCGTAGAGGGGCGG  461
             |||||||||||| ||||||||||||||||||||||||||||||||||| ||||||||||||
Sbjct  128   ATATGCTTTTTT-ATGTTAATGGAAAAATATAGTAATACTAGATAATAGTAGAGGGGCGG  70

Query  462   ATGTAGCCAAGTGGATCAAGGCAGTGGATTGTGAATCCACCATGCGCGG  510
             |||||||||||||||||||||||||||||||||||||||||||||||||
Sbjct  69    ATGTAGCCAAGTGGATCAAGGCAGTGGATTGTGAATCCACCATGCGCGG  21
```

```
>KF746378.1 Helianthus tuberosus isolate DB33 plastid, complete genome
Length=151044

 Score = 701 bits (379),  Expect = 0.0
 Identities = 483/529 (91%), Gaps = 23/529 (4%)
 Strand=Plus/Minus

Query  2    GTTATGCATGAACGTAATGCTCATAATTTCCCTCTAGACTTAGCTGCTATTGAAGCTCCA  61
            ||||||||||||||||||||||||||||||||||||||||||||||||||||||||||||
Sbjct  547  GTTATGCATGAACGTAATGCTCATAATTTCCCTCTAGACTTAGCTGCTATTGAAGCTCCA  488

Query  62   TATACAAATGGATAAGCCTTTGGTCTGATTGTATAGGAGTTTTTGAACTAAAAAAGGAGC  121
            | |||||||||||||| |||||||||||||||||||||||||||||||||||||||||||
Sbjct  487  TCTACAAATGGATAAGACTTTGGTCTGATTGTATAGGAGTTTTTGAACTAAAAAAGGAGC  428

Query  122  AATAAC-GCCCTCTTGATAAAACAAGAGGGAAGCTATTGCTCCtttttttt-ATTTAGTA  179
            |||| |  ||||||||||||||||||||||  | ||||||||||||||||| ||||||||
Sbjct  427  AATAGCTTCCCTCTTGATAAAACAAGAGGG-CGTTATTGCTCCTTTTTTTTTATTTAGTA  369

Query  180  CTATTTGCCTTACATAGTTTCTTTAAA-------AATAACAAGTACTTTTTATAGTTTGG  232
            |||||||||||||||||||||||||||       ||||||||||||||||||||||||||
Sbjct  368  CTATTTGCCTTACATAGTTTCTTTAAACTTTAAAAATAACAAGTACTTTTTATAGTTTGG  309

Query  233  TTCGATTTGCGTGttttttctttgtattcatttatattataggtttctatatcatata--  290
            |||||||||  |||||||  ||||||||||||||||||||||||||||||||||||||  
Sbjct  308  TTCGATTTGTTTGTTTTT-ATTTGTATTCATTTATATTATAGGTTTCTATATCATATACT  250

Query  291  --t--ttttcccaatcttttatgaagtttt--atttACAATTTAATTTCAATCGAAAATA  344
              |  |||||| ||||||||||||||||||   |||||||||||| |||||| |||||||
Sbjct  249  TTTACTTTTCCTAATCTTTTATGAAGTTTTTTTTTTACAATTTAAATTCAATTGAAAATA  190

Query  345  GAGAAAAATGACAATTTTGCTT--TT-TGATTTATATTGATTAAATCAAAATAAGAAAGA  401
            || |||||||| ||||||||||  || | | || |||| ||| ||| ||||| |||||||
Sbjct  189  GATAAAAATGAAAATTTTGCTTATTTATTACTTTTATTTATTCAATAAAAATGAGAAAGA  130

Query  402  ATATGCtttttttATGTTAATGGAAAAATATAGTAATACTAGATAATCGTAGAGGGGCGG  461
            |||||||||||| |||||||||||||||||||||||||||||||||| ||||||||||||
Sbjct  129  ATATGCTTTTTT-ATGTTAATGGAAAAATATAGTAATACTAGATAATAGTAGAGGGGCGG  71

Query  462  ATGTAGCCAAGTGGATCAAGGCAGTGGATTGTGAATCCACCATGCGCGG  510
            |||||||||||||||||||||||||||||||||||||||||||||||||
Sbjct  70   ATGTAGCCAAGTGGATCAAGGCAGTGGATTGTGAATCCACCATGCGCGG  22


>KF746364.1 Helianthus divaricatus isolate DB19 plastid, complete genome
Length=151047

 Score = 701 bits (379),  Expect = 0.0
 Identities = 483/529 (91%), Gaps = 23/529 (4%)
 Strand=Plus/Minus

Query  2    GTTATGCATGAACGTAATGCTCATAATTTCCCTCTAGACTTAGCTGCTATTGAAGCTCCA  61
            ||||||||||||||||||||||||||||||||||||||||||||||||||||||||||||
Sbjct  547  GTTATGCATGAACGTAATGCTCATAATTTCCCTCTAGACTTAGCTGCTATTGAAGCTCCA  488

Query  62   TATACAAATGGATAAGCCTTTGGTCTGATTGTATAGGAGTTTTTGAACTAAAAAAGGAGC  121
```

```
                | |||||||||||||| |||||||||||||||||||||||||||||||||||||||||||
Sbjct  487  TCTACAAATGGATAAGACTTTGGTCTGATTGTATAGGAGTTTTTGAACTAAAAAAGGAGC  428

Query  122  AATAAC-GCCCTCTTGATAAAACAAGAGGGAAGCTATTGCTCCtttttttt-ATTTAGTA  179
            |||| |  ||||||||||||||||||||||  | ||||||||||||||||| ||||||||
Sbjct  427  AATAGCTTCCCTCTTGATAAAACAAGAGGG-CGTTATTGCTCCTTTTTTTTTATTTAGTA  369

Query  180  CTATTTGCCTTACATAGTTTCTTTAAA-------AATAACAAGTACTTTTTATAGTTTGG  232
            |||||||||||||||||||||||||||       ||||||||||||||||||||||||||
Sbjct  368  CTATTTGCCTTACATAGTTTCTTTAAACTTTAAAAATAACAAGTACTTTTTATAGTTTGG  309

Query  233  TTCGATTTGCGTGttttttctttgtattcatttatattataggtttctatatcatata--  290
            |||||||||  |||||||  ||||||||||||||||||||||||||||||||||||||
Sbjct  308  TTCGATTTGTTTGTTTTT-ATTTGTATTCATTTATATTATAGGTTTCTATATCATATACT  250

Query  291  --t--ttttcccaatcttttatgaagtttt--atttACAATTTAATTTCAATCGAAAATA  344
              |  |||||| ||||||||||||||||||   |||||||||||| |||||| |||||||
Sbjct  249  TTTACTTTTCCTAATCTTTTATGAAGTTTTTTTTTTACAATTTAAATTCAATTGAAAATA  190

Query  345  GAGAAAAATGACAATTTTGCTT--TT-TGATTTATATTGATTAAATCAAAATAAGAAAGA  401
            || |||||||| ||||||||||  || | | || |||| ||| ||| ||||| |||||||
Sbjct  189  GATAAAAATGAAAATTTTGCTTATTTATTACTTTTATTTATTCAATAAAAATGAGAAAGA  130

Query  402  ATATGCtttttttATGTTAATGGAAAAATATAGTAATACTAGATAATCGTAGAGGGGCGG  461
            |||||||||||| |||||||||||||||||||||||||||||||||| ||||||||||||
Sbjct  129  ATATGCTTTTTT-ATGTTAATGGAAAAATATAGTAATACTAGATAATAGTAGAGGGGCGG  71

Query  462  ATGTAGCCAAGTGGATCAAGGCAGTGGATTGTGAATCCACCATGCGCGG  510
            |||||||||||||||||||||||||||||||||||||||||||||||||
Sbjct  70   ATGTAGCCAAGTGGATCAAGGCAGTGGATTGTGAATCCACCATGCGCGG  22


>MG696658.1 Helianthus tuberosus chloroplast, complete genome
Length=151431

 Score = 701 bits (379),  Expect = 0.0
 Identities = 483/529 (91%), Gaps = 23/529 (4%)
 Strand=Plus/Minus

Query  2    GTTATGCATGAACGTAATGCTCATAATTTCCCTCTAGACTTAGCTGCTATTGAAGCTCCA  61
            ||||||||||||||||||||||||||||||||||||||||||||||||||||||||||||
Sbjct  673  GTTATGCATGAACGTAATGCTCATAATTTCCCTCTAGACTTAGCTGCTATTGAAGCTCCA  614

Query  62   TATACAAATGGATAAGCCTTTGGTCTGATTGTATAGGAGTTTTTGAACTAAAAAAGGAGC  121
            | |||||||||||||| |||||||||||||||||||||||||||||||||||||||||||
Sbjct  613  TCTACAAATGGATAAGACTTTGGTCTGATTGTATAGGAGTTTTTGAACTAAAAAAGGAGC  554

Query  122  AATAAC-GCCCTCTTGATAAAACAAGAGGGAAGCTATTGCTCCtttttttt-ATTTAGTA  179
            |||| |  ||||||||||||||||||||||  | ||||||||||||||||| ||||||||
Sbjct  553  AATAGCTTCCCTCTTGATAAAACAAGAGGG-CGTTATTGCTCCTTTTTTTTTATTTAGTA  495

Query  180  CTATTTGCCTTACATAGTTTCTTTAAA-------AATAACAAGTACTTTTTATAGTTTGG  232
            |||||||||||||||||||||||||||       ||||||||||||||||||||||||||
Sbjct  494  CTATTTGCCTTACATAGTTTCTTTAAACTTTAAAAATAACAAGTACTTTTTATAGTTTGG  435

Query  233  TTCGATTTGCGTGttttttctttgtattcatttatattataggtttctatatcatata--  290
            |||||||||  |||||||  ||||||||||||||||||||||||||||||||||||||
```

```
Sbjct  434  TTCGATTTGTTTGTTTTT-ATTTGTATTCATTTATATTATAGGTTTCTATATCATATACT  376

Query  291  --t--ttttcccaatcttttatgaagtttt--atttACAATTTAATTTCAATCGAAAATA  344
              |  |||||| ||||||||||||||||||   |||||||||||| |||||| |||||||
Sbjct  375  TTTACTTTTCCTAATCTTTTATGAAGTTTTTTTTTTACAATTTAAATTCAATTGAAAATA  316

Query  345  GAGAAAAATGACAATTTTGCTT--TT-TGATTTATATTGATTAAATCAAAATAAGAAAGA  401
            || |||||||| ||||||||||  || | | || |||| ||| ||| ||||| |||||||
Sbjct  315  GATAAAAATGAAAATTTTGCTTATTTATTACTTTTATTTATTCAATAAAAATGAGAAAGA  256

Query  402  ATATGCtttttttATGTTAATGGAAAAATATAGTAATACTAGATAATCGTAGAGGGGCGG  461
            |||||||||||| |||||||||||||||||||||||||||||||||| ||||||||||||
Sbjct  255  ATATGCTTTTTT-ATGTTAATGGAAAAATATAGTAATACTAGATAATAGTAGAGGGGCGG  197

Query  462  ATGTAGCCAAGTGGATCAAGGCAGTGGATTGTGAATCCACCATGCGCGG  510
            |||||||||||||||||||||||||||||||||||||||||||||||||
Sbjct  196  ATGTAGCCAAGTGGATCAAGGCAGTGGATTGTGAATCCACCATGCGCGG  148


>MT793848.1 Litothamnus nitidus chloroplast, complete genome
Length=151517

 Score = 701 bits (379),  Expect = 0.0
 Identities = 475/519 (92%), Gaps = 15/519 (3%)
 Strand=Plus/Minus

Query  2    GTTATGCATGAACGTAATGCTCATAATTTCCCTCTAGACTTAGCTGCTATTGAAGCTCCA  61
            ||||||||||||||||||||||||||||||||||||||||||||||||||||||||||||
Sbjct  534  GTTATGCATGAACGTAATGCTCATAATTTCCCTCTAGACTTAGCTGCTATTGAAGCTCCA  475

Query  62   TATACAAATGGATAAGCCTTTGGTCTGATTGTATAGGAGTTTTTGAACTAAAAAAGGAGC  121
            | |||||||||||||| |||||||||||||||||||||||||||||||||||||||||||
Sbjct  474  TCTACAAATGGATAAGACTTTGGTCTGATTGTATAGGAGTTTTTGAACTAAAAAAGGAGC  415

Query  122  AATAAC-GCCCTCTTGATAAAACAAGAGGGAAGCTATTGCTCCttttttttATTTAGTAC  180
            |||| |  |||||||| |  | ||| ||||  | |||||||||||||||| |||||||||
Sbjct  414  AATAGCTTCCCTCTTGTTTTATCAATAGGG-CGTTATTGCTCCTTTTTTT-ATTTAGTAC  357

Query  181  TATTTGCCTTACATAGTTTCTTTAAAAATAACAAGTACTTTTTATAGTTTGGTTCGATTT  240
            ||||||||||| ||||||||||||||||||||||| ||||||||||||||||||||| ||
Sbjct  356  TATTTGCCTTATATAGTTTCTTTAAAAATAACAAGGACTTTTTATAGTTTGGTTCGAGTT  297

Query  241  GCGTGttttttctttgtattcatttatattataggtttctatatcatatatttttcccaa  300
            |||||||||||||||||||||||||||||||||||||||||||| ||||| |||||||||
Sbjct  296  GCGTGTTTTTTCTTTGTATTCATTTATATTATAGGTTTCTATATAATATACTTTTCCCAA  237

Query  301  tcttttatgaagttttatttACAATTTAATTTCAATCGAAAATAGAGAAAAATGACAATT  360
            |||||||||||||||||||| ||||| |||||||||| |||||||| |||||| | ||||
Sbjct  236  TCTTTTATGAAGTTTTATTTCCAATTCAATTTCAATCTAAAATAGATAAAAATTAAAATT  177

Query  361  TTGCTT--TT-TGA------TTTATATTGATTAAATCAAAATAAGAAAGAATATGCtttt  411
            || |||  || | |      ||||| || ||||||| || | |||||| |||||||||||
Sbjct  176  TTTCTTATTTATTACTTTTCTTTATCTTTATTAAAT-AAGA-AAGAAATAATATGCTTTT  119

Query  412  tttATGTTAATGGAAAAATATAGTAATACTAGATAATCGTAGAGGGGCGGATGTAGCCAA  471
            || |||||||||||||||||||||||||||||||||| ||||||||||||||||||||||
Sbjct  118  TT-ATGTTAATGGAAAAATATAGTAATACTAGATAATAGTAGAGGGGCGGATGTAGCCAA  60
```

```
Query  472  GTGGATCAAGGCAGTGGATTGTGAATCCACCATGCGCGG  510
            |||||||||||||||||||||||||||||||||||||||
Sbjct  59   GTGGATCAAGGCAGTGGATTGTGAATCCACCATGCGCGG  21


 Score = 104 bits (56),  Expect = 1e-17
 Identities = 58/59 (98%), Gaps = 0/59 (0%)
 Strand=Plus/Plus

Query  111  AAAAAAGGAGCAATAACGCCCTCTTGATAAAACAAGAGGGAAGCTATTGCTCCtttttt  169
            |||||||||||||||||||||| ||||||||||||||||||||||||||||||||||||
Sbjct  367  AAAAAAGGAGCAATAACGCCCTATTGATAAAACAAGAGGGAAGCTATTGCTCCTTTTTT  425


>KF746373.1 Helianthus decapetalus isolate DB28 plastid, complete genome
Length=151040

 Score = 701 bits (379),  Expect = 0.0
 Identities = 483/529 (91%), Gaps = 23/529 (4%)
 Strand=Plus/Minus

Query  2    GTTATGCATGAACGTAATGCTCATAATTTCCCTCTAGACTTAGCTGCTATTGAAGCTCCA  61
            ||||||||||||||||||||||||||||||||||||||||||||||||||||||||||||
Sbjct  547  GTTATGCATGAACGTAATGCTCATAATTTCCCTCTAGACTTAGCTGCTATTGAAGCTCCA  488

Query  62   TATACAAATGGATAAGCCTTTGGTCTGATTGTATAGGAGTTTTTGAACTAAAAAAGGAGC  121
            | |||||||||||||| |||||||||||||||||||||||||||||||||||||||||||
Sbjct  487  TCTACAAATGGATAAGACTTTGGTCTGATTGTATAGGAGTTTTTGAACTAAAAAAGGAGC  428

Query  122  AATAAC-GCCCTCTTGATAAAACAAGAGGGAAGCTATTGCTCCttttttttATTTAGTAC  180
            |||| |  ||||||||||||||||||||||  | ||||||||||||||||||||||||||
Sbjct  427  AATAGCTTCCCTCTTGATAAAACAAGAGGG-CGTTATTGCTCCTTTTTTTTATTTAGTAC  369

Query  181  TATTTGCCTTACATAGTTTCTTTAAA-------AATAACAAGTACTTTTTATAGTTTGGT  233
            ||||||||||||||||||||||||||       |||||||||||||||||||||||||||
Sbjct  368  TATTTGCCTTACATAGTTTCTTTAAACTTTAAAAATAACAAGTACTTTTTATAGTTTGGT  309

Query  234  TCGATTTGCGTGttttttctttgtattcatttatattataggtttctatatcatata---  290
            ||||||||  |||||||  ||||||||||||||||||||||||||||||||||||||
Sbjct  308  TCGATTTGTTTGTTTTT-ATTTGTATTCATTTATATTATAGGTTTCTATATCATATACTT  250

Query  291  -t--ttttcccaatcttttatgaagtttt---atttACAATTTAATTTCAATCGAAAATA  344
             |  |||||| ||||||||||||||||||    |||||||||||| |||||| |||||||
Sbjct  249  TTACTTTTCCTAATCTTTTATGAAGTTTTTTTTTTTTACAATTTAAATTCAATTGAAAATA  190

Query  345  GAGAAAAATGACAATTTTGCTT--TT-TGATTTATATTGATTAAATCAAAATAAGAAAGA  401
            || |||||||| ||||||||||  || | | || |||| ||| ||| ||||| |||||||
Sbjct  189  GATAAAAATGAAAATTTTGCTTATTTATTACTTTTATTTATTCAATAAAAATGAGAAAGA  130

Query  402  ATATGCtttttttATGTTAATGGAAAAATATAGTAATACTAGATAATCGTAGAGGGGCGG  461
            |||||||||||| |||||||||||||||||||||||||||||||||| ||||||||||||
Sbjct  129  ATATGCTTTTTT-ATGTTAATGGAAAAATATAGTAATACTAGATAATAGTAGAGGGGCGG  71

Query  462  ATGTAGCCAAGTGGATCAAGGCAGTGGATTGTGAATCCACCATGCGCGG  510
            |||||||||||||||||||||||||||||||||||||||||||||||||
Sbjct  70   ATGTAGCCAAGTGGATCAAGGCAGTGGATTGTGAATCCACCATGCGCGG  22
```

```
  Database: Core nucleotide BLAST database
    Posted date:  Sep 11, 2025  11:25 PM
  Number of letters in database: 923,306,297,115
  Number of sequences in database:  118,282,385

Lambda      K        H
    1.33    0.621     1.12
Gapped
Lambda      K        H
    1.28    0.460    0.850
Matrix: blastn matrix:1 -2
Gap Penalties: Existence: 0, Extension: 0
Number of Sequences: 118282385
Number of Hits to DB: 0
Number of extensions: 0
Number of successful extensions: 0
Number of sequences better than 0.05: 4068
Number of HSP's better than 0.05 without gapping: 0
Number of HSP's gapped: 5530
Number of HSP's successfully gapped: 5493
Length of query: 510
Length of database: 923306297115
Length adjustment: 36
Effective length of query: 474
Effective length of database: 919048131255
Effective search space: 435628814214870
Effective search space used: 435628814214870
A: 0
X1: 13 (25.0 bits)
X2: 32 (59.1 bits)
X3: 54 (99.7 bits)
S1: 13 (25.1 bits)
S2: 29 (54.7 bits)
```
