# Supplementary material for: Organ-Resolved Salicylate–Phenylpropanoid Metabolite Profiling of Echinacea purpurea, E. pallida, and E. tennesseensis Tissues Extracted with 50% Aqueous Methanol by LC–MS/MS and LC–HRMS
Source: Molecules. 2026 Aug 25;31(17):2963. doi: 10.3390/molecules31172963 (PMC13567413; doi:10.3390/molecules31172963)
Supplement: Supplementary file 1 [file molecules-31-02963-s001.zip › SupplementaryFiles/Supplementary Fig S2.pdf]

Plant Identification Report
Lot: 119661250903-1 Echinacea angustifolia leaves

Date reported: 17-Sep-25

Reported by: mdu

**Call:**	**Best matches *Echinacea tennesseensis* or *Echinacea speciosa*.**
**Comments:**	This sample from dried plant leaves. *Echinacea angustifolia* was also identified with generic NS primers as the only *Echinacea* species (results not shown); however, GenBank has low amounts of full 18S accessions for other *Echinacea* species and therefore this marker cannot be relied on for ID.
**Instrument name:**	SimpliAmp; SeqStudio
**Primer(s) used/Sequenced by:**	Generic trnH-psbA primers/mdu
**Extraction Method/Extracted by:**	RIDA kit/mdu
**DNAseq Run File:**	Seq25091501

```
BLASTN 2.17.0+
Reference: Zheng Zhang, Scott Schwartz, Lukas Wagner, and
Webb Miller (2000), "A greedy algorithm for aligning DNA
sequences", J Comput Biol 2000; 7(1-2):203-14.


Reference for database indexing: Aleksandr Morgulis, George
Coulouris, Yan Raytselis, Thomas L. Madden, Richa Agarwala,
Alejandro A. Schaffer (2008), "Database Indexing for
Production MegaBLAST Searches", Bioinformatics 24:1757-1764.


RID: CPAZY2N4015


Database: Core nucleotide BLAST database
           118,456,123 sequences; 925,171,125,281 total letters
Query=
Length=301
```

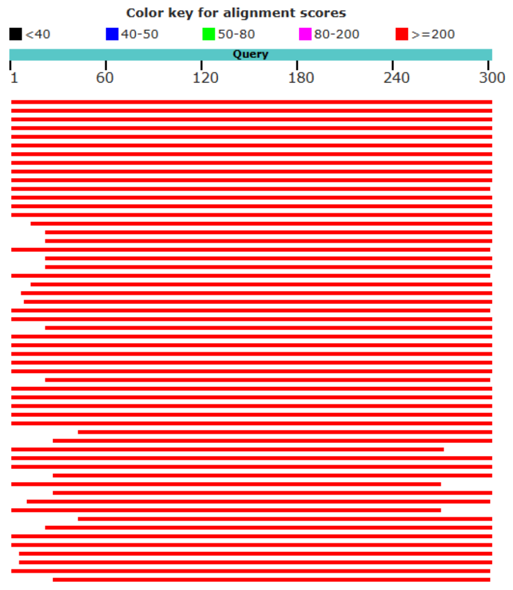

Sequences producing significant alignments:	Score (Bits)	E Value	Max Ident
**NC_034326.1 Echinacea tennesseensis plastid, complete genome**	**555**	**2e-153**	**100%**
**NC_034325.1 Echinacea speciosa plastid, complete genome**	**555**	**2e-153**	**100%**
EU785934.1 Echinacea purpurea PsbA (psbA) gene, partial cds; p...	549	9e-152	100%
ON641367.1 Echinacea purpurea isolate BPTPS168 voucher LISE:96...	549	9e-152	100%
EU785933.1 Echinacea angustifolia PsbA (psbA) gene, partial cd...	547	3e-151	100%
NC_034327.1 Echinacea purpurea plastid, complete genome	547	3e-151	100%
MK055334.1 Echinacea purpurea plastid, complete genome	545	1e-150	99%
NC_034322.1 Echinacea laevigata plastid, complete genome	542	1e-149	99%
EU785935.1 Echinacea pallida PsbA (psbA) gene, partial cds; ps...	542	1e-149	99%
NC_034321.1 Echinacea pallida plastid, complete genome	542	1e-149	99%
NC_034328.1 Echinacea sanguinea plastid, complete genome	540	5e-149	99%
NC_034320.1 Echinacea paradoxa plastid, complete genome	536	7e-148	99%
NC_034323.1 Echinacea atrorubens plastid, complete genome	536	7e-148	99%
NC_034324.1 Echinacea angustifolia plastid, complete genome	531	3e-146	99%
LS999860.1 Echinacea pallida DNA containing psbA-trnH IGS, spe...	520	7e-143	99%
MF348385.1 Echinacea purpurea isolate AD0EG44 photosystem II p...	516	9e-142	100%
MF348589.1 Echinacea purpurea isolate AD3HL50 photosystem II p...	516	9e-142	100%
NC_081940.1 Enydra sessilis chloroplast, complete genome	514	3e-141	98%
MF348644.1 Echinacea pallida isolate AD3HJ47 photosystem II pr...	503	7e-138	99%
MF348389.1 Echinacea pallida isolate AD0EE57 photosystem II pr...	503	7e-138	99%
NC_082051.1 Gaillardia pulchella chloroplast, complete sequence	496	1e-135	97%
MZ324674.1 Hulsea algida voucher Baldwin 678 (DAV) photosystem...	492	2e-134	98%
KF241798.1 Ambrosia artemisiifolia isolate AZ-1 PsbA (psbA) ge...	490	5e-134	97%
OR465018.1 Silphium perfoliatum clone 1072700_5 psbA-trnH inte...	490	5e-134	97%
MH464287.1 Balduina angustifolia voucher FLAS 234578 psbA-trnH...	488	2e-133	96%
NC_046787.1 Galinsoga parviflora voucher GPARV20171209 chlorop...	488	2e-133	96%
MF348803.1 Engelmannia peristenia isolate AG2KK77 photosystem ...	486	7e-133	98%
NC_081944.1 Venegasia carpesioides voucher US Weitzman et al. ...	483	9e-132	96%
OZ286192.1 Venegasia carpesioides genome assembly, organelle: ...	483	9e-132	96%
NC_031853.1 Galinsoga quadriradiata voucher GQUAD20160711 chlo...	483	9e-132	96%
NC_068130.1 Silphium integrifolium chloroplast, complete genome	483	9e-132	96%
NC_060408.1 Silphium perfoliatum chloroplast, complete sequence	483	9e-132	96%
OK469451.1 Jefea brevifolia voucher BRIT:Gostel611 photosystem...	481	3e-131	98%
MG019037.1 Ambrosia artemisiifolia chloroplast, complete genome	481	3e-131	96%
AB427162.1 Ambrosia artemisiifolia chloroplast psbA gene for D...	479	1e-130	96%
NC_035875.1 Ambrosia artemisiifolia chloroplast, complete genome	479	1e-130	96%
EF590669.1 Ambrosia artemisiifolia voucher NMNH Wells 4495 Psb...	479	1e-130	96%
NC_036810.2 Ambrosia trifida chloroplast, complete genome	479	1e-130	96%
MF037493.1 Echinacea paradoxa voucher Rebman1085 trnH-psbA int...	477	4e-130	100%
AY215540.1 Engelmannia peristenia voucher Clevinger 376 (TEX) ...	477	4e-130	98%
NC_054222.1 Xanthium spinosum chloroplast, complete genome	475	2e-129	99%
MT793838.1 Mikania lehmannii chloroplast, complete genome	475	2e-129	95%
MT793855.1 Mikania sylvatica chloroplast, complete genome	475	2e-129	95%
AY215509.1 Alepidocline annua voucher Panero 3109 (TEX) psbA g...	473	6e-129	98%
OZ286201.1 Guardiola tulocarpus genome assembly, organelle: pl...	472	2e-128	99%
AY215627.1 Zaluzania megacephala voucher Panero 2249 (TEX) psb...	472	2e-128	98%
GU818385.1 Helenium autumnale isolate 1146 PsbA (psbA) gene, p...	472	2e-128	96%
NC_081936.1 Guardiola tulocarpus voucher TEX Panero 3062 (TEX)...	472	2e-128	99%
MF037490.1 Echinacea angustifolia voucher Rolfsmeier19250 trnH...	470	7e-128	100%
OK469409.1 Eupatorieae sp. Gostel 807 voucher BRIT:Gostel807 p...	470	7e-128	97%
MZ292978.1 Acmella paniculata chloroplast, complete genome	470	7e-128	95%
NC_081937.1 Greenmaniella resinosa voucher Baldwin 977 (UC) ch...	470	7e-128	95%
KF241797.1 Ambrosia artemisiifolia isolate PH-53 PsbA (psbA) g...	470	7e-128	96%

KF241793.1 Ambrosia artemisiifolia isolate NJI-7 PsbA (psbA) g...	470	7e-128	96%
GU818408.1 Madia sp. 1163 PsbA (psbA) gene, partial cds; psbA-...	468	3e-127	95%
AY215541.1 Enydra sessilis voucher Panero 3126 (TEX) psbA gene...	466	9e-127	97%
KX346912.1 Xanthium sibiricum voucher S-CEZi-04 PsbA (psbA) ge...	466	9e-127	98%
OQ832296.1 Xanthium strumarium voucher MONT:87396 photosystem ...	466	9e-127	97%
KX261083.1 Platyschkuhria integrifolia var. integrifolia isola...	466	9e-127	96%
KX261084.1 Platyschkuhria integrifolia var. integrifolia isola...	466	9e-127	96%
OQ404763.1 Ambrosia artemisiifolia trnH-psbA intergenic spacer...	466	9e-127	96%
NC_072298.1 Xanthium strumarium chloroplast, complete genome	466	9e-127	98%
PP639077.1 Verbesina alternifolia chloroplast, complete genome	466	9e-127	95%
NC_042232.1 Xanthium sibiricum chloroplast, complete genome	466	9e-127	98%
KF241794.1 Ambrosia artemisiifolia isolate mb-46 PsbA (psbA) g...	464	3e-126	96%
OQ434029.1 Ambrosia trifida clone 1039837_5 trnH-psbA intergen...	464	3e-126	96%
MF037494.1 Echinacea sanguinea voucher Bengtson9 trnH-psbA int...	464	3e-126	99%
KF241795.1 Ambrosia artemisiifolia isolate NJF-12 PsbA (psbA) ...	464	3e-126	96%
NC_080520.1 Synotis erythropappa chloroplast, complete genome	462	1e-125	98%
EF538052.1 Synotis nagensium PsbA (psbA) gene, partial cds; ps...	462	1e-125	98%
MF143709.1 Galinsoga parviflora voucher Li ZY, Xiang XG, Xu SZ...	462	1e-125	96%
NC_069230.1 Synotis nagensium chloroplast, complete genome	462	1e-125	98%
KF241812.1 Ambrosia artemisiifolia isolate AZ-2 PsbA (psbA) ge...	462	1e-125	96%
OZ186474.1 Xanthium orientale genome assembly, organelle: plas...	460	4e-125	97%
MZ636529.1 Xanthium spinosum chloroplast, complete genome	460	4e-125	97%
MK836182.1 Flaveria bidentis chloroplast, complete genome	460	4e-125	94%
KX346911.1 Xanthium sibiricum voucher S-CEZi-03 PsbA (psbA) ge...	460	4e-125	97%
NC_081947.1 Ianthopappus corymbosus voucher TEX Panero & Crozi...	460	4e-125	95%
NC_053284.1 Aldama kunthiana voucher voucher Silva s.n. (ESA 1...	459	2e-124	95%
NC_057179.1 Aldama tuberosa voucher Loeuille 719 chloroplast, ...	459	2e-124	95%
NC_057174.1 Aldama robusta voucher Bringel 985 chloroplast, co...	459	2e-124	95%
LS999859.1 Echinacea angustifolia DNA containing psbA-trnH IGS...	459	2e-124	100%
NC_053296.1 Aldama canescens voucher Schilling 17 chloroplast,...	459	2e-124	95%
NC_053279.1 Aldama filifolia voucher Loeuille 849 chloroplast,...	459	2e-124	95%
NC_053285.1 Aldama linearis voucher Schilling 70 chloroplast, ...	459	2e-124	95%
NC_057182.1 Aldama vernonioides voucher Magenta 460 chloroplas...	459	2e-124	95%
NC_057180.1 Aldama tukumanensis voucher Heiden 1837 chloroplas...	459	2e-124	95%
NC_053295.1 Aldama bracteata voucher Filartiga 15 chloroplast,...	459	2e-124	95%
NC_057175.1 Aldama rubra voucher Magenta 388 chloroplast, comp...	459	2e-124	95%
KX346910.1 Xanthium sibiricum voucher S-CEZi-02 PsbA (psbA) ge...	459	2e-124	97%
NC_053282.1 Aldama goyazii voucher Magenta 716 chloroplast, co...	459	2e-124	95%
NC_053286.1 Aldama macrorhiza voucher Magenta 476 chloroplast,...	459	2e-124	95%
NC_057173.1 Aldama revoluta voucher Loeuille 799 chloroplast, ...	459	2e-124	95%
NC_053281.1 Aldama gardneri voucher Filartiga 16 chloroplast, ...	459	2e-124	95%
NC_053288.1 Aldama nudibasilaris voucher Filartiga 1 chloropla...	459	2e-124	95%
NC_053298.1 Aldama trichophylla voucher Magenta 390 chloroplas...	459	2e-124	95%
NC_053293.1 Aldama aspilioides voucher Filartiga 18 chloroplas...	459	2e-124	95%
NC_053291.1 Aldama anchusifolia voucher Filartiga 8 chloroplas...	459	2e-124	95%
NC_053297.1 Aldama corumbensis voucher Loeuille 909 chloroplas...	459	2e-124	95%
AY215553.1 Helenium bigelovii voucher Baldwin 681 (DAV) psbA g...	459	2e-124	97%

ALIGNMENTS
>NC_034326.1 Echinacea tennesseensis plastid, complete genome
KX548223.1 Echinacea tennesseensis plastid, complete genome
Length=151877

Score = 555 bits (300), Expect = 2e-153
Identities = 300/300 (100%), Gaps = 0/300 (0%)
Strand=Plus/Minus

```
Query  2    TTATGCATGAACGTAATGCTCATAATTTCCCTCTAGACTTAGCTGCTATTGAAGCTCCAT  61
            ||||||||||||||||||||||||||||||||||||||||||||||||||||||||||||
Sbjct  533  TTATGCATGAACGTAATGCTCATAATTTCCCTCTAGACTTAGCTGCTATTGAAGCTCCAT  474

Query  62   CTACAAATGGATAAGCCTTTGGTCTGATTGTATAGGAGTTTTTGAACTAAAAAAGGAGCA  121
            ||||||||||||||||||||||||||||||||||||||||||||||||||||||||||||
Sbjct  473  CTACAAATGGATAAGCCTTTGGTCTGATTGTATAGGAGTTTTTGAACTAAAAAAGGAGCA  414

Query  122  ATAACGCCCTCTTGATAAAACAAGAGGGAAGCTATTGCTCCttttttttATTTAGTACTA  181
            ||||||||||||||||||||||||||||||||||||||||||||||||||||||||||||
Sbjct  413  ATAACGCCCTCTTGATAAAACAAGAGGGAAGCTATTGCTCCTTTTTTTTATTTAGTACTA  354

Query  182  TTTGCCTTACATAGTTTCTTTAAAAATAACAAGTACTTTTTATAGTTTGGTTCGATTTGC  241
            ||||||||||||||||||||||||||||||||||||||||||||||||||||||||||||
Sbjct  353  TTTGCCTTACATAGTTTCTTTAAAAATAACAAGTACTTTTTATAGTTTGGTTCGATTTGC  294

Query  242  GTGtttttctttgtattcatttatattataggtttctatatcatatattttcCCAATC  301
            ||||||||||||||||||||||||||||||||||||||||||||||||||||||||||||
Sbjct  293  GTGTTTTTCTTTGTATTCATTTATATTATAGGTTTCTATATCATATATTTTCCCAATC  234


>NC_034325.1 Echinacea speciosa plastid, complete genome
 KX548222.1 Echinacea speciosa plastid, complete genome
Length=151860

 Score = 555 bits (300),  Expect = 2e-153
 Identities = 300/300 (100%), Gaps = 0/300 (0%)
 Strand=Plus/Minus

Query  2    TTATGCATGAACGTAATGCTCATAATTTCCCTCTAGACTTAGCTGCTATTGAAGCTCCAT  61
            ||||||||||||||||||||||||||||||||||||||||||||||||||||||||||||
Sbjct  533  TTATGCATGAACGTAATGCTCATAATTTCCCTCTAGACTTAGCTGCTATTGAAGCTCCAT  474

Query  62   CTACAAATGGATAAGCCTTTGGTCTGATTGTATAGGAGTTTTTGAACTAAAAAAGGAGCA  121
            ||||||||||||||||||||||||||||||||||||||||||||||||||||||||||||
Sbjct  473  CTACAAATGGATAAGCCTTTGGTCTGATTGTATAGGAGTTTTTGAACTAAAAAAGGAGCA  414

Query  122  ATAACGCCCTCTTGATAAAACAAGAGGGAAGCTATTGCTCCttttttttATTTAGTACTA  181
            ||||||||||||||||||||||||||||||||||||||||||||||||||||||||||||
Sbjct  413  ATAACGCCCTCTTGATAAAACAAGAGGGAAGCTATTGCTCCTTTTTTTTATTTAGTACTA  354

Query  182  TTTGCCTTACATAGTTTCTTTAAAAATAACAAGTACTTTTTATAGTTTGGTTCGATTTGC  241
            ||||||||||||||||||||||||||||||||||||||||||||||||||||||||||||
Sbjct  353  TTTGCCTTACATAGTTTCTTTAAAAATAACAAGTACTTTTTATAGTTTGGTTCGATTTGC  294

Query  242  GTGtttttctttgtattcatttatattataggtttctatatcatatattttcCCAATC  301
            ||||||||||||||||||||||||||||||||||||||||||||||||||||||||||||
Sbjct  293  GTGTTTTTCTTTGTATTCATTTATATTATAGGTTTCTATATCATATATTTTCCCAATC  234


>EU785934.1 Echinacea purpurea PsbA (psbA) gene, partial cds; psbA-trnH intergenic
spacer, complete sequence; and tRNA-His (trnH) gene,
partial sequence; chloroplast
Length=508

 Score = 549 bits (297),  Expect = 9e-152
 Identities = 299/300 (99%), Gaps = 0/300 (0%)
```

```
 Strand=Plus/Plus

Query  2    TTATGCATGAACGTAATGCTCATAATTTCCCTCTAGACTTAGCTGCTATTGAAGCTCCAT  61
            ||||||||||||||||||||||||||||||||||||||||||||||||||||||||||||
Sbjct  2    TTATGCATGAACGTAATGCTCATAATTTCCCTCTAGACTTAGCTGCTATTGAAGCTCCAT  61

Query  62   CTACAAATGGATAAGCCTTTGGTCTGATTGTATAGGAGTTTTTGAACTAAAAAAGGAGCA  121
             |||||||||||||||||||||||||||||||||||||||||||||||||||||||||||
Sbjct  62   ATACAAATGGATAAGCCTTTGGTCTGATTGTATAGGAGTTTTTGAACTAAAAAAGGAGCA  121

Query  122  ATAACGCCCTCTTGATAAAACAAGAGGGAAGCTATTGCTCCttttttttATTTAGTACTA  181
            ||||||||||||||||||||||||||||||||||||||||||||||||||||||||||||
Sbjct  122  ATAACGCCCTCTTGATAAAACAAGAGGGAAGCTATTGCTCCTTTTTTTTATTTAGTACTA  181

Query  182  TTTGCCTTACATAGTTTCTTTAAAAATAACAAGTACTTTTTATAGTTTGGTTCGATTTGC  241
            ||||||||||||||||||||||||||||||||||||||||||||||||||||||||||||
Sbjct  182  TTTGCCTTACATAGTTTCTTTAAAAATAACAAGTACTTTTTATAGTTTGGTTCGATTTGC  241

Query  242  GTGtttttctttgtattcatttatattataggtttctatatcatatattttcCCAATC  301
            ||||||||||||||||||||||||||||||||||||||||||||||||||||||||||||
Sbjct  242  GTGTTTTTCTTTGTATTCATTTATATTATAGGTTTCTATATCATATATTTTTCCCAATC  301
```

>ON641367.1 Echinacea purpurea isolate BPTPS168 voucher LISE:96479 chloroplast, complete genome
Length=151924

```
 Score = 549 bits (297),  Expect = 9e-152
 Identities = 299/300 (99%), Gaps = 0/300 (0%)
 Strand=Plus/Plus

Query  2      TTATGCATGAACGTAATGCTCATAATTTCCCTCTAGACTTAGCTGCTATTGAAGCTCCAT  61
              ||||||||||||||||||||||||||||||||||||||||||||||||||||||||||||
Sbjct  83081  TTATGCATGAACGTAATGCTCATAATTTCCCTCTAGACTTAGCTGCTATTGAAGCTCCAT  83140

Query  62     CTACAAATGGATAAGCCTTTGGTCTGATTGTATAGGAGTTTTTGAACTAAAAAAGGAGCA  121
               |||||||||||||||||||||||||||||||||||||||||||||||||||||||||||
Sbjct  83141  ATACAAATGGATAAGCCTTTGGTCTGATTGTATAGGAGTTTTTGAACTAAAAAAGGAGCA  83200

Query  122    ATAACGCCCTCTTGATAAAACAAGAGGGAAGCTATTGCTCCttttttttATTTAGTACTA  181
              ||||||||||||||||||||||||||||||||||||||||||||||||||||||||||||
Sbjct  83201  ATAACGCCCTCTTGATAAAACAAGAGGGAAGCTATTGCTCCTTTTTTTTATTTAGTACTA  83260

Query  182    TTTGCCTTACATAGTTTCTTTAAAAATAACAAGTACTTTTTATAGTTTGGTTCGATTTGC  241
              ||||||||||||||||||||||||||||||||||||||||||||||||||||||||||||
Sbjct  83261  TTTGCCTTACATAGTTTCTTTAAAAATAACAAGTACTTTTTATAGTTTGGTTCGATTTGC  83320

Query  242    GTGtttttctttgtattcatttatattataggtttctatatcatatattttcCCAATC  301
              ||||||||||||||||||||||||||||||||||||||||||||||||||||||||||||
Sbjct  83321  GTGTTTTTCTTTGTATTCATTTATATTATAGGTTTCTATATCATATATTTTTCCCAATC  83380
```

>EU785933.1 Echinacea angustifolia PsbA (psbA) gene, partial cds; psbA-trnH intergenic spacer, complete sequence; and tRNA-His (trnH) gene, partial sequence; chloroplast
Length=506

```
 Score = 547 bits (296),  Expect = 3e-151
 Identities = 299/300 (99%), Gaps = 1/300 (0%)
 Strand=Plus/Plus

Query  2    TTATGCATGAACGTAATGCTCATAATTTCCCTCTAGACTTAGCTGCTATTGAAGCTCCAT  61
            ||||||||||||||||||||||||||||||||||||||||||||||||||||||||||||
Sbjct  2    TTATGCATGAACGTAATGCTCATAATTTCCCTCTAGACTTAGCTGCTATTGAAGCTCCAT  61

Query  62   CTACAAATGGATAAGCCTTTGGTCTGATTGTATAGGAGTTTTTGAACTAAAAAAGGAGCA  121
            ||||||||||||||||||||||||||||||||||||||||||||||||||||||||||||
Sbjct  62   CTACAAATGGATAAGCCTTTGGTCTGATTGTATAGGAGTTTTTGAACTAAAAAAGGAGCA  121

Query  122  ATAACGCCCTCTTGATAAAACAAGAGGGAAGCTATTGCTCCttttttttATTTAGTACTA  181
            ||||||||||||||||||||||||||||||||||||||||| ||||||||||||||||||
Sbjct  122  ATAACGCCCTCTTGATAAAACAAGAGGGAAGCTATTGCTCC-TTTTTTTATTTAGTACTA  180

Query  182  TTTGCCTTACATAGTTTCTTTAAAAATAACAAGTACTTTTTATAGTTTGGTTCGATTTGC  241
            ||||||||||||||||||||||||||||||||||||||||||||||||||||||||||||
Sbjct  181  TTTGCCTTACATAGTTTCTTTAAAAATAACAAGTACTTTTTATAGTTTGGTTCGATTTGC  240

Query  242  GTGtttttttctttgtattcatttatattataggtttctatatcatatatttttcCCAATC  301
            ||||||||||||||||||||||||||||||||||||||||||||||||||||||||||||
Sbjct  241  GTGTTTTTTCTTTGTATTCATTTATATTATAGGTTTCTATATCATATATTTTTCCCAATC  300


>NC_034327.1 Echinacea purpurea plastid, complete genome
 KX548224.1 Echinacea purpurea plastid, complete genome
Length=151913

 Score = 547 bits (296),  Expect = 3e-151
 Identities = 299/300 (99%), Gaps = 1/300 (0%)
 Strand=Plus/Minus

Query  2    TTATGCATGAACGTAATGCTCATAATTTCCCTCTAGACTTAGCTGCTATTGAAGCTCCAT  61
            ||||||||||||||||||||||||||||||||||||||||||||||||||||||||||||
Sbjct  524  TTATGCATGAACGTAATGCTCATAATTTCCCTCTAGACTTAGCTGCTATTGAAGCTCCAT  465

Query  62   CTACAAATGGATAAGCCTTTGGTCTGATTGTATAGGAGTTTTTGAACTAAAAAAGGAGCA  121
            ||||||||||||||||||||||||||||||||||||||||||||||||||||||||||||
Sbjct  464  CTACAAATGGATAAGCCTTTGGTCTGATTGTATAGGAGTTTTTGAACTAAAAAAGGAGCA  405

Query  122  ATAACGCCCTCTTGATAAAACAAGAGGGAAGCTATTGCTCCttttttttATTTAGTACTA  181
            ||||||||||||||||||||||||||||||||||||||||||||||||| ||||||||||
Sbjct  404  ATAACGCCCTCTTGATAAAACAAGAGGGAAGCTATTGCTCCTTTTTTT-ATTTAGTACTA  346

Query  182  TTTGCCTTACATAGTTTCTTTAAAAATAACAAGTACTTTTTATAGTTTGGTTCGATTTGC  241
            ||||||||||||||||||||||||||||||||||||||||||||||||||||||||||||
Sbjct  345  TTTGCCTTACATAGTTTCTTTAAAAATAACAAGTACTTTTTATAGTTTGGTTCGATTTGC  286

Query  242  GTGtttttttctttgtattcatttatattataggtttctatatcatatatttttcCCAATC  301
            ||||||||||||||||||||||||||||||||||||||||||||||||||||||||||||
Sbjct  285  GTGTTTTTTCTTTGTATTCATTTATATTATAGGTTTCTATATCATATATTTTTCCCAATC  226


>MK055334.1 Echinacea purpurea plastid, complete genome
Length=151948
```

```
 Score = 545 bits (295),  Expect = 1e-150
 Identities = 300/302 (99%), Gaps = 2/302 (1%)
 Strand=Plus/Minus

Query  2    TTATGCATGAACGTAATGCTCATAATTTCCCTCTAGACTTAGCTGCTATTGAAGCTCCAT  61
            ||||||||||||||||||||||||||||||||||||||||||||||||||||||||||||
Sbjct  535  TTATGCATGAACGTAATGCTCATAATTTCCCTCTAGACTTAGCTGCTATTGAAGCTCCAT  476

Query  62   CTACAAATGGATAAGCCTTTGGTCTGATTGTATAGGAGTTTTTGAACTAAAAAAGGAGCA  121
            ||||||||||||||||||||||||||||||||||||||||||||||||||||||||||||
Sbjct  475  CTACAAATGGATAAGCCTTTGGTCTGATTGTATAGGAGTTTTTGAACTAAAAAAGGAGCA  416

Query  122  ATAACGCCCTCTTGATAAAACAAGAGGGAAGCTATTGCTCCtttttttt-ATTTAGTACT  180
            ||||||||||||||||||||||||||||||||||||||||||||||||| ||||||||||
Sbjct  415  ATAACGCCCTCTTGATAAAACAAGAGGGAAGCTATTGCTCCTTTTTTTTTATTTAGTACT  356

Query  181  ATTTGCCTTACATAGTTTCTTTAAAAATAACAAGTACTTTTTATAGTTTGGTTCGATTTG  240
            ||||||||||||||||||||||||||||||||||||||||||||||||||||||||||||
Sbjct  355  ATTTGCCTTACATAGTTTCTTTAAAAATAACAAGTACTTTTTATAGTTTGGTTCGATTTG  296

Query  241  CGTGtttttt-ctttgtattcatttatattataggtttctatatcatatatttttcCCAA  299
            |||||||||| |||||||||||||||||||||||||||||||||||||||||||||||||
Sbjct  295  CGTGTTTTTTTCTTTGTATTCATTTATATTATAGGTTTCTATATCATATATTTTTCCCAA  236

Query  300  TC  301
            ||
Sbjct  235  TC  234


>NC_034322.1 Echinacea laevigata plastid, complete genome
 KX548219.1 Echinacea laevigata plastid, complete genome
Length=151886

 Score = 542 bits (293),  Expect = 1e-149
 Identities = 298/300 (99%), Gaps = 1/300 (0%)
 Strand=Plus/Minus

Query  2    TTATGCATGAACGTAATGCTCATAATTTCCCTCTAGACTTAGCTGCTATTGAAGCTCCAT  61
            ||||||||||||||||||||||||||||||||||||||||||||||||||||||||||||
Sbjct  536  TTATGCATGAACGTAATGCTCATAATTTCCCTCTAGACTTAGCTGCTATTGAAGCTCCAT  477

Query  62   CTACAAATGGATAAGCCTTTGGTCTGATTGTATAGGAGTTTTTGAACTAAAAAAGGAGCA  121
            ||||||||||||||||||||||||||||||||||||||||||||||||||||||||||||
Sbjct  476  CTACAAATGGATAAGCCTTTGGTCTGATTGTATAGGAGTTTTTGAACTAAAAAAGGAGCA  417

Query  122  ATAACGCCCTCTTGATAAAACAAGAGGGAAGCTATTGCTCCttttttttATTTAGTACTA  181
            ||||||||||||||||||||||||||||||||||||||||||||||||| |||||||||||
Sbjct  416  ATAACGCCCTCTTGATAAAACAAGAGGGAAGCTATTGCTCCTTTTTTTT-ATTTAGTACTA  358

Query  182  TTTGCCTTACATAGTTTCTTTAAAAATAACAAGTACTTTTTATAGTTTGGTTCGATTTGC  241
            ||||||||||||||||||||||||||||| ||||||||||||||||||||||||||||||
Sbjct  357  TTTGCCTTACATAGTTTCTTTAAAAATAATAAGTACTTTTTATAGTTTGGTTCGATTTGC  298

Query  242  GTGtttttctttgtattcatttatattataggtttctatatcatatatttttcCCAATC  301
            ||||||||||||||||||||||||||||||||||||||||||||||||||||||||||||
Sbjct  297  GTGTTTTTTCTTTGTATTCATTTATATTATAGGTTTCTATATCATATATTTTTCCCAATC  238
```

```
>EU785935.1 Echinacea pallida PsbA (psbA) gene, partial cds; psbA-trnH intergenic
spacer, complete sequence; and tRNA-His (trnH) gene,
partial sequence; chloroplast
Length=508

 Score = 542 bits (293),  Expect = 1e-149
 Identities = 298/300 (99%), Gaps = 1/300 (0%)
 Strand=Plus/Plus

Query  2    TTATGCATGAACGTAATGCTCATAATTTCCCTCTAGACTTAGCTGCTATTGAAGCTCCAT  61
            ||||||||||||||||||||||||||||||||||||||||||||||||||||||||||||
Sbjct  2    TTATGCATGAACGTAATGCTCATAATTTCCCTCTAGACTTAGCTGCTATTGAAGCTCCAT  61

Query  62   CTACAAATGGATAAGCCTTTGGTCTGATTGTATAGGAGTTTTTGAACTAAAAAAGGAGCA  121
            ||||||||||||||||||||||||||||||||||||||||||||||||||||||||||||
Sbjct  62   CTACAAATGGATAAGCCTTTGGTCTGATTGTATAGGAGTTTTTGAACTAAAAAAGGAGCA  121

Query  122  ATAACGCCCTCTTGATAAAACAAGAGGGAAGCTATTGCTCCttttttttATTTAGTACTA  181
            ||||||||||||||||||||||||||||||||||||||||| ||||||||||||||||||
Sbjct  122  ATAACGCCCTCTTGATAAAACAAGAGGGAAGCTATTGCTCC-TTTTTTTATTTAGTACTA  180

Query  182  TTTGCCTTACATAGTTTCTTTAAAAATAACAAGTACTTTTTATAGTTTGGTTCGATTTGC  241
            ||||||||||||||||||||||||||||||||||||||||||||||||||||||||||||
Sbjct  181  TTTGCCTTACATAGTTTCTTTAAAAATAACAAGTACTTTTTATAGTTTGGTTCGATTTGC  240

Query  242  GTGttttttctttgtattcatttatattataggtttctatatcatatatttttcCCAATC  301
            ||||||||||||||||||||||||||||||||||||||||||||| ||||||||||||||
Sbjct  241  GTGTTTTTTCTTTGTATTCATTTATATTATAGGTTTCTATATCATTTATTTTTCCCAATC  300


>NC_034321.1 Echinacea pallida plastid, complete genome
 KX548218.1 Echinacea pallida plastid, complete genome
Length=151883

 Score = 542 bits (293),  Expect = 1e-149
 Identities = 298/300 (99%), Gaps = 1/300 (0%)
 Strand=Plus/Minus

Query  2    TTATGCATGAACGTAATGCTCATAATTTCCCTCTAGACTTAGCTGCTATTGAAGCTCCAT  61
            ||||||||||||||||||||||||||||||||||||||||||||||||||||||||||||
Sbjct  533  TTATGCATGAACGTAATGCTCATAATTTCCCTCTAGACTTAGCTGCTATTGAAGCTCCAT  474

Query  62   CTACAAATGGATAAGCCTTTGGTCTGATTGTATAGGAGTTTTTGAACTAAAAAAGGAGCA  121
            ||||||||||||||||||||||||||||||||||||||||||||||||||||||||||||
Sbjct  473  CTACAAATGGATAAGCCTTTGGTCTGATTGTATAGGAGTTTTTGAACTAAAAAAGGAGCA  414

Query  122  ATAACGCCCTCTTGATAAAACAAGAGGGAAGCTATTGCTCCttttttttATTTAGTACTA  181
            |||||||||||||||||||||||||||||||||||||||||||||||| |||||||||||
Sbjct  413  ATAACGCCCTCTTGATAAAACAAGAGGGAAGCTATTGCTCCTTTTTTT-ATTTAGTACTA  355

Query  182  TTTGCCTTACATAGTTTCTTTAAAAATAACAAGTACTTTTTATAGTTTGGTTCGATTTGC  241
            ||||||||||||||||||||||||||||||||||||||||||||||||||||||||||||
Sbjct  354  TTTGCCTTACATAGTTTCTTTAAAAATAACAAGTACTTTTTATAGTTTGGTTCGATTTGC  295

Query  242  GTGttttttctttgtattcatttatattataggtttctatatcatatatttttcCCAATC  301
            ||||||||||||||||||||||||||||||||||||||||||||| ||||||||||||||
```

```
Sbjct  294  GTGTTTTTTCTTTGTATTCATTTATATTATAGGTTTCTATATCATTTATTTTTCCCAATC  235


>NC_034328.1 Echinacea sanguinea plastid, complete genome
 KX548225.1 Echinacea sanguinea plastid, complete genome
Length=151926

 Score = 540 bits (292),  Expect = 5e-149
 Identities = 297/299 (99%), Gaps = 1/299 (0%)
 Strand=Plus/Minus

Query  2    TTATGCATGAACGTAATGCTCATAATTTCCCTCTAGACTTAGCTGCTATTGAAGCTCCAT  61
            ||||||||||||||||||||||||||||||||||||||||||||||||||||||||||||
Sbjct  533  TTATGCATGAACGTAATGCTCATAATTTCCCTCTAGACTTAGCTGCTATTGAAGCTCCAT  474

Query  62   CTACAAATGGATAAGCCTTTGGTCTGATTGTATAGGAGTTTTTGAACTAAAAAAGGAGCA  121
            ||||||||||||||||||||||||||||||||||||||||||||||||||||||||||||
Sbjct  473  CTACAAATGGATAAGCCTTTGGTCTGATTGTATAGGAGTTTTTGAACTAAAAAAGGAGCA  414

Query  122  ATAACGCCCTCTTGATAAAACAAGAGGGAAGCTATTGCTCCttttttttATTTAGTACTA  181
            |||||||||||||||||||||||||||||||||||||||||||||||| |||||||||||
Sbjct  413  ATAACGCCCTCTTGATAAAACAAGAGGGAAGCTATTGCTCCTTTTTTT-ATTTAGTACTA  355

Query  182  TTTGCCTTACATAGTTTCTTTAAAAATAACAAGTACTTTTTATAGTTTGGTTCGATTTGC  241
            ||||||||||||||||||||||||||||||||||||||||||||||||||||||||||||
Sbjct  354  TTTGCCTTACATAGTTTCTTTAAAAATAACAAGTACTTTTTATAGTTTGGTTCGATTTGC  295

Query  242  GTGttttttctttgtattcatttatattataggtttctatatcatatatttttcCCAAT  300
            ||||||||||||||||||||||||||||||||||||||||||||| |||||||||||||
Sbjct  294  GTGTTTTTTCTTTGTATTCATTTATATTATAGGTTTCTATATCATTTATTTTTCCCAAT  236


>NC_034320.1 Echinacea paradoxa plastid, complete genome
 KX548217.1 Echinacea paradoxa plastid, complete genome
Length=151837

 Score = 536 bits (290),  Expect = 7e-148
 Identities = 297/300 (99%), Gaps = 1/300 (0%)
 Strand=Plus/Minus

Query  2    TTATGCATGAACGTAATGCTCATAATTTCCCTCTAGACTTAGCTGCTATTGAAGCTCCAT  61
            ||||||||||||||||||||||||||||||||||||||||||||||||||||||||||||
Sbjct  532  TTATGCATGAACGTAATGCTCATAATTTCCCTCTAGACTTAGCTGCTATTGAAGCTCCAT  473

Query  62   CTACAAATGGATAAGCCTTTGGTCTGATTGTATAGGAGTTTTTGAACTAAAAAAGGAGCA  121
            ||||||||||||||||||||||||||||||||||||||||||||||||||||||||||||
Sbjct  472  CTACAAATGGATAAGCCTTTGGTCTGATTGTATAGGAGTTTTTGAACTAAAAAAGGAGCA  413

Query  122  ATAACGCCCTCTTGATAAAACAAGAGGGAAGCTATTGCTCCttttttttATTTAGTACTA  181
            |||||| ||||||||||||||||||||||||||||||||||||||||| |||||||||||
Sbjct  412  ATAACGTCCTCTTGATAAAACAAGAGGGAAGCTATTGCTCCTTTTTTT-ATTTAGTACTA  354

Query  182  TTTGCCTTACATAGTTTCTTTAAAAATAACAAGTACTTTTTATAGTTTGGTTCGATTTGC  241
            ||||||||||||||||||||||||||||||||||||||||||||||||||||||||||||
Sbjct  353  TTTGCCTTACATAGTTTCTTTAAAAATAACAAGTACTTTTTATAGTTTGGTTCGATTTGC  294

Query  242  GTGttttttctttgtattcatttatattataggtttctatatcatatatttttcCCAATC  301
```

```
                |||||||||||||||||||||||||||||||||||||||||||||| ||||||||||||||
Sbjct  293    GTGTTTTTTCTTTGTATTCATTTATATTATAGGTTTCTATATCATTTATTTTTCCCAATC  234
```

```
>NC_034323.1 Echinacea atrorubens plastid, complete genome
 KX548220.1 Echinacea atrorubens plastid, complete genome
Length=151912

 Score = 536 bits (290),  Expect = 7e-148
 Identities = 297/300 (99%), Gaps = 1/300 (0%)
 Strand=Plus/Minus

Query  2      TTATGCATGAACGTAATGCTCATAATTTCCCTCTAGACTTAGCTGCTATTGAAGCTCCAT  61
              ||||||||||||||||||||||||||||||||||||||||||||||||||||||||||||
Sbjct  532    TTATGCATGAACGTAATGCTCATAATTTCCCTCTAGACTTAGCTGCTATTGAAGCTCCAT  473

Query  62     CTACAAATGGATAAGCCTTTGGTCTGATTGTATAGGAGTTTTTGAACTAAAAAAGGAGCA  121
              ||||||||||||||||||||||||||||||||||||||||||||||||||||||||||||
Sbjct  472    CTACAAATGGATAAGCCTTTGGTCTGATTGTATAGGAGTTTTTGAACTAAAAAAGGAGCA  413

Query  122    ATAACGCCCTCTTGATAAAACAAGAGGGAAGCTATTGCTCCttttttttATTTAGTACTA  181
              |||||| ||||||||||||||||||||||||||||||||||||||||| |||||||||||
Sbjct  412    ATAACGTCCTCTTGATAAAACAAGAGGGAAGCTATTGCTCCTTTTTTT-ATTTAGTACTA  354

Query  182    TTTGCCTTACATAGTTTCTTTAAAAATAACAAGTACTTTTTATAGTTTGGTTCGATTTGC  241
              ||||||||||||||||||||||||||||||||||||||||||||||||||||||||||||
Sbjct  353    TTTGCCTTACATAGTTTCTTTAAAAATAACAAGTACTTTTTATAGTTTGGTTCGATTTGC  294

Query  242    GTGttttttctttgtattcatttatattataggtttctatatcatatatttttcCCAATC  301
              ||||||||||||||||||||||||||||||||||||||||||||| ||||||||||||||
Sbjct  293    GTGTTTTTTCTTTGTATTCATTTATATTATAGGTTTCTATATCATTTATTTTTCCCAATC  234


>NC_034324.1 Echinacea angustifolia plastid, complete genome
 KX548221.1 Echinacea angustifolia plastid, complete genome
Length=151935

 Score = 531 bits (287),  Expect = 3e-146
 Identities = 296/300 (99%), Gaps = 2/300 (1%)
 Strand=Plus/Minus

Query  2      TTATGCATGAACGTAATGCTCATAATTTCCCTCTAGACTTAGCTGCTATTGAAGCTCCAT  61
              ||||||||||||||||||||||||||||||||||||||||||||||||||||||||||||
Sbjct  531    TTATGCATGAACGTAATGCTCATAATTTCCCTCTAGACTTAGCTGCTATTGAAGCTCCAT  472

Query  62     CTACAAATGGATAAGCCTTTGGTCTGATTGTATAGGAGTTTTTGAACTAAAAAAGGAGCA  121
              ||||||||||||||||||||||||||||||||||||||||||||||||||||||||||||
Sbjct  471    CTACAAATGGATAAGCCTTTGGTCTGATTGTATAGGAGTTTTTGAACTAAAAAAGGAGCA  412

Query  122    ATAACGCCCTCTTGATAAAACAAGAGGGAAGCTATTGCTCCttttttttATTTAGTACTA  181
              |||| |||||||||||||||||||||| |||||||||||||||||||| |||||||||||
Sbjct  411    ATAATGCCCTCTTGATAAAACAAGAGGAAAGCTATTGCTCCTTTTTTT-ATTTAGTACTA  353

Query  182    TTTGCCTTACATAGTTTCTTTAAAAATAACAAGTACTTTTTATAGTTTGGTTCGATTTGC  241
              ||||||||||||||||||||||||||||||||||||||||||||||||||||||||||||
Sbjct  352    TTTGCCTTACATAGTTTCTTTAAAAATAACAAGTACTTTTTATAGTTTGGTTCGATTTGC  293
```

```
Query  242   GTGtttttctttgtattcatttatattataggtttctatatcatatatttttcCCAATC  301
             |||||||| ||||||||||||||||||||||||||||||||||||||||||||||||||||
Sbjct  292   GTGTTTTT-CTTTGTATTCATTTATATTATAGGTTTCTATATCATATATTTTTCCCAATC  234


>LS999860.1 Echinacea pallida DNA containing psbA-trnH IGS, specimen voucher
FEM_DBE_018
Length=384

 Score = 520 bits (281),  Expect = 7e-143
 Identities = 286/288 (99%), Gaps = 1/288 (0%)
 Strand=Plus/Plus

Query  14    GTAATGCTCATAATTTCCCTCTAGACTTAGCTGCTATTGAAGCTCCATCTACAAATGGAT  73
             ||||||||||||||||||||||||||||||||||||||||||||||||||||||||||||
Sbjct  1     GTAATGCTCATAATTTCCCTCTAGACTTAGCTGCTATTGAAGCTCCATCTACAAATGGAT  60

Query  74    AAGCCTTTGGTCTGATTGTATAGGAGTTTTTGAACTAAAAAAGGAGCAATAACGCCCTCT  133
             ||||||||||||||||||||||||||||||||||||||||||||||||||||||||||||
Sbjct  61    AAGCCTTTGGTCTGATTGTATAGGAGTTTTTGAACTAAAAAAGGAGCAATAACGCCCTCT  120

Query  134   TGATAAAACAAGAGGGAAGCTATTGCTCCttttttttATTTAGTACTATTTGCCTTACAT  193
             ||||||||||||||||||||||||||||| ||||||||||||||||||||||||||||||
Sbjct  121   TGATAAAACAAGAGGGAAGCTATTGCTCC-TTTTTTTATTTAGTACTATTTGCCTTACAT  179

Query  194   AGTTTCTTTAAAAATAACAAGTACTTTTTATAGTTTGGTTCGATTTGCGTGtttttctt  253
             |||||||||||||||||||||||||||||||||||||||||||||||||||||||||||
Sbjct  180   AGTTTCTTTAAAAATAACAAGTACTTTTTATAGTTTGGTTCGATTTGCGTGTTTTTCTT  239

Query  254   tgtattcatttatattataggtttctatatcatatatttttcCCAATC  301
             |||||||||||||||||||||||||||||||||| |||||||||||||
Sbjct  240   TGTATTCATTTATATTATAGGTTTCTATATCATTTATTTTTCCCAATC  287


>MF348385.1 Echinacea purpurea isolate AD0EG44 photosystem II protein D1
(psbA) gene, partial cds; and psbA-trnH intergenic spacer, partial
sequence; chloroplast
Length=485

 Score = 516 bits (279),  Expect = 9e-142
 Identities = 279/279 (100%), Gaps = 0/279 (0%)
 Strand=Plus/Plus

Query  23    ATAATTTCCCTCTAGACTTAGCTGCTATTGAAGCTCCATCTACAAATGGATAAGCCTTTG  82
             ||||||||||||||||||||||||||||||||||||||||||||||||||||||||||||
Sbjct  1     ATAATTTCCCTCTAGACTTAGCTGCTATTGAAGCTCCATCTACAAATGGATAAGCCTTTG  60

Query  83    GTCTGATTGTATAGGAGTTTTTGAACTAAAAAAGGAGCAATAACGCCCTCTTGATAAAAC  142
             ||||||||||||||||||||||||||||||||||||||||||||||||||||||||||||
Sbjct  61    GTCTGATTGTATAGGAGTTTTTGAACTAAAAAAGGAGCAATAACGCCCTCTTGATAAAAC  120

Query  143   AAGAGGGAAGCTATTGCTCCttttttttATTTAGTACTATTTGCCTTACATAGTTTCTTT  202
             ||||||||||||||||||||||||||||||||||||||||||||||||||||||||||||
Sbjct  121   AAGAGGGAAGCTATTGCTCCTTTTTTTTATTTAGTACTATTTGCCTTACATAGTTTCTTT  180

Query  203   AAAAATAACAAGTACTTTTTATAGTTTGGTTCGATTTGCGTGtttttctttgtattcat  262
             |||||||||||||||||||||||||||||||||||||||||||||||||||||||||||
```

```
Sbjct  181  AAAAATAACAAGTACTTTTTATAGTTTGGTTCGATTTGCGTGTTTTTTCTTTGTATTCAT  240

Query  263  ttatattataggtttctatatcatatattttttcCCAATC  301
            |||||||||||||||||||||||||||||||||||||||
Sbjct  241  TTATATTATAGGTTTCTATATCATATATTTTTCCCAATC  279
```

>MF348589.1 Echinacea purpurea isolate AD3HL50 photosystem II protein D1 (psbA) gene, partial cds; and psbA-trnH intergenic spacer, partial sequence; chloroplast
Length=485

```
 Score = 516 bits (279),  Expect = 9e-142
 Identities = 279/279 (100%), Gaps = 0/279 (0%)
 Strand=Plus/Plus

Query  23   ATAATTTCCCTCTAGACTTAGCTGCTATTGAAGCTCCATCTACAAATGGATAAGCCTTTG  82
            ||||||||||||||||||||||||||||||||||||||||||||||||||||||||||||
Sbjct  1    ATAATTTCCCTCTAGACTTAGCTGCTATTGAAGCTCCATCTACAAATGGATAAGCCTTTG  60

Query  83   GTCTGATTGTATAGGAGTTTTTGAACTAAAAAAGGAGCAATAACGCCCCTCTTGATAAAAC  142
            ||||||||||||||||||||||||||||||||||||||||||||||||||||||||||||
Sbjct  61   GTCTGATTGTATAGGAGTTTTTGAACTAAAAAAGGAGCAATAACGCCCCTCTTGATAAAAC  120

Query  143  AAGAGGGAAGCTATTGCTCCttttttttATTTAGTACTATTTGCCTTACATAGTTTCTTT  202
            ||||||||||||||||||||||||||||||||||||||||||||||||||||||||||||
Sbjct  121  AAGAGGGAAGCTATTGCTCCTTTTTTTTATTTAGTACTATTTGCCTTACATAGTTTCTTT  180

Query  203  AAAAATAACAAGTACTTTTTATAGTTTGGTTCGATTTGCGTGttttttctttgtattcat  262
            ||||||||||||||||||||||||||||||||||||||||||||||||||||||||||||
Sbjct  181  AAAAATAACAAGTACTTTTTATAGTTTGGTTCGATTTGCGTGTTTTTTCTTTGTATTCAT  240

Query  263  ttatattataggtttctatatcatatattttttcCCAATC  301
            |||||||||||||||||||||||||||||||||||||||
Sbjct  241  TTATATTATAGGTTTCTATATCATATATTTTTCCCAATC  279
```

>NC_081940.1 Enydra sessilis chloroplast, complete genome
 OR069728.1 Enydra sessilis chloroplast, complete genome
Length=151340

```
 Score = 514 bits (278),  Expect = 3e-141
 Identities = 293/300 (98%), Gaps = 1/300 (0%)
 Strand=Plus/Minus

Query  2    TTATGCATGAACGTAATGCTCATAATTTCCCTCTAGACTTAGCTGCTATTGAAGCTCCAT  61
            ||||||||||||||||||||||||||||||||||||||||||||||||||||||||||||
Sbjct  547  TTATGCATGAACGTAATGCTCATAATTTCCCTCTAGACTTAGCTGCTATTGAAGCTCCAT  488

Query  62   CTACAAATGGATAAGCCTTTGGTCTGATTGTATAGGAGTTTTTGAACTAAAAAAGGAGCA  121
            ||||||||||||||| ||||||||||||||||||||||||||||||||||||||||||||
Sbjct  487  CTACAAATGGATAAGACTTTGGTCTGATTGTATAGGAGTTTTTGAACTAAAAAAGGAGCA  428

Query  122  ATAACGCCCCTCTTGATAAAACAAGAGGGAAGCTATTGCTCCttttttttATTTAGTACTA  181
            ||||||||||||||||||||||||||||||||||||||||||||||||||||||||||||
Sbjct  427  ATAACGCCCCTCTTGATAAAACAAGAGGGAAGCTATTGCTCCTTTTTTTTATTTAGTACTA  368
```

```
Query  182  TTTGCCTTACATAGTTTCTTTAAAAATAACAAGTACTTTTTATAGTTTGGTTCGATTTGC  241
            ||||||||||||||||||||||||||||||||| ||||||| ||||||||||||||||||
Sbjct  367  TTTGCCTTACATAGTTTCTTTAAAAATAACAAGGACTTTTTCTAGTTTGGTTCGATTTGC  308

Query  242  GTGttttttctttgtattcatttatattataggtttctatatcatatatttttcCC-AAT  300
            ||||||| ||||||||||||||||||||||||||||||||||||| || ||||||| |||
Sbjct  307  GTGTTTTATCTTTGTATTCATTTATATTATAGGTTTCTATATCATTTACTTTTCCCCAAT  248


>MF348644.1 Echinacea pallida isolate AD3HJ47 photosystem II protein D1 (psbA)
gene, partial cds; and psbA-trnH intergenic spacer, partial
sequence; chloroplast
Length=466

 Score = 503 bits (272),  Expect = 7e-138
 Identities = 277/279 (99%), Gaps = 1/279 (0%)
 Strand=Plus/Plus

Query  23   ATAATTTCCCTCTAGACTTAGCTGCTATTGAAGCTCCATCTACAAATGGATAAGCCTTTG  82
            ||||||||||||||||||||||||||||||||||||||||||||||||||||||||||||
Sbjct  1    ATAATTTCCCTCTAGACTTAGCTGCTATTGAAGCTCCATCTACAAATGGATAAGCCTTTG  60

Query  83   GTCTGATTGTATAGGAGTTTTTGAACTAAAAAAGGAGCAATAACGCCCTCTTGATAAAAC  142
            ||||||||||||||||||||||||||||||||||||||||||||||||||||||||||||
Sbjct  61   GTCTGATTGTATAGGAGTTTTTGAACTAAAAAAGGAGCAATAACGCCCTCTTGATAAAAC  120

Query  143  AAGAGGGAAGCTATTGCTCCttttttttATTTAGTACTATTTGCCTTACATAGTTTCTTT  202
            |||||||||||||||||||| |||||||||||||||||||||||||||||||||||||||
Sbjct  121  AAGAGGGAAGCTATTGCTCC-TTTTTTTATTTAGTACTATTTGCCTTACATAGTTTCTTT  179

Query  203  AAAAATAACAAGTACTTTTTATAGTTTGGTTCGATTTGCGTGtttttctttgtattcat  262
            |||||||| |||||||||||||||||||||||||||||||||||||||||||||||||||
Sbjct  180  AAAAATAATAAGTACTTTTTATAGTTTGGTTCGATTTGCGTGTTTTTTCTTTGTATTCAT  239

Query  263  ttatattataggtttctatatcatatatttttcCCAATC  301
            |||||||||||||||||||||||||||||||||||||||
Sbjct  240  TTATATTATAGGTTTCTATATCATATATTTTTCCCAATC  278


>MF348389.1 Echinacea pallida isolate AD0EE57 photosystem II protein D1 (psbA)
gene, partial cds; and psbA-trnH intergenic spacer, partial
sequence; chloroplast
Length=466

 Score = 503 bits (272),  Expect = 7e-138
 Identities = 277/279 (99%), Gaps = 1/279 (0%)
 Strand=Plus/Plus

Query  23   ATAATTTCCCTCTAGACTTAGCTGCTATTGAAGCTCCATCTACAAATGGATAAGCCTTTG  82
            ||||||||||||||||||||||||||||||||||||||||||||||||||||||||||||
Sbjct  1    ATAATTTCCCTCTAGACTTAGCTGCTATTGAAGCTCCATCTACAAATGGATAAGCCTTTG  60

Query  83   GTCTGATTGTATAGGAGTTTTTGAACTAAAAAAGGAGCAATAACGCCCTCTTGATAAAAC  142
            ||||||||||||||||||||||||||||||||||||||||||||||||||||||||||||
Sbjct  61   GTCTGATTGTATAGGAGTTTTTGAACTAAAAAAGGAGCAATAACGCCCTCTTGATAAAAC  120

Query  143  AAGAGGGAAGCTATTGCTCCttttttttATTTAGTACTATTTGCCTTACATAGTTTCTTT  202
```

```
              |||||||||||||||||||| |||||||||||||||||||||||||||||||||||||||
Sbjct  121   AAGAGGGAAGCTATTGCTCC-TTTTTTTATTTAGTACTATTTGCCTTACATAGTTTCTTT  179

Query  203   AAAAATAACAAGTACTTTTTATAGTTTGGTTCGATTTGCGTgtttttctttgtattcat  262
              |||||||| |||||||||||||||||||||||||||||||||||||||||||||||||||
Sbjct  180   AAAAATAATAAGTACTTTTTATAGTTTGGTTCGATTTGCGTGTTTTTCTTTGTATTCAT  239

Query  263   ttatattataggtttctatatcatatattttcCCAATC  301
              |||||||||||||||||||||||||||||||||||||||
Sbjct  240   TTATATTATAGGTTTCTATATCATATATTTTTCCCAATC  278


>NC_082051.1 Gaillardia pulchella chloroplast, complete sequence
 OR124734.1 Gaillardia pulchella chloroplast, complete genome
Length=151778

 Score = 496 bits (268),  Expect = 1e-135
 Identities = 289/299 (97%), Gaps = 2/299 (1%)
 Strand=Plus/Minus

Query  2     TTATGCATGAACGTAATGCTCATAATTTCCCTCTAGACTTAGCTGCTATTGAAGCTCCAT  61
              ||||||||||||||||||||||||||||||||||||||||||||||||||||||||||||
Sbjct  533   TTATGCATGAACGTAATGCTCATAATTTCCCTCTAGACTTAGCTGCTATTGAAGCTCCAT  474

Query  62    CTACAAATGGATAAGCCTTTGGTCTGATTGTATAGGAGTTTTTGAACTAAAAAAGGAGCA  121
              ||||||||||||||| |||||||||||||||||| ||||||||||||||||||||||||||
Sbjct  473   CTACAAATGGATAAGACTTTGGTCTGATTGTATAAGAGTTTTTGAACTAAAAAAGGAGCA  414

Query  122   ATAACGCCCTCTTGATAAAACAAGAGGGAAGCTATTGCTCCttttttttATTTAGTACTA  181
              ||||||| |||||||||||||||||||||||||||||||||||||||| |||||||||||
Sbjct  413   ATAACGCTCTCTTGATAAAACAAGAGGGAAGCTATTGCTCCTTTTTTT-ATTTAGTACTA  355

Query  182   TTTGCCTTACATAGTTTCTTTAAAAATAACAAGTACTTTTTATAGTTTGGTTCGATTTGC  241
              ||| ||||||||||||||||||||||||||||| ||||||||||||||||||||||||||
Sbjct  354   TTTTCCTTACATAGTTTCTTTAAAAATAACAAGGACTTTTTATAGTTTGGTTCGATTTGC  295

Query  242   GTGtttttctttgtattcatttatattataggtttctatatcatatattttcCCAAT  300
              ||||||| | |||||||||||||||||||||||||||||||||||| | ||||||||||
Sbjct  294   GTGTTTTCTGTTTGTATTCATTTATATTATAGGTTTCTATATCATA-ACTTTTCCCAAT  237


>MZ324674.1 Hulsea algida voucher Baldwin 678 (DAV) photosystem II protein
D1 (psbA) gene, partial cds; psbA-trnH intergenic spacer,
complete sequence; and tRNA-His (trnH) gene, partial sequence;
plastid
Length=490

 Score = 492 bits (266),  Expect = 2e-134
 Identities = 281/288 (98%), Gaps = 1/288 (0%)
 Strand=Plus/Plus

Query  14    GTAATGCTCATAATTTCCCTCTAGACTTAGCTGCTATTGAAGCTCCATCTACAAATGGAT  73
              ||||||||| ||||||||||||||||||||||||||||||||||||||||||||||||||
Sbjct  1     GTAATGCTCGTAATTTCCCTCTAGACTTAGCTGCTATTGAAGCTCCATCTACAAATGGAT  60

Query  74    AAGCCTTTGGTCTGATTGTATAGGAGTTTTTGAACTAAAAAAGGAGCAATAACGCCCTCT  133
              ||| ||||||||||||||||||||||||||||||||||||||||||||||||||||||||
```

```
Sbjct  61   AAGACTTTGGTCTGATTGTATAGGAGTTTTTGAACTAAAAAAGGAGCAATAACGCCCTCT  120

Query  134  TGATAAAACAAGAGGGAAGCTATTGCTCCttttttttATTTAGTACTATTTGCCTTACAT  193
            ||||||||||||||||||||||||||||||||||||||||||||||||||||||||||||
Sbjct  121  TGATAAAACAAGAGGGAAGCTATTGCTCCTTTTTTTTATTTAGTACTATTTGCCTTACAT  180

Query  194  AGTTTCTTTAAAAATAACAAGTACTTTTTATAGTTTGGTTCGATTTGCGTGttttttctt  253
            ||||||||||||||||||||| ||| ||||||||||||||||||||||||||||||| ||
Sbjct  181  AGTTTCTTTAAAAATAACAAGGACTCTTTATAGTTTGGTTCGATTTGCGTGTTTTTTATT  240

Query  254  tgtattcatttatattataggtttctatatcatatatttttcCCAATC  301
            |||||||||||||||||||||||||||||||||||| |||||| ||||
Sbjct  241  TGTATTCATTTATATTATAGGTTTCTATATCATATACTTTTCC-AATC  287


>KF241798.1 Ambrosia artemisiifolia isolate AZ-1 PsbA (psbA) gene, partial
cds; and psbA-trnH intergenic spacer, partial sequence; chloroplast
Length=469

 Score = 490 bits (265),  Expect = 5e-134
 Identities = 285/294 (97%), Gaps = 4/294 (1%)
 Strand=Plus/Plus

Query  8    ATGAACGTAATGCTCATAATTTCCCTCTAGACTTAGCTGCTATTGAAGCTCCATCTACAA  67
            ||||||||||||||||||||||||||||||||||||||||||||||||||||||||||||
Sbjct  1    ATGAACGTAATGCTCATAATTTCCCTCTAGACTTAGCTGCTATTGAAGCTCCATCTACAA  60

Query  68   ATGGATAAGCCTTTGGTCTGATTGTATAGGAGTTTTTGAACTAAAAAAGGAGCAATAACG  127
            ||||||||| ||||||||||||||||||||||||||||||||||||||||||||||||||
Sbjct  61   ATGGATAAGACTTTGGTCTGATTGTATAGGAGTTTTTGAACTAAAAAAGGAGCAATAACG  120

Query  128  CCCTCTTGATAAAACAAGAGGGAAGCTATTGCTCCttttttttATTTAGTACTATTTGCC  187
            ||||||||||||||||||||||||||||||||||||||||||||||||||| ||||||||
Sbjct  121  CCCTCTTGATAAAACAAGAGGGAAGCTATTGCTCCTTTTTTTTATTTAGTATTATTTGCC  180

Query  188  TTACATAGTTTCTTTAAAAATAACAAGTACTTTTTATAGTTTGGTTCGATTTGCGTGttt  247
            |||||||||||||||||||||||||||||||||||||||||||||||||||||| |||||
Sbjct  181  TTACATAGTTTCTTTAAAAATAACAAGTACTTTTTATAGTTTGGTTCGATTTGCATGTTT  240

Query  248  tttctttgtattcatttatattataggtttctatatcatatatttttcCCAATC  301
            ||| ||||||||||||||||||||||||||||| || |||  |||| |||||||
Sbjct  241  TTTATTTGTATTCATTTATATTATAGGTTTCTA-AT-ATAC-TTTT-CCCAATC  290


>OR465018.1 Silphium perfoliatum clone 1072700_5 psbA-trnH intergenic spacer,
partial sequence; and photosystem II protein D1 (psbA)
gene, partial cds; plastid
Length=468

 Score = 490 bits (265),  Expect = 5e-134
 Identities = 284/293 (97%), Gaps = 2/293 (1%)
 Strand=Plus/Minus

Query  10   GAACGTAATGCTCATAATTTCCCTCTAGACTTAGCTGCTATTGAAGCTCCATCTACAAAT  69
            ||||||||||||||||||||||||||||||||||||||||||||||||||||||||||||
Sbjct  468  GAACGTAATGCTCATAATTTCCCTCTAGACTTAGCTGCTATTGAAGCTCCATCTACAAAT  409
```

```
Query  70   GGATAAGCCTTTGGTCTGATTGTATAGGAGTTTTTGAACTAAAAAAGGAGCAATAAC-GC  128
            ||||||| ||||||||||||||||||||||||||||||||||||||||||||||| |  |
Sbjct  408  GGATAAGACTTTGGTCTGATTGTATAGGAGTTTTTGAACTAAAAAAGGAGCAATAGCTTC  349

Query  129  CCTCTTGATAAAACAAGAGGGAAGCTATTGCTCCttttttttATTTAGTACTATTTGCCT  188
            |||||||||||||||||||||  | |||||||||||||||||||||||||||||||||||
Sbjct  348  CCTCTTGATAAAACAAGAGGG-CGTTATTGCTCCTTTTTTTTATTTAGTACTATTTGCCT  290

Query  189  TACATAGTTTCTTTAAAAATAACAAGTACTTTTTATAGTTTGGTTCGATTTGCGTGtttt  248
            ||||||||||||||||||||||||||||||||||||||||||||||||||||||||||||
Sbjct  289  TACATAGTTTCTTTAAAAATAACAAGTACTTTTTATAGTTTGGTTCGATTTGCGTGTTTT  230

Query  249  ttctttgtattcatttatattataggtttctatatcatatatttttcCCAATC  301
            ||||||||||||||||||||||||||||||||||||||||| |||||||| |||
Sbjct  229  TTCTTTGTATTCATTTATATTATAGGTTTCTATATCATATACTTTTCCCGATC  177
```

>MH464287.1 Balduina angustifolia voucher FLAS 234578 psbA-trnH intergenic spacer, partial sequence; chloroplast
Length=510

Score = 488 bits (264), Expect = 2e-133
Identities = 288/299 (96%), Gaps = 3/299 (1%)
Strand=Plus/Plus

```
Query  2    TTATGCATGAACGTAATGCTCATAATTTCCCTCTAGACTTAGCTGCTATTGAAGCTCCAT  61
            |||||||| |||||||||||||||||||||||||||||||||||||||||||||||||||
Sbjct  5    TTATGCAT-AACGTAATGCTCATAATTTCCCTCTAGACTTAGCTGCTATTGAAGCTCCAT  63

Query  62   CTACAAATGGATAAGCCTTTGGTCTGATTGTATAGGAGTTTTTGAACTAAAAAAGGAGCA  121
            ||||||||||||||| ||||| |||||||||||| |||||||||||||||||||||||||
Sbjct  64   CTACAAATGGATAAGACTTTGATCTGATTGTATAAGAGTTTTTGAACTAAAAAAGGAGCA  123

Query  122  ATAACGCCCTCTTGATAAAACAAGAGGGAAGCTATTGCTCCttttttttATTTAGTACTA  181
            ||||||||||||||||||||||||||||||||||||||||| ||||||||||||||||||
Sbjct  124  ATAACGCCCTCTTGATAAAACAAGAGGGAAGCTATTGCTCC-TTTTTTTATTTAGTACTA  182

Query  182  TTTGCCTTACATAGTTTCTTTAAAAATAACAAGTACTTTTTATAGTTTGGTTCGATTTGC  241
            ||| ||||||||||||||||||||||||||||| ||||||||||||||||||||||||||
Sbjct  183  TTTTCCTTACATAGTTTCTTTAAAAATAACAAGGACTTTTTATAGTTTGGTTCGATTTGC  242

Query  242  GTGttttttctttgtattcatttatattataggtttctatatcatatatttttcCCAAT  300
            ||||||| | |||||||||||||||||||||||||||||||||||| | ||||||||||
Sbjct  243  GTGTTTTCTGTTTGTATTCATTTATATTATAGGTTTCTATATCATA-ACTTTTCCCAAT  300
```

>NC_046787.1 Galinsoga parviflora voucher GPARV20171209 chloroplast, complete genome
MK737938.1 Galinsoga parviflora chloroplast, complete genome
Length=151811

Score = 488 bits (264), Expect = 2e-133
Identities = 289/301 (96%), Gaps = 2/301 (1%)
Strand=Plus/Minus

```
Query  2    TTATGCATGAACGTAATGCTCATAATTTCCCTCTAGACTTAGCTGCTATTGAAGCTCCAT  61
            ||||||||||||||||||||||||||||||||||||||||||||||||||||||||||||
```

```
Sbjct  523  TTATGCATGAACGTAATGCTCATAATTTCCCTCTAGACTTAGCTGCTATTGAAGCTCCAT  464

Query  62   CTACAAATGGATAAGCCTTTGGTCTGATTGTATAGGAGTTTTTGAACTAAAAAAGGAGCA  121
            ||||||||||||||| ||||||||||||||||||||||||||||||||||||||||||||
Sbjct  463  CTACAAATGGATAAGACTTTGGTCTGATTGTATAGGAGTTTTTGAACTAAAAAAGGAGCA  404

Query  122  ATAACGCCCTCTTGATAAAACAAGAGGGAAGCTATTGCTCCttttttttATTTAGTACTA  181
            ||||||||||||| |||||||||||||||||||||||||||||||||| |||||||||||
Sbjct  403  ATAACGCCCTCTTCATAAAACAAGAGGGAAGCTATTGCTCCTTTTTTT-ATTTAGTACTA  345

Query  182  TTTGCCTTACATAGTTTCTTTAAAAATAACAAGTACTTTTTATAGTTTGGTTCGATTTGC  241
            |||||||| |||||||||||||||||||||||| ||||||| ||||||||||| |||||
Sbjct  344  TTTGCCTTCCATAGTTTCTTTAAAAATAACAAGGACTTTTTCTAGTTTGGTTCTATTTGT  285

Query  242  GTGtttttt-ctttgtattcatttatattataggtttctatatcatatatttttcCCAAT  300
            || |||||| |||| |||||||||||||||||||||||||||||||||| ||||||||||
Sbjct  284  GTTTTTTTTTCTTTATATTCATTTATATTATAGGTTTCTATATCATATACTTTTCCCAAT  225

Query  301  C  301
            |
Sbjct  224  C  224


>MF348803.1 Engelmannia peristenia isolate AG2KK77 photosystem II protein
D1 (psbA) gene, partial cds; and psbA-trnH intergenic spacer,
partial sequence; chloroplast
Length=438

 Score = 486 bits (263),  Expect = 7e-133
 Identities = 274/279 (98%), Gaps = 1/279 (0%)
 Strand=Plus/Plus

Query  23   ATAATTTCCCTCTAGACTTAGCTGCTATTGAAGCTCCATCTACAAATGGATAAGCCTTTG  82
            |||||||||||||||||||||||||||||||||||||||||||||||||||||| |||||
Sbjct  1    ATAATTTCCCTCTAGACTTAGCTGCTATTGAAGCTCCATCTACAAATGGATAAGACTTTG  60

Query  83   GTCTGATTGTATAGGAGTTTTTGAACTAAAAAAGGAGCAATAACGCCCTCTTGATAAAAC  142
            ||||||||||||||||||||||||||||||||||||||||||||| ||||||||||||||
Sbjct  61   GTCTGATTGTATAGGAGTTTTTGAACTAAAAAAGGAGCAATAACGTCCTCTTGATAAAAC  120

Query  143  AAGAGGGAAGCTATTGCTCCttttttttATTTAGTACTATTTGCCTTACATAGTTTCTTT  202
            |||||||||||||||||||| |||||||||||||||||||||||||||||||||||||||
Sbjct  121  AAGAGGGAAGCTATTGCTCC-TTTTTTTATTTAGTACTATTTGCCTTACATAGTTTCTTT  179

Query  203  AAAAATAACAAGTACTTTTTATAGTTTGGTTCGATTTGCGTGtttttctttgtattcat  262
            |||||||||||||||||||||||||||||||| |||||||||||||||||||||||||||
Sbjct  180  AAAAATAACAAGTACTTTTTATAGTTTGGTTCAATTTGCGTGTTTTTTCTTTGTATTCAT  239

Query  263  ttatattataggtttctatatcatatatttttcCCAATC  301
            ||||||||||||||||||||||||||| |||||||||||
Sbjct  240  TTATATTATAGGTTTCTATATCATATACTTTTCCCAATC  278


>NC_081944.1 Venegasia carpesioides voucher US Weitzman et al. 412 chloroplast,
complete genome
 OR069732.1 Venegasia carpesioides voucher US Weitzman et al. 412 chloroplast,
complete genome
```

```
Length=152212

 Score = 483 bits (261),  Expect = 9e-132
 Identities = 288/301 (96%), Gaps = 2/301 (1%)
 Strand=Plus/Minus

Query  2    TTATGCATGAACGTAATGCTCATAATTTCCCTCTAGACTTAGCTGCTATTGAAGCTCCAT  61
            ||||||||||||||||||||||||||||||||||||||||||||||||||||||||||||
Sbjct  557  TTATGCATGAACGTAATGCTCATAATTTCCCTCTAGACTTAGCTGCTATTGAAGCTCCAT  498

Query  62   CTACAAATGGATAAGCCTTTGGTCTGATTGTATAGGAGTTTTTGAACTAAAAAAGGAGCA  121
            ||||||||||||||| ||||||||||||||||||||||||||||||||||||||||||||
Sbjct  497  CTACAAATGGATAAGACTTTGGTCTGATTGTATAGGAGTTTTTGAACTAAAAAAGGAGCA  438

Query  122  ATAACG-CCCTCTTGATAAAACAAGAGGGAAGCTATTGCTCCttttttttATTTAGTACT  180
            ||| |  |||||||| |  | ||||||||  |||||||||||||||||||||||||||||
Sbjct  437  ATAGCTTCCCTCTTGTTTTATCAAGAGGGC-GCTATTGCTCCTTTTTTTTATTTAGTACT  379

Query  181  ATTTGCCTTACATAGTTTCTTTAAAAATAACAAGTACTTTTTATAGTTTGGTTCGATTTG  240
            |||||||||||||||||||||||||||||||||| ||||||| |||||||||||||||||
Sbjct  378  ATTTGCCTTACATAGTTTCTTTAAAAATAACAAGGACTTTTTCTAGTTTGGTTCGATTTG  319

Query  241  CGTGtttttctttgtattcatttatattataggtttctatatcatatatttttcCCAAT  300
            |||||||||||||||||||||||||||||||||||||||||||||||| |||||||||||
Sbjct  318  CGTGTTTTTCTTTGTATTCATTTATATTATAGGTTTCTATATCATATACTTTTCCCAAT  259

Query  301  C  301
            |
Sbjct  258  C  258


 Score = 104 bits (56),  Expect = 8e-18
 Identities = 58/59 (98%), Gaps = 0/59 (0%)
 Strand=Plus/Plus

Query  110  AAAAAAGGAGCAATAACGCCCTCTTGATAAAACAAGAGGGAAGCTATTGCTCCtttttt  168
            ||||||||||||||| |||||||||||||||||||||||||||||||||||||||||||
Sbjct  391  AAAAAAGGAGCAATAGCGCCCTCTTGATAAAACAAGAGGGAAGCTATTGCTCCTTTTTT  449


>OZ286192.1 Venegasia carpesioides genome assembly, organelle: plastid
Length=152266

 Score = 483 bits (261),  Expect = 9e-132
 Identities = 288/301 (96%), Gaps = 2/301 (1%)
 Strand=Plus/Minus

Query  2    TTATGCATGAACGTAATGCTCATAATTTCCCTCTAGACTTAGCTGCTATTGAAGCTCCAT  61
            ||||||||||||||||||||||||||||||||||||||||||||||||||||||||||||
Sbjct  557  TTATGCATGAACGTAATGCTCATAATTTCCCTCTAGACTTAGCTGCTATTGAAGCTCCAT  498

Query  62   CTACAAATGGATAAGCCTTTGGTCTGATTGTATAGGAGTTTTTGAACTAAAAAAGGAGCA  121
            ||||||||||||||| ||||||||||||||||||||||||||||||||||||||||||||
Sbjct  497  CTACAAATGGATAAGACTTTGGTCTGATTGTATAGGAGTTTTTGAACTAAAAAAGGAGCA  438

Query  122  ATAACG-CCCTCTTGATAAAACAAGAGGGAAGCTATTGCTCCttttttttATTTAGTACT  180
            ||| |  |||||||| |  | ||||||||  |||||||||||||||||||||||||||||
```

```
Sbjct  437  ATAGCTTCCCTCTTGTTTTATCAAGAGGGC-GCTATTGCTCCTTTTTTTTATTTAGTACT  379

Query  181  ATTTGCCTTACATAGTTTCTTTAAAAATAACAAGTACTTTTTATAGTTTGGTTCGATTTG  240
            |||||||||||||||||||||||||||||||||| ||||||| |||||||||||||||||
Sbjct  378  ATTTGCCTTACATAGTTTCTTTAAAAATAACAAGGACTTTTTCTAGTTTGGTTCGATTTG  319

Query  241  CGTGtttttctttgtattcatttatattataggtttctatatcatatatttttcCCAAT  300
            |||||||||||||||||||||||||||||||||||||||||||||||| |||||||||||
Sbjct  318  CGTGTTTTTCTTTGTATTCATTTATATTATAGGTTTCTATATCATATACTTTTCCCAAT  259

Query  301  C  301
            |
Sbjct  258  C  258


 Score = 104 bits (56),  Expect = 8e-18
 Identities = 58/59 (98%), Gaps = 0/59 (0%)
 Strand=Plus/Plus

Query  110  AAAAAAGGAGCAATAACGCCCTCTTGATAAAACAAGAGGGAAGCTATTGCTCCtttttt  168
            ||||||||||||||| |||||||||||||||||||||||||||||||||||||||||||
Sbjct  391  AAAAAAGGAGCAATAGCGCCCTCTTGATAAAACAAGAGGGAAGCTATTGCTCCTTTTTT  449


>NC_031853.1 Galinsoga quadriradiata voucher GQUAD20160711 chloroplast, complete
genome
 KX752097.1 Galinsoga quadriradiata chloroplast, complete genome
Length=151917

 Score = 483 bits (261),  Expect = 9e-132
 Identities = 289/302 (96%), Gaps = 3/302 (1%)
 Strand=Plus/Minus

Query  2    TTATGCATGAACGTAATGCTCATAATTTCCCTCTAGACTTAGCTGCTATTGAAGCTCCAT  61
            ||||||||||||||||||||||||||||||||||||||||||||||||||||||||||||
Sbjct  524  TTATGCATGAACGTAATGCTCATAATTTCCCTCTAGACTTAGCTGCTATTGAAGCTCCAT  465

Query  62   CTACAAATGGATAAGCCTTTGGTCTGATTGTATAGGAGTTTTTGAACTAAAAAAGGAGCA  121
            ||||||||||||||| ||||||||||||||||||||||||||||||||||||||||||||
Sbjct  464  CTACAAATGGATAAGACTTTGGTCTGATTGTATAGGAGTTTTTGAACTAAAAAAGGAGCA  405

Query  122  ATAACGCCCTCTTGATAAAACAAGAGGGAAGCTATTGCTCCttttttttATTTAGTACTA  181
            ||||||||||||| |||||||||||||||||||||||||||||||||| |||||||||||
Sbjct  404  ATAACGCCCTCTTCATAAAACAAGAGGGAAGCTATTGCTCCTTTTTTT-ATTTAGTACTA  346

Query  182  TTTGCCTTACATAGTTTCTTTAAAAATAACAAGTACTTTTTATAGTTTGGTTCGATTTGC  241
            |||||||| |||||||||||||||||||||||| ||||||| ||||||||||| |||||
Sbjct  345  TTTGCCTTCCATAGTTTCTTTAAAAATAACAAGGACTTTTTCTAGTTTGGTTCTATTTGT  286

Query  242  GTGtttttt--ctttgtattcatttatattataggtttctatatcatatatttttcCCAA  299
            || ||||||  |||| |||||||||||||||||||||||||||||||||| |||||||||
Sbjct  285  GTTTTTTTTTTCTTTATATTCATTTATATTATAGGTTTCTATATCATATACTTTTCCCAA  226

Query  300  TC  301
            ||
Sbjct  225  TC  224
```

```
>NC_068130.1 Silphium integrifolium chloroplast, complete genome
 OM162161.1 Silphium integrifolium chloroplast, complete genome
Length=152058

 Score = 483 bits (261),  Expect = 9e-132
 Identities = 288/301 (96%), Gaps = 2/301 (1%)
 Strand=Plus/Minus

Query  2    TTATGCATGAACGTAATGCTCATAATTTCCCTCTAGACTTAGCTGCTATTGAAGCTCCAT  61
            ||||||||||||||||||||||||||||||||||||||||||||||||||||||||||||
Sbjct  559  TTATGCATGAACGTAATGCTCATAATTTCCCTCTAGACTTAGCTGCTATTGAAGCTCCAT  500

Query  62   CTACAAATGGATAAGCCTTTGGTCTGATTGTATAGGAGTTTTTGAACTAAAAAAGGAGCA  121
            ||||||||||||||| ||||||||||||||||||||||||||||||||||||||||||||
Sbjct  499  CTACAAATGGATAAGACTTTGGTCTGATTGTATAGGAGTTTTTGAACTAAAAAAGGAGCA  440

Query  122  ATAACG-CCCTCTTGATAAAACAAGAGGGAAGCTATTGCTCCttttttttATTTAGTACT  180
            ||| |  |||||||| |  | ||||||||  | |||||||||||||||||||||||||||
Sbjct  439  ATAGCTTCCCTCTTGTTTTATCAAGAGGGC-GTTATTGCTCCTTTTTTTTATTTAGTACT  381

Query  181  ATTTGCCTTACATAGTTTCTTTAAAAATAACAAGTACTTTTTATAGTTTGGTTCGATTTG  240
            ||||||||||||||||||||||||||||||||||||||||||||||||||||||||||||
Sbjct  380  ATTTGCCTTACATAGTTTCTTTAAAAATAACAAGTACTTTTTATAGTTTGGTTCGATTTG  321

Query  241  CGTGtttttctttgtattcatttatattataggtttctatatcatatatttttcCCAAT  300
            ||||||||||||||||||||||||||||||||||||||||||||||||| ||||||| ||
Sbjct  320  CGTGTTTTTCTTTGTATTCATTTATATTATAGGTTTCTATATCATATACTTTTCCCGAT  261

Query  301  C  301
            |
Sbjct  260  C  260


 Score = 110 bits (59),  Expect = 2e-19
 Identities = 59/59 (100%), Gaps = 0/59 (0%)
 Strand=Plus/Plus

Query  110  AAAAAAGGAGCAATAACGCCCTCTTGATAAAACAAGAGGGAAGCTATTGCTCCtttttt  168
            |||||||||||||||||||||||||||||||||||||||||||||||||||||||||||
Sbjct  393  AAAAAAGGAGCAATAACGCCCTCTTGATAAAACAAGAGGGAAGCTATTGCTCCTTTTTT  451


>NC_060408.1 Silphium perfoliatum chloroplast, complete sequence
 MN445187.1 Silphium perfoliatum chloroplast, complete genome
Length=151924

 Score = 483 bits (261),  Expect = 9e-132
 Identities = 288/301 (96%), Gaps = 2/301 (1%)
 Strand=Plus/Minus

Query  2    TTATGCATGAACGTAATGCTCATAATTTCCCTCTAGACTTAGCTGCTATTGAAGCTCCAT  61
            ||||||||||||||||||||||||||||||||||||||||||||||||||||||||||||
Sbjct  562  TTATGCATGAACGTAATGCTCATAATTTCCCTCTAGACTTAGCTGCTATTGAAGCTCCAT  503

Query  62   CTACAAATGGATAAGCCTTTGGTCTGATTGTATAGGAGTTTTTGAACTAAAAAAGGAGCA  121
            ||||||||||||||| ||||||||||||||||||||||||||||||||||||||||||||
```

```
Sbjct  502  CTACAAATGGATAAGACTTTGGTCTGATTGTATAGGAGTTTTTGAACTAAAAAAGGAGCA  443

Query  122  ATAACG-CCCTCTTGATAAAACAAGAGGGAAGCTATTGCTCCtttttttATTTAGTACT  180
            ||| |  |||||||| |  | ||||||||  | |||||||||||||||||||||||||||
Sbjct  442  ATAGCTTCCCTCTTGTTTTATCAAGAGGGC-GTTATTGCTCCTTTTTTTATTTAGTACT  384

Query  181  ATTTGCCTTACATAGTTTCTTTAAAAATAACAAGTACTTTTTATAGTTTGGTTCGATTTG  240
            ||||||||||||||||||||||||||||||||||||||||||||||||||||||||||||
Sbjct  383  ATTTGCCTTACATAGTTTCTTTAAAAATAACAAGTACTTTTTATAGTTTGGTTCGATTTG  324

Query  241  CGTGttttttctttgtattcatttatattataggtttctatatcatatatttttcCCAAT  300
            ||||||||||||||||||||||||||||||||||||||||||||||||| ||||||| ||
Sbjct  323  CGTGTTTTTTCTTTGTATTCATTTATATTATAGGTTTCTATATCATATACTTTTCCCGAT  264

Query  301  C  301
            |
Sbjct  263  C  263


 Score = 110 bits (59),  Expect = 2e-19
 Identities = 59/59 (100%), Gaps = 0/59 (0%)
 Strand=Plus/Plus

Query  110  AAAAAAGGAGCAATAACGCCCTCTTGATAAAACAAGAGGGAAGCTATTGCTCCtttttt  168
            |||||||||||||||||||||||||||||||||||||||||||||||||||||||||||
Sbjct  396  AAAAAAGGAGCAATAACGCCCTCTTGATAAAACAAGAGGGAAGCTATTGCTCCTTTTTT  454


>OK469451.1 Jefea brevifolia voucher BRIT:Gostel611 photosystem II protein
D1 (psbA) gene, partial cds; psbA-trnH intergenic spacer,
complete sequence; and tRNA-His (trnH) gene, partial sequence;
chloroplast
Length=510

 Score = 481 bits (260),  Expect = 3e-131
 Identities = 273/279 (98%), Gaps = 2/279 (1%)
 Strand=Plus/Plus

Query  23   ATAATTTCCCTCTAGACTTAGCTGCTATTGAAGCTCCATCTACAAATGGATAAGCCTTTG  82
            |||||||||||||||||||||||||||||||||||||||||||||||||||||| |||||
Sbjct  1    ATAATTTCCCTCTAGACTTAGCTGCTATTGAAGCTCCATCTACAAATGGATAAGACTTTG  60

Query  83   GTCTGATTGTATAGGAGTTTTTGAACTAAAAAAGGAGCAATAACGCCCTCTTGATAAAAC  142
            ||||||||||||||||||||||||||||||||||||||||||||||||||||||||||||
Sbjct  61   GTCTGATTGTATAGGAGTTTTTGAACTAAAAAAGGAGCAATAACGCCCTCTTGATAAAAC  120

Query  143  AAGAGGGAAGCTATTGCTCC-ttttttttATTTAGTACTATTTGCCTTACATAGTTTCTT  201
            |||||||||||||||||||| |||||||||||||||||||||||||||||||||||||||
Sbjct  121  AAGAGGGAAGCTATTGCTCCTTTTTTTTTATTTAGTACTATTTGCCTTACATAGTTTCTT  180

Query  202  TAAAAATAACAAGTACTTTTTATAGTTTGGTTCGATTTGCGTGttttttctttgtattca  261
            ||||||||||||| ||||||||||||||||||||||||||||||||||||||||||||||
Sbjct  181  TAAAAATAACAAGGACTTTTTATAGTTTGGTTCGATTTGCGTGTTTTTTCTTTGTATTCA  240

Query  262  tttatattataggtttctatatcatatatttttcCCAAT  300
            |||| |||||||||||||||||| |||| ||||||||||
Sbjct  241  TTTAGATTATAGGTTTCTATATC-TATACTTTTCCCAAT  278
```

>MG019037.1 Ambrosia artemisiifolia chloroplast, complete genome
Length=152223

Score = 481 bits (260), Expect = 3e-131
Identities = 289/301 (96%), Gaps = 10/301 (3%)
Strand=Plus/Minus

```
Query  2    TTATGCATGAACGTAATGCTCATAATTTCCCTCTAGACTTAGCTGCTATTGAAGCTCCAT  61
            ||||||||||||||||||||||||||||||||||||||||||||||||||||||||||||
Sbjct  534  TTATGCATGAACGTAATGCTCATAATTTCCCTCTAGACTTAGCTGCTATTGAAGCTCCAT  475

Query  62   CTACAAATGGATAAGCCTTTGGTCTGATTGTATAGGAGTTTTTGAACTAAAAAAGGAGCA  121
            ||||||||||||||| ||||||||||||||||||||||||||||||||||||||||||||
Sbjct  474  CTACAAATGGATAAGACTTTGGTCTGATTGTATAGGAGTTTTTGAACTAAAAAAGGAGCA  415

Query  122  ATAACG-CCCTCTTGATAAAACAAGAGGGAAGCTATTGCTCCttttttttATTTAGTACT  180
            |||||| |||||||||||||||||||||||||||||||||||||||||||||||||||||
Sbjct  414  ATAACGTCCCTCTTGATAAAACAAGAGGGAAGCTATTGCTCCTTTTTTTTATTTAGTACT  355

Query  181  ATTTGCCTTACATAGTTTCTTTAAAAATAACAAGTACTTTTATAGTTTGGTTCGATTTG  240
            |||||||||||||||||||||||||||||         ||||||||||||||||||||||
Sbjct  354  ATTTGCCTTACATAGTTTCTTTAAAAATA---------TTTTATAGTTTGGTTCGATTTG  304

Query  241  CGTGtttttctttgtattcatttatattataggtttctatatcatatatttttcCCAAT  300
            ||||||||||||||||||||||||||||||||||||||||||||||||| ||||||||||
Sbjct  303  CGTGTTTTTCTTTGTATTCATTTATATTATAGGTTTCTATATCATATACTTTTCCCAAT  244

Query  301  C  301
            |
Sbjct  243  C  243
```

>AB427162.1 Ambrosia artemisiifolia chloroplast psbA gene for D1 protein, complete cds
Length=1693

Score = 479 bits (259), Expect = 1e-130
Identities = 288/300 (96%), Gaps = 9/300 (3%)
Strand=Plus/Plus

```
Query  2     TTATGCATGAACGTAATGCTCATAATTTCCCTCTAGACTTAGCTGCTATTGAAGCTCCAT  61
             ||||||||||||||||||||||||||||||||||||||||||||||||||||||||||||
Sbjct  1207  TTATGCATGAACGTAATGCTCATAATTTCCCTCTAGACTTAGCTGCTATTGAAGCTCCAT  1266

Query  62    CTACAAATGGATAAGCCTTTGGTCTGATTGTATAGGAGTTTTTGAACTAAAAAAGGAGCA  121
             ||||||||||||||| ||||||||||||||||||||||||||||||||||||||||||||
Sbjct  1267  CTACAAATGGATAAGACTTTGGTCTGATTGTATAGGAGTTTTTGAACTAAAAAAGGAGCA  1326

Query  122   ATAACGCCCTCTTGATAAAACAAGAGGGAAGCTATTGCTCCttttttttATTTAGTACTA  181
             |||||| |||||||||||||||||||||||||||||||||||||||||||||||||||||
Sbjct  1327  ATAACGTCCTCTTGATAAAACAAGAGGGAAGCTATTGCTCCTTTTTTTTATTTAGTACTA  1386

Query  182   TTTGCCTTACATAGTTTCTTTAAAAATAACAAGTACTTTTATAGTTTGGTTCGATTTGC  241
             ||||||||||||||||||||||||||||         |||||||||||||||||||||||
Sbjct  1387  TTTGCCTTACATAGTTTCTTTAAAAATA---------TTTTATAGTTTGGTTCGATTTGC  1437
```

```
Query  242   GTGtttttctttgtattcatttatattataggtttctatatcatatatttttcCCAATC  301
             |||||||||||||||||||||||||||||||||||||||||||||||| |||||||||||
Sbjct  1438  GTGTTTTTCTTTGTATTCATTTATATTATAGGTTTCTATATCATATACTTTTCCCAATC  1497


>NC_035875.1 Ambrosia artemisiifolia chloroplast, complete genome
 MF362689.1 Ambrosia artemisiifolia chloroplast, complete genome
Length=152215

 Score = 479 bits (259),  Expect = 1e-130
 Identities = 288/300 (96%), Gaps = 9/300 (3%)
 Strand=Plus/Minus

Query  2    TTATGCATGAACGTAATGCTCATAATTTCCCTCTAGACTTAGCTGCTATTGAAGCTCCAT  61
            ||||||||||||||||||||||||||||||||||||||||||||||||||||||||||||
Sbjct  706  TTATGCATGAACGTAATGCTCATAATTTCCCTCTAGACTTAGCTGCTATTGAAGCTCCAT  647

Query  62   CTACAAATGGATAAGCCTTTGGTCTGATTGTATAGGAGTTTTTGAACTAAAAAAGGAGCA  121
            ||||||||||||||| ||||||||||||||||||||||||||||||||||||||||||||
Sbjct  646  CTACAAATGGATAAGACTTTGGTCTGATTGTATAGGAGTTTTTGAACTAAAAAAGGAGCA  587

Query  122  ATAACGCCCTCTTGATAAAACAAGAGGGAAGCTATTGCTCCttttttttATTTAGTACTA  181
            |||||| |||||||||||||||||||||||||||||||||||||||||||||||||||||
Sbjct  586  ATAACGTCCTCTTGATAAAACAAGAGGGAAGCTATTGCTCCTTTTTTTTATTTAGTACTA  527

Query  182  TTTGCCTTACATAGTTTCTTTAAAAATAACAAGTACTTTTATAGTTTGGTTCGATTTGC  241
            ||||||||||||||||||||||||||||         |||||||||||||||||||||||
Sbjct  526  TTTGCCTTACATAGTTTCTTTAAAAATA---------TTTTATAGTTTGGTTCGATTTGC  476

Query  242  GTGtttttctttgtattcatttatattataggtttctatatcatatatttttcCCAATC  301
            |||||||||||||||||||||||||||||||||||||||||||||||| |||||||||||
Sbjct  475  GTGTTTTTCTTTGTATTCATTTATATTATAGGTTTCTATATCATATACTTTTCCCAATC  416


>EF590669.1 Ambrosia artemisiifolia voucher NMNH Wells 4495 PsbA (psbA) gene
and psbA-trnH intergenic spacer, partial sequence; chloroplast
 EF590670.1 Ambrosia trifida voucher NMNH Wells 30 PsbA (psbA) gene and psbA-trnH
intergenic spacer, partial sequence; chloroplast
Length=460

 Score = 479 bits (259),  Expect = 1e-130
 Identities = 288/300 (96%), Gaps = 9/300 (3%)
 Strand=Plus/Plus

Query  2    TTATGCATGAACGTAATGCTCATAATTTCCCTCTAGACTTAGCTGCTATTGAAGCTCCAT  61
            ||||||||||||||||||||||||||||||||||||||||||||||||||||||||||||
Sbjct  61   TTATGCATGAACGTAATGCTCATAATTTCCCTCTAGACTTAGCTGCTATTGAAGCTCCAT  120

Query  62   CTACAAATGGATAAGCCTTTGGTCTGATTGTATAGGAGTTTTTGAACTAAAAAAGGAGCA  121
            ||||||||||||||| ||||||||||||||||||||||||||||||||||||||||||||
Sbjct  121  CTACAAATGGATAAGACTTTGGTCTGATTGTATAGGAGTTTTTGAACTAAAAAAGGAGCA  180

Query  122  ATAACGCCCTCTTGATAAAACAAGAGGGAAGCTATTGCTCCttttttttATTTAGTACTA  181
            |||||| |||||||||||||||||||||||||||||||||||||||||||||||||||||
Sbjct  181  ATAACGTCCTCTTGATAAAACAAGAGGGAAGCTATTGCTCCTTTTTTTTATTTAGTACTA  240
```

```
Query  182  TTTGCCTTACATAGTTTCTTTAAAAATAACAAGTACTTTTTATAGTTTGGTTCGATTTGC  241
            ||||||||||||||||||||||||||||         |||||||||||||||||||||||
Sbjct  241  TTTGCCTTACATAGTTTCTTTAAAAATA---------TTTTATAGTTTGGTTCGATTTGC  291

Query  242  GTGtttttctttgtattcatttatattataggtttctatatcatatatttttcCCAATC  301
            |||||||||||||||||||||||||||||||||||||||||||||||| |||||||||||
Sbjct  292  GTGTTTTTCTTTGTATTCATTTATATTATAGGTTTCTATATCATATACTTTTCCCAATC  351
```

>NC_036810.2 Ambrosia trifida chloroplast, complete genome
 MG029118.2 Ambrosia trifida chloroplast, complete genome
Length=152040

 Score = 479 bits (259), Expect = 1e-130
 Identities = 288/300 (96%), Gaps = 9/300 (3%)
 Strand=Plus/Minus

```
Query  2    TTATGCATGAACGTAATGCTCATAATTTCCCTCTAGACTTAGCTGCTATTGAAGCTCCAT  61
            ||||||||||||||||||||||||||||||||||||||||||||||||||||||||||||
Sbjct  533  TTATGCATGAACGTAATGCTCATAATTTCCCTCTAGACTTAGCTGCTATTGAAGCTCCAT  474

Query  62   CTACAAATGGATAAGCCTTTGGTCTGATTGTATAGGAGTTTTTGAACTAAAAAAGGAGCA  121
            ||||||||||||||| ||||||||||||||||||||||||||||||||||||||||||||
Sbjct  473  CTACAAATGGATAAGACTTTGGTCTGATTGTATAGGAGTTTTTGAACTAAAAAAGGAGCA  414

Query  122  ATAACGCCCTCTTGATAAAACAAGAGGGAAGCTATTGCTCCtttttttATTTAGTACTA  181
            |||||| |||||||||||||||||||||||||||||||||||||||||||||||||||||
Sbjct  413  ATAACGTCCTCTTGATAAAACAAGAGGGAAGCTATTGCTCCTTTTTTTATTTAGTACTA  354

Query  182  TTTGCCTTACATAGTTTCTTTAAAAATAACAAGTACTTTTTATAGTTTGGTTCGATTTGC  241
            ||||||||||||||||||||||||||||         |||||||||||||||||||||||
Sbjct  353  TTTGCCTTACATAGTTTCTTTAAAAATA---------TTTTATAGTTTGGTTCGATTTGC  303

Query  242  GTGtttttctttgtattcatttatattataggtttctatatcatatatttttcCCAATC  301
            |||||||||||||||||||||||||||||||||||||||||||||||| |||||||||||
Sbjct  302  GTGTTTTTCTTTGTATTCATTTATATTATAGGTTTCTATATCATATACTTTTCCCAATC  243
```

>MF037493.1 Echinacea paradoxa voucher Rebman1085 trnH-psbA intergenic spacer region, partial sequence; chloroplast
Length=429

 Score = 477 bits (258), Expect = 4e-130
 Identities = 258/258 (100%), Gaps = 0/258 (0%)
 Strand=Plus/Plus

```
Query  44   CTGCTATTGAAGCTCCATCTACAAATGGATAAGCCTTTGGTCTGATTGTATAGGAGTTTT  103
            ||||||||||||||||||||||||||||||||||||||||||||||||||||||||||||
Sbjct  1    CTGCTATTGAAGCTCCATCTACAAATGGATAAGCCTTTGGTCTGATTGTATAGGAGTTTT  60

Query  104  TGAACTAAAAAAGGAGCAATAACGCCCTCTTGATAAAACAAGAGGGAAGCTATTGCTCCt  163
            ||||||||||||||||||||||||||||||||||||||||||||||||||||||||||||
Sbjct  61   TGAACTAAAAAAGGAGCAATAACGCCCTCTTGATAAAACAAGAGGGAAGCTATTGCTCCT  120

Query  164  tttttttATTTAGTACTATTTGCCTTACATAGTTTCTTTAAAAATAACAAGTACTTTTTA  223
            ||||||||||||||||||||||||||||||||||||||||||||||||||||||||||||
Sbjct  121  TTTTTTTATTTAGTACTATTTGCCTTACATAGTTTCTTTAAAAATAACAAGTACTTTTTA  180
```

```
Query  224  TAGTTTGGTTCGATTTGCGTGttttttctttgtattcatttatattataggtttctatat  283
            ||||||||||||||||||||||||||||||||||||||||||||||||||||||||||||
Sbjct  181  TAGTTTGGTTCGATTTGCGTGTTTTTTCTTTGTATTCATTTATATTATAGGTTTCTATAT  240

Query  284  catatatttttcCCAATC  301
            ||||||||||||||||||
Sbjct  241  CATATATTTTTCCCAATC  258
```

>AY215540.1 Engelmannia peristenia voucher Clevinger 376 (TEX) psbA gene and psbA-trnH intergenic spacer, partial sequence; chloroplast
Length=403

Score = 477 bits (258), Expect = 4e-130
Identities = 269/274 (98%), Gaps = 1/274 (0%)
Strand=Plus/Minus

```
Query  28   TTCCCTCTAGACTTAGCTGCTATTGAAGCTCCATCTACAAATGGATAAGCCTTTGGTCTG  87
            ||||||||||||||||||||||||||||||||||||||||||||||||| ||||||||||
Sbjct  403  TTCCCTCTAGACTTAGCTGCTATTGAAGCTCCATCTACAAATGGATAAGACTTTGGTCTG  344

Query  88   ATTGTATAGGAGTTTTTGAACTAAAAAAGGAGCAATAACGCCCTCTTGATAAAACAAGAG  147
            |||||||||||||||||||||||||||||||||||||||| |||||||||||||||||||
Sbjct  343  ATTGTATAGGAGTTTTTGAACTAAAAAAGGAGCAATAACGTCCTCTTGATAAAACAAGAG  284

Query  148  GGAAGCTATTGCTCCtttttttttATTTAGTACTATTTGCCTTACATAGTTTCTTAAAAA  207
            ||||||||||||||||||||||| ||||||||||||||||||||||||||||||||||||
Sbjct  283  GGAAGCTATTGCTCCTTTTTTTT-ATTTAGTACTATTTGCCTTACATAGTTTCTTAAAAA  225

Query  208  TAACAAGTACTTTTTATAGTTTGGTTCGATTTGCGTGttttttctttgtattcatttata  267
            ||||||||||||||||||||||||||| ||||||||||||||||||||||||||||||||
Sbjct  224  TAACAAGTACTTTTTATAGTTTGGTTCAATTTGCGTGTTTTTTCTTTGTATTCATTTATA  165

Query  268  ttataggtttctatatcatatatttttcCCAATC  301
            |||||||||||||||||||||| |||||||||||
Sbjct  164  TTATAGGTTTCTATATCATATACTTTTCCCAATC  131
```

>NC_054222.1 Xanthium spinosum chloroplast, complete genome
MT668935.1 Xanthium spinosum chloroplast, complete genome
Length=152422

Score = 475 bits (257), Expect = 2e-129
Identities = 266/270 (99%), Gaps = 1/270 (0%)
Strand=Plus/Minus

```
Query  2    TTATGCATGAACGTAATGCTCATAATTTCCCTCTAGACTTAGCTGCTATTGAAGCTCCAT  61
            ||||||||||||||||||||||||||||||||||||||||||||||||||||||||||||
Sbjct  675  TTATGCATGAACGTAATGCTCATAATTTCCCTCTAGACTTAGCTGCTATTGAAGCTCCAT  616

Query  62   CTACAAATGGATAAGCCTTTGGTCTGATTGTATAGGAGTTTTTGAACTAAAAAAGGAGCA  121
            ||||||||||||||| ||||||||||||||||||||||||||||||||||||||||||||
Sbjct  615  CTACAAATGGATAAGACTTTGGTCTGATTGTATAGGAGTTTTTGAACTAAAAAAGGAGCA  556

Query  122  ATAACGCCCTCTTGATAAAACAAGAGGGAAGCTATTGCTCCttttttttATTTAGTACTA  181
            |||||||||||||||||||||||||||||||||||||||||||||||| |||||||||||
```

```
Sbjct  555  ATAACGCCCTCTTGATAAAACAAGAGGGAAGCTATTGCTCCTTTTTTT-ATTTAGTACTA  497

Query  182  TTTGCCTTACATAGTTTCTTTAAAAATAACAAGTACTTTTTATAGTTTGGTTCGATTTGC  241
            |||||||||||||||||||||||||| |||||||||||||||||||||||| ||||||||
Sbjct  496  TTTGCCTTACATAGTTTCTTTAAAAAAAACAAGTACTTTTTATAGTTTGGTTTGATTTGC  437

Query  242  GTGttttttctttgtattcatttatattat  271
            ||||||||||||||||||||||||||||||
Sbjct  436  GTGTTTTTTCTTTGTATTCATTTATATTAT  407
```

>MT793838.1 Mikania lehmannii chloroplast, complete genome
Length=152062

```
 Score = 475 bits (257),  Expect = 2e-129
 Identities = 295/311 (95%), Gaps = 12/311 (4%)
 Strand=Plus/Minus

Query  2    TTATGCATGAACGTAATGCTCATAATTTCCCTCTAGACTTAGCTGCTATTGAAGCTCCAT  61
            ||||||||||||||||||||||||||||||||||||||||||||||||||||||||||||
Sbjct  535  TTATGCATGAACGTAATGCTCATAATTTCCCTCTAGACTTAGCTGCTATTGAAGCTCCAT  476

Query  62   CTACAAATGGATAAGCCTTTGGTCTGATTGTATAGGAGTTTTTGAACTAAAAAAGGAGCA  121
            ||||||||||||||| ||||||||||||||||||||||||||||||||||||||||||||
Sbjct  475  CTACAAATGGATAAGACTTTGGTCTGATTGTATAGGAGTTTTTGAACTAAAAAAGGAGCA  416

Query  122  ATAACGCCCTCTTGATAAAACAAGAGGGAAGCTATTGCTCCttttttttATTTAGTACTA  181
            ||||||||||||||||| |||||||||||||||||||||||||||||| |||||||||||
Sbjct  415  ATAACGCCCTCTTGATACAACAAGAGGGAAGCTATTGCTCCTTTTTTT-ATTTAGTACTA  357

Query  182  TTTGCCTTACATAGTTTCTTTAAAAATAAC--AAG--T-------ACTTTTTATAGTTTG  230
            ||||||||||||||||||||||||||||||  ||   |       |||||||||||||||
Sbjct  356  TTTGCCTTACATAGTTTCTTTAAAAATAACTTAAAAATAACAAGGACTTTTTATAGTTTG  297

Query  231  GTTCGATTTGCGTGttttttctttgtattcatttatattataggtttctatatcatatat  290
            ||||||||||||||||||||||||||||||||||||||||||||||||||||| ||||||
Sbjct  296  GTTCGATTTGCGTGTTTTTTCTTTGTATTCATTTATATTATAGGTTTCTATATAATATAT  237

Query  291  ttttcCCAATC  301
            |||||||||||
Sbjct  236  TTTTCCCAATC  226
```

>MT793855.1 Mikania sylvatica chloroplast, complete genome
Length=152099

```
 Score = 475 bits (257),  Expect = 2e-129
 Identities = 295/311 (95%), Gaps = 12/311 (4%)
 Strand=Plus/Minus

Query  2    TTATGCATGAACGTAATGCTCATAATTTCCCTCTAGACTTAGCTGCTATTGAAGCTCCAT  61
            ||||||||||||||||||||||||||||||||||||||||||||||||||||||||||||
Sbjct  534  TTATGCATGAACGTAATGCTCATAATTTCCCTCTAGACTTAGCTGCTATTGAAGCTCCAT  475

Query  62   CTACAAATGGATAAGCCTTTGGTCTGATTGTATAGGAGTTTTTGAACTAAAAAAGGAGCA  121
            ||||||||||||||| ||||||||||||||||||||||||||||||||||||||||||||
Sbjct  474  CTACAAATGGATAAGACTTTGGTCTGATTGTATAGGAGTTTTTGAACTAAAAAAGGAGCA  415
```

```
Query  122  ATAACGCCCTCTTGATAAAACAAGAGGGAAGCTATTGCTCCttttttttATTTAGTACTA  181
            ||||||||||||||||| |||||||||||||||||||||||||||||| |||||||||||
Sbjct  414  ATAACGCCCTCTTGATACAACAAGAGGGAAGCTATTGCTCCTTTTTTT-ATTTAGTACTA  356

Query  182  TTTGCCTTACATAGTTTCTTTAAAAATAAC-----------AAGTACTTTTTATAGTTTG  230
            ||||||||||||||||||||||||||||||           ||| |||||||||||||||
Sbjct  355  TTTGCCTTACATAGTTTCTTTAAAAATAACTTAAAAAAAACAAGGACTTTTTATAGTTTG  296

Query  231  GTTCGATTTGCGTGttttttctttgtattcatttatattataggtttctatatcatatat  290
            ||||||||||||||||||||||||||||||||||||||||||||||||||||| ||||||
Sbjct  295  GTTCGATTTGCGTGTTTTTTCTTTGTATTCATTTATATTATAGGTTTCTATATAATATAT  236

Query  291  ttttcCCAATC  301
            |||||||||||
Sbjct  235  TTTTCCCAATC  225
```

>AY215509.1 Alepidocline annua voucher Panero 3109 (TEX) psbA gene and psbA-trnH intergenic spacer, partial sequence; chloroplast
AY215583.1 Oteiza scandens voucher Panero 2745 (TEX) psbA gene and psbA-trnH intergenic spacer, partial sequence; chloroplast
Length=433

Score = 473 bits (256), Expect = 6e-129
Identities = 268/274 (98%), Gaps = 0/274 (0%)
Strand=Plus/Minus

```
Query  28   TTCCCTCTAGACTTAGCTGCTATTGAAGCTCCATCTACAAATGGATAAGCCTTTGGTCTG  87
            ||||||||||||||||||||||||||||||||||||||||||||||||| ||||||||||
Sbjct  433  TTCCCTCTAGACTTAGCTGCTATTGAAGCTCCATCTACAAATGGATAAGACTTTGGTCTG  374

Query  88   ATTGTATAGGAGTTTTTGAACTAAAAAAGGAGCAATAACGCCCTCTTGATAAAACAAGAG  147
            ||||||||||||||||||||||||||||||||||||||||||||||| ||||||||||||
Sbjct  373  ATTGTATAGGAGTTTTTGAACTAAAAAAGGAGCAATAACGCCCTCTTAATAAAACAAGAG  314

Query  148  GGAAGCTATTGCTCCttttttttATTTAGTACTATTTGCCTTACATAGTTTCTTTAAAAA  207
            |||||||||||||||||||||||||||||||||||||||||| |||||||||||||||||
Sbjct  313  GGAAGCTATTGCTCCTTTTTTTTATTTAGTACTATTTGCCTTCCATAGTTTCTTTAAAAA  254

Query  208  TAACAAGTACTTTTTATAGTTTGGTTCGATTTGCGTGttttttctttgtattcatttata  267
            ||||||| ||||||||||||||||||||||||| ||||||||||||||||||||||||||
Sbjct  253  TAACAAGGACTTTTTATAGTTTGGTTCGATTTGTGTGTTTTTTCTTTGTATTCATTTATA  194

Query  268  ttataggtttctatatcatatatttttcCCAATC  301
            |||||||||||||||||||||| |||||||||||
Sbjct  193  TTATAGGTTTCTATATCATATACTTTTCCCAATC  160
```

>OZ286201.1 Guardiola tulocarpus genome assembly, organelle: plastid
Length=151368

Score = 472 bits (255), Expect = 2e-128
Identities = 264/268 (99%), Gaps = 2/268 (1%)
Strand=Plus/Minus

```
Query  2    TTATGCATGAACGTAATGCTCATAATTTCCCTCTAGACTTAGCTGCTATTGAAGCTCCAT  61
```

```
            ||||||||||||||||||||||||||||||||||||||||||||||||||||||||||||
Sbjct  595  TTATGCATGAACGTAATGCTCATAATTTCCCTCTAGACTTAGCTGCTATTGAAGCTCCAT  536

Query  62   CTACAAATGGATAAGCCTTTGGTCTGATTGTATAGGAGTTTTTGAACTAAAAAAGGAGCA  121
            ||||||||||||||| ||||||||||||||||||||||||||||||||||||||||||||
Sbjct  535  CTACAAATGGATAAGACTTTGGTCTGATTGTATAGGAGTTTTTGAACTAAAAAAGGAGCA  476

Query  122  ATAACGCCCTCTTGATAAAACAAGAGGGAAGCTATTGCTCCtttttttATTTAGTACTA  181
            ||||||||||||||||||||||||||  ||||||||||||||||||||||||||||||||
Sbjct  475  ATAACGCCCTCTTGATAAAACAAGAG--AAGCTATTGCTCCTTTTTTTATTTAGTACTA  418

Query  182  TTTGCCTTACATAGTTTCTTTAAAAATAACAAGTACTTTTTATAGTTTGGTTCGATTTGC  241
            ||||||||||||||||||||||||||||||||| ||||||||||||||||||||||||||
Sbjct  417  TTTGCCTTACATAGTTTCTTTAAAAATAACAAGGACTTTTTATAGTTTGGTTCGATTTGC  358

Query  242  GTGtttttctttgtattcatttatatt  269
            ||||||||||||||||||||||||||||
Sbjct  357  GTGTTTTTCTTTGTATTCATTTATATT  330
```

>AY215627.1 Zaluzania megacephala voucher Panero 2249 (TEX) psbA gene and psbA-trnH intergenic spacer, partial sequence; chloroplast
Length=438

Score = 472 bits (255), Expect = 2e-128
Identities = 268/274 (98%), Gaps = 1/274 (0%)
Strand=Plus/Minus

```
Query  28   TTCCCTCTAGACTTAGCTGCTATTGAAGCTCCATCTACAAATGGATAAGCCTTTGGTCTG  87
            ||||||||||||||||||||||||||||||||||||||||||||||||| ||||| ||||
Sbjct  438  TTCCCTCTAGACTTAGCTGCTATTGAAGCTCCATCTACAAATGGATAAGACTTTGTTCTG  379

Query  88   ATTGTATAGGAGTTTTTGAACTAAAAAAGGAGCAATAACGCCCTCTTGATAAAACAAGAG  147
            ||||||||||||||||||||||||||||||||||||||||||||||||||||||||||||
Sbjct  378  ATTGTATAGGAGTTTTTGAACTAAAAAAGGAGCAATAACGCCCTCTTGATAAAACAAGAG  319

Query  148  GGAAGCTATTGCTCCttttttttATTTAGTACTATTTGCCTTACATAGTTTCTTTAAAAA  207
            |||||||||||||||||||||| |||||||| ||||||||||||||||||||||||||||
Sbjct  318  GGAAGCTATTGCTCCTTTTTTT-ATTTAGTAATATTTGCCTTACATAGTTTCTTTAAAAA  260

Query  208  TAACAAGTACTTTTTATAGTTTGGTTCGATTTGCGTGtttttctttgtattcatttata  267
            |||||||||||| |||||||||||||||||||||||||||||||||||||||||||||||
Sbjct  259  TAACAAGTACTTGTTATAGTTTGGTTCGATTTGCGTGTTTTTCTTTGTATTCATTTATA  200

Query  268  ttataggtttctatatcatatatttttcCCAATC  301
            |||||||||||||||||||||| |||||||||||
Sbjct  199  TTATAGGTTTCTATATCATATAATTTTCCCAATC  166
```

>GU818385.1 Helenium autumnale isolate 1146 PsbA (psbA) gene, partial cds; psbA-trnH intergenic spacer, complete sequence; and tRNA-His (trnH) gene, partial sequence; plastid
Length=480

Score = 472 bits (255), Expect = 2e-128
Identities = 278/289 (96%), Gaps = 2/289 (1%)
Strand=Plus/Plus

```
Query  12   ACGTAATGCTCATAATTTCCCTCTAGACTTAGCTGCTATTGAAGCTCCATCTACAAATGG  71
            ||||||||||||||||||||||||||||||||||||||||||||||||||||||||||||
Sbjct  1    ACGTAATGCTCATAATTTCCCTCTAGACTTAGCTGCTATTGAAGCTCCATCTACAAATGG  60

Query  72   ATAAGCCTTTGGTCTGATTGTATAGGAGTTTTTGAACTAAAAAAGGAGCAATAACGCCCT  131
            ||||| ||||||||||||||||||||||||||||||||||||||||||||||||||||||
Sbjct  61   ATAAGACTTTGGTCTGATTGTATAGGAGTTTTTGAACTAAAAAAGGAGCAATAACGCCCT  120

Query  132  CTTGATAAAACAAGAGGGAAGCTATTGCTCCttttttttATTTAGTACTATTTGCCTTAC  191
            ||||||||||||||||||||||||||||||| |||||||||||||| |||||| ||||||
Sbjct  121  CTTGATAAAACAAGAGGGAAGCTATTGCTCC-TTTTTTTATTTAGTGCTATTTTCCTTAC  179

Query  192  ATAGTTTCTTTAAAAATAACAAGTACTTTTTATAGTTTGGTTCGATTTGCGTGttttttc  251
            ||||||||||||||||||||||| ||||||| |||||||||| |||||||||||||| |
Sbjct  180  ATAGTTTCTTTAAAAATAACAAGGACTTTTTCTAGTTTGGTTTGATTTGCGTGTTTTCTG  239

Query  252  tttgtattcatttatattataggtttctatatcatatatttttcCCAAT  300
            |||||||||||||||||||||||||||||||||||| | ||||||||||
Sbjct  240  TTTGTATTCATTTATATTATAGGTTTCTATATCATA-ACTTTTCCCAAT  287
```

>NC_081936.1 Guardiola tulocarpus voucher TEX Panero 3062 (TEX) chloroplast, complete genome
 OR069724.1 Guardiola tulocarpus voucher TEX Panero 3062 (TEX) chloroplast, complete genome
Length=151368

 Score = 472 bits (255), Expect = 2e-128
 Identities = 264/268 (99%), Gaps = 2/268 (1%)
 Strand=Plus/Minus

```
Query  2    TTATGCATGAACGTAATGCTCATAATTTCCCTCTAGACTTAGCTGCTATTGAAGCTCCAT  61
            ||||||||||||||||||||||||||||||||||||||||||||||||||||||||||||
Sbjct  595  TTATGCATGAACGTAATGCTCATAATTTCCCTCTAGACTTAGCTGCTATTGAAGCTCCAT  536

Query  62   CTACAAATGGATAAGCCTTTGGTCTGATTGTATAGGAGTTTTTGAACTAAAAAAGGAGCA  121
            ||||||||||||||| ||||||||||||||||||||||||||||||||||||||||||||
Sbjct  535  CTACAAATGGATAAGACTTTGGTCTGATTGTATAGGAGTTTTTGAACTAAAAAAGGAGCA  476

Query  122  ATAACGCCCTCTTGATAAAACAAGAGGGAAGCTATTGCTCCttttttttATTTAGTACTA  181
            ||||||||||||||||||||||||||  ||||||||||||||||||||||||||||||||
Sbjct  475  ATAACGCCCTCTTGATAAAACAAGAG--AAGCTATTGCTCCTTTTTTTTATTTAGTACTA  418

Query  182  TTTGCCTTACATAGTTTCTTTAAAAATAACAAGTACTTTTTATAGTTTGGTTCGATTTGC  241
            ||||||||||||||||||||||||||||||||| ||||||||||||||||||||||||||
Sbjct  417  TTTGCCTTACATAGTTTCTTTAAAAATAACAAGGACTTTTTATAGTTTGGTTCGATTTGC  358

Query  242  GTGttttttctttgtattcatttatatt  269
            ||||||||||||||||||||||||||||
Sbjct  357  GTGTTTTTTCTTTGTATTCATTTATATT  330
```

>MF037490.1 Echinacea angustifolia voucher Rolfsmeier19250 trnH-psbA intergenic spacer region, partial sequence; chloroplast
Length=409

```
 Score = 470 bits (254),  Expect = 7e-128
 Identities = 257/258 (99%), Gaps = 1/258 (0%)
 Strand=Plus/Plus

Query  44   CTGCTATTGAAGCTCCATCTACAAATGGATAAGCCTTTGGTCTGATTGTATAGGAGTTTT  103
            ||||||||||||||||||||||||||||||||||||||||||||||||||||||||||||
Sbjct  1    CTGCTATTGAAGCTCCATCTACAAATGGATAAGCCTTTGGTCTGATTGTATAGGAGTTTT  60

Query  104  TGAACTAAAAAAGGAGCAATAACGCCCTCTTGATAAAACAAGAGGGAAGCTATTGCTCCt  163
            ||||||||||||||||||||||||||||||||||||||||||||||||||||||||||| 
Sbjct  61   TGAACTAAAAAAGGAGCAATAACGCCCTCTTGATAAAACAAGAGGGAAGCTATTGCTCC-  119

Query  164  tttttttATTTAGTACTATTTGCCTTACATAGTTTCTTTAAAAATAACAAGTACTTTTTA  223
            ||||||||||||||||||||||||||||||||||||||||||||||||||||||||||||
Sbjct  120  TTTTTTTATTTAGTACTATTTGCCTTACATAGTTTCTTTAAAAATAACAAGTACTTTTTA  179

Query  224  TAGTTTGGTTCGATTTGCGTGttttttctttgtattcatttatattataggtttctatat  283
            ||||||||||||||||||||||||||||||||||||||||||||||||||||||||||||
Sbjct  180  TAGTTTGGTTCGATTTGCGTGTTTTTTCTTTGTATTCATTTATATTATAGGTTTCTATAT  239

Query  284  catatatttttcCCAATC  301
            ||||||||||||||||||
Sbjct  240  CATATATTTTTCCCAATC  257


>OK469409.1 Eupatorieae sp. Gostel 807 voucher BRIT:Gostel807 photosystem
II protein D1 (psbA) gene, partial cds; psbA-trnH intergenic
spacer, complete sequence; and tRNA-His (trnH) gene, partial
sequence; chloroplast
Length=462

 Score = 470 bits (254),  Expect = 7e-128
 Identities = 271/279 (97%), Gaps = 1/279 (0%)
 Strand=Plus/Plus

Query  23   ATAATTTCCCTCTAGACTTAGCTGCTATTGAAGCTCCATCTACAAATGGATAAGCCTTTG  82
            |||||||||||||||||||||||||||||||||||||||||||||||||||||| |||||
Sbjct  1    ATAATTTCCCTCTAGACTTAGCTGCTATTGAAGCTCCATCTACAAATGGATAAGACTTTG  60

Query  83   GTCTGATTGTATAGGAGTTTTTGAACTAAAAAAGGAGCAATAACGCCCTCTTGATAAAAC  142
            ||||||||||||||||||||||||||||||||||||||||||||||||||||||||||||
Sbjct  61   GTCTGATTGTATAGGAGTTTTTGAACTAAAAAAGGAGCAATAACGCCCTCTTGATAAAAC  120

Query  143  AAGAGGGAAGCTATTGCTCCttttttttATTTAGTACTATTTGCCTTACATAGTTTCTTT  202
            |||||||||||||||||||| ||||||||||||||||||||||||||| ||||||| |||
Sbjct  121  AAGAGGGAAGCTATTGCTCC-TTTTTTTATTTAGTACTATTTGCCTTATATAGTTTATTT  179

Query  203  AAAAATAACAAGTACTTTTTATAGTTTGGTTCGATTTGCGTGttttttctttgtattcat  262
            |||||||||||| |||||||||||||||||||||||||||| ||||||||||||||||||
Sbjct  180  AAAAATAACAAGGACTTTTTATAGTTTGGTTCGATTTGCGTATTTTTTCTTTGTATTCAT  239

Query  263  ttatattataggtttctatatcatatatttttcCCAATC  301
            ||||||||||||||||||||| ||||| |||||||||||
Sbjct  240  TTATATTATAGGTTTCTATATAATATACTTTTCCCAATC  278


>MZ292978.1 Acmella paniculata chloroplast, complete genome
```

```
Length=152117

 Score = 470 bits (254),  Expect = 7e-128
 Identities = 286/301 (95%), Gaps = 3/301 (1%)
 Strand=Plus/Minus

Query  2    TTATGCATGAACGTAATGCTCATAATTTCCCTCTAGACTTAGCTGCTATTGAAGCTCCAT  61
            ||||||||||||||||||||||||||||||||||||||||||||||||||||||||||||
Sbjct  530  TTATGCATGAACGTAATGCTCATAATTTCCCTCTAGACTTAGCTGCTATTGAAGCTCCAT  471

Query  62   CTACAAATGGATAAGCCTTTGGTCTGATTGTATAGGAGTTTTTGAACTAAAAAAGGAGCA  121
            |||| |||||||||| ||||||||||||||||||||||||||||||||||||||||||||
Sbjct  470  CTACCAATGGATAAGACTTTGGTCTGATTGTATAGGAGTTTTTGAACTAAAAAAGGAGCA  411

Query  122  ATAAC-GCCCTCTTGATAAAACAAGAGGGAAGCTATTGCTCCttttttttATTTAGTACT  180
            ||| |  |||||||| |  | ||||||||  | |||||||||||||||| ||||||||||
Sbjct  410  ATAGCTTCCCTCTTGTTTTATCAAGAGGG-CGTTATTGCTCCTTTTTTT-ATTTAGTACT  353

Query  181  ATTTGCCTTACATAGTTTCTTTAAAAATAACAAGTACTTTTTATAGTTTGGTTCGATTTG  240
            ||||||||||||||||||||||||||||||||||||||||||||||||||||||||||||
Sbjct  352  ATTTGCCTTACATAGTTTCTTTAAAAATAACAAGTACTTTTTATAGTTTGGTTCGATTTG  293

Query  241  CGTGttttttctttgtattcatttatattataggtttctatatcatatatttttcCCAAT  300
            ||||||||||||||||||||||||||||||||||||| ||||||||||| ||||||||||
Sbjct  292  CGTGTTTTTTCTTTGTATTCATTTATATTATAGGTTTATATATCATATACTTTTCCCAAT  233

Query  301  C  301
            |
Sbjct  232  C  232


 Score = 110 bits (59),  Expect = 2e-19
 Identities = 59/59 (100%), Gaps = 0/59 (0%)
 Strand=Plus/Plus

Query  110  AAAAAAGGAGCAATAACGCCCTCTTGATAAAACAAGAGGGAAGCTATTGCTCCtttttt  168
            |||||||||||||||||||||||||||||||||||||||||||||||||||||||||||
Sbjct  364  AAAAAAGGAGCAATAACGCCCTCTTGATAAAACAAGAGGGAAGCTATTGCTCCTTTTTT  422


>NC_081937.1 Greenmaniella resinosa voucher Baldwin 977 (UC) chloroplast, 
complete genome
 OR069725.1 Greenmaniella resinosa voucher Baldwin 977 (UC) chloroplast, 
complete genome
Length=152246

 Score = 470 bits (254),  Expect = 7e-128
 Identities = 286/301 (95%), Gaps = 3/301 (1%)
 Strand=Plus/Minus

Query  2    TTATGCATGAACGTAATGCTCATAATTTCCCTCTAGACTTAGCTGCTATTGAAGCTCCAT  61
            ||||||||||||||||||||||||||||||||||||||||||||||||||||||||||||
Sbjct  548  TTATGCATGAACGTAATGCTCATAATTTCCCTCTAGACTTAGCTGCTATTGAAGCTCCAT  489

Query  62   CTACAAATGGATAAGCCTTTGGTCTGATTGTATAGGAGTTTTTGAACTAAAAAAGGAGCA  121
            ||||||||||||||| ||||||||||||||||||||||||||||||||||||||||||||
Sbjct  488  CTACAAATGGATAAGACTTTGGTCTGATTGTATAGGAGTTTTTGAACTAAAAAAGGAGCA  429
```

```
Query  122  ATAACG-CCCTCTTGATAAAACAAGAGGGAAGCTATTGCTCCttttttttATTTAGTACT  180
            ||| |  |||||||| |  | ||||||||  | ||||||||| |||||||||||||||||
Sbjct  428  ATAGCTTCCCTCTTGTTTTATCAAGAGGGC-GTTATTGCTCC-TTTTTTTATTTAGTACT  371

Query  181  ATTTGCCTTACATAGTTTCTTTAAAAATAACAAGTACTTTTTATAGTTTGGTTCGATTTG  240
            |||||||||||||||||||||||||||||||||| |||||||||||||||||||||||||
Sbjct  370  ATTTGCCTTACATAGTTTCTTTAAAAATAACAAGGACTTTTTATAGTTTGGTTCGATTTG  311

Query  241  CGTGttttttctttgtattcatttatattataggtttctatatcatatatttttcCCAAT  300
            |||||||| |||||||||||||||||||||||||||||||||||||||| ||||||||||
Sbjct  310  CGTGTTTTCTCTTTGTATTCATTTATATTATAGGTTTCTATATCATATACTTTTCCCAAT  251

Query  301  C  301
            |
Sbjct  250  C  250


 Score = 110 bits (59),  Expect = 2e-19
 Identities = 59/59 (100%), Gaps = 0/59 (0%)
 Strand=Plus/Plus

Query  110  AAAAAAGGAGCAATAACGCCCTCTTGATAAAACAAGAGGGAAGCTATTGCTCCtttttt  168
            |||||||||||||||||||||||||||||||||||||||||||||||||||||||||||
Sbjct  382  AAAAAAGGAGCAATAACGCCCTCTTGATAAAACAAGAGGGAAGCTATTGCTCCTTTTTT  440


>KF241797.1 Ambrosia artemisiifolia isolate PH-53 PsbA (psbA) gene, partial
cds; and psbA-trnH intergenic spacer, partial sequence; chloroplast
Length=474

 Score = 470 bits (254),  Expect = 7e-128
 Identities = 283/295 (96%), Gaps = 9/295 (3%)
 Strand=Plus/Plus

Query  7    CATGAACGTAATGCTCATAATTTCCCTCTAGACTTAGCTGCTATTGAAGCTCCATCTACA  66
            ||||||||||||||||||||||||||||||||||||||||||||||||||||||||||||
Sbjct  1    CATGAACGTAATGCTCATAATTTCCCTCTAGACTTAGCTGCTATTGAAGCTCCATCTACA  60

Query  67   AATGGATAAGCCTTTGGTCTGATTGTATAGGAGTTTTTGAACTAAAAAAGGAGCAATAAC  126
            |||||||||| |||||||||||||||||||||||||||||||||||||||||||||||||
Sbjct  61   AATGGATAAGACTTTGGTCTGATTGTATAGGAGTTTTTGAACTAAAAAAGGAGCAATAAC  120

Query  127  GCCCTCTTGATAAAACAAGAGGGAAGCTATTGCTCCttttttttATTTAGTACTATTTGC  186
            | ||||||||||||||||||||||||||||||||||||||||||||||||||||||||||
Sbjct  121  GTCCTCTTGATAAAACAAGAGGGAAGCTATTGCTCCTTTTTTTTATTTAGTACTATTTGC  180

Query  187  CTTACATAGTTTCTTTAAAAATAACAAGTACTTTTTATAGTTTGGTTCGATTTGCGTGtt  246
            |||||||||||||||||||||||         ||||||||||||||||||||||||||||
Sbjct  181  CTTACATAGTTTCTTTAAAAATA---------TTTTATAGTTTGGTTCGATTTGCGTGTT  231

Query  247  ttttctttgtattcatttatattataggtttctatatcatatatttttcCCAATC  301
            ||||||||||||||||||||||||||||||||||||||||||| |||||||||||
Sbjct  232  TTTTCTTTGTATTCATTTATATTATAGGTTTCTATATCATATACTTTTCCCAATC  286


>KF241793.1 Ambrosia artemisiifolia isolate NJI-7 PsbA (psbA) gene, partial
```

```
cds; and psbA-trnH intergenic spacer, partial sequence; chloroplast
Length=485

 Score = 470 bits (254),  Expect = 7e-128
 Identities = 283/295 (96%), Gaps = 9/295 (3%)
 Strand=Plus/Plus

Query  7    CATGAACGTAATGCTCATAATTTCCCTCTAGACTTAGCTGCTATTGAAGCTCCATCTACA  66
            ||||||||||||||||||||||||||||||||||||||||||||||||||||||||||||
Sbjct  1    CATGAACGTAATGCTCATAATTTCCCTCTAGACTTAGCTGCTATTGAAGCTCCATCTACA  60

Query  67   AATGGATAAGCCTTTGGTCTGATTGTATAGGAGTTTTTGAACTAAAAAAGGAGCAATAAC  126
            |||||||||| |||||||||||||||||||||||||||||||||||||||||||||||||
Sbjct  61   AATGGATAAGACTTTGGTCTGATTGTATAGGAGTTTTTGAACTAAAAAAGGAGCAATAAC  120

Query  127  GCCCTCTTGATAAAACAAGAGGGAAGCTATTGCTCCttttttttATTTAGTACTATTTGC  186
            | ||||||||||||||||||||||||||||||||||||||||||||||||||||||||||
Sbjct  121  GTCCTCTTGATAAAACAAGAGGGAAGCTATTGCTCCTTTTTTTTATTTAGTACTATTTGC  180

Query  187  CTTACATAGTTTCTTTAAAAATAACAAGTACTTTTTATAGTTTGGTTCGATTTGCGTGtt  246
            |||||||||||||||||||||||         ||||||||||||||||||||||||||||
Sbjct  181  CTTACATAGTTTCTTTAAAAATA---------TTTTATAGTTTGGTTCGATTTGCGTGTT  231

Query  247  ttttctttgtattcatttatattataggtttctatatcatatatttttcCCAATC  301
            ||||||||||||||||||||||||||||||||||||||||||| |||||||||||
Sbjct  232  TTTTCTTTGTATTCATTTATATTATAGGTTTCTATATCATATACTTTTCCCAATC  286


>GU818408.1 Madia sp. 1163 PsbA (psbA) gene, partial cds; psbA-trnH intergenic
spacer, complete sequence; and tRNA-His (trnH) gene, partial
sequence; plastid
Length=510

 Score = 468 bits (253),  Expect = 3e-127
 Identities = 289/305 (95%), Gaps = 7/305 (2%)
 Strand=Plus/Plus

Query  2    TTATGCATGAACGTAATGCTCATAATTTCCCTCTAGACTTAGCTGCTATTGAAGCTCCAT  61
            ||||||||||||||||||||||||||||||||||||||||||||||||||||||||||||
Sbjct  1    TTATGCATGAACGTAATGCTCATAATTTCCCTCTAGACTTAGCTGCTATTGAAGCTCCAT  60

Query  62   CTACAAATGGATAAGCCTTTGGTCTGATTGTATAGGAGTTTTTGAACTAAAAAAGGAGCA  121
            ||||||||||||||| ||||||||||||||||||||||||||||||||||||||||||||
Sbjct  61   CTACAAATGGATAAGACTTTGGTCTGATTGTATAGGAGTTTTTGAACTAAAAAAGGAGCA  120

Query  122  ATAACGCCCTCTTGATAAAACAAGAGGGAAGCTATTGCTCCttttttttATTTAGTACTA  181
            ||||||| ||||||||||||||||||||||||||||||||| ||||||||||||||||||
Sbjct  121  ATAACGCTCTCTTGATAAAACAAGAGGGAAGCTATTGCTCC-TTTTTTTATTTAGTACTA  179

Query  182  TTTGCCTTACATAGTTTCTTTAAAAATAACAAGTACTTTTTATAGTTTGGTTCGATTTGC  241
            ||| ||||||||||||||||| |||||||||||||||||| ||||||| |||||||||||
Sbjct  180  TTTTCCTTACATAGTTTCTTTCAAAATAACAAGTACTTTTGATAGTTTAGTTCGATTTGC  239

Query  242  GTGttttttctttgtattcatttatatt-atag-----gtttctatatcatatatttttc  295
            ||||||||| |||||||||||||||||| |||      |||||||||||||||| |||||
Sbjct  240  GTGTTTTTTATTTGTATTCATTTATATTTATATAATACGTTTCTATATCATATACTTTTC  299
```

```
Query  296  CCAAT  300
            |||||
Sbjct  300  CCAAT  304
```

>AY215541.1 Enydra sessilis voucher Panero 3126 (TEX) psbA gene and psbA-trnH intergenic spacer, partial sequence; chloroplast
Length=428

```
 Score = 466 bits (252),  Expect = 9e-127
 Identities = 267/274 (97%), Gaps = 1/274 (0%)
 Strand=Plus/Minus

Query  28   TTCCCTCTAGACTTAGCTGCTATTGAAGCTCCATCTACAAATGGATAAGCCTTTGGTCTG  87
            ||||||||||||||||||||||||||||||||||||||||||||||||| ||||||||||
Sbjct  428  TTCCCTCTAGACTTAGCTGCTATTGAAGCTCCATCTACAAATGGATAAGACTTTGGTCTG  369

Query  88   ATTGTATAGGAGTTTTTGAACTAAAAAAGGAGCAATAACGCCCTCTTGATAAAACAAGAG  147
            ||||||||||||||||||||||||||||||||||||||||||||||||||||||||||||
Sbjct  368  ATTGTATAGGAGTTTTTGAACTAAAAAAGGAGCAATAACGCCCTCTTGATAAAACAAGAG  309

Query  148  GGAAGCTATTGCTCCttttttttATTTAGTACTATTTGCCTTACATAGTTTCTTTAAAAA  207
            ||||||||||||||||||||||||||||||||||||||||||||||||||||||||||||
Sbjct  308  GGAAGCTATTGCTCCTTTTTTTTATTTAGTACTATTTGCCTTACATAGTTTCTTTAAAAA  249

Query  208  TAACAAGTACTTTTTATAGTTTGGTTCGATTTGCGTGttttttctttgtattcatttata  267
            ||||||| ||||||| ||||||||||||||||||||||||| ||||||||||||||||||
Sbjct  248  TAACAAGGACTTTTTCTAGTTTGGTTCGATTTGCGTGTTTTATCTTTGTATTCATTTATA  189

Query  268  ttataggtttctatatcatatatttttcCC-AAT  300
            ||||||||||||||||||| || ||||||| |||
Sbjct  188  TTATAGGTTTCTATATCATTTACTTTTCCCCAAT  155
```

>KX346912.1 Xanthium sibiricum voucher S-CEZi-04 PsbA (psbA) gene, partial cds; and psbA-trnH intergenic spacer, partial sequence; chloroplast
Length=529

```
 Score = 466 bits (252),  Expect = 9e-127
 Identities = 264/270 (98%), Gaps = 0/270 (0%)
 Strand=Plus/Plus

Query  2    TTATGCATGAACGTAATGCTCATAATTTCCCTCTAGACTTAGCTGCTATTGAAGCTCCAT  61
            ||||||||||||||||||||||||||||||||||||||||||||||||||||||||||||
Sbjct  1    TTATGCATGAACGTAATGCTCATAATTTCCCTCTAGACTTAGCTGCTATTGAAGCTCCAT  60

Query  62   CTACAAATGGATAAGCCTTTGGTCTGATTGTATAGGAGTTTTTGAACTAAAAAAGGAGCA  121
            ||||||||||||||| |||||||||||||||||| |||||||||||||||||||||||||
Sbjct  61   CTACAAATGGATAAGACTTTGGTCTGATTGTATAAGAGTTTTTGAACTAAAAAAGGAGCA  120

Query  122  ATAACGCCCTCTTGATAAAACAAGAGGGAAGCTATTGCTCCttttttttATTTAGTACTA  181
            ||||||||||||||||||||||||||||||||||||||||||||||||||||||||||||
Sbjct  121  ATAACGCCCTCTTGATAAAACAAGAGGGAAGCTATTGCTCCTTTTTTTTATTTAGTACTA  180

Query  182  TTTGCCTTACATAGTTTCTTTAAAAATAACAAGTACTTTTTATAGTTTGGTTCGATTTGC  241
            ||||||||||||||||||||||||||||| ||||||||||||||||||| ||  ||||||
Sbjct  181  TTTGCCTTACATAGTTTCTTTAAAAATAAAAAGTACTTTTTATAGTTTGTTTTAATTTGC  240
```

```
Query  242  GTGtttttctttgtattcatttatattat  271
            ||||||||||||||||||||||||||||||
Sbjct  241  GTGTTTTTCTTTGTATTCATTTATATTAT  270
```

>OQ832296.1 Xanthium strumarium voucher MONT:87396 photosystem II protein D1 (psbA) gene, partial cds; psbA-trnH intergenic spacer, complete sequence; and tRNA-His (trnH) gene, partial sequence; chloroplast
Length=480

Score = 466 bits (252), Expect = 9e-127
Identities = 270/279 (97%), Gaps = 0/279 (0%)
Strand=Plus/Plus

```
Query  23   ATAATTTCCCTCTAGACTTAGCTGCTATTGAAGCTCCATCTACAAATGGATAAGCCTTTG  82
            |||||||||||||||||||||||||||||||||||||||||||||||||||||| |||||
Sbjct  1    ATAATTTCCCTCTAGACTTAGCTGCTATTGAAGCTCCATCTACAAATGGATAAGACTTTG  60

Query  83   GTCTGATTGTATAGGAGTTTTTGAACTAAAAAAGGAGCAATAACGCCCTCTTGATAAAAC  142
            ||||||||||||| ||||||||||||||||||||||||||||||||||||||||||||||
Sbjct  61   GTCTGATTGTATAAGAGTTTTTGAACTAAAAAAGGAGCAATAACGCCCTCTTGATAAAAC  120

Query  143  AAGAGGGAAGCTATTGCTCCttttttttATTTAGTACTATTTGCCTTACATAGTTTCTTT  202
            ||||||||||||||||||||||||||||||||||||||||||||||||||||||||||||
Sbjct  121  AAGAGGGAAGCTATTGCTCCTTTTTTTTATTTAGTACTATTTGCCTTACATAGTTTCTTT  180

Query  203  AAAAATAACAAGTACTTTTTATAGTTTGGTTCGATTTGCGTGtttttctttgtattcat  262
            |||||| | ||||||||||||||||||| ||  |||||||||||||||||||||||||||
Sbjct  181  AAAAATCAAAAGTACTTTTTATAGTTTGTTTTAATTTGCGTGTTTTTCTTTGTATTCAT  240

Query  263  ttatattataggtttctatatcatatattttcCCAATC  301
            |||||||||| |||||||||||||||| |||||||||||
Sbjct  241  TTATATTATATGTTTCTATATCATATACTTTTCCCAATC  279
```

>KX261083.1 Platyschkuhria integrifolia var. integrifolia isolate 1 PsbA gene, partial cds; and psbA-trnH intergenic spacer, partial sequence; chloroplast
Length=511

Score = 466 bits (252), Expect = 9e-127
Identities = 276/288 (96%), Gaps = 0/288 (0%)
Strand=Plus/Plus

```
Query  14   GTAATGCTCATAATTTCCCTCTAGACTTAGCTGCTATTGAAGCTCCATCTACAAATGGAT  73
            ||||||||| |||||||||||||||||||||||||||||||| |||||||||||||||||
Sbjct  1    GTAATGCTCGTAATTTCCCTCTAGACTTAGCTGCTATTGAAGTTCCATCTACAAATGGAT  60

Query  74   AAGCCTTTGGTCTGATTGTATAGGAGTTTTTGAACTAAAAAAGGAGCAATAACGCCCTCT  133
            |||  |||||||||||||||||||| ||||||||||||||||||||||||||||||||||
Sbjct  61   AAGTTTTTGGTCTGATTGTATAGGATTTTTTGAACTAAAAAAGGAGCAATAACGCCCTCT  120

Query  134  TGATAAAACAAGAGGGAAGCTATTGCTCCttttttttATTTAGTACTATTTGCCTTACAT  193
            ||||||||||||||||||||||||||||||||||||||||||||||||||||||||||||
Sbjct  121  TGATAAAACAAGAGGGAAGCTATTGCTCCTTTTTTTTATTTAGTACTATTTGCCTTACAT  180
```

```
Query  194  AGTTTCTTTAAAAATAACAAGTACTTTTTATAGTTTGGTTCGATTTGCGTGttttttctt  253
            |||||||||||||||||||||  |||||||||||||||||||||||||||||||| ||||
Sbjct  181  AGTTTCTTTAAAAATAACAAGGGCTTTTTATAGTTTGGTTCGATTTGCGTGTTTTCTCTT  240

Query  254  tgtattcatttatattataggtttctatatcatatatttttcCCAATC  301
            ||||||| ||||| |||||||||||||| ||||||| |||||||||||
Sbjct  241  TGTATTCTTTTATCTTATAGGTTTCTATCTCATATACTTTTCCCAATC  288
```

>KX261084.1 Platyschkuhria integrifolia var. integrifolia isolate 2 PsbA gene, partial cds; and psbA-trnH intergenic spacer, partial sequence; chloroplast
Length=511

```
 Score = 466 bits (252),  Expect = 9e-127
 Identities = 276/288 (96%), Gaps = 0/288 (0%)
 Strand=Plus/Plus

Query  14   GTAATGCTCATAATTTCCCTCTAGACTTAGCTGCTATTGAAGCTCCATCTACAAATGGAT  73
            ||||||||| |||||||||||||||||||||||||||||||| |||||||||||||||||
Sbjct  1    GTAATGCTCGTAATTTCCCTCTAGACTTAGCTGCTATTGAAGTTCCATCTACAAATGGAT  60

Query  74   AAGCCTTTGGTCTGATTGTATAGGAGTTTTTGAACTAAAAAAGGAGCAATAACGCCCTCT  133
            |||  |||||||||||||||||||| ||||||||||||||||||||||||||||||||||
Sbjct  61   AAGTTTTTGGTCTGATTGTATAGGATTTTTTGAACTAAAAAAGGAGCAATAACGCCCTCT  120

Query  134  TGATAAAACAAGAGGGAAGCTATTGCTCCtttttttATTTAGTACTATTTGCCTTACAT  193
            ||||||||||||||||||||||||||||||||||||||||||||||||||||||||||||
Sbjct  121  TGATAAAACAAGAGGGAAGCTATTGCTCCTTTTTTTTATTTAGTACTATTTGCCTTACAT  180

Query  194  AGTTTCTTTAAAAATAACAAGTACTTTTTATAGTTTGGTTCGATTTGCGTGttttttctt  253
            |||||||||||||||||||||  |||||||||||||||||||||||||||||||| ||||
Sbjct  181  AGTTTCTTTAAAAATAACAAGGGCTTTTTATAGTTTGGTTCGATTTGCGTGTTTTCTCTT  240

Query  254  tgtattcatttatattataggtttctatatcatatatttttcCCAATC  301
            ||||||| ||||| |||||||||||||| ||||||| |||||||||||
Sbjct  241  TGTATTCTTTTATCTTATAGGTTTCTATCTCATATACTTTTCCCAATC  288
```

>OQ404763.1 Ambrosia artemisiifolia trnH-psbA intergenic spacer, partial sequence; and photosystem II protein D1 (psbA) gene, partial cds; plastid
Length=435

```
 Score = 466 bits (252),  Expect = 9e-127
 Identities = 283/296 (96%), Gaps = 9/296 (3%)
 Strand=Plus/Minus

Query  6    GCATGAACGTAATGCTCATAATTTCCCTCTAGACTTAGCTGCTATTGAAGCTCCATCTAC  65
            ||| ||||||||||||||||||||||||||||||||||||||||||||||||||||||||
Sbjct  435  GCACGAACGTAATGCTCATAATTTCCCTCTAGACTTAGCTGCTATTGAAGCTCCATCTAC  376

Query  66   AAATGGATAAGCCTTTGGTCTGATTGTATAGGAGTTTTTGAACTAAAAAAGGAGCAATAA  125
            ||||||||||| ||||||||||||||||||||||||||||||||||||||||||||||||
Sbjct  375  AAATGGATAAGACTTTGGTCTGATTGTATAGGAGTTTTTGAACTAAAAAAGGAGCAATAA  316
```

```
Query  126  CGCCCTCTTGATAAAACAAGAGGGAAGCTATTGCTCCttttttttATTTAGTACTATTTG  185
            || |||||||||||||||||||||||||||||||||||||||||||||||||||||||||
Sbjct  315  CGTCCTCTTGATAAAACAAGAGGGAAGCTATTGCTCCTTTTTTTTATTTAGTACTATTTG  256

Query  186  CCTTACATAGTTTCTTTAAAAATAACAAGTACTTTTTATAGTTTGGTTCGATTTGCGTGt  245
            ||||||||||||||||||||||||         |||||||||||||||||||||||||||
Sbjct  255  CCTTACATAGTTTCTTTAAAAATA---------TTTTATAGTTTGGTTCGATTTGCGTGT  205

Query  246  tttttctttgtattcatttatattataggtttctatatcatatatttttcCCAATC  301
            |||||||||||||||||||||||||||||||||||||||||||| |||||||||||
Sbjct  204  TTTTTCTTTGTATTCATTTATATTATAGGTTTCTATATCATATACTTTTCCCAATC  149


>NC_072298.1 Xanthium strumarium chloroplast, complete genome
 OP606043.1 Xanthium strumarium chloroplast, complete genome
Length=151852

 Score = 466 bits (252),  Expect = 9e-127
 Identities = 264/270 (98%), Gaps = 0/270 (0%)
 Strand=Plus/Minus

Query  2    TTATGCATGAACGTAATGCTCATAATTTCCCTCTAGACTTAGCTGCTATTGAAGCTCCAT  61
            ||||||||||||||||||||||||||||||||||||||||||||||||||||||||||||
Sbjct  552  TTATGCATGAACGTAATGCTCATAATTTCCCTCTAGACTTAGCTGCTATTGAAGCTCCAT  493

Query  62   CTACAAATGGATAAGCCTTTGGTCTGATTGTATAGGAGTTTTTGAACTAAAAAAGGAGCA  121
            ||||||||||||||| |||||||||||||||||| |||||||||||||||||||||||||
Sbjct  492  CTACAAATGGATAAGACTTTGGTCTGATTGTATAAGAGTTTTTGAACTAAAAAAGGAGCA  433

Query  122  ATAACGCCCTCTTGATAAAACAAGAGGGAAGCTATTGCTCCttttttttATTTAGTACTA  181
            ||||||||||||||||||||||||||||||||||||||||||||||||||||||||||||
Sbjct  432  ATAACGCCCTCTTGATAAAACAAGAGGGAAGCTATTGCTCCTTTTTTTTATTTAGTACTA  373

Query  182  TTTGCCTTACATAGTTTCTTTAAAAATAACAAGTACTTTTTATAGTTTGGTTCGATTTGC  241
            ||||||||||||||||||||||||||||| ||||||||||||||||||| ||  ||||||
Sbjct  372  TTTGCCTTACATAGTTTCTTTAAAAATAAAAAGTACTTTTTATAGTTTGTTTTAATTTGC  313

Query  242  GTGttttttctttgtattcatttatattat  271
            ||||||||||||||||||||||||||||||
Sbjct  312  GTGTTTTTTCTTTGTATTCATTTATATTAT  283


>PP639077.1 Verbesina alternifolia chloroplast, complete genome
Length=152050

 Score = 466 bits (252),  Expect = 9e-127
 Identities = 286/301 (95%), Gaps = 8/301 (3%)
 Strand=Plus/Minus

Query  2    TTATGCATGAACGTAATGCTCATAATTTCCCTCTAGACTTAGCTGCTATTGAAGCTCCAT  61
            ||||||||||||||||||||||||||||||||||||||||||||||||||||||||||||
Sbjct  528  TTATGCATGAACGTAATGCTCATAATTTCCCTCTAGACTTAGCTGCTATTGAAGCTCCAT  469

Query  62   CTACAAATGGATAAGCCTTTGGTCTGATTGTA------TAGGAGTTTTTGAACTAAAAAA  115
            ||||||||||||||| ||| ||||||||||||      ||||||||||||||||||||||
Sbjct  468  CTACAAATGGATAAGACTTCGGTCTGATTGTAATTGTATAGGAGTTTTTGAACTAAAAAA  409
```

```
Query  116  GG-AGCAATAACGCCCTCTTGATAAAACAAGAGGGAAGCTATTGCTCCttttttttATTT  174
            || |||||||||||||||||||||||||||||||||||||||||||||||||||| ||||
Sbjct  408  GGGAGCAATAACGCCCTCTTGATAAAACAAGAGGGAAGCTATTGCTCCTTTTTTT-ATTT  350

Query  175  AGTACTATTTGCCTTACATAGTTTCTTTAAAAATAACAAGTACTTTTTATAGTTTGGTTC  234
            |||||||||||||||||||||||||||||||||||||||| ||||||||| |||||||||
Sbjct  349  AGTACTATTTGCCTTACATAGTTTCTTTAAAAATAACAAGGACTTTTTATTGTTTGGTTC  290

Query  235  GATTTGCGTGttttttctttgtattcatttatattataggtttctatatcatatattttt  294
            | |||||||||||||||||||||||||||||||||||||||||||||| |||||| ||||
Sbjct  289  GGTTTGCGTGTTTTTTCTTTGTATTCATTTATATTATAGGTTTCTATACCATATACTTTT  230

Query  295  c  295
            |
Sbjct  229  C  229
```

>NC_042232.1 Xanthium sibiricum chloroplast, complete genome
 MH473582.1 Xanthium sibiricum chloroplast, complete genome
Length=151897

 Score = 466 bits (252), Expect = 9e-127
 Identities = 264/270 (98%), Gaps = 0/270 (0%)
 Strand=Plus/Minus

```
Query  2    TTATGCATGAACGTAATGCTCATAATTTCCCTCTAGACTTAGCTGCTATTGAAGCTCCAT  61
            ||||||||||||||||||||||||||||||||||||||||||||||||||||||||||||
Sbjct  552  TTATGCATGAACGTAATGCTCATAATTTCCCTCTAGACTTAGCTGCTATTGAAGCTCCAT  493

Query  62   CTACAAATGGATAAGCCTTTGGTCTGATTGTATAGGAGTTTTTGAACTAAAAAAGGAGCA  121
            |||||||||||||| ||||||||||||||||||| |||||||||||||||||||||||||
Sbjct  492  CTACAAATGGATAAGACTTTGGTCTGATTGTATAAGAGTTTTTGAACTAAAAAAGGAGCA  433

Query  122  ATAACGCCCTCTTGATAAAACAAGAGGGAAGCTATTGCTCCttttttttATTTAGTACTA  181
            ||||||||||||||||||||||||||||||||||||||||||||||||||||||||||||
Sbjct  432  ATAACGCCCTCTTGATAAAACAAGAGGGAAGCTATTGCTCCTTTTTTTTATTTAGTACTA  373

Query  182  TTTGCCTTACATAGTTTCTTTAAAAATAACAAGTACTTTTTATAGTTTGGTTCGATTTGC  241
            ||||||||||||||||||||||||||||| ||||||||||||||||||| ||  ||||||
Sbjct  372  TTTGCCTTACATAGTTTCTTTAAAAATAAAAAGTACTTTTTATAGTTTGTTTTAATTTGC  313

Query  242  GTGtttttctttgtattcatttatattat  271
            ||||||||||||||||||||||||||||||
Sbjct  312  GTGTTTTTTCTTTGTATTCATTTATATTAT  283
```

>KF241794.1 Ambrosia artemisiifolia isolate mb-46 PsbA (psbA) gene, partial
cds; and psbA-trnH intergenic spacer, partial sequence; chloroplast
Length=414

 Score = 464 bits (251), Expect = 3e-126
 Identities = 282/295 (96%), Gaps = 9/295 (3%)
 Strand=Plus/Plus

```
Query  7    CATGAACGTAATGCTCATAATTTCCCTCTAGACTTAGCTGCTATTGAAGCTCCATCTACA  66
            ||||||||||||||||||||||||||||||||||||||||||||||||||||||||||||
Sbjct  1    CATGAACGTAATGCTCATAATTTCCCTCTAGACTTAGCTGCTATTGAAGCTCCATCTACA  60
```

```
Query  67   AATGGATAAGCCTTTGGTCTGATTGTATAGGAGTTTTTGAACTAAAAAAGGAGCAATAAC  126
            |||||||||| |||||||||||||||||||||||||||||||||||||||||||||||||
Sbjct  61   AATGGATAAGACTTTGGTCTGATTGTATAGGAGTTTTTGAACTAAAAAAGGAGCAATAAC  120

Query  127  GCCCTCTTGATAAAACAAGAGGGAAGCTATTGCTCCttttttttATTTAGTACTATTTGC  186
            | ||||||||||||||||||||||||||||||||||||||||||||||||||||||||||
Sbjct  121  GTCCTCTTGATAAAACAAGAGGGAAGCTATTGCTCCTTTTTTTTATTTAGTACTATTTGC  180

Query  187  CTTACATAGTTTCTTTAAAAATAACAAGTACTTTTTATAGTTTGGTTCGATTTGCGTGtt  246
            |||||||||||||||||||||||         ||||||||||||||||||||||||||||
Sbjct  181  CTTACATAGTTTCTTTAAAAATA---------TTTTATAGTTTGGTTCGATTTGCGTGTT  231

Query  247  ttttctttgtattcatttatattataggtttctatatcatatatttttcCCAATC  301
            |||||||||||||||||| |||||||||||||||||||||||| |||||||||||
Sbjct  232  TTTTCTTTGTATTCATTTCTATTATAGGTTTCTATATCATATACTTTTCCCAATC  286
```

```
>OQ434029.1 Ambrosia trifida clone 1039837_5 trnH-psbA intergenic spacer,
partial sequence; and photosystem II protein D1 (psbA) gene,
partial cds; plastid
Length=432

 Score = 464 bits (251),  Expect = 3e-126
 Identities = 280/292 (96%), Gaps = 9/292 (3%)
 Strand=Plus/Minus

Query  10   GAACGTAATGCTCATAATTTCCCTCTAGACTTAGCTGCTATTGAAGCTCCATCTACAAAT  69
            ||||||||||||||||||||||||||||||||||||||||||||||||||||||||||||
Sbjct  432  GAACGTAATGCTCATAATTTCCCTCTAGACTTAGCTGCTATTGAAGCTCCATCTACAAAT  373

Query  70   GGATAAGCCTTTGGTCTGATTGTATAGGAGTTTTTGAACTAAAAAAGGAGCAATAACGCC  129
            ||||||| |||||||||||||||||||||||||||||||||||||||||||||||||| |
Sbjct  372  GGATAAGACTTTGGTCTGATTGTATAGGAGTTTTTGAACTAAAAAAGGAGCAATAACGTC  313

Query  130  CTCTTGATAAAACAAGAGGGAAGCTATTGCTCCttttttttATTTAGTACTATTTGCCTT  189
            ||||||||||||||||||||||||||||||||||||||||||||||||||||||||||||
Sbjct  312  CTCTTGATAAAACAAGAGGGAAGCTATTGCTCCTTTTTTTTATTTAGTACTATTTGCCTT  253

Query  190  ACATAGTTTCTTTAAAAATAACAAGTACTTTTTATAGTTTGGTTCGATTTGCGTGttttt  249
            ||||||||||||||||||||         |||||||||||||||||||||||||||||||
Sbjct  252  ACATAGTTTCTTTAAAAATA---------TTTTATAGTTTGGTTCGATTTGCGTGTTTTT  202

Query  250  tctttgtattcatttatattataggtttctatatcatatatttttcCCAATC  301
            |||||||||||||||||||||||||||||||||||||||| |||||||||||
Sbjct  201  TCTTTGTATTCATTTATATTATAGGTTTCTATATCATATACTTTTCCCAATC  150
```

```
>MF037494.1 Echinacea sanguinea voucher Bengtson9 trnH-psbA intergenic spacer
region, partial sequence; chloroplast
Length=407

 Score = 464 bits (251),  Expect = 3e-126
 Identities = 256/258 (99%), Gaps = 1/258 (0%)
 Strand=Plus/Plus

Query  44   CTGCTATTGAAGCTCCATCTACAAATGGATAAGCCTTTGGTCTGATTGTATAGGAGTTTT  103
```

```
            ||||||||||||||||||||||||||||||||||||||||||||||||||||||||||||
Sbjct  1    CTGCTATTGAAGCTCCATCTACAAATGGATAAGCCTTTGGTCTGATTGTATAGGAGTTTT  60

Query  104  TGAACTAAAAAAGGAGCAATAACGCCCTCTTGATAAAACAAGAGGGAAGCTATTGCTCCt  163
            ||||||||||||||||||||||||||||||||||||||||||||||||||||||||||| 
Sbjct  61   TGAACTAAAAAAGGAGCAATAACGCCCTCTTGATAAAACAAGAGGGAAGCTATTGCTCC-  119

Query  164  tttttttATTTAGTACTATTTGCCTTACATAGTTTCTTTAAAAATAACAAGTACTTTTTA  223
            ||||||||||||||||||||||||||||||||||||||||||||||||||||||||||||
Sbjct  120  TTTTTTTATTTAGTACTATTTGCCTTACATAGTTTCTTTAAAAATAACAAGTACTTTTTA  179

Query  224  TAGTTTGGTTCGATTTGCGTGtttttttctttgtattcatttatattataggtttctatat  283
            ||||||||||||||||||||||||||||||||||||||||||||||||||||||||||||
Sbjct  180  TAGTTTGGTTCGATTTGCGTGTTTTTTCTTTGTATTCATTTATATTATAGGTTTCTATAT  239

Query  284  catatatttttcCCAATC  301
            ||| ||||||||||||||
Sbjct  240  CATTTATTTTTCCCAATC  257


>KF241795.1 Ambrosia artemisiifolia isolate NJF-12 PsbA (psbA) gene, partial
cds; and psbA-trnH intergenic spacer, partial sequence;
chloroplast
Length=445

 Score = 464 bits (251),  Expect = 3e-126
 Identities = 282/295 (96%), Gaps = 10/295 (3%)
 Strand=Plus/Plus

Query  7    CATGAACGTAATGCTCATAATTTCCCTCTAGACTTAGCTGCTATTGAAGCTCCATCTACA  66
            ||||||||||||||||||||||||||||||||||||||||||||||||||||||||||||
Sbjct  1    CATGAACGTAATGCTCATAATTTCCCTCTAGACTTAGCTGCTATTGAAGCTCCATCTACA  60

Query  67   AATGGATAAGCCTTTGGTCTGATTGTATAGGAGTTTTTGAACTAAAAAAGGAGCAATAAC  126
            |||||||||| |||||||||||||||||||||||||||||||||||||||||||||||||
Sbjct  61   AATGGATAAGACTTTGGTCTGATTGTATAGGAGTTTTTGAACTAAAAAAGGAGCAATAAC  120

Query  127  GCCCTCTTGATAAAACAAGAGGGAAGCTATTGCTCCtttttttATTTAGTACTATTTGC  186
            | |||||||||||||||||||||||||||||||||| |||||||||||||||||||||||
Sbjct  121  GTCCTCTTGATAAAACAAGAGGGAAGCTATTGCTCC-TTTTTTTATTTAGTACTATTTGC  179

Query  187  CTTACATAGTTTCTTTAAAAATAACAAGTACTTTTTATAGTTTGGTTCGATTTGCGTGtt  246
            |||||||||||||||||||||||         ||||||||||||||||||||||||||||
Sbjct  180  CTTACATAGTTTCTTTAAAAATA---------TTTTATAGTTTGGTTCGATTTGCGTGTT  230

Query  247  ttttctttgtattcatttatattataggtttctatatcatatatttttcCCAATC  301
            ||||||||||||||||||||||||||||||||||||||||||| |||||||||||
Sbjct  231  TTTTCTTTGTATTCATTTATATTATAGGTTTCTATATCATATACTTTTCCCAATC  285


>NC_080520.1 Synotis erythropappa chloroplast, complete genome
 OQ985056.1 Synotis erythropappa chloroplast, complete genome
Length=151228

 Score = 462 bits (250),  Expect = 1e-125
 Identities = 263/269 (98%), Gaps = 1/269 (0%)
 Strand=Plus/Minus
```

```
Query  2    TTATGCATGAACGTAATGCTCATAATTTCCCTCTAGACTTAGCTGCTATTGAAGCTCCAT  61
            ||||||||||||||||||||||||||||||||||||||||||||||||||||||||||||
Sbjct  496  TTATGCATGAACGTAATGCTCATAATTTCCCTCTAGACTTAGCTGCTATTGAAGCTCCAT  437

Query  62   CTACAAATGGATAAGCCTTTGGTCTGATTGTATAGGAGTTTTTGAACTAAAAAAGGAGCA  121
            ||||||||||||||| ||||||||||||||||||||||||||||||||||||||||||||
Sbjct  436  CTACAAATGGATAAGACTTTGGTCTGATTGTATAGGAGTTTTTGAACTAAAAAAGGAGCA  377

Query  122  ATAACGCCCTCTTGATAAAACAAGAGGGAAGCTATTGCTCCttttttttATTTAGTACTA  181
            ||||||||||||||||||||||||||||||||||||||||||||||||||||||||||||
Sbjct  376  ATAACGCCCTCTTGATAAAACAAGAGGGAAGCTATTGCTCCTTTTTTTTATTTAGTACTA  317

Query  182  TTTGCCTTACATAGTTTCTTTAAAAATAACAAGTACTTTTTATAGTTTGGTTCGATTTGC  241
            ||||  ||||||||||||||||||||||||||| |||||||||| |||||||||||| ||
Sbjct  316  TTTGT-TTACATAGTTTCTTTAAAAATAACAAGGACTTTTTATAATTTGGTTCGATTCGC  258

Query  242  GTGtttttctttgtattcatttatatta  270
            |||||||||||||||||||||||||||||
Sbjct  257  GTGTTTTTTCTTTGTATTCATTTATATTA  229
```

>EF538052.1 Synotis nagensium PsbA (psbA) gene, partial cds; psbA-trnH intergenic spacer, complete sequence; and tRNA-His (trnH) gene, partial sequence; chloroplast
Length=543

Score = 462 bits (250), Expect = 1e-125
Identities = 263/269 (98%), Gaps = 1/269 (0%)
Strand=Plus/Plus

```
Query  2    TTATGCATGAACGTAATGCTCATAATTTCCCTCTAGACTTAGCTGCTATTGAAGCTCCAT  61
            ||||||||||||||||||||||||||||||||||||||||||||||||||||||||||||
Sbjct  3    TTATGCATGAACGTAATGCTCATAATTTCCCTCTAGACTTAGCTGCTATTGAAGCTCCAT  62

Query  62   CTACAAATGGATAAGCCTTTGGTCTGATTGTATAGGAGTTTTTGAACTAAAAAAGGAGCA  121
            ||||||||||||||| ||||||||||||||||||||||||||||||||||||||||||||
Sbjct  63   CTACAAATGGATAAGACTTTGGTCTGATTGTATAGGAGTTTTTGAACTAAAAAAGGAGCA  122

Query  122  ATAACGCCCTCTTGATAAAACAAGAGGGAAGCTATTGCTCCttttttttATTTAGTACTA  181
            ||||||||||||||||||||||||||||||||||||||||||||||||||||||||||||
Sbjct  123  ATAACGCCCTCTTGATAAAACAAGAGGGAAGCTATTGCTCCTTTTTTTTATTTAGTACTA  182

Query  182  TTTGCCTTACATAGTTTCTTTAAAAATAACAAGTACTTTTTATAGTTTGGTTCGATTTGC  241
            ||||  ||||||||||||||||||||||||||| |||||||||| |||||||||||| ||
Sbjct  183  TTTGT-TTACATAGTTTCTTTAAAAATAACAAGGACTTTTTATAATTTGGTTCGATTCGC  241

Query  242  GTGtttttctttgtattcatttatatta  270
            |||||||||||||||||||||||||||||
Sbjct  242  GTGTTTTTTCTTTGTATTCATTTATATTA  270
```

>MF143709.1 Galinsoga parviflora voucher Li ZY, Xiang XG, Xu SZ 201509216 PsbA (psbA) gene, partial cds; and psbA-trnH intergenic spacer, partial sequence; chloroplast
Length=417

```
 Score = 462 bits (250),  Expect = 1e-125
 Identities = 278/291 (96%), Gaps = 3/291 (1%)
 Strand=Plus/Plus

Query  13   CGTAATGCTCATAATTTCCCTCTAGACTTAGCTGCTATTGAAGCTCCATCTACAAATGGA  72
            ||||||||||||||||||||||||||||||||||||||||||||||||||||||||||||
Sbjct  1    CGTAATGCTCATAATTTCCCTCTAGACTTAGCTGCTATTGAAGCTCCATCTACAAATGGA  60

Query  73   TAAGCCTTTGGTCTGATTGTATAGGAGTTTTTGAACTAAAAAAGGAGCAATAACGCCCTC  132
            |||| |||||||||||||||||||||||||||||||||||||||||||||||||||||||
Sbjct  61   TAAGACTTTGGTCTGATTGTATAGGAGTTTTTGAACTAAAAAAGGAGCAATAACGCCCTC  120

Query  133  TTGATAAAACAAGAGGGAAGCTATTGCTCCttttttttATTTAGTACTATTTGCCTTACA  192
            || ||||||||||||||||||||||||||| |||||||||||||||||||||||||| ||
Sbjct  121  TTCATAAAACAAGAGGGAAGCTATTGCTCC-TTTTTTTATTTAGTACTATTTGCCTTCCA  179

Query  193  TAGTTTCTTTAAAAATAACAAGTACTTTTTATAGTTTGGTTCGATTTGCGTG--tttttt  250
            |||||||||||||||||||||| ||||||| ||||||||||| ||||| ||   ||||||
Sbjct  180  TAGTTTCTTTAAAAATAACAAGGACTTTTTCTAGTTTGGTTCTATTTGTGTTTTTTTTTT  239

Query  251  ctttgtattcatttatattataggtttctatatcatatattttcCCAATC  301
            |||| ||||||||||||||||||||||||||||||||||| |||||||||||
Sbjct  240  CTTTATATTCATTTATATTATAGGTTTCTATATCATATACTTTTCCCAATC  290


>NC_069230.1 Synotis nagensium chloroplast, complete genome
 OM912603.1 Synotis nagensium chloroplast, complete genome
Length=151298

 Score = 462 bits (250),  Expect = 1e-125
 Identities = 263/269 (98%), Gaps = 1/269 (0%)
 Strand=Plus/Minus

Query  2    TTATGCATGAACGTAATGCTCATAATTTCCCTCTAGACTTAGCTGCTATTGAAGCTCCAT  61
            ||||||||||||||||||||||||||||||||||||||||||||||||||||||||||||
Sbjct  566  TTATGCATGAACGTAATGCTCATAATTTCCCTCTAGACTTAGCTGCTATTGAAGCTCCAT  507

Query  62   CTACAAATGGATAAGCCTTTGGTCTGATTGTATAGGAGTTTTTGAACTAAAAAAGGAGCA  121
            ||||||||||||||| ||||||||||||||||||||||||||||||||||||||||||||
Sbjct  506  CTACAAATGGATAAGACTTTGGTCTGATTGTATAGGAGTTTTTGAACTAAAAAAGGAGCA  447

Query  122  ATAACGCCCTCTTGATAAAACAAGAGGGAAGCTATTGCTCCttttttttATTTAGTACTA  181
            ||||||||||||||||||||||||||||||||||||||||||||||||||||||||||||
Sbjct  446  ATAACGCCCTCTTGATAAAACAAGAGGGAAGCTATTGCTCCTTTTTTTTATTTAGTACTA  387

Query  182  TTTGCCTTACATAGTTTCTTTAAAAATAACAAGTACTTTTTATAGTTTGGTTCGATTTGC  241
            ||||  ||||||||||||||||||||||||||| |||||||||| |||||||||||| ||
Sbjct  386  TTTGT-TTACATAGTTTCTTTAAAAATAACAAGGACTTTTTATAATTTGGTTCGATTCGC  328

Query  242  GTGtttttctttgtattcatttatatta  270
            |||||||||||||||||||||||||||||
Sbjct  327  GTGTTTTTCTTTGTATTCATTTATATTA  299


>KF241812.1 Ambrosia artemisiifolia isolate AZ-2 PsbA (psbA) gene, partial
cds; and psbA-trnH intergenic spacer, partial sequence; chloroplast
Length=460
```

```
 Score = 462 bits (250),  Expect = 1e-125
 Identities = 281/294 (96%), Gaps = 9/294 (3%)
 Strand=Plus/Plus

Query  8    ATGAACGTAATGCTCATAATTTCCCTCTAGACTTAGCTGCTATTGAAGCTCCATCTACAA  67
            ||||||||||||||||||||||||||||||||||||||||||||||||||| ||||||||
Sbjct  1    ATGAACGTAATGCTCATAATTTCCCTCTAGACTTAGCTGCTATTGAAGCTCAATCTACAA  60

Query  68   ATGGATAAGCCTTTGGTCTGATTGTATAGGAGTTTTTGAACTAAAAAAGGAGCAATAACG  127
            ||||||||| ||||||||||||||||||||||||||||||||||||||||||||||||||
Sbjct  61   ATGGATAAGACTTTGGTCTGATTGTATAGGAGTTTTTGAACTAAAAAAGGAGCAATAACG  120

Query  128  CCCTCTTGATAAAACAAGAGGGAAGCTATTGCTCCttttttttATTTAGTACTATTTGCC  187
             |||||||||||||||||||||||||||||||||||||||||||||||||||||||||||
Sbjct  121  TCCTCTTGATAAAACAAGAGGGAAGCTATTGCTCCTTTTTTTTATTTAGTACTATTTGCC  180

Query  188  TTACATAGTTTCTTTAAAAATAACAAGTACTTTTTATAGTTTGGTTCGATTTGCGTGttt  247
            ||||||||||||||||||||      | |  |||||||||||||||||||||||||||||
Sbjct  181  TTACATAGTTTCTTTAAAAA------G-A--TTTTATAGTTTGGTTCGATTTGCGTGTTT  231

Query  248  tttctttgtattcatttatattataggtttctatatcatatattttcCCAATC  301
            |||||||||||||||||||||||||||||||||||||||||| |||||||||||
Sbjct  232  TTTCTTTGTATTCATTTATATTATAGGTTTCTATATCATATACTTTTCCCAATC  285


>OZ186474.1 Xanthium orientale genome assembly, organelle: plastid:chloroplast
Length=151592

 Score = 460 bits (249),  Expect = 4e-125
 Identities = 263/270 (97%), Gaps = 0/270 (0%)
 Strand=Plus/Minus

Query  2     TTATGCATGAACGTAATGCTCATAATTTCCCTCTAGACTTAGCTGCTATTGAAGCTCCAT  61
             ||||||||||||||||||||||||||||||||||||||||||||||||||||||||||||
Sbjct  1947  TTATGCATGAACGTAATGCTCATAATTTCCCTCTAGACTTAGCTGCTATTGAAGCTCCAT  1888

Query  62    CTACAAATGGATAAGCCTTTGGTCTGATTGTATAGGAGTTTTTGAACTAAAAAAGGAGCA  121
             ||||||||||||||| |||||||||||||||||| |||||||||||||||||||||||||
Sbjct  1887  CTACAAATGGATAAGACTTTGGTCTGATTGTATAAGAGTTTTTGAACTAAAAAAGGAGCA  1828

Query  122   ATAACGCCCTCTTGATAAAACAAGAGGGAAGCTATTGCTCCttttttttATTTAGTACTA  181
             ||||||||||||||||||||||||||||||||||||||||||||||||||||||||||||
Sbjct  1827  ATAACGCCCTCTTGATAAAACAAGAGGGAAGCTATTGCTCCTTTTTTTTATTTAGTACTA  1768

Query  182   TTTGCCTTACATAGTTTCTTTAAAAATAACAAGTACTTTTTATAGTTTGGTTCGATTTGC  241
             ||||||||||||||||||||||||||| | ||||||||||||||||||| ||  ||||||
Sbjct  1767  TTTGCCTTACATAGTTTCTTTAAAAATCAAAAGTACTTTTTATAGTTTGTTTTAATTTGC  1708

Query  242   GTGtttttctttgtattcatttatattat  271
             |||||||||||||||||||||||||||||
Sbjct  1707  GTGTTTTTCTTTGTATTCATTTATATTAT  1678


>MZ636529.1 Xanthium spinosum chloroplast, complete genome
Length=151512
```

```
 Score = 460 bits (249),  Expect = 4e-125
 Identities = 263/270 (97%), Gaps = 0/270 (0%)
 Strand=Plus/Minus

Query  2    TTATGCATGAACGTAATGCTCATAATTTCCCTCTAGACTTAGCTGCTATTGAAGCTCCAT  61
            ||||||||||||||||||||||||||||||||||||||||||||||||||||||||||||
Sbjct  552  TTATGCATGAACGTAATGCTCATAATTTCCCTCTAGACTTAGCTGCTATTGAAGCTCCAT  493

Query  62   CTACAAATGGATAAGCCTTTGGTCTGATTGTATAGGAGTTTTTGAACTAAAAAAGGAGCA  121
            ||||||||||||||| |||||||||||||||||| |||||||||||||||||||||||||
Sbjct  492  CTACAAATGGATAAGACTTTGGTCTGATTGTATAAGAGTTTTTGAACTAAAAAAGGAGCA  433

Query  122  ATAACGCCCTCTTGATAAAACAAGAGGGAAGCTATTGCTCCttttttttATTTAGTACTA  181
            ||||||||||||||||||||||||||||||||||||||||||||||||||||||||||||
Sbjct  432  ATAACGCCCTCTTGATAAAACAAGAGGGAAGCTATTGCTCCTTTTTTTTATTTAGTACTA  373

Query  182  TTTGCCTTACATAGTTTCTTTAAAAATAACAAGTACTTTTTATAGTTTGGTTCGATTTGC  241
            ||||||||||||||||||||||||||| | ||||||||||||||||||| ||  ||||||
Sbjct  372  TTTGCCTTACATAGTTTCTTTAAAAATCAAAAGTACTTTTTATAGTTTGTTTTAATTTGC  313

Query  242  GTGtttttctttgtattcatttatattat  271
            ||||||||||||||||||||||||||||||
Sbjct  312  GTGTTTTTCTTTGTATTCATTTATATTAT  283


>MK836182.1 Flaveria bidentis chloroplast, complete genome
Length=152230

 Score = 460 bits (249),  Expect = 4e-125
 Identities = 284/301 (94%), Gaps = 2/301 (1%)
 Strand=Plus/Minus

Query  2    TTATGCATGAACGTAATGCTCATAATTTCCCTCTAGACTTAGCTGCTATTGAAGCTCCAT  61
            ||||||||||||||||||||||||||||||||||||||||||||||||||||||||||||
Sbjct  600  TTATGCATGAACGTAATGCTCATAATTTCCCTCTAGACTTAGCTGCTATTGAAGCTCCAT  541

Query  62   CTACAAATGGATAAGCCTTTGGTCTGATTGTATAGGAGTTTTTGAACTAAAAAAGGAGCA  121
            ||||||||||||||| ||||||||||||||||||||||||||||||||||||||||||||
Sbjct  540  CTACAAATGGATAAGACTTTGGTCTGATTGTATAGGAGTTTTTGAACTAAAAAAGGAGCA  481

Query  122  ATAACG-CCCTCTTGATAAAACAAGAGGGAAGCTATTGCTCCttttttttATTTAGTACT  180
            ||| |  |||||||| |  | |||||| |  | |||||||||||||||||||||||||||
Sbjct  480  ATAGCTTCCCTCTTGTTTTATCAAGAGAGC-GTTATTGCTCCTTTTTTTTATTTAGTACT  422

Query  181  ATTTGCCTTACATAGTTTCTTTAAAAATAACAAGTACTTTTTATAGTTTGGTTCGATTTG  240
            |||||| |||||||||||||||||||||||||||| |||||||||||||||||||||||||
Sbjct  421  ATTTGCTTTACATAGTTTCTTTAAAAATAACAAGGACTTTTTATAGTTTGGTTCGATTTG  362

Query  241  CGTGttttttctttgtattcatttatattataggtttctatatcatatatttttcCCAAT  300
            |||||||| |||||||||||||||||||||||| ||||||||||||||| ||||||||||
Sbjct  361  CGTGTTTTGTCTTTGTATTCATTTATATTATAGCTTTCTATATCATATACTTTTCCCAAT  302

Query  301  C  301
            |
Sbjct  301  C  301
```

```
 Score = 104 bits (56),  Expect = 8e-18
 Identities = 58/59 (98%), Gaps = 0/59 (0%)
 Strand=Plus/Plus

Query  110  AAAAAAGGAGCAATAACGCCCTCTTGATAAAACAAGAGGGAAGCTATTGCTCCtttttt  168
            ||||||||||||||||||| |||||||||||||||||||||||||||||||||||||||
Sbjct  434  AAAAAAGGAGCAATAACGCTCTCTTGATAAAACAAGAGGGAAGCTATTGCTCCTTTTTT  492


>KX346911.1 Xanthium sibiricum voucher S-CEZi-03 PsbA (psbA) gene, partial
cds; and psbA-trnH intergenic spacer, partial sequence; chloroplast
Length=523

 Score = 460 bits (249),  Expect = 4e-125
 Identities = 263/270 (97%), Gaps = 0/270 (0%)
 Strand=Plus/Plus

Query  2    TTATGCATGAACGTAATGCTCATAATTTCCCTCTAGACTTAGCTGCTATTGAAGCTCCAT  61
            ||||||||||||||||||||||||||||||||||||||||||||||||||||||||||||
Sbjct  2    TTATGCATGAACGTAATGCTCATAATTTCCCTCTAGACTTAGCTGCTATTGAAGCTCCAT  61

Query  62   CTACAAATGGATAAGCCTTTGGTCTGATTGTATAGGAGTTTTTGAACTAAAAAAGGAGCA  121
            ||||||||||||||| |||||||||||||||||| |||||||||||||||||||||||||
Sbjct  62   CTACAAATGGATAAGACTTTGGTCTGATTGTATAAGAGTTTTTGAACTAAAAAAGGAGCA  121

Query  122  ATAACGCCCTCTTGATAAAACAAGAGGGAAGCTATTGCTCCttttttttATTTAGTACTA  181
            ||||||||||||||||||||||||||||||||||||||||||||||||||||||||||||
Sbjct  122  ATAACGCCCTCTTGATAAAACAAGAGGGAAGCTATTGCTCCTTTTTTTTATTTAGTACTA  181

Query  182  TTTGCCTTACATAGTTTCTTTAAAAATAACAAGTACTTTTTATAGTTTGGTTCGATTTGC  241
            ||||||||||||||||||||||||||| | ||||||||||||||||||| ||  ||||||
Sbjct  182  TTTGCCTTACATAGTTTCTTTAAAAATCAAAAGTACTTTTTATAGTTTGTTTTAATTTGC  241

Query  242  GTGtttttctttgtattcatttatattat  271
            |||||||||||||||||||||||||||||
Sbjct  242  GTGTTTTTCTTTGTATTCATTTATATTAT  271


>NC_081947.1 Ianthopappus corymbosus voucher TEX Panero & Crozier 8530 chloroplast,
complete genome
 OR069735.1 Ianthopappus corymbosus voucher TEX Panero & Crozier 8530 chloroplast,
complete genome
Length=152376

 Score = 460 bits (249),  Expect = 4e-125
 Identities = 284/300 (95%), Gaps = 5/300 (2%)
 Strand=Plus/Minus

Query  2    TTATGCATGAACGTAATGCTCATAATTTCCCTCTAGACTTAGCTGCTATTGAAGCTCCAT  61
            ||||||||||||||||||||||||||||||||||||||||||||||||| ||||||||||
Sbjct  543  TTATGCATGAACGTAATGCTCATAATTTCCCTCTAGACTTAGCTGCTATCGAAGCTCCAT  484

Query  62   CTACAAATGGATAAGCCTTTGGTCTGATTGTATAGGAGTTTTTGAACTAAAAAAGGAGCA  121
            ||||||||||||||| ||||||||||||||||||||||||||||||||||||||||||||
Sbjct  483  CTACAAATGGATAAGACTTTGGTCTGATTGTATAGGAGTTTTTGAACTAAAAAAGGAGCA  424

Query  122  ATAACGCCCTCTTGATAAAACAAGAGGGAAGCTATTGCTCCttttttttATTTAGTACTA  181
```

```
                |||||||||||||||||||||||||||||||||||||||||||||||||||||||||| ||
Sbjct  423   ATAACGCCCTCTTGATAAAACAAGAGGGAAGCTATTGCTCCTTTTTTTTATTTAGTAGTA  364

Query  182   TTTGCCTTACATAGTTTCTTTAAAAATAACAAGTACTTTTTATAGTTTGGTTCGATTTGC  241
             ||| |||||||||||||||||||||||||||||   ||||||||||||||||||||| ||
Sbjct  363   TTTTCCTTACATAGTTTCTTTAAAAATAACAAGGGGTTTTTATAGTTTGGTTCGATTAGC  304

Query  242   GTGttttttctttgtattcatttatattataggtttctatatcatatatttttcCCAATC  301
             ||||||| |||||||||| |||||||||||||||||  |||| || | |||| |||||||
Sbjct  303   GTGTTTTCTCTTTGTATTTATTTATATTATAGGTTT--ATAT-AT-TCTTTT-CCCAATC  249
```

>NC_053284.1 Aldama kunthiana voucher voucher Silva s.n. (ESA 122873) chloroplast, complete genome
 MN337895.1 Aldama kunthiana chloroplast, complete genome
Length=151327

 Score = 459 bits (248), Expect = 2e-124
 Identities = 284/300 (95%), Gaps = 7/300 (2%)
 Strand=Plus/Minus

```
Query  2     TTATGCATGAACGTAATGCTCATAATTTCCCTCTAGACTTAGCTGCTATTGAAGCTCCAT  61
             ||||||||||||||||||||||||||||||||||||||||||||||||||||||||||||
Sbjct  547   TTATGCATGAACGTAATGCTCATAATTTCCCTCTAGACTTAGCTGCTATTGAAGCTCCAT  488

Query  62    CTACAAATGGATAAGCCTTTGGTCTGATTGTATAGGAGTTTTTGAACTAAAAAAGGAGCA  121
             ||||||||||||||| ||||||||||||||||||||||||||||||||||||||||||||
Sbjct  487   CTACAAATGGATAAGACTTTGGTCTGATTGTATAGGAGTTTTTGAACTAAAAAAGGAGCA  428

Query  122   ATAACGCCCTCTTGATAAAACAAGAGGGAAGCTATTGCTCCttttttttATTTAGTACTA  181
             |||||||||||||| |  | ||||||||||||||||||||||||||||||||||||||||
Sbjct  427   ATAACGCCCTCTTGTTTTATCAAGAGGGAAGCTATTGCTCCTTTTTTTTATTTAGTACTA  368

Query  182   TTTGCCTTACATAGTTTCTTTAAA-------AATAACAAGTACTTTTTATAGTTTGGTTC  234
             ||||||||||||||||||||||||       |||||||||||||||||||||||||||||
Sbjct  367   TTTGCCTTACATAGTTTCTTTAAACTTTAAAAATAACAAGTACTTTTTATAGTTTGGTTC  308

Query  235   GATTTGCGTGttttttctttgtattcatttatattataggtttctatatcatatattttt  294
             ||||||  |||||||| |||||||||||||||||||||||||||||||||||||| ||||
Sbjct  307   GATTTGTTTGTTTTTTATTTGTATTCATTTATATTATAGGTTTCTATATCATATACTTTT  248
```

>NC_057179.1 Aldama tuberosa voucher Loeuille 719 chloroplast, complete genome
 MN356033.1 Aldama tuberosa chloroplast, complete genome
Length=151323

 Score = 459 bits (248), Expect = 2e-124
 Identities = 284/300 (95%), Gaps = 7/300 (2%)
 Strand=Plus/Minus

```
Query  2     TTATGCATGAACGTAATGCTCATAATTTCCCTCTAGACTTAGCTGCTATTGAAGCTCCAT  61
             ||||||||||||||||||||||||||||||||||||||||||||||||||||||||||||
Sbjct  549   TTATGCATGAACGTAATGCTCATAATTTCCCTCTAGACTTAGCTGCTATTGAAGCTCCAT  490

Query  62    CTACAAATGGATAAGCCTTTGGTCTGATTGTATAGGAGTTTTTGAACTAAAAAAGGAGCA  121
             ||||||||||||||| ||||||||||||||||||||||||||||||||||||||||||||
Sbjct  489   CTACAAATGGATAAGACTTTGGTCTGATTGTATAGGAGTTTTTGAACTAAAAAAGGAGCA  430
```

```
Query  122   ATAACGCCCTCTTGATAAAACAAGAGGGAAGCTATTGCTCCttttttttATTTAGTACTA  181
             |||||||||||||| |  | ||||||||||||||||||||||||||||||||||||||||
Sbjct  429   ATAACGCCCTCTTGTTTTATCAAGAGGGAAGCTATTGCTCCTTTTTTTTATTTAGTACTA  370

Query  182   TTTGCCTTACATAGTTTCTTTAAA-------AATAACAAGTACTTTTTATAGTTTGGTTC  234
             ||||||||||||||||||||||||       |||||||||||||||||||||||||||||
Sbjct  369   TTTGCCTTACATAGTTTCTTTAAACTTTAAAAATAACAAGTACTTTTTATAGTTTGGTTC  310

Query  235   GATTTGCGTGttttttctttgtattcatttatattataggtttctatatcatatattttt  294
             ||||||  |||||||| |||||||||||||||||||||||||||||||||||||| ||||
Sbjct  309   GATTTGTTTGTTTTTTATTTGTATTCATTTATATTATAGGTTTCTATATCATATACTTTT  250
```

>NC_057174.1 Aldama robusta voucher Bringel 985 chloroplast, complete genome
MN356028.1 Aldama robusta chloroplast, complete genome
Length=151343

Score = 459 bits (248), Expect = 2e-124
Identities = 284/300 (95%), Gaps = 7/300 (2%)
Strand=Plus/Minus

```
Query  2     TTATGCATGAACGTAATGCTCATAATTTCCCTCTAGACTTAGCTGCTATTGAAGCTCCAT  61
             ||||||||||||||||||||||||||||||||||||||||||||||||||||||||||||
Sbjct  553   TTATGCATGAACGTAATGCTCATAATTTCCCTCTAGACTTAGCTGCTATTGAAGCTCCAT  494

Query  62    CTACAAATGGATAAGCCTTTGGTCTGATTGTATAGGAGTTTTTGAACTAAAAAAGGAGCA  121
             ||||||||||||||| ||||||||||||||||||||||||||||||||||||||||||||
Sbjct  493   CTACAAATGGATAAGACTTTGGTCTGATTGTATAGGAGTTTTTGAACTAAAAAAGGAGCA  434

Query  122   ATAACGCCCTCTTGATAAAACAAGAGGGAAGCTATTGCTCCttttttttATTTAGTACTA  181
             |||||||||||||| |  | ||||||||||||||||||||||||||||||||||||||||
Sbjct  433   ATAACGCCCTCTTGTTTTATCAAGAGGGAAGCTATTGCTCCTTTTTTTTATTTAGTACTA  374

Query  182   TTTGCCTTACATAGTTTCTTTAAA-------AATAACAAGTACTTTTTATAGTTTGGTTC  234
             ||||||||||||||||||||||||       |||||||||||||||||||||||||||||
Sbjct  373   TTTGCCTTACATAGTTTCTTTAAACTTTAAAAATAACAAGTACTTTTTATAGTTTGGTTC  314

Query  235   GATTTGCGTGttttttctttgtattcatttatattataggtttctatatcatatattttt  294
             ||||||  |||||||| |||||||||||||||||||||||||||||||||||||| ||||
Sbjct  313   GATTTGTTTGTTTTTTATTTGTATTCATTTATATTATAGGTTTCTATATCATATACTTTT  254
```

>LS999859.1 Echinacea angustifolia DNA containing psbA-trnH IGS, specimen voucher FEM_DBE_015
Length=437

Score = 459 bits (248), Expect = 2e-124
Identities = 251/252 (99%), Gaps = 1/252 (0%)
Strand=Plus/Plus

```
Query  50    TTGAAGCTCCATCTACAAATGGATAAGCCTTTGGTCTGATTGTATAGGAGTTTTTGAACT  109
             ||||||||||||||||||||||||||||||||||||||||||||||||||||||||||||
Sbjct  1     TTGAAGCTCCATCTACAAATGGATAAGCCTTTGGTCTGATTGTATAGGAGTTTTTGAACT  60

Query  110   AAAAAAGGAGCAATAACGCCCTCTTGATAAAACAAGAGGGAAGCTATTGCTCCttttttt  169
             ||||||||||||||||||||||||||||||||||||||||||||||||||||| ||||||
```

```
Sbjct  61   AAAAAAGGAGCAATAACGCCCTCTTGATAAAACAAGAGGGAAGCTATTGCTCC-TTTTTT  119

Query  170  tATTTAGTACTATTTGCCTTACATAGTTTCTTTAAAAATAACAAGTACTTTTTATAGTTT  229
            ||||||||||||||||||||||||||||||||||||||||||||||||||||||||||||
Sbjct  120  TATTTAGTACTATTTGCCTTACATAGTTTCTTTAAAAATAACAAGTACTTTTTATAGTTT  179

Query  230  GGTTCGATTTGCGTGtttttctttgtattcatttatattataggtttctatatcatata  289
            ||||||||||||||||||||||||||||||||||||||||||||||||||||||||||||
Sbjct  180  GGTTCGATTTGCGTGTTTTTTCTTTGTATTCATTTATATTATAGGTTTCTATATCATATA  239

Query  290  tttttcCCAATC  301
            ||||||||||||
Sbjct  240  TTTTTCCCAATC  251
```

>NC_053296.1 Aldama canescens voucher Schilling 17 chloroplast, complete genome
 MN337908.1 Aldama canescens chloroplast, complete genome
Length=151386

 Score = 459 bits (248), Expect = 2e-124
 Identities = 284/300 (95%), Gaps = 7/300 (2%)
 Strand=Plus/Minus

```
Query  2    TTATGCATGAACGTAATGCTCATAATTTCCCTCTAGACTTAGCTGCTATTGAAGCTCCAT  61
            ||||||||||||||||||||||||||||||||||||||||||||||||||||||||||||
Sbjct  545  TTATGCATGAACGTAATGCTCATAATTTCCCTCTAGACTTAGCTGCTATTGAAGCTCCAT  486

Query  62   CTACAAATGGATAAGCCTTTGGTCTGATTGTATAGGAGTTTTTGAACTAAAAAAGGAGCA  121
            ||||||||||||||| ||||||||||||||||||||||||||||||||||||||||||||
Sbjct  485  CTACAAATGGATAAGACTTTGGTCTGATTGTATAGGAGTTTTTGAACTAAAAAAGGAGCA  426

Query  122  ATAACGCCCTCTTGATAAAACAAGAGGGAAGCTATTGCTCCtttttttATTTAGTACTA  181
            |||||||||||||| |  | ||||||||||||||||||||||||||||||||||||||||
Sbjct  425  ATAACGCCCTCTTGTTTTATCAAGAGGGAAGCTATTGCTCCTTTTTTTTATTTAGTACTA  366

Query  182  TTTGCCTTACATAGTTTCTTTAAA-------AATAACAAGTACTTTTTATAGTTTGGTTC  234
            ||||||||||||||||||||||||       |||||||||||||||||||||||||||||
Sbjct  365  TTTGCCTTACATAGTTTCTTTAAACTTTAAAAATAACAAGTACTTTTTATAGTTTGGTTC  306

Query  235  GATTTGCGTGttttttctttgtattcatttatattataggtttctatatcatatattttt  294
            ||||||  |||||||| ||||||||||||||||||||||||||||||||||||||| ||||
Sbjct  305  GATTTGTTTGTTTTTTATTTGTATTCATTTATATTATAGGTTTCTATATCATATACTTTT  246
```

>NC_053279.1 Aldama filifolia voucher Loeuille 849 chloroplast, complete genome
 MN337890.1 Aldama filifolia chloroplast, complete genome
Length=151333

 Score = 459 bits (248), Expect = 2e-124
 Identities = 284/300 (95%), Gaps = 7/300 (2%)
 Strand=Plus/Minus

```
Query  2    TTATGCATGAACGTAATGCTCATAATTTCCCTCTAGACTTAGCTGCTATTGAAGCTCCAT  61
            ||||||||||||||||||||||||||||||||||||||||||||||||||||||||||||
Sbjct  553  TTATGCATGAACGTAATGCTCATAATTTCCCTCTAGACTTAGCTGCTATTGAAGCTCCAT  494

Query  62   CTACAAATGGATAAGCCTTTGGTCTGATTGTATAGGAGTTTTTGAACTAAAAAAGGAGCA  121
```

```
                |||||||||||||||| ||||||||||||||||||||||||||||||||||||||||||||
Sbjct  493   CTACAAATGGATAAGACTTTGGTCTGATTGTATAGGAGTTTTTGAACTAAAAAAGGAGCA  434

Query  122   ATAACGCCCTCTTGATAAAACAAGAGGGAAGCTATTGCTCCttttttttATTTAGTACTA  181
             |||||||||||||| |  | ||||||||||||||||||||||||||||||||||||||||
Sbjct  433   ATAACGCCCTCTTGTTTTATCAAGAGGGAAGCTATTGCTCCTTTTTTTTATTTAGTACTA  374

Query  182   TTTGCCTTACATAGTTTCTTTAAA-------AATAACAAGTACTTTTTATAGTTTGGTTC  234
             ||||||||||||||||||||||||       |||||||||||||||||||||||||||||
Sbjct  373   TTTGCCTTACATAGTTTCTTTAAACTTTAAAAATAACAAGTACTTTTTATAGTTTGGTTC  314

Query  235   GATTTGCGTGttttttctttgtattcatttatattataggtttctatatcatatattttt  294
             ||||||  |||||||| |||||||||||||||||||||||||||||||||||||| ||||
Sbjct  313   GATTTGTTTGTTTTTTATTTGTATTCATTTATATTATAGGTTTCTATATCATATACTTTT  254


>NC_053285.1 Aldama linearis voucher Schilling 70 chloroplast, complete genome
 MN337896.1 Aldama linearis chloroplast, complete genome
Length=151314

 Score = 459 bits (248),  Expect = 2e-124
 Identities = 284/300 (95%), Gaps = 7/300 (2%)
 Strand=Plus/Minus

Query  2     TTATGCATGAACGTAATGCTCATAATTTCCCTCTAGACTTAGCTGCTATTGAAGCTCCAT  61
             ||||||||||||||||||||||||||||||||||||||||||||||||||||||||||||
Sbjct  546   TTATGCATGAACGTAATGCTCATAATTTCCCTCTAGACTTAGCTGCTATTGAAGCTCCAT  487

Query  62    CTACAAATGGATAAGCCTTTGGTCTGATTGTATAGGAGTTTTTGAACTAAAAAAGGAGCA  121
             ||||||||||||||| ||||||||||||||||||||||||||||||||||||||||||||
Sbjct  486   CTACAAATGGATAAGACTTTGGTCTGATTGTATAGGAGTTTTTGAACTAAAAAAGGAGCA  427

Query  122   ATAACGCCCTCTTGATAAAACAAGAGGGAAGCTATTGCTCCttttttttATTTAGTACTA  181
             |||||||||||||| |  | ||||||||||||||||||||||||||||||||||||||||
Sbjct  426   ATAACGCCCTCTTGTTTTATCAAGAGGGAAGCTATTGCTCCTTTTTTTTATTTAGTACTA  367

Query  182   TTTGCCTTACATAGTTTCTTTAAA-------AATAACAAGTACTTTTTATAGTTTGGTTC  234
             ||||||||||||||||||||||||       |||||||||||||||||||||||||||||
Sbjct  366   TTTGCCTTACATAGTTTCTTTAAACTTTAAAAATAACAAGTACTTTTTATAGTTTGGTTC  307

Query  235   GATTTGCGTGttttttctttgtattcatttatattataggtttctatatcatatattttt  294
             ||||||  |||||||| |||||||||||||||||||||||||||||||||||||| ||||
Sbjct  306   GATTTGTTTGTTTTTTATTTGTATTCATTTATATTATAGGTTTCTATATCATATACTTTT  247


>NC_057182.1 Aldama vernonioides voucher Magenta 460 chloroplast, complete
genome
 MN356036.1 Aldama vernonioides chloroplast, complete genome
Length=151221

 Score = 459 bits (248),  Expect = 2e-124
 Identities = 284/300 (95%), Gaps = 7/300 (2%)
 Strand=Plus/Minus

Query  2     TTATGCATGAACGTAATGCTCATAATTTCCCTCTAGACTTAGCTGCTATTGAAGCTCCAT  61
             ||||||||||||||||||||||||||||||||||||||||||||||||||||||||||||
Sbjct  548   TTATGCATGAACGTAATGCTCATAATTTCCCTCTAGACTTAGCTGCTATTGAAGCTCCAT  489
```

```
Query  62    CTACAAATGGATAAGCCTTTGGTCTGATTGTATAGGAGTTTTTGAACTAAAAAAGGAGCA  121
             |||||||||||||| |||||||||||||||||||||||||||||||||||||||||||||
Sbjct  488   CTACAAATGGATAAGACTTTGGTCTGATTGTATAGGAGTTTTTGAACTAAAAAAGGAGCA  429

Query  122   ATAACGCCCTCTTGATAAAACAAGAGGGAAGCTATTGCTCCtttttttATTTAGTACTA  181
             |||||||||||||| |  | ||||||||||||||||||||||||||||||||||||||||
Sbjct  428   ATAACGCCCTCTTGTTTTATCAAGAGGGAAGCTATTGCTCCTTTTTTTTATTTAGTACTA  369

Query  182   TTTGCCTTACATAGTTTCTTTAAA-------AATAACAAGTACTTTTTATAGTTTGGTTC  234
             ||||||||||||||||||||||||       |||||||||||||||||||||||||||||
Sbjct  368   TTTGCCTTACATAGTTTCTTTAAACTTTAAAAATAACAAGTACTTTTTATAGTTTGGTTC  309

Query  235   GATTTGCGTGttttttctttgtattcatttatattataggtttctatatcatatattttt  294
             ||||||  |||||||| |||||||||||||||||||||||||||||||||||||| ||||
Sbjct  308   GATTTGTTTGTTTTTTATTTGTATTCATTTATATTATAGGTTTCTATATCATATACTTTT  249


>NC_057180.1 Aldama tukumanensis voucher Heiden 1837 chloroplast, complete
genome
 MN356034.1 Aldama tukumanensis chloroplast, complete genome
Length=151336

 Score = 459 bits (248),  Expect = 2e-124
 Identities = 284/300 (95%), Gaps = 7/300 (2%)
 Strand=Plus/Minus

Query  2     TTATGCATGAACGTAATGCTCATAATTTCCCTCTAGACTTAGCTGCTATTGAAGCTCCAT  61
             ||||||||||||||||||||||||||||||||||||||||||||||||||||||||||||
Sbjct  548   TTATGCATGAACGTAATGCTCATAATTTCCCTCTAGACTTAGCTGCTATTGAAGCTCCAT  489

Query  62    CTACAAATGGATAAGCCTTTGGTCTGATTGTATAGGAGTTTTTGAACTAAAAAAGGAGCA  121
             |||||||||||||| |||||||||||||||||||||||||||||||||||||||||||||
Sbjct  488   CTACAAATGGATAAGACTTTGGTCTGATTGTATAGGAGTTTTTGAACTAAAAAAGGAGCA  429

Query  122   ATAACGCCCTCTTGATAAAACAAGAGGGAAGCTATTGCTCCtttttttATTTAGTACTA  181
             |||||||||||||| |  | ||||||||||||||||||||||||||||||||||||||||
Sbjct  428   ATAACGCCCTCTTGTTTTATCAAGAGGGAAGCTATTGCTCCTTTTTTTTATTTAGTACTA  369

Query  182   TTTGCCTTACATAGTTTCTTTAAA-------AATAACAAGTACTTTTTATAGTTTGGTTC  234
             ||||||||||||||||||||||||       |||||||||||||||||||||||||||||
Sbjct  368   TTTGCCTTACATAGTTTCTTTAAACTTTAAAAATAACAAGTACTTTTTATAGTTTGGTTC  309

Query  235   GATTTGCGTGttttttctttgtattcatttatattataggtttctatatcatatattttt  294
             ||||||  |||||||| |||||||||||||||||||||||||||||||||||||| ||||
Sbjct  308   GATTTGTTTGTTTTTTATTTGTATTCATTTATATTATAGGTTTCTATATCATATACTTTT  249


>NC_053295.1 Aldama bracteata voucher Filartiga 15 chloroplast, complete genome
 MN337907.1 Aldama bracteata chloroplast, complete genome
Length=151339

 Score = 459 bits (248),  Expect = 2e-124
 Identities = 284/300 (95%), Gaps = 7/300 (2%)
 Strand=Plus/Minus

Query  2     TTATGCATGAACGTAATGCTCATAATTTCCCTCTAGACTTAGCTGCTATTGAAGCTCCAT  61
```

```
            ||||||||||||||||||||||||||||||||||||||||||||||||||||||||||||
Sbjct  545  TTATGCATGAACGTAATGCTCATAATTTCCCTCTAGACTTAGCTGCTATTGAAGCTCCAT  486

Query  62   CTACAAATGGATAAGCCTTTGGTCTGATTGTATAGGAGTTTTTGAACTAAAAAAGGAGCA  121
            ||||||||||||||| ||||||||||||||||||||||||||||||||||||||||||||
Sbjct  485  CTACAAATGGATAAGACTTTGGTCTGATTGTATAGGAGTTTTTGAACTAAAAAAGGAGCA  426

Query  122  ATAACGCCCTCTTGATAAAACAAGAGGGAAGCTATTGCTCCttttttttATTTAGTACTA  181
            |||||||||||||| |  | ||||||||||||||||||||||||||||||||||||||||
Sbjct  425  ATAACGCCCTCTTGTTTTATCAAGAGGGAAGCTATTGCTCCTTTTTTTTATTTAGTACTA  366

Query  182  TTTGCCTTACATAGTTTCTTTAAA-------AATAACAAGTACTTTTTATAGTTTGGTTC  234
            ||||||||||||||||||||||||       |||||||||||||||||||||||||||||
Sbjct  365  TTTGCCTTACATAGTTTCTTTAAACTTTAAAAATAACAAGTACTTTTTATAGTTTGGTTC  306

Query  235  GATTTGCGTGttttttctttgtattcatttatattataggtttctatatcatatattttt  294
            ||||||  |||||||| ||||||||||||||||||||||||||||||||||||||| ||||
Sbjct  305  GATTTGTTTGTTTTTTATTTGTATTCATTTATATTATAGGTTTCTATATCATATACTTTT  246


>NC_057175.1 Aldama rubra voucher Magenta 388 chloroplast, complete genome
 MN356029.1 Aldama rubra chloroplast, complete genome
Length=151322

 Score = 459 bits (248),  Expect = 2e-124
 Identities = 284/300 (95%), Gaps = 7/300 (2%)
 Strand=Plus/Minus

Query  2    TTATGCATGAACGTAATGCTCATAATTTCCCTCTAGACTTAGCTGCTATTGAAGCTCCAT  61
            ||||||||||||||||||||||||||||||||||||||||||||||||||||||||||||
Sbjct  548  TTATGCATGAACGTAATGCTCATAATTTCCCTCTAGACTTAGCTGCTATTGAAGCTCCAT  489

Query  62   CTACAAATGGATAAGCCTTTGGTCTGATTGTATAGGAGTTTTTGAACTAAAAAAGGAGCA  121
            ||||||||||||||| ||||||||||||||||||||||||||||||||||||||||||||
Sbjct  488  CTACAAATGGATAAGACTTTGGTCTGATTGTATAGGAGTTTTTGAACTAAAAAAGGAGCA  429

Query  122  ATAACGCCCTCTTGATAAAACAAGAGGGAAGCTATTGCTCCttttttttATTTAGTACTA  181
            |||||||||||||| |  | ||||||||||||||||||||||||||||||||||||||||
Sbjct  428  ATAACGCCCTCTTGTTTTATCAAGAGGGAAGCTATTGCTCCTTTTTTTTATTTAGTACTA  369

Query  182  TTTGCCTTACATAGTTTCTTTAAA-------AATAACAAGTACTTTTTATAGTTTGGTTC  234
            ||||||||||||||||||||||||       |||||||||||||||||||||||||||||
Sbjct  368  TTTGCCTTACATAGTTTCTTTAAACTTTAAAAATAACAAGTACTTTTTATAGTTTGGTTC  309

Query  235  GATTTGCGTGttttttctttgtattcatttatattataggtttctatatcatatattttt  294
            ||||||  |||||||| ||||||||||||||||||||||||||||||||||||||| ||||
Sbjct  308  GATTTGTTTGTTTTTTATTTGTATTCATTTATATTATAGGTTTCTATATCATATACTTTT  249


>KX346910.1 Xanthium sibiricum voucher S-CEZi-02 PsbA (psbA) gene, partial
cds; and psbA-trnH intergenic spacer, partial sequence; chloroplast
Length=521

 Score = 459 bits (248),  Expect = 2e-124
 Identities = 262/269 (97%), Gaps = 0/269 (0%)
 Strand=Plus/Plus
```

```
Query  3    TATGCATGAACGTAATGCTCATAATTTCCCTCTAGACTTAGCTGCTATTGAAGCTCCATC  62
            ||||||||||||||||||||||||||||||||||||||||||||||||||||||||||||
Sbjct  1    TATGCATGAACGTAATGCTCATAATTTCCCTCTAGACTTAGCTGCTATTGAAGCTCCATC  60

Query  63   TACAAATGGATAAGCCTTTGGTCTGATTGTATAGGAGTTTTTGAACTAAAAAAGGAGCAA  122
            |||||||||||||| |||||||||||||||||| ||||||||||||||||||||||||||
Sbjct  61   TACAAATGGATAAGACTTTGGTCTGATTGTATAAGAGTTTTTGAACTAAAAAAGGAGCAA  120

Query  123  TAACGCCCTCTTGATAAAACAAGAGGGAAGCTATTGCTCCttttttttATTTAGTACTAT  182
            ||||||||||||||||||||||||||||||||||||||||||||||||||||||||||||
Sbjct  121  TAACGCCCTCTTGATAAAACAAGAGGGAAGCTATTGCTCCTTTTTTTTATTTAGTACTAT  180

Query  183  TTGCCTTACATAGTTTCTTTAAAAATAACAAGTACTTTTTATAGTTTGGTTCGATTTGCG  242
            |||||||||||||||||||||||||| | ||||||||||||||||||| ||  |||||||
Sbjct  181  TTGCCTTACATAGTTTCTTTAAAAATCAAAAGTACTTTTTATAGTTTGTTTTAATTTGCG  240

Query  243  TGttttttctttgtattcatttatattat  271
            |||||||||||||||||||||||||||||
Sbjct  241  TGTTTTTTCTTTGTATTCATTTATATTAT  269
```

>NC_053282.1 Aldama goyazii voucher Magenta 716 chloroplast, complete genome
 MN337893.1 Aldama goyazii chloroplast, complete genome
Length=151279

 Score = 459 bits (248), Expect = 2e-124
 Identities = 284/300 (95%), Gaps = 7/300 (2%)
 Strand=Plus/Minus

```
Query  2    TTATGCATGAACGTAATGCTCATAATTTCCCTCTAGACTTAGCTGCTATTGAAGCTCCAT  61
            ||||||||||||||||||||||||||||||||||||||||||||||||||||||||||||
Sbjct  547  TTATGCATGAACGTAATGCTCATAATTTCCCTCTAGACTTAGCTGCTATTGAAGCTCCAT  488

Query  62   CTACAAATGGATAAGCCTTTGGTCTGATTGTATAGGAGTTTTTGAACTAAAAAAGGAGCA  121
            ||||||||||||||| ||||||||||||||||||||||||||||||||||||||||||||
Sbjct  487  CTACAAATGGATAAGACTTTGGTCTGATTGTATAGGAGTTTTTGAACTAAAAAAGGAGCA  428

Query  122  ATAACGCCCTCTTGATAAAACAAGAGGGAAGCTATTGCTCCttttttttATTTAGTACTA  181
            |||||||||||||| |  | ||||||||||||||||||||||||||||||||||||||||
Sbjct  427  ATAACGCCCTCTTGTTTTATCAAGAGGGAAGCTATTGCTCCTTTTTTTTATTTAGTACTA  368

Query  182  TTTGCCTTACATAGTTTCTTTAAA-------AATAACAAGTACTTTTTATAGTTTGGTTC  234
            ||||||||||||||||||||||||       |||||||||||||||||||||||||||||
Sbjct  367  TTTGCCTTACATAGTTTCTTTAAACTTTAAAAATAACAAGTACTTTTTATAGTTTGGTTC  308

Query  235  GATTTGCGTGttttttctttgtattcatttatattataggtttctatatcatatattttt  294
            ||||||  |||||||| |||||||||||||||||||||||||||||||||||||| ||||
Sbjct  307  GATTTGTTTGTTTTTTATTTGTATTCATTTATATTATAGGTTTCTATATCATATACTTTT  248
```

>NC_053286.1 Aldama macrorhiza voucher Magenta 476 chloroplast, complete genome
 MN337897.1 Aldama macrorhiza voucher Magenta 476 chloroplast, complete genome
Length=151314

 Score = 459 bits (248), Expect = 2e-124
 Identities = 284/300 (95%), Gaps = 7/300 (2%)
 Strand=Plus/Minus

```
Query  2    TTATGCATGAACGTAATGCTCATAATTTCCCTCTAGACTTAGCTGCTATTGAAGCTCCAT  61
            ||||||||||||||||||||||||||||||||||||||||||||||||||||||||||||
Sbjct  547  TTATGCATGAACGTAATGCTCATAATTTCCCTCTAGACTTAGCTGCTATTGAAGCTCCAT  488

Query  62   CTACAAATGGATAAGCCTTTGGTCTGATTGTATAGGAGTTTTTGAACTAAAAAAGGAGCA  121
            ||||||||||||||| ||||||||||||||||||||||||||||||||||||||||||||
Sbjct  487  CTACAAATGGATAAGACTTTGGTCTGATTGTATAGGAGTTTTTGAACTAAAAAAGGAGCA  428

Query  122  ATAACGCCCTCTTGATAAAACAAGAGGGAAGCTATTGCTCCtttttttATTTAGTACTA  181
            |||||||||||||| |  | ||||||||||||||||||||||||||||||||||||||||
Sbjct  427  ATAACGCCCTCTTGTTTTATCAAGAGGGAAGCTATTGCTCCTTTTTTTTATTTAGTACTA  368

Query  182  TTTGCCTTACATAGTTTCTTTAAA-------AATAACAAGTACTTTTTATAGTTTGGTTC  234
            ||||||||||||||||||||||||       |||||||||||||||||||||||||||||
Sbjct  367  TTTGCCTTACATAGTTTCTTTAAACTTTAAAAATAACAAGTACTTTTTATAGTTTGGTTC  308

Query  235  GATTTGCGTGttttttctttgtattcatttatattataggtttctatatcatatattttt  294
            ||||||  |||||||| |||||||||||||||||||||||||||||||||||||| ||||
Sbjct  307  GATTTGTTTGTTTTTTATTTGTATTCATTTATATTATAGGTTTCTATATCATATACTTTT  248


>NC_057173.1 Aldama revoluta voucher Loeuille 799 chloroplast, complete genome
 MN356027.1 Aldama revoluta chloroplast, complete genome
Length=151376

 Score = 459 bits (248),  Expect = 2e-124
 Identities = 284/300 (95%), Gaps = 7/300 (2%)
 Strand=Plus/Minus

Query  2    TTATGCATGAACGTAATGCTCATAATTTCCCTCTAGACTTAGCTGCTATTGAAGCTCCAT  61
            ||||||||||||||||||||||||||||||||||||||||||||||||||||||||||||
Sbjct  552  TTATGCATGAACGTAATGCTCATAATTTCCCTCTAGACTTAGCTGCTATTGAAGCTCCAT  493

Query  62   CTACAAATGGATAAGCCTTTGGTCTGATTGTATAGGAGTTTTTGAACTAAAAAAGGAGCA  121
            ||||||||||||||| ||||||||||||||||||||||||||||||||||||||||||||
Sbjct  492  CTACAAATGGATAAGACTTTGGTCTGATTGTATAGGAGTTTTTGAACTAAAAAAGGAGCA  433

Query  122  ATAACGCCCTCTTGATAAAACAAGAGGGAAGCTATTGCTCCtttttttATTTAGTACTA  181
            |||||||||||||| |  | ||||||||||||||||||||||||||||||||||||||||
Sbjct  432  ATAACGCCCTCTTGTTTTATCAAGAGGGAAGCTATTGCTCCTTTTTTTTATTTAGTACTA  373

Query  182  TTTGCCTTACATAGTTTCTTTAAA-------AATAACAAGTACTTTTTATAGTTTGGTTC  234
            ||||||||||||||||||||||||       |||||||||||||||||||||||||||||
Sbjct  372  TTTGCCTTACATAGTTTCTTTAAACTTTAAAAATAACAAGTACTTTTTATAGTTTGGTTC  313

Query  235  GATTTGCGTGttttttctttgtattcatttatattataggtttctatatcatatattttt  294
            ||||||  |||||||| |||||||||||||||||||||||||||||||||||||| ||||
Sbjct  312  GATTTGTTTGTTTTTTATTTGTATTCATTTATATTATAGGTTTCTATATCATATACTTTT  253


>NC_053281.1 Aldama gardneri voucher Filartiga 16 chloroplast, complete genome
 MN337892.1 Aldama gardneri chloroplast, complete genome
Length=151312

 Score = 459 bits (248),  Expect = 2e-124
 Identities = 284/300 (95%), Gaps = 7/300 (2%)
```

```
 Strand=Plus/Minus

Query  2    TTATGCATGAACGTAATGCTCATAATTTCCCTCTAGACTTAGCTGCTATTGAAGCTCCAT  61
            ||||||||||||||||||||||||||||||||||||||||||||||||||||||||||||
Sbjct  554  TTATGCATGAACGTAATGCTCATAATTTCCCTCTAGACTTAGCTGCTATTGAAGCTCCAT  495

Query  62   CTACAAATGGATAAGCCTTTGGTCTGATTGTATAGGAGTTTTTGAACTAAAAAAGGAGCA  121
            ||||||||||||||| ||||||||||||||||||||||||||||||||||||||||||||
Sbjct  494  CTACAAATGGATAAGACTTTGGTCTGATTGTATAGGAGTTTTTGAACTAAAAAAGGAGCA  435

Query  122  ATAACGCCCTCTTGATAAAACAAGAGGGAAGCTATTGCTCCttttttttATTTAGTACTA  181
            |||||||||||||| |  | ||||||||||||||||||||||||||||||||||||||||
Sbjct  434  ATAACGCCCTCTTGTTTTATCAAGAGGGAAGCTATTGCTCCTTTTTTTTATTTAGTACTA  375

Query  182  TTTGCCTTACATAGTTTCTTTAAA-------AATAACAAGTACTTTTTATAGTTTGGTTC  234
            ||||||||||||||||||||||||       |||||||||||||||||||||||||||||
Sbjct  374  TTTGCCTTACATAGTTTCTTTAAACTTTAAAAATAACAAGTACTTTTTATAGTTTGGTTC  315

Query  235  GATTTGCGTGttttttctttgtattcatttatattataggtttctatatcatatattttt  294
            ||||||  |||||||| |||||||||||||||||||||||||||||||||||||| ||||
Sbjct  314  GATTTGTTTGTTTTTTATTTGTATTCATTTATATTATAGGTTTCTATATCATATACTTTT  255


>NC_053288.1 Aldama nudibasilaris voucher Filartiga 1 chloroplast, complete
genome
 MN337899.1 Aldama nudibasilaris chloroplast, complete genome
Length=151316

 Score = 459 bits (248),  Expect = 2e-124
 Identities = 284/300 (95%), Gaps = 7/300 (2%)
 Strand=Plus/Minus

Query  2    TTATGCATGAACGTAATGCTCATAATTTCCCTCTAGACTTAGCTGCTATTGAAGCTCCAT  61
            ||||||||||||||||||||||||||||||||||||||||||||||||||||||||||||
Sbjct  548  TTATGCATGAACGTAATGCTCATAATTTCCCTCTAGACTTAGCTGCTATTGAAGCTCCAT  489

Query  62   CTACAAATGGATAAGCCTTTGGTCTGATTGTATAGGAGTTTTTGAACTAAAAAAGGAGCA  121
            ||||||||||||||| ||||||||||||||||||||||||||||||||||||||||||||
Sbjct  488  CTACAAATGGATAAGACTTTGGTCTGATTGTATAGGAGTTTTTGAACTAAAAAAGGAGCA  429

Query  122  ATAACGCCCTCTTGATAAAACAAGAGGGAAGCTATTGCTCCttttttttATTTAGTACTA  181
            |||||||||||||| |  | ||||||||||||||||||||||||||||||||||||||||
Sbjct  428  ATAACGCCCTCTTGTTTTATCAAGAGGGAAGCTATTGCTCCTTTTTTTTATTTAGTACTA  369

Query  182  TTTGCCTTACATAGTTTCTTTAAA-------AATAACAAGTACTTTTTATAGTTTGGTTC  234
            ||||||||||||||||||||||||       |||||||||||||||||||||||||||||
Sbjct  368  TTTGCCTTACATAGTTTCTTTAAACTTTAAAAATAACAAGTACTTTTTATAGTTTGGTTC  309

Query  235  GATTTGCGTGttttttctttgtattcatttatattataggtttctatatcatatattttt  294
            ||||||  |||||||| |||||||||||||||||||||||||||||||||||||| ||||
Sbjct  308  GATTTGTTTGTTTTTTATTTGTATTCATTTATATTATAGGTTTCTATATCATATACTTTT  249


>NC_053298.1 Aldama trichophylla voucher Magenta 390 chloroplast, complete
genome
 MN337911.1 Aldama trichophylla chloroplast, complete genome
Length=151353
```

```
 Score = 459 bits (248),  Expect = 2e-124
 Identities = 284/300 (95%), Gaps = 7/300 (2%)
 Strand=Plus/Minus

Query  2    TTATGCATGAACGTAATGCTCATAATTTCCCTCTAGACTTAGCTGCTATTGAAGCTCCAT  61
            ||||||||||||||||||||||||||||||||||||||||||||||||||||||||||||
Sbjct  548  TTATGCATGAACGTAATGCTCATAATTTCCCTCTAGACTTAGCTGCTATTGAAGCTCCAT  489

Query  62   CTACAAATGGATAAGCCTTTGGTCTGATTGTATAGGAGTTTTTGAACTAAAAAAGGAGCA  121
            ||||||||||||||| ||||||||||||||||||||||||||||||||||||||||||||
Sbjct  488  CTACAAATGGATAAGACTTTGGTCTGATTGTATAGGAGTTTTTGAACTAAAAAAGGAGCA  429

Query  122  ATAACGCCCTCTTGATAAAACAAGAGGGAAGCTATTGCTCCttttttttATTTAGTACTA  181
            |||||||||||||| |  | ||||||||||||||||||||||||||||||||||||||||
Sbjct  428  ATAACGCCCTCTTGTTTTATCAAGAGGGAAGCTATTGCTCCTTTTTTTTATTTAGTACTA  369

Query  182  TTTGCCTTACATAGTTTCTTTAAA-------AATAACAAGTACTTTTTATAGTTTGGTTC  234
            ||||||||||||||||||||||||       |||||||||||||||||||||||||||||
Sbjct  368  TTTGCCTTACATAGTTTCTTTAAACTTTAAAAATAACAAGTACTTTTTATAGTTTGGTTC  309

Query  235  GATTTGCGTGttttttctttgtattcatttatattataggtttctatatcatatattttt  294
            ||||||  |||||||| |||||||||||||||||||||||||||||||||||||| ||||
Sbjct  308  GATTTGTTTGTTTTTTATTTGTATTCATTTATATTATAGGTTTCTATATCATATACTTTT  249


>NC_053293.1 Aldama aspilioides voucher Filartiga 18 chloroplast, complete
genome
 MN337905.1 Viguiera aspilioides voucher Filartiga 18 chloroplast, complete
genome
Length=151362

 Score = 459 bits (248),  Expect = 2e-124
 Identities = 284/300 (95%), Gaps = 7/300 (2%)
 Strand=Plus/Minus

Query  2    TTATGCATGAACGTAATGCTCATAATTTCCCTCTAGACTTAGCTGCTATTGAAGCTCCAT  61
            ||||||||||||||||||||||||||||||||||||||||||||||||||||||||||||
Sbjct  545  TTATGCATGAACGTAATGCTCATAATTTCCCTCTAGACTTAGCTGCTATTGAAGCTCCAT  486

Query  62   CTACAAATGGATAAGCCTTTGGTCTGATTGTATAGGAGTTTTTGAACTAAAAAAGGAGCA  121
            ||||||||||||||| ||||||||||||||||||||||||||||||||||||||||||||
Sbjct  485  CTACAAATGGATAAGACTTTGGTCTGATTGTATAGGAGTTTTTGAACTAAAAAAGGAGCA  426

Query  122  ATAACGCCCTCTTGATAAAACAAGAGGGAAGCTATTGCTCCttttttttATTTAGTACTA  181
            |||||||||||||| |  | ||||||||||||||||||||||||||||||||||||||||
Sbjct  425  ATAACGCCCTCTTGTTTTATCAAGAGGGAAGCTATTGCTCCTTTTTTTTATTTAGTACTA  366

Query  182  TTTGCCTTACATAGTTTCTTTAAA-------AATAACAAGTACTTTTTATAGTTTGGTTC  234
            ||||||||||||||||||||||||       |||||||||||||||||||||||||||||
Sbjct  365  TTTGCCTTACATAGTTTCTTTAAACTTTAAAAATAACAAGTACTTTTTATAGTTTGGTTC  306

Query  235  GATTTGCGTGttttttctttgtattcatttatattataggtttctatatcatatattttt  294
            ||||||  |||||||| |||||||||||||||||||||||||||||||||||||| ||||
Sbjct  305  GATTTGTTTGTTTTTTATTTGTATTCATTTATATTATAGGTTTCTATATCATATACTTTT  246
```

```
>NC_053291.1 Aldama anchusifolia voucher Filartiga 8 chloroplast, complete
genome
 MN337902.1 Aldama anchusifolia chloroplast, complete genome
Length=151330

 Score = 459 bits (248),  Expect = 2e-124
 Identities = 284/300 (95%), Gaps = 7/300 (2%)
 Strand=Plus/Minus

Query  2    TTATGCATGAACGTAATGCTCATAATTTCCCTCTAGACTTAGCTGCTATTGAAGCTCCAT  61
            ||||||||||||||||||||||||||||||||||||||||||||||||||||||||||||
Sbjct  546  TTATGCATGAACGTAATGCTCATAATTTCCCTCTAGACTTAGCTGCTATTGAAGCTCCAT  487

Query  62   CTACAAATGGATAAGCCTTTGGTCTGATTGTATAGGAGTTTTTGAACTAAAAAAGGAGCA  121
            ||||||||||||||| ||||||||||||||||||||||||||||||||||||||||||||
Sbjct  486  CTACAAATGGATAAGACTTTGGTCTGATTGTATAGGAGTTTTTGAACTAAAAAAGGAGCA  427

Query  122  ATAACGCCCTCTTGATAAAACAAGAGGGAAGCTATTGCTCCttttttttATTTAGTACTA  181
            |||||||||||||| |  | ||||||||||||||||||||||||||||||||||||||||
Sbjct  426  ATAACGCCCTCTTGTTTTATCAAGAGGGAAGCTATTGCTCCTTTTTTTTATTTAGTACTA  367

Query  182  TTTGCCTTACATAGTTTCTTTAAA-------AATAACAAGTACTTTTTATAGTTTGGTTC  234
            ||||||||||||||||||||||||       |||||||||||||||||||||||||||||
Sbjct  366  TTTGCCTTACATAGTTTCTTTAAACTTTAAAAATAACAAGTACTTTTTATAGTTTGGTTC  307

Query  235  GATTTGCGTGtttttctttgtattcatttatattataggtttctatatcatatattttt  294
            ||||||  |||||||| ||||||||||||||||||||||||||||||||||||||| ||||
Sbjct  306  GATTTGTTTGTTTTTTATTTGTATTCATTTATATTATAGGTTTCTATATCATATACTTTT  247


>NC_053297.1 Aldama corumbensis voucher Loeuille 909 chloroplast, complete
genome
 MN337909.1 Aldama corumbensis chloroplast, complete genome
Length=151327

 Score = 459 bits (248),  Expect = 2e-124
 Identities = 284/300 (95%), Gaps = 7/300 (2%)
 Strand=Plus/Minus

Query  2    TTATGCATGAACGTAATGCTCATAATTTCCCTCTAGACTTAGCTGCTATTGAAGCTCCAT  61
            ||||||||||||||||||||||||||||||||||||||||||||||||||||||||||||
Sbjct  544  TTATGCATGAACGTAATGCTCATAATTTCCCTCTAGACTTAGCTGCTATTGAAGCTCCAT  485

Query  62   CTACAAATGGATAAGCCTTTGGTCTGATTGTATAGGAGTTTTTGAACTAAAAAAGGAGCA  121
            ||||||||||||||| ||||||||||||||||||||||||||||||||||||||||||||
Sbjct  484  CTACAAATGGATAAGACTTTGGTCTGATTGTATAGGAGTTTTTGAACTAAAAAAGGAGCA  425

Query  122  ATAACGCCCTCTTGATAAAACAAGAGGGAAGCTATTGCTCCttttttttATTTAGTACTA  181
            |||||||||||||| |  | ||||||||||||||||||||||||||||||||||||||||
Sbjct  424  ATAACGCCCTCTTGTTTTATCAAGAGGGAAGCTATTGCTCCTTTTTTTTATTTAGTACTA  365

Query  182  TTTGCCTTACATAGTTTCTTTAAA-------AATAACAAGTACTTTTTATAGTTTGGTTC  234
            ||||||||||||||||||||||||       |||||||||||||||||||||||||||||
Sbjct  364  TTTGCCTTACATAGTTTCTTTAAACTTTAAAAATAACAAGTACTTTTTATAGTTTGGTTC  305

Query  235  GATTTGCGTGtttttctttgtattcatttatattataggtttctatatcatatattttt  294
            ||||||  |||||||| ||||||||||||||||||||||||||||||||||||||| ||||
```

```
Sbjct  304  GATTTGTTTGTTTTTTATTTGTATTCATTTATATTATAGGTTTCTATATCATATACTTTT  245


>AY215553.1 Helenium bigelovii voucher Baldwin 681 (DAV) psbA gene and psbA-trnH
intergenic spacer, partial sequence; chloroplast
Length=422

 Score = 459 bits (248),  Expect = 2e-124
 Identities = 265/273 (97%), Gaps = 2/273 (1%)
 Strand=Plus/Minus

Query  28   TTCCCTCTAGACTTAGCTGCTATTGAAGCTCCATCTACAAATGGATAAGCCTTTGGTCTG  87
            |||||||||||||||||||||||||||||||||||||||||||||||||| ||||||||||
Sbjct  422  TTCCCTCTAGACTTAGCTGCTATTGAAGCTCCATCTACAAATGGATAAGACTTTGGTCTG  363

Query  88   ATTGTATAGGAGTTTTTGAACTAAAAAAGGAGCAATAACGCCCTCTTGATAAAACAAGAG  147
            ||||||||||||||||||||||||||||||||||||||||||||||||||||||||||||
Sbjct  362  ATTGTATAGGAGTTTTTGAACTAAAAAAGGAGCAATAACGCCCTCTTGATAAAACAAGAG  303

Query  148  GGAAGCTATTGCTCCttttttttATTTAGTACTATTTGCCTTACATAGTTTCTTTAAAAA  207
            ||||||||||||||||||||||| |||||||||||||| |||||||||||||||||||||
Sbjct  302  GGAAGCTATTGCTCCTTTTTTT-ATTTAGTACTATTTTCCTTACATAGTTTCTTTAAAAA  244

Query  208  TAACAAGTACTTTTTATAGTTTGGTTCGATTTGCGTGtttttctttgtattcatttata  267
            ||||||| |||||||||||||||||||||||||||||||| | ||||||||||||||||
Sbjct  243  TAACAAGGACTTTTTATAGTTTGGTTCGATTTGCGTGTTTTCTGTTTGTATTCATTTATA  184

Query  268  ttataggtttctatatcatatatttttcCCAAT  300
            |||||||||||||||||||| | ||||||||||
Sbjct  183  TTATAGGTTTCTATATCATA-ACTTTTCCCAAT  152


  Database: Core nucleotide BLAST database
    Posted date:  Sep 11, 2025  11:25 PM
  Number of letters in database: 922,953,073,383
  Number of sequences in database:  118,225,923

Lambda      K        H
    1.33    0.621     1.12
Gapped
Lambda      K        H
    1.28    0.460    0.850
Matrix: blastn matrix:1 -2
Gap Penalties: Existence: 0, Extension: 0
Number of Sequences: 118225923
Number of Hits to DB: 0
Number of extensions: 0
Number of successful extensions: 0
Number of sequences better than 0.05: 1181
Number of HSP's better than 0.05 without gapping: 0
Number of HSP's gapped: 1383
Number of HSP's successfully gapped: 1376
Length of query: 301
Length of database: 922953073383
Length adjustment: 35
Effective length of query: 266
Effective length of database: 918815166078
```

```
Effective search space: 244404834176748
Effective search space used: 244404834176748
A: 0
X1: 13 (25.0 bits)
X2: 32 (59.1 bits)
X3: 54 (99.7 bits)
S1: 13 (25.1 bits)
S2: 28 (52.8 bits)
```
