# Supplementary material for: Organ-Resolved Salicylate–Phenylpropanoid Metabolite Profiling of Echinacea purpurea, E. pallida, and E. tennesseensis Tissues Extracted with 50% Aqueous Methanol by LC–MS/MS and LC–HRMS
Source: Molecules. 2026 Aug 25;31(17):2963. doi: 10.3390/molecules31172963 (PMC13567413; doi:10.3390/molecules31172963)
Supplement: Supplementary file 1 [file molecules-31-02963-s001.zip › SupplementaryFiles/Supplementary Fig S3.pdf]

Plant Identification Report
Lot: 119665250903-1 Echinacea tennesseensis leaves

Date reported: 17-Sep-25

Reported by: mdu

Call: **Best matches *Echinacea pallida*.**

Comments: This sample from dried plant leaves. *Echinacea angustifolia* was also identified with generic NS primers as the only *Echinacea* species (results not shown); however, GenBank has low amounts of full 18S accessions for other *Echinacea* species and therefore this marker cannot be relied on for ID.

Instrument name: SimpliAmp; SeqStudio

Primer(s) used/Sequenced by: Generic trnH-psbA primers/mdu

Extraction Method/Extracted by: RIDA kit/mdu

DNAseq Run File: Seq25091501

```
BLASTN 2.17.0+
Reference: Zheng Zhang, Scott Schwartz, Lukas Wagner, and
Webb Miller (2000), "A greedy algorithm for aligning DNA
sequences", J Comput Biol 2000; 7(1-2):203-14.


Reference for database indexing: Aleksandr Morgulis, George
Coulouris, Yan Raytselis, Thomas L. Madden, Richa Agarwala,
Alejandro A. Schaffer (2008), "Database Indexing for
Production MegaBLAST Searches", Bioinformatics 24:1757-1764.


RID: CPB9XBAM014


Database: Core nucleotide BLAST database
           118,456,123 sequences; 925,171,125,281 total letters
Query=
Length=506
```

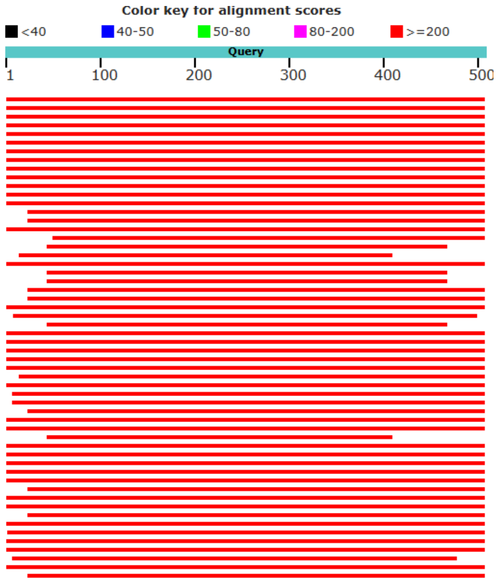

Sequences producing significant alignments:	Score (Bits)	E Value	Max Ident
**EU785935.1 Echinacea pallida PsbA (psbA) gene, partial cds; ps...**	**776**	**0.0**	**95%**
**NC_034321.1 Echinacea pallida plastid, complete genome**	**776**	**0.0**	**95%**
NC_034328.1 Echinacea sanguinea plastid, complete genome	765	0.0	95%
NC_034320.1 Echinacea paradoxa plastid, complete genome	763	0.0	95%
NC_034323.1 Echinacea atrorubens plastid, complete genome	763	0.0	95%
NC_034326.1 Echinacea tennesseensis plastid, complete genome	760	0.0	95%
NC_034325.1 Echinacea speciosa plastid, complete genome	760	0.0	95%
EU785933.1 Echinacea angustifolia PsbA (psbA) gene, partial cd...	758	0.0	95%
EU785934.1 Echinacea purpurea PsbA (psbA) gene, partial cds; p...	754	0.0	94%
ON641367.1 Echinacea purpurea isolate BPTPS168 voucher LISE:96...	754	0.0	94%
NC_034322.1 Echinacea laevigata plastid, complete genome	750	0.0	94%
MK055334.1 Echinacea purpurea plastid, complete genome	750	0.0	94%
NC_034324.1 Echinacea angustifolia plastid, complete genome	747	0.0	94%
MF348644.1 Echinacea pallida isolate AD3HJ47 photosystem II pr...	715	0.0	94%
MF348389.1 Echinacea pallida isolate AD0EE57 photosystem II pr...	715	0.0	94%
NC_034327.1 Echinacea purpurea plastid, complete genome	706	0.0	93%
LS999859.1 Echinacea angustifolia DNA containing psbA-trnH IGS...	667	0.0	94%
MF037494.1 Echinacea sanguinea voucher Bengtson9 trnH-psbA int...	664	0.0	96%
LS999860.1 Echinacea pallida DNA containing psbA-trnH IGS, spe...	662	0.0	97%
NC_081940.1 Enydra sessilis chloroplast, complete genome	654	0.0	91%
MF037491.1 Echinacea atrorubens voucher Barnard1532 trnH-psbA ...	651	0.0	95%
MF037490.1 Echinacea angustifolia voucher Rolfsmeier19250 trnH...	649	0.0	95%
MF348385.1 Echinacea purpurea isolate AD0EG44 photosystem II p...	645	2e-180	91%
MF348589.1 Echinacea purpurea isolate AD3HL50 photosystem II p...	645	2e-180	91%
MZ292978.1 Acmella paniculata chloroplast, complete genome	634	4e-177	90%
KF241798.1 Ambrosia artemisiifolia isolate AZ-1 PsbA (psbA) ge...	634	4e-177	91%
MF037492.1 Echinacea pallida voucher Rebman1053 trnH-psbA inte...	623	9e-174	94%
AB427162.1 Ambrosia artemisiifolia chloroplast psbA gene for D...	617	4e-172	90%
NC_035875.1 Ambrosia artemisiifolia chloroplast, complete genome	617	4e-172	90%
MG019037.1 Ambrosia artemisiifolia chloroplast, complete genome	617	4e-172	90%
NC_036810.2 Ambrosia trifida chloroplast, complete genome	617	4e-172	90%
NC_054222.1 Xanthium spinosum chloroplast, complete genome	608	3e-169	89%
MZ324674.1 Hulsea algida voucher Baldwin 678 (DAV) photosystem...	608	3e-169	90%
NC_053299.1 Iostephane heterophylla voucher Schilling 94 chlor...	606	9e-169	89%
KF241797.1 Ambrosia artemisiifolia isolate PH-53 PsbA (psbA) g...	601	4e-167	89%
KF241793.1 Ambrosia artemisiifolia isolate NJI-7 PsbA (psbA) g...	601	4e-167	89%
MF348657.1 Acmella oleracea isolate AD3HI37 photosystem II pro...	599	2e-166	90%
MT793838.1 Mikania lehmannii chloroplast, complete genome	593	7e-165	89%
MT793855.1 Mikania sylvatica chloroplast, complete genome	593	7e-165	89%
MF037493.1 Echinacea paradoxa voucher Rebman1085 trnH-psbA int...	592	3e-164	96%
NC_053285.1 Aldama linearis voucher Schilling 70 chloroplast, ...	590	9e-164	88%
NC_060408.1 Silphium perfoliatum chloroplast, complete sequence	588	3e-163	88%
NC_081944.1 Venegasia carpesioides voucher US Weitzman et al. ...	586	1e-162	88%
OZ286192.1 Venegasia carpesioides genome assembly, organelle: ...	586	1e-162	88%
NC_057172.1 Aldama excelsa voucher Schilling H2481 chloroplast...	584	4e-162	88%
OK469409.1 Eupatorieae sp. Gostel 807 voucher BRIT:Gostel807 p...	584	4e-162	89%
MZ636529.1 Xanthium spinosum chloroplast, complete genome	584	4e-162	88%
NC_072298.1 Xanthium strumarium chloroplast, complete genome	584	4e-162	88%
MF348479.1 Rudbeckia triloba isolate AD5JS71 photosystem II pr...	584	4e-162	89%
NC_042232.1 Xanthium sibiricum chloroplast, complete genome	584	4e-162	88%
KX346912.1 Xanthium sibiricum voucher S-CEZi-04 PsbA (psbA) ge...	582	2e-161	88%
NC_081937.1 Greenmaniella resinosa voucher Baldwin 977 (UC) ch...	582	2e-161	88%
NC_068130.1 Silphium integrifolium chloroplast, complete genome	582	2e-161	88%

KF241795.1 Ambrosia artemisiifolia isolate NJF-12 PsbA (psbA) ...	582	2e-161	90%
NC_053296.1 Aldama canescens voucher Schilling 17 chloroplast,...	580	6e-161	88%
HQ596826.1 Rudbeckia triloba voucher AP397 trnH-psbA intergeni...	579	2e-160	89%
GQ248245.1 Ambrosia trifida voucher NMNH Wells 30 psbA-trnH in...	577	7e-160	89%
DQ006141.1 Ambrosia artemisiifolia voucher Wells 4495 US PsbA ...	577	7e-160	89%
AY215541.1 Enydra sessilis voucher Panero 3126 (TEX) psbA gene...	575	3e-159	91%
NC_057174.1 Aldama robusta voucher Bringel 985 chloroplast, co...	575	3e-159	88%
NC_053279.1 Aldama filifolia voucher Loeuille 849 chloroplast,...	575	3e-159	88%
NC_057176.1 Aldama santacatarinensis voucher Magenta 706 chlor...	575	3e-159	88%
NC_053290.1 Aldama pilosa voucher Filartiga 10 chloroplast, co...	575	3e-159	88%
MT793848.1 Litothamnus nitidus chloroplast, complete genome	575	3e-159	88%
NC_053297.1 Aldama corumbensis voucher Loeuille 909 chloroplas...	575	3e-159	88%
OZ247556.1 Inula salicina genome assembly, organelle: plastid:...	573	9e-159	88%
MT576958.1 Tithonia diversifolia chloroplast, complete genome	573	9e-159	88%
KX346913.1 Xanthium sibiricum voucher S-CEZi-07 PsbA (psbA) ge...	573	9e-159	88%
NC_053300.1 Tithonia diversifolia voucher Loeuille 678 chlorop...	573	9e-159	88%
GQ248244.1 Ambrosia artemisiifolia voucher NMNH Wells 4495 psb...	573	9e-159	89%
OZ244507.1 Inula conyza genome assembly, organelle: plastid:ch...	573	9e-159	88%
MF348836.1 Chrysogonum virginianum isolate AG2KK87 photosystem...	573	9e-159	89%
MT700543.1 Pappobolus lanatus var. lanatus chloroplast, comple...	573	9e-159	88%
NC_068630.1 Duhaldea cappa chloroplast, complete genome	573	9e-159	88%
DQ006148.1 Chrysogonum virginianum voucher Beyersdorfer 113 US...	573	9e-159	89%
KY940274.1 Sphagneticola trilobata chloroplast, complete genome	573	9e-159	88%
NC_063729.1 Inula japonica chloroplast, complete genome	571	3e-158	88%
NC_072347.1 Pentanema britannicum chloroplast, complete genome	571	3e-158	88%
MW770456.1 Inula helianthus-aquatilis chloroplast, complete ge...	571	3e-158	88%
KX261063.1 Bahia ambrosioides PsbA gene, partial cds; and psbA...	571	3e-158	88%
HQ596592.1 Ambrosia artemisiifolia voucher AP337 trnH-psbA int...	571	3e-158	89%
MW770455.1 Inula helianthus-aquatilis chloroplast, complete ge...	571	3e-158	88%
NC_063571.1 Inula linariifolia chloroplast, complete genome	571	3e-158	88%
MN974527.1 Inula japonica chloroplast, complete genome	571	3e-158	88%
NC_053284.1 Aldama kunthiana voucher voucher Silva s.n. (ESA 1...	569	1e-157	88%
NC_057177.1 Aldama squalida voucher Loeuille 790 chloroplast, ...	569	1e-157	88%
NC_053295.1 Aldama bracteata voucher Filartiga 15 chloroplast,...	569	1e-157	88%
NC_057171.1 Aldama discolor voucher Bombo 72 chloroplast, comp...	569	1e-157	88%
NC_053282.1 Aldama goyazii voucher Magenta 716 chloroplast, co...	569	1e-157	88%
NC_053294.1 Aldama bakeriana voucher Loeuille 867 chloroplast,...	569	1e-157	88%
NC_053286.1 Aldama macrorhiza voucher Magenta 476 chloroplast,...	569	1e-157	88%
NC_057181.1 Aldama veredensis voucher Loeuille 921 chloroplast...	569	1e-157	88%
NC_057178.1 Aldama tenuifolia voucher Silva s.n. (ESA 122870) ...	569	1e-157	88%
NC_053293.1 Aldama aspilioides voucher Filartiga 18 chloroplas...	569	1e-157	88%
NC_086875.1 Helianthus agrestis isolate USDA PI 673199 cultiva...	568	4e-157	88%
KF746370.1 Helianthus grosseserratus isolate DB25 plastid, com...	568	4e-157	88%
OQ832296.1 Xanthium strumarium voucher MONT:87396 photosystem ...	568	4e-157	92%
OQ434029.1 Ambrosia trifida clone 1039837_5 trnH-psbA intergen...	568	4e-157	90%
OQ404763.1 Ambrosia artemisiifolia trnH-psbA intergenic spacer...	568	4e-157	90%
NC_023109.1 Helianthus divaricatus isolate DB07 plastid, compl...	568	4e-157	88%

ALIGNMENTS
>EU785935.1 Echinacea pallida PsbA (psbA) gene, partial cds; psbA-trnH intergenic
spacer, complete sequence; and tRNA-His (trnH) gene,
partial sequence; chloroplast
Length=508

Score = 776 bits (420), Expect = 0.0
Identities = 479/503 (95%), Gaps = 22/503 (4%)
Strand=Plus/Plus

```
Query  2    GTTATGCATGAACGTAATGCTCATAATTTCCCTCTAGACTTAGCTGCTATTGAAGCTCCA  61
            ||||||||||||||||||||||||||||||||||||||||||||||||||||||||||||
Sbjct  1    GTTATGCATGAACGTAATGCTCATAATTTCCCTCTAGACTTAGCTGCTATTGAAGCTCCA  60

Query  62   TCTACAAATGGATAAGCCTTTGGTCTGATTGTATAGGAGTTTTTGAACTAAAAAAGGAGC  121
            ||||||||||||||||||||||||||||||||||||||||||||||||||||||||||||
Sbjct  61   TCTACAAATGGATAAGCCTTTGGTCTGATTGTATAGGAGTTTTTGAACTAAAAAAGGAGC  120

Query  122  AATAACGCCCTCTTGATAAAACAAGAGGGAAGCTATTGCTCCtttttttATTTAGTACTA  181
            ||||||||||||||||||||||||||||||||||||||||||||||||||||||||||||
Sbjct  121  AATAACGCCCTCTTGATAAAACAAGAGGGAAGCTATTGCTCCTTTTTTTATTTAGTACTA  180

Query  182  TTTGCCTTACATAGTTTCTTTAAAAATAACAAGTACTTTTTATAGTTTGGTTCGatttgc  241
            ||||||||||||||||||||||||||||||||||||||||||||||||||||||||||||
Sbjct  181  TTTGCCTTACATAGTTTCTTTAAAAATAACAAGTACTTTTTATAGTTTGGTTCGATTTGC  240

Query  242  gtgtttttctttgtattcatttatattataggtttctatatcatttatttttcccaatc  301
            ||||||||||||||||||||||||||||||||||||||||||||||||||||||||||||
Sbjct  241  GTGTTTTTCTTTGTATTCATTTATATTATAGGTTTCTATATCATTTATTTTTCCCAATC  300

Query  302  ttttatgaagttttatttaCAATTTAAATTTCAATCGaaaaaaagagaaaaaatgacaaa  361
            ||||||||||||||||||||||||| ||||||||||||||| | |||||||| ||||||
Sbjct  301  TTTTATGAAGTTTTATTTACAATTT-AATTTCAATCGAAAATA-GAGAAAAA-TGACAA-  356

Query  362  ttttgcttttttgattttatattgattaaaatcaaaaataaagaaaagaattttgctttt  421
            ||||||||||| || ||||||||||||||| |||||| ||| |||| |||| | ||||||
Sbjct  357  TTTTGCTTTTT-GA-TTTATATTGATTAAA-TCAAAA-TAA-GAAA-GAATAT-GCTTTT  409

Query  422  ttttATTGTTAAATGGaaaaaaaTATAGTTAATACTAGATAATCCGTAGAAGGGGGCGGG  481
            ||| || ||||| ||||||||| ||||| ||||||||||||||| ||||| |||| |||
Sbjct  410  TTT-AT-GTTAA-TGGAAAAAA-TATAG-TAATACTAGATAATC-GTAGA-GGGG-CGG-  460

Query  482  ATGTAGGCCAAAGTGGATCAAGG  504
            |||||| |||| |||||||||||
Sbjct  461  ATGTAG-CCAA-GTGGATCAAGG  481


>NC_034321.1 Echinacea pallida plastid, complete genome
 KX548218.1 Echinacea pallida plastid, complete genome
Length=151883

 Score = 776 bits (420),  Expect = 0.0
 Identities = 479/503 (95%), Gaps = 22/503 (4%)
 Strand=Plus/Minus

Query  2    GTTATGCATGAACGTAATGCTCATAATTTCCCTCTAGACTTAGCTGCTATTGAAGCTCCA  61
            ||||||||||||||||||||||||||||||||||||||||||||||||||||||||||||
Sbjct  534  GTTATGCATGAACGTAATGCTCATAATTTCCCTCTAGACTTAGCTGCTATTGAAGCTCCA  475

Query  62   TCTACAAATGGATAAGCCTTTGGTCTGATTGTATAGGAGTTTTTGAACTAAAAAAGGAGC  121
            ||||||||||||||||||||||||||||||||||||||||||||||||||||||||||||
Sbjct  474  TCTACAAATGGATAAGCCTTTGGTCTGATTGTATAGGAGTTTTTGAACTAAAAAAGGAGC  415

Query  122  AATAACGCCCTCTTGATAAAACAAGAGGGAAGCTATTGCTCCtttttttATTTAGTACTA  181
            ||||||||||||||||||||||||||||||||||||||||||||||||||||||||||||
Sbjct  414  AATAACGCCCTCTTGATAAAACAAGAGGGAAGCTATTGCTCCTTTTTTTATTTAGTACTA  355
```

```
Query  182   TTTGCCTTACATAGTTTCTTTAAAAATAACAAGTACTTTTTATAGTTTGGTTCGatttgc  241
             ||||||||||||||||||||||||||||||||||||||||||||||||||||||||||||
Sbjct  354   TTTGCCTTACATAGTTTCTTTAAAAATAACAAGTACTTTTTATAGTTTGGTTCGATTTGC  295

Query  242   gtgtttttctttgtattcatttatattataggtttctatatcatttatttttcccaatc  301
             ||||||||||||||||||||||||||||||||||||||||||||||||||||||||||||
Sbjct  294   GTGTTTTTCTTTGTATTCATTTATATTATAGGTTTCTATATCATTTATTTTTCCCAATC  235

Query  302   ttttatgaagttttatttaCAATTTAAATTTCAATCGaaaaaaagagaaaaaaatgacaaa  361
             ||||||||||||||||||||||||||| ||||||||||||| | |||||||| ||||||
Sbjct  234   TTTTATGAAGTTTTATTTACAATTTAA-TTTCAATCGAAAATA-GAGAAAAA-TGACAA-  179

Query  362   ttttgcttttttgattttatattgattaaaatcaaaaataaagaaaagaattttgctttt  421
             ||||||||||| || ||||||||||||||| |||||| ||| |||| |||| | ||||||
Sbjct  178   TTTTGCTTTTT-GA-TTTATATTGATTAAA-TCAAAA-TAA-GAAA-GAATAT-GCTTTT  126

Query  422   ttttATTGTTAAATGGaaaaaaaTATAGTTAATACTAGATAATCCGTAGAAGGGGGCGGG  481
             ||| || ||||| |||||||||| ||||| |||||||||||||||| ||||| |||| |||
Sbjct  125   TTT-AT-GTTAA-TGGAAAAAA-TATAG-TAATACTAGATAATC-GTAGA-GGGG-CGG-  75

Query  482   ATGTAGGCCAAAGTGGATCAAGG  504
             |||||| |||| |||||||||||
Sbjct  74    ATGTAG-CCAA-GTGGATCAAGG  54


>NC_034328.1 Echinacea sanguinea plastid, complete genome
 KX548225.1 Echinacea sanguinea plastid, complete genome
Length=151926

 Score = 765 bits (414),  Expect = 0.0
 Identities = 477/503 (95%), Gaps = 22/503 (4%)
 Strand=Plus/Minus

Query  2     GTTATGCATGAACGTAATGCTCATAATTTCCCTCTAGACTTAGCTGCTATTGAAGCTCCA  61
             ||||||||||||||||||||||||||||||||||||||||||||||||||||||||||||
Sbjct  534   GTTATGCATGAACGTAATGCTCATAATTTCCCTCTAGACTTAGCTGCTATTGAAGCTCCA  475

Query  62    TCTACAAATGGATAAGCCTTTGGTCTGATTGTATAGGAGTTTTTGAACTAAAAAAGGAGC  121
             ||||||||||||||||||||||||||||||||||||||||||||||||||||||||||||
Sbjct  474   TCTACAAATGGATAAGCCTTTGGTCTGATTGTATAGGAGTTTTTGAACTAAAAAAGGAGC  415

Query  122   AATAACGCCCTCTTGATAAAACAAGAGGGAAGCTATTGCTCCtttttttATTTAGTACTA  181
             ||||||||||||||||||||||||||||||||||||||||||||||||||||||||||||
Sbjct  414   AATAACGCCCTCTTGATAAAACAAGAGGGAAGCTATTGCTCCTTTTTTTATTTAGTACTA  355

Query  182   TTTGCCTTACATAGTTTCTTTAAAAATAACAAGTACTTTTTATAGTTTGGTTCGatttgc  241
             ||||||||||||||||||||||||||||||||||||||||||||||||||||||||||||
Sbjct  354   TTTGCCTTACATAGTTTCTTTAAAAATAACAAGTACTTTTTATAGTTTGGTTCGATTTGC  295

Query  242   gtgtttttctttgtattcatttatattataggtttctatatcatttatttttcccaatc  301
             |||||||||||||||||||||||||||||||||||||||||||||||||||||||||||
Sbjct  294   GTGTTTTTCTTTGTATTCATTTATATTATAGGTTTCTATATCATTTATTTTTCCCAATA  235

Query  302   ttttatgaagttttatttaCAATTTAAATTTCAATCGaaaaaaagagaaaaaaatgacaaa  361
             ||||||||||||||||||||||||||| ||||||||||||| | || ||||| |||| ||
Sbjct  234   TTTTATGAAGTTTTATTTACAATTTAA-TTTCAATCGAAAATA-GATAAAAA-TGAC-AA  179
```

```
Query  362   ttttgctttttttgatttatattgattaaaatcaaaaataaagaaaagaattttgctttt  421
             |||||||||||| | ||||||||||||||| |||||| ||| |||| |||| | ||||||
Sbjct  178   TTTTGCTTTTTT-A-TTTATATTGATTAAA-TCAAAA-TAA-GAAA-GAATAT-GCTTTT  126

Query  422   ttttATTGTTAAATGGaaaaaaaTATAGTTAATACTAGATAATCCGTAGAAGGGGGCGGG  481
             ||| || ||||| ||||||||| ||||| ||||||||||||||| ||||| |||| ||| 
Sbjct  125   TTT-AT-GTTAA-TGGAAAAAA-TATAG-TAATACTAGATAATC-GTAGA-GGGG-CGG-  75

Query  482   ATGTAGGCCAAAGTGGATCAAGG  504
             |||||| |||| |||||||||||
Sbjct  74    ATGTAG-CCAA-GTGGATCAAGG  54


>NC_034320.1 Echinacea paradoxa plastid, complete genome
 KX548217.1 Echinacea paradoxa plastid, complete genome
Length=151837

 Score = 763 bits (413),  Expect = 0.0
 Identities = 477/503 (95%), Gaps = 23/503 (5%)
 Strand=Plus/Minus

Query  2     GTTATGCATGAACGTAATGCTCATAATTTCCCTCTAGACTTAGCTGCTATTGAAGCTCCA  61
             ||||||||||||||||||||||||||||||||||||||||||||||||||||||||||||
Sbjct  533   GTTATGCATGAACGTAATGCTCATAATTTCCCTCTAGACTTAGCTGCTATTGAAGCTCCA  474

Query  62    TCTACAAATGGATAAGCCTTTGGTCTGATTGTATAGGAGTTTTTGAACTAAAAAAGGAGC  121
             ||||||||||||||||||||||||||||||||||||||||||||||||||||||||||||
Sbjct  473   TCTACAAATGGATAAGCCTTTGGTCTGATTGTATAGGAGTTTTTGAACTAAAAAAGGAGC  414

Query  122   AATAACGCCCTCTTGATAAAACAAGAGGGAAGCTATTGCTCCtttttttATTTAGTACTA  181
             ||||||| ||||||||||||||||||||||||||||||||||||||||||||||||||||
Sbjct  413   AATAACGTCCTCTTGATAAAACAAGAGGGAAGCTATTGCTCCTTTTTTTATTTAGTACTA  354

Query  182   TTTGCCTTACATAGTTTCTTTAAAAATAACAAGTACTTTTTATAGTTTGGTTCGatttgc  241
             ||||||||||||||||||||||||||||||||||||||||||||||||||||||||||||
Sbjct  353   TTTGCCTTACATAGTTTCTTTAAAAATAACAAGTACTTTTTATAGTTTGGTTCGATTTGC  294

Query  242   gtgtttttttctttgtattcatttatattataggtttctatatcatttatttttcccaatc  301
             ||||||||||||||||||||||||||||||||||||||||||||||||||||||||||||
Sbjct  293   GTGTTTTTTCTTTGTATTCATTTATATTATAGGTTTCTATATCATTTATTTTTCCCAATC  234

Query  302   ttttatgaagttttatttaCAATTTAAATTTCAATCGaaaaaaagagaaaaaatgacaaa  361
             ||||||||||||||||||||||||||| ||||||||||||| | |||||||| |||||| 
Sbjct  233   TTTTATGAAGTTTTATTTACAATTTAA-TTTCAATCGAAAATA-GAGAAAAA-TGACAA-  178

Query  362   ttttgctttttttgatttatattgattaaaatcaaaaataaagaaaagaattttgctttt  421
             ||||||||||| || ||||||||||||||| |||||| ||| |||| |||| | ||||||
Sbjct  177   TTTTGCTTTTT-GA-TTTATATTGATTAAA-TCAAAA-TAA-GAAA-GAATAT-GCTTTT  125

Query  422   ttttATTGTTAAATGGaaaaaaaTATAGTTAATACTAGATAATCCGTAGAAGGGGGCGGG  481
             ||  || ||||| ||||||||| ||||| ||||||||||||||| ||||| |||| ||| 
Sbjct  124   TT--AT-GTTAA-TGGAAAAAA-TATAG-TAATACTAGATAATC-GTAGA-GGGG-CGG-  75

Query  482   ATGTAGGCCAAAGTGGATCAAGG  504
             |||||| |||| |||||||||||
Sbjct  74    ATGTAG-CCAA-GTGGATCAAGG  54
```

```
>NC_034323.1 Echinacea atrorubens plastid, complete genome
 KX548220.1 Echinacea atrorubens plastid, complete genome
Length=151912

 Score = 763 bits (413),  Expect = 0.0
 Identities = 477/503 (95%), Gaps = 23/503 (5%)
 Strand=Plus/Minus

Query  2    GTTATGCATGAACGTAATGCTCATAATTTCCCTCTAGACTTAGCTGCTATTGAAGCTCCA  61
            ||||||||||||||||||||||||||||||||||||||||||||||||||||||||||||
Sbjct  533  GTTATGCATGAACGTAATGCTCATAATTTCCCTCTAGACTTAGCTGCTATTGAAGCTCCA  474

Query  62   TCTACAAATGGATAAGCCTTTGGTCTGATTGTATAGGAGTTTTTGAACTAAAAAAGGAGC  121
            ||||||||||||||||||||||||||||||||||||||||||||||||||||||||||||
Sbjct  473  TCTACAAATGGATAAGCCTTTGGTCTGATTGTATAGGAGTTTTTGAACTAAAAAAGGAGC  414

Query  122  AATAACGCCCTCTTGATAAAACAAGAGGGAAGCTATTGCTCCtttttttATTTAGTACTA  181
            ||||||| ||||||||||||||||||||||||||||||||||||||||||||||||||||
Sbjct  413  AATAACGTCCTCTTGATAAAACAAGAGGGAAGCTATTGCTCCTTTTTTTATTTAGTACTA  354

Query  182  TTTGCCTTACATAGTTTCTTTAAAAATAACAAGTACTTTTTATAGTTTGGTTCGatttgc  241
            ||||||||||||||||||||||||||||||||||||||||||||||||||||||||||||
Sbjct  353  TTTGCCTTACATAGTTTCTTTAAAAATAACAAGTACTTTTTATAGTTTGGTTCGATTTGC  294

Query  242  gtgtttttctttgtattcatttatattataggtttctatatcatttatttttcccaatc  301
            ||||||||||||||||||||||||||||||||||||||||||||||||||||||||||||
Sbjct  293  GTGTTTTTCTTTGTATTCATTTATATTATAGGTTTCTATATCATTTATTTTTCCCAATC  234

Query  302  ttttatgaagttttatttaCAATTTAAATTTCAATCGaaaaaaagagaaaaaaatgacaaa  361
            ||||||||||||||||||||||||||| ||||||||||||| | |||||||| ||||||
Sbjct  233  TTTTATGAAGTTTTATTTACAATTTAA-TTTCAATCGAAAATA-GAGAAAAA-TGACAA-  178

Query  362  ttttgctttttgattttatattgattaaaatcaaaaataaagaaaagaattttgctttt  421
            ||||||||||| || ||||||||||||||| |||||| ||| |||| |||| | ||||||
Sbjct  177  TTTTGCTTTTT-GA-TTTATATTGATTAAA-TCAAAA-TAA-GAAA-GAATAT-GCTTTT  125

Query  422  ttttATTGTTAAATGGaaaaaaaTATAGTTAATACTAGATAATCCGTAGAAGGGGGCGGG  481
            ||  || ||||| ||||||||| ||||| ||||||||||||||| ||||| |||| |||
Sbjct  124  TT--AT-GTTAA-TGGAAAAAA-TATAG-TAATACTAGATAATC-GTAGA-GGGG-CGG-  75

Query  482  ATGTAGGCCAAAGTGGATCAAGG  504
            |||||| |||| |||||||||||
Sbjct  74   ATGTAG-CCAA-GTGGATCAAGG  54


>NC_034326.1 Echinacea tennesseensis plastid, complete genome
 KX548223.1 Echinacea tennesseensis plastid, complete genome
Length=151877

 Score = 760 bits (411),  Expect = 0.0
 Identities = 477/504 (95%), Gaps = 24/504 (5%)
 Strand=Plus/Minus

Query  2    GTTATGCATGAACGTAATGCTCATAATTTCCCTCTAGACTTAGCTGCTATTGAAGCTCCA  61
            ||||||||||||||||||||||||||||||||||||||||||||||||||||||||||||
```

```
Sbjct  534   GTTATGCATGAACGTAATGCTCATAATTTCCCTCTAGACTTAGCTGCTATTGAAGCTCCA  475

Query  62    TCTACAAATGGATAAGCCTTTGGTCTGATTGTATAGGAGTTTTTGAACTAAAAAAGGAGC  121
             ||||||||||||||||||||||||||||||||||||||||||||||||||||||||||||
Sbjct  474   TCTACAAATGGATAAGCCTTTGGTCTGATTGTATAGGAGTTTTTGAACTAAAAAAGGAGC  415

Query  122   AATAACGCCCTCTTGATAAAACAAGAGGGAAGCTATTGCTCCttttttt-ATTTAGTACT  180
             ||||||||||||||||||||||||||||||||||||||||||||||||| ||||||||||
Sbjct  414   AATAACGCCCTCTTGATAAAACAAGAGGGAAGCTATTGCTCCTTTTTTTTATTTAGTACT  355

Query  181   ATTTGCCTTACATAGTTTCTTTAAAAATAACAAGTACTTTTTATAGTTTGGTTCGatttg  240
             ||||||||||||||||||||||||||||||||||||||||||||||||||||||||||||
Sbjct  354   ATTTGCCTTACATAGTTTCTTTAAAAATAACAAGTACTTTTTATAGTTTGGTTCGATTTG  295

Query  241   cgtgtttttctttgtattcatttatattataggtttctatatcatttatttttcccaat  300
             ||||||||||||||||||||||||||||||||||||||||||||| ||||||||||||||
Sbjct  294   CGTGTTTTTCTTTGTATTCATTTATATTATAGGTTTCTATATCATATATTTTTCCCAAT  235

Query  301   cttttatgaagttttatttaCAATTTAAATTTCAATCGaaaaaaagagaaaaaatgacaa  360
             |||||||||||||||||||||||||||| ||||||||||||| | |||||||| ||||||
Sbjct  234   CTTTTATGAAGTTTTATTTACAATTTAA-TTTCAATCGAAAATA-GAGAAAAA-TGACAA  178

Query  361   attttgctttttgattttatattgattaaaatcaaaaataaagaaaagaattttgcttt  420
              ||||||||||| || ||||||||||||||| |||||| ||| |||| |||| | |||||
Sbjct  177   -TTTTGCTTTTT-GA-TTTATATTGATTAAA-TCAAAA-TAA-GAAA-GAATAT-GCTTT  126

Query  421   tttttATTGTTAAATGGaaaaaaaTATAGTTAATACTAGATAATCCGTAGAAGGGGGCGG  480
             |||| || ||||| ||||||||  ||||| ||||||||||||||| ||||| |||| |||
Sbjct  125   TTTT-AT-GTTAA-TGGAAAAA--TATAG-TAATACTAGATAATC-GTAGA-GGGG-CGG  75

Query  481   GATGTAGGCCAAAGTGGATCAAGG  504
              |||||| |||| |||||||||||
Sbjct  74    -ATGTAG-CCAA-GTGGATCAAGG  54


>NC_034325.1 Echinacea speciosa plastid, complete genome
 KX548222.1 Echinacea speciosa plastid, complete genome
Length=151860

 Score = 760 bits (411),  Expect = 0.0
 Identities = 477/504 (95%), Gaps = 24/504 (5%)
 Strand=Plus/Minus

Query  2     GTTATGCATGAACGTAATGCTCATAATTTCCCTCTAGACTTAGCTGCTATTGAAGCTCCA  61
             ||||||||||||||||||||||||||||||||||||||||||||||||||||||||||||
Sbjct  534   GTTATGCATGAACGTAATGCTCATAATTTCCCTCTAGACTTAGCTGCTATTGAAGCTCCA  475

Query  62    TCTACAAATGGATAAGCCTTTGGTCTGATTGTATAGGAGTTTTTGAACTAAAAAAGGAGC  121
             ||||||||||||||||||||||||||||||||||||||||||||||||||||||||||||
Sbjct  474   TCTACAAATGGATAAGCCTTTGGTCTGATTGTATAGGAGTTTTTGAACTAAAAAAGGAGC  415

Query  122   AATAACGCCCTCTTGATAAAACAAGAGGGAAGCTATTGCTCCttttttt-ATTTAGTACT  180
             ||||||||||||||||||||||||||||||||||||||||||||||||| ||||||||||
Sbjct  414   AATAACGCCCTCTTGATAAAACAAGAGGGAAGCTATTGCTCCTTTTTTTTATTTAGTACT  355

Query  181   ATTTGCCTTACATAGTTTCTTTAAAAATAACAAGTACTTTTTATAGTTTGGTTCGatttg  240
             ||||||||||||||||||||||||||||||||||||||||||||||||||||||||||||
```

```
Sbjct  354  ATTTGCCTTACATAGTTTCTTTAAAAATAACAAGTACTTTTTATAGTTTGGTTCGATTTG  295

Query  241  cgtgtttttctttgtattcatttatattataggtttctatatcatttatttttcccaat  300
            ||||||||||||||||||||||||||||||||||||||||||||| ||||||||||||||
Sbjct  294  CGTGTTTTTCTTTGTATTCATTTATATTATAGGTTTCTATATCATATATTTTTCCCAAT  235

Query  301  cttttatgaagttttatttaCAATTTAAATTTCAATCGaaaaaaagagaaaaaatgacaa  360
            |||||||||||||||||||||||||||| ||||||||||||| | |||||||| ||||||
Sbjct  234  CTTTTATGAAGTTTTATTTACAATTTAA-TTTCAATCGAAAATA-GAGAAAAA-TGACAA  178

Query  361  atttgcttttttgatttatattgattaaaatcaaaaataaagaaaagaattttgcttt  420
             ||||||||||| || ||||||||||||||| |||||| ||| |||| |||| | |||||
Sbjct  177  -TTTTGCTTTTT-GA-TTTATATTGATTAAA-TCAAAA-TAA-GAAA-GAATAT-GCTTT  126

Query  421  tttttATTGTTAAATGGaaaaaaaTATAGTTAATACTAGATAATCCGTAGAAGGGGGCGG  480
            |||| || ||||| ||||||||  ||||| ||||||||||||||| ||||| |||| |||
Sbjct  125  TTTT-AT-GTTAA-TGGAAAAA--TATAG-TAATACTAGATAATC-GTAGA-GGGG-CGG  75

Query  481  GATGTAGGCCAAAGTGGATCAAGG  504
             |||||| |||| |||||||||||
Sbjct  74   -ATGTAG-CCAA-GTGGATCAAGG  54


>EU785933.1 Echinacea angustifolia PsbA (psbA) gene, partial cds; psbA-trnH
intergenic spacer, complete sequence; and tRNA-His (trnH)
gene, partial sequence; chloroplast
Length=506

 Score = 758 bits (410),  Expect = 0.0
 Identities = 476/503 (95%), Gaps = 24/503 (5%)
 Strand=Plus/Plus

Query  2    GTTATGCATGAACGTAATGCTCATAATTTCCCTCTAGACTTAGCTGCTATTGAAGCTCCA  61
            ||||||||||||||||||||||||||||||||||||||||||||||||||||||||||||
Sbjct  1    GTTATGCATGAACGTAATGCTCATAATTTCCCTCTAGACTTAGCTGCTATTGAAGCTCCA  60

Query  62   TCTACAAATGGATAAGCCTTTGGTCTGATTGTATAGGAGTTTTTGAACTAAAAAAGGAGC  121
            ||||||||||||||||||||||||||||||||||||||||||||||||||||||||||||
Sbjct  61   TCTACAAATGGATAAGCCTTTGGTCTGATTGTATAGGAGTTTTTGAACTAAAAAAGGAGC  120

Query  122  AATAACGCCCTCTTGATAAAACAAGAGGGAAGCTATTGCTCCtttttttATTTAGTACTA  181
            ||||||||||||||||||||||||||||||||||||||||||||||||||||||||||||
Sbjct  121  AATAACGCCCTCTTGATAAAACAAGAGGGAAGCTATTGCTCCTTTTTTTATTTAGTACTA  180

Query  182  TTTGCCTTACATAGTTTCTTTAAAAATAACAAGTACTTTTTATAGTTTGGTTCGatttgc  241
            ||||||||||||||||||||||||||||||||||||||||||||||||||||||||||||
Sbjct  181  TTTGCCTTACATAGTTTCTTTAAAAATAACAAGTACTTTTTATAGTTTGGTTCGATTTGC  240

Query  242  gtgtttttctttgtattcatttatattataggtttctatatcatttatttttcccaatc  301
            |||||||||||||||||||||||||||||||||||||||||||| |||||||||||||||
Sbjct  241  GTGTTTTTCTTTGTATTCATTTATATTATAGGTTTCTATATCATATATTTTTCCCAATC  300

Query  302  ttttatgaagttttatttaCAATTTAAATTTCAATCGaaaaaaagagaaaaaatgacaaa  361
            ||||||||||||||||||||||||| ||||||||||||||| | |||||||| |||| ||
Sbjct  301  TTTTATGAAGTTTTATTTACAATTT-AATTTCAATCGAAAATA-GAGAAAAA-TGAC-AA  356

Query  362  ttttgctttttgatttatattgattaaaatcaaaaataaagaaaagaattttgctttt  421
```

```
                |||||||||||| | ||||||||||||||| |||||| ||| |||| |||| | ||||||
Sbjct  357  TTTTGCTTTTTT-A-TTTATATTGATTAAA-TCAAAA-TAA-GAAA-GAATAT-GCTTTT  409

Query  422  ttttATTGTTAAATGGaaaaaaaTATAGTTAATACTAGATAATCCGTAGAAGGGGGCGGG  481
            ||  || ||||| ||||||||  ||||| ||||||||||||||| ||||| |||| ||| 
Sbjct  410  TT--AT-GTTAA-TGGAAAAA--TATAG-TAATACTAGATAATC-GTAGA-GGGG-CGG-  458

Query  482  ATGTAGGCCAAAGTGGATCAAGG  504
            |||||| |||| |||||||||||
Sbjct  459  ATGTAG-CCAA-GTGGATCAAGG  479
```

>EU785934.1 Echinacea purpurea PsbA (psbA) gene, partial cds; psbA-trnH intergenic spacer, complete sequence; and tRNA-His (trnH) gene,
partial sequence; chloroplast
Length=508

Score = 754 bits (408), Expect = 0.0
Identities = 476/504 (94%), Gaps = 24/504 (5%)
Strand=Plus/Plus

```
Query  2    GTTATGCATGAACGTAATGCTCATAATTTCCCTCTAGACTTAGCTGCTATTGAAGCTCCA  61
            ||||||||||||||||||||||||||||||||||||||||||||||||||||||||||||
Sbjct  1    GTTATGCATGAACGTAATGCTCATAATTTCCCTCTAGACTTAGCTGCTATTGAAGCTCCA  60

Query  62   TCTACAAATGGATAAGCCTTTGGTCTGATTGTATAGGAGTTTTTGAACTAAAAAAGGAGC  121
            | ||||||||||||||||||||||||||||||||||||||||||||||||||||||||||
Sbjct  61   TATACAAATGGATAAGCCTTTGGTCTGATTGTATAGGAGTTTTTGAACTAAAAAAGGAGC  120

Query  122  AATAACGCCCTCTTGATAAAACAAGAGGGAAGCTATTGCTCC-ttttttATTTAGTACT  180
            |||||||||||||||||||||||||||||||||||||||||| |||||||||||||||||
Sbjct  121  AATAACGCCCTCTTGATAAAACAAGAGGGAAGCTATTGCTCCTTTTTTTATTTAGTACT  180

Query  181  ATTTGCCTTACATAGTTTCTTTAAAAATAACAAGTACTTTTTATAGTTTGGTTCGatttg  240
            ||||||||||||||||||||||||||||||||||||||||||||||||||||||||||||
Sbjct  181  ATTTGCCTTACATAGTTTCTTTAAAAATAACAAGTACTTTTTATAGTTTGGTTCGATTTG  240

Query  241  cgtgttttttctttgtattcatttatattataggtttctatatcatttatttttcccaat  300
            ||||||||||||||||||||||||||||||||||||||||||||||| |||||||||||||
Sbjct  241  CGTGTTTTTTCTTTGTATTCATTTATATTATAGGTTTCTATATCATATATTTTTCCCAAT  300

Query  301  cttttatgaagttttatttaCAATTTAAATTTCAATCGaaaaaaagagaaaaaatgacaa  360
            |||||||||||||||||||||||||| ||||||||||||||| | |||||||| ||||||
Sbjct  301  CTTTTATGAAGTTTTATTTACAATTT-AATTTCAATCGAAAATA-GAGAAAAA-TGACAA  357

Query  361  atttgcttttttgattttatattgattaaaatcaaaaataaagaaaagaattttgcttt  420
             ||||||||||| || ||||||||||||||| |||||| ||| |||| |||| | |||||
Sbjct  358  -TTTTGCTTTTT-GA-TTTATATTGATTAAA-TCAAAA-TAA-GAAA-GAATAT-GCTTT  409

Query  421  tttttATTGTTAAATGGaaaaaaaTATAGTTAATACTAGATAATCCGTAGAAGGGGGCGG  480
            |||| || ||||| ||||||||  ||||| ||||||||||||||| ||||| |||| |||
Sbjct  410  TTTT-AT-GTTAA-TGGAAAAA--TATAG-TAATACTAGATAATC-GTAGA-GGGG-CGG  460

Query  481  GATGTAGGCCAAAGTGGATCAAGG  504
             |||||| |||| |||||||||||
Sbjct  461  -ATGTAG-CCAA-GTGGATCAAGG  481
```

>ON641367.1 Echinacea purpurea isolate BPTPS168 voucher LISE:96479 chloroplast, complete genome
Length=151924

```
 Score = 754 bits (408),  Expect = 0.0
 Identities = 476/504 (94%), Gaps = 24/504 (5%)
 Strand=Plus/Plus

Query  2      GTTATGCATGAACGTAATGCTCATAATTTCCCTCTAGACTTAGCTGCTATTGAAGCTCCA  61
              ||||||||||||||||||||||||||||||||||||||||||||||||||||||||||||
Sbjct  83080  GTTATGCATGAACGTAATGCTCATAATTTCCCTCTAGACTTAGCTGCTATTGAAGCTCCA  83139

Query  62     TCTACAAATGGATAAGCCTTTGGTCTGATTGTATAGGAGTTTTTGAACTAAAAAAGGAGC  121
              | ||||||||||||||||||||||||||||||||||||||||||||||||||||||||||
Sbjct  83140  TATACAAATGGATAAGCCTTTGGTCTGATTGTATAGGAGTTTTTGAACTAAAAAAGGAGC  83199

Query  122    AATAACGCCCTCTTGATAAAACAAGAGGGAAGCTATTGCTCC-tttttttATTTAGTACT  180
              |||||||||||||||||||||||||||||||||||||||||| |||||||||||||||||
Sbjct  83200  AATAACGCCCTCTTGATAAAACAAGAGGGAAGCTATTGCTCCTTTTTTTTATTTAGTACT  83259

Query  181    ATTTGCCTTACATAGTTTCTTTAAAAATAACAAGTACTTTTTATAGTTTGGTTCGatttg  240
              ||||||||||||||||||||||||||||||||||||||||||||||||||||||||||||
Sbjct  83260  ATTTGCCTTACATAGTTTCTTTAAAAATAACAAGTACTTTTTATAGTTTGGTTCGATTTG  83319

Query  241    cgtgtttttctttgtattcatttatattataggtttctatatcatttattttcccaat  300
              ||||||||||||||||||||||||||||||||||||||||||||| ||||||||||||||
Sbjct  83320  CGTGTTTTTCTTTGTATTCATTTATATTATAGGTTTCTATATCATATATTTTTCCCAAT  83379

Query  301    cttttatgaagttttatttaCAATTTAAATTTCAATCGaaaaaaagagaaaaaatgacaa  360
              ||||||||||||||||||||||||| |||||||||||||||| | |||||||| ||||||
Sbjct  83380  CTTTTATGAAGTTTATTTACAATTT-AATTTCAATCGAAAATA-GAGAAAAA-TGACAA  83436

Query  361    attttgctttttgattttatattgattaaaatcaaaaataaagaaaagaattttgcttt  420
               ||||||||||| || ||||||||||||||| |||||| ||| |||| |||| | |||||
Sbjct  83437  -TTTTGCTTTTT-GA-TTTATATTGATTAAA-TCAAAA-TAA-GAAA-GAATAT-GCTTT  83488

Query  421    tttttATTGTTAAATGGaaaaaaaTATAGTTAATACTAGATAATCCGTAGAAGGGGGCGG  480
              |||| || ||||| ||||||||  ||||| ||||||||||||||| ||||| |||| |||
Sbjct  83489  TTTT-AT-GTTAA-TGGAAAAA--TATAG-TAATACTAGATAATC-GTAGA-GGGG-CGG  83539

Query  481    GATGTAGGCCAAAGTGGATCAAGG  504
               |||||| |||| |||||||||||
Sbjct  83540  -ATGTAG-CCAA-GTGGATCAAGG  83560
```

>NC_034322.1 Echinacea laevigata plastid, complete genome
 KX548219.1 Echinacea laevigata plastid, complete genome
Length=151886

```
 Score = 750 bits (406),  Expect = 0.0
 Identities = 476/505 (94%), Gaps = 23/505 (5%)
 Strand=Plus/Minus

Query  2    GTTATGCATGAACGTAATGCTCATAATTTCCCTCTAGACTTAGCTGCTATTGAAGCTCCA  61
            ||||||||||||||||||||||||||||||||||||||||||||||||||||||||||||
Sbjct  537  GTTATGCATGAACGTAATGCTCATAATTTCCCTCTAGACTTAGCTGCTATTGAAGCTCCA  478
```

```
Query  62   TCTACAAATGGATAAGCCTTTGGTCTGATTGTATAGGAGTTTTTGAACTAAAAAAGGAGC  121
            ||||||||||||||||||||||||||||||||||||||||||||||||||||||||||||
Sbjct  477  TCTACAAATGGATAAGCCTTTGGTCTGATTGTATAGGAGTTTTTGAACTAAAAAAGGAGC  418

Query  122  AATAACGCCCTCTTGATAAAACAAGAGGGAAGCTATTGCTCCtttttttATTTAGTACTA  181
            ||||||||||||||||||||||||||||||||||||||||||||||||||||||||||||
Sbjct  417  AATAACGCCCTCTTGATAAAACAAGAGGGAAGCTATTGCTCCTTTTTTTATTTAGTACTA  358

Query  182  TTTGCCTTACATAGTTTCTTTAAAAATAACAAGTACTTTTTATAGTTTGGTTCGatttgc  241
            ||||||||||||||||||||||||||||| ||||||||||||||||||||||||||||||
Sbjct  357  TTTGCCTTACATAGTTTCTTTAAAAATAATAAGTACTTTTTATAGTTTGGTTCGATTTGC  298

Query  242  gtgttttttctttgtattcatttatattataggtttctatatcatttatttttcccaatc  301
            ||||||||||||||||||||||||||||||||||||||||||||| ||||||||||||||
Sbjct  297  GTGTTTTTTCTTTGTATTCATTTATATTATAGGTTTCTATATCATATATTTTTCCCAATC  238

Query  302  ttttatgaagttttatttaCAATTTAAATTTCAATCGaaaaaaagagaaaaaatgacaaa  361
            ||||||||||||||||||||||||||| ||||||||||||| | || ||||| |||||| 
Sbjct  237  TTTTATGAAGTTTTATTTACAATTTAA-TTTCAATCGAAAATA-GATAAAAA-TGACAA-  182

Query  362  ttttgctttttttgattttatattgattaaaatcaaaaataa-aga-aaagaattttgctt  419
            ||||||||||| || ||||||||||||||| | |||||||| | | ||||||| | ||||
Sbjct  181  TTTTGCTTTTT-GA-TTTATATTGATTAAA-T-AAAAATAAGAAAGAAAGAATAT-GCTT  127

Query  420  ttttttATTGTTAAATGGaaaaaaaTATAGTTAATACTAGATAATCCGTAGAAGGGGGCG  479
            ||||| || ||||| ||||||||  ||||| ||||||||||||||| ||||| |||| ||
Sbjct  126  TTTTT-AT-GTTAA-TGGAAAAA--TATAG-TAATACTAGATAATC-GTAGA-GGGG-CG  76

Query  480  GGATGTAGGCCAAAGTGGATCAAGG  504
            | |||||| |||| |||||||||||
Sbjct  75   G-ATGTAG-CCAA-GTGGATCAAGG  54


>MK055334.1 Echinacea purpurea plastid, complete genome
Length=151948

 Score = 750 bits (406),  Expect = 0.0
 Identities = 477/506 (94%), Gaps = 26/506 (5%)
 Strand=Plus/Minus

Query  2    GTTATGCATGAACGTAATGCTCATAATTTCCCTCTAGACTTAGCTGCTATTGAAGCTCCA  61
            ||||||||||||||||||||||||||||||||||||||||||||||||||||||||||||
Sbjct  536  GTTATGCATGAACGTAATGCTCATAATTTCCCTCTAGACTTAGCTGCTATTGAAGCTCCA  477

Query  62   TCTACAAATGGATAAGCCTTTGGTCTGATTGTATAGGAGTTTTTGAACTAAAAAAGGAGC  121
            ||||||||||||||||||||||||||||||||||||||||||||||||||||||||||||
Sbjct  476  TCTACAAATGGATAAGCCTTTGGTCTGATTGTATAGGAGTTTTTGAACTAAAAAAGGAGC  417

Query  122  AATAACGCCCTCTTGATAAAACAAGAGGGAAGCTATTGCTCCttttttt--ATTTAGTAC  179
            |||||||||||||||||||||||||||||||||||||||||||||||||  |||||||||
Sbjct  416  AATAACGCCCTCTTGATAAAACAAGAGGGAAGCTATTGCTCCTTTTTTTTTATTTAGTAC  357

Query  180  TATTTGCCTTACATAGTTTCTTTAAAAATAACAAGTACTTTTTATAGTTTGGTTCGattt  239
            ||||||||||||||||||||||||||||||||||||||||||||||||||||||||||||
Sbjct  356  TATTTGCCTTACATAGTTTCTTTAAAAATAACAAGTACTTTTTATAGTTTGGTTCGATTT  297
```

```
Query  240  gcgtgtttttt-ctttgtattcatttatattataggtttctatatcatttatttttccca  298
            ||||||||||| ||||||||||||||||||||||||||||||||||||| ||||||||||
Sbjct  296  GCGTGTTTTTTTCTTTGTATTCATTTATATTATAGGTTTCTATATCATATATTTTTCCCA  237

Query  299  atcttttatgaagttttatttaCAATTTAAATTTCAATCGaaaaaaagagaaaaaatgac  358
            |||||||||||||||||||||||||||||| ||||||||||||| | |||||||| ||||
Sbjct  236  ATCTTTTATGAAGTTTTATTTACAATTTAA-TTTCAATCGAAAATA-GAGAAAAA-TGAC  180

Query  359  aaattttgcttttttgattttatattgattaaaatcaaaaataaagaaaagaattttgct  418
            || ||||||||||| || ||||||||||||||| |||||| ||| |||| |||| | |||
Sbjct  179  AA-TTTTGCTTTTT-GA-TTTATATTGATTAAA-TCAAAA-TAA-GAAA-GAATAT-GCT  128

Query  419  tttttttATTGTTAAATGGaaaaaaaTATAGTTAATACTAGATAATCCGTAGAAGGGGGC  478
            |||||| || ||||| ||||||||  ||||| ||||||||||||||| ||||| |||| |
Sbjct  127  TTTTTT-AT-GTTAA-TGGAAAAA--TATAG-TAATACTAGATAATC-GTAGA-GGGG-C  77

Query  479  GGGATGTAGGCCAAAGTGGATCAAGG  504
            || |||||| |||| |||||||||||
Sbjct  76   GG-ATGTAG-CCAA-GTGGATCAAGG  54


>NC_034324.1 Echinacea angustifolia plastid, complete genome
 KX548221.1 Echinacea angustifolia plastid, complete genome
Length=151935

 Score = 747 bits (404),  Expect = 0.0
 Identities = 474/503 (94%), Gaps = 24/503 (5%)
 Strand=Plus/Minus

Query  2    GTTATGCATGAACGTAATGCTCATAATTTCCCTCTAGACTTAGCTGCTATTGAAGCTCCA  61
            ||||||||||||||||||||||||||||||||||||||||||||||||||||||||||||
Sbjct  532  GTTATGCATGAACGTAATGCTCATAATTTCCCTCTAGACTTAGCTGCTATTGAAGCTCCA  473

Query  62   TCTACAAATGGATAAGCCTTTGGTCTGATTGTATAGGAGTTTTTGAACTAAAAAAGGAGC  121
            ||||||||||||||||||||||||||||||||||||||||||||||||||||||||||||
Sbjct  472  TCTACAAATGGATAAGCCTTTGGTCTGATTGTATAGGAGTTTTTGAACTAAAAAAGGAGC  413

Query  122  AATAACGCCCTCTTGATAAAACAAGAGGGAAGCTATTGCTCCtttttttATTTAGTACTA  181
            ||||| |||||||||||||||||||||| |||||||||||||||||||||||||||||||
Sbjct  412  AATAATGCCCTCTTGATAAAACAAGAGGAAAGCTATTGCTCCTTTTTTTATTTAGTACTA  353

Query  182  TTTGCCTTACATAGTTTCTTTAAAAATAACAAGTACTTTTTATAGTTTGGTTCGatttgc  241
            ||||||||||||||||||||||||||||||||||||||||||||||||||||||||||||
Sbjct  352  TTTGCCTTACATAGTTTCTTTAAAAATAACAAGTACTTTTTATAGTTTGGTTCGATTTGC  293

Query  242  gtgttttttctttgtattcatttatattataggtttctatatcatttatttttcccaatc  301
            |||||||| |||||||||||||||||||||||||||||||||||| ||||||||||||||
Sbjct  292  GTGTTTTT-CTTTGTATTCATTTATATTATAGGTTTCTATATCATATATTTTTCCCAATC  234

Query  302  ttttatgaagttttatttaCAATTTAAATTTCAATCGaaaaaaagagaaaaaatgacaaa  361
            ||||||||||||||||||||||||||| ||||||||||||| | |||||||| ||||||
Sbjct  233  TTTTATGAAGTTTTATTTACAATTTAA-TTTCAATCGAAAATA-GAGAAAAA-TGACAA-  178

Query  362  ttttgcttttttgattttatattgattaaaatcaaaaataaagaaaagaattttgctttt  421
            ||||||||||| || ||||||||||||||| |||||| ||| |||| |||| | ||||||
Sbjct  177  TTTTGCTTTTT-GA-TTTATATTGATTAAA-TCAAAA-TAA-GAAA-GAATAT-GCTTTT  125
```

```
Query  422   ttttATTGTTAAATGGaaaaaaaTATAGTTAATACTAGATAATCCGTAGAAGGGGGCGGG  481
             ||| || ||||| ||||||||  ||||| ||||||||||||||| ||||| |||| |||
Sbjct  124   TTT-AT-GTTAA-TGGAAAAA--TATAG-TAATACTAGATAATC-GTAGA-GGGG-CGG-  75

Query  482   ATGTAGGCCAAAGTGGATCAAGG  504
             |||||| |||| |||||||||||
Sbjct  74    ATGTAG-CCAA-GTGGATCAAGG  54


>MF348644.1 Echinacea pallida isolate AD3HJ47 photosystem II protein D1 (psbA)
gene, partial cds; and psbA-trnH intergenic spacer, partial
sequence; chloroplast
Length=466

 Score = 715 bits (387),  Expect = 0.0
 Identities = 455/483 (94%), Gaps = 23/483 (5%)
 Strand=Plus/Plus

Query  24    ATAATTTCCCTCTAGACTTAGCTGCTATTGAAGCTCCATCTACAAATGGATAAGCCTTTG  83
             ||||||||||||||||||||||||||||||||||||||||||||||||||||||||||||
Sbjct  1     ATAATTTCCCTCTAGACTTAGCTGCTATTGAAGCTCCATCTACAAATGGATAAGCCTTTG  60

Query  84    GTCTGATTGTATAGGAGTTTTTGAACTAAAAAAGGAGCAATAACGCCCTCTTGATAAAAC  143
             ||||||||||||||||||||||||||||||||||||||||||||||||||||||||||||
Sbjct  61    GTCTGATTGTATAGGAGTTTTTGAACTAAAAAAGGAGCAATAACGCCCTCTTGATAAAAC  120

Query  144   AAGAGGGAAGCTATTGCTCCtttttttATTTAGTACTATTTGCCTTACATAGTTTCTTTA  203
             ||||||||||||||||||||||||||||||||||||||||||||||||||||||||||||
Sbjct  121   AAGAGGGAAGCTATTGCTCCTTTTTTTATTTAGTACTATTTGCCTTACATAGTTTCTTTA  180

Query  204   AAAATAACAAGTACTTTTTATAGTTTGGTTCGatttgcgtgtttttttctttgtattcatt  263
             ||||||| ||||||||||||||||||||||||||||||||||||||||||||||||||||
Sbjct  181   AAAATAATAAGTACTTTTTATAGTTTGGTTCGATTTGCGTGTTTTTTCTTTGTATTCATT  240

Query  264   tatattataggtttctatatcatttattttttcccaatcttttatgaagttttatttaCAA  323
             ||||||||||||||||||||||| ||||||||||||||||||||||||||||||||||||
Sbjct  241   TATATTATAGGTTTCTATATCATATATTTTTCCCAATCTTTTATGAAGTTTTATTTACAA  300

Query  324   TTTAAATTTCAATCGaaaaaaagagaaaaaatgacaaattttgcttttttgattttatat  383
             ||| ||||||||||||||| | |||||||| |||||| ||||||||||| || |||||||
Sbjct  301   TTT-AATTTCAATCGAAAATA-GAGAAAAA-TGACAA-TTTTGCTTTTT-GA-TTTATAT  354

Query  384   tgattaaaatcaaaaataa-aga-aaagaattttgcttttttttATTGTTAAATGGaaaa  441
             |||||||| | |||||||| | | ||||||| | ||||||||| || ||||| |||||||
Sbjct  355   TGATTAAA-T-AAAAATAAGAAAGAAAGAATAT-GCTTTTTTT-AT-GTTAA-TGGAAAA  408

Query  442   aaaTATAGTTAATACTAGATAATCCGTAGAAGGGGGCGGGATGTAGGCCAAAGTGGATCA  501
             |  ||||| ||||||||||||||| ||||| |||| ||| |||||| |||| ||||||||
Sbjct  409   A--TATAG-TAATACTAGATAATC-GTAGA-GGGG-CGG-ATGTAG-CCAA-GTGGATCA  459

Query  502   AGG  504
             |||
Sbjct  460   AGG  462


>MF348389.1 Echinacea pallida isolate AD0EE57 photosystem II protein D1 (psbA)
gene, partial cds; and psbA-trnH intergenic spacer, partial
```

```
sequence; chloroplast
Length=466

 Score = 715 bits (387),  Expect = 0.0
 Identities = 455/483 (94%), Gaps = 23/483 (5%)
 Strand=Plus/Plus

Query  24   ATAATTTCCCTCTAGACTTAGCTGCTATTGAAGCTCCATCTACAAATGGATAAGCCTTTG  83
            ||||||||||||||||||||||||||||||||||||||||||||||||||||||||||||
Sbjct  1    ATAATTTCCCTCTAGACTTAGCTGCTATTGAAGCTCCATCTACAAATGGATAAGCCTTTG  60

Query  84   GTCTGATTGTATAGGAGTTTTTGAACTAAAAAAGGAGCAATAACGCCCTCTTGATAAAAC  143
            ||||||||||||||||||||||||||||||||||||||||||||||||||||||||||||
Sbjct  61   GTCTGATTGTATAGGAGTTTTTGAACTAAAAAAGGAGCAATAACGCCCTCTTGATAAAAC  120

Query  144  AAGAGGGAAGCTATTGCTCCtttttttATTTAGTACTATTTGCCTTACATAGTTTCTTTA  203
            ||||||||||||||||||||||||||||||||||||||||||||||||||||||||||||
Sbjct  121  AAGAGGGAAGCTATTGCTCCTTTTTTTATTTAGTACTATTTGCCTTACATAGTTTCTTTA  180

Query  204  AAAATAACAAGTACTTTTTATAGTTTGGTTCGatttgcgtgtttttttctttgtattcatt  263
            ||||||| ||||||||||||||||||||||||||||||||||||||||||||||||||||
Sbjct  181  AAAATAATAAGTACTTTTTATAGTTTGGTTCGATTTGCGTGTTTTTTCTTTGTATTCATT  240

Query  264  tatattataggtttctatatcatttatttttcccaatcttttatgaagttttatttaCAA  323
            ||||||||||||||||||||||| ||||||||||||||||||||||||||||||||||||
Sbjct  241  TATATTATAGGTTTCTATATCATATATTTTTCCCAATCTTTTATGAAGTTTTATTTACAA  300

Query  324  TTTAAATTTCAATCGaaaaaaagagaaaaaatgacaaattttgctttttgattttatat  383
            ||| ||||||||||||||| | |||||||| |||||| ||||||||||| || |||||||
Sbjct  301  TTT-AATTTCAATCGAAAATA-GAGAAAAA-TGACAA-TTTTGCTTTTT-GA-TTTATAT  354

Query  384  tgattaaaatcaaaaataa-aga-aaagaattttgcttttttttATTGTTAAATGGaaaa  441
            |||||||| | |||||||| | | ||||||| | ||||||||| || ||||| |||||||
Sbjct  355  TGATTAAA-T-AAAAATAAGAAAGAAAGAATAT-GCTTTTTTT-AT-GTTAA-TGGAAAA  408

Query  442  aaaTATAGTTAATACTAGATAATCCGTAGAAGGGGGCGGGATGTAGGCCAAAGTGGATCA  501
            |  ||||| ||||||||||||||| ||||| |||| ||| |||||| |||| ||||||||
Sbjct  409  A--TATAG-TAATACTAGATAATC-GTAGA-GGGG-CGG-ATGTAG-CCAA-GTGGATCA  459

Query  502  AGG  504
            |||
Sbjct  460  AGG  462


>NC_034327.1 Echinacea purpurea plastid, complete genome
 KX548224.1 Echinacea purpurea plastid, complete genome
Length=151913

 Score = 706 bits (382),  Expect = 0.0
 Identities = 468/503 (93%), Gaps = 31/503 (6%)
 Strand=Plus/Minus

Query  2    GTTATGCATGAACGTAATGCTCATAATTTCCCTCTAGACTTAGCTGCTATTGAAGCTCCA  61
            ||||||||||||||||||||||||||||||||||||||||||||||||||||||||||||
Sbjct  525  GTTATGCATGAACGTAATGCTCATAATTTCCCTCTAGACTTAGCTGCTATTGAAGCTCCA  466

Query  62   TCTACAAATGGATAAGCCTTTGGTCTGATTGTATAGGAGTTTTTGAACTAAAAAAGGAGC  121
```

```
                ||||||||||||||||||||||||||||||||||||||||||||||||||||||||||||
Sbjct  465   TCTACAAATGGATAAGCCTTTGGTCTGATTGTATAGGAGTTTTTGAACTAAAAAAGGAGC  406

Query  122   AATAACGCCCTCTTGATAAAACAAGAGGGAAGCTATTGCTCCtttttttATTTAGTACTA  181
             ||||||||||||||||||||||||||||||||||||||||||||||||||||||||||||
Sbjct  405   AATAACGCCCTCTTGATAAAACAAGAGGGAAGCTATTGCTCCTTTTTTTATTTAGTACTA  346

Query  182   TTTGCCTTACATAGTTTCTTTAAAAATAACAAGTACTTTTTATAGTTTGGTTCGatttgc  241
             ||||||||||||||||||||||||||||||||||||||||||||||||||||||||||||
Sbjct  345   TTTGCCTTACATAGTTTCTTTAAAAATAACAAGTACTTTTTATAGTTTGGTTCGATTTGC  286

Query  242   gtgttttttctttgtattcatttatattataggtttctatatcatttatttttcccaatc  301
             ||||||||||||||||||||||||||||||||||||||||||||| ||||||||||||||
Sbjct  285   GTGTTTTTTCTTTGTATTCATTTATATTATAGGTTTCTATATCATATATTTTTCCCAATC  226

Query  302   ttttatgaagttttatttaCAATTTAAATTTCAATCGaaaaaaagagaaaaaatgacaaa  361
             ||||||||||||||||||||||||||| ||||||||||||| | |||||||| ||| ||
Sbjct  225   TTTTATGAAGTTTTATTTACAATTTAA-TTTCAATCGAAAATA-GAGAAAAA-TGAGAA-  170

Query  362   ttttgctttttttgattttatattgattaaaatcaaaaataaagaaaagaattttgctttt  421
             ||||||||||| || ||||||||||||||| |||||| ||| |||| |||| | ||||||
Sbjct  169   TTTTGCTTTTT-GA-TTTATATTGATTAAA-TCAAAA-TAA-GAAA-GAATAT-GCTTTT  117

Query  422   ttttATTGTTAAATGGaaaaaaTATAGTTAATACTAGATAATCCGTAGAAGGGGGCGGG  481
             ||| || ||||| ||||||||  |||||| | ||  |  |   | ||||| |||| |||
Sbjct  116   TTT-AT-GTTAA-TGGAAAAA--TATAGT-A-TA--A--T---C-GTAGA-GGGG-CGG-  75

Query  482   ATGTAGGCCAAAGTGGATCAAGG  504
             |||||| |||| |||||||||||
Sbjct  74    ATGTAG-CCAA-GTGGATCAAGG  54


>LS999859.1 Echinacea angustifolia DNA containing psbA-trnH IGS, specimen
voucher FEM_DBE_015
Length=437

 Score = 667 bits (361),  Expect = 0.0
 Identities = 427/454 (94%), Gaps = 24/454 (5%)
 Strand=Plus/Plus

Query  51    TTGAAGCTCCATCTACAAATGGATAAGCCTTTGGTCTGATTGTATAGGAGTTTTTGAACT  110
             ||||||||||||||||||||||||||||||||||||||||||||||||||||||||||||
Sbjct  1     TTGAAGCTCCATCTACAAATGGATAAGCCTTTGGTCTGATTGTATAGGAGTTTTTGAACT  60

Query  111   AAAAAAGGAGCAATAACGCCCTCTTGATAAAACAAGAGGGAAGCTATTGCTCCttttttt  170
             ||||||||||||||||||||||||||||||||||||||||||||||||||||||||||||
Sbjct  61    AAAAAAGGAGCAATAACGCCCTCTTGATAAAACAAGAGGGAAGCTATTGCTCCTTTTTTT  120

Query  171   ATTTAGTACTATTTGCCTTACATAGTTTCTTTAAAAATAACAAGTACTTTTTATAGTTTG  230
             ||||||||||||||||||||||||||||||||||||||||||||||||||||||||||||
Sbjct  121   ATTTAGTACTATTTGCCTTACATAGTTTCTTTAAAAATAACAAGTACTTTTTATAGTTTG  180

Query  231   GTTCGatttgcgtgttttttctttgtattcatttatattataggtttctatatcatttat  290
             |||||||||||||||||||||||||||||||||||||||||||||||||||||||| |||
Sbjct  181   GTTCGATTTGCGTGTTTTTTCTTTGTATTCATTTATATTATAGGTTTCTATATCATATAT  240

Query  291   ttttcccaatcttttatgaagttttatttaCAATTTAAATTTCAATCGaaaaaaagagaa  350
```

```
                ||||||||||||||||||||||||||||||||||| ||||||||||||||| | |||||
Sbjct  241   TTTTCCCAATCTTTTATGAAGTTTATTTACAATTT-AATTTCAATCGAAAATA-GAGAA  298

Query  351   aaaatgacaaattttgctttttttgattttatattgattaaaatcaaaaataaagaaaaga  410
             ||| |||| |||||||||||||| | ||||||||||||||| |||||| ||| |||| ||
Sbjct  299   AAA-TGAC-AATTTTGCTTTTTT-A-TTTATATTGATTAAA-TCAAAA-TAA-GAAA-GA  350

Query  411   atttgcttttttttATTGTTAAATGGaaaaaaaTATAGTTAATACTAGATAATCCGTAG  470
             || | ||||||||  || ||||| ||||||||  ||||| ||||||||||||||| ||||
Sbjct  351   ATAT-GCTTTTTT--AT-GTTAA-TGGAAAAA--TATAG-TAATACTAGATAATC-GTAG  401

Query  471   AAGGGGGCGGGATGTAGGCCAAAGTGGATCAAGG  504
             | |||| ||| |||||| |||| |||||||||||
Sbjct  402   A-GGGG-CGG-ATGTAG-CCAA-GTGGATCAAGG  430


>MF037494.1 Echinacea sanguinea voucher Bengtson9 trnH-psbA intergenic spacer
region, partial sequence; chloroplast
Length=407

 Score = 664 bits (359),  Expect = 0.0
 Identities = 403/421 (96%), Gaps = 16/421 (4%)
 Strand=Plus/Plus

Query  45    CTGCTATTGAAGCTCCATCTACAAATGGATAAGCCTTTGGTCTGATTGTATAGGAGTTTT  104
             ||||||||||||||||||||||||||||||||||||||||||||||||||||||||||||
Sbjct  1     CTGCTATTGAAGCTCCATCTACAAATGGATAAGCCTTTGGTCTGATTGTATAGGAGTTTT  60

Query  105   TGAACTAAAAAAGGAGCAATAACGCCCTCTTGATAAAACAAGAGGGAAGCTATTGCTCCt  164
             ||||||||||||||||||||||||||||||||||||||||||||||||||||||||||||
Sbjct  61    TGAACTAAAAAAGGAGCAATAACGCCCTCTTGATAAAACAAGAGGGAAGCTATTGCTCCT  120

Query  165   ttttttATTTAGTACTATTTGCCTTACATAGTTTCTTTAAAAATAACAAGTACTTTTTAT  224
             ||||||||||||||||||||||||||||||||||||||||||||||||||||||||||||
Sbjct  121   TTTTTTATTTAGTACTATTTGCCTTACATAGTTTCTTTAAAAATAACAAGTACTTTTTAT  180

Query  225   AGTTTGGTTCGatttgcgtgtttttctttgtattcatttatattataggtttctatatc  284
             ||||||||||||||||||||||||||||||||||||||||||||||||||||||||||||
Sbjct  181   AGTTTGGTTCGATTTGCGTGTTTTTCTTTGTATTCATTTATATTATAGGTTTCTATATC  240

Query  285   atttattttcccaatcttttatgaagttttatttaCAATTTAAATTTCAATCGaaaaaa  344
             |||||||||||||||||||||||||||||||||||||||| ||||||||||||||| |
Sbjct  241   ATTTATTTTCCCAATCTTTTATGAAGTTTATTTACAATTT-AATTTCAATCGAAAATA  299

Query  345   agagaaaaaatgacaaattttgctttttttgattttatattgattaaaatcaaaaataaag  404
              |||||||| |||||| ||||||||||| || ||||||||||||||| |||||| ||| |
Sbjct  300   -GAGAAAAA-TGACAA-TTTTGCTTTTT-GA-TTTATATTGATTAAA-TCAAAA-TAA-G  351

Query  405   aaaagaattttgctttttttttATTGTTAAATGGaaaaaaaTATAGTTAATACTAGATAAT  464
             ||| |||| | ||||||||| || ||||| ||||||||| ||||| ||||||||||||||
Sbjct  352   AAA-GAATAT-GCTTTTTTT-AT-GTTAA-TGGAAAAAA-TATAG-TAATACTAGATAAT  404

Query  465   C  465
             |
Sbjct  405   C  405
```

```
>LS999860.1 Echinacea pallida DNA containing psbA-trnH IGS, specimen voucher
FEM_DBE_018
Length=384

 Score = 662 bits (358),  Expect = 0.0
 Identities = 383/393 (97%), Gaps = 9/393 (2%)
 Strand=Plus/Plus

Query  15   GTAATGCTCATAATTTCCCTCTAGACTTAGCTGCTATTGAAGCTCCATCTACAAATGGAT  74
            ||||||||||||||||||||||||||||||||||||||||||||||||||||||||||||
Sbjct  1    GTAATGCTCATAATTTCCCTCTAGACTTAGCTGCTATTGAAGCTCCATCTACAAATGGAT  60

Query  75   AAGCCTTTGGTCTGATTGTATAGGAGTTTTTGAACTAAAAAAGGAGCAATAACGCCCTCT  134
            ||||||||||||||||||||||||||||||||||||||||||||||||||||||||||||
Sbjct  61   AAGCCTTTGGTCTGATTGTATAGGAGTTTTTGAACTAAAAAAGGAGCAATAACGCCCTCT  120

Query  135  TGATAAAACAAGAGGGAAGCTATTGCTCCtttttttATTTAGTACTATTTGCCTTACATA  194
            ||||||||||||||||||||||||||||||||||||||||||||||||||||||||||||
Sbjct  121  TGATAAAACAAGAGGGAAGCTATTGCTCCTTTTTTTATTTAGTACTATTTGCCTTACATA  180

Query  195  GTTTCTTTAAAAATAACAAGTACTTTTTATAGTTTGGTTCGatttgcgtgtttttttcttt  254
            ||||||||||||||||||||||||||||||||||||||||||||||||||||||||||||
Sbjct  181  GTTTCTTTAAAAATAACAAGTACTTTTTATAGTTTGGTTCGATTTGCGTGTTTTTTCTTT  240

Query  255  gtattcatttatattataggtttctatatcatttatttttcccaatcttttatgaagttt  314
            ||||||||||||||||||||||||||||||||||||||||||||||||||||||||||||
Sbjct  241  GTATTCATTTATATTATAGGTTTCTATATCATTTATTTTTCCCAATCTTTTATGAAGTTT  300

Query  315  tatttaCAATTTAAATTTCAATCGaaaaaaagagaaaaaatgacaaatttgctttttttg  374
            |||||||||||| |||||||||||||| | |||||||| |||||| ||||||||||| |
Sbjct  301  TATTTACAATTT-AATTTCAATCGAAAATA-GAGAAAAA-TGACAA-TTTTGCTTTTT-G  355

Query  375  atttatattgattaaaatcaaaaataaagaaa  407
            | ||||||||||||||| |||||| ||| ||||
Sbjct  356  A-TTTATATTGATTAAA-TCAAAA-TAA-GAAA  384


>NC_081940.1 Enydra sessilis chloroplast, complete genome
 OR069728.1 Enydra sessilis chloroplast, complete genome
Length=151340

 Score = 654 bits (354),  Expect = 0.0
 Identities = 460/506 (91%), Gaps = 28/506 (6%)
 Strand=Plus/Minus

Query  2    GTTATGCATGAACGTAATGCTCATAATTTCCCTCTAGACTTAGCTGCTATTGAAGCTCCA  61
            ||||||||||||||||||||||||||||||||||||||||||||||||||||||||||||
Sbjct  548  GTTATGCATGAACGTAATGCTCATAATTTCCCTCTAGACTTAGCTGCTATTGAAGCTCCA  489

Query  62   TCTACAAATGGATAAGCCTTTGGTCTGATTGTATAGGAGTTTTTGAACTAAAAAAGGAGC  121
            |||||||||||||||| |||||||||||||||||||||||||||||||||||||||||||
Sbjct  488  TCTACAAATGGATAAGACTTTGGTCTGATTGTATAGGAGTTTTTGAACTAAAAAAGGAGC  429

Query  122  AATAACGCCCTCTTGATAAAACAAGAGGGAAGCTATTGCTCCttttttt-ATTTAGTACT  180
            ||||||||||||||||||||||||||||||||||||||||||||||||| ||||||||||
Sbjct  428  AATAACGCCCTCTTGATAAAACAAGAGGGAAGCTATTGCTCCTTTTTTTTATTTAGTACT  369
```

```
Query  181   ATTTGCCTTACATAGTTTCTTTAAAAATAACAAGTACTTTTTATAGTTTGGTTCGatttg  240
             |||||||||||||||||||||||||||||||||| ||||||| |||||||||||||||||
Sbjct  368   ATTTGCCTTACATAGTTTCTTTAAAAATAACAAGGACTTTTTCTAGTTTGGTTCGATTTG  309

Query  241   cgtgtttttctttgtattcatttatattataggtttctatatcatttatttttccc-aa  299
             |||||||| |||||||||||||||||||||||||||||||||||||||| ||||||| ||
Sbjct  308   CGTGTTTTATCTTTGTATTCATTTATATTATAGGTTTCTATATCATTTACTTTTCCCCAA  249

Query  300   tcttt-tatgaagttttatttaCAATTTAAATTTCAATCGaaaaaaagagaaaaaatgac  358
             | ||| ||||||||||||||| ||||| || ||||||||||||| | || ||||| |||
Sbjct  248   TATTTGTATGAAGTTTTATTTCCAATTCAA-TTTCAATCGAAAATA-GATAAAAA-TGA-  193

Query  359   aaattttgcttttttgattttatattgattaaaatcaaaaataaagaaaagaattttgct  418
             ||||||||||| ||| |||   | |||||| |  | ||||| |  |||| ||||  ||||
Sbjct  192   AAATTTTGCTTATTT-ATTAC-T-TTGATTTAT-T-AAAAA-A--GAAA-GAATA-TGCT  143

Query  419   tttttttATTGTTAAATGGaaaaaaaTATAGTTAATACTAGATAATCCGTAGAAGGGGGC  478
             ||||||||| ||||| ||||||||  ||||| |||||||||| |||  ||||| |||| |
Sbjct  142   TTTTTTTAT-GTTAA-TGGAAAAA--TATAG-TAATACTAGAGAATA-GTAGA-GGGG-C  91

Query  479   GGGATGTAGGCCAAAGTGGATCAAGG  504
             || |||||| |||| |||||||||||
Sbjct  90    GG-ATGTAG-CCAA-GTGGATCAAGG  68
```

>MF037491.1 Echinacea atrorubens voucher Barnard1532 trnH-psbA intergenic spacer region, partial sequence; chloroplast
Length=406

```
 Score = 651 bits (352),  Expect = 0.0
 Identities = 401/421 (95%), Gaps = 17/421 (4%)
 Strand=Plus/Plus

Query  45    CTGCTATTGAAGCTCCATCTACAAATGGATAAGCCTTTGGTCTGATTGTATAGGAGTTTT  104
             ||||||||||||||||||||||||||||||||||||||||||||||||||||||||||||
Sbjct  1     CTGCTATTGAAGCTCCATCTACAAATGGATAAGCCTTTGGTCTGATTGTATAGGAGTTTT  60

Query  105   TGAACTAAAAAAGGAGCAATAACGCCCTCTTGATAAAACAAGAGGGAAGCTATTGCTCCt  164
             |||||||||||||||||||||||| |||||||||||||||||||||||||||||||||||
Sbjct  61    TGAACTAAAAAAGGAGCAATAACGTCCTCTTGATAAAACAAGAGGGAAGCTATTGCTCCT  120

Query  165   tttttATTTAGTACTATTTGCCTTACATAGTTTCTTTAAAAATAACAAGTACTTTTTAT  224
             ||||||||||||||||||||||||||||||||||||||||||||||||||||||||||||
Sbjct  121   TTTTTATTTAGTACTATTTGCCTTACATAGTTTCTTTAAAAATAACAAGTACTTTTTAT  180

Query  225   AGTTTGGTTCGatttgcgtgtttttctttgtattcatttatattataggtttctatatc  284
             ||||||||||||||||||||||||||||||||||||||||||||||||||||||||||||
Sbjct  181   AGTTTGGTTCGATTTGCGTGTTTTTCTTTGTATTCATTTATATTATAGGTTTCTATATC  240

Query  285   atttatttttcccaatcttttatgaagttttatttaCAATTTAAATTTCAATCGaaaaaa  344
             |||||||||||||||||||||||||||||||||||||||||| ||||||||||||||| |
Sbjct  241   ATTTATTTTTCCCAATCTTTTATGAAGTTTTATTTACAATTT-AATTTCAATCGAAAATA  299

Query  345   agagaaaaaatgacaaattttgctttttgattttatattgattaaaatcaaaaataaag  404
              |||||||| |||||| ||||||||||| || ||||||||||||||| |||||| ||| |
Sbjct  300   -GAGAAAAA-TGACAA-TTTTGCTTTTT-GA-TTTATATTGATTAAA-TCAAAA-TAA-G  351
```

```
Query  405   aaaagaattttgcttttttttATTGTTAAATGGaaaaaaaTATAGTTAATACTAGATAAT  464
             ||| |||| | ||||||||  || ||||| ||||||||| ||||| ||||||||||||||
Sbjct  352   AAA-GAATAT-GCTTTTTT--AT-GTTAA-TGGAAAAAA-TATAG-TAATACTAGATAAT  403

Query  465   C  465
             |
Sbjct  404   C  404
```

>MF037490.1 Echinacea angustifolia voucher Rolfsmeier19250 trnH-psbA intergenic spacer region, partial sequence; chloroplast
Length=409

```
 Score = 649 bits (351),  Expect = 0.0
 Identities = 401/422 (95%), Gaps = 16/422 (4%)
 Strand=Plus/Plus

Query  45    CTGCTATTGAAGCTCCATCTACAAATGGATAAGCCTTTGGTCTGATTGTATAGGAGTTTT  104
             ||||||||||||||||||||||||||||||||||||||||||||||||||||||||||||
Sbjct  1     CTGCTATTGAAGCTCCATCTACAAATGGATAAGCCTTTGGTCTGATTGTATAGGAGTTTT  60

Query  105   TGAACTAAAAAAGGAGCAATAACGCCCTCTTGATAAAACAAGAGGGAAGCTATTGCTCCt  164
             ||||||||||||||||||||||||||||||||||||||||||||||||||||||||||||
Sbjct  61    TGAACTAAAAAAGGAGCAATAACGCCCTCTTGATAAAACAAGAGGGAAGCTATTGCTCCT  120

Query  165   tttttATTTAGTACTATTTGCCTTACATAGTTTCTTTAAAAATAACAAGTACTTTTTAT  224
             ||||||||||||||||||||||||||||||||||||||||||||||||||||||||||||
Sbjct  121   TTTTTTATTTAGTACTATTTGCCTTACATAGTTTCTTTAAAAATAACAAGTACTTTTTAT  180

Query  225   AGTTTGGTTCGatttgcgtgtttttttctttgtattcatttatattataggtttctatatc  284
             ||||||||||||||||||||||||||||||||||||||||||||||||||||||||||||
Sbjct  181   AGTTTGGTTCGATTTGCGTGTTTTTTCTTTGTATTCATTTATATTATAGGTTTCTATATC  240

Query  285   atttatttttcccaatcttttatgaagttttatttaC-AATTTAAATTTCAATCGaaaaa  343
             || |||||||||||||||||||||||||||||||||| ||||| |||||||||||||||
Sbjct  241   ATATATTTTTCCCAATCTTTTATGAAGTTTTATTTACGAATTT-AATTTCAATCGAAAAT  299

Query  344   aagagaaaaaatgacaaattttgctttttttgattttatattgattaaaatcaaaaataaa  403
             | ||| ||||||||| |||||||||||||| | ||||||||||||||| ||||||||||
Sbjct  300   A-GAG-AAAAATGACGAATTTTGCTTTTTT-A-TTTATATTGATTAAA-TCAAAAATAA-  353

Query  404   gaaaagaattttgcttttttttATTGTTAAATGGaaaaaaaTATAGTTAATACTAGATAA  463
             ||||||||| | ||||||||  || || || ||||||||  ||||| |||||||||||||
Sbjct  354   GAAAAGAATAT-GCTTTTTT--AT-GTGAA-TGGAAAAA--TATAG-TAATACTAGATAA  405

Query  464   TC  465
             ||
Sbjct  406   TC  407
```

>MF348385.1 Echinacea purpurea isolate AD0EG44 photosystem II protein D1 (psbA) gene, partial cds; and psbA-trnH intergenic spacer, partial sequence; chloroplast
Length=485

```
 Score = 645 bits (349),  Expect = 2e-180
 Identities = 456/500 (91%), Gaps = 38/500 (8%)
```

```
 Strand=Plus/Plus

Query  24   ATAATTTCCCTCTAGACTTAGCTGCTATTGAAGCTCCATCTACAAATGGATAAGCCTTTG  83
            ||||||||||||||||||||||||||||||||||||||||||||||||||||||||||||
Sbjct  1    ATAATTTCCCTCTAGACTTAGCTGCTATTGAAGCTCCATCTACAAATGGATAAGCCTTTG  60

Query  84   GTCTGATTGTATAGGAGTTTTTGAACTAAAAAAGGAGCAATAACGCCCTCTTGATAAAAC  143
            ||||||||||||||||||||||||||||||||||||||||||||||||||||||||||||
Sbjct  61   GTCTGATTGTATAGGAGTTTTTGAACTAAAAAAGGAGCAATAACGCCCTCTTGATAAAAC  120

Query  144  AAGAGGGAAGCTATTGCTCC-tttttttATTTAGTACTATTTGCCTTACATAGTTTCTTT  202
            |||||||||||||||||||| |||||||||||||||||||||||||||||||||||||||
Sbjct  121  AAGAGGGAAGCTATTGCTCCTTTTTTTTATTTAGTACTATTTGCCTTACATAGTTTCTTT  180

Query  203  AAAAATAACAAGTACTTTTTATAGTTTGGTTCGatttgcgtgtttttttctttgtattcat  262
            ||||||||||||||||||||||||||||||||||||||||||||||||||||||||||||
Sbjct  181  AAAAATAACAAGTACTTTTTATAGTTTGGTTCGATTTGCGTGTTTTTTCTTTGTATTCAT  240

Query  263  ttatattataggtttctatatcatttattttcccaatcttttatgaagttttatttaCA  322
            |||||||||||||||||||||||| |||||||||||||||||||||||||||||||||||
Sbjct  241  TTATATTATAGGTTTCTATATCATATATTTTCCCAATCTTTTATGAAGTTTTATTTACA  300

Query  323  ATTTAAATTTCAATCGaaaaaaagagaaaaaatgacaaattttgctt-ttttgattttat  381
            |||| ||||||||||||||| | |||||||| |||||| |||  ||| |||||| |||||
Sbjct  301  ATTT-AATTTCAATCGAAAATA-GAGAAAAA-TGACAA-TTT--CTTATTTTGA-TTTAT  353

Query  382  attgattaaa--a---tca-aaa---at-aaag-aaaa---gaatt--t-tgcttttttt  424
            ||||||||||  |   | | |||   || |||  ||||   |||    | ||||||||||
Sbjct  354  ATTGATTAAATCAAAATAAGAAATTGATTAAATCAAAATAAGAAAGAATATGCTTTTTTT  413

Query  425  tATTGTTAAATGGaaaaaaaTATAGTTAATACTAGATAATCCGTAGAAGGGGGCGGGATG  484
             || ||||| ||||||||  ||||| ||||||||||||||| ||||| |||| ||| |||
Sbjct  414  -AT-GTTAA-TGGAAAAA--TATAG-TAATACTAGATAATC-GTAGA-GGGG-CGG-ATG  463

Query  485  TAGGCCAAAGTGGATCAAGG  504
            ||| |||| |||||||||||
Sbjct  464  TAG-CCAA-GTGGATCAAGG  481


>MF348589.1 Echinacea purpurea isolate AD3HL50 photosystem II protein D1
(psbA) gene, partial cds; and psbA-trnH intergenic spacer, partial
sequence; chloroplast
Length=485

 Score = 645 bits (349),  Expect = 2e-180
 Identities = 456/500 (91%), Gaps = 38/500 (8%)
 Strand=Plus/Plus

Query  24   ATAATTTCCCTCTAGACTTAGCTGCTATTGAAGCTCCATCTACAAATGGATAAGCCTTTG  83
            ||||||||||||||||||||||||||||||||||||||||||||||||||||||||||||
Sbjct  1    ATAATTTCCCTCTAGACTTAGCTGCTATTGAAGCTCCATCTACAAATGGATAAGCCTTTG  60

Query  84   GTCTGATTGTATAGGAGTTTTTGAACTAAAAAAGGAGCAATAACGCCCTCTTGATAAAAC  143
            ||||||||||||||||||||||||||||||||||||||||||||||||||||||||||||
Sbjct  61   GTCTGATTGTATAGGAGTTTTTGAACTAAAAAAGGAGCAATAACGCCCTCTTGATAAAAC  120

Query  144  AAGAGGGAAGCTATTGCTCC-tttttttATTTAGTACTATTTGCCTTACATAGTTTCTTT  202
```

```
                |||||||||||||||||||| |||||||||||||||||||||||||||||||||||||||
Sbjct  121   AAGAGGGAAGCTATTGCTCCTTTTTTTATTTAGTACTATTTGCCTTACATAGTTTCTTT  180

Query  203   AAAAATAACAAGTACTTTTTATAGTTTGGTTCGatttgcgtgttttttctttgtattcat  262
             ||||||||||||||||||||||||||||||||||||||||||||||||||||||||||||
Sbjct  181   AAAAATAACAAGTACTTTTTATAGTTTGGTTCGATTTGCGTGTTTTTTCTTTGTATTCAT  240

Query  263   ttatattataggtttctatatcatttatttttcccaatcttttatgaagttttatttaCA  322
             |||||||||||||||||||||||| |||||||||||||||||||||||||||||||||||
Sbjct  241   TTATATTATAGGTTTCTATATCATATATTTTTCCCAATCTTTTATGAAGTTTTATTTACA  300

Query  323   ATTTAAATTTCAATCGaaaaaaagagaaaaaatgacaaattttgctt-ttttgattttat  381
             |||| ||||||||||||||| | |||||||| |||||| |||  ||| |||||| |||||
Sbjct  301   ATTT-AATTTCAATCGAAAATA-GAGAAAAA-TGACAA-TTT--CTTATTTTGA-TTTAT  353

Query  382   attgattaaa--a---tca-aaa---at-aaag-aaaa---gaatt--t-tgcttttttt  424
             ||||||||||  |   | | |||   || |||  ||||   |||    | ||||||||||
Sbjct  354   ATTGATTAAATCAAAATAAGAAATTGATTAAATCAAAATAAGAAAGAATATGCTTTTTTT  413

Query  425   tATTGTTAAATGGaaaaaaaTATAGTTAATACTAGATAATCCGTAGAAGGGGGCGGGATG  484
              || ||||| ||||||||  ||||| ||||||||||||||| ||||| |||| ||| |||
Sbjct  414   -AT-GTTAA-TGGAAAAA--TATAG-TAATACTAGATAATC-GTAGA-GGGG-CGG-ATG  463

Query  485   TAGGCCAAAGTGGATCAAGG  504
             ||| |||| |||||||||||
Sbjct  464   TAG-CCAA-GTGGATCAAGG  481


>MZ292978.1 Acmella paniculata chloroplast, complete genome
Length=152117

 Score = 634 bits (343),  Expect = 4e-177
 Identities = 456/506 (90%), Gaps = 26/506 (5%)
 Strand=Plus/Minus

Query  2     GTTATGCATGAACGTAATGCTCATAATTTCCCTCTAGACTTAGCTGCTATTGAAGCTCCA  61
             ||||||||||||||||||||||||||||||||||||||||||||||||||||||||||||
Sbjct  531   GTTATGCATGAACGTAATGCTCATAATTTCCCTCTAGACTTAGCTGCTATTGAAGCTCCA  472

Query  62    TCTACAAATGGATAAGCCTTTGGTCTGATTGTATAGGAGTTTTTGAACTAAAAAAGGAGC  121
             ||||| |||||||||| |||||||||||||||||||||||||||||||||||||||||||
Sbjct  471   TCTACCAATGGATAAGACTTTGGTCTGATTGTATAGGAGTTTTTGAACTAAAAAAGGAGC  412

Query  122   AATAAC-GCCCTCTTGATAAAACAAGAGGGAAGCTATTGCTCCtttttttATTTAGTACT  180
             |||| |  |||||||| |  | ||||||||  | ||||||||||||||||||||||||||
Sbjct  411   AATAGCTTCCCTCTTGTTTTATCAAGAGGG-CGTTATTGCTCCTTTTTTTATTTAGTACT  353

Query  181   ATTTGCCTTACATAGTTTCTTTAAAAATAACAAGTACTTTTTATAGTTTGGTTCGatttg  240
             ||||||||||||||||||||||||||||||||||||||||||||||||||||||||||||
Sbjct  352   ATTTGCCTTACATAGTTTCTTTAAAAATAACAAGTACTTTTTATAGTTTGGTTCGATTTG  293

Query  241   cgtgttttttctttgtattcatttatattataggtttctatatcatttatttttcccaat  300
             ||||||||||||||||||||||||||||||||||||| |||||||| || ||||||||||
Sbjct  292   CGTGTTTTTTCTTTGTATTCATTTATATTATAGGTTTATATATCATATACTTTTCCCAAT  233

Query  301   cttttatgaagttttatttaCAATTTAAATTTCAATCGaaaaaaagagaaaaaatgacaa  360
             |||||||||||| |||||||||||| || ||||||||||||| | || ||||| |||| |
```

```
Sbjct  232  CTTTTATGAAGTGTTATTTACAATTCAA-TTTCAATCGAAAATA-GATAAAAA-TGAC-A  177

Query  361  attttgcttttttgattttatattgattaaaatcaaaaataa-aga-aaagaattttgct  418
            ||||||||| |||  |||||| || |  ||| | |||||||| | | ||| |||  ||||
Sbjct  176  ATTTTGCTTATTT--TTTTAT-TTAAG-AAA-T-AAAAATAAGAAATAAATAATA-TGCT  124

Query  419  tttttttATTGTTAAATGGaaaaaaaTATAGTTAATACTAGATAATCCGTAGAAGGGGGC  478
            ||||||||| ||||| ||||||||  ||||| ||||||||||||||  ||||| |||| |
Sbjct  123  TTTTTTTAT-GTTAA-TGGAAAAA--TATAG-TAATACTAGATAATA-GTAGA-GGGG-C  72

Query  479  GGGATGTAGGCCAAAGTGGATCAAGG  504
            || |||||| |||| |||||||||||
Sbjct  71   GG-ATGTAG-CCAA-GTGGATCAAGG  49


 Score = 110 bits (59),  Expect = 3e-19
 Identities = 59/59 (100%), Gaps = 0/59 (0%)
 Strand=Plus/Plus

Query  111  AAAAAAGGAGCAATAACGCCCTCTTGATAAAACAAGAGGGAAGCTATTGCTCCtttttt  169
            |||||||||||||||||||||||||||||||||||||||||||||||||||||||||||
Sbjct  364  AAAAAAGGAGCAATAACGCCCTCTTGATAAAACAAGAGGGAAGCTATTGCTCCTTTTTT  422


>KF241798.1 Ambrosia artemisiifolia isolate AZ-1 PsbA (psbA) gene, partial
cds; and psbA-trnH intergenic spacer, partial sequence; chloroplast
Length=469

 Score = 634 bits (343),  Expect = 4e-177
 Identities = 449/494 (91%), Gaps = 31/494 (6%)
 Strand=Plus/Plus

Query  9    ATGAACGTAATGCTCATAATTTCCCTCTAGACTTAGCTGCTATTGAAGCTCCATCTACAA  68
            ||||||||||||||||||||||||||||||||||||||||||||||||||||||||||||
Sbjct  1    ATGAACGTAATGCTCATAATTTCCCTCTAGACTTAGCTGCTATTGAAGCTCCATCTACAA  60

Query  69   ATGGATAAGCCTTTGGTCTGATTGTATAGGAGTTTTTGAACTAAAAAAGGAGCAATAACG  128
            ||||||||| ||||||||||||||||||||||||||||||||||||||||||||||||||
Sbjct  61   ATGGATAAGACTTTGGTCTGATTGTATAGGAGTTTTTGAACTAAAAAAGGAGCAATAACG  120

Query  129  CCCTCTTGATAAAACAAGAGGGAAGCTATTGCTCC-tttttttATTTAGTACTATTTGCC  187
            ||||||||||||||||||||||||||||||||||| ||||||||||||||| ||||||||
Sbjct  121  CCCTCTTGATAAAACAAGAGGGAAGCTATTGCTCCTTTTTTTTATTTAGTATTATTTGCC  180

Query  188  TTACATAGTTTCTTTAAAAATAACAAGTACTTTTTATAGTTTGGTTCGatttgcgtgttt  247
            |||||||||||||||||||||||||||||||||||||||||||||||||||||| |||||
Sbjct  181  TTACATAGTTTCTTTAAAAATAACAAGTACTTTTTATAGTTTGGTTCGATTTGCATGTTT  240

Query  248  tttctttgtattcatttatattataggtttctatatcatttatttttcccaatcttttat  307
            ||| ||||||||||||||||||||||||||||| || |  || |||||||||||||||||
Sbjct  241  TTTATTTGTATTCATTTATATTATAGGTTTCTA-AT-A--TACTTTTCCCAATCTTTTAT  296

Query  308  gaagttttatttaCAATTTAAATTTCAATCGaaaaaaagagaaaaaatgacaaattttgc  367
            ||||||||||||||||||  ||||||||||||||| | || ||||| |||||| ||||||
Sbjct  297  GAAGTTTTATTTACAATTC-AATTTCAATCGAAAATA-GATAAAAA-TGACAA-TTTTGC  352

Query  368  tt-ttt-tga-ttttatattgattaaaatcaaaaat-a-aagaaaagaattttgcttttt  422
```

```
                || ||| | | |||||| || |||||| | |||||| | ||  ||| |||  ||||||||
Sbjct  353   TTATTTATTACTTTTAT-TT-ATTAAA-T-AAAAATCAGAAAGAAATAATA-TGCTTTTT  407

Query  423   tttATTGTTAAATGGaaaaaaaTATAGTTAATACTAGATAATCCGTAGAAGGGGGCGGGA  482
             |||||| | || ||||||||  |||| |||||||||||||||  ||||| |||| ||| |
Sbjct  408   TTTATT-T-AA-TGGAAAAA--TATA-TTAATACTAGATAATA-GTAGA-GGGG-CGG-A  457

Query  483   TGTAGGCCAAAGTG  496
             ||||| |||| |||
Sbjct  458   TGTAG-CCAA-GTG  469
```

>MF037492.1 Echinacea pallida voucher Rebman1053 trnH-psbA intergenic spacer region, partial sequence; chloroplast
Length=405

```
 Score = 623 bits (337),  Expect = 9e-174
 Identities = 396/421 (94%), Gaps = 18/421 (4%)
 Strand=Plus/Plus

Query  45    CTGCTATTGAAGCTCCATCTACAAATGGATAAGCCTTTGGTCTGATTGTATAGGAGTTTT  104
             ||||||||||||||||||||||||||||||||||||||||||||||||||||||||||||
Sbjct  1     CTGCTATTGAAGCTCCATCTACAAATGGATAAGCCTTTGGTCTGATTGTATAGGAGTTTT  60

Query  105   TGAACTAAAAAAGGAGCAATAACGCCCTCTTGATAAAACAAGAGGGAAGCTATTGCTCCt  164
             |||||||||||||||||||||||||||||||| |  | |||||||||||||||||||||
Sbjct  61    TGAACTAAAAAAGGAGCAATAACGCCCTCTTGTTTTATCAAGAGGGAAGCTATTGCTCCT  120

Query  165   tttttATTTAGTACTATTTGCCTTACATAGTTTCTTTAAAAATAACAAGTACTTTTTAT  224
             ||||||||||||||||||||||||||||||||||||||||||||||||||||||||||||
Sbjct  121   TTTTTATTTAGTACTATTTGCCTTACATAGTTTCTTTAAAAATAACAAGTACTTTTTAT  180

Query  225   AGTTTGGTTCGatttgcgtgtttttctttgtattcatttatattataggtttctatatc  284
             ||||||||||||||||||||||||||||||||||||||||||||||||||||||||||||
Sbjct  181   AGTTTGGTTCGATTTGCGTGTTTTTCTTTGTATTCATTTATATTATAGGTTTCTATATC  240

Query  285   atttattttcccaatcttttatgaagttttatttaCAATTTAAATTTCAATCGaaaaaa  344
             || |||||||||||||||||||||||||||||||||||||| |||||||||||||| |
Sbjct  241   ATATATTTTTCCCAATCTTTTATGAAGTTTATTTACAATTT-AATTTCAATCGAAAATA  299

Query  345   agagaaaaaatgacaaattttgctttttgattttatattgattaaaatcaaaaataaag  404
              |||||||| |||| |||||||||||||| | ||||||||||||||| |||||| ||| |
Sbjct  300   -GAGAAAAA-TGAC-AATTTTGCTTTTTT-A-TTTATATTGATTAAA-TCAAAA-TAA-G  351

Query  405   aaaagaattttgctttttttttATTGTTAAATGGaaaaaaaTATAGTTAATACTAGATAAT  464
             ||| |||| | ||||||||  || ||||| ||||||||  ||||| ||||||||||||||
Sbjct  352   AAA-GAATAT-GCTTTTTT--AT-GTTAA-TGGAAAAA--TATAG-TAATACTAGATAAT  402

Query  465   C  465
             |
Sbjct  403   C  403
```

>AB427162.1 Ambrosia artemisiifolia chloroplast psbA gene for D1 protein, complete cds
Length=1693

```
 Score = 617 bits (334),  Expect = 4e-172
 Identities = 457/509 (90%), Gaps = 37/509 (7%)
 Strand=Plus/Plus

Query  2     GTTATGCATGAACGTAATGCTCATAATTTCCCTCTAGACTTAGCTGCTATTGAAGCTCCA  61
             ||||||||||||||||||||||||||||||||||||||||||||||||||||||||||||
Sbjct  1206  GTTATGCATGAACGTAATGCTCATAATTTCCCTCTAGACTTAGCTGCTATTGAAGCTCCA  1265

Query  62    TCTACAAATGGATAAGCCTTTGGTCTGATTGTATAGGAGTTTTTGAACTAAAAAAGGAGC  121
             |||||||||||||||| |||||||||||||||||||||||||||||||||||||||||||
Sbjct  1266  TCTACAAATGGATAAGACTTTGGTCTGATTGTATAGGAGTTTTTGAACTAAAAAAGGAGC  1325

Query  122   AATAACGCCCTCTTGATAAAACAAGAGGGAAGCTATTGCTCC-ttttttATTTAGTACT  180
             ||||||| |||||||||||||||||||||||||||||||||| |||||||||||||||||
Sbjct  1326  AATAACGTCCTCTTGATAAAACAAGAGGGAAGCTATTGCTCCTTTTTTTATTTAGTACT  1385

Query  181   ATTTGCCTTACATAGTTTCTTTAAAAATAACAAGTACTTTTTATAGTTTGGTTCGatttg  240
             |||||||||||||||||||||||||||||         ||||||||||||||||||||||
Sbjct  1386  ATTTGCCTTACATAGTTTCTTTAAAAATA---------TTTTATAGTTTGGTTCGATTTG  1436

Query  241   cgtgttttttctttgtattcatttatattataggtttctatatcatttatttttcccaat  300
             |||||||||||||||||||||||||||||||||||||||||||||| || ||||||||||
Sbjct  1437  CGTGTTTTTTCTTTGTATTCATTTATATTATAGGTTTCTATATCATATACTTTTCCCAAT  1496

Query  301   cttttatgaagttttatttaCAATTTAAATTTCAATCGaaaaaaagagaaaaaatgacaa  360
             |||||||||||||||||||||||||  ||||||||||||||| | || ||||| ||||||
Sbjct  1497  CTTTTATGAAGTTTTATTTACAATTC-AATTTCAATCGAAAATA-GACAAAAA-TGACAA  1553

Query  361   attttgctt-ttt-tga-ttttatattgattaaaatcaaaa-a-taaagaaaagaatttt  415
              |||| ||| ||| | | |||||| || |||||| | |||| | ||||||||  ||| |
Sbjct  1554  TTTTT-CTTATTTATTACTTTTAT-TT-ATTAAA-TAAAAAGAATAAAGAAAT-AATAT-  1607

Query  416   gcttttttttATTGTTAAATGGaaaaaaaTATAGTTAATACTAGATAATCCGTAGAAGGG  475
             |||||||   ||| | || ||| ||||| ||||| ||||||||||||| | ||||| |||
Sbjct  1608  GCTTTTTG--ATT-T-AA-TGGGAAAAA-TATAG-TAATACTAGATAA-CAGTAGA-GGG  1658

Query  476   GGCGGGATGTAGGCCAAAGTGGATCAAGG  504
             | ||| |||||| |||| |||||||||||
Sbjct  1659  G-CGG-ATGTAG-CCAA-GTGGATCAAGG  1683


>NC_035875.1 Ambrosia artemisiifolia chloroplast, complete genome
 MF362689.1 Ambrosia artemisiifolia chloroplast, complete genome
Length=152215

 Score = 617 bits (334),  Expect = 4e-172
 Identities = 457/509 (90%), Gaps = 38/509 (7%)
 Strand=Plus/Minus

Query  2    GTTATGCATGAACGTAATGCTCATAATTTCCCTCTAGACTTAGCTGCTATTGAAGCTCCA  61
            ||||||||||||||||||||||||||||||||||||||||||||||||||||||||||||
Sbjct  707  GTTATGCATGAACGTAATGCTCATAATTTCCCTCTAGACTTAGCTGCTATTGAAGCTCCA  648

Query  62   TCTACAAATGGATAAGCCTTTGGTCTGATTGTATAGGAGTTTTTGAACTAAAAAAGGAGC  121
            |||||||||||||||| |||||||||||||||||||||||||||||||||||||||||||
Sbjct  647  TCTACAAATGGATAAGACTTTGGTCTGATTGTATAGGAGTTTTTGAACTAAAAAAGGAGC  588
```

```
Query  122   AATAACGCCCTCTTGATAAAACAAGAGGGAAGCTATTGCTCCttttttt-ATTTAGTACT  180
             ||||||| ||||||||||||||||||||||||||||||||||||||||| ||||||||||
Sbjct  587   AATAACGTCCTCTTGATAAAACAAGAGGGAAGCTATTGCTCCTTTTTTTTATTTAGTACT  528

Query  181   ATTTGCCTTACATAGTTTCTTTAAAAATAACAAGTACTTTTTATAGTTTGGTTCGatttg  240
             |||||||||||||||||||||||||||||         ||||||||||||||||||||||
Sbjct  527   ATTTGCCTTACATAGTTTCTTTAAAAATA---------TTTTATAGTTTGGTTCGATTTG  477

Query  241   cgtgttttttctttgtattcatttatattataggtttctatatcatttatttttcccaat  300
             |||||||||||||||||||||||||||||||||||||||||||||||| || ||||||||||
Sbjct  476   CGTGTTTTTTCTTTGTATTCATTTATATTATAGGTTTCTATATCATATACTTTTCCCAAT  417

Query  301   cttttatgaagttttatttaCAATTTAAATTTCAATCGaaaaaaagagaaaaaatgacaa  360
             ||||||||||||||||||||||||| || ||||||||||||| | || ||||| ||||||
Sbjct  416   CTTTTATGAAGTTTTATTTACAATTCAA-TTTCAATCGAAAATA-GACAAAAA-TGACAA  360

Query  361   attttgctt-ttt-tga-ttttatattgattaaaatcaaaa-a-taaagaaaagaatttt  415
              |||| ||| ||| | | |||||| || |||||| | |||| | ||||||||  ||| |
Sbjct  359   TTTTT-CTTATTTATTACTTTTAT-TT-ATTAAA-TAAAAAGAATAAAGAAAT-AATAT-  306

Query  416   gcttttttttATTGTTAAATGGaaaaaaaTATAGTTAATACTAGATAATCCGTAGAAGGG  475
             |||||||   ||| | || ||||||||  ||||| ||||||||||||| | ||||| |||
Sbjct  305   GCTTTTTG--ATT-T-AA-TGGAAAAA--TATAG-TAATACTAGATAA-CAGTAGA-GGG  256

Query  476   GGCGGGATGTAGGCCAAAGTGGATCAAGG  504
             | ||| |||||| |||| |||||||||||
Sbjct  255   G-CGG-ATGTAG-CCAA-GTGGATCAAGG  231
```

```
>MG019037.1 Ambrosia artemisiifolia chloroplast, complete genome
Length=152223

 Score = 617 bits (334),  Expect = 4e-172
 Identities = 458/510 (90%), Gaps = 39/510 (8%)
 Strand=Plus/Minus

Query  2     GTTATGCATGAACGTAATGCTCATAATTTCCCTCTAGACTTAGCTGCTATTGAAGCTCCA  61
             ||||||||||||||||||||||||||||||||||||||||||||||||||||||||||||
Sbjct  535   GTTATGCATGAACGTAATGCTCATAATTTCCCTCTAGACTTAGCTGCTATTGAAGCTCCA  476

Query  62    TCTACAAATGGATAAGCCTTTGGTCTGATTGTATAGGAGTTTTTGAACTAAAAAAGGAGC  121
             |||||||||||||||| |||||||||||||||||||||||||||||||||||||||||||
Sbjct  475   TCTACAAATGGATAAGACTTTGGTCTGATTGTATAGGAGTTTTTGAACTAAAAAAGGAGC  416

Query  122   AATAACG-CCCTCTTGATAAAACAAGAGGGAAGCTATTGCTCCttttttt-ATTTAGTAC  179
             ||||||| |||||||||||||||||||||||||||||||||||||||||| |||||||||
Sbjct  415   AATAACGTCCCTCTTGATAAAACAAGAGGGAAGCTATTGCTCCTTTTTTTTATTTAGTAC  356

Query  180   TATTTGCCTTACATAGTTTCTTTAAAAATAACAAGTACTTTTTATAGTTTGGTTCGattt  239
             ||||||||||||||||||||||||||||||         |||||||||||||||||||||
Sbjct  355   TATTTGCCTTACATAGTTTCTTTAAAAATA---------TTTTATAGTTTGGTTCGATTT  305

Query  240   gcgtgttttttctttgtattcatttatattataggtttctatatcatttatttttcccaa  299
             ||||||||||||||||||||||||||||||||||||||||||||||| || |||||||||
Sbjct  304   GCGTGTTTTTTCTTTGTATTCATTTATATTATAGGTTTCTATATCATATACTTTTCCCAA  245

Query  300   tcttttatgaagttttatttaCAATTTAAATTTCAATCGaaaaaaagagaaaaaatgaca  359
```

```
             ||||||||||||||||||||||||||| || ||||||||||||| | || ||||| |||||
Sbjct  244  TCTTTTATGAAGTTTTATTTACAATTCAA-TTTCAATCGAAAATA-GACAAAAA-TGACA  188

Query  360  aatttgctt-ttt-tga-ttttatattgattaaaatcaaaa-a-taaagaaaagaattt  414
            | |||| ||| ||| | | |||||| || |||||| | |||| | ||||||||  ||| |
Sbjct  187  ATTTTT-CTTATTTATTACTTTTAT-TT-ATTAAA-TAAAAAGAATAAAGAAAT-AATAT  133

Query  415  tgcttttttttATTGTTAAATGGaaaaaaaTATAGTTAATACTAGATAATCCGTAGAAGG  474
             |||||||   ||| | || ||||||||  ||||| ||||||||||||| | ||||| ||
Sbjct  132  -GCTTTTTG--ATT-T-AA-TGGAAAAA--TATAG-TAATACTAGATAA-CAGTAGA-GG  84

Query  475  GGGCGGGATGTAGGCCAAAGTGGATCAAGG  504
            || ||| |||||| |||| |||||||||||
Sbjct  83   GG-CGG-ATGTAG-CCAA-GTGGATCAAGG  58


>NC_036810.2 Ambrosia trifida chloroplast, complete genome
 MG029118.2 Ambrosia trifida chloroplast, complete genome
Length=152040

 Score = 617 bits (334),  Expect = 4e-172
 Identities = 457/509 (90%), Gaps = 38/509 (7%)
 Strand=Plus/Minus

Query  2    GTTATGCATGAACGTAATGCTCATAATTTCCCTCTAGACTTAGCTGCTATTGAAGCTCCA  61
            ||||||||||||||||||||||||||||||||||||||||||||||||||||||||||||
Sbjct  534  GTTATGCATGAACGTAATGCTCATAATTTCCCTCTAGACTTAGCTGCTATTGAAGCTCCA  475

Query  62   TCTACAAATGGATAAGCCTTTGGTCTGATTGTATAGGAGTTTTTGAACTAAAAAAGGAGC  121
            |||||||||||||||| |||||||||||||||||||||||||||||||||||||||||||
Sbjct  474  TCTACAAATGGATAAGACTTTGGTCTGATTGTATAGGAGTTTTTGAACTAAAAAAGGAGC  415

Query  122  AATAACGCCCTCTTGATAAAACAAGAGGGAAGCTATTGCTCCttttttt-ATTTAGTACT  180
            ||||||| ||||||||||||||||||||||||||||||||||||||||| ||||||||||
Sbjct  414  AATAACGTCCTCTTGATAAAACAAGAGGGAAGCTATTGCTCCTTTTTTTTATTTAGTACT  355

Query  181  ATTTGCCTTACATAGTTTCTTTAAAAATAACAAGTACTTTTTATAGTTTGGTTCGatttg  240
            |||||||||||||||||||||||||||         |||||||||||||||||||||
Sbjct  354  ATTTGCCTTACATAGTTTCTTTAAAAATA---------TTTTATAGTTTGGTTCGATTTG  304

Query  241  cgtgtttttttctttgtattcatttatattataggtttctatatcatttattttcccaat  300
            |||||||||||||||||||||||||||||||||||||||||||||| || ||||||||||
Sbjct  303  CGTGTTTTTTCTTTGTATTCATTTATATTATAGGTTTCTATATCATATACTTTTCCCAAT  244

Query  301  cttttatgaagttttatttaCAATTTAAATTTCAATCGaaaaaaagagaaaaaatgacaa  360
            ||||||||||||||||||||||||| || ||||||||||||| | || ||||| ||||||
Sbjct  243  CTTTTATGAAGTTTTATTTACAATTCAA-TTTCAATCGAAAATA-GACAAAAA-TGACAA  187

Query  361  atttgctt-ttt-tga-ttttatattgattaaaatcaaaa-a-taaagaaaagaattt  415
             |||| ||| ||| | | |||||| || |||||| | |||| | ||||||||  ||| |
Sbjct  186  TTTTT-CTTATTTATTACTTTTAT-TT-ATTAAA-TAAAAAGAATAAAGAAAT-AATAT-  133

Query  416  gcttttttttATTGTTAAATGGaaaaaaaTATAGTTAATACTAGATAATCCGTAGAAGGG  475
            |||||||   ||| | || ||||||||  ||||| ||||||||||||| | ||||| |||
Sbjct  132  GCTTTTTG--ATT-T-AA-TGGAAAAA--TATAG-TAATACTAGATAA-CAGTAGA-GGG  83

Query  476  GGCGGGATGTAGGCCAAAGTGGATCAAGG  504
```

```
            | ||| |||||| |||| |||||||||||
Sbjct  82   G-CGG-ATGTAG-CCAA-GTGGATCAAGG  58


>NC_054222.1 Xanthium spinosum chloroplast, complete genome
 MT668935.1 Xanthium spinosum chloroplast, complete genome
Length=152422

 Score = 608 bits (329),  Expect = 3e-169
 Identities = 465/524 (89%), Gaps = 36/524 (7%)
 Strand=Plus/Minus

Query  2    GTTATGCATGAACGTAATGCTCATAATTTCCCTCTAGACTTAGCTGCTATTGAAGCTCCA  61
            ||||||||||||||||||||||||||||||||||||||||||||||||||||||||||||
Sbjct  676  GTTATGCATGAACGTAATGCTCATAATTTCCCTCTAGACTTAGCTGCTATTGAAGCTCCA  617

Query  62   TCTACAAATGGATAAGCCTTTGGTCTGATTGTATAGGAGTTTTTGAACTAAAAAAGGAGC  121
            |||||||||||||||| |||||||||||||||||||||||||||||||||||||||||||
Sbjct  616  TCTACAAATGGATAAGACTTTGGTCTGATTGTATAGGAGTTTTTGAACTAAAAAAGGAGC  557

Query  122  AATAACGCCCTCTTGATAAAACAAGAGGGAAGCTATTGCTCCtttttttATTTAGTACTA  181
            ||||||||||||||||||||||||||||||||||||||||||||||||||||||||||||
Sbjct  556  AATAACGCCCTCTTGATAAAACAAGAGGGAAGCTATTGCTCCTTTTTTTATTTAGTACTA  497

Query  182  TTTGCCTTACATAGTTTCTTTAAAAATAACAAGTACTTTTTATAGTTTGGTTCGatttgc  241
            |||||||||||||||||||||||||| ||||||||||||||||||||||||| |||||||
Sbjct  496  TTTGCCTTACATAGTTTCTTTAAAAAAAACAAGTACTTTTTATAGTTTGGTTTGATTTGC  437

Query  242  gtgttttttctttgtattcatttatattat--ag-----------gtttctatatcattt  288
            ||||||||||||||||||||||||||||||  |            ||||||||||||| |
Sbjct  436  GTGTTTTTTCTTTGTATTCATTTATATTATTCATTTATATTATATGTTTCTATATCATAT  377

Query  289  atttttcccaatcttttatgaagttttatttaCAATTTAAATTTCAATCGaaaaaaagag  348
            | ||||||||||||||||||||||||||||||||||| || ||||||||||||| |  | 
Sbjct  376  ACTTTTCCCAATCTTTTATGAAGTTTTATTTACAATTCAA-TTTCAATCGAAAATAT-AT  319

Query  349  aaaaaatgacaaattttgctt-ttt-tga-ttttatattg-a-ttaaaatcaaaaataa-  402
            ||||| | | ||||||||||| ||| | | |||| | ||  | || |||| |||||||| 
Sbjct  318  AAAAA-TAA-AAATTTTGCTTATTTATTACTTTTCTTTTTTAATTTAAAT-AAAAATAAT  262

Query  403  aga-aaagaattttgcttttttttATTGTTA-AATGGaaaaaaaTATAGTTAATACTAGA  460
            | | ||| |||  ||||||||||| || ||  ||||||||||  ||||| ||||||||||
Sbjct  261  AAATAAATAATA-TGCTTTTTTTTTTTTTTTTAATGGAAAAA--TATAG-TAATACTAGA  206

Query  461  TAATCCGTAGAAGGGGGCGGGATGTAGGCCAAAGTGGATCAAGG  504
            ||||  ||||| |||| ||| |||||| |||| |||||||||||
Sbjct  205  TAATA-GTAGA-GGGG-CGG-ATGTAG-CCAA-GTGGATCAAGG  168


>MZ324674.1 Hulsea algida voucher Baldwin 678 (DAV) photosystem II protein
D1 (psbA) gene, partial cds; psbA-trnH intergenic spacer,
complete sequence; and tRNA-His (trnH) gene, partial sequence;
plastid
Length=490

 Score = 608 bits (329),  Expect = 3e-169
 Identities = 445/496 (90%), Gaps = 28/496 (6%)
```

```
 Strand=Plus/Plus

Query  15   GTAATGCTCATAATTTCCCTCTAGACTTAGCTGCTATTGAAGCTCCATCTACAAATGGAT  74
            ||||||||| ||||||||||||||||||||||||||||||||||||||||||||||||||
Sbjct  1    GTAATGCTCGTAATTTCCCTCTAGACTTAGCTGCTATTGAAGCTCCATCTACAAATGGAT  60

Query  75   AAGCCTTTGGTCTGATTGTATAGGAGTTTTTGAACTAAAAAAGGAGCAATAACGCCCCTCT  134
            ||| ||||||||||||||||||||||||||||||||||||||||||||||||||||||||
Sbjct  61   AAGACTTTGGTCTGATTGTATAGGAGTTTTTGAACTAAAAAAGGAGCAATAACGCCCCTCT  120

Query  135  TGATAAAACAAGAGGGAAGCTATTGCTCC-tttttttATTTAGTACTATTTGCCTTACAT  193
            ||||||||||||||||||||||||||||| ||||||||||||||||||||||||||||||
Sbjct  121  TGATAAAACAAGAGGGAAGCTATTGCTCCTTTTTTTTATTTAGTACTATTTGCCTTACAT  180

Query  194  AGTTTCTTTAAAAATAACAAGTACTTTTTATAGTTTGGTTCGatttgcgtgttttttctt  253
            ||||||||||||||||||||| ||| ||||||||||||||||||||||||||||||| ||
Sbjct  181  AGTTTCTTTAAAAATAACAAGGACTCTTTATAGTTTGGTTCGATTTGCGTGTTTTTTATT  240

Query  254  tgtattcatttatattataggtttctatatcatttatttttcccaatcttttatgaagtt  313
            ||||||||||||||||||||||||||||||||| || |||| ||||||||||||||||||
Sbjct  241  TGTATTCATTTATATTATAGGTTTCTATATCATATACTTTT-CCAATCTTTTATGAAGTT  299

Query  314  ttatttaCAATTTAAATTTCAATCGaaaaa--aagaga-aaaaatgacaaattttgctt-  369
            |||||| |||||  |||||||||||||||    ||   | ||||||  ||| ||||||||
Sbjct  300  TTATTTCCAATTC-AATTTCAATCGAAAATTGAATTTATAAAAATT-CAATTTTTGCTTA  357

Query  370  ttt-tgattttatattgattaaaatcaaaaataaagaaaagaattttgcttttttttATT  428
            ||| | | ||| | || |||||| | || ||||||||||  ||| | ||||||||  ||
Sbjct  358  TTTATTACTTT-T-TT-ATTAAATTAAATAATAAAGAAAT-AATAT-GCTTTTTT--AT-  409

Query  429  GTTAAATGGaaaaaaaTATAGTTAATACTAGATAATCCGTAGAAGGGGGCGGGATGTAGG  488
            ||||| || |||||||||  | ||||||||||||||  ||||| |||| ||| ||||||
Sbjct  410  GTTAA-TG-AAAAAAATA--G-TAATACTAGATAATA-GTAGA-GGGG-CGG-ATGTAG-  459

Query  489  CCAAAGTGGATCAAGG  504
            |||| |||||||||||
Sbjct  460  CCAA-GTGGATCAAGG  474


>NC_053299.1 Iostephane heterophylla voucher Schilling 94 chloroplast, complete
genome
 MT700542.1 Iostephane heterophylla chloroplast, complete genome
Length=151495

 Score = 606 bits (328),  Expect = 9e-169
 Identities = 459/515 (89%), Gaps = 38/515 (7%)
 Strand=Plus/Minus

Query  2    GTTATGCATGAACGTAATGCTCATAATTTCCCTCTAGACTTAGCTGCTATTGAAGCTCCA  61
            ||||||||||||||||||||||||||||||||||||||||||||||||||||||||||||
Sbjct  537  GTTATGCATGAACGTAATGCTCATAATTTCCCTCTAGACTTAGCTGCTATTGAAGCTCCA  478

Query  62   TCTACAAATGGATAAGCCTTTGGTCTGATTGTATAGGAGTTTTTGAACTAAAAAAGGAGC  121
            |||||||||||||||| |||||||||||||||||||||||||||||||||||||||||||
Sbjct  477  TCTACAAATGGATAAGACTTTGGTCTGATTGTATAGGAGTTTTTGAACTAAAAAAGGAGC  418

Query  122  AATAACG-CCCTCTTGATAAAACAAGAGGGAAGCTATTGCTCCtttttttATTTAGTACT  180
```

```
                ||||  |  ||||||||||||||||||||||  |  ||||||||||||||||||||||||||
Sbjct  417  AATAGCTTCCCTCTTGATAAAACAAGAGGGC-GTTATTGCTCCTTTTTTTATTTAGTACT  359

Query  181  ATTTGCCTTACATAGTTTCTTTAAA-------AATAACAAGTACTTTTTATAGTTTGGTT  233
            |||||||||||||||||||||||||       ||||||||||||||||||||||||||||
Sbjct  358  ATTTGCCTTACATAGTTTCTTTAAACTTTAAAAATAACAAGTACTTTTTATAGTTTGGTT  299

Query  234  CGatttgcgtgttttttctttgtattcatttatattataggtttctatatcatttatttt  293
            |||||||| |||||||| ||||||||||||||||||||||||||||||||||| ||||||
Sbjct  298  CGATTTGCTTGTTTTTTATTTGTATTCATTTATATTATAGGTTTCTATATCATATATTTT  239

Query  294  tcccaatcttttatgaagttttattt-aCAATTTAAATTTCAATCGaaaaaaagagaaaa  352
            ||| | |||||||||||||||| ||| ||||||| ||||||||||||||| | || ||||
Sbjct  238  TCCTACTCTTTTATGAAGTTTTTTTTTACAATTT-AATTTCAATCGAAAATA-GATAAAA  181

Query  353  aatgacaaattttgctt-ttt-tga-ttttatattgattaaaatcaaaaataaagaaaag  409
              |||||| |||||||| ||| | | |||||| || ||| || | |||||| | |||| |
Sbjct  180  --TGACAA-TTTTGCTTATTTATTACTTTTAT-TT-ATTCAA-T-AAAAATGA-GAAA-G  130

Query  410  aattttgcttttttttATTGTTAAATGGaaaaaaaTATAGTTAATACTAGATAATCCGTA  469
            ||| | ||||||||  || ||||| ||||||||  ||||| ||||||||||||||  |||
Sbjct  129  AATAT-GCTTTTTT--AT-GTTAA-TGGAAAAA--TATAG-TAATACTAGATAATA-GTA  79

Query  470  GAAGGGGGCGGGATGTAGGCCAAAGTGGATCAAGG  504
            || |||| ||| |||||| |||| |||||||||||
Sbjct  78   GA-GGGG-CGG-ATGTAG-CCAA-GTGGATCAAGG  49
```

>KF241797.1 Ambrosia artemisiifolia isolate PH-53 PsbA (psbA) gene, partial cds; and psbA-trnH intergenic spacer, partial sequence; chloroplast
Length=474

Score = 601 bits (325), Expect = 4e-167
Identities = 450/503 (89%), Gaps = 38/503 (8%)
Strand=Plus/Plus

```
Query  8    CATGAACGTAATGCTCATAATTTCCCTCTAGACTTAGCTGCTATTGAAGCTCCATCTACA  67
            ||||||||||||||||||||||||||||||||||||||||||||||||||||||||||||
Sbjct  1    CATGAACGTAATGCTCATAATTTCCCTCTAGACTTAGCTGCTATTGAAGCTCCATCTACA  60

Query  68   AATGGATAAGCCTTTGGTCTGATTGTATAGGAGTTTTTGAACTAAAAAAGGAGCAATAAC  127
            |||||||||| |||||||||||||||||||||||||||||||||||||||||||||||||
Sbjct  61   AATGGATAAGACTTTGGTCTGATTGTATAGGAGTTTTTGAACTAAAAAAGGAGCAATAAC  120

Query  128  GCCCTCTTGATAAAACAAGAGGGAAGCTATTGCTCC-tttttttATTTAGTACTATTTGC  186
            | |||||||||||||||||||||||||||||||||| |||||||||||||||||||||||
Sbjct  121  GTCCTCTTGATAAAACAAGAGGGAAGCTATTGCTCCTTTTTTTTATTTAGTACTATTTGC  180

Query  187  CTTACATAGTTTCTTTAAAAATAACAAGTACTTTTTATAGTTTGGTTCGatttgcgtgtt  246
            |||||||||||||||||||||||         ||||||||||||||||||||||||||||
Sbjct  181  CTTACATAGTTTCTTTAAAAATA---------TTTTATAGTTTGGTTCGATTTGCGTGTT  231

Query  247  ttttctttgtattcatttatattataggtttctatatcatttatttttcccaatctttta  306
            |||||||||||||||||||||||||||||||||||||||| || ||||||||||||||||
Sbjct  232  TTTTCTTTGTATTCATTTATATTATAGGTTTCTATATCATATACTTTTCCCAATCTTTTA  291

Query  307  tgaagttttatttaCAATTTAAATTTCAATCGaaaaaaagagaaaaaatgacaaattttg  366
```

```
            |||||||||||||||||||  ||||||| ||||||| | || ||||| |||||| ||||
Sbjct  292  TGAAGTTTTATTTACAATTC-AATTTCACTCGAAAATA-GACAAAAA-TGACAATTTTT-  347

Query  367  ctt-ttt-tga-ttttatattgattaaaatcaaaa-a-taaagaaaagaattttgctttt  421
            ||| ||| | | |||||| || |||||| | |||| | ||||||||  ||| | ||||||
Sbjct  348  CTTATTTATTACTTTTAT-TT-ATTAAA-TAAAAAGAATAAAGAAAT-AATAT-GCTTTT  402

Query  422  ttttATTGTTAAATGGaaaaaaaTATAGTTAATACTAGATAATCCGTAGAAGGGGGCGGG  481
            |   ||| | || ||||||||  ||||| ||||||||||||| | ||||| |||| |||
Sbjct  403  TG--ATT-T-AA-TGGAAAAA--TATAG-TAATACTAGATAA-CAGTAGA-GGGG-CGG-  450

Query  482  ATGTAGGCCAAAGTGGATCAAGG  504
            |||||| |||| |||||||||||
Sbjct  451  ATGTAG-CCAA-GTGGATCAAGG  471


>KF241793.1 Ambrosia artemisiifolia isolate NJI-7 PsbA (psbA) gene, partial
cds; and psbA-trnH intergenic spacer, partial sequence; chloroplast
Length=485

 Score = 601 bits (325),  Expect = 4e-167
 Identities = 450/503 (89%), Gaps = 38/503 (8%)
 Strand=Plus/Plus

Query  8    CATGAACGTAATGCTCATAATTTCCCTCTAGACTTAGCTGCTATTGAAGCTCCATCTACA  67
            ||||||||||||||||||||||||||||||||||||||||||||||||||||||||||||
Sbjct  1    CATGAACGTAATGCTCATAATTTCCCTCTAGACTTAGCTGCTATTGAAGCTCCATCTACA  60

Query  68   AATGGATAAGCCTTTGGTCTGATTGTATAGGAGTTTTTGAACTAAAAAAGGAGCAATAAC  127
            |||||||||| |||||||||||||||||||||||||||||||||||||||||||||||||
Sbjct  61   AATGGATAAGACTTTGGTCTGATTGTATAGGAGTTTTTGAACTAAAAAAGGAGCAATAAC  120

Query  128  GCCCTCTTGATAAAACAAGAGGGAAGCTATTGCTCC-tttttttATTTAGTACTATTTGC  186
            | |||||||||||||||||||||||||||||||||| |||||||||||||||||||||||
Sbjct  121  GTCCTCTTGATAAAACAAGAGGGAAGCTATTGCTCCTTTTTTTTATTTAGTACTATTTGC  180

Query  187  CTTACATAGTTTCTTTAAAAATAACAAGTACTTTTTATAGTTTGGTTCGatttgcgtgtt  246
            |||||||||||||||||||||||         ||||||||||||||||||||||||||||
Sbjct  181  CTTACATAGTTTCTTTAAAAATA---------TTTTATAGTTTGGTTCGATTTGCGTGTT  231

Query  247  ttttctttgtattcatttatattataggtttctatatcatttatttttcccaatctttta  306
            |||||||||||||||||||||||||||||||||||||||| || ||||||||||||||||
Sbjct  232  TTTTCTTTGTATTCATTTATATTATAGGTTTCTATATCATATACTTTTCCCAATCTTTTA  291

Query  307  tgaagttttatttaCAATTTAAATTTCAATCGaaaaaaagagaaaaaatgacaaattttg  366
            |||||||||||||||||||  ||||| ||||||||| | || ||||| |||||| ||||
Sbjct  292  TGAAGTTTTATTTACAATTC-AATTTAAATCGAAAATA-GACAAAAA-TGACAATTTTT-  347

Query  367  ctt-ttt-tga-ttttatattgattaaaatcaaaa-a-taaagaaaagaattttgctttt  421
            ||| ||| | | |||||| || |||||| | |||| | ||||||||  ||| | ||||||
Sbjct  348  CTTATTTATTACTTTTAT-TT-ATTAAA-TAAAAAGAATAAAGAAAT-AATAT-GCTTTT  402

Query  422  ttttATTGTTAAATGGaaaaaaaTATAGTTAATACTAGATAATCCGTAGAAGGGGGCGGG  481
            |   ||| | || ||||||||  ||||| ||||||||||||| | ||||| |||| |||
Sbjct  403  TG--ATT-T-AA-TGGAAAAA--TATAG-TAATACTAGATAA-CAGTAGA-GGGG-CGG-  450

Query  482  ATGTAGGCCAAAGTGGATCAAGG  504
```

```
             |||||| |||| |||||||||||
Sbjct  451   ATGTAG-CCAA-GTGGATCAAGG  471
```

>MF348657.1 Acmella oleracea isolate AD3HI37 photosystem II protein D1 (psbA) gene, partial cds; and psbA-trnH intergenic spacer, partial sequence; chloroplast
Length=465

 Score = 599 bits (324), Expect = 2e-166
 Identities = 435/484 (90%), Gaps = 26/484 (5%)
 Strand=Plus/Plus

```
Query  24    ATAATTTCCCTCTAGACTTAGCTGCTATTGAAGCTCCATCTACAAATGGATAAGCCTTTG  83
             ||||||||||||||||||||||||||||||||||||||||||| |||||||||| |||||
Sbjct  1     ATAATTTCCCTCTAGACTTAGCTGCTATTGAAGCTCCATCTACCAATGGATAAGACTTTG  60

Query  84    GTCTGATTGTATAGGAGTTTTTGAACTAAAAAAGGAGCAATAAC-GCCCTCTTGATAAAA  142
             |||||||||||||||||||||||||||||||||||||||||| |  |||||||| |  | 
Sbjct  61    GTCTGATTGTATAGGAGTTTTTGAACTAAAAAAGGAGCAATAGCTTCCCTCTTGTTTTAT  120

Query  143   CAAGAGGGAAGCTATTGCTCCttttttATTTAGTACTATTTGCCTTACATAGTTTCTTT  202
             ||||||||  | ||||||||||||||||||||||||||||||||||||||||||||||||
Sbjct  121   CAAGAGGG-CGTTATTGCTCCTTTTTTTATTTAGTACTATTTGCCTTACATAGTTTCTTT  179

Query  203   AAAAATAACAAGTACTTTTTATAGTTTGGTTCGatttgcgtgtttttctttgtattcat  262
             ||||||||||||||||||||||||||||||||||||||||||||||||||||||||||||
Sbjct  180   AAAAATAACAAGTACTTTTTATAGTTTGGTTCGATTTGCGTGTTTTTCTTTGTATTCAT  239

Query  263   ttatattataggtttctatatcatttatttttcccaatcttttatgaagttttatttaCA  322
             ||||||||||||||| |||||||| || ||||||||||||||||||||||||||||||||
Sbjct  240   TTATATTATAGGTTTATATATCATATACTTTTCCCAATCTTTTATGAAGTTTTATTTACA  299

Query  323   ATTTAAATTTCAATCGaaaaaaagagaaaaaatgacaaattttgctttttttgattttata  382
             ||| || || ||||| ||||| ||| ||||| ||| ||||||||||| |||  ||| || 
Sbjct  300   ATTCAAGTT-CAATC-AAAAATAGATAAAAA-TGA-AAATTTTGCTTATTT--TTT-AT-  351

Query  383   ttgattaaaatcaaaaataa-aga-aaagaattttgctttttttttATTGTTAAATGGaaa  440
             || |||||| | |||||||| | | ||| |||  ||||||||||||| ||||| ||||||
Sbjct  352   TT-ATTAAA-T-AAAAATAAGAAAGAAATAATA-TGCTTTTTTTTAT-GTTAA-TGGAAA  405

Query  441   aaaaTATAGTTAATACTAGATAATCCGTAGAAGGGGGCGGATGTAGGCCAAAGTGGATC  500
             ||  ||||| ||||||||||||||  ||||| |||| ||| |||||| |||| |||||||
Sbjct  406   AAT-TATAG-TAATACTAGATAATA-GTAGA-GGGG-CGG-ATGTAG-CCAA-GTGGATC  457

Query  501   AAGG  504
             ||||
Sbjct  458   AAGG  461
```

 Score = 110 bits (59), Expect = 3e-19
 Identities = 59/59 (100%), Gaps = 0/59 (0%)
 Strand=Plus/Minus

```
Query  111   AAAAAAGGAGCAATAACGCCCTCTTGATAAAACAAGAGGGAAGCTATTGCTCCtttttt  169
             |||||||||||||||||||||||||||||||||||||||||||||||||||||||||||
Sbjct  146   AAAAAAGGAGCAATAACGCCCTCTTGATAAAACAAGAGGGAAGCTATTGCTCCTTTTTT  88
```

```
>MT793838.1 Mikania lehmannii chloroplast, complete genome
Length=152062

 Score = 593 bits (321),  Expect = 7e-165
 Identities = 456/514 (89%), Gaps = 37/514 (7%)
 Strand=Plus/Minus

Query  2    GTTATGCATGAACGTAATGCTCATAATTTCCCTCTAGACTTAGCTGCTATTGAAGCTCCA  61
            ||||||||||||||||||||||||||||||||||||||||||||||||||||||||||||
Sbjct  536  GTTATGCATGAACGTAATGCTCATAATTTCCCTCTAGACTTAGCTGCTATTGAAGCTCCA  477

Query  62   TCTACAAATGGATAAGCCTTTGGTCTGATTGTATAGGAGTTTTTGAACTAAAAAAGGAGC  121
            |||||||||||||||| |||||||||||||||||||||||||||||||||||||||||||
Sbjct  476  TCTACAAATGGATAAGACTTTGGTCTGATTGTATAGGAGTTTTTGAACTAAAAAAGGAGC  417

Query  122  AATAACGCCCTCTTGATAAAACAAGAGGGAAGCTATTGCTCCtttttttATTTAGTACTA  181
            |||||||||||||||||| |||||||||||||||||||||||||||||||||||||||||
Sbjct  416  AATAACGCCCTCTTGATACAACAAGAGGGAAGCTATTGCTCCTTTTTTTATTTAGTACTA  357

Query  182  TTTGCCTTACATAGTTTCTTTAAAAATAAC--AAG--T-------ACTTTTTATAGTTTG  230
            ||||||||||||||||||||||||||||||  ||   |       |||||||||||||||
Sbjct  356  TTTGCCTTACATAGTTTCTTTAAAAATAACTTAAAAATAACAAGGACTTTTTATAGTTTG  297

Query  231  GTTCGatttgcgtgtttttctttgtattcatttatattataggtttctatatcatttat  290
            ||||||||||||||||||||||||||||||||||||||||||||||||||||| || |||
Sbjct  296  GTTCGATTTGCGTGTTTTTTCTTTGTATTCATTTATATTATAGGTTTCTATATAATATAT  237

Query  291  ttttcccaatcttttatgaagttttatttaCAATTTAAATTTCAATCGaaaaaaagagaa  350
            |||||||||||||||||||||||| |||| ||||| || ||||||||||||| | ||  |
Sbjct  236  TTTTCCCAATCTTTTATGAAGTTTGATTTCCAATTCAA-TTTCAATCGAAAATA-GATCA  179

Query  351  aaaatgacaaattttgcttttttgattttatattgattaaaatcaaaaataaagaaaaga  410
            ||| ||| ||||||||||| ||| |||   | || |||    ||  |||||| ||||  |
Sbjct  178  AAA-TGA-AAATTTTGCTTATTT-ATTACTT-TT-ATT----TCTTAAATAA-GAAAT-A  130

Query  411  attttgcttttttttATTGTTAAATGGaaaaaaaTATAGTTAATACTAGATAATCCGTAG  470
            || | | ||||||| || ||||| ||||||||  ||||| ||||||||||||||  ||||
Sbjct  129  ATAT-G-TTTTTTTGAT-GTTAA-TGGAAAAA--TATAG-TAATACTAGATAATA-GTAG  78

Query  471  AAGGGGGCGGGATGTAGGCCAAAGTGGATCAAGG  504
            | |||| ||| |||||| |||| |||||||||||
Sbjct  77   A-GGGG-CGG-ATGTAG-CCAA-GTGGATCAAGG  49


>MT793855.1 Mikania sylvatica chloroplast, complete genome
Length=152099

 Score = 593 bits (321),  Expect = 7e-165
 Identities = 456/514 (89%), Gaps = 38/514 (7%)
 Strand=Plus/Minus

Query  2    GTTATGCATGAACGTAATGCTCATAATTTCCCTCTAGACTTAGCTGCTATTGAAGCTCCA  61
            ||||||||||||||||||||||||||||||||||||||||||||||||||||||||||||
Sbjct  535  GTTATGCATGAACGTAATGCTCATAATTTCCCTCTAGACTTAGCTGCTATTGAAGCTCCA  476
```

```
Query  62   TCTACAAATGGATAAGCCTTTGGTCTGATTGTATAGGAGTTTTTGAACTAAAAAAGGAGC  121
            |||||||||||||||| |||||||||||||||||||||||||||||||||||||||||||
Sbjct  475  TCTACAAATGGATAAGACTTTGGTCTGATTGTATAGGAGTTTTTGAACTAAAAAAGGAGC  416

Query  122  AATAACGCCCTCTTGATAAAACAAGAGGGAAGCTATTGCTCCtttttttATTTAGTACTA  181
            |||||||||||||||||| |||||||||||||||||||||||||||||||||||||||||
Sbjct  415  AATAACGCCCTCTTGATACAACAAGAGGGAAGCTATTGCTCCTTTTTTTATTTAGTACTA  356

Query  182  TTTGCCTTACATAGTTTCTTTAAAAATAAC-----------AAGTACTTTTTATAGTTTG  230
            ||||||||||||||||||||||||||||||           ||| |||||||||||||||
Sbjct  355  TTTGCCTTACATAGTTTCTTTAAAAATAACTTAAAAAAAACAAGGACTTTTTATAGTTTG  296

Query  231  GTTCGatttgcgtgtttttctttgtattcatttatattataggtttctatatcatttat  290
            ||||||||||||||||||||||||||||||||||||||||||||||||||||| || |||
Sbjct  295  GTTCGATTTGCGTGTTTTTCTTTGTATTCATTTATATTATAGGTTTCTATATAATATAT  236

Query  291  ttttcccaatcttttatgaagttttatttaCAATTTAAATTTCAATCGaaaaaaagagaa  350
            |||||||||||||||||||||||| |||| ||||| || ||||||||||||| | ||  |
Sbjct  235  TTTTCCCAATCTTTTATGAAGTTTGATTTCCAATTCAA-TTTCAATCGAAAATA-GATCA  178

Query  351  aaaatgacaaattttgcttttttgattttatattgattaaaatcaaaaataaagaaaaga  410
            ||| ||| ||||||||||| ||| |||   | || ||| |  | |||  ||| ||||  |
Sbjct  177  AAA-TGA-AAATTTTGCTTATTT-ATTACTT-TT-ATTTAT-T-AAA--TAA-GAAAT-A  129

Query  411  atttgcttttttttATTGTTAAATGGaaaaaaaTATAGTTAATACTAGATAATCCGTAG  470
            || | | ||||||| || ||||| ||||||||  ||||| ||||||||||||||  ||||
Sbjct  128  ATAT-G-TTTTTTT-AT-GTTAA-TGGAAAAA--TATAG-TAATACTAGATAATA-GTAG  78

Query  471  AAGGGGGCGGGATGTAGGCCAAAGTGGATCAAGG  504
            | |||| ||| |||||| |||| |||||||||||
Sbjct  77   A-GGGG-CGG-ATGTAG-CCAA-GTGGATCAAGG  49


>MF037493.1 Echinacea paradoxa voucher Rebman1085 trnH-psbA intergenic spacer
region, partial sequence; chloroplast
Length=429

 Score = 592 bits (320),  Expect = 3e-164
 Identities = 351/364 (96%), Gaps = 10/364 (3%)
 Strand=Plus/Plus

Query  45   CTGCTATTGAAGCTCCATCTACAAATGGATAAGCCTTTGGTCTGATTGTATAGGAGTTTT  104
            ||||||||||||||||||||||||||||||||||||||||||||||||||||||||||||
Sbjct  1    CTGCTATTGAAGCTCCATCTACAAATGGATAAGCCTTTGGTCTGATTGTATAGGAGTTTT  60

Query  105  TGAACTAAAAAAGGAGCAATAACGCCCTCTTGATAAAACAAGAGGGAAGCTATTGCTCC-  163
            ||||||||||||||||||||||||||||||||||||||||||||||||||||||||||| 
Sbjct  61   TGAACTAAAAAAGGAGCAATAACGCCCTCTTGATAAAACAAGAGGGAAGCTATTGCTCCT  120

Query  164  tttttttATTTAGTACTATTTGCCTTACATAGTTTCTTTAAAAATAACAAGTACTTTTTA  223
            ||||||||||||||||||||||||||||||||||||||||||||||||||||||||||||
Sbjct  121  TTTTTTTATTTAGTACTATTTGCCTTACATAGTTTCTTTAAAAATAACAAGTACTTTTTA  180

Query  224  TAGTTTGGTTCGatttgcgtgtttttctttgtattcatttatattataggtttctatat  283
            ||||||||||||||||||||||||||||||||||||||||||||||||||||||||||||
Sbjct  181  TAGTTTGGTTCGATTTGCGTGTTTTTCTTTGTATTCATTTATATTATAGGTTTCTATAT  240
```

```
Query  284  catttattttcccaatcttttatgaagttttatttaCAATTTAAATTTCAATCGaaaaa  343
            ||| ||||||||||||||||||||||||||||||||||||||| |||||||||||||||
Sbjct  241  CATATATTTTTCCCAATCTTTTATGAAGTTTTATTTACAATTT-AATTTCAATCGAAAAT  299

Query  344  aagagaaaaaatgacaaattttgctttttgattttatattgattaaaatcaaaaataaa  403
            | |||||||| |||||| ||||||||||| || |||||||||||||||  ||||| |||
Sbjct  300  A-GAGAAAAA-TGACAA-TTTTGCTTTTT-GA-TTTATATTGATTAAAG-CAAAA-TAA-  351

Query  404  gaaa  407
            ||||
Sbjct  352  GAAA  355
```

>NC_053285.1 Aldama linearis voucher Schilling 70 chloroplast, complete genome
 MN337896.1 Aldama linearis chloroplast, complete genome
Length=151314

 Score = 590 bits (319), Expect = 9e-164
 Identities = 460/520 (88%), Gaps = 41/520 (8%)
 Strand=Plus/Minus

```
Query  2    GTTATGCATGAACGTAATGCTCATAATTTCCCTCTAGACTTAGCTGCTATTGAAGCTCCA  61
            ||||||||||||||||||||||||||||||||||||||||||||||||||||||||||||
Sbjct  547  GTTATGCATGAACGTAATGCTCATAATTTCCCTCTAGACTTAGCTGCTATTGAAGCTCCA  488

Query  62   TCTACAAATGGATAAGCCTTTGGTCTGATTGTATAGGAGTTTTTGAACTAAAAAAGGAGC  121
            |||||||||||||||| |||||||||||||||||||||||||||||||||||||||||||
Sbjct  487  TCTACAAATGGATAAGACTTTGGTCTGATTGTATAGGAGTTTTTGAACTAAAAAAGGAGC  428

Query  122  AATAACGCCCTCTTGATAAAACAAGAGGGAAGCTATTGCTCCttttttt-ATTTAGTACT  180
            ||||||||||||||| |  | |||||||||||||||||||||||||||| ||||||||||
Sbjct  427  AATAACGCCCTCTTGTTTTATCAAGAGGGAAGCTATTGCTCCTTTTTTTTATTTAGTACT  368

Query  181  ATTTGCCTTACATAGTTTCTTTAAA-------AATAACAAGTACTTTTTATAGTTTGGTT  233
            |||||||||||||||||||||||||       ||||||||||||||||||||||||||||
Sbjct  367  ATTTGCCTTACATAGTTTCTTTAAACTTTAAAAATAACAAGTACTTTTTATAGTTTGGTT  308

Query  234  CGatttgcgtgtttttctttgtattcatttatattataggtttctatatcatttatttt  293
            |||||||  |||||||| ||||||||||||||||||||||||||||||||||| || |||
Sbjct  307  CGATTTGTTTGTTTTTTATTTGTATTCATTTATATTATAGGTTTCTATATCATATACTTT  248

Query  294  t-c----cc-aatcttttatgaagttttatttaCAATTTAAATTTCAATCGaaaaaaaga  347
            | |    || |||||||||||||||||| |||||||||| |||| |||||||||| | ||
Sbjct  247  TACTTTTCCTAATCTTTTATGAAGTTTTTTTTACAATTTCAATT-CAATCGAAAATA-GA  190

Query  348  gaaaaaatgacaaattttgctt-ttt-tga-ttttatattgattaaaatcaaaaataaag  404
             ||||| |||||| |||||||| ||| | | |||||| || ||| || | |||||| | |
Sbjct  189  TAAAAA-TGACAA-TTTTGCTTATTTATTACTTTTAT-TT-ATTCAA-T-AAAAATGA-G  137

Query  405  aaaagaattttgcttttttttATTGTTAAATGGaaaaaaaTATAGTTAATACTAGATAAT  464
            ||| |||| | ||||||||  || ||||| ||||||||  ||||| ||||||||||||||
Sbjct  136  AAA-GAATAT-GCTTTTTT--AT-GTTAA-TGGAAAAA--TATAG-TAATACTAGATAAT  86

Query  465  CCGTAGAAGGGGGCGGGATGTAGGCCAAAGTGGATCAAGG  504
              ||||| |||| ||| |||||| |||| |||||||||||
Sbjct  85   A-GTAGA-GGGG-CGG-ATGTAG-CCAA-GTGGATCAAGG  52
```

```
>NC_060408.1 Silphium perfoliatum chloroplast, complete sequence
 MN445187.1 Silphium perfoliatum chloroplast, complete genome
Length=151924

 Score = 588 bits (318),  Expect = 3e-163
 Identities = 463/526 (88%), Gaps = 38/526 (7%)
 Strand=Plus/Minus

Query  2    GTTATGCATGAACGTAATGCTCATAATTTCCCTCTAGACTTAGCTGCTATTGAAGCTCCA  61
            ||||||||||||||||||||||||||||||||||||||||||||||||||||||||||||
Sbjct  563  GTTATGCATGAACGTAATGCTCATAATTTCCCTCTAGACTTAGCTGCTATTGAAGCTCCA  504

Query  62   TCTACAAATGGATAAGCCTTTGGTCTGATTGTATAGGAGTTTTTGAACTAAAAAAGGAGC  121
            |||||||||||||||| |||||||||||||||||||||||||||||||||||||||||||
Sbjct  503  TCTACAAATGGATAAGACTTTGGTCTGATTGTATAGGAGTTTTTGAACTAAAAAAGGAGC  444

Query  122  AATAAC-GCCCTCTTGATAAAACAAGAGGGAAGCTATTGCTCCttttttt-ATTTAGTAC  179
            |||| |  |||||||| |  | ||||||||  | |||||||||||||||| |||||||||
Sbjct  443  AATAGCTTCCCTCTTGTTTTATCAAGAGGG-CGTTATTGCTCCTTTTTTTATTTAGTAC  385

Query  180  TATTTGCCTTACATAGTTTCTTTAAAAATAACAAGTACTTTTTATAGTTTGGTTCGattt  239
            ||||||||||||||||||||||||||||||||||||||||||||||||||||||||||||
Sbjct  384  TATTTGCCTTACATAGTTTCTTTAAAAATAACAAGTACTTTTTATAGTTTGGTTCGATTT  325

Query  240  gcgtgttttttctttgtattcatttatattataggtttctatatcatttatttttcccaa  299
            ||||||||||||||||||||||||||||||||||||||||||||||| || ||||||| |
Sbjct  324  GCGTGTTTTTTCTTTGTATTCATTTATATTATAGGTTTCTATATCATATACTTTTCCCGA  265

Query  300  tcttttatgaagttttatttaCAATTTAAATTTCAATCGaaaaaaagagaaaaaatgaca  359
            ||||||||||||||| |||||||||| || ||||||||||||| | || ||||| | |||
Sbjct  264  TCTTTTATGAAGTTTGATTTACAATTCAA-TTTCAATCGAAAATA-GATAAAAATT-ACA  208

Query  360  aattttgctt-ttt-tga-ttttat--attgattaaaa-tca-----aaaataaaga-aa  407
            | |||||||| ||| | | ||||||  ||| | ||||| | |     |||||||| | ||
Sbjct  207  A-TTTTGCTTATTTATTACTTTTATTTATTAAATAAAAATGATTTTTAAAATAAAAATAA  149

Query  408  -a--gaatt--t-tgcttttttttATTGTTAAATGGaaaaa-aa--TATAGTTAATACTA  458
             |  ||| |  | |||||||||| || ||||| || ||||| ||  ||||| ||||||||
Sbjct  148  GAAAGAAATAATATGCTTTTTTT-AT-GTTAA-TGAAAAAATAAAATATAG-TAATACTA  93

Query  459  GATAATCCGTAGAAGGGGGCGGGATGTAGGCCAAAGTGGATCAAGG  504
            ||||||  ||||| |||| ||| |||||| |||| |||||||||||
Sbjct  92   GATAATA-GTAGA-GGGG-CGG-ATGTAG-CCAA-GTGGATCAAGG  53


 Score = 110 bits (59),  Expect = 3e-19
 Identities = 59/59 (100%), Gaps = 0/59 (0%)
 Strand=Plus/Plus

Query  111  AAAAAAGGAGCAATAACGCCCTCTTGATAAAACAAGAGGGAAGCTATTGCTCCtttttt  169
            |||||||||||||||||||||||||||||||||||||||||||||||||||||||||||
Sbjct  396  AAAAAAGGAGCAATAACGCCCTCTTGATAAAACAAGAGGGAAGCTATTGCTCCTTTTTT  454


>NC_081944.1 Venegasia carpesioides voucher US Weitzman et al. 412 chloroplast,
complete genome
```

```
 OR069732.1 Venegasia carpesioides voucher US Weitzman et al. 412 chloroplast,
complete genome
Length=152212

 Score = 586 bits (317),  Expect = 1e-162
 Identities = 459/521 (88%), Gaps = 35/521 (7%)
 Strand=Plus/Minus

Query  2    GTTATGCATGAACGTAATGCTCATAATTTCCCTCTAGACTTAGCTGCTATTGAAGCTCCA  61
            ||||||||||||||||||||||||||||||||||||||||||||||||||||||||||||
Sbjct  558  GTTATGCATGAACGTAATGCTCATAATTTCCCTCTAGACTTAGCTGCTATTGAAGCTCCA  499

Query  62   TCTACAAATGGATAAGCCTTTGGTCTGATTGTATAGGAGTTTTTGAACTAAAAAAGGAGC  121
            |||||||||||||||| |||||||||||||||||||||||||||||||||||||||||||
Sbjct  498  TCTACAAATGGATAAGACTTTGGTCTGATTGTATAGGAGTTTTTGAACTAAAAAAGGAGC  439

Query  122  AATAACG-CCCTCTTGATAAAACAAGAGGGAAGCTATTGCTCC-tttttttATTTAGTAC  179
            |||| |  |||||||| |  | ||||||||  ||||||||||| ||||||||||||||||
Sbjct  438  AATAGCTTCCCTCTTGTTTTATCAAGAGGGC-GCTATTGCTCCTTTTTTTTATTTAGTAC  380

Query  180  TATTTGCCTTACATAGTTTCTTTAAAAATAACAAGTACTTTTTATAGTTTGGTTCGattt  239
            ||||||||||||||||||||||||||||||||||| ||||||| ||||||||||||||||
Sbjct  379  TATTTGCCTTACATAGTTTCTTTAAAAATAACAAGGACTTTTTCTAGTTTGGTTCGATTT  320

Query  240  gcgtgttttttctttgtattcatttatattataggtttctatatcatttattttcccaa  299
            ||||||||||||||||||||||||||||||||||||||||||||||| || |||||||||
Sbjct  319  GCGTGTTTTTTCTTTGTATTCATTTATATTATAGGTTTCTATATCATATACTTTTCCCAA  260

Query  300  tcttttatgaagttttatttaCAATTTAAATTTCAATCGaaaaaaagagaaaaaatgaca  359
            ||||||||||||||| |||| ||||| || ||||||||||||| | || ||||| | |||
Sbjct  259  TCTTTTATGAAGTTTGATTTCCAATTCAA-TTTCAATCGAAAATA-GATAAAAATT-ACA  203

Query  360  aattttgctt-ttt-tga-ttttat--attgattaa-aatcaaa-aata---aag-aa-a  407
            | |||||||| ||| | | ||||||  ||| | ||| ||  ||| ||||   ||  || |
Sbjct  202  A-TTTTGCTTATTTATTACTTTTATTTATTAAATAAGAAAGAAATAATATGTAAATAAGA  144

Query  408  a-gaatt--t-tgcttttttttATTGTTAAATGGaaaaaaaTATAGTTAATACTAGATAA  463
            | ||| |  | |||||||||| || ||||| ||||||||  ||||| |||||||||| ||
Sbjct  143  AAGAAATAATATGCTTTTTTT-AT-GTTAA-TGGAAAAA--TATAG-TAATACTAGAGAA  90

Query  464  TCCGTAGAAGGGGGCGGGATGTAGGCCAAAGTGGATCAAGG  504
            |  ||||| |||| ||| |||||| |||| |||||||||||
Sbjct  89   TA-GTAGA-GGGG-CGG-ATGTAG-CCAA-GTGGATCAAGG  55


 Score = 104 bits (56),  Expect = 1e-17
 Identities = 58/59 (98%), Gaps = 0/59 (0%)
 Strand=Plus/Plus

Query  111  AAAAAAGGAGCAATAACGCCCTCTTGATAAAACAAGAGGGAAGCTATTGCTCCtttttt  169
            ||||||||||||||| |||||||||||||||||||||||||||||||||||||||||||
Sbjct  391  AAAAAAGGAGCAATAGCGCCCTCTTGATAAAACAAGAGGGAAGCTATTGCTCCTTTTTT  449


>OZ286192.1 Venegasia carpesioides genome assembly, organelle: plastid
Length=152266
```

```
 Score = 586 bits (317),  Expect = 1e-162
 Identities = 459/521 (88%), Gaps = 35/521 (7%)
 Strand=Plus/Minus

Query  2    GTTATGCATGAACGTAATGCTCATAATTTCCCTCTAGACTTAGCTGCTATTGAAGCTCCA  61
            ||||||||||||||||||||||||||||||||||||||||||||||||||||||||||||
Sbjct  558  GTTATGCATGAACGTAATGCTCATAATTTCCCTCTAGACTTAGCTGCTATTGAAGCTCCA  499

Query  62   TCTACAAATGGATAAGCCTTTGGTCTGATTGTATAGGAGTTTTTGAACTAAAAAAGGAGC  121
            |||||||||||||||| |||||||||||||||||||||||||||||||||||||||||||
Sbjct  498  TCTACAAATGGATAAGACTTTGGTCTGATTGTATAGGAGTTTTTGAACTAAAAAAGGAGC  439

Query  122  AATAACG-CCCTCTTGATAAAACAAGAGGGAAGCTATTGCTCC-ttttttATTTAGTAC  179
            |||| |  |||||||| |  | ||||||||  ||||||||||| |||||||||||||||
Sbjct  438  AATAGCTTCCCTCTTGTTTTATCAAGAGGGC-GCTATTGCTCCTTTTTTTATTTAGTAC  380

Query  180  TATTTGCCTTACATAGTTTCTTTAAAAATAACAAGTACTTTTTATAGTTTGGTTCGattt  239
            |||||||||||||||||||||||||||||||||||| ||||||| |||||||||||||||
Sbjct  379  TATTTGCCTTACATAGTTTCTTTAAAAATAACAAGGACTTTTTCTAGTTTGGTTCGATTT  320

Query  240  gcgtgtttttctttgtattcatttatattataggtttctatatcatttatttttcccaa  299
            ||||||||||||||||||||||||||||||||||||||||||||| || |||||||||
Sbjct  319  GCGTGTTTTTCTTTGTATTCATTTATATTATAGGTTTCTATATCATATACTTTTCCCAA  260

Query  300  tcttttatgaagttttatttaCAATTTAAATTTCAATCGaaaaaaagagaaaaaaatgaca  359
            ||||||||||||||| |||| ||||| || ||||||||||||| | || ||||| | |||
Sbjct  259  TCTTTTATGAAGTTTGATTTCCAATTCAA-TTTCAATCGAAAATA-GATAAAAATT-ACA  203

Query  360  aattttgctt-ttt-tga-ttttat--attgattaa-aatcaaa-aata---aag-aa-a  407
            | |||||||| ||| | | ||||||  ||| | ||| ||  ||| ||||    ||  || |
Sbjct  202  A-TTTTGCTTATTTATTACTTTTATTTATTAAATAAGAAAGAAATAATATGTAAATAAGA  144

Query  408  a-gaatt--t-tgcttttttttATTGTTAAATGGaaaaaaaTATAGTTAATACTAGATAA  463
            | ||| |  | |||||||||| || ||||| ||||||||  ||||| |||||||||| ||
Sbjct  143  AAGAAATAATATGCTTTTTTT-AT-GTTAA-TGGAAAAA--TATAG-TAATACTAGAGAA  90

Query  464  TCCGTAGAAGGGGGCGGGATGTAGGCCAAAGTGGATCAAGG  504
            |  ||||| |||| ||| |||||| |||| |||||||||||
Sbjct  89   TA-GTAGA-GGGG-CGG-ATGTAG-CCAA-GTGGATCAAGG  55


 Score = 104 bits (56),  Expect = 1e-17
 Identities = 58/59 (98%), Gaps = 0/59 (0%)
 Strand=Plus/Plus

Query  111  AAAAAAGGAGCAATAACGCCCTCTTGATAAAACAAGAGGGAAGCTATTGCTCCtttttt  169
            ||||||||||||||| |||||||||||||||||||||||||||||||||||||||||||
Sbjct  391  AAAAAAGGAGCAATAGCGCCCTCTTGATAAAACAAGAGGGAAGCTATTGCTCCTTTTTT  449


>NC_057172.1 Aldama excelsa voucher Schilling H2481 chloroplast, complete
genome
 MN356026.1 Aldama excelsa chloroplast, complete genome
Length=151312

 Score = 584 bits (316),  Expect = 4e-162
 Identities = 461/523 (88%), Gaps = 42/523 (8%)
```

```
 Strand=Plus/Minus

Query  2    GTTATGCATGAACGTAATGCTCATAATTTCCCTCTAGACTTAGCTGCTATTGAAGCTCCA  61
            ||||||||||||||||||||||||||||||||||||||||||||||||||||||||||||
Sbjct  558  GTTATGCATGAACGTAATGCTCATAATTTCCCTCTAGACTTAGCTGCTATTGAAGCTCCA  499

Query  62   TCTACAAATGGATAAGCCTTTGGTCTGATTGTATAGGAGTTTTTGAACTAAAAAAGGAGC  121
            |||||||||||||||| |||||||||||||||||||||||||||||||||||||||||||
Sbjct  498  TCTACAAATGGATAAGACTTTGGTCTGATTGTATAGGAGTTTTTGAACTAAAAAAGGAGC  439

Query  122  AATAACGCCCTCTTGATAAAACAAGAGGGAAGCTATTGCTCCttttttt--ATTTAGTAC  179
            ||||||||||||||| |  | ||||||||||||||||||||||||||||  |||||||||
Sbjct  438  AATAACGCCCTCTTGTTTTATCAAGAGGGAAGCTATTGCTCCTTTTTTTTTATTTAGTAC  379

Query  180  TATTTGCCTTACATAGTTTCTTTAAA-------AATAACAAGTACTTTTTATAGTTTGGT  232
            ||||||||||||||||||||||||||       |||||||||||||||||||||||||||
Sbjct  378  TATTTGCCTTACATAGTTTCTTTAAACTTTAAAAATAACAAGTACTTTTTATAGTTTGGT  319

Query  233  TCGatttgcgtgttttttctttgtattcatttatattataggtttctatatcatttattt  292
            ||||||||  |||||||| ||||||||||||||||||||||||||||||||||| || ||
Sbjct  318  TCGATTTGTTTGTTTTTTATTTGTATTCATTTATATTATAGGTTTCTATATCATATACTT  259

Query  293  tt-c----cc-aatcttttatgaagttttatttaCAATTTAAATTTCAATCGaaaaaaag  346
            || |    || ||||||||||||||||| ||||||||||| |||| |||||||||| | |
Sbjct  258  TTACTTTTCCTAATCTTTTATGAAGTTTTTTTTACAATTTCAATT-CAATCGAAAATA-G  201

Query  347  agaaaaaatgacaaattttgctt-ttt-tga-ttttat--attgattaaaatcaaaaata  401
            | ||||| |||||| |||||||| ||| | | ||||||  ||| ||| || | ||||||
Sbjct  200  ATAAAAA-TGACAA-TTTTGCTTATTTATTACTTTTATTTATTTATTCAA-T-AAAAATG  145

Query  402  aagaaaagaattttgcttttttttATTGTTAAATGGaaaaaaaTATAGTTAATACTAGAT  461
            | |||| |||| | ||||||||  || ||||| ||||||||  ||||| |||||||||||
Sbjct  144  A-GAAA-GAATAT-GCTTTTTT--AT-GTTAA-TGGAAAAA--TATAG-TAATACTAGAT  95

Query  462  AATCCGTAGAAGGGGGCGGGATGTAGGCCAAAGTGGATCAAGG  504
            |||  |||||||||  ||| |||||| |||| |||||||||||
Sbjct  94   AATA-GTAGAAGGG--CGG-ATGTAG-CCAA-GTGGATCAAGG  58
```

>OK469409.1 Eupatorieae sp. Gostel 807 voucher BRIT:Gostel807 photosystem II protein D1 (psbA) gene, partial cds; psbA-trnH intergenic spacer, complete sequence; and tRNA-His (trnH) gene, partial sequence; chloroplast
Length=462

```
 Score = 584 bits (316),  Expect = 4e-162
 Identities = 433/484 (89%), Gaps = 29/484 (6%)
 Strand=Plus/Plus

Query  24   ATAATTTCCCTCTAGACTTAGCTGCTATTGAAGCTCCATCTACAAATGGATAAGCCTTTG  83
            |||||||||||||||||||||||||||||||||||||||||||||||||||||| |||||
Sbjct  1    ATAATTTCCCTCTAGACTTAGCTGCTATTGAAGCTCCATCTACAAATGGATAAGACTTTG  60

Query  84   GTCTGATTGTATAGGAGTTTTTGAACTAAAAAAGGAGCAATAACGCCCTCTTGATAAAAC  143
            ||||||||||||||||||||||||||||||||||||||||||||||||||||||||||||
Sbjct  61   GTCTGATTGTATAGGAGTTTTTGAACTAAAAAAGGAGCAATAACGCCCTCTTGATAAAAC  120
```

```
Query  144  AAGAGGGAAGCTATTGCTCCtttttttATTTAGTACTATTTGCCTTACATAGTTTCTTTA  203
            ||||||||||||||||||||||||||||||||||||||||||||||| ||||||| ||||
Sbjct  121  AAGAGGGAAGCTATTGCTCCTTTTTTTATTTAGTACTATTTGCCTTATATAGTTTATTTA  180

Query  204  AAAATAACAAGTACTTTTTATAGTTTGGTTCGatttgcgtgtttttctttgtattcatt  263
            ||||||||||| |||||||||||||||||||||||||||| |||||||||||||||||||
Sbjct  181  AAAATAACAAGGACTTTTTATAGTTTGGTTCGATTTGCGTATTTTTCTTTGTATTCATT  240

Query  264  tatattataggtttctatatcatttatttttcccaatcttttatgaagttttatttaCAA  323
            |||||||||||||||||||| || || |||||||||||||||||||||||||||||  ||
Sbjct  241  TATATTATAGGTTTCTATATAATATACTTTTCCCAATCTTTTATGAAGTTTTATTTCGAA  300

Query  324  TTTAAATTTCAATCGaaaaaaagagaaaaaatgacaaattttgctt-ttt-tga-tttta  380
            ||  ||||||||||||||| | || ||||| | | ||||||| ||| ||| | | ||||
Sbjct  301  TTC-AATTTCAATCGAAAATA-GATAAAAATT-A-AAATTTTTCTTATTTATTACTTTTC  356

Query  381  tattgattaaaatcaaaaataaagaaaagaattttgcttttttttATTGTTAAATGGaaa  440
            | || |||||| | || || |  ||||  ||| | ||||||||  || ||||| ||||||
Sbjct  357  T-TT-ATTAAA-T-AAGAA-A--GAAAT-AATAT-GCTTTTTT--AT-GTTAA-TGGAAA  403

Query  441  aaaaTATAGTTAATACTAGATAATCCGTAGAAGGGGGCGGGATGTAGGCCAAAGTGGATC  500
            ||  || || ||||||||||||||  ||||| |||| ||| |||||| |||| |||||||
Sbjct  404  AA--TAGAG-TAATACTAGATAATA-GTAGA-GGGG-CGG-ATGTAG-CCAA-GTGGATC  454

Query  501  AAGG  504
            ||||
Sbjct  455  AAGG  458
```

>MZ636529.1 Xanthium spinosum chloroplast, complete genome
Length=151512

Score = 584 bits (316), Expect = 4e-162
Identities = 460/522 (88%), Gaps = 40/522 (8%)
Strand=Plus/Minus

```
Query  2    GTTATGCATGAACGTAATGCTCATAATTTCCCTCTAGACTTAGCTGCTATTGAAGCTCCA  61
            ||||||||||||||||||||||||||||||||||||||||||||||||||||||||||||
Sbjct  553  GTTATGCATGAACGTAATGCTCATAATTTCCCTCTAGACTTAGCTGCTATTGAAGCTCCA  494

Query  62   TCTACAAATGGATAAGCCTTTGGTCTGATTGTATAGGAGTTTTTGAACTAAAAAAGGAGC  121
            |||||||||||||||| |||||||||||||||||| ||||||||||||||||||||||||
Sbjct  493  TCTACAAATGGATAAGACTTTGGTCTGATTGTATAAGAGTTTTTGAACTAAAAAAGGAGC  434

Query  122  AATAACGCCCTCTTGATAAAACAAGAGGGAAGCTATTGCTCCttttttt-ATTTAGTACT  180
            ||||||||||||||||||||||||||||||||||||||||||||||||| ||||||||||
Sbjct  433  AATAACGCCCTCTTGATAAAACAAGAGGGAAGCTATTGCTCCTTTTTTTTATTTAGTACT  374

Query  181  ATTTGCCTTACATAGTTTCTTTAAAAATAACAAGTACTTTTTATAGTTTGGTTCGatttg  240
            |||||||||||||||||||||||||||| | ||||||||||||||||||| ||  |||||
Sbjct  373  ATTTGCCTTACATAGTTTCTTTAAAAATCAAAAGTACTTTTTATAGTTTGTTTTAATTTG  314

Query  241  cgtgttttttctttgtattcatttatattat--ag-----------gtttctatatcatt  287
            |||||||||||||||||||||||||||||||  |            |||||||||||||
Sbjct  313  CGTGTTTTTTCTTTGTATTCATTTATATTATTCATTTATATTATATGTTTCTATATCATA  254

Query  288  tatttttcccaatcttttatgaagttttatttaCAATTTAAATTTCAATCGaaaaaaaga  347
```

```
            || ||||||||||||||||||||||||||||||||||| |||||||||||||||| |  |
Sbjct  253  TACTTTTCCCAATCTTTTATGAAGTTTTATTTACAATT-AAATTTCAATCGAAAATAT-A  196

Query  348  gaaaaaatgacaaattttgcttttttgatt-ttat-attgattaaaatcaaaa-a-taaa  403
             ||||| | | ||||||||||||||| ||| || | ||| | |||| |||||| | ||||
Sbjct  195  TAAAAA-TCA-AAATTTTGCTTTTTT-ATTATTTTGATTTAATAAA-TCAAAATAATAAA  140

Query  404  gaaaagaattttgcttttttttATTGTTAAATGGaaaaaaaTATAGTTAATACTAGATA-  462
             |||| ||| | ||||||||| ||| |  |||||||||| ||| | | ||||||| ||| 
Sbjct  139  -AAAATAATAT-GCTTTTTTT-ATT-T--AATGGAAAAATATA-A-T-AATACTATATAT  89

Query  463  ATCCGTAGAAGGGGGCGGGATGTAGGCCAAAGTGGATCAAGG  504
            ||  ||||| |||| ||| |||||| |||| |||||||||||
Sbjct  88   ATA-GTAGA-GGGG-CGG-ATGTAG-CCAA-GTGGATCAAGG  53


>NC_072298.1 Xanthium strumarium chloroplast, complete genome
 OP606043.1 Xanthium strumarium chloroplast, complete genome
Length=151852

 Score = 584 bits (316),  Expect = 4e-162
 Identities = 460/522 (88%), Gaps = 40/522 (8%)
 Strand=Plus/Minus

Query  2    GTTATGCATGAACGTAATGCTCATAATTTCCCTCTAGACTTAGCTGCTATTGAAGCTCCA  61
            ||||||||||||||||||||||||||||||||||||||||||||||||||||||||||||
Sbjct  553  GTTATGCATGAACGTAATGCTCATAATTTCCCTCTAGACTTAGCTGCTATTGAAGCTCCA  494

Query  62   TCTACAAATGGATAAGCCTTTGGTCTGATTGTATAGGAGTTTTTGAACTAAAAAAGGAGC  121
            |||||||||||||||| |||||||||||||||||| ||||||||||||||||||||||||
Sbjct  493  TCTACAAATGGATAAGACTTTGGTCTGATTGTATAAGAGTTTTTGAACTAAAAAAGGAGC  434

Query  122  AATAACGCCCTCTTGATAAAACAAGAGGGAAGCTATTGCTCCttttttt-ATTTAGTACT  180
            ||||||||||||||||||||||||||||||||||||||||||||||||| ||||||||||
Sbjct  433  AATAACGCCCTCTTGATAAAACAAGAGGGAAGCTATTGCTCCTTTTTTTTATTTAGTACT  374

Query  181  ATTTGCCTTACATAGTTTCTTTAAAAATAACAAGTACTTTTTATAGTTTGGTTCGatttg  240
            |||||||||||||||||||||||||||||| ||||||||||||||||||| ||  |||||
Sbjct  373  ATTTGCCTTACATAGTTTCTTTAAAAATAAAAAGTACTTTTTATAGTTTGTTTTAATTTG  314

Query  241  cgtgttttttctttgtattcatttatattat--ag-----------gtttctatatcatt  287
            |||||||||||||||||||||||||||||||  |            ||||||||||||| 
Sbjct  313  CGTGTTTTTTCTTTGTATTCATTTATATTATTCATTTATATTATATGTTTCTATATCATA  254

Query  288  tatttttcccaatcttttatgaagttttatttaCAATTTAAATTTCAATCGaaaaaaaga  347
            || ||||||||||||||||||||||||||||||||||| |||||||||| ||||| |  |
Sbjct  253  TACTTTTCCCAATCTTTTATGAAGTTTTATTTACAATT-AAATTTCAATTGAAAATAT-A  196

Query  348  gaaaaaatgacaaattttgcttttttgatt-ttat-attgattaaaatcaaaa-a-taaa  403
             |||||  |  ||||||||||||||| ||| || | ||| |||||| |||||| | ||||
Sbjct  195  TAAAAA--GCAAAATTTTGCTTTTTT-ATTATTTTGATTTATTAAA-TCAAAATAATAAA  140

Query  404  gaaaagaattttgcttttttttATTGTTAAATGGaaaaaaaTATAGTTAATACTAGATA-  462
             |||| ||| | ||||||||| ||| |  |||||||||| ||| | | ||||||| ||| 
Sbjct  139  -AAAATAATAT-GCTTTTTTT-ATT-T--AATGGAAAAATATA-A-T-AATACTATATAT  89

Query  463  ATCCGTAGAAGGGGGCGGGATGTAGGCCAAAGTGGATCAAGG  504
```

```
            ||  ||||| |||| ||| |||||| |||| |||||||||||
Sbjct  88   ATA-GTAGA-GGGG-CGG-ATGTAG-CCAA-GTGGATCAAGG  53


>MF348479.1 Rudbeckia triloba isolate AD5JS71 photosystem II protein D1 (psbA)
gene, partial cds; and psbA-trnH intergenic spacer, partial
sequence; chloroplast
Length=479

 Score = 584 bits (316),  Expect = 4e-162
 Identities = 443/497 (89%), Gaps = 38/497 (8%)
 Strand=Plus/Plus

Query  24   ATAATTTCCCTCTAGACTTAGCTGCTATTGAAGCTCCATCTACAAATGGATAAGCCTTTG  83
            |||||||||||||||||||||||||||||||||||||||||||||||||||||| |||||
Sbjct  1    ATAATTTCCCTCTAGACTTAGCTGCTATTGAAGCTCCATCTACAAATGGATAAGACTTTG  60

Query  84   GTCTGATTGTATAGGAGTTTTTGAACTAAAAAAGGAGCAATAACGCCCTCTTG------A  137
            |||||||||||||| ||||||||||||||||||||||||||| ||||||||||      |
Sbjct  61   GTCTGATTGTATAGAAGTTTTTGAACTAAAAAAGGAGCAATAGCGCCCTCTTGATAAAAA  120

Query  138  TAAAACAAGAGGGAAGCTATTGCTCCttttttATTTAGTACTATTTGCCTTACATAGTT  197
            ||||||||||||||||||||||||||||||||||||||||||||||||| ||||||||||
Sbjct  121  TAAAACAAGAGGGAAGCTATTGCTCCTTTTTTTATTTAGTACTATTTGCTTTACATAGTT  180

Query  198  TCTTTAAAAATAACAAGTACTTTTTATAGTTTGGTTCGatttgcgtgtttttctttgta  257
            ||||||||||| ||||||||||||||||||||||||||||||||||||||||||||||||
Sbjct  181  TCTTTAAAAATGACAAGTACTTTTTATAGTTTGGTTCGATTTGCGTGTTTTTCTTTGTA  240

Query  258  ttca-----tttatattataggtttctatatcatttattttcccaatcttttatgaagt  312
            ||||     ||||||||||||||||||||||||| || ||||||||||||||||||||||
Sbjct  241  TTCATTTATTTTATATTATAGGTTTCTATATCATATACTTTTCCCAATCTTTTATGAAGT  300

Query  313  tttatttaCAATTTAAATTTCAATCGaaaaaaagagaaaaaatgacaaattttgctt-tt  371
            |||||||||||||  ||||||||||||||| | || ||||| ||| |||||||| || ||
Sbjct  301  TTTATTTACAATTC-AATTTCAATCGAAAATA-GATAAAAA-TGA-AAATTTTGATTATT  356

Query  372  t-tga-ttttatattgattaaaatcaaaaataa-aga-aaagaatttgctttttttttAT  427
            | | | |||||| || |||||| | |||||||| | | ||| ||| | ||||||||||
Sbjct  357  TATTACTTTTAT-TT-ATTAAA-T-AAAAATAAGAAAGAATAATAT-GCTTTTTTTTT--  409

Query  428  TGTTAAATGGaaaaaaaTATAGTTAATACTAGATAATCCGTAGAAGGGGGCGGGATGTAG  487
             ||||| ||||||||  ||||| ||||||||||||||  ||||| |||| ||| ||||||
Sbjct  410  -GTTAA-TGGAAAAA--TATAG-TAATACTAGATAATA-GTAGA-GGGG-CGG-ATGTAG  460

Query  488  GCCAAAGTGGATCAAGG  504
             |||| |||||||||||
Sbjct  461  -CCAA-GTGGATCAAGG  475


>NC_042232.1 Xanthium sibiricum chloroplast, complete genome
 MH473582.1 Xanthium sibiricum chloroplast, complete genome
Length=151897

 Score = 584 bits (316),  Expect = 4e-162
 Identities = 460/522 (88%), Gaps = 40/522 (8%)
 Strand=Plus/Minus
```

```
Query  2    GTTATGCATGAACGTAATGCTCATAATTTCCCTCTAGACTTAGCTGCTATTGAAGCTCCA  61
            ||||||||||||||||||||||||||||||||||||||||||||||||||||||||||||
Sbjct  553  GTTATGCATGAACGTAATGCTCATAATTTCCCTCTAGACTTAGCTGCTATTGAAGCTCCA  494

Query  62   TCTACAAATGGATAAGCCTTTGGTCTGATTGTATAGGAGTTTTTGAACTAAAAAAGGAGC  121
            |||||||||||||||| ||||||||||||||||| |||||||||||||||||||||||||
Sbjct  493  TCTACAAATGGATAAGACTTTGGTCTGATTGTATAAGAGTTTTTGAACTAAAAAAGGAGC  434

Query  122  AATAACGCCCTCTTGATAAAACAAGAGGGAAGCTATTGCTCCttttttt-ATTTAGTACT  180
            ||||||||||||||||||||||||||||||||||||||||||||||||| ||||||||||
Sbjct  433  AATAACGCCCTCTTGATAAAACAAGAGGGAAGCTATTGCTCCTTTTTTTTATTTAGTACT  374

Query  181  ATTTGCCTTACATAGTTTCTTTAAAAATAACAAGTACTTTTTATAGTTTGGTTCGatttg  240
            |||||||||||||||||||||||||||||| ||||||||||||||||||| ||  |||||
Sbjct  373  ATTTGCCTTACATAGTTTCTTTAAAAATAAAAAGTACTTTTTATAGTTTGTTTTAATTTG  314

Query  241  cgtgttttttctttgtattcatttatattat--ag-----------gtttctatatcatt  287
            |||||||||||||||||||||||||||||||  |            |||||||||||||
Sbjct  313  CGTGTTTTTTCTTTGTATTCATTTATATTATTCATTTATATTATATGTTTCTATATCATA  254

Query  288  tatttttcccaatcttttatgaagttttatttaCAATTTAAATTTCAATCGaaaaaaaga  347
            || ||||||||||||||||||||||||||||||||||| |||||||||| ||||| |  |
Sbjct  253  TACTTTTCCCAATCTTTTATGAAGTTTTATTTACAATT-AAATTTCAATTGAAAATAT-A  196

Query  348  gaaaaaatgacaaattttgcttttttgatt-ttat-attgattaaaatcaaaa-a-taaa  403
             |||||  |  ||||||||||||||| ||| || | ||| |||||| |||||| | ||||
Sbjct  195  TAAAAA--GCAAAATTTTGCTTTTTT-ATTATTTTGATTTATTAAA-TCAAAATAATAAA  140

Query  404  gaaaagaattttgcttttttttATTGTTAAATGGaaaaaaaTATAGTTAATACTAGATA-  462
             |||| ||| | ||||||||| ||| |  |||||||||| ||| | | ||||||| ||| 
Sbjct  139  -AAAATAATAT-GCTTTTTTT-ATT-T--AATGGAAAAATATA-A-T-AATACTATATAT  89

Query  463  ATCCGTAGAAGGGGGCGGGATGTAGGCCAAAGTGGATCAAGG  504
            ||  ||||| |||| ||| |||||| |||| |||||||||||
Sbjct  88   ATA-GTAGA-GGGG-CGG-ATGTAG-CCAA-GTGGATCAAGG  53
```

>KX346912.1 Xanthium sibiricum voucher S-CEZi-04 PsbA (psbA) gene, partial cds; and psbA-trnH intergenic spacer, partial sequence; chloroplast
Length=529

Score = 582 bits (315), Expect = 2e-161
Identities = 459/521 (88%), Gaps = 40/521 (8%)
Strand=Plus/Plus

```
Query  3    TTATGCATGAACGTAATGCTCATAATTTCCCTCTAGACTTAGCTGCTATTGAAGCTCCAT  62
            ||||||||||||||||||||||||||||||||||||||||||||||||||||||||||||
Sbjct  1    TTATGCATGAACGTAATGCTCATAATTTCCCTCTAGACTTAGCTGCTATTGAAGCTCCAT  60

Query  63   CTACAAATGGATAAGCCTTTGGTCTGATTGTATAGGAGTTTTTGAACTAAAAAAGGAGCA  122
            ||||||||||||||| ||||||||||||||||| ||||||||||||||||||||||||||
Sbjct  61   CTACAAATGGATAAGACTTTGGTCTGATTGTATAAGAGTTTTTGAACTAAAAAAGGAGCA  120

Query  123  ATAACGCCCTCTTGATAAAACAAGAGGGAAGCTATTGCTCC-tttttttATTTAGTACTA  181
            ||||||||||||||||||||||||||||||||||||||||| ||||||||||||||||||
Sbjct  121  ATAACGCCCTCTTGATAAAACAAGAGGGAAGCTATTGCTCCTTTTTTTTATTTAGTACTA  180
```

```
Query  182  TTTGCCTTACATAGTTTCTTTAAAAATAACAAGTACTTTTTATAGTTTGGTTCGatttgc  241
            ||||||||||||||||||||||||||||| ||||||||||||||||||| ||  ||||||
Sbjct  181  TTTGCCTTACATAGTTTCTTTAAAAATAAAAAGTACTTTTTATAGTTTGTTTTAATTTGC  240

Query  242  gtgttttttctttgtattcatttatattat--ag-----------gtttctatatcattt  288
            ||||||||||||||||||||||||||||||  |            ||||||||||||| |
Sbjct  241  GTGTTTTTTCTTTGTATTCATTTATATTATTCATTTATATTATATGTTTCTATATCATAT  300

Query  289  atttttcccaatcttttatgaagttttatttaCAATTTAAATTTCAATCGaaaaaaagag  348
            | ||||||||||||||||||||||||||||||||| |||||||||||| ||||| |  |
Sbjct  301  ACTTTTCCCAATCTTTTATGAAGTTTTATTTACAA-TTAAATTTCAATTGAAAATAT-AT  358

Query  349  aaaaaatgacaaattttgcttttttgatt-ttat-attgattaaaatcaaaa-a-taaag  404
            |||||  |  ||||||||||||||| ||| || | ||| |||||| |||||| | ||||
Sbjct  359  AAAAA--GCAAAATTTTGCTTTTTT-ATTATTTTGATTTATTAAA-TCAAAATAATAAA-  413

Query  405  aaaagaattttgcttttttttATTGTTAAATGGaaaaaaaTATAGTTAATACTAGATA-A  463
            |||| ||| | ||||||||| ||| |  |||||||||| ||| | | ||||||| ||| |
Sbjct  414  AAAATAATAT-GCTTTTTTT-ATT-T--AATGGAAAAATATA-A-T-AATACTATATATA  465

Query  464  TCCGTAGAAGGGGGCGGGATGTAGGCCAAAGTGGATCAAGG  504
            |  ||||| |||| ||| |||||| |||| |||||||||||
Sbjct  466  TA-GTAGA-GGGG-CGG-ATGTAG-CCAA-GTGGATCAAGG  500
```

>NC_081937.1 Greenmaniella resinosa voucher Baldwin 977 (UC) chloroplast, complete genome
OR069725.1 Greenmaniella resinosa voucher Baldwin 977 (UC) chloroplast, complete genome
Length=152246

Score = 582 bits (315), Expect = 2e-161
Identities = 452/513 (88%), Gaps = 30/513 (6%)
Strand=Plus/Minus

```
Query  2    GTTATGCATGAACGTAATGCTCATAATTTCCCTCTAGACTTAGCTGCTATTGAAGCTCCA  61
            ||||||||||||||||||||||||||||||||||||||||||||||||||||||||||||
Sbjct  549  GTTATGCATGAACGTAATGCTCATAATTTCCCTCTAGACTTAGCTGCTATTGAAGCTCCA  490

Query  62   TCTACAAATGGATAAGCCTTTGGTCTGATTGTATAGGAGTTTTTGAACTAAAAAAGGAGC  121
            |||||||||||||||| |||||||||||||||||||||||||||||||||||||||||||
Sbjct  489  TCTACAAATGGATAAGACTTTGGTCTGATTGTATAGGAGTTTTTGAACTAAAAAAGGAGC  430

Query  122  AATAACG-CCCTCTTGATAAAACAAGAGGGAAGCTATTGCTCCtttttttATTTAGTACT  180
            |||| |  |||||||| |  | ||||||||  | ||||||||||||||||||||||||||
Sbjct  429  AATAGCTTCCCTCTTGTTTTATCAAGAGGGC-GTTATTGCTCCTTTTTTTATTTAGTACT  371

Query  181  ATTTGCCTTACATAGTTTCTTTAAAAATAACAAGTACTTTTTATAGTTTGGTTCGatttg  240
            |||||||||||||||||||||||||||||||||| |||||||||||||||||||||||||
Sbjct  370  ATTTGCCTTACATAGTTTCTTTAAAAATAACAAGGACTTTTTATAGTTTGGTTCGATTTG  311

Query  241  cgtgttttttctttgtattcatttatattataggtttctatatcatttatttttcccaat  300
            |||||||| ||||||||||||||||||||||||||||||||||||| || ||||||||||
Sbjct  310  CGTGTTTTCTCTTTGTATTCATTTATATTATAGGTTTCTATATCATATACTTTTCCCAAT  251

Query  301  cttttatgaagttttatttaCAATTTAAATTTCAATCGaaaaaaagagaaaaaatgacaa  360
```

```
            ||||||||||||||||||| ||||| || ||||||||||||| | || ||||| | | ||
Sbjct  250  CTTTTATGAAGTTTTATTTCCAATTCAA-TTTCAATCGAAAATA-GATAAAAATT-A-AA  195

Query  361  attttgcttttttgattttatattgattaaa-atcaaa-aa-taa-a-ga-aaagaatt-  413
             |  |  ||| ||  ||| || || |||||| |  ||| || ||| | || |||||| | 
Sbjct  194  -TGCTTATTTATTACTTTGAT-TT-ATTAAATAAGAAAGAAATAATATGAGAAAGAAATA  138

Query  414  -t-tgcttttttttATTGTTAAATGGaaaaaaaTATAGTTAATACTAGATAATCCGTAGA  471
             | |||||||||| || ||||| ||||||||  | ||| ||||||||||||||  |||||
Sbjct  137  ATATGCTTTTTTT-AT-GTTAA-TGGAAAAA--TCTAG-TAATACTAGATAATA-GTAGA  85

Query  472  AGGGGGCGGGATGTAGGCCAAAGTGGATCAAGG  504
             |||| ||| |||||| |||| |||||||||||
Sbjct  84   -GGGG-CGG-ATGTAG-CCAA-GTGGATCAAGG  57


 Score = 110 bits (59),  Expect = 3e-19
 Identities = 59/59 (100%), Gaps = 0/59 (0%)
 Strand=Plus/Plus

Query  111  AAAAAAGGAGCAATAACGCCCTCTTGATAAAACAAGAGGGAAGCTATTGCTCCtttttt  169
            |||||||||||||||||||||||||||||||||||||||||||||||||||||||||||
Sbjct  382  AAAAAAGGAGCAATAACGCCCTCTTGATAAAACAAGAGGGAAGCTATTGCTCCTTTTTT  440


>NC_068130.1 Silphium integrifolium chloroplast, complete genome
 OM162161.1 Silphium integrifolium chloroplast, complete genome
Length=152058

 Score = 582 bits (315),  Expect = 2e-161
 Identities = 462/526 (88%), Gaps = 38/526 (7%)
 Strand=Plus/Minus

Query  2    GTTATGCATGAACGTAATGCTCATAATTTCCCTCTAGACTTAGCTGCTATTGAAGCTCCA  61
            ||||||||||||||||||||||||||||||||||||||||||||||||||||||||||||
Sbjct  560  GTTATGCATGAACGTAATGCTCATAATTTCCCTCTAGACTTAGCTGCTATTGAAGCTCCA  501

Query  62   TCTACAAATGGATAAGCCTTTGGTCTGATTGTATAGGAGTTTTTGAACTAAAAAAGGAGC  121
            |||||||||||||||| |||||||||||||||||||||||||||||||||||||||||||
Sbjct  500  TCTACAAATGGATAAGACTTTGGTCTGATTGTATAGGAGTTTTTGAACTAAAAAAGGAGC  441

Query  122  AATAAC-GCCCTCTTGATAAAACAAGAGGGAAGCTATTGCTCCttttttt-ATTTAGTAC  179
            |||| |  |||||||| |  | ||||||||  | |||||||||||||||| |||||||||
Sbjct  440  AATAGCTTCCCTCTTGTTTTATCAAGAGGG-CGTTATTGCTCCTTTTTTTTATTTAGTAC  382

Query  180  TATTTGCCTTACATAGTTTCTTTAAAAATAACAAGTACTTTTTATAGTTTGGTTCGattt  239
            ||||||||||||||||||||||||||||||||||||||||||||||||||||||||||||
Sbjct  381  TATTTGCCTTACATAGTTTCTTTAAAAATAACAAGTACTTTTTATAGTTTGGTTCGATTT  322

Query  240  gcgtgttttttctttgtattcatttatattataggtttctatatcatttatttttcccaa  299
            ||||||||||||||||||||||||||||||||||||||||||||||| || ||||||| |
Sbjct  321  GCGTGTTTTTTCTTTGTATTCATTTATATTATAGGTTTCTATATCATATACTTTTCCCGA  262

Query  300  tcttttatgaagttttatttaCAATTTAAATTTCAATCGaaaaaaagagaaaaaatgaca  359
            ||||||||||||||| |||||||||| || ||||||||||||| | || ||||| | |||
Sbjct  261  TCTTTTATGAAGTTTGATTTACAATTCAA-TTTCAATCGAAAATA-GATAAAAATT-ACA  205
```

```
Query  360  aattttgctt-ttt-tga-ttttat--attgattaaaa-tca----aaaa-taaaga-aa  407
            | |||||||| ||| | | ||||||  ||| | ||||| | |    |||| |||| | ||
Sbjct  204  A-TTTTGCTTATTTATTACTTTTATTTATTAAATAAAAATGATTTTAAAAATAAAAATAA  146

Query  408  -a--gaatt--t-tgcttttttttATTGTTAAATGGaaaaa-aa--TATAGTTAATACTA  458
             |  ||| |  | |||||||||| || ||||| || ||||| ||  ||||| ||||||||
Sbjct  145  GAAAGAAATAATATGCTTTTTTT-AT-GTTAA-TGAAAAAATAAAATATAG-TAATACTA  90

Query  459  GATAATCCGTAGAAGGGGGCGGGATGTAGGCCAAAGTGGATCAAGG  504
            ||||||  ||| | |||| ||| |||||| |||| |||||||||||
Sbjct  89   GATAATA-GTATA-GGGG-CGG-ATGTAG-CCAA-GTGGATCAAGG  50
```

Score = 110 bits (59), Expect = 3e-19
Identities = 59/59 (100%), Gaps = 0/59 (0%)
Strand=Plus/Plus

```
Query  111  AAAAAAGGAGCAATAACGCCCTCTTGATAAAACAAGAGGGAAGCTATTGCTCCtttttt  169
            |||||||||||||||||||||||||||||||||||||||||||||||||||||||||||
Sbjct  393  AAAAAAGGAGCAATAACGCCCTCTTGATAAAACAAGAGGGAAGCTATTGCTCCTTTTTT  451
```

>KF241795.1 Ambrosia artemisiifolia isolate NJF-12 PsbA (psbA) gene, partial cds; and psbA-trnH intergenic spacer, partial sequence; chloroplast
Length=445

Score = 582 bits (315), Expect = 2e-161
Identities = 426/473 (90%), Gaps = 33/473 (7%)
Strand=Plus/Plus

```
Query  8    CATGAACGTAATGCTCATAATTTCCCTCTAGACTTAGCTGCTATTGAAGCTCCATCTACA  67
            ||||||||||||||||||||||||||||||||||||||||||||||||||||||||||||
Sbjct  1    CATGAACGTAATGCTCATAATTTCCCTCTAGACTTAGCTGCTATTGAAGCTCCATCTACA  60

Query  68   AATGGATAAGCCTTTGGTCTGATTGTATAGGAGTTTTTGAACTAAAAAAGGAGCAATAAC  127
            |||||||||| |||||||||||||||||||||||||||||||||||||||||||||||||
Sbjct  61   AATGGATAAGACTTTGGTCTGATTGTATAGGAGTTTTTGAACTAAAAAAGGAGCAATAAC  120

Query  128  GCCCTCTTGATAAAACAAGAGGGAAGCTATTGCTCCtttttttATTTAGTACTATTTGCC  187
            | ||||||||||||||||||||||||||||||||||||||||||||||||||||||||||
Sbjct  121  GTCCTCTTGATAAAACAAGAGGGAAGCTATTGCTCCTTTTTTTATTTAGTACTATTTGCC  180

Query  188  TTACATAGTTTCTTTAAAAATAACAAGTACTTTTTATAGTTTGGTTCGatttgcgtgttt  247
            ||||||||||||||||||||||         |||||||||||||||||||||||||||||
Sbjct  181  TTACATAGTTTCTTTAAAAATA---------TTTTATAGTTTGGTTCGATTTGCGTGTTT  231

Query  248  tttctttgtattcatttatattataggtttctatatcatttattttcccaatcttttat  307
            ||||||||||||||||||||||||||||||||||||||| || |||||||||||||||||
Sbjct  232  TTTCTTTGTATTCATTTATATTATAGGTTTCTATATCATATACTTTTCCCAATCTTTTAT  291

Query  308  gaagttttatttaCAATTTAAATTTCAATCGaaaaaaagagaaaaaatgacaaattttgc  367
            ||||||||||||||||||  ||||||||||||||| | || ||||| |||||| |||| |
Sbjct  292  GAAGTTTTATTTACAATTC-AATTTCAATCGAAAATA-GACAAAAA-TGACAATTTTT-C  347

Query  368  tt-ttt-tga-ttttatattgattaaaatcaaaa-a-taaagaaaagaattttgcttttt  422
            || ||| | | |||||| || |||||| | |||| | ||||||||  ||| | |||||||
```

```
Sbjct  348  TTATTTATTACTTTTAT-TT-ATTAAA-TAAAAAGAATAAAGAAAT-AATAT-GCTTTTT  402

Query  423  tttATTGTTAAATGGaaaaaaaTATAGTTAATACTAGATAATCCGTAGAAGGG  475
               ||| | || ||||||||  ||||| ||||||||||||| | ||||| |||
Sbjct  403  G--ATT-T-AA-TGGAAAAA--TATAG-TAATACTAGATAA-CAGTAGA-GGG  445


>NC_053296.1 Aldama canescens voucher Schilling 17 chloroplast, complete genome
 MN337908.1 Aldama canescens chloroplast, complete genome
Length=151386

 Score = 580 bits (314),  Expect = 6e-161
 Identities = 460/522 (88%), Gaps = 43/522 (8%)
 Strand=Plus/Minus

Query  2    GTTATGCATGAACGTAATGCTCATAATTTCCCTCTAGACTTAGCTGCTATTGAAGCTCCA  61
            ||||||||||||||||||||||||||||||||||||||||||||||||||||||||||||
Sbjct  546  GTTATGCATGAACGTAATGCTCATAATTTCCCTCTAGACTTAGCTGCTATTGAAGCTCCA  487

Query  62   TCTACAAATGGATAAGCCTTTGGTCTGATTGTATAGGAGTTTTTGAACTAAAAAAGGAGC  121
            |||||||||||||||| |||||||||||||||||||||||||||||||||||||||||||
Sbjct  486  TCTACAAATGGATAAGACTTTGGTCTGATTGTATAGGAGTTTTTGAACTAAAAAAGGAGC  427

Query  122  AATAACGCCCTCTTGATAAAACAAGAGGGAAGCTATTGCTCCttttttt-ATTTAGTACT  180
            ||||||||||||||| |  | |||||||||||||||||||||||||||| ||||||||||
Sbjct  426  AATAACGCCCTCTTGTTTTATCAAGAGGGAAGCTATTGCTCCTTTTTTTTATTTAGTACT  367

Query  181  ATTTGCCTTACATAGTTTCTTTAAA-------AATAACAAGTACTTTTTATAGTTTGGTT  233
            |||||||||||||||||||||||||       ||||||||||||||||||||||||||||
Sbjct  366  ATTTGCCTTACATAGTTTCTTTAAACTTTAAAAATAACAAGTACTTTTTATAGTTTGGTT  307

Query  234  CGatttgcgtgttttttctttgtattcatttatattataggtttctatatcatttatttt  293
            |||||||  |||||||| ||||||||||||||||||||||||||||||||||| || |||
Sbjct  306  CGATTTGTTTGTTTTTTATTTGTATTCATTTATATTATAGGTTTCTATATCATATACTTT  247

Query  294  t-c----cc-aatcttttatgaagttttattt--aCAATTTAAATTTCAATCGaaaaaaa  345
            | |    || |||||||||||||||||| |||  ||||| |||| |||||||||||| |
Sbjct  246  TACTTTTCCTAATCTTTTATGAAGTTTTTTTTTTACAATCTAAA-TTCAATCGAAAATA-  189

Query  346  gagaaaaaatgacaaattttgctt-ttt-tga-ttttatattgattaaaatcaaaaataa  402
            || ||||| |||||| |||||||| ||| | | |||||| || ||| || | |||||| |
Sbjct  188  GATAAAAA-TGACAA-TTTTGCTTATTTATTACTTTTAT-TT-ATTCAA-T-AAAAATGA  135

Query  403  agaaaagaattttgcttttttttATTGTTAAATGGaaaaaaaTATAGTTAATACTAGATA  462
             |||| |||| | ||||||||  || ||||| ||||||||  ||||| ||||||||||||
Sbjct  134  -GAAA-GAATAT-GCTTTTTT--AT-GTTAA-TGGAAAAA--TATAG-TAATACTAGATA  85

Query  463  ATCCGTAGAAGGGGGCGGGATGTAGGCCAAAGTGGATCAAGG  504
            ||  ||||| |||| ||| |||||| |||| |||||||||||
Sbjct  84   ATA-GTAGA-GGGG-CGG-ATGTAG-CCAA-GTGGATCAAGG  49


>HQ596826.1 Rudbeckia triloba voucher AP397 trnH-psbA intergenic spacer,
partial sequence; chloroplast
Length=480

 Score = 579 bits (313),  Expect = 2e-160
```

Identities = 443/498 (89%), Gaps = 39/498 (8%)
Strand=Plus/Minus

```
Query  24   ATAATTTCCCTCTAGACTTAGCTGCTATTGAAGCTCCATCTACAAATGGATAAGCCTTTG  83
            |||||||||||||||||||||||||||||||||||||||||||||||||||||| |||||
Sbjct  480  ATAATTTCCCTCTAGACTTAGCTGCTATTGAAGCTCCATCTACAAATGGATAAGACTTTG  421

Query  84   GTCTGATTGTATAGGAGTTTTTGAACTAAAAAAGGAGCAATAACGCCCTCTTGATAAAA-  142
            |||||||||||||| ||||||||||||||||||||||||||| |||||||||||||||| 
Sbjct  420  GTCTGATTGTATAGAAGTTTTTGAACTAAAAAAGGAGCAATAGCGCCCTCTTGATAAAAA  361

Query  143  -----CAAGAGGGAAGCTATTGCTCCtttttttATTTAGTACTATTTGCCTTACATAGTT  197
                 |||||||||||||||||||||||||||||||||||||||||||| ||||||||||
Sbjct  360  TAAAACAAGAGGGAAGCTATTGCTCCTTTTTTTATTTAGTACTATTTGCTTTACATAGTT  301

Query  198  TCTTTAAAAATAACAAGTACTTTTTATAGTTTGGTTCGatttgcgtgtttttctttgta  257
            ||||||||||| ||||||||||||||||||||||||||||||||||||||||||||||||
Sbjct  300  TCTTTAAAAATGACAAGTACTTTTTATAGTTTGGTTCGATTTGCGTGTTTTTCTTTGTA  241

Query  258  ttcatttat-----attataggtttctatatcatttattttcccaatcttttatgaagt  312
            |||||||||     ||||||||||||||||||| || |||||||||||||||||||||
Sbjct  240  TTCATTTATTTTATATTATAGGTTTCTATATCATATACTTTTCCCAATCTTTTATGAAGT  181

Query  313  tttatttaCAATTTAAATTTCAATCGaaaaaaagagaaaaaatgacaaattttgctt-tt  371
            ||||||||||||| || ||||||||||||| | || ||||| ||| |||||||| || ||
Sbjct  180  TTTATTTACAATTCAA-TTTCAATCGAAAATA-GATAAAAA-TGA-AAATTTTGATTATT  125

Query  372  t-tga-ttttatattgattaaaatcaaaaataa-aga-aaagaattttgctttttttAT  427
            | | | |||||| || |||||| | |||||||| | | ||| ||| | ||||||||||
Sbjct  124  TATTACTTTTAT-TT-ATTAAA-T-AAAAATAAGAAAGAAATAATAT-GCTTTTTTTT--  72

Query  428  TGTTAAATGGaaaaaaaTATAGTTAATACTAGATAATCCGTAGAAGGGGGCGGGATGTAG  487
             ||||| ||||||||  ||||| ||||||||||||||  ||||| |||| ||| ||||||
Sbjct  71   -GTTAA-TGGAAAAA--TATAG-TAATACTAGATAATA-GTAGA-GGGG-CGG-ATGTAG  21

Query  488  GCCAAAGTGGATC-AAGG  504
             |||| ||||||| ||||
Sbjct  20   -CCAA-GTGGATCNAAGG  5
```

>GQ248245.1 Ambrosia trifida voucher NMNH Wells 30 psbA-trnH intergenic spacer, partial sequence; chloroplast
Length=459

Score = 577 bits (312), Expect = 7e-160
Identities = 435/487 (89%), Gaps = 38/487 (8%)
Strand=Plus/Plus

```
Query  24   ATAATTTCCCTCTAGACTTAGCTGCTATTGAAGCTCCATCTACAAATGGATAAGCCTTTG  83
            |||||||||||||||||||||||||||||||||||||||||||||||||||||| |||||
Sbjct  1    ATAATTTCCCTCTAGACTTAGCTGCTATTGAAGCTCCATCTACAAATGGATAAGACTTTG  60

Query  84   GTCTGATTGTATAGGAGTTTTTGAACTAAAAAAGGAGCAATAACGCCCTCTTGATAAAAC  143
            |||||||||||||||||||||||||||||||||||||||||||||| ||||||||||||||
Sbjct  61   GTCTGATTGTATAGGAGTTTTTGAACTAAAAAAGGAGCAATAACGTCCTCTTGATAAAAC  120

Query  144  AAGAGGGAAGCTATTGCTCC-tttttttATTTAGTACTATTTGCCTTACATAGTTTCTTT  202
```

```
                ||||||||||||||||||||| ||||||||||||||||||||||||||||||||||||||||
Sbjct  121  AAGAGGGAAGCTATTGCTCCTTTTTTTTATTTAGTACTATTTGCCTTACATAGTTTCTTT  180

Query  203  AAAAATAACAAGTACTTTTTATAGTTTGGTTCGatttgcgtgtttttctttgtattcat  262
            |||||||         ||||||||||||||||||||||||||||||||||||||||||||
Sbjct  181  AAAAATA---------TTTTATAGTTTGGTTCGATTTGCGTGTTTTTCTTTGTATTCAT  231

Query  263  ttatattataggtttctatatcatttattttcccaatcttttatgaagttttatttaCA  322
            |||||||||||||||||||||||| || ||||||||||||||||||||||||||||||||
Sbjct  232  TTATATTATAGGTTTCTATATCATATACTTTTCCCAATCTTTTATGAAGTTTTATTTACA  291

Query  323  ATTTAAATTTCAATCGaaaaaaagagaaaaaatgacaaattttgctt-ttt-tga-tttt  379
            |||  ||||||||||||||| | || ||||| |||||| |||| ||| ||| | | ||||
Sbjct  292  ATTC-AATTTCAATCGAAAATA-GACAAAAA-TGACAATTTTT-CTTATTTATTACTTTT  347

Query  380  atattgattaaaatcaaaa-a-taaagaaaagaattttgcttttttttATTGTTAAATGG  437
            || || |||||| | |||| | ||||||||  ||| | |||||||   ||| | || |||
Sbjct  348  AT-TT-ATTAAA-TAAAAAGAATAAAGAAAT-AATAT-GCTTTTTG--ATT-T-AA-TGG  397

Query  438  aaaaaaaTATAGTTAATACTAGATAATCCGTAGAAGGGGGCGGATGTAGGCCAAAGTGG  497
            |||||  ||||| ||||||||||||| | ||||| |||| ||| |||||| |||| ||||
Sbjct  398  AAAAA--TATAG-TAATACTAGATAA-CAGTAGA-GGGG-CGG-ATGTAG-CCAA-GTGG  448

Query  498  ATCAAGG  504
            |||||||
Sbjct  449  ATCAAGG  455


>DQ006141.1 Ambrosia artemisiifolia voucher Wells 4495 US PsbA (psbA) gene,
partial cds; psbA-trnH intergenic spacer, complete sequence;
and tRNA-His (trnH) gene, partial sequence; chloroplast
 DQ006142.1 Ambrosia trifida voucher Wiser 30 US PsbA (psbA) gene, partial
cds; psbA-trnH intergenic spacer, complete sequence; and tRNA-His
(trnH) gene, partial sequence; chloroplast
Length=456

 Score = 577 bits (312),  Expect = 7e-160
 Identities = 435/487 (89%), Gaps = 38/487 (8%)
 Strand=Plus/Plus

Query  24   ATAATTTCCCTCTAGACTTAGCTGCTATTGAAGCTCCATCTACAAATGGATAAGCCTTTG  83
            |||||||||||||||||||||||||||||||||||||||||||||||||||||| |||||
Sbjct  1    ATAATTTCCCTCTAGACTTAGCTGCTATTGAAGCTCCATCTACAAATGGATAAGACTTTG  60

Query  84   GTCTGATTGTATAGGAGTTTTTGAACTAAAAAAGGAGCAATAACGCCCTCTTGATAAAAC  143
            ||||||||||||||||||||||||||||||||||||||||||||| ||||||||||||||
Sbjct  61   GTCTGATTGTATAGGAGTTTTTGAACTAAAAAAGGAGCAATAACGTCCTCTTGATAAAAC  120

Query  144  AAGAGGGAAGCTATTGCTCC-tttttttATTTAGTACTATTTGCCTTACATAGTTTCTTT  202
            |||||||||||||||||||| |||||||||||||||||||||||||||||||||||||||
Sbjct  121  AAGAGGGAAGCTATTGCTCCTTTTTTTTATTTAGTACTATTTGCCTTACATAGTTTCTTT  180

Query  203  AAAAATAACAAGTACTTTTTATAGTTTGGTTCGatttgcgtgtttttctttgtattcat  262
            |||||||         ||||||||||||||||||||||||||||||||||||||||||||
Sbjct  181  AAAAATA---------TTTTATAGTTTGGTTCGATTTGCGTGTTTTTCTTTGTATTCAT  231

Query  263  ttatattataggtttctatatcatttattttcccaatcttttatgaagttttatttaCA  322
```

```
                ||||||||||||||||||||||||| || ||||||||||||||||||||||||||||||||
Sbjct  232   TTATATTATAGGTTTCTATATCATATACTTTTCCCAATCTTTTATGAAGTTTTATTTACA  291

Query  323   ATTTAAATTTCAATCGaaaaaaagagaaaaaaatgacaaattttgctt-ttt-tga-tttt  379
             |||  ||||||||||||||| | || ||||| |||||| |||| ||| ||| | | ||||
Sbjct  292   ATTC-AATTTCAATCGAAAATA-GACAAAAA-TGACAATTTTT-CTTATTTATTACTTTT  347

Query  380   atattgattaaaatcaaaa-a-taaagaaaagaattttgcttttttttATTGTTAAATGG  437
             || || |||||| | |||| | ||||||||  ||| | |||||||   ||| | || |||
Sbjct  348   AT-TT-ATTAAA-TAAAAGAATAAAGAAAT-AATAT-GCTTTTTG--ATT-T-AA-TGG  397

Query  438   aaaaaaaTATAGTTAATACTAGATAATCCGTAGAAGGGGGCGGGATGTAGGCCAAAGTGG  497
             |||||  ||||| ||||||||||||| | ||||| |||| ||| |||||| |||| ||||
Sbjct  398   AAAAA--TATAG-TAATACTAGATAA-CAGTAGA-GGGG-CGG-ATGTAG-CCAA-GTGG  448

Query  498   ATCAAGG  504
             |||||||
Sbjct  449   ATCAAGG  455


>AY215541.1 Enydra sessilis voucher Panero 3126 (TEX) psbA gene and psbA-trnH
intergenic spacer, partial sequence; chloroplast
Length=428

 Score = 575 bits (311),  Expect = 3e-159
 Identities = 405/446 (91%), Gaps = 23/446 (5%)
 Strand=Plus/Minus

Query  29    TTCCCTCTAGACTTAGCTGCTATTGAAGCTCCATCTACAAATGGATAAGCCTTTGGTCTG  88
             ||||||||||||||||||||||||||||||||||||||||||||||||| ||||||||||
Sbjct  428   TTCCCTCTAGACTTAGCTGCTATTGAAGCTCCATCTACAAATGGATAAGACTTTGGTCTG  369

Query  89    ATTGTATAGGAGTTTTTGAACTAAAAAAGGAGCAATAACGCCCTCTTGATAAAACAAGAG  148
             ||||||||||||||||||||||||||||||||||||||||||||||||||||||||||||
Sbjct  368   ATTGTATAGGAGTTTTTGAACTAAAAAAGGAGCAATAACGCCCTCTTGATAAAACAAGAG  309

Query  149   GGAAGCTATTGCTCCttttttt-ATTTAGTACTATTTGCCTTACATAGTTTCTTTAAAAA  207
             |||||||||||||||||||||| |||||||||||||||||||||||||||||||||||||
Sbjct  308   GGAAGCTATTGCTCCTTTTTTTTATTTAGTACTATTTGCCTTACATAGTTTCTTTAAAAA  249

Query  208   TAACAAGTACTTTTTATAGTTTGGTTCGatttgcgtgttttttctttgtattcatttata  267
             ||||||| ||||||| ||||||||||||||||||||||||| ||||||||||||||||||
Sbjct  248   TAACAAGGACTTTTTCTAGTTTGGTTCGATTTGCGTGTTTTATCTTTGTATTCATTTATA  189

Query  268   ttataggtttctatatcatttattttccc-aatcttt-tatgaagttttatttaCAATT  325
             |||||||||||||||||||||| ||||||| ||| ||| ||||||||||||||| |||||
Sbjct  188   TTATAGGTTTCTATATCATTTACTTTTCCCCAATATTTGTATGAAGTTTTATTTCCAATT  129

Query  326   TAAATTTCAATCGaaaaaaagagaaaaaaatgacaaattttgctttttttgattttatattg  385
              || ||||||||||||| | || ||||| ||| ||||||||||| ||| |||   | |||
Sbjct  128   CAA-TTTCAATCGAAAATA-GATAAAAA-TGA-AAATTTTGCTTATTT-ATTAC-T-TTG  76

Query  386   attaaaatcaaaaataaagaaaagaattttgcttttttttATTGTTAAATGGaaaaaaT  445
             ||| |  | ||||| |  |||| ||||  ||||||||||||| ||||| ||||||||  |
Sbjct  75    ATTTAT-T-AAAAA-A--GAAA-GAATA-TGCTTTTTTTTAT-GTTAA-TGGAAAAA--T  27

Query  446   ATAGTTAATACTAGATAATCCGTAGA  471
```

```
             |||| |||||||||| |||  |||||
Sbjct  26    ATAG-TAATACTAGAGAATA-GTAGA  3


>NC_057174.1 Aldama robusta voucher Bringel 985 chloroplast, complete genome
 MN356028.1 Aldama robusta chloroplast, complete genome
Length=151343

 Score = 575 bits (311),  Expect = 3e-159
 Identities = 458/521 (88%), Gaps = 42/521 (8%)
 Strand=Plus/Minus

Query  2    GTTATGCATGAACGTAATGCTCATAATTTCCCTCTAGACTTAGCTGCTATTGAAGCTCCA  61
            ||||||||||||||||||||||||||||||||||||||||||||||||||||||||||||
Sbjct  554  GTTATGCATGAACGTAATGCTCATAATTTCCCTCTAGACTTAGCTGCTATTGAAGCTCCA  495

Query  62   TCTACAAATGGATAAGCCTTTGGTCTGATTGTATAGGAGTTTTTGAACTAAAAAAGGAGC  121
            |||||||||||||||| |||||||||||||||||||||||||||||||||||||||||||
Sbjct  494  TCTACAAATGGATAAGACTTTGGTCTGATTGTATAGGAGTTTTTGAACTAAAAAAGGAGC  435

Query  122  AATAACGCCCTCTTGATAAAACAAGAGGGAAGCTATTGCTCCttttttt-ATTTAGTACT  180
            ||||||||||||||| |  | |||||||||||||||||||||||||||| ||||||||||
Sbjct  434  AATAACGCCCTCTTGTTTTATCAAGAGGGAAGCTATTGCTCCTTTTTTTTATTTAGTACT  375

Query  181  ATTTGCCTTACATAGTTTCTTTAAA-------AATAACAAGTACTTTTTATAGTTTGGTT  233
            |||||||||||||||||||||||||       ||||||||||||||||||||||||||||
Sbjct  374  ATTTGCCTTACATAGTTTCTTTAAACTTTAAAAATAACAAGTACTTTTTATAGTTTGGTT  315

Query  234  CGatttgcgtgttttttctttgtattcatttatattataggtttctatatcatttatttt  293
            |||||||  |||||||| ||||||||||||||||||||||||||||||||||| || |||
Sbjct  314  CGATTTGTTTGTTTTTTATTTGTATTCATTTATATTATAGGTTTCTATATCATATACTTT  255

Query  294  t-c----cc-aatcttttatgaagttttattt-aCAATTTAAATTTCAATCGaaaaaaag  346
            | |    || |||||||||||||||||| ||| ||||| |||| |||||||||||| | |
Sbjct  254  TACTTTTCCTAATCTTTTATGAAGTTTTTTTTTACAATCTAAA-TTCAATCGAAAATA-G  197

Query  347  agaaaaaatgacaaattttgctt-ttt-tga-ttttatattgattaaaatcaaaaataaa  403
            | ||||| |||||| ||||| || ||| | | |||||| || ||| || | |||||| |
Sbjct  196  ATAAAAA-TGACAA-TTTTGTTTATTTATTACTTTTAT-TT-ATTCAA-T-AAAAATGA-  144

Query  404  gaaaagaattttgcttttttttATTGTTAAATGGaaaaaaaTATAGTTAATACTAGATAA  463
            |||| |||| | ||||||||  || ||||| ||| ||||  ||||| |||||||||||||
Sbjct  143  GAAA-GAATAT-GCTTTTTT--AT-GTTAA-TGGGAAAA--TATAG-TAATACTAGATAA  93

Query  464  TCCGTAGAAGGGGGCGGATGTAGGCCAAAGTGGATCAAGG  504
            |  ||||| |||| ||| |||||| |||| |||||||||||
Sbjct  92   TA-GTAGA-GGGG-CGG-ATGTAG-CCAA-GTGGATCAAGG  58


>NC_053279.1 Aldama filifolia voucher Loeuille 849 chloroplast, complete genome
 MN337890.1 Aldama filifolia chloroplast, complete genome
Length=151333

 Score = 575 bits (311),  Expect = 3e-159
 Identities = 458/521 (88%), Gaps = 42/521 (8%)
 Strand=Plus/Minus
```

```
Query  2    GTTATGCATGAACGTAATGCTCATAATTTCCCTCTAGACTTAGCTGCTATTGAAGCTCCA  61
            ||||||||||||||||||||||||||||||||||||||||||||||||||||||||||||
Sbjct  554  GTTATGCATGAACGTAATGCTCATAATTTCCCTCTAGACTTAGCTGCTATTGAAGCTCCA  495

Query  62   TCTACAAATGGATAAGCCTTTGGTCTGATTGTATAGGAGTTTTTGAACTAAAAAAGGAGC  121
            ||||||||||||||| ||||||||||||||||||||||||||||||||||||||||||||
Sbjct  494  TCTACAAATGGATAAGACTTTGGTCTGATTGTATAGGAGTTTTTGAACTAAAAAAGGAGC  435

Query  122  AATAACGCCCTCTTGATAAAACAAGAGGGAAGCTATTGCTCCttttttt-ATTTAGTACT  180
            ||||||||||||||| |  | |||||||||||||||||||||||||||| ||||||||||
Sbjct  434  AATAACGCCCTCTTGTTTTATCAAGAGGGAAGCTATTGCTCCTTTTTTTTATTTAGTACT  375

Query  181  ATTTGCCTTACATAGTTTCTTTAAA-------AATAACAAGTACTTTTTATAGTTTGGTT  233
            |||||||||||||||||||||||||       ||||||||||||||||||||||||||||
Sbjct  374  ATTTGCCTTACATAGTTTCTTTAAACTTTAAAAATAACAAGTACTTTTTATAGTTTGGTT  315

Query  234  CGatttgcgtgttttttctttgtattcatttatattataggtttctatatcatttatttt  293
            |||||||  |||||||| ||||||||||||||||||||||||||||||||||| || |||
Sbjct  314  CGATTTGTTTGTTTTTTATTTGTATTCATTTATATTATAGGTTTCTATATCATATACTTT  255

Query  294  t-c----cc-aatcttttatgaagttttattt-aCAATTTAAATTTCAATCGaaaaaaag  346
            | |    || |||||||||||||||||| ||| ||||| |||| |||||||||||| | |
Sbjct  254  TACTTTTCCTAATCTTTTATGAAGTTTTTTTTTACAATCTAAA-TTCAATCGAAAATA-G  197

Query  347  agaaaaaatgacaaattttgctt-ttt-tga-ttttatattgattaaaatcaaaaataaa  403
            | ||||| |||||| ||||| || ||| | | |||||| || ||| || | |||||| |
Sbjct  196  ATAAAAA-TGACAA-TTTTGTTTATTTATTACTTTTAT-TT-ATTCAA-T-AAAAATGA-  144

Query  404  gaaaagaattttgcttttttttATTGTTAAATGGaaaaaaaTATAGTTAATACTAGATAA  463
            |||| |||| | ||||||||  || ||||| ||| ||||  ||||| |||||||||||||
Sbjct  143  GAAA-GAATAT-GCTTTTTT--AT-GTTAA-TGGGAAAA--TATAG-TAATACTAGATAA  93

Query  464  TCCGTAGAAGGGGGCGGGATGTAGGCCAAAGTGGATCAAGG  504
            |  ||||| |||| ||| |||||| |||| |||||||||||
Sbjct  92   TA-GTAGA-GGGG-CGG-ATGTAG-CCAA-GTGGATCAAGG  58


>NC_057176.1 Aldama santacatarinensis voucher Magenta 706 chloroplast, complete
genome
 MN356030.1 Aldama santacatarinensis chloroplast, complete genome
Length=151362

 Score = 575 bits (311),  Expect = 3e-159
 Identities = 458/521 (88%), Gaps = 42/521 (8%)
 Strand=Plus/Minus

Query  2    GTTATGCATGAACGTAATGCTCATAATTTCCCTCTAGACTTAGCTGCTATTGAAGCTCCA  61
            ||||||||||||||||||||||||||||||||||||||||||||||||||||||||||||
Sbjct  554  GTTATGCATGAACGTAATGCTCATAATTTCCCTCTAGACTTAGCTGCTATTGAAGCTCCA  495

Query  62   TCTACAAATGGATAAGCCTTTGGTCTGATTGTATAGGAGTTTTTGAACTAAAAAAGGAGC  121
            ||||||||||||||| ||||||||||||||||||||||||||||||||||||||||||||
Sbjct  494  TCTACAAATGGATAAGACTTTGGTCTGATTGTATAGGAGTTTTTGAACTAAAAAAGGAGC  435

Query  122  AATAACGCCCTCTTGATAAAACAAGAGGGAAGCTATTGCTCCtttttttATTTAGTACTA  181
            ||||||||||||||| |  | |||||||||||||||||||||||||||||||||||||||
Sbjct  434  AATAACGCCCTCTTGTTTTATCAAGAGGGAAGCTATTGCTCCTTTTTTTATTTAGTACTA  375
```

```
Query  182   TTTGCCTTACATAGTTTCTTTAAA-------AATAACAAGTACTTTTTATAGTTTGGTTC  234
             ||||||||||||||||||||||||       |||||||||||||||||||||||||||||
Sbjct  374   TTTGCCTTACATAGTTTCTTTAAACTTTAAAAATAACAAGTACTTTTTATAGTTTGGTTC  315

Query  235   Gatttgcgtgtttttctttgtattcatttatattataggtttctatatcatttattttt  294
             ||||||  |||||||| ||||||||||||||||||||||||||||||||||| || ||||
Sbjct  314   GATTTGTTTGTTTTTTATTTGTATTCATTTATATTATAGGTTTCTATATCATATACTTTT  255

Query  295   -c----cc-aatcttttatgaagttttattt--aCAATTTAAATTTCAATCGaaaaaaag  346
              |    || ||||||||||||||||||| |||  ||||| |||| |||||||||||| | |
Sbjct  254   ACTTTTCCTAATCTTTTATGAAGTTTTTTTTTTACAATCTAAA-TTCAATCGAAAATA-G  197

Query  347   agaaaaaatgacaaattttgctt-ttt-tga-ttttatattgattaaaatcaaaaataaa  403
             | ||||| |||||| ||||| || ||| | | |||||| || ||| || | |||||| |
Sbjct  196   ATAAAAA-TGACAA-TTTTGTTTATTTATTACTTTTAT-TT-ATTCAA-T-AAAAATGA-  144

Query  404   gaaaagaattttgctttttttATTGTTAAATGGaaaaaaaTATAGTTAATACTAGATAA  463
             |||| |||| | ||||||||  || ||||| ||| ||||  ||||| |||||||||||||
Sbjct  143   GAAA-GAATAT-GCTTTTTT--AT-GTTAA-TGGGAAAA--TATAG-TAATACTAGATAA  93

Query  464   TCCGTAGAAGGGGGCGGGATGTAGGCCAAAGTGGATCAAGG  504
             |  ||||| |||| ||| |||||| |||| |||||||||||
Sbjct  92    TA-GTAGA-GGGG-CGG-ATGTAG-CCAA-GTGGATCAAGG  58


>NC_053290.1 Aldama pilosa voucher Filartiga 10 chloroplast, complete genome
 MN337901.1 Aldama pilosa chloroplast, complete genome
Length=151354

 Score = 575 bits (311),  Expect = 3e-159
 Identities = 458/521 (88%), Gaps = 42/521 (8%)
 Strand=Plus/Minus

Query  2     GTTATGCATGAACGTAATGCTCATAATTTCCCTCTAGACTTAGCTGCTATTGAAGCTCCA  61
             ||||||||||||||||||||||||||||||||||||||||||||||||||||||||||||
Sbjct  556   GTTATGCATGAACGTAATGCTCATAATTTCCCTCTAGACTTAGCTGCTATTGAAGCTCCA  497

Query  62    TCTACAAATGGATAAGCCTTTGGTCTGATTGTATAGGAGTTTTTGAACTAAAAAAGGAGC  121
             |||||||||||||||| |||||||||||||||||||||||||||||||||||||||||||
Sbjct  496   TCTACAAATGGATAAGACTTTGGTCTGATTGTATAGGAGTTTTTGAACTAAAAAAGGAGC  437

Query  122   AATAACGCCCTCTTGATAAAACAAGAGGGAAGCTATTGCTCCtttttttATTTAGTACTA  181
             ||||||||||||||| |  | ||||||||||||||||||||||||||||||||||||||||
Sbjct  436   AATAACGCCCTCTTGTTTTATCAAGAGGGAAGCTATTGCTCCTTTTTTTATTTAGTACTA  377

Query  182   TTTGCCTTACATAGTTTCTTTAAA-------AATAACAAGTACTTTTTATAGTTTGGTTC  234
             ||||||||||||||||||||||||       |||||||||||||||||||||||||||||
Sbjct  376   TTTGCCTTACATAGTTTCTTTAAACTTTAAAAATAACAAGTACTTTTTATAGTTTGGTTC  317

Query  235   Gatttgcgtgtttttctttgtattcatttatattataggtttctatatcatttattttt  294
             ||||||  |||||||| ||||||||||||||||||||||||||||||||||| || ||||
Sbjct  316   GATTTGTTTGTTTTTTATTTGTATTCATTTATATTATAGGTTTCTATATCATATACTTTT  257

Query  295   -c----cc-aatcttttatgaagttttattt--aCAATTTAAATTTCAATCGaaaaaaag  346
              |    || ||||||||||||||||||| |||  ||||| |||| |||||||||||| | |
Sbjct  256   ACTTTTCCTAATCTTTTATGAAGTTTTTTTTTTACAATCTAAA-TTCAATCGAAAATA-G  199
```

```
Query  347  agaaaaaatgacaaattttgctt-ttt-tga-ttttatattgattaaaatcaaaaataaa  403
            | ||||| |||||| ||||| || ||| | | |||||| || ||| || | |||||| |
Sbjct  198  ATAAAAA-TGACAA-TTTTGTTTATTTATTACTTTTAT-TT-ATTCAA-T-AAAAATGA-  146

Query  404  gaaaagaattttgcttttttttATTGTTAAATGGaaaaaaaTATAGTTAATACTAGATAA  463
            |||| |||| | ||||||||  || ||||| ||| ||||  ||||| |||||||||||||
Sbjct  145  GAAA-GAATAT-GCTTTTTT--AT-GTTAA-TGGGAAAA--TATAG-TAATACTAGATAA  95

Query  464  TCCGTAGAAGGGGGCGGGATGTAGGCCAAAGTGGATCAAGG  504
            |  ||||| |||| ||| |||||| |||| |||||||||||
Sbjct  94   TA-GTAGA-GGGG-CGG-ATGTAG-CCAA-GTGGATCAAGG  60


>MT793848.1 Litothamnus nitidus chloroplast, complete genome
Length=151517

 Score = 575 bits (311),  Expect = 3e-159
 Identities = 447/508 (88%), Gaps = 27/508 (5%)
 Strand=Plus/Minus

Query  2    GTTATGCATGAACGTAATGCTCATAATTTCCCTCTAGACTTAGCTGCTATTGAAGCTCCA  61
            ||||||||||||||||||||||||||||||||||||||||||||||||||||||||||||
Sbjct  534  GTTATGCATGAACGTAATGCTCATAATTTCCCTCTAGACTTAGCTGCTATTGAAGCTCCA  475

Query  62   TCTACAAATGGATAAGCCTTTGGTCTGATTGTATAGGAGTTTTTGAACTAAAAAAGGAGC  121
            |||||||||||||||| |||||||||||||||||||||||||||||||||||||||||||
Sbjct  474  TCTACAAATGGATAAGACTTTGGTCTGATTGTATAGGAGTTTTTGAACTAAAAAAGGAGC  415

Query  122  AATAACG-CCCTCTTGATAAAACAAGAGGGAAGCTATTGCTCCtttttttATTTAGTACT  180
            |||| |  |||||||| |  | ||| ||||  | ||||||||||||||||||||||||||
Sbjct  414  AATAGCTTCCCTCTTGTTTTATCAATAGGGC-GTTATTGCTCCTTTTTTTATTTAGTACT  356

Query  181  ATTTGCCTTACATAGTTTCTTTAAAAATAACAAGTACTTTTTATAGTTTGGTTCGatttg  240
            |||||||||| ||||||||||||||||||||||| ||||||||||||||||||||| |||
Sbjct  355  ATTTGCCTTATATAGTTTCTTTAAAAATAACAAGGACTTTTTATAGTTTGGTTCGAGTTG  296

Query  241  cgtgttttttctttgtattcatttatattataggtttctatatcatttatttttcccaat  300
            ||||||||||||||||||||||||||||||||||||||||||| || || ||||||||||
Sbjct  295  CGTGTTTTTTCTTTGTATTCATTTATATTATAGGTTTCTATATAATATACTTTTCCCAAT  236

Query  301  cttttatgaagttttatttaCAATTTAAATTTCAATCGaaaaaaagagaaaaaatgacaa  360
            |||||||||||||||||||| ||||| || |||||||| |||| | || ||||| | | ||
Sbjct  235  CTTTTATGAAGTTTTATTTCCAATTCAA-TTTCAATCTAAAATA-GATAAAAATT-A-AA  180

Query  361  atttgctt-ttt-tga-ttttatattgat-taaaatcaaaaataaagaaaagaattttg  416
            ||||| ||| ||| | | |||| | || || |  | | || || |||||||  ||| | |
Sbjct  179  ATTTTTCTTATTTATTACTTTTCT-TT-ATCTTTATTAAATAAGAAAGAAAT-AATAT-G  124

Query  417  cttttttttATTGTTAAATGGaaaaaaaTATAGTTAATACTAGATAATCCGTAGAAGGGG  476
            |||||||  || ||||| ||||||||  ||||| ||||||||||||||  ||||| ||||
Sbjct  123  CTTTTTT--AT-GTTAA-TGGAAAAA--TATAG-TAATACTAGATAATA-GTAGA-GGGG  73

Query  477  GCGGGATGTAGGCCAAAGTGGATCAAGG  504
             ||| |||||| |||| |||||||||||
Sbjct  72   -CGG-ATGTAG-CCAA-GTGGATCAAGG  49
```

```
 Score = 104 bits (56),  Expect = 1e-17
 Identities = 58/59 (98%), Gaps = 0/59 (0%)
 Strand=Plus/Plus

Query  111  AAAAAAGGAGCAATAACGCCCTCTTGATAAAACAAGAGGGAAGCTATTGCTCCtttttt  169
            |||||||||||||||||||||| ||||||||||||||||||||||||||||||||||||
Sbjct  367  AAAAAAGGAGCAATAACGCCCTATTGATAAAACAAGAGGGAAGCTATTGCTCCTTTTTT  425


>NC_053297.1 Aldama corumbensis voucher Loeuille 909 chloroplast, complete
genome
 MN337909.1 Aldama corumbensis chloroplast, complete genome
Length=151327

 Score = 575 bits (311),  Expect = 3e-159
 Identities = 458/521 (88%), Gaps = 42/521 (8%)
 Strand=Plus/Minus

Query  2    GTTATGCATGAACGTAATGCTCATAATTTCCCTCTAGACTTAGCTGCTATTGAAGCTCCA  61
            ||||||||||||||||||||||||||||||||||||||||||||||||||||||||||||
Sbjct  545  GTTATGCATGAACGTAATGCTCATAATTTCCCTCTAGACTTAGCTGCTATTGAAGCTCCA  486

Query  62   TCTACAAATGGATAAGCCTTTGGTCTGATTGTATAGGAGTTTTTGAACTAAAAAAGGAGC  121
            |||||||||||||||| |||||||||||||||||||||||||||||||||||||||||||
Sbjct  485  TCTACAAATGGATAAGACTTTGGTCTGATTGTATAGGAGTTTTTGAACTAAAAAAGGAGC  426

Query  122  AATAACGCCCTCTTGATAAAACAAGAGGGAAGCTATTGCTCCttttttt-ATTTAGTACT  180
            ||||||||||||||| |  | |||||||||||||||||||||||||||| ||||||||||
Sbjct  425  AATAACGCCCTCTTGTTTTATCAAGAGGGAAGCTATTGCTCCTTTTTTTTATTTAGTACT  366

Query  181  ATTTGCCTTACATAGTTTCTTTAAA-------AATAACAAGTACTTTTTATAGTTTGGTT  233
            |||||||||||||||||||||||||       ||||||||||||||||||||||||||||
Sbjct  365  ATTTGCCTTACATAGTTTCTTTAAACTTTAAAAATAACAAGTACTTTTTATAGTTTGGTT  306

Query  234  CGatttgcgtgttttttctttgtattcatttatattataggtttctatatcatttatttt  293
            |||||||  |||||||| ||||||||||||||||||||||||||||||||||| || |||
Sbjct  305  CGATTTGTTTGTTTTTTATTTGTATTCATTTATATTATAGGTTTCTATATCATATACTTT  246

Query  294  t-c----cc-aatcttttatgaagttttattt-aCAATTTAAATTTCAATCGaaaaaaag  346
            | |    || |||||||||||||||||| ||| ||||| |||| |||||||||||| | |
Sbjct  245  TACTTTTCCTAATCTTTTATGAAGTTTTTTTTTACAATCTAAA-TTCAATCGAAAATA-G  188

Query  347  agaaaaaatgacaaattttgctt-ttt-tga-ttttatattgattaaaatcaaaaataaa  403
            | ||||| |||||| ||||| || ||| | | |||||| || ||| || | |||||| | 
Sbjct  187  ATAAAAA-TGACAA-TTTTGTTTATTTATTACTTTTAT-TT-ATTCAA-T-AAAAATGA-  135

Query  404  gaaaagaattttgcttttttttATTGTTAAATGGaaaaaaaTATAGTTAATACTAGATAA  463
            |||| |||| | ||||||||  || ||||| ||| ||||  ||||| |||||||||||||
Sbjct  134  GAAA-GAATAT-GCTTTTTT--AT-GTTAA-TGGGAAAA--TATAG-TAATACTAGATAA  84

Query  464  TCCGTAGAAGGGGGCGGGATGTAGGCCAAAGTGGATCAAGG  504
            |  ||||| |||| ||| |||||| |||| |||||||||||
Sbjct  83   TA-GTAGA-GGGG-CGG-ATGTAG-CCAA-GTGGATCAAGG  49


>OZ247556.1 Inula salicina genome assembly, organelle: plastid:chloroplast
```

```
Length=151103

 Score = 573 bits (310),  Expect = 9e-159
 Identities = 453/516 (88%), Gaps = 34/516 (7%)
 Strand=Plus/Minus

Query  2     GTTATGCATGAACGTAATGCTCATAATTTCCCTCTAGACTTAGCTGCTATTGAAGCTCCA  61
             ||||||||||||||||||||||| ||||||||||||||||||||||||||||||||||||
Sbjct  1852  GTTATGCATGAACGTAATGCTCACAATTTCCCTCTAGACTTAGCTGCTATTGAAGCTCCA  1793

Query  62    TCTACAAATGGATAAGCCTTTGGTCTGATTGTATAGGAGTTTTTGAACTAAAAAA-GGAG  120
             |||||||||||||||| |||||||||||||||||||||||||||||||||||||| || |
Sbjct  1792  TCTACAAATGGATAAGACTTTGGTCTGATTGTATAGGAGTTTTTGAACTAAAAAAAGGGG  1733

Query  121   CAATAACGCCCTCTTGATAAAACAAGAGGGAAGCTATTGCTCCtttttttATTTAGTACT  180
             ||||||||||||||||||||||||||||||  | ||||||||||||||||||||||||||
Sbjct  1732  CAATAACGCCCTCTTGATAAAACAAGAGGG-CGTTATTGCTCCTTTTTTTATTTAGTACT  1674

Query  181   ATTTGCCTTACATAGTTTCTTTAAAAATAACAAGTACTTTTTATAGTTTGGTTCGatttg  240
             ||||||||||||||||||||||||||||||||||  ||| |||||||||||||||||| |
Sbjct  1673  ATTTGCCTTACATAGTTTCTTTAAAAATAACAAGGGCTTCTTATAGTTTGGTTCGATTCG  1614

Query  241   cgtgttttttctttgtattcatttatattataggtttctatatcatttatttttcccaat  300
             |||||||| |||||||||||||||||||| |||||||||||||| |||     |||||||
Sbjct  1613  CGTGTTTTCTCTTTGTATTCATTTATATTGTAGGTTTCTATATCCTTT-----TCCCAAT  1559

Query  301   cttttatgaagttttatttaCAATTTAAATTTCAATCGaaaaaaagagaaaaaatgacaa  360
             ||||||||||||||||||| ||||| || ||||||||||||| | || ||||| ||| ||
Sbjct  1558  CTTTTATGAAGTTTTATTTCCAATTCAA-TTTCAATCGAAAATA-GATAAAAA-TGA-AA  1503

Query  361   attttgcttttttgattttatattgattaaaatcaaaaataaagaaa-agaatt-t-tgc  417
             ||||||||| ||| |||   | || |||  | | || |||||||||| || ||  | |||
Sbjct  1502  ATTTTGCTTATTT-ATTAC-T-TTCATTTCA-TAAATAATAAAGAAATAGTATGATATGC  1447

Query  418   t-tttttttAT-TGTTAAATGGaaaaa-a-a-TA-TAGT---TAATACTAGATAATCCGT  468
             | ||||||| | |||||| |||||||| | | || || |   ||||||||||||||  ||
Sbjct  1446  TCTTTTTTTTTATGTTAA-TGGAAAAATATAGTAATACTAGATAATACTAGATAATA-GT  1389

Query  469   AGAAGGGGGCGGGATGTAGGCCAAAGTGGATCAAGG  504
             | |||||| ||| |||||| |||| |||||||||||
Sbjct  1388  A-AAGGGG-CGG-ATGTAG-CCAA-GTGGATCAAGG  1358


>MT576958.1 Tithonia diversifolia chloroplast, complete genome
Length=151161

 Score = 573 bits (310),  Expect = 9e-159
 Identities = 457/520 (88%), Gaps = 42/520 (8%)
 Strand=Plus/Minus

Query  2    GTTATGCATGAACGTAATGCTCATAATTTCCCTCTAGACTTAGCTGCTATTGAAGCTCCA  61
            ||||||||||||||||||||||||||||||||||||||||||||||||||||||||||||
Sbjct  543  GTTATGCATGAACGTAATGCTCATAATTTCCCTCTAGACTTAGCTGCTATTGAAGCTCCA  484

Query  62   TCTACAAATGGATAAGCCTTTGGTCTGATTGTATAGGAGTTTTTGAACTAAAAAAGGAGC  121
            |||||||||||||||| |||||||||||||||||||||||||||||||||||||||||||
Sbjct  483  TCTACAAATGGATAAGACTTTGGTCTGATTGTATAGGAGTTTTTGAACTAAAAAAGGAGC  424
```

```
Query  122  AATAACGCCCTCTTGATAAAACAAGAGGGAAGCTATTGCTCCttttttt-ATTTAGTACT  180
            ||||||||||||||| |  | |||||||||||||||||||||||||||| ||||||||||
Sbjct  423  AATAACGCCCTCTTGTTTTATCAAGAGGGAAGCTATTGCTCCTTTTTTTTATTTAGTACT  364

Query  181  ATTTGCCTTACATAGTTTCTTTAAA-------AATAACAAGTACTTTTTATAGTTTGGTT  233
            |||||||||||||||||||||||||       ||||||||||||||||| ||||||||||
Sbjct  363  ATTTGCCTTACATAGTTTCTTTAAACTTTAAAAATAACAAGTACTTTTTCTAGTTTGGTT  304

Query  234  CGatttgcgtgttttttctttgtattcatttatattataggtttctatatcatttatttt  293
            |||||||  |||||||| ||||||||||||||||||||||||||||||||||| || |||
Sbjct  303  CGATTTGTTTGTTTTTTATTTGTATTCATTTATATTATAGGTTTCTATATCATATACTTT  244

Query  294  t-c----cc-aatcttttatgaagttttatttaCAATTTAAATTTCAATCGaaaaaaaga  347
            | |    || |||||||||||||||||| |||||||||| |||| |||||||||  | ||
Sbjct  243  TACTTTTCCTAATCTTTTATGAAGTTTT-TTTACAATTTCAATT-CAATCGAAACTA-GA  187

Query  348  gaaaaaatgacaaattttgctt-ttt-tga-ttttatattgattaaaatcaaaaataaag  404
             ||||| |||||| ||||| || ||| | | |||||| || ||| || | |||||| | |
Sbjct  186  TAAAAA-TGACAA-TTTTGTTTATTTATTACTTTTAT-TT-ATTCAA-T-AAAAATGA-G  134

Query  405  aaaagaattttgcttttttttATTGTTAAATGGaaaaaaaTATAGTTAATACTAGATAAT  464
            ||| |||| | ||||||||  || ||||| ||||||||  ||||| ||||||||||||||
Sbjct  133  AAA-GAATAT-GCTTTTTT--AT-GTTAA-TGGAAAAA--TATAG-TAATACTAGATAAT  83

Query  465  CCGTAGAAGGGGGCGGGATGTAGGCCAAAGTGGATCAAGG  504
              ||||| |||| ||| |||||| |||| |||||||||||
Sbjct  82   A-GTAGA-GGGG-CGG-ATGTAG-CCAA-GTGGATCAAGG  49


>KX346913.1 Xanthium sibiricum voucher S-CEZi-07 PsbA (psbA) gene, partial
cds; and psbA-trnH intergenic spacer, partial sequence; chloroplast
Length=525

 Score = 573 bits (310),  Expect = 9e-159
 Identities = 456/519 (88%), Gaps = 40/519 (8%)
 Strand=Plus/Plus

Query  5    ATGCATGAACGTAATGCTCATAATTTCCCTCTAGACTTAGCTGCTATTGAAGCTCCATCT  64
            |||||||||||||||||||||||||||||| |||||||||||||||||||||||||||||
Sbjct  1    ATGCATGAACGTAATGCTCATAATTTCCCTTTAGACTTAGCTGCTATTGAAGCTCCATCT  60

Query  65   ACAAATGGATAAGCCTTTGGTCTGATTGTATAGGAGTTTTTGAACTAAAAAAGGAGCAAT  124
            ||||||||||||| |||||||||||||||||| |||||||||||||||||||||||||||
Sbjct  61   ACAAATGGATAAGACTTTGGTCTGATTGTATAAGAGTTTTTGAACTAAAAAAGGAGCAAT  120

Query  125  AACGCCCTCTTGATAAAACAAGAGGGAAGCTATTGCTCC-tttttttATTTAGTACTATT  183
            ||||||||||||||||||||||||||||||||||||||| ||||||||||||||||||||
Sbjct  121  AACGCCCTCTTGATAAAACAAGAGGGAAGCTATTGCTCCTTTTTTTTATTTAGTACTATT  180

Query  184  TGCCTTACATAGTTTCTTTAAAAATAACAAGTACTTTTTATAGTTTGGTTCGatttgcgt  243
            ||||||||||||||||||||||||| | ||||||||||||||||||| ||  ||||||||
Sbjct  181  TGCCTTACATAGTTTCTTTAAAAATCAAAAGTACTTTTTATAGTTTGTTTTAATTTGCGT  240

Query  244  gttttttctttgtattcatttatattat--ag-----------gtttctatatcatttat  290
            ||||||||||||||||||||||||||||  |            ||||||||||||| || 
Sbjct  241  GTTTTTTCTTTGTATTCATTTATATTATTCATTTATATTATATGTTTCTATATCATATAC  300
```

```
Query  291   ttttcccaatcttttatgaagttttatttaCAATTTAAATTTCAATCGaaaaaaagagaa  350
             ||||||||||||||||||||||||||||||||| |||||||||||||||||| |  | ||
Sbjct  301   TTTTCCCAATCTTTTATGAAGTTTTATTTACAA-TTAAATTTCAATCGAAAATAT-ATAA  358

Query  351   aaaatgacaaattttgcttttttgatt-ttat-attgattaaaatcaaaa-a-taaagaa  406
             ||| | | ||||||||||||||| ||| || | ||| | |||| |||||| | |||| ||
Sbjct  359   AAA-TCA-AAATTTTGCTTTTTT-ATTATTTTGATTTAATAAA-TCAAAATAATAAA-AA  413

Query  407   aagaattttgcttttttttATTGTTAAATGGaaaaaaaTATAGTTAATACTAGATA-ATC  465
             || ||| | ||||||||| ||| |  |||||||||| ||| | | ||||||| ||| ||
Sbjct  414   AATAATAT-GCTTTTTTT-ATT-T--AATGGAAAAATATA-A-T-AATACTATATATATA  465

Query  466   CGTAGAAGGGGGCGGGATGTAGGCCAAAGTGGATCAAGG  504
              ||||| |||| ||| |||||| |||| |||||||||||
Sbjct  466   -GTAGA-GGGG-CGG-ATGTAG-CCAA-GTGGATCAAGG  498
```

>NC_053300.1 Tithonia diversifolia voucher Loeuille 678 chloroplast, complete genome
 MT700544.1 Tithonia diversifolia chloroplast, complete genome
Length=151356

```
 Score = 573 bits (310),  Expect = 9e-159
 Identities = 457/520 (88%), Gaps = 42/520 (8%)
 Strand=Plus/Minus

Query  2     GTTATGCATGAACGTAATGCTCATAATTTCCCTCTAGACTTAGCTGCTATTGAAGCTCCA  61
             ||||||||||||||||||||||||||||||||||||||||||||||||||||||||||||
Sbjct  543   GTTATGCATGAACGTAATGCTCATAATTTCCCTCTAGACTTAGCTGCTATTGAAGCTCCA  484

Query  62    TCTACAAATGGATAAGCCTTTGGTCTGATTGTATAGGAGTTTTTGAACTAAAAAAGGAGC  121
             |||||||||||||||| |||||||||||||||||||||||||||||||||||||||||||
Sbjct  483   TCTACAAATGGATAAGACTTTGGTCTGATTGTATAGGAGTTTTTGAACTAAAAAAGGAGC  424

Query  122   AATAACGCCCTCTTGATAAAACAAGAGGGAAGCTATTGCTCCttttttt-ATTTAGTACT  180
             ||||||||||||||| |  | |||||||||||||||||||||||||||| ||||||||||
Sbjct  423   AATAACGCCCTCTTGTTTTATCAAGAGGGAAGCTATTGCTCCTTTTTTTTATTTAGTACT  364

Query  181   ATTTGCCTTACATAGTTTCTTTAAA-------AATAACAAGTACTTTTTATAGTTTGGTT  233
             |||||||||||||||||||||||||       ||||||||||||||||| ||||||||||
Sbjct  363   ATTTGCCTTACATAGTTTCTTTAAACTTTAAAAATAACAAGTACTTTTTCTAGTTTGGTT  304

Query  234   CGatttgcgtgttttttctttgtattcatttatattataggtttctatatcatttatttt  293
             |||||||  |||||||| ||||||||||||||||||||||||||||||||||| || |||
Sbjct  303   CGATTTGTTTGTTTTTTATTTGTATTCATTTATATTATAGGTTTCTATATCATATACTTT  244

Query  294   t-c----cc-aatcttttatgaagttttatttaCAATTTAAATTTCAATCGaaaaaaaga  347
             | |    || |||||||||||||||||| |||||||||| |||| |||||||||  | ||
Sbjct  243   TACTTTTCCTAATCTTTTATGAAGTTTT-TTTACAATTTCAATT-CAATCGAAACTA-GA  187

Query  348   gaaaaaatgacaaattttgctt-ttt-tga-ttttatattgattaaaatcaaaaataaag  404
              ||||| |||||| ||||| || ||| | | |||||| || ||| || | |||||| | |
Sbjct  186   TAAAAA-TGACAA-TTTTGTTTATTTATTACTTTTAT-TT-ATTCAA-T-AAAAATGA-G  134

Query  405   aaaagaattttgcttttttttATTGTTAAATGGaaaaaaaTATAGTTAATACTAGATAAT  464
             ||| |||| | ||||||||  || ||||| ||||||||  ||||| ||||||||||||||
```

```
Sbjct  133  AAA-GAATAT-GCTTTTTT--AT-GTTAA-TGGAAAAA--TATAG-TAATACTAGATAAT  83

Query  465  CCGTAGAAGGGGGCGGGATGTAGGCCAAAGTGGATCAAGG  504
              ||||| |||| ||| |||||| |||| |||||||||||
Sbjct  82   A-GTAGA-GGGG-CGG-ATGTAG-CCAA-GTGGATCAAGG  49
```

>GQ248244.1 Ambrosia artemisiifolia voucher NMNH Wells 4495 psbA-trnH intergenic spacer, partial sequence; chloroplast
Length=459

Score = 573 bits (310), Expect = 9e-159
Identities = 434/487 (89%), Gaps = 38/487 (8%)
Strand=Plus/Plus

```
Query  24   ATAATTTCCCTCTAGACTTAGCTGCTATTGAAGCTCCATCTACAAATGGATAAGCCTTTG  83
            |||||||||||||||||||||||||||||||||||||||||||||||||||||| |||||
Sbjct  1    ATAATTTCCCTCTAGACTTAGCTGCTATTGAAGCTCCATCTACAAATGGATAAGACTTTG  60

Query  84   GTCTGATTGTATAGGAGTTTTTGAACTAAAAAAGGAGCAATAACGCCCTCTTGATAAAAC  143
            ||||||||||||||||||||||||||||||||||||||||||||| ||||||||||||||
Sbjct  61   GTCTGATTGTATAGGAGTTTTTGAACTAAAAAAGGAGCAATAACGTCCTCTTGATAAAAC  120

Query  144  AAGAGGGAAGCTATTGCTCC-ttttttATTTAGTACTATTTGCCTTACATAGTTTCTTT  202
            |||||||||||||||||||| |||||||||||||||||||||||||||||||||||||||
Sbjct  121  AAGAGGGAAGCTATTGCTCCTTTTTTTATTTAGTACTATTTGCCTTACATAGTTTCTTT  180

Query  203  AAAAATAACAAGTACTTTTTATAGTTTGGTTCGatttgcgtgtttttctttgtattcat  262
            |||||||         ||||||||||||||||||||||||||||||||||||||||||||
Sbjct  181  AAAAATA---------TTTTATAGTTTGGTTCGATTTGCGTGTTTTTCTTTGTATTCAT  231

Query  263  ttatattataggtttctatatcatttattttcccaatcttttatgaagttttatttaCA  322
            |||||||||||||||||||||||| || ||||||||||||||||||||||||||||||||
Sbjct  232  TTATATTATAGGTTTCTATATCATATACTTTTCCCAATCTTTTATGAAGTTTTATTTACA  291

Query  323  ATTTAAATTTCAATCGaaaaaaagagaaaaaaatgacaaattttgctt-ttt-tga-tttt  379
            |||  ||||||||||||||| | || ||||| |||||| |||| ||| ||| | | ||||
Sbjct  292  ATTC-AATTTCAATCGAAAATA-GACAAAAA-TGACAATTTTT-CTTATTTATTACTTTT  347

Query  380  atattgattaaaatcaaaa-a-taaagaaaagaattttgcttttttttATTGTTAAATGG  437
            || || |||||| | |||| | ||||||||  ||| | |||||||   ||| | || |||
Sbjct  348  AT-TT-ATTAAA-TAAAAAGAATAAAGAAAT-AATAT-GCTTTTTG--ATT-T-AA-TGG  397

Query  438  aaaaaaaTATAGTTAATACTAGATAATCCGTAGAAGGGGGCGGGATGTAGGCCAAAGTGG  497
            |||||  ||||| ||||||||||||| | ||||| |||| ||| |||||| || | ||||
Sbjct  398  AAAAA--TATAG-TAATACTAGATAA-CAGTAGA-GGGG-CGG-ATGTAG-CCNA-GTGG  448

Query  498  ATCAAGG  504
            |||||||
Sbjct  449  ATCAAGG  455
```

>OZ244507.1 Inula conyza genome assembly, organelle: plastid:chloroplast
Length=150892

Score = 573 bits (310), Expect = 9e-159
Identities = 453/516 (88%), Gaps = 34/516 (7%)

Strand=Plus/Minus

```
Query  2     GTTATGCATGAACGTAATGCTCATAATTTCCCTCTAGACTTAGCTGCTATTGAAGCTCCA  61
             ||||||||||||||||||||||| ||||||||||||||||||||||||||||||||||||
Sbjct  1850  GTTATGCATGAACGTAATGCTCACAATTTCCCTCTAGACTTAGCTGCTATTGAAGCTCCA  1791

Query  62    TCTACAAATGGATAAGCCTTTGGTCTGATTGTATAGGAGTTTTTGAACTAAAAAA-GGAG  120
             |||||||||||||||| |||||||||||||||||||||||||||||||||||||| || |
Sbjct  1790  TCTACAAATGGATAAGACTTTGGTCTGATTGTATAGGAGTTTTTGAACTAAAAAAAGGGG  1731

Query  121   CAATAACGCCCTCTTGATAAAACAAGAGGGAAGCTATTGCTCCtttttttATTTAGTACT  180
             ||||||||||||||||||||||||||||||  | ||||||||||||||||||||||||||
Sbjct  1730  CAATAACGCCCTCTTGATAAAACAAGAGGG-CGTTATTGCTCCTTTTTTTATTTAGTACT  1672

Query  181   ATTTGCCTTACATAGTTTCTTTAAAAATAACAAGTACTTTTTATAGTTTGGTTCGatttg  240
             ||||||||||||||||||||||||||||||||||  ||| |||||||||||||||||| |
Sbjct  1671  ATTTGCCTTACATAGTTTCTTTAAAAATAACAAGGGCTTCTTATAGTTTGGTTCGATTCG  1612

Query  241   cgtgttttttctttgtattcatttatattataggtttctatatcatttatttttcccaat  300
             |||||||| |||||||||||||||||||| |||||||||||||| |||     |||||||
Sbjct  1611  CGTGTTTTCTCTTTGTATTCATTTATATTGTAGGTTTCTATATCCTTT-----TCCCAAT  1557

Query  301   cttttatgaagttttatttaCAATTTAAATTTCAATCGaaaaaaagagaaaaaatgacaa  360
             ||||||||||||||||||| ||||| || ||||||||||||| | || ||||| ||| ||
Sbjct  1556  CTTTTATGAAGTTTTATTTCCAATTCAA-TTTCAATCGAAAATA-GATAAAAA-TGA-AA  1501

Query  361   attttgctttttgattttatattgattaaaatcaaaaataaagaaa-agaatt-t-tgc  417
             ||||||||| ||| |||   | || |||  | | || |||||||||| || ||  | |||
Sbjct  1500  ATTTTGCTTATTT-ATTAC-T-TTCATTTCA-TAAATAATAAAGAAATAGTATGATATGC  1445

Query  418   t-tttttttAT-TGTTAAATGGaaaaa-a-a-TA-TAGT---TAATACTAGATAATCCGT  468
             | ||||||| | |||||| |||||||| | | || || |   ||||||||||||||  ||
Sbjct  1444  TCTTTTTTTTTATGTTAA-TGGAAAAATATAGTAATACTAGATAATACTAGATAATA-GT  1387

Query  469   AGAAGGGGGCGGGATGTAGGCCAAAGTGGATCAAGG  504
             | |||||| ||| |||||| |||| |||||||||||
Sbjct  1386  A-AAGGGG-CGG-ATGTAG-CCAA-GTGGATCAAGG  1356
```

>MF348836.1 Chrysogonum virginianum isolate AG2KK87 photosystem II protein
D1 (psbA) gene, partial cds; and psbA-trnH intergenic spacer,
partial sequence; chloroplast
Length=473

Score = 573 bits (310), Expect = 9e-159
Identities = 434/489 (89%), Gaps = 28/489 (6%)
Strand=Plus/Plus

```
Query  24   ATAATTTCCCTCTAGACTTAGCTGCTATTGAAGCTCCATCTACAAATGGATAAGCCTTTG  83
            |||||||||||||||||||||||||||||||||||||||||||||||||||||| |||||
Sbjct  1    ATAATTTCCCTCTAGACTTAGCTGCTATTGAAGCTCCATCTACAAATGGATAAGACTTTG  60

Query  84   GTCTGATTGTATAGGAGTTTTTGAACTAAAAAAGGAGCAATAAC-GCCCTCTTGATAAAA  142
            |||||||||||||||||||||||||||||||| ||||||||| |  |||||||| |  |
Sbjct  61   GTCTGATTGTATAGGAGTTTTTGAACTAAAAA-GGAGCAATAGCTTCCCTCTTGTTTTAT  119

Query  143  CAAGAGGGAAGCTATTGCTCCtttttttATTTAGTACTATTTGCCTTACATAGTTTCTTT  202
```

```
                ||||||||  | ||||||||||||||||||||||||||||||||||||||||||||||||
Sbjct  120  CAAGAGGG-CGTTATTGCTCCTTTTTTTATTTAGTACTATTTGCCTTACATAGTTTCTTT  178

Query  203  AAAAATAACAAGTACTTTTTATAGTTTGGTTCGatttgcgtgtttttctttgtattcat  262
            ||||||||||||||||||||||||||||||||||||||||||||||||||||||||||||
Sbjct  179  AAAAATAACAAGTACTTTTTATAGTTTGGTTCGATTTGCGTGTTTTTCTTTGTATTCAT  238

Query  263  ttatattataggtttctatatcatttatttttcccaatcttttatgaagttttatttaCA  322
            ||||||||||||||| |||||||| || |||||||||||||||||||||||| |||||||
Sbjct  239  TTATATTATAGGTTTATATATCATATACTTTTCCCAATCTTTTATGAAGTTTGATTTACA  298

Query  323  ATTTAAATTTCAATCGaaaaaaagagaaaaaatgacaaattttgctt-ttt-tga-tttt  379
            |||  ||||||||||||||| | || ||||| |||||| |||||||| ||| | | ||||
Sbjct  299  ATTC-AATTTCAATCGAAAATA-GATAAAAA-TGACAA-TTTTGCTTATTTATTACTTTT  354

Query  380  atattgattaaaatcaaaaataaagaaaagaattttgctttttt-t-t-ATTGTTAAATG  436
            || || |||||| | ||||||||  |||  ||| | |||||||| | | | ||   |||
Sbjct  355  AT-TT-ATTAAA-T-AAAAATAAT-AAAT-AATAT-GCTTTTTTATGTTAATGAAAAAAT  407

Query  437  Gaaa-aaaaTATAGTTAATACTAGATAATCCGTAGAAGGGGGCGGATGTAGGCCAAAGT  495
             ||| ||||||||| ||||||||||||||  ||||| |||| ||| |||||| |||| ||
Sbjct  408  AAAAGAAAATATAG-TAATACTAGATAATA-GTAGA-GGGG-CGG-ATGTAG-CCAA-GT  460

Query  496  GGATCAAGG  504
            |||||||||
Sbjct  461  GGATCAAGG  469


 Score = 108 bits (58),  Expect = 1e-18
 Identities = 58/58 (100%), Gaps = 0/58 (0%)
 Strand=Plus/Minus

Query  111  AAAAAAGGAGCAATAACGCCCTCTTGATAAAACAAGAGGGAAGCTATTGCTCCttttt  168
            ||||||||||||||||||||||||||||||||||||||||||||||||||||||||||
Sbjct  145  AAAAAAGGAGCAATAACGCCCTCTTGATAAAACAAGAGGGAAGCTATTGCTCCTTTTT  88


>MT700543.1 Pappobolus lanatus var. lanatus chloroplast, complete genome
Length=151358

 Score = 573 bits (310),  Expect = 9e-159
 Identities = 457/520 (88%), Gaps = 41/520 (8%)
 Strand=Plus/Minus

Query  2    GTTATGCATGAACGTAATGCTCATAATTTCCCTCTAGACTTAGCTGCTATTGAAGCTCCA  61
            ||||||||||||||||||||||||||||||||||||||||||||||||||||||||||||
Sbjct  544  GTTATGCATGAACGTAATGCTCATAATTTCCCTCTAGACTTAGCTGCTATTGAAGCTCCA  485

Query  62   TCTACAAATGGATAAGCCTTTGGTCTGATTGTATAGGAGTTTTTGAACTAAAAAAGGAGC  121
            |||||||||||||||| |||||||||||||||||||||||||||||||||||||||||||
Sbjct  484  TCTACAAATGGATAAGACTTTGGTCTGATTGTATAGGAGTTTTTGAACTAAAAAAGGAGC  425

Query  122  AATAACGCCCTCTTGATAAAACAAGAGGGAAGCTATTGCTCCttttttt-ATTTAGTACT  180
            ||||||| ||||||| |  | |||||||||||||||||||||||||||| ||||||||||
Sbjct  424  AATAACGTCCTCTTGTTTTATCAAGAGGGAAGCTATTGCTCCTTTTTTTTATTTAGTACT  365

Query  181  ATTTGCCTTACATAGTTTCTTTAAA-------AATAACAAGTACTTTTTATAGTTTGGTT  233
```

```
            |||||||||||||||||||||||||       ||||||||||||||||||||||||||||
Sbjct  364  ATTTGCCTTACATAGTTTCTTTAAACTTTAAAAATAACAAGTACTTTTTATAGTTTGGTT  305

Query  234  CGatttgcgtgttttttctttgtattcatttatattataggt------ttctatatcatt  287
            |||||||  |||||||| ||||||||||||||||||||||||      ||||||||||| 
Sbjct  304  CGATTTGTTTGTTTTTTATTTGTATTCATTTATATTATAGGTATAGGTTTCTATATCATA  245

Query  288  tatttttcccaatcttttatgaagttttatttaCAATTTAAATTTCAATCGaaaaaaaga  347
            || |||||| |||||||||||||||||| ||||||||||||||| |||||||||| | ||
Sbjct  244  TACTTTTCCTAATCTTTTATGAAGTTTTTTTTACAATTTAAATT-CAATCGAAAATA-GA  187

Query  348  gaaaaaatgacaaattttgctt-ttt-tga-ttttatattgattaaaatcaaaaataaag  404
             ||||| |||||| ||||| || ||| | | |||||| || ||| || | |||||| | |
Sbjct  186  TAAAAA-TGACAA-TTTTGTTTATTTATTACTTTTAT-TT-ATTCAA-T-AAAAATGA-G  134

Query  405  aaaagaattttgcttttttttATTGTTAAATGGaaaaaaaTATAGTTAATACTAGATAAT  464
            ||| |||| | ||||||||  || ||||| ||||||||  ||||| |||||||| |||||
Sbjct  133  AAA-GAATAT-GCTTTTTT--AT-GTTAA-TGGAAAAA--TATAG-TAATACTATATAAT  83

Query  465  CCGTAGAAGGGGGCGGGATGTAGGCCAAAGTGGATCAAGG  504
              ||||| |||| ||| |||||| |||| |||||||||||
Sbjct  82   A-GTAGA-GGGG-CGG-ATGTAG-CCAA-GTGGATCAAGG  49
```

>NC_068630.1 Duhaldea cappa chloroplast, complete genome
OM457000.1 Duhaldea cappa chloroplast, complete genome
Length=150819

Score = 573 bits (310), Expect = 9e-159
Identities = 450/512 (88%), Gaps = 31/512 (6%)
Strand=Plus/Minus

```
Query  2    GTTATGCATGAACGTAATGCTCATAATTTCCCTCTAGACTTAGCTGCTATTGAAGCTCCA  61
            ||||||||||||||||||||||||||||||||||||||||||||||||||||||||||||
Sbjct  546  GTTATGCATGAACGTAATGCTCATAATTTCCCTCTAGACTTAGCTGCTATTGAAGCTCCA  487

Query  62   TCTACAAATGGATAAGCCTTTGGTCTGATTGTATAGGAGTTTTTGAACTAAAAAAGGAGC  121
            ||||||||||| |||| |||||||||||||||||||||||||||||||||||||||| ||
Sbjct  486  TCTACAAATGGCTAAGACTTTGGTCTGATTGTATAGGAGTTTTTGAACTAAAAAAGGGGC  427

Query  122  AATAACGCCCTCTTGATAAAACAAGAGGGAAGCTATTGCTCCtttttttATTTAGTACTA  181
            |||||||||||||||||||||||||||||  | |||||||||||||||||||||||||||
Sbjct  426  AATAACGCCCTCTTGATAAAACAAGAGGGC-GTTATTGCTCCTTTTTTTATTTAGTACTA  368

Query  182  TTTGCCTTACATAGTTTCTTTAAAAATAACAAGTACTTTTTATAGTTTGGTTCGatttgc  241
            |||||||||||||||||||||||||||||||||  ||| |||||||||||||||||| ||
Sbjct  367  TTTGCCTTACATAGTTTCTTTAAAAATAACAAGGGCTTCTTATAGTTTGGTTCGATTCGC  308

Query  242  gtgttttttctttgtattcatttatattataggtttctatatcatttatttttcccaatc  301
            ||||||| ||||||||||||||||||||||| ||||||||||| |||     ||||||||
Sbjct  307  GTGTTTTCTCTTTGTATTCATTTATATTATATGTTTCTATATCCTTT-----TCCCAATC  253

Query  302  ttttatgaagttttatttaCAATTTAAATTTCAATCGaaaaaaagagaaaaaatgacaaa  361
            |||||||||||||||||| ||||| || ||||||||||||| | || ||||  ||| |||
Sbjct  252  TTTTATGAAGTTTTATTTCCAATTCAA-TTTCAATCGAAAATA-GATAAAAG-TGA-AAA  197

Query  362  ttttgctttttttgattttatattgattaaaatcaaaaataaagaaaagaat-tttgcttt  420
```

```
            |||||||| ||| | |   | ||||||  | | || ||||||||||  ||| | | ||||
Sbjct  196  TTTTGCTTATTT-AGTAC-T-TTGATTTCA-TAAATAATAAAGAAAT-AATATGTTCTTT  142

Query  421  tttttAT-TGTTAAATGGaaaaaaaTATAGTTAATACTAGATAATCC--G-TAG-A--AG  473
            ||||| | |||||| ||||||||  ||||| |||||||||||||| |  | ||  |  ||
Sbjct  141  TTTTTTTATGTTAA-TGGAAAAA--TATAG-TAATACTAGATAATACTAGATAATATTAG  86

Query  474  -GGGGCGGGATGTAGGCCAAAGTGGATCAAGG  504
             ||||||| |||||| |||| |||||||||||
Sbjct  85   AGGGGCGG-ATGTAG-CCAA-GTGGATCAAGG  57
```

>DQ006148.1 Chrysogonum virginianum voucher Beyersdorfer 113 US PsbA (psbA)
gene, partial cds; psbA-trnH intergenic spacer, complete
sequence; and tRNA-His (trnH) gene, partial sequence; chloroplast
Length=470

Score = 573 bits (310), Expect = 9e-159
Identities = 434/489 (89%), Gaps = 28/489 (6%)
Strand=Plus/Plus

```
Query  24   ATAATTTCCCTCTAGACTTAGCTGCTATTGAAGCTCCATCTACAAATGGATAAGCCTTTG  83
            |||||||||||||||||||||||||||||||||||||||||||||||||||||| |||||
Sbjct  1    ATAATTTCCCTCTAGACTTAGCTGCTATTGAAGCTCCATCTACAAATGGATAAGACTTTG  60

Query  84   GTCTGATTGTATAGGAGTTTTTGAACTAAAAAAGGAGCAATAAC-GCCCTCTTGATAAAA  142
            |||||||||||||||||||||||||||||||| ||||||||| |  |||||||| |  |
Sbjct  61   GTCTGATTGTATAGGAGTTTTTGAACTAAAAA-GGAGCAATAGCTTCCCTCTTGTTTTAT  119

Query  143  CAAGAGGGAAGCTATTGCTCCtttttttATTTAGTACTATTTGCCTTACATAGTTTCTTT  202
            ||||||||  | ||||||||||||||||||||||||||||||||||||||||||||||||
Sbjct  120  CAAGAGGG-CGTTATTGCTCCTTTTTTTATTTAGTACTATTTGCCTTACATAGTTTCTTT  178

Query  203  AAAAATAACAAGTACTTTTTATAGTTTGGTTCGatttgcgtgtttttttctttgtattcat  262
            ||||||||||||||||||||||||||||||||||||||||||||||||||||||||||||
Sbjct  179  AAAAATAACAAGTACTTTTTATAGTTTGGTTCGATTTGCGTGTTTTTTCTTTGTATTCAT  238

Query  263  ttatattataggtttctatatcatttatttttcccaatcttttatgaagttttatttaCA  322
            ||||||||||||||| |||||||| || |||||||||||||||||||||||| |||||||
Sbjct  239  TTATATTATAGGTTTATATATCATATACTTTTCCCAATCTTTTATGAAGTTTGATTTACA  298

Query  323  ATTTAAATTTCAATCGaaaaaaagagaaaaaatgacaaattttgctt-ttt-tga-tttt  379
            |||  ||||||||||||||| | || ||||| |||||| |||||||| ||| | | ||||
Sbjct  299  ATTC-AATTTCAATCGAAAATA-GATAAAAA-TGACAA-TTTTGCTTATTTATTACTTTT  354

Query  380  atattgattaaaatcaaaaataaagaaaagaattttgctttttt-t-t-ATTGTTAAATG  436
            || || |||||| | ||||||||  |||  ||| | |||||||| | | | ||  |||
Sbjct  355  AT-TT-ATTAAA-T-AAAAATAAT-AAAT-AATAT-GCTTTTTTATGTTAATGAAAAAAT  407

Query  437  Gaaa-aaaaTATAGTTAATACTAGATAATCCGTAGAAGGGGGCGGGATGTAGGCCAAAGT  495
             ||| ||||||||| ||||||||||||||  ||||| |||| ||| |||||| |||| ||
Sbjct  408  AAAAGAAAATATAG-TAATACTAGATAATA-GTAGA-GGGG-CGG-ATGTAG-CCAA-GT  460

Query  496  GGATCAAGG  504
            |||||||||
Sbjct  461  GGATCAAGG  469
```

```
 Score = 108 bits (58),  Expect = 1e-18
 Identities = 58/58 (100%), Gaps = 0/58 (0%)
 Strand=Plus/Minus

Query  111  AAAAAAGGAGCAATAACGCCCTCTTGATAAAACAAGAGGGAAGCTATTGCTCCttttt  168
            ||||||||||||||||||||||||||||||||||||||||||||||||||||||||||
Sbjct  145  AAAAAAGGAGCAATAACGCCCTCTTGATAAAACAAGAGGGAAGCTATTGCTCCTTTTT  88


>KY940274.1 Sphagneticola trilobata chloroplast, complete genome
Length=151939

 Score = 573 bits (310),  Expect = 9e-159
 Identities = 449/511 (88%), Gaps = 30/511 (6%)
 Strand=Plus/Minus

Query  2    GTTATGCATGAACGTAATGCTCATAATTTCCCTCTAGACTTAGCTGCTATTGAAGCTCCA  61
            ||||||||||||||||||||||||||||||||||||||||||||||||||||||||||||
Sbjct  546  GTTATGCATGAACGTAATGCTCATAATTTCCCTCTAGACTTAGCTGCTATTGAAGCTCCA  487

Query  62   TCTACAAATGGATAAGCCTTTGGTCTGATTGTATAGGAGTTTTTGAACTAAAAAAGGAGC  121
             ||||||||||||||| |||||||||||||||||||||||||||||||||||||||||||
Sbjct  486  GCTACAAATGGATAAGGCTTTGGTCTGATTGTATAGGAGTTTTTGAACTAAAAAAGGAGC  427

Query  122  AATAAC-GCCCTCTTGATAAAACAAGAGGGAAGCTATTGCTCCtttttttATTTAGTACT  180
            |||| |  |||||||| |  | ||||||||  | ||||||||||||||||||||||||||
Sbjct  426  AATAGCTTCCCTCTTGTTTTATCAAGAGGG-CGTTATTGCTCCTTTTTTTATTTAGTACT  368

Query  181  ATTTGCCTTACATAGTTTCTTTAAAAATAACAAGTACTTTTTATAGTTTGGTTCGatttg  240
            |||||||||||||||||||||||||||||||||| ||||| |||||||||||||||||||
Sbjct  367  ATTTGCCTTACATAGTTTCTTTAAAAATAACAAGAACTTTCTATAGTTTGGTTCGATTTG  308

Query  241  cgtgtttttctttgtattcatttatattataggtttctatatcatttatttttcccaat  300
            ||||||||||||||||||||||||||||||||||||||||| || | || |||| |||||
Sbjct  307  CGTGTTTTTCTTTGTATTCATTTATATTATAGGTTTCTATTTC-TATACTTTTACCAAT  249

Query  301  cttttatgaagttttatttaCAATTTAAATTTCAATCGaaaaaaagagaaaaaatgacaa  360
            ||||||||||||||||||| ||||| || ||||||||||||| | || ||||| ||| ||
Sbjct  248  CTTTTATGAAGTTTTATTTCCAATTCAA-TTTCAATCGAAAATA-GATAAAAA-TGA-AA  193

Query  361  atttgctt-ttt-tgatt--ttatattgattaaaatcaa-aataa-aga-aaagaatt  413
            ||||||||| ||| | |||  || ||| ||| ||||| |  |||||| | | ||| |||
Sbjct  192  ATTTGCTTATTTATTATTACTTTTATAGATAAAAATGATTAAATAAGAAAGAAATAATA  133

Query  414  ttgcttttttttATTGTTAAATGGaaaaaaTATAGTTAATACTAGATAATCCGTAGAAG  473
            | |||||||| |   |  || ||||||||  ||||| ||||||| ||||||  ||||| |
Sbjct  132  T-GCTTTTTTGT---G--AA-TGGAAAAA--TATAG-TAATACTCGATAATA-GTAGA-G  85

Query  474  GGGGCGGGATGTAGGCCAAAGTGGATCAAGG  504
            ||| ||| |||||| |||| |||||||||||
Sbjct  84   GGG-CGG-ATGTAG-CCAA-GTGGATCAAGG  58


 Score = 110 bits (59),  Expect = 3e-19
 Identities = 59/59 (100%), Gaps = 0/59 (0%)
 Strand=Plus/Plus
```

```
Query  111  AAAAAAGGAGCAATAACGCCCTCTTGATAAAACAAGAGGGAAGCTATTGCTCCtttttt  169
            |||||||||||||||||||||||||||||||||||||||||||||||||||||||||||
Sbjct  379  AAAAAAGGAGCAATAACGCCCTCTTGATAAAACAAGAGGGAAGCTATTGCTCCTTTTTT  437
```

>NC_063729.1 Inula japonica chloroplast, complete genome
 MW115528.1 Inula japonica chloroplast, complete genome
Length=150755

 Score = 571 bits (309), Expect = 3e-158
 Identities = 452/515 (88%), Gaps = 33/515 (6%)
 Strand=Plus/Minus

```
Query  2    GTTATGCATGAACGTAATGCTCATAATTTCCCTCTAGACTTAGCTGCTATTGAAGCTCCA  61
            ||||||||||||||||||||||| ||||||||||||||||||||||||||||||||||||
Sbjct  548  GTTATGCATGAACGTAATGCTCACAATTTCCCTCTAGACTTAGCTGCTATTGAAGCTCCA  489

Query  62   TCTACAAATGGATAAGCCTTTGGTCTGATTGTATAGGAGTTTTTGAACTAAAAAA-GGAG  120
            |||||||||||||||| |||||||||||||||||||||||||||||||||||||| || |
Sbjct  488  TCTACAAATGGATAAGACTTTGGTCTGATTGTATAGGAGTTTTTGAACTAAAAAAAGGGG  429

Query  121  CAATAACGCCCTCTTGATAAAACAAGAGGGAAGCTATTGCTCCtttttttATTTAGTACT  180
            ||||||||||||||||||||||||||||||  | ||||||||||||||||||||||||||
Sbjct  428  CAATAACGCCCTCTTGATAAAACAAGAGGG-CGTTATTGCTCCTTTTTTTATTTAGTACT  370

Query  181  ATTTGCCTTACATAGTTTCTTTAAAAATAACAAGTACTTTTTATAGTTTGGTTCGatttg  240
            ||||||||||||||||||||||||||||||||||  ||| |||||||||||||||||| |
Sbjct  369  ATTTGCCTTACATAGTTTCTTTAAAAATAACAAGGGCTTCTTATAGTTTGGTTCGATTCG  310

Query  241  cgtgttttttctttgtattcatttatattataggtttctatatcatttatttttcccaat  300
            |||||||| |||||||||||||||||||| |||||||||||||| |||     |||||||
Sbjct  309  CGTGTTTTCTCTTTGTATTCATTTATATTGTAGGTTTCTATATCCTTT-----TCCCAAT  255

Query  301  cttttatgaagttttatttaCAATTTAAATTTCAATCGaaaaaaagagaaaaaatgacaa  360
            ||||||||||||||||||| ||||| || ||||||||||||| | || ||||| ||| ||
Sbjct  254  CTTTTATGAAGTTTTATTTCCAATTCAA-TTTCAATCGAAAATA-GATAAAAA-TGA-AA  199

Query  361  attttgcttttttgattttatattgattaaaatcaaaaataaagaaa-agaatt-t-tgc  417
            ||||||||| ||| |||   | || |||  | | || |||||||||| || ||  | |||
Sbjct  198  ATTTTGCTTATTT-ATTAC-T-TTCATTTCA-TAAATAATAAAGAAATAGTATCATATGC  143

Query  418  t-tttttttATTGTTAAATGGaaaaa-a-a-TA-TAGT---TAATACTAGATAATCCGTA  469
            | |||||||  |||||| |||||||| | | || || |   ||||||||||||||  |||
Sbjct  142  TCTTTTTTTTATGTTAA-TGGAAAAATATAGTAATACTAGATAATACTAGATAATA-GTA  85

Query  470  GAAGGGGGCGGGATGTAGGCCAAAGTGGATCAAGG  504
             |||||| ||| |||||| |||| |||||||||||
Sbjct  84   -AAGGGG-CGG-ATGTAG-CCAA-GTGGATCAAGG  55
```

>NC_072347.1 Pentanema britannicum chloroplast, complete genome
 ON642333.1 Pentanema britannicum chloroplast, complete genome
Length=150774

 Score = 571 bits (309), Expect = 3e-158
 Identities = 452/515 (88%), Gaps = 33/515 (6%)

```
 Strand=Plus/Minus

Query  2    GTTATGCATGAACGTAATGCTCATAATTTCCCTCTAGACTTAGCTGCTATTGAAGCTCCA  61
            ||||||||||||||||||||||| ||||||||||||||||||||||||||||||||||||
Sbjct  541  GTTATGCATGAACGTAATGCTCACAATTTCCCTCTAGACTTAGCTGCTATTGAAGCTCCA  482

Query  62   TCTACAAATGGATAAGCCTTTGGTCTGATTGTATAGGAGTTTTTGAACTAAAAAA-GGAG  120
            |||||||||||||||| |||||||||||||||||||||||||||||||||||||| || |
Sbjct  481  TCTACAAATGGATAAGACTTTGGTCTGATTGTATAGGAGTTTTTGAACTAAAAAAAGGGG  422

Query  121  CAATAACGCCCTCTTGATAAAACAAGAGGGAAGCTATTGCTCCtttttttATTTAGTACT  180
            ||||||||||||||||||||||||||||||  | ||||||||||||||||||||||||||
Sbjct  421  CAATAACGCCCTCTTGATAAAACAAGAGGG-CGTTATTGCTCCTTTTTTTATTTAGTACT  363

Query  181  ATTTGCCTTACATAGTTTCTTTAAAAATAACAAGTACTTTTTATAGTTTGGTTCGatttg  240
            ||||||||||||||||||||||||||||||||||  ||| |||||||||||||||||| |
Sbjct  362  ATTTGCCTTACATAGTTTCTTTAAAAATAACAAGGGCTTCTTATAGTTTGGTTCGATTCG  303

Query  241  cgtgttttttctttgtattcatttatattataggtttctatatcatttatttttcccaat  300
            |||||||| |||||||||||||||||||| |||||||||||||| |||     |||||||
Sbjct  302  CGTGTTTTCTCTTTGTATTCATTTATATTGTAGGTTTCTATATCCTTT-----TCCCAAT  248

Query  301  cttttatgaagttttatttaCAATTTAAATTTCAATCGaaaaaaagagaaaaaatgacaa  360
            ||||||||||||||||||| ||||| || ||||||||||||| | || ||||| ||| ||
Sbjct  247  CTTTTATGAAGTTTTATTTCCAATTCAA-TTTCAATCGAAAATA-GATAAAAA-TGA-AA  192

Query  361  attttgcttttttgattttatattgattaaaatcaaaaataaagaaa-agaatt-t-tgc  417
            ||||||||| ||| |||   | || |||  | | || |||||||||| || ||  | |||
Sbjct  191  ATTTTGCTTATTT-ATTAC-T-TTCATTTCA-TAAATAATAAAGAAATAGTATCATATGC  136

Query  418  t-tttttttATTGTTAAATGGaaaaa-a-a-TA-TAGT---TAATACTAGATAATCCGTA  469
            | |||||||  |||||| |||||||| | | || || |   ||||||||||||||  |||
Sbjct  135  TCTTTTTTTTATGTTAA-TGGAAAAATATAGTAATACTAGATAATACTAGATAATA-GTA  78

Query  470  GAAGGGGGCGGGATGTAGGCCAAAGTGGATCAAGG  504
             |||||| ||| |||||| |||| |||||||||||
Sbjct  77   -AAGGGG-CGG-ATGTAG-CCAA-GTGGATCAAGG  48


>MW770456.1 Inula helianthus-aquatilis chloroplast, complete genome
Length=150821

 Score = 571 bits (309),  Expect = 3e-158
 Identities = 452/515 (88%), Gaps = 33/515 (6%)
 Strand=Plus/Minus

Query  2    GTTATGCATGAACGTAATGCTCATAATTTCCCTCTAGACTTAGCTGCTATTGAAGCTCCA  61
            ||||||||||||||||||||||| ||||||||||||||||||||||||||||||||||||
Sbjct  548  GTTATGCATGAACGTAATGCTCACAATTTCCCTCTAGACTTAGCTGCTATTGAAGCTCCA  489

Query  62   TCTACAAATGGATAAGCCTTTGGTCTGATTGTATAGGAGTTTTTGAACTAAAAAA-GGAG  120
            |||||||||||||||| |||||||||||||||||||||||||||||||||||||| || |
Sbjct  488  TCTACAAATGGATAAGACTTTGGTCTGATTGTATAGGAGTTTTTGAACTAAAAAAAGGGG  429

Query  121  CAATAACGCCCTCTTGATAAAACAAGAGGGAAGCTATTGCTCCtttttttATTTAGTACT  180
            ||||||||||||||||||||||||||||||  | ||||||||||||||||||||||||||
Sbjct  428  CAATAACGCCCTCTTGATAAAACAAGAGGG-CGTTATTGCTCCTTTTTTTATTTAGTACT  370
```

```
Query  181  ATTTGCCTTACATAGTTTCTTTAAAAATAACAAGTACTTTTTATAGTTTGGTTCGatttg  240
            ||||||||||||||||||||||||||||||||||  ||| |||||||||||||||||| |
Sbjct  369  ATTTGCCTTACATAGTTTCTTTAAAAATAACAAGGGCTTCTTATAGTTTGGTTCGATTCG  310

Query  241  cgtgttttttctttgtattcatttatattataggtttctatatcatttatttttcccaat  300
            |||||||| |||||||||||||||||||| |||||||||||||| |||     |||||||
Sbjct  309  CGTGTTTTCTCTTTGTATTCATTTATATTGTAGGTTTCTATATCCTTT-----TCCCAAT  255

Query  301  cttttatgaagttttatttaCAATTTAAATTTCAATCGaaaaaaagagaaaaaatgacaa  360
            ||||||||||||||||||| ||||| || ||||||||||||| | || ||||| ||| ||
Sbjct  254  CTTTTATGAAGTTTTATTTCCAATTCAA-TTTCAATCGAAAATA-GATAAAAA-TGA-AA  199

Query  361  attttgcttttttgattttatattgattaaaatcaaaaataaagaaa-agaatt-t-tgc  417
            ||||||||| ||| |||   | || |||  | | || |||||||||| || ||  | |||
Sbjct  198  ATTTTGCTTATTT-ATTAC-T-TTCATTTCA-TAAATAATAAAGAAATAGTATCATATGC  143

Query  418  t-tttttttATTGTTAAATGGaaaaa-a-a-TA-TAGT---TAATACTAGATAATCCGTA  469
            | |||||||  |||||| |||||||| | | || || |   ||||||||||||||  |||
Sbjct  142  TCTTTTTTTTATGTTAA-TGGAAAAATATAGTAATACTAGATAATACTAGATAATA-GTA  85

Query  470  GAAGGGGGCGGGATGTAGGCCAAAGTGGATCAAGG  504
             |||||| ||| |||||| |||| |||||||||||
Sbjct  84   -AAGGGG-CGG-ATGTAG-CCAA-GTGGATCAAGG  55
```

>KX261063.1 Bahia ambrosioides PsbA gene, partial cds; and psbA-trnH intergenic spacer, partial sequence; chloroplast
Length=486

```
 Score = 571 bits (309),  Expect = 3e-158
 Identities = 435/492 (88%), Gaps = 24/492 (5%)
 Strand=Plus/Plus

Query  15   GTAATGCTCATAATTTCCCTCTAGACTTAGCTGCTATTGAAGCTCCATCTACAAATGGAT  74
            ||||||||| ||||||||||||||||||||||||||||||||||||||||||||||||||
Sbjct  1    GTAATGCTCGTAATTTCCCTCTAGACTTAGCTGCTATTGAAGCTCCATCTACAAATGGAT  60

Query  75   AAGCCTTTGGTCTGATTGTATAGGAGTTTTTGAACTAAAAAAGGAGCAATAACG-CCCTC  133
            ||| ||||||||||||||||||||||||||||||||||||||||||||||| |  |||||
Sbjct  61   AAGGCTTTGGTCTGATTGTATAGGAGTTTTTGAACTAAAAAAGGAGCAATAGCTTCCCTC  120

Query  134  TTGATAAAACAAGAGGGAAGCTATTGCTCC-tttttttATTTAGTACTATTTGCCTTACA  192
            ||| |  | ||||||||  | ||||||||| |||||||||||||||||||||||||||||
Sbjct  121  TTGTTTTATCAAGAGGGC-GTTATTGCTCCTTTTTTTTATTTAGTACTATTTGCCTTACA  179

Query  193  TAGTTTCTTTAAAAATAACAAGTACTTTTTATAGTTTGGTTCGatttgcgtgttttttct  252
            ||||||||||||||||||||||  |||||||||||||||||||||||||||||||| |||
Sbjct  180  TAGTTTCTTTAAAAATAACAAGGGCTTTTTATAGTTTGGTTCGATTTGCGTGTTTTCTCT  239

Query  253  ttgtattcatttatattataggtttctatatcatttatttttcccaatcttttatgaagt  312
            |||||||||||||| |||||||||||||| |||| || |||||||||||||||||||| |
Sbjct  240  TTGTATTCATTTATCTTATAGGTTTCTATCTCATATACTTTTCCCAATCTTTTATGAACT  299

Query  313  tttatttaCAATTTAAATTTCAATCGaaaaaaagagaaaaaatgacaaattttgcttttt  372
            ||| ||| |||||  ||||||||||||||||| || ||||| ||| ||||||||||| ||
Sbjct  300  TTTCTTTCCAATTC-AATTTCAATCGAAAAAA-GATAAAAA-TGA-AAATTTTGCTTATT  355
```

```
Query  373  tgatttatattgattaaaatcaaaaataaagaaaagaattttgcttttttttATTGTTA  432
            |  |||  | |||||| |  | || || |||||||| ||   |||||||||||| ||||
Sbjct  356  T--TTTACT-TTGATTTAT-TAAATAAGAAAGAAAA-AAGA-TGCTTTTTTTTAT-GTTA  408

Query  433  AATGGaaaaaaaTATAGTTAATACTAGATAATCCGTAGAAGGGGGCGGGATGTAGGCCAA  492
            | ||||||||  ||||| |||||||||||||  ||||| |||| ||| |||||| ||||
Sbjct  409  A-TGGAAAAA--TATAG-TAATACTAGATAATA-GTAGA-GGGG-CGG-ATGTAG-CCAA  459

Query  493  AGTGGATCAAGG  504
             |||||||||||
Sbjct  460  -GTGGATCAAGG  470


 Score = 110 bits (59),  Expect = 3e-19
 Identities = 59/59 (100%), Gaps = 0/59 (0%)
 Strand=Plus/Minus

Query  111  AAAAAAGGAGCAATAACGCCCTCTTGATAAAACAAGAGGGAAGCTATTGCTCCtttttt  169
            |||||||||||||||||||||||||||||||||||||||||||||||||||||||||||
Sbjct  155  AAAAAAGGAGCAATAACGCCCTCTTGATAAAACAAGAGGGAAGCTATTGCTCCTTTTTT  97


>HQ596592.1 Ambrosia artemisiifolia voucher AP337 trnH-psbA intergenic spacer,
partial sequence; chloroplast
Length=460

 Score = 571 bits (309),  Expect = 3e-158
 Identities = 435/488 (89%), Gaps = 39/488 (8%)
 Strand=Plus/Minus

Query  24   ATAATTTCCCTCTAGACTTAGCTGCTATTGAAGCTCCATCTACAAATGGATAAGCCTTTG  83
            |||||||||||||||||||||||||||||||||||||||||||||||||||||| |||||
Sbjct  460  ATAATTTCCCTCTAGACTTAGCTGCTATTGAAGCTCCATCTACAAATGGATAAGACTTTG  401

Query  84   GTCTGATTGTATAGGAGTTTTTGAACTAAAAAAGGAGCAATAACGCCCTCTTGATAAAAC  143
            ||||||||||||||||||||||||||||||||||||||||||||| ||||||||||||||
Sbjct  400  GTCTGATTGTATAGGAGTTTTTGAACTAAAAAAGGAGCAATAACGTCCTCTTGATAAAAC  341

Query  144  AAGAGGGAAGCTATTGCTCCttttttt-ATTTAGTACTATTTGCCTTACATAGTTTCTTT  202
            ||||||||||||||||||||||||||| ||||||||||||||||||||||||||||||||
Sbjct  340  AAGAGGGAAGCTATTGCTCCTTTTTTTTATTTAGTACTATTTGCCTTACATAGTTTCTTT  281

Query  203  AAAAATAACAAGTACTTTTTATAGTTTGGTTCGatttgcgtgtttttctttgtattcat  262
            |||||||         ||||||||||||||||||||||||||||||||||||||||||||
Sbjct  280  AAAAATA---------TTTTATAGTTTGGTTCGATTTGCGTGTTTTTCTTTGTATTCAT  230

Query  263  ttatattataggtttctatatcatttattttcccaatcttttatgaagttttatttaCA  322
            ||||||||||||||||||||||| || |||||||||||||||||||||||||||||||||
Sbjct  229  TTATATTATAGGTTTCTATATCATATACTTTTCCCAATCTTTTATGAAGTTTTATTTACA  170

Query  323  ATTTAAATTTCAATCGaaaaaaagagaaaaaatgacaaattttgctt-ttt-tga-tttt  379
            ||| || ||||||||||||| | || ||||| |||||| |||| ||| ||| | | ||||
Sbjct  169  ATTCAA-TTTCAATCGAAAATA-GACAAAAA-TGACAATTTTT-CTTATTTATTACTTTT  114

Query  380  atattgattaaaatcaaaa-a-taaagaaaagaattttgcttttttttATTGTTAAATGG  437
            || || |||||| | |||| | ||||||||  ||| | |||||||   ||| | || |||
```

```
Sbjct  113  AT-TT-ATTAAA-TAAAAAGAATAAAGAAAT-AATAT-GCTTTTTG--ATT-T-AA-TGG  64

Query  438  aaaaaaaTATAGTTAATACTAGATAATCCGTAGAAGGGGGCGGGATGTAGGCCAAAGTGG  497
            |||||  ||||| ||||||||||||| | ||||| |||| ||| |||||| |||| ||||
Sbjct  63   AAAAA--TATAG-TAATACTAGATAA-CAGTAGA-GGGG-CGG-ATGTAG-CCAA-GTGG  13

Query  498  ATC-AAGG  504
            ||| ||||
Sbjct  12   ATCNAAGG  5


>MW770455.1 Inula helianthus-aquatilis chloroplast, complete genome
Length=150812

 Score = 571 bits (309),  Expect = 3e-158
 Identities = 452/515 (88%), Gaps = 33/515 (6%)
 Strand=Plus/Minus

Query  2    GTTATGCATGAACGTAATGCTCATAATTTCCCTCTAGACTTAGCTGCTATTGAAGCTCCA  61
            ||||||||||||||||||||||| ||||||||||||||||||||||||||||||||||||
Sbjct  548  GTTATGCATGAACGTAATGCTCACAATTTCCCTCTAGACTTAGCTGCTATTGAAGCTCCA  489

Query  62   TCTACAAATGGATAAGCCTTTGGTCTGATTGTATAGGAGTTTTTGAACTAAAAAA-GGAG  120
            |||||||||||||||| |||||||||||||||||||||||||||||||||||||| || |
Sbjct  488  TCTACAAATGGATAAGACTTTGGTCTGATTGTATAGGAGTTTTTGAACTAAAAAAAGGGG  429

Query  121  CAATAACGCCCTCTTGATAAAACAAGAGGGAAGCTATTGCTCCtttttttATTTAGTACT  180
            ||||||||||||||||||||||||||||||  | ||||||||||||||||||||||||||
Sbjct  428  CAATAACGCCCTCTTGATAAAACAAGAGGG-CGTTATTGCTCCTTTTTTTATTTAGTACT  370

Query  181  ATTTGCCTTACATAGTTTCTTTAAAAATAACAAGTACTTTTTATAGTTTGGTTCGatttg  240
            ||||||||||||||||||||||||||||||||||  ||| |||||||||||||||||| |
Sbjct  369  ATTTGCCTTACATAGTTTCTTTAAAAATAACAAGGGCTTCTTATAGTTTGGTTCGATTCG  310

Query  241  cgtgttttttctttgtattcatttatattataggtttctatatcatttatttttcccaat  300
            |||||||| |||||||||||||||||||| |||||||||||||| |||     |||||||
Sbjct  309  CGTGTTTTCTCTTTGTATTCATTTATATTGTAGGTTTCTATATCCTTT-----TCCCAAT  255

Query  301  cttttatgaagttttatttaCAATTTAAATTTCAATCGaaaaaaagagaaaaaatgacaa  360
            ||||||||||||||||||| ||||| || ||||||||||||| | || ||||| ||| ||
Sbjct  254  CTTTTATGAAGTTTTATTTCCAATTCAA-TTTCAATCGAAAATA-GATAAAAA-TGA-AA  199

Query  361  attttgcttttttgattttatattgattaaaatcaaaaataaagaaa-agaatt-t-tgc  417
            ||||||||| ||| |||   | || |||  | | || |||||||||| || ||  | |||
Sbjct  198  ATTTTGCTTATTT-ATTAC-T-TTCATTTCA-TAAATAATAAAGAAATAGTATCATATGC  143

Query  418  t-tttttttATTGTTAAATGGaaaaa-a-a-TA-TAGT---TAATACTAGATAATCCGTA  469
            | |||||||  |||||| |||||||| | | || || |   ||||||||||||||  |||
Sbjct  142  TCTTTTTTTTATGTTAA-TGGAAAAATATAGTAATACTAGATAATACTAGATAATA-GTA  85

Query  470  GAAGGGGGCGGGATGTAGGCCAAAGTGGATCAAGG  504
             |||||| ||| |||||| |||| |||||||||||
Sbjct  84   -AAGGGG-CGG-ATGTAG-CCAA-GTGGATCAAGG  55


>NC_063571.1 Inula linariifolia chloroplast, complete genome
 MN954653.1 Inula linariifolia chloroplast, complete genome
```

```
Length=150812

 Score = 571 bits (309),  Expect = 3e-158
 Identities = 452/515 (88%), Gaps = 33/515 (6%)
 Strand=Plus/Minus

Query  2    GTTATGCATGAACGTAATGCTCATAATTTCCCTCTAGACTTAGCTGCTATTGAAGCTCCA  61
            ||||||||||||||||||||||| ||||||||||||||||||||||||||||||||||||
Sbjct  541  GTTATGCATGAACGTAATGCTCACAATTTCCCTCTAGACTTAGCTGCTATTGAAGCTCCA  482

Query  62   TCTACAAATGGATAAGCCTTTGGTCTGATTGTATAGGAGTTTTTGAACTAAAAAA-GGAG  120
            |||||||||||||||| |||||||||||||||||||||||||||||||||||||| || |
Sbjct  481  TCTACAAATGGATAAGACTTTGGTCTGATTGTATAGGAGTTTTTGAACTAAAAAAAGGGG  422

Query  121  CAATAACGCCCTCTTGATAAAACAAGAGGGAAGCTATTGCTCCtttttttATTTAGTACT  180
            ||||||||||||||||||||||||||||||  | ||||||||||||||||||||||||||
Sbjct  421  CAATAACGCCCTCTTGATAAAACAAGAGGG-CGTTATTGCTCCTTTTTTTATTTAGTACT  363

Query  181  ATTTGCCTTACATAGTTTCTTTAAAAATAACAAGTACTTTTTATAGTTTGGTTCGatttg  240
            ||||||||||||||||||||||||||||||||||  ||| |||||||||||||||||| |
Sbjct  362  ATTTGCCTTACATAGTTTCTTTAAAAATAACAAGGGCTTCTTATAGTTTGGTTCGATTCG  303

Query  241  cgtgttttttctttgtattcatttatattataggtttctatatcatttatttttcccaat  300
            |||||||| |||||||||||||||||||| |||||||||||||| |||     |||||||
Sbjct  302  CGTGTTTTCTCTTTGTATTCATTTATATTGTAGGTTTCTATATCCTTT-----TCCCAAT  248

Query  301  cttttatgaagttttatttaCAATTTAAATTTCAATCGaaaaaaagagaaaaaatgacaa  360
            ||||||||||||||||||| ||||| || ||||||||||||| | || ||||| ||| ||
Sbjct  247  CTTTTATGAAGTTTTATTTCCAATTCAA-TTTCAATCGAAAATA-GATAAAAA-TGA-AA  192

Query  361  attttgcttttttgattttatattgattaaaatcaaaaataaagaaa-agaatt-t-tgc  417
            ||||||||| ||| |||   | || |||  | | || |||||||||| || ||  | |||
Sbjct  191  ATTTTGCTTATTT-ATTAC-T-TTCATTTCA-TAAATAATAAAGAAATAGTATCATATGC  136

Query  418  t-tttttttATTGTTAAATGGaaaaa-a-a-TA-TAGT---TAATACTAGATAATCCGTA  469
            | |||||||  |||||| |||||||| | | || || |   ||||||||||||||  |||
Sbjct  135  TCTTTTTTTTATGTTAA-TGGAAAAATATAGTAATACTAGATAATACTAGATAATA-GTA  78

Query  470  GAAGGGGGCGGGATGTAGGCCAAAGTGGATCAAGG  504
             |||||| ||| |||||| |||| |||||||||||
Sbjct  77   -AAGGGG-CGG-ATGTAG-CCAA-GTGGATCAAGG  48


>MN974527.1 Inula japonica chloroplast, complete genome
Length=150754

 Score = 571 bits (309),  Expect = 3e-158
 Identities = 452/515 (88%), Gaps = 33/515 (6%)
 Strand=Plus/Minus

Query  2    GTTATGCATGAACGTAATGCTCATAATTTCCCTCTAGACTTAGCTGCTATTGAAGCTCCA  61
            ||||||||||||||||||||||| ||||||||||||||||||||||||||||||||||||
Sbjct  541  GTTATGCATGAACGTAATGCTCACAATTTCCCTCTAGACTTAGCTGCTATTGAAGCTCCA  482

Query  62   TCTACAAATGGATAAGCCTTTGGTCTGATTGTATAGGAGTTTTTGAACTAAAAAA-GGAG  120
            |||||||||||||||| |||||||||||||||||||||||||||||||||||||| || |
Sbjct  481  TCTACAAATGGATAAGACTTTGGTCTGATTGTATAGGAGTTTTTGAACTAAAAAAAGGGG  422
```

```
Query  121   CAATAACGCCCTCTTGATAAAACAAGAGGGAAGCTATTGCTCCtttttttATTTAGTACT  180
             ||||||||||||||||||||||||||||||  | ||||||||||||||||||||||||||
Sbjct  421   CAATAACGCCCTCTTGATAAAACAAGAGGG-CGTTATTGCTCCTTTTTTTATTTAGTACT  363

Query  181   ATTTGCCTTACATAGTTTCTTTAAAAATAACAAGTACTTTTTATAGTTTGGTTCGatttg  240
             ||||||||||||||||||||||||||||||||||  ||| |||||||||||||||||| |
Sbjct  362   ATTTGCCTTACATAGTTTCTTTAAAAATAACAAGGGCTTCTTATAGTTTGGTTCGATTCG  303

Query  241   cgtgtttttctttgtattcatttatattataggtttctatatcatttatttttcccaat  300
             |||||||| |||||||||||||||||||| |||||||||||||| |||     |||||||
Sbjct  302   CGTGTTTTCTCTTTGTATTCATTTATATTGTAGGTTTCTATATCCTTT-----TCCCAAT  248

Query  301   cttttatgaagttttatttaCAATTTAAATTTCAATCGaaaaaaagagaaaaaatgacaa  360
             ||||||||||||||||||| ||||| || ||||||||||||| | || ||||| ||| ||
Sbjct  247   CTTTTATGAAGTTTTATTTCCAATTCAA-TTTCAATCGAAAATA-GATAAAAA-TGA-AA  192

Query  361   attttgcttttttgattttatattgattaaaatcaaaaataaagaaa-agaatt-t-tgc  417
             ||||||||| ||| |||   | || |||  | | || |||||||||| || ||  | |||
Sbjct  191   ATTTTGCTTATTT-ATTAC-T-TTCATTTCA-TAAATAATAAAGAAATAGTATCATATGC  136

Query  418   t-tttttttATTGTTAAATGGaaaaa-a-a-TA-TAGT---TAATACTAGATAATCCGTA  469
             | |||||||  |||||| |||||||| | | || || |   ||||||||||||||  |||
Sbjct  135   TCTTTTTTTTATGTTAA-TGGAAAAATATAGTAATACTAGATAATACTAGATAATA-GTA  78

Query  470   GAAGGGGGCGGGATGTAGGCCAAAGTGGATCAAGG  504
              |||||| ||| |||||| |||| |||||||||||
Sbjct  77    -AAGGGG-CGG-ATGTAG-CCAA-GTGGATCAAGG  48
```

>NC_053284.1 Aldama kunthiana voucher voucher Silva s.n. (ESA 122873) chloroplast, complete genome
 MN337895.1 Aldama kunthiana chloroplast, complete genome
Length=151327

 Score = 569 bits (308), Expect = 1e-157
 Identities = 458/522 (88%), Gaps = 43/522 (8%)
 Strand=Plus/Minus

```
Query  2     GTTATGCATGAACGTAATGCTCATAATTTCCCTCTAGACTTAGCTGCTATTGAAGCTCCA  61
             ||||||||||||||||||||||||||||||||||||||||||||||||||||||||||||
Sbjct  548   GTTATGCATGAACGTAATGCTCATAATTTCCCTCTAGACTTAGCTGCTATTGAAGCTCCA  489

Query  62    TCTACAAATGGATAAGCCTTTGGTCTGATTGTATAGGAGTTTTTGAACTAAAAAAGGAGC  121
             |||||||||||||||| |||||||||||||||||||||||||||||||||||||||||||
Sbjct  488   TCTACAAATGGATAAGACTTTGGTCTGATTGTATAGGAGTTTTTGAACTAAAAAAGGAGC  429

Query  122   AATAACGCCCTCTTGATAAAACAAGAGGGAAGCTATTGCTCCttttttt-ATTTAGTACT  180
             ||||||||||||||| |  | |||||||||||||||||||||||||||| ||||||||||
Sbjct  428   AATAACGCCCTCTTGTTTTATCAAGAGGGAAGCTATTGCTCCTTTTTTTTATTTAGTACT  369

Query  181   ATTTGCCTTACATAGTTTCTTTAAA-------AATAACAAGTACTTTTTATAGTTTGGTT  233
             |||||||||||||||||||||||||       ||||||||||||||||||||||||||||
Sbjct  368   ATTTGCCTTACATAGTTTCTTTAAACTTTAAAAATAACAAGTACTTTTTATAGTTTGGTT  309

Query  234   CGatttgcgtgtttttctttgtattcatttatattataggtttctatatcatttatttt  293
             |||||||  |||||||| ||||||||||||||||||||||||||||||||||| || |||
```

```
Sbjct  308  CGATTTGTTTGTTTTTTATTTGTATTCATTTATATTATAGGTTTCTATATCATATACTTT  249

Query  294  t-c----cc-aatcttttatgaagttttattt--aCAATTTAAATTTCAATCGaaaaaaa  345
            | |    || |||||||||||||||||| |||  ||||| |||| |||||||||||| |
Sbjct  248  TACTTTTCCTAATCTTTTATGAAGTTTTTTTTTTACAATCTAAA-TTCAATCGAAAATA-  191

Query  346  gagaaaaaatgacaaattttgctt-ttt-tga-ttttatattgattaaaatcaaaaataa  402
            || ||||| |||||| ||||| || ||| | | |||||| || ||| || | |||||| |
Sbjct  190  GATAAAAA-TGACAA-TTTTGTTTATTTATTACTTTTAT-TT-ATTCAA-T-AAAAATGA  137

Query  403  agaaaagaattttgctttttttttATTGTTAAATGGaaaaaaaTATAGTTAATACTAGATA  462
             |||| |||| | ||||||||  || ||||| ||| ||||  ||||| ||||||||||||
Sbjct  136  -GAAA-GAATAT-GCTTTTTT--AT-GTTAA-TGGGAAAA--TATAG-TAATACTAGATA  87

Query  463  ATCCGTAGAAGGGGGCGGGATGTAGGCCAAAGTGGATCAAGG  504
            ||  ||||| |||| ||| |||||| |||| |||||||||||
Sbjct  86   ATA-GTAGA-GGGG-CGG-ATGTAG-CCAA-GTGGATCAAGG  51
```

>NC_057177.1 Aldama squalida voucher Loeuille 790 chloroplast, complete genome
 MN356031.1 Aldama squalida chloroplast, complete genome
Length=151399

 Score = 569 bits (308), Expect = 1e-157
 Identities = 458/522 (88%), Gaps = 43/522 (8%)
 Strand=Plus/Minus

```
Query  2    GTTATGCATGAACGTAATGCTCATAATTTCCCTCTAGACTTAGCTGCTATTGAAGCTCCA  61
            ||||||||||||||||||||||||||||||||||||||||||||||||||||||||||||
Sbjct  555  GTTATGCATGAACGTAATGCTCATAATTTCCCTCTAGACTTAGCTGCTATTGAAGCTCCA  496

Query  62   TCTACAAATGGATAAGCCTTTGGTCTGATTGTATAGGAGTTTTTGAACTAAAAAAGGAGC  121
            |||||||||||||||| |||||||||||||||||||||||||||||||||||||||||||
Sbjct  495  TCTACAAATGGATAAGACTTTGGTCTGATTGTATAGGAGTTTTTGAACTAAAAAAGGAGC  436

Query  122  AATAACGCCCTCTTGATAAAACAAGAGGGAAGCTATTGCTCCttttttt--ATTTAGTAC  179
            ||||||||||||||| |  | ||||||||||||||||||||||||||||  |||||||||
Sbjct  435  AATAACGCCCTCTTGTTTTATCAAGAGGGAAGCTATTGCTCCTTTTTTTTTATTTAGTAC  376

Query  180  TATTTGCCTTACATAGTTTCTTTAAA-------AATAACAAGTACTTTTTATAGTTTGGT  232
            ||||||||||||||||||||||||||       |||||||||||||||||||||||||||
Sbjct  375  TATTTGCCTTACATAGTTTCTTTAAACTTTAAAAATAACAAGTACTTTTTATAGTTTGGT  316

Query  233  TCGatttgcgtgtttttctttgtattcatttatattataggtttctatatcatttattt  292
            ||||||||  ||||||| |||||||||||||||||||||||||||||||||||| || ||
Sbjct  315  TCGATTTGTTTGTTTTTATTTGTATTCATTTATATTATAGGTTTCTATATCATATACTT  256

Query  293  tt-c----cc-aatcttttatgaagttttattt-aCAATTTAAATTTCAATCGaaaaaaa  345
            || |    || |||||||||||||||||| ||| ||||| |||| |||||||||||| |
Sbjct  255  TTACTTTTCCTAATCTTTTATGAAGTTTTTTTTTACAATCTAAA-TTCAATCGAAAATA-  198

Query  346  gagaaaaaatgacaaattttgctt-ttt-tga-ttttatattgattaaaatcaaaaataa  402
            || ||||| |||||| ||||| || ||| | | |||||| || ||| || | |||||| |
Sbjct  197  GATAAAAA-TGACAA-TTTTGTTTATTTATTACTTTTAT-TT-ATTCAA-T-AAAAATGA  144

Query  403  agaaaagaattttgctttttttttATTGTTAAATGGaaaaaaaTATAGTTAATACTAGATA  462
             |||| |||| | ||||||||  || ||||| ||| ||||  ||||| ||||||||||||
```

```
Sbjct  143  -GAAA-GAATAT-GCTTTTTT--AT-GTTAA-TGGGAAAA--TATAG-TAATACTAGATA  94

Query  463  ATCCGTAGAAGGGGGCGGGATGTAGGCCAAAGTGGATCAAGG  504
            ||  ||||| |||| ||| |||||| |||| |||||||||||
Sbjct  93   ATA-GTAGA-GGGG-CGG-ATGTAG-CCAA-GTGGATCAAGG  58


>NC_053295.1 Aldama bracteata voucher Filartiga 15 chloroplast, complete genome
 MN337907.1 Aldama bracteata chloroplast, complete genome
Length=151339

 Score = 569 bits (308),  Expect = 1e-157
 Identities = 458/522 (88%), Gaps = 43/522 (8%)
 Strand=Plus/Minus

Query  2    GTTATGCATGAACGTAATGCTCATAATTTCCCTCTAGACTTAGCTGCTATTGAAGCTCCA  61
            ||||||||||||||||||||||||||||||||||||||||||||||||||||||||||||
Sbjct  546  GTTATGCATGAACGTAATGCTCATAATTTCCCTCTAGACTTAGCTGCTATTGAAGCTCCA  487

Query  62   TCTACAAATGGATAAGCCTTTGGTCTGATTGTATAGGAGTTTTTGAACTAAAAAAGGAGC  121
            |||||||||||||||| |||||||||||||||||||||||||||||||||||||||||||
Sbjct  486  TCTACAAATGGATAAGACTTTGGTCTGATTGTATAGGAGTTTTTGAACTAAAAAAGGAGC  427

Query  122  AATAACGCCCTCTTGATAAAACAAGAGGGAAGCTATTGCTCCttttttt-ATTTAGTACT  180
            ||||||||||||||| |  | |||||||||||||||||||||||||||| ||||||||||
Sbjct  426  AATAACGCCCTCTTGTTTTATCAAGAGGGAAGCTATTGCTCCTTTTTTTTATTTAGTACT  367

Query  181  ATTTGCCTTACATAGTTTCTTTAAA-------AATAACAAGTACTTTTTATAGTTTGGTT  233
            |||||||||||||||||||||||||       ||||||||||||||||||||||||||||
Sbjct  366  ATTTGCCTTACATAGTTTCTTTAAACTTTAAAAATAACAAGTACTTTTTATAGTTTGGTT  307

Query  234  CGatttgcgtgttttttctttgtattcatttatattataggtttctatatcatttatttt  293
            |||||||  |||||||| ||||||||||||||||||||||||||||||||||| || |||
Sbjct  306  CGATTTGTTTGTTTTTATTTGTATTCATTTATATTATAGGTTTCTATATCATATACTTT  247

Query  294  t-c----cc-aatcttttatgaagttttattt--aCAATTTAAATTTCAATCGaaaaaaa  345
            | |    || |||||||||||||||||| |||  ||||| |||| |||||||||||| |
Sbjct  246  TACTTTTCCTAATCTTTTATGAAGTTTTTTTTTTACAATCTAAA-TTCAATCGAAAATA-  189

Query  346  gagaaaaaatgacaaattttgctt-ttt-tga-ttttatattgattaaaatcaaaaataa  402
            || ||||| |||||| ||||| || ||| | | |||||| || ||| || | |||||| |
Sbjct  188  GATAAAAA-TGACAA-TTTTGTTTATTTATTACTTTTAT-TT-ATTCAA-T-AAAAATGA  135

Query  403  agaaaagaattttgcttttttttATTGTTAAATGGaaaaaaaTATAGTTAATACTAGATA  462
             |||| |||| | ||||||||  || ||||| ||| ||||  ||||| ||||||||||||
Sbjct  134  -GAAA-GAATAT-GCTTTTTT--AT-GTTAA-TGGGAAAA--TATAG-TAATACTAGATA  85

Query  463  ATCCGTAGAAGGGGGCGGGATGTAGGCCAAAGTGGATCAAGG  504
            ||  ||||| |||| ||| |||||| |||| |||||||||||
Sbjct  84   ATA-GTAGA-GGGG-CGG-ATGTAG-CCAA-GTGGATCAAGG  49


>NC_057171.1 Aldama discolor voucher Bombo 72 chloroplast, complete genome
 MN356025.1 Aldama discolor chloroplast, complete genome
Length=151360

 Score = 569 bits (308),  Expect = 1e-157
```

```
 Identities = 458/522 (88%), Gaps = 43/522 (8%)
 Strand=Plus/Minus

Query  2    GTTATGCATGAACGTAATGCTCATAATTTCCCTCTAGACTTAGCTGCTATTGAAGCTCCA  61
            ||||||||||||||||||||||||||||||||||||||||||||||||||||||||||||
Sbjct  548  GTTATGCATGAACGTAATGCTCATAATTTCCCTCTAGACTTAGCTGCTATTGAAGCTCCA  489

Query  62   TCTACAAATGGATAAGCCTTTGGTCTGATTGTATAGGAGTTTTTGAACTAAAAAAGGAGC  121
            |||||||||||||||| |||||||||||||||||||||||||||||||||||||||||||
Sbjct  488  TCTACAAATGGATAAGACTTTGGTCTGATTGTATAGGAGTTTTTGAACTAAAAAAGGAGC  429

Query  122  AATAACGCCCTCTTGATAAAACAAGAGGGAAGCTATTGCTCCttttttt--ATTTAGTAC  179
            ||||||||||||||| |  | ||||||||||||||||||||||||||||  |||||||||
Sbjct  428  AATAACGCCCTCTTGTTTTATCAAGAGGGAAGCTATTGCTCCTTTTTTTTTATTTAGTAC  369

Query  180  TATTTGCCTTACATAGTTTCTTTAAA-------AATAACAAGTACTTTTTATAGTTTGGT  232
            ||||||||||||||||||||||||||       |||||||||||||||||||||||||||
Sbjct  368  TATTTGCCTTACATAGTTTCTTTAAACTTTAAAAATAACAAGTACTTTTTATAGTTTGGT  309

Query  233  TCGatttgcgtgtttttctttgtattcatttatattataggtttctatatcatttattt  292
            ||||||||  |||||||| ||||||||||||||||||||||||||||||||||| || ||
Sbjct  308  TCGATTTGTTTGTTTTTATTTGTATTCATTTATATTATAGGTTTCTATATCATATACTT  249

Query  293  tt-c----cc-aatcttttatgaagttttattt-aCAATTTAAATTTCAATCGaaaaaaa  345
            || |    || ||||||||||||||||| ||| |||||  |||| |||||||||||| |
Sbjct  248  TTACTTTTCCTAATCTTTTATGAAGTTTTTTTTACAATCTAAA-TTCAATCGAAAATA-  191

Query  346  gagaaaaaatgacaaattttgctt-ttt-tga-ttttatattgattaaaatcaaaaataa  402
            || ||||| |||||| ||||| || ||| | | |||||| || ||| || | |||||| |
Sbjct  190  GATAAAAA-TGACAA-TTTTGTTTATTTATTACTTTTAT-TT-ATTCAA-T-AAAAATGA  137

Query  403  agaaaagaattttgcttttttttATTGTTAAATGGaaaaaaaTATAGTTAATACTAGATA  462
             |||| |||| | ||||||||  || ||||| ||| ||||  ||||| ||||||||||||
Sbjct  136  -GAAA-GAATAT-GCTTTTTT--AT-GTTAA-TGGGAAAA--TATAG-TAATACTAGATA  87

Query  463  ATCCGTAGAAGGGGGCGGGATGTAGGCCAAAGTGGATCAAGG  504
            ||  ||||| |||| ||| |||||| |||| |||||||||||
Sbjct  86   ATA-GTAGA-GGGG-CGG-ATGTAG-CCAA-GTGGATCAAGG  51


>NC_053282.1 Aldama goyazii voucher Magenta 716 chloroplast, complete genome
 MN337893.1 Aldama goyazii chloroplast, complete genome
Length=151279

 Score = 569 bits (308),  Expect = 1e-157
 Identities = 458/522 (88%), Gaps = 43/522 (8%)
 Strand=Plus/Minus

Query  2    GTTATGCATGAACGTAATGCTCATAATTTCCCTCTAGACTTAGCTGCTATTGAAGCTCCA  61
            ||||||||||||||||||||||||||||||||||||||||||||||||||||||||||||
Sbjct  548  GTTATGCATGAACGTAATGCTCATAATTTCCCTCTAGACTTAGCTGCTATTGAAGCTCCA  489

Query  62   TCTACAAATGGATAAGCCTTTGGTCTGATTGTATAGGAGTTTTTGAACTAAAAAAGGAGC  121
            |||||||||||||||| |||||||||||||||||||||||||||||||||||||||||||
Sbjct  488  TCTACAAATGGATAAGACTTTGGTCTGATTGTATAGGAGTTTTTGAACTAAAAAAGGAGC  429

Query  122  AATAACGCCCTCTTGATAAAACAAGAGGGAAGCTATTGCTCCttttttt-ATTTAGTACT  180
```

```
            ||||||||||||||| |  | |||||||||||||||||||||||||||| ||||||||||
Sbjct  428  AATAACGCCCTCTTGTTTTATCAAGAGGGAAGCTATTGCTCCTTTTTTTTATTTAGTACT  369

Query  181  ATTTGCCTTACATAGTTTCTTTAAA-------AATAACAAGTACTTTTTATAGTTTGGTT  233
            |||||||||||||||||||||||||       ||||||||||||||||||||||||||||
Sbjct  368  ATTTGCCTTACATAGTTTCTTTAAACTTTAAAAATAACAAGTACTTTTTATAGTTTGGTT  309

Query  234  CGatttgcgtgttttttctttgtattcatttatattataggtttctatatcatttatttt  293
            |||||||  |||||||| ||||||||||||||||||||||||||||||||||| || |||
Sbjct  308  CGATTTGTTTGTTTTTTATTTGTATTCATTTATATTATAGGTTTCTATATCATATACTTT  249

Query  294  t-c----cc-aatcttttatgaagttttattt--aCAATTTAAATTTCAATCGaaaaaaa  345
            | |    || |||||||||||||||||| |||  ||||| |||| |||||||||||| |
Sbjct  248  TACTTTTCCTAATCTTTTATGAAGTTTTTTTTTTACAATCTAAA-TTCAATCGAAAATA-  191

Query  346  gagaaaaaatgacaaattttgctt-ttt-tga-ttttatattgattaaaatcaaaaataa  402
            || ||||| |||||| ||||| || ||| | | |||||| || ||| || | |||||| |
Sbjct  190  GATAAAAA-TGACAA-TTTTGTTTATTTATTACTTTTAT-TT-ATTCAA-T-AAAAATGA  137

Query  403  agaaaagaattttgcttttttttATTGTTAAATGGaaaaaaaTATAGTTAATACTAGATA  462
             |||| |||| | ||||||||  || ||||| ||| ||||  ||||| ||||||||||||
Sbjct  136  -GAAA-GAATAT-GCTTTTTT--AT-GTTAA-TGGGAAAA--TATAG-TAATACTAGATA  87

Query  463  ATCCGTAGAAGGGGGCGGGATGTAGGCCAAAGTGGATCAAGG  504
            ||  ||||| |||| ||| |||||| |||| |||||||||||
Sbjct  86   ATA-GTAGA-GGGG-CGG-ATGTAG-CCAA-GTGGATCAAGG  51
```

>NC_053294.1 Aldama bakeriana voucher Loeuille 867 chloroplast, complete genome
MN337906.1 Aldama bakeriana chloroplast, complete genome
Length=151376

Score = 569 bits (308), Expect = 1e-157
Identities = 458/522 (88%), Gaps = 43/522 (8%)
Strand=Plus/Minus

```
Query  2    GTTATGCATGAACGTAATGCTCATAATTTCCCTCTAGACTTAGCTGCTATTGAAGCTCCA  61
            ||||||||||||||||||||||||||||||||||||||||||||||||||||||||||||
Sbjct  546  GTTATGCATGAACGTAATGCTCATAATTTCCCTCTAGACTTAGCTGCTATTGAAGCTCCA  487

Query  62   TCTACAAATGGATAAGCCTTTGGTCTGATTGTATAGGAGTTTTTGAACTAAAAAAGGAGC  121
            ||||||||||||||| ||||||||||||||||||||||||||||||||||||||||||||
Sbjct  486  TCTACAAATGGATAAGACTTTGGTCTGATTGTATAGGAGTTTTTGAACTAAAAAAGGAGC  427

Query  122  AATAACGCCCTCTTGATAAAACAAGAGGGAAGCTATTGCTCCttttttt--ATTTAGTAC  179
            ||||||||||||||| |  | ||||||||||||||||||||||||||||  |||||||||
Sbjct  426  AATAACGCCCTCTTGTTTTATCAAGAGGGAAGCTATTGCTCCTTTTTTTTTATTTAGTAC  367

Query  180  TATTTGCCTTACATAGTTTCTTTAAA-------AATAACAAGTACTTTTTATAGTTTGGT  232
            ||||||||||||||||||||||||||       |||||||||||||||||||||||||||
Sbjct  366  TATTTGCCTTACATAGTTTCTTTAAACTTTAAAAATAACAAGTACTTTTTATAGTTTGGT  307

Query  233  TCGatttgcgtgttttttctttgtattcatttatattataggtttctatatcatttattt  292
            ||||||||  |||||||| ||||||||||||||||||||||||||||||||||| || ||
Sbjct  306  TCGATTTGTTTGTTTTTTATTTGTATTCATTTATATTATAGGTTTCTATATCATATACTT  247

Query  293  tt-c----cc-aatcttttatgaagttttattt-aCAATTTAAATTTCAATCGaaaaaaa  345
```

```
             || |    || ||||||||||||||||| ||| ||||| |||| |||||||||||| |
Sbjct  246   TTACTTTTCCTAATCTTTTATGAAGTTTTTTTTTACAATCTAAA-TTCAATCGAAAATA-  189

Query  346   gagaaaaaatgacaaattttgctt-ttt-tga-ttttatattgattaaaatcaaaaataa  402
             || ||||| |||||| ||||| || ||| | | |||||| || ||| || | |||||| |
Sbjct  188   GATAAAAA-TGACAA-TTTTGTTTATTTATTACTTTTAT-TT-ATTCAA-T-AAAAATGA  135

Query  403   agaaaagaattttgctttttttttATTGTTAAATGGaaaaaaaTATAGTTAATACTAGATA  462
              |||| |||| | ||||||||  || ||||| ||| ||||  ||||| ||||||||||||
Sbjct  134   -GAAA-GAATAT-GCTTTTTT--AT-GTTAA-TGGGAAAA--TATAG-TAATACTAGATA  85

Query  463   ATCCGTAGAAGGGGGCGGGATGTAGGCCAAAGTGGATCAAGG  504
             ||  ||||| |||| ||| |||||| |||| |||||||||||
Sbjct  84    ATA-GTAGA-GGGG-CGG-ATGTAG-CCAA-GTGGATCAAGG  49


>NC_053286.1 Aldama macrorhiza voucher Magenta 476 chloroplast, complete genome
 MN337897.1 Aldama macrorhiza voucher Magenta 476 chloroplast, complete genome
Length=151314

 Score = 569 bits (308),  Expect = 1e-157
 Identities = 458/522 (88%), Gaps = 43/522 (8%)
 Strand=Plus/Minus

Query  2     GTTATGCATGAACGTAATGCTCATAATTTCCCTCTAGACTTAGCTGCTATTGAAGCTCCA  61
             ||||||||||||||||||||||||||||||||||||||||||||||||||||||||||||
Sbjct  548   GTTATGCATGAACGTAATGCTCATAATTTCCCTCTAGACTTAGCTGCTATTGAAGCTCCA  489

Query  62    TCTACAAATGGATAAGCCTTTGGTCTGATTGTATAGGAGTTTTTGAACTAAAAAAGGAGC  121
             |||||||||||||||| |||||||||||||||||||||||||||||||||||||||||||
Sbjct  488   TCTACAAATGGATAAGACTTTGGTCTGATTGTATAGGAGTTTTTGAACTAAAAAAGGAGC  429

Query  122   AATAACGCCCTCTTGATAAAACAAGAGGGAAGCTATTGCTCCttttttt-ATTTAGTACT  180
             |||||||||||||| |  | |||||||||||||||||||||||||||| ||||||||||
Sbjct  428   AATAACGCCCTCTTGTTTTATCAAGAGGGAAGCTATTGCTCCTTTTTTTTATTTAGTACT  369

Query  181   ATTTGCCTTACATAGTTTCTTTAAA-------AATAACAAGTACTTTTTATAGTTTGGTT  233
             |||||||||||||||||||||||||       ||||||||||||||||||||||||||||
Sbjct  368   ATTTGCCTTACATAGTTTCTTTAAACTTTAAAAATAACAAGTACTTTTTATAGTTTGGTT  309

Query  234   CGatttgcgtgtttttctttgtattcatttatattataggtttctatatcatttatttt  293
             |||||||  |||||||| ||||||||||||||||||||||||||||||||||| || |||
Sbjct  308   CGATTTGTTTGTTTTTTATTTGTATTCATTTATATTATAGGTTTCTATATCATATACTTT  249

Query  294   t-c----cc-aatcttttatgaagttttattt--aCAATTTAAATTTCAATCGaaaaaaa  345
             | |    || ||||||||||||||||| |||  ||||| |||| |||||||||||| |
Sbjct  248   TACTTTTCCTAATCTTTTATGAAGTTTTTTTTTACAATCTAAA-TTCAATCGAAAATA-  191

Query  346   gagaaaaaatgacaaattttgctt-ttt-tga-ttttatattgattaaaatcaaaaataa  402
             || ||||| |||||| ||||| || ||| | | |||||| || ||| || | |||||| |
Sbjct  190   GATAAAAA-TGACAA-TTTTGTTTATTTATTACTTTTAT-TT-ATTCAA-T-AAAAATGA  137

Query  403   agaaaagaattttgctttttttttATTGTTAAATGGaaaaaaaTATAGTTAATACTAGATA  462
              |||| |||| | ||||||||  || ||||| ||| ||||  ||||| ||||||||||||
Sbjct  136   -GAAA-GAATAT-GCTTTTTT--AT-GTTAA-TGGGAAAA--TATAG-TAATACTAGATA  87

Query  463   ATCCGTAGAAGGGGGCGGGATGTAGGCCAAAGTGGATCAAGG  504
```

```
             ||  ||||| |||| ||| |||||| |||| |||||||||||
Sbjct  86   ATA-GTAGA-GGGG-CGG-ATGTAG-CCAA-GTGGATCAAGG  51
```

>NC_057181.1 Aldama veredensis voucher Loeuille 921 chloroplast, complete genome
 MN356035.1 Aldama veredensis chloroplast, complete genome
Length=151331

```
 Score = 569 bits (308),  Expect = 1e-157
 Identities = 458/522 (88%), Gaps = 43/522 (8%)
 Strand=Plus/Minus

Query  2    GTTATGCATGAACGTAATGCTCATAATTTCCCTCTAGACTTAGCTGCTATTGAAGCTCCA  61
            ||||||||||||||||||||||||||||||||||||||||||||||||||||||||||||
Sbjct  548  GTTATGCATGAACGTAATGCTCATAATTTCCCTCTAGACTTAGCTGCTATTGAAGCTCCA  489

Query  62   TCTACAAATGGATAAGCCTTTGGTCTGATTGTATAGGAGTTTTTGAACTAAAAAAGGAGC  121
            |||||||||||||||| |||||||||||||||||||||||||||||||||||||||||||
Sbjct  488  TCTACAAATGGATAAGACTTTGGTCTGATTGTATAGGAGTTTTTGAACTAAAAAAGGAGC  429

Query  122  AATAACG-CCCTCTTGATAAAACAAGAGGGAAGCTATTGCTCCtttttt-ATTTAGTAC  179
            ||||||| |||||||| |  | ||||||||||||||||||||||||||| |||||||||
Sbjct  428  AATAACGNCCCTCTTGTTTTATCAAGAGGGAAGCTATTGCTCCTTTTTTTTATTTAGTAC  369

Query  180  TATTTGCCTTACATAGTTTCTTTAAA-------AATAACAAGTACTTTTTATAGTTTGGT  232
            ||||||||||||||||||||||||||       |||||||||||||||||||||||||||
Sbjct  368  TATTTGCCTTACATAGTTTCTTTAAACTTTAAAAATAACAAGTACTTTTTATAGTTTGGT  309

Query  233  TCGatttgcgtgtttttctttgtattcatttatattataggtttctatatcatttattt  292
            ||||||||  |||||||| ||||||||||||||||||||||||||||||||||| || ||
Sbjct  308  TCGATTTGTTTGTTTTTTATTTGTATTCATTTATATTATAGGTTTCTATATCATATACTT  249

Query  293  tt-c----cc-aatcttttatgaagttttattt-aCAATTTAAATTTCAATCGaaaaaaa  345
            || |    || |||||||||||||||||| ||| ||||| |||| |||||||||||| |
Sbjct  248  TTACTTTTCCTAATCTTTTATGAAGTTTTTTTTACAATCTAAA-TTCAATCGAAAATA-  191

Query  346  gagaaaaaatgacaaattttgctt-ttt-tga-ttttatattgattaaaatcaaaaataa  402
            || ||||| |||||| ||||| || ||| | | |||||| || ||| || | |||||| |
Sbjct  190  GATAAAAA-TGACAA-TTTTGTTTATTTATTACTTTTAT-TT-ATTCAA-T-AAAAATGA  137

Query  403  agaaaagaattttgcttttttttATTGTTAAATGGaaaaaaaTATAGTTAATACTAGATA  462
             |||| |||| | ||||||||  || ||||| ||| ||||  ||||| ||||||||||||
Sbjct  136  -GAAA-GAATAT-GCTTTTTT--AT-GTTAA-TGGGAAAA--TATAG-TAATACTAGATA  87

Query  463  ATCCGTAGAAGGGGGCGGGATGTAGGCCAAAGTGGATCAAGG  504
            ||  ||||| |||| ||| |||||| |||| |||||||||||
Sbjct  86   ATA-GTAGA-GGGG-CGG-ATGTAG-CCAA-GTGGATCAAGG  51
```

>NC_057178.1 Aldama tenuifolia voucher Silva s.n. (ESA 122870) chloroplast, complete genome
 MN356032.1 Aldama tenuifolia chloroplast, complete genome
Length=151372

```
 Score = 569 bits (308),  Expect = 1e-157
 Identities = 458/522 (88%), Gaps = 43/522 (8%)
```

```
 Strand=Plus/Minus

Query  2    GTTATGCATGAACGTAATGCTCATAATTTCCCTCTAGACTTAGCTGCTATTGAAGCTCCA  61
            ||||||||||||||||||||||||||||||||||||||||||||||||||||||||||||
Sbjct  548  GTTATGCATGAACGTAATGCTCATAATTTCCCTCTAGACTTAGCTGCTATTGAAGCTCCA  489

Query  62   TCTACAAATGGATAAGCCTTTGGTCTGATTGTATAGGAGTTTTTGAACTAAAAAAGGAGC  121
            |||||||||||||||| |||||||||||||||||||||||||||||||||||||||||||
Sbjct  488  TCTACAAATGGATAAGACTTTGGTCTGATTGTATAGGAGTTTTTGAACTAAAAAAGGAGC  429

Query  122  AATAACG-CCCTCTTGATAAAACAAGAGGGAAGCTATTGCTCCttttttt-ATTTAGTAC  179
            ||||||| |||||||| |  | |||||||||||||||||||||||||||| |||||||||
Sbjct  428  AATAACGNCCCTCTTGTTTTATCAAGAGGGAAGCTATTGCTCCTTTTTTTTATTTAGTAC  369

Query  180  TATTTGCCTTACATAGTTTCTTTAAA-------AATAACAAGTACTTTTTATAGTTTGGT  232
            ||||||||||||||||||||||||||       |||||||||||||||||||||||||||
Sbjct  368  TATTTGCCTTACATAGTTTCTTTAAACTTTAAAAATAACAAGTACTTTTTATAGTTTGGT  309

Query  233  TCGatttgcgtgttttttctttgtattcatttatattataggtttctatatcatttattt  292
            ||||||||  |||||||| ||||||||||||||||||||||||||||||||||| || ||
Sbjct  308  TCGATTTGTTTGTTTTTATTTGTATTCATTTATATTATAGGTTTCTATATCATATACTT  249

Query  293  tt-c----cc-aatcttttatgaagttttattt-aCAATTTAAATTTCAATCGaaaaaaa  345
            || |    || |||||||||||||||||| ||| ||||| |||| |||||||||||| |
Sbjct  248  TTACTTTTCCTAATCTTTTATGAAGTTTTTTTTTACAATCTAAA-TTCAATCGAAAATA-  191

Query  346  gagaaaaaatgacaaattttgctt-ttt-tga-ttttatattgattaaaatcaaaaataa  402
            || ||||| |||||| ||||| || ||| | | |||||| || ||| || | |||||| |
Sbjct  190  GATAAAAA-TGACAA-TTTTGTTTATTTATTACTTTTAT-TT-ATTCAA-T-AAAAATGA  137

Query  403  agaaaagaattttgcttttttttATTGTTAAATGGaaaaaaaTATAGTTAATACTAGATA  462
             |||| |||| | ||||||||  || ||||| ||| ||||  ||||| ||||||||||||
Sbjct  136  -GAAA-GAATAT-GCTTTTTT--AT-GTTAA-TGGGAAAA--TATAG-TAATACTAGATA  87

Query  463  ATCCGTAGAAGGGGGCGGGATGTAGGCCAAAGTGGATCAAGG  504
            ||  ||||| |||| ||| |||||| |||| |||||||||||
Sbjct  86   ATA-GTAGA-GGGG-CGG-ATGTAG-CCAA-GTGGATCAAGG  51


>NC_053293.1 Aldama aspilioides voucher Filartiga 18 chloroplast, complete
genome
 MN337905.1 Viguiera aspilioides voucher Filartiga 18 chloroplast, complete
genome
Length=151362

 Score = 569 bits (308),  Expect = 1e-157
 Identities = 458/522 (88%), Gaps = 43/522 (8%)
 Strand=Plus/Minus

Query  2    GTTATGCATGAACGTAATGCTCATAATTTCCCTCTAGACTTAGCTGCTATTGAAGCTCCA  61
            ||||||||||||||||||||||||||||||||||||||||||||||||||||||||||||
Sbjct  546  GTTATGCATGAACGTAATGCTCATAATTTCCCTCTAGACTTAGCTGCTATTGAAGCTCCA  487

Query  62   TCTACAAATGGATAAGCCTTTGGTCTGATTGTATAGGAGTTTTTGAACTAAAAAAGGAGC  121
            |||||||||||||||| |||||||||||||||||||||||||||||||||||||||||||
Sbjct  486  TCTACAAATGGATAAGACTTTGGTCTGATTGTATAGGAGTTTTTGAACTAAAAAAGGAGC  427
```

```
Query  122  AATAACGCCCTCTTGATAAAACAAGAGGGAAGCTATTGCTCCttttttt-ATTTAGTACT  180
            ||||||||||||||| |  | |||||||||||||||||||||||||||| ||||||||||
Sbjct  426  AATAACGCCCTCTTGTTTTATCAAGAGGGAAGCTATTGCTCCTTTTTTTTATTTAGTACT  367

Query  181  ATTTGCCTTACATAGTTTCTTTAAA-------AATAACAAGTACTTTTTATAGTTTGGTT  233
            |||||||||||||||||||||||||       ||||||||||||||||||||||||||||
Sbjct  366  ATTTGCCTTACATAGTTTCTTTAAACTTTAAAAATAACAAGTACTTTTTATAGTTTGGTT  307

Query  234  CGatttgcgtgttttttctttgtattcatttatattataggtttctatatcatttatttt  293
            |||||||  |||||||| ||||||||||||||||||||||||||||||||||| || |||
Sbjct  306  CGATTTGTTTGTTTTTTATTTGTATTCATTTATATTATAGGTTTCTATATCATATACTTT  247

Query  294  t-c----cc-aatcttttatgaagttttattt--aCAATTTAAATTTCAATCGaaaaaaa  345
            | |    || |||||||||||||||||| |||  ||||| |||| |||||||||||| |
Sbjct  246  TACTTTTCCTAATCTTTTATGAAGTTTTTTTTTTACAATCTAAA-TTCAATCGAAAATA-  189

Query  346  gagaaaaaatgacaaattttgctt-ttt-tga-ttttatattgattaaaatcaaaaataa  402
            || ||||| |||||| ||||| || ||| | | |||||| || ||| || | |||||| |
Sbjct  188  GATAAAAA-TGACAA-TTTTGTTTATTTATTACTTTTAT-TT-ATTCAA-T-AAAAATGA  135

Query  403  agaaaagaattttgcttttttttATTGTTAAATGGaaaaaaaTATAGTTAATACTAGATA  462
             |||| |||| | ||||||||  || ||||| ||| ||||  ||||| ||||||||||||
Sbjct  134  -GAAA-GAATAT-GCTTTTTT--AT-GTTAA-TGGGAAAA--TATAG-TAATACTAGATA  85

Query  463  ATCCGTAGAAGGGGGCGGGATGTAGGCCAAAGTGGATCAAGG  504
            ||  ||||| |||| ||| |||||| |||| |||||||||||
Sbjct  84   ATA-GTAGA-GGGG-CGG-ATGTAG-CCAA-GTGGATCAAGG  49
```

>NC_086875.1 Helianthus agrestis isolate USDA PI 673199 cultivar PI 468416 chloroplast, complete genome
 PP068992.1 Helianthus agrestis isolate USDA PI 673199 cultivar PI 468416 chloroplast, complete genome
Length=151108

 Score = 568 bits (307), Expect = 4e-157
 Identities = 457/521 (88%), Gaps = 44/521 (8%)
 Strand=Plus/Minus

```
Query  2    GTTATGCATGAACGTAATGCTCATAATTTCCCTCTAGACTTAGCTGCTATTGAAGCTCCA  61
            ||||||||||||||||||||||||||||||||||||||||||||||||||||||||||||
Sbjct  544  GTTATGCATGAACGTAATGCTCATAATTTCCCTCTAGACTTAGCTGCTATTGAAGCTCCA  485

Query  62   TCTACAAATGGATAAGCCTTTGGTCTGATTGTATAGGAGTTTTTGAACTAAAAAAGGAGC  121
            |||||||||||||||| |||||||||||||||||||||||||||||||||||||||||||
Sbjct  484  TCTACAAATGGATAAGACTTTGGTCTGATTGTATAGGAGTTTTTGAACTAAAAAAGGAGC  425

Query  122  AATAACG-CCCTCTTGATAAAACAAGAGGGAAGCTATTGCTCCtttttttATTTAGTACT  180
            |||| |  ||||||||||||||||||||||  | ||||||||||||||||||||||||||
Sbjct  424  AATAGCTTCCCTCTTGATAAAACAAGAGGGC-GTTATTGCTCCTTTTTTTATTTAGTACT  366

Query  181  ATTTGCCTTACATAGTTTCTTTAAA-------AATAACAAGTACTTTTTATAGTTTGGTT  233
            |||||||||||||||||||||||||       ||||||||||||||||||||||||||||
Sbjct  365  ATTTGCCTTACATAGTTTCTTTAAACTTTAAAAATAACAAGTACTTTTTATAGTTTGGTT  306

Query  234  CGatttgcgtgttttttctttgtattcatttatattataggtttctatatcatttatttt  293
            |||||||  |||||||| ||||||||||||||||||||||||||||||||||| || |||
```

```
Sbjct  305  CGATTTGTTTGTTTTTA-TTTGTATTCATTTATATTATAGGTTTCTATATCATATACTTT  247

Query  294  t-c----cc-aatcttttatgaagttttattt-aCAATTTAAATTTCAATCGaaaaaaag  346
            | |    || |||||||||||||||||| ||| |||||||||| |||||| ||||| | |
Sbjct  246  TACTTTTCCTAATCTTTTATGAAGTTTTTTTTTACAATTTAAA-TTCAATTGAAAATA-G  189

Query  347  agaaaaaatgacaaattttgctt-ttt-tga-ttttatattgattaaaatcaaaaataaa  403
            | ||||| ||| ||||||||||| ||| | | |||||| || ||| || | |||||| |
Sbjct  188  ATAAAAA-TGA-AAATTTTGCTTATTTATTACTTTTAT-TT-ATTCAA-T-AAAAATGA-  136

Query  404  gaaaagaattttgcttttttttATTGTTAAATGGaaaaaaaTATAGTTAATACTAGATAA  463
            |||| |||  | ||||||||  || ||||| ||||||||  ||||| |||||||||||||
Sbjct  135  GAAA-GAAGAT-GCTTTTTT--AT-GTTAA-TGGAAAAA--TATAG-TAATACTAGATAA  85

Query  464  TCCGTAGAAGGGGGCGGGATGTAGGCCAAAGTGGATCAAGG  504
            |  ||||| |||| ||| |||||| |||| |||||||||||
Sbjct  84   TA-GTAGA-GGGG-CGG-ATGTAG-CCAA-GTGGATCAAGG  50


>KF746370.1 Helianthus grosseserratus isolate DB25 plastid, complete genome
Length=151045

 Score = 568 bits (307),  Expect = 4e-157
 Identities = 458/522 (88%), Gaps = 45/522 (9%)
 Strand=Plus/Minus

Query  2    GTTATGCATGAACGTAATGCTCATAATTTCCCTCTAGACTTAGCTGCTATTGAAGCTCCA  61
            ||||||||||||||||||||||||||||||||||||||||||||||||||||||||||||
Sbjct  545  GTTATGCATGAACGTAATGCTCATAATTTCCCTCTAGACTTAGCTGCTATTGAAGCTCCA  486

Query  62   TCTACAAATGGATAAGCCTTTGGTCTGATTGTATAGGAGTTTTTGAACTAAAAAAGGAGC  121
            |||||||||||||||| |||||||||||||||||||||||||||||||||||||||||||
Sbjct  485  TCTACAAATGGATAAGACTTTGGTCTGATTGTATAGGAGTTTTTGAACTAAAAAAGGAGC  426

Query  122  AATAACG-CCCTCTTGATAAAACAAGAGGGAAGCTATTGCTCC-tttttttATTTAGTAC  179
            |||| |  ||||||||||||||||||||||  | ||||||||| ||||||||||||||||
Sbjct  425  AATAGCTTCCCTCTTGATAAAACAAGAGGGC-GTTATTGCTCCTTTTTTTTATTTAGTAC  367

Query  180  TATTTGCCTTACATAGTTTCTTTAAA-------AATAACAAGTACTTTTTATAGTTTGGT  232
            ||||||||||||||||||||||||||       |||||||||||||||||||||||||||
Sbjct  366  TATTTGCCTTACATAGTTTCTTTAAACTTTAAAAATAACAAGTACTTTTTATAGTTTGGT  307

Query  233  TCGatttgcgtgttttttctttgtattcatttatattataggtttctatatcatttattt  292
            ||||||||  |||||||  ||||||||||||||||||||||||||||||||||| || ||
Sbjct  306  TCGATTTGTTTGTTTTTA-TTTGTATTCATTTATATTATAGGTTTCTATATCATATACTT  248

Query  293  tt-c----cc-aatcttttatgaagttttattt-aCAATTTAAATTTCAATCGaaaaaaa  345
            || |    || |||||||||||||||||| ||| |||||||||| |||||| ||||| |
Sbjct  247  TTACTTTTCCTAATCTTTTATGAAGTTTTTTTTTACAATTTAAA-TTCAATTGAAAATA-  190

Query  346  gagaaaaaatgacaaattttgctt-ttt-tga-ttttatattgattaaaatcaaaaataa  402
            || ||||| ||| ||||||||||| ||| | | |||||| || ||| || | |||||| |
Sbjct  189  GATAAAAA-TGA-AAATTTTGCTTATTTATTACTTTTAT-TT-ATTCAA-T-AAAAATGA  136

Query  403  agaaaagaattttgcttttttttATTGTTAAATGGaaaaaaaTATAGTTAATACTAGATA  462
             |||| |||| | ||||||||  || ||||| ||||||||  ||||| ||||||||||||
Sbjct  135  -GAAA-GAATAT-GCTTTTTT--AT-GTTAA-TGGAAAAA--TATAG-TAATACTAGATA  86
```

```
Query  463   ATCCGTAGAAGGGGGCGGGATGTAGGCCAAAGTGGATCAAGG  504
             ||  ||||| |||| ||| |||||| |||| |||||||||||
Sbjct  85    ATA-GTAGA-GGGG-CGG-ATGTAG-CCAA-GTGGATCAAGG  50
```

>OQ832296.1 Xanthium strumarium voucher MONT:87396 photosystem II protein D1 (psbA) gene, partial cds; psbA-trnH intergenic spacer, complete sequence; and tRNA-His (trnH) gene, partial sequence; chloroplast
Length=480

```
 Score = 568 bits (307),  Expect = 4e-157
 Identities = 378/410 (92%), Gaps = 14/410 (3%)
 Strand=Plus/Plus

Query  24    ATAATTTCCCTCTAGACTTAGCTGCTATTGAAGCTCCATCTACAAATGGATAAGCCTTTG  83
             |||||||||||||||||||||||||||||||||||||||||||||||||||||| |||||
Sbjct  1     ATAATTTCCCTCTAGACTTAGCTGCTATTGAAGCTCCATCTACAAATGGATAAGACTTTG  60

Query  84    GTCTGATTGTATAGGAGTTTTTGAACTAAAAAAGGAGCAATAACGCCCTCTTGATAAAAC  143
             ||||||||||||| ||||||||||||||||||||||||||||||||||||||||||||||
Sbjct  61    GTCTGATTGTATAAGAGTTTTTGAACTAAAAAAGGAGCAATAACGCCCTCTTGATAAAAC  120

Query  144   AAGAGGGAAGCTATTGCTCC-ttttttATTTAGTACTATTTGCCTTACATAGTTTCTTT  202
             |||||||||||||||||||| |||||||||||||||||||||||||||||||||||||||
Sbjct  121   AAGAGGGAAGCTATTGCTCCTTTTTTTATTTAGTACTATTTGCCTTACATAGTTTCTTT  180

Query  203   AAAAATAACAAGTACTTTTTATAGTTTGGTTCGatttgcgtgtttttctttgtattcat  262
             |||||| | ||||||||||||||||||| ||  |||||||||||||||||||||||||||
Sbjct  181   AAAAATCAAAAGTACTTTTTATAGTTTGTTTTAATTTGCGTGTTTTTCTTTGTATTCAT  240

Query  263   ttatattataggtttctatatcatttatttttcccaatcttttatgaagttttatttaCA  322
             |||||||||| ||||||||||||| || ||||||||||||||||||||||||||||||||
Sbjct  241   TTATATTATATGTTTCTATATCATATACTTTTCCCAATCTTTTATGAAGTTTTATTTACA  300

Query  323   ATTTAAATTTCAATCGaaaaaaagagaaaaaatgacaaattttgcttttttgatt-ttat  381
             | |||||||||||||||||| |  | ||||| | | ||||||||||||||| ||| || |
Sbjct  301   A-TTAAATTTCAATCGAAAATAT-ATAAAAA-TCA-AAATTTTGCTTTTTT-ATTATTTT  355

Query  382   -attgattaaaatcaaaa-a-taaagaaaagaattttgcttttttttATT  428
              ||| |||||| |||||| | |||| |||| ||| | ||||||||| |||
Sbjct  356   GATTTATTAAA-TCAAAATAATAAA-AAAATAATAT-GCTTTTTTT-ATT  401
```

>OQ434029.1 Ambrosia trifida clone 1039837_5 trnH-psbA intergenic spacer, partial sequence; and photosystem II protein D1 (psbA) gene, partial cds; plastid
Length=432

```
 Score = 568 bits (307),  Expect = 4e-157
 Identities = 413/458 (90%), Gaps = 32/458 (7%)
 Strand=Plus/Minus

Query  11    GAACGTAATGCTCATAATTTCCCTCTAGACTTAGCTGCTATTGAAGCTCCATCTACAAAT  70
             ||||||||||||||||||||||||||||||||||||||||||||||||||||||||||||
Sbjct  432   GAACGTAATGCTCATAATTTCCCTCTAGACTTAGCTGCTATTGAAGCTCCATCTACAAAT  373
```

```
Query  71   GGATAAGCCTTTGGTCTGATTGTATAGGAGTTTTTGAACTAAAAAAGGAGCAATAACGCC  130
            ||||||| ||||||||||||||||||||||||||||||||||||||||||||||||||| |
Sbjct  372  GGATAAGACTTTGGTCTGATTGTATAGGAGTTTTTGAACTAAAAAAGGAGCAATAACGTC  313

Query  131  CTCTTGATAAAACAAGAGGGAAGCTATTGCTCCttttttt-ATTTAGTACTATTTGCCTT  189
            |||||||||||||||||||||||||||||||||||||||| |||||||||||||||||||
Sbjct  312  CTCTTGATAAAACAAGAGGGAAGCTATTGCTCCTTTTTTTTATTTAGTACTATTTGCCTT  253

Query  190  ACATAGTTTCTTTAAAAATAACAAGTACTTTTTATAGTTTGGTTCGatttgcgtgttttt  249
            ||||||||||||||||||||         |||||||||||||||||||||||||||||||
Sbjct  252  ACATAGTTTCTTTAAAAATA---------TTTTATAGTTTGGTTCGATTTGCGTGTTTTT  202

Query  250  tctttgtattcatttatattataggtttctatatcatttatttttcccaatcttttatga  309
            ||||||||||||||||||||||||||||||||||||| || |||||||||||||||||||
Sbjct  201  TCTTTGTATTCATTTATATTATAGGTTTCTATATCATATACTTTTCCCAATCTTTTATGA  142

Query  310  agttttatttaCAATTTAAATTTCAATCGaaaaaaagagaaaaaatgacaaattttgctt  369
            |||||||||||||||| || ||||||||||||| | || ||||| |||||| |||| |||
Sbjct  141  AGTTTTATTTACAATTCAA-TTTCAATCGAAAATA-GACAAAAA-TGACAATTTTT-CTT  86

Query  370  -ttt-tga-ttttatattgattaaaatcaaaa-a-taaagaaaagaatttgcttttttt  424
             ||| | | |||||| || |||||| | |||| | ||||||||  ||| | |||||||
Sbjct  85   ATTTATTACTTTTAT-TT-ATTAAA-TAAAAAGAATAAAGAAAT-AATAT-GCTTTTTG-  32

Query  425  tATTGTTAAATGGaaaaaaaTATAGTTAATACTAGATA  462
             ||| | || ||||||||  |||||| |||||||||||
Sbjct  31   -ATT-T-AA-TGGAAAAA--TATAGT-AATACTAGATA  1
```

>OQ404763.1 Ambrosia artemisiifolia trnH-psbA intergenic spacer, partial sequence; and photosystem II protein D1 (psbA) gene, partial cds; plastid
Length=435

Score = 568 bits (307), Expect = 4e-157
Identities = 415/461 (90%), Gaps = 32/461 (7%)
Strand=Plus/Minus

```
Query  7    GCATGAACGTAATGCTCATAATTTCCCTCTAGACTTAGCTGCTATTGAAGCTCCATCTAC  66
            ||| ||||||||||||||||||||||||||||||||||||||||||||||||||||||||
Sbjct  435  GCACGAACGTAATGCTCATAATTTCCCTCTAGACTTAGCTGCTATTGAAGCTCCATCTAC  376

Query  67   AAATGGATAAGCCTTTGGTCTGATTGTATAGGAGTTTTTGAACTAAAAAAGGAGCAATAA  126
            ||||||||||| ||||||||||||||||||||||||||||||||||||||||||||||||
Sbjct  375  AAATGGATAAGACTTTGGTCTGATTGTATAGGAGTTTTTGAACTAAAAAAGGAGCAATAA  316

Query  127  CGCCCTCTTGATAAAACAAGAGGGAAGCTATTGCTCCttttttt-ATTTAGTACTATTTG  185
            || ||||||||||||||||||||||||||||||||||||||||| |||||||||||||||
Sbjct  315  CGTCCTCTTGATAAAACAAGAGGGAAGCTATTGCTCCTTTTTTTTATTTAGTACTATTTG  256

Query  186  CCTTACATAGTTTCTTTAAAAATAACAAGTACTTTTTATAGTTTGGTTCGatttgcgtgt  245
            ||||||||||||||||||||||||         |||||||||||||||||||||||||||
Sbjct  255  CCTTACATAGTTTCTTTAAAAATA---------TTTTATAGTTTGGTTCGATTTGCGTGT  205

Query  246  tttttctttgtattcatttatattataggtttctatatcatttatttttcccaatctttt  305
            ||||||||||||||||||||||||||||||||||||||||| || |||||||||||||||
```

```
Sbjct  204  TTTTTCTTTGTATTCATTTATATTATAGGTTTCTATATCATATACTTTTCCCAATCTTTT  145

Query  306  atgaagttttatttaCAATTTAAATTTCAATCGaaaaaaagagaaaaaatgacaaatttt  365
            |||||||||||||||||||| || ||||||||||||| | || ||||| |||||| ||||
Sbjct  144  ATGAAGTTTTATTTACAATTCAA-TTTCAATCGAAAATA-GACAAAAA-TGACAATTTTT  88

Query  366  gctt-ttt-tga-ttttatattgattaaaatcaaaa-a-taaagaaaagaattttgcttt  420
             ||| ||| | | |||||| || |||||| | |||| | ||||||||  ||| | |||||
Sbjct  87   -CTTATTTATTACTTTTAT-TT-ATTAAA-TAAAAAGAATAAAGAAAT-AATAT-GCTTT  34

Query  421  tttttATTGTTAAATGGaaaaaaaTATAGTTAATACTAGAT  461
            ||   ||| | || ||||||||  |||||| ||||||||||
Sbjct  33   TTG--ATT-T-AA-TGGAAAAA--TATAGT-AATACTAGAT  1
```

>NC_023109.1 Helianthus divaricatus isolate DB07 plastid, complete genome
 KF746352.1 Helianthus divaricatus isolate DB07 plastid, complete genome
Length=151045

 Score = 568 bits (307), Expect = 4e-157
 Identities = 458/522 (88%), Gaps = 45/522 (9%)
 Strand=Plus/Minus

```
Query  2    GTTATGCATGAACGTAATGCTCATAATTTCCCTCTAGACTTAGCTGCTATTGAAGCTCCA  61
            ||||||||||||||||||||||||||||||||||||||||||||||||||||||||||||
Sbjct  545  GTTATGCATGAACGTAATGCTCATAATTTCCCTCTAGACTTAGCTGCTATTGAAGCTCCA  486

Query  62   TCTACAAATGGATAAGCCTTTGGTCTGATTGTATAGGAGTTTTTGAACTAAAAAAGGAGC  121
            |||||||||||||||| |||||||||||||||||||||||||||||||||||||||||||
Sbjct  485  TCTACAAATGGATAAGACTTTGGTCTGATTGTATAGGAGTTTTTGAACTAAAAAAGGAGC  426

Query  122  AATAACG-CCCTCTTGATAAAACAAGAGGGAAGCTATTGCTCC-tttttttATTTAGTAC  179
            |||| |  ||||||||||||||||||||||  | ||||||||| ||||||||||||||||
Sbjct  425  AATAGCTTCCCTCTTGATAAAACAAGAGGGC-GTTATTGCTCCTTTTTTTTATTTAGTAC  367

Query  180  TATTTGCCTTACATAGTTTCTTTAAA-------AATAACAAGTACTTTTTATAGTTTGGT  232
            ||||||||||||||||||||||||||       |||||||||||||||||||||||||||
Sbjct  366  TATTTGCCTTACATAGTTTCTTTAAACTTTAAAAATAACAAGTACTTTTTATAGTTTGGT  307

Query  233  TCGatttgcgtgttttttctttgtattcatttatattataggtttctatatcatttattt  292
            ||||||||  |||||||  ||||||||||||||||||||||||||||||||||| || ||
Sbjct  306  TCGATTTGTTTGTTTTTA-TTTGTATTCATTTATATTATAGGTTTCTATATCATATACTT  248

Query  293  tt-c----cc-aatcttttatgaagttttattt-aCAATTTAAATTTCAATCGaaaaaaa  345
            || |    || |||||||||||||||||| ||| |||||||||| |||||| ||||| |
Sbjct  247  TTACTTTTCCTAATCTTTTATGAAGTTTTTTTTTACAATTTAAA-TTCAATTGAAAATA-  190

Query  346  gagaaaaaatgacaaattttgctt-ttt-tga-ttttatattgattaaaatcaaaaataa  402
            || ||||| ||| ||||||||||| ||| | | |||||| || ||| || | |||||| |
Sbjct  189  GATAAAAA-TGA-AAATTTTGCTTATTTATTACTTTTAT-TT-ATTCAA-T-AAAAATGA  136

Query  403  agaaaagaattttgcttttttttATTGTTAAATGGaaaaaaaTATAGTTAATACTAGATA  462
             |||| |||| | ||||||||  || ||||| ||||||||  ||||| ||||||||||||
Sbjct  135  -GAAA-GAATAT-GCTTTTTT--AT-GTTAA-TGGAAAAA--TATAG-TAATACTAGATA  86

Query  463  ATCCGTAGAAGGGGGCGGGATGTAGGCCAAAGTGGATCAAGG  504
            ||  ||||| |||| ||| |||||| |||| |||||||||||
```

```
Sbjct  85   ATA-GTAGA-GGGG-CGG-ATGTAG-CCAA-GTGGATCAAGG  50


  Database: Core nucleotide BLAST database
    Posted date:  Sep 11, 2025  11:25 PM
  Number of letters in database: 923,306,297,115
  Number of sequences in database:  118,282,385

Lambda      K        H
    1.33    0.621     1.12
Gapped
Lambda      K        H
    1.28    0.460    0.850
Matrix: blastn matrix:1 -2
Gap Penalties: Existence: 0, Extension: 0
Number of Sequences: 118282385
Number of Hits to DB: 0
Number of extensions: 0
Number of successful extensions: 0
Number of sequences better than 0.05: 1601
Number of HSP's better than 0.05 without gapping: 0
Number of HSP's gapped: 1924
Number of HSP's successfully gapped: 1892
Length of query: 506
Length of database: 923306297115
Length adjustment: 36
Effective length of query: 470
Effective length of database: 919048131255
Effective search space: 431952621689850
Effective search space used: 431952621689850
A: 0
X1: 13 (25.0 bits)
X2: 32 (59.1 bits)
X3: 54 (99.7 bits)
S1: 13 (25.1 bits)
S2: 29 (54.7 bits)
```
