# Supplementary material for: Organ-Resolved Salicylate–Phenylpropanoid Metabolite Profiling of Echinacea purpurea, E. pallida, and E. tennesseensis Tissues Extracted with 50% Aqueous Methanol by LC–MS/MS and LC–HRMS
Source: Molecules. 2026 Aug 25;31(17):2963. doi: 10.3390/molecules31172963 (PMC13567413; doi:10.3390/molecules31172963)
Supplement: Supplementary file 1 [file molecules-31-02963-s001.zip › SupplementaryFiles/Supplementary Table S1 .pdf]

Table S1. LC–HRMS parameters used for retrospective feature screening and supporting annotation evidence.

Proposed annotation	Acquisition mode	Candidate formula	Theoretical target *m/z* , [M−H]⁻	Monitored product ion(s), *m/z*	RT (min)	Extraction window (±ppm)	Trace ID	Annotation status and limitation
Methyl salicylate candidate	Targeted MS/MS	$C_8H_8O_3$	151.0401	136.0166; 137.0244; 119.0139; 93.0346; 65.0391	9.1	5	Trace 1	Putative MS/MS-supported annotation; no authentic-standard RT confirmation
trans -Cinnamic acid candidate	Targeted MS/MS	$C_9H_8O_2$	147.0452	103.0553; 89.0397; 77.0397; 65.0391	6.6	15	Trace 2	Putative MS/MS-supported annotation; wider window used for retrospective extraction
SA hexoside candidate, feature 1	Targeted MS/MS	$C_{13}H_{16}O_8$	299.0772	137.0234	0.92	10	Trace 3	Tentatively assigned as SA 2-*O* -glucoside; positional and linkage isomers unresolved
SA hexoside candidate, feature 2	Targeted MS/MS	$C_{13}H_{16}O_8$	299.0772	137.0234	1.53	10	Trace 3	Tentatively assigned as SA 5-*O* -glucoside; positional and linkage isomers unresolved
SA hexose-ester candidate	Targeted MS/MS	$C_{13}H_{16}O_8$	299.0772	93.033	1.26	10	Trace 4	Tentatively assigned as SA glucose ester; isomer unresolved
Chorismate-related candidate, feature 1	Full-scan MS	$C_{10}H_{10}O_6$	225.0405	Not available	2.07	10	Trace 5	Tentatively assigned as isochorismate based on exact mass and retention order; isomers unresolved
Chorismate-related candidate, feature 2	Full-scan MS	$C_{10}H_{10}O_6$	225.0405	Not available	2.65	10	Trace 5	Tentatively assigned as chorismate; isomers unresolved
Chorismate-related candidate, feature 3	Full-scan MS	$C_{10}H_{10}O_6$	225.0405	Not available	3.3	10	Trace 5	Tentatively assigned as prephenate; isomers unresolved
Isochorismate-9-glutamate candidate	Targeted MS/MS	$C_{15}H_{17}NO_9$	354.0836	174.076	0.69	5	Trace 6	Putative MS/MS-supported candidate; proposed product ion requires standard confirmation

Caffeic acid candidate	Full-scan MS	$C_9H_8O_4$	179.035	Not available	2.22	10	Trace 7	Tentative exact-mass candidate; 10 ppm window required for consistent extraction
p -Coumaric acid candidate	Full-scan MS	$C_9H_8O_3$	163.0401	Not available	3.31	8	Trace 8	Tentative exact-mass candidate; wider window required for consistent extraction
Z -Sinapic acid candidate	Full-scan MS	$C_{11}H_{12}O_5$	223.0612	Not available	2.71	5	Trace 9	Tentative stereoisomer assignment; *E/Z* configuration not confirmed
E -Sinapic acid candidate	Full-scan MS	$C_{11}H_{12}O_5$	223.0612	Not available	6.14	5	Trace 9	Tentative stereoisomer assignment; *E/Z* configuration not confirmed
Caffeoylquinic-acid isomer, feature 1	Full-scan MS	$C_{16}H_{18}O_9$	353.0878	Not available	1.17	5	Trace 10	Tentatively assigned as neochlorogenic acid; positional isomer unresolved
Caffeoylquinic-acid isomer, feature 2	Full-scan MS	$C_{16}H_{18}O_9$	353.0878	Not available	1.62	5	Trace 10	Tentatively assigned as chlorogenic acid; positional isomer unresolved
Caffeoylquinic-acid isomer, feature 3	Full-scan MS	$C_{16}H_{18}O_9$	353.0878	Not available	1.72	5	Trace 10	Tentatively assigned as *cis* -5-*O* -caffeoylquinic acid; configuration unresolved
Caffeoylquinic-acid isomer, feature 4	Full-scan MS	$C_{16}H_{18}O_9$	353.0878	Not available	2.4	5	Trace 10	Tentatively assigned as cryptochlorogenic acid; positional isomer unresolved
Dicaffeoylquinic-acid isomer, feature 1	Full-scan MS	$C_{25}H_{24}O_{12}$	515.1195	Not available	4.68	5	Trace 11	Tentatively assigned as 3,4-dicaffeoylquinic acid; positional isomer unresolved
Dicaffeoylquinic-acid isomer, feature 2	Full-scan MS	$C_{25}H_{24}O_{12}$	515.1195	Not available	4.87	5	Trace 11	Tentatively assigned as 3,5-dicaffeoylquinic acid; positional isomer unresolved
Dicaffeoylquinic-acid isomer, feature 3	Full-scan MS	$C_{25}H_{24}O_{12}$	515.1195	Not available	5.41	5	Trace 11	Tentatively assigned as 4,5-dicaffeoylquinic acid; positional isomer unresolved

Caftaric acid candidate	Full-scan MS	$C_{13}H_{12}O_9$	311.0409	Not available	1.5	5	Trace 12	Tentative exact-mass candidate
Cichoric acid candidate	Full-scan MS	$C_{22}H_{18}O_{12}$	473.0725	Not available	0.63	5	Trace 13	Tentative exact-mass candidate
Quercetin candidate	Full-scan MS	$C_{15}H_{10}O_7$	301.0354	Not available	7.12	5	Trace 14	Tentative exact-mass candidate
Luteolin/kaempferol isomer candidate, feature 1	Full-scan MS	$C_{15}H_{10}O_6$	285.0405	Not available	7.11	5	Trace 15	Tentatively assigned as luteolin; structural isomers unresolved
Luteolin/kaempferol isomer candidate, feature 2	Full-scan MS	$C_{15}H_{10}O_6$	285.0405	Not available	8.64	5	Trace 15	Tentatively assigned as kaempferol; structural isomers unresolved
Apigenin candidate	Full-scan MS	$C_{15}H_{10}O_5$	269.0455	Not available	8.39	5	Trace 16	Tentative exact-mass candidate
Hydroxybenzoic-acid isomer, feature 1	Full-scan MS	$C_7H_6O_3$	137.0244	Not available	1.81	10	Trace 17	Tentatively associated with SA; LC–HRMS assignment independent of targeted SA confirmation
Hydroxybenzoic-acid isomer, feature 2	Full-scan MS	$C_7H_6O_3$	137.0244	Not available	5.68	10	Trace 17	Tentatively assigned as *p* -HBA; isomer unresolved by full-scan evidence alone
Dihydroxybenzoic-acid isomer, feature 1	Full-scan MS	$C_7H_6O_4$	153.0193	Not available	1.27	10	Trace 18	Tentatively assigned as gentisic acid; positional isomer unresolved
Dihydroxybenzoic-acid isomer, feature 2	Full-scan MS	$C_7H_6O_4$	153.0193	Not available	2.2	5	Trace 18	Tentatively assigned as 2,3-dihydroxybenzoic acid; positional isomer unresolved
Dihydroxybenzoic-acid isomer, feature 3	Full-scan MS	$C_7H_6O_4$	153.0193	Not available	2.84	5	Trace 18	Tentatively assigned as protocatechuic acid; positional isomer unresolved
Salicyloyl-malate candidate	Full-scan MS	$C_{11}H_{10}O_7$	253.0354	Not available	3.46	5	Trace 19	Tentative exact-mass candidate; structural isomers unresolved
Sulfated-salicylate candidate	Full-scan MS	$C_7H_6O_6S$	216.9812	Not available	0.7	15	Trace 20	Formula-level candidate; wider window and lack of MS/MS require cautious interpretation
Apigenin-*O* -hexoside candidate	Full-scan MS	$C_{21}H_{20}O_{10}$	431.0984	Not available	3.97	5	Trace 21	Tentative compound-class assignment; sugar linkage unresolved

Luteolin-type diglycoside candidates	Full-scan MS	$C_{27}H_{30}O_{15}$	593.1512	Not available	2.31; 3.54	5	Trace 22	Tentative compound-class assignment; aglycone, sugar identity, and linkage unresolved
Kaempferol-type diglycoside candidates	Full-scan MS	$C_{27}H_{30}O_{15}$	593.1512	Not available	4.33; 4.64; 4.91	5	Trace 22	Tentative compound-class assignment; isomeric with other flavonoid diglycosides
Apigenin-type diglycoside candidates	Full-scan MS	$C_{26}H_{28}O_{14}$	563.1406	Not available	3.06; 5.09	5	Trace 23	Tentative compound-class assignment; sugar identity and linkage unresolved
Luteolin/kaempferol-*O* -hexoside candidates, features 1–2	Full-scan MS	$C_{21}H_{20}O_{11}$	447.0933	Not available	3.10; 3.27	5	Trace 24	Tentatively assigned as luteolin-*O* -hexosides; isomeric aglycones and linkages unresolved
Luteolin/kaempferol-*O* -hexoside candidates, features 3–4	Full-scan MS	$C_{21}H_{20}O_{11}$	447.0933	Not available	3.55; 4.92	5	Trace 24	Tentatively assigned as kaempferol-*O* -hexosides; isomeric aglycones and linkages unresolved
2-Hydroxyacryloyl-*N* -glutamate candidate	Full-scan MS	$C_8H_{11}NO_6$	216.0514	Not available	0.86	5	Trace 25	Formula-level candidate; proposed relationship to I9G requires confirmation
Ferulic acid candidate	Full-scan MS	$C_{10}H_{10}O_4$	193.0506	Not available	5.65	5	Trace 26	Tentative exact-mass candidate
Sinapoyl-hexose candidates	Full-scan MS	$C_{17}H_{22}O_{10}$	385.114	Not available	1.55; 2.09; 2.40; 2.49; 2.77; 2.91; 3.25; 3.65; 3.87; 4.01	5	Trace 27	Tentative compound-class assignment; multiple structural and linkage isomers
Sinapoyl-malate isomer, feature 1	Full-scan MS	$C_{15}H_{16}O_9$	339.0722	Not available	1.29	5	Trace 28	Tentatively assigned as *Z* -sinapoyl malate; configuration not confirmed
Sinapoyl-malate isomer, feature 2	Full-scan MS	$C_{15}H_{16}O_9$	339.0722	Not available	6.15	5	Trace 28	Tentatively assigned as *E* -sinapoyl malate; configuration not confirmed
Caffeoyl-shikimate candidate	Full-scan MS	$C_{16}H_{16}O_8$	335.0772	Not available	2.62	5	Trace 29	Tentative exact-mass candidate

Caffeoyl–feruloyl-malate-related candidates	Full-scan MS	$C_{25}H_{19}O_{12}$	510.0804	Not available	3.09; 3.29; 4.25; 5.24; 5.38; 5.66	5	Trace 30	Formula and proposed structural class require verification; multiple isomers unresolved

Abbreviations: MS/MS, tandem mass spectrometry; RT, retention time; SA, salicylic acid; p-HBA, p-hydroxybenzoic acid; I9G, isochorismate-9-glutamate.

Ion assignment: Theoretical precursor values were calculated as monoisotopic $[M-H]^-$ ions from the listed neutral molecular formulas. Product-ion values are reported separately for targeted MS/MS features. Precursor ions were extracted at their theoretical target m/z.

Extraction window: Values represent the extracted-ion windows used for retrospective feature screening and should not be interpreted as observed mass errors or instrument-accuracy specifications. Most features were screened at ±5 ppm. Wider windows of ±8–15 ppm were applied selectively to predefined low-m/z, low-intensity, or highly abundant features that were not consistently captured at ±5 ppm. High ion populations in complex extracts may produce space-charge or ion-interaction effects that cause small shifts in apparent m/z. All candidate peaks were manually reviewed, and use of a wider window did not increase annotation confidence.

Annotation confidence: Proposed annotations were based on accurate mass, candidate elemental composition, retention behavior, isotopic pattern, and MS/MS fragments where available. Unless an authentic reference standard was analyzed under identical conditions, compound names represent tentative candidates or putative annotations rather than confirmed identities. Retention order alone was not considered sufficient to distinguish positional, structural, or stereochemical isomers.

Sample-level measurements: Observed precursor m/z and mass error varied among samples and duplicate injections and are therefore not listed individually in this screening-parameter table. Retained signals were manually reviewed for chromatographic peak shape, retention behavior, isotope pattern, duplicate-injection consistency, and MS/MS evidence where available. Use of a wider extraction window did not increase annotation confidence.
