# Supplementary material for: Organ-Resolved Salicylate–Phenylpropanoid Metabolite Profiling of Echinacea purpurea, E. pallida, and E. tennesseensis Tissues Extracted with 50% Aqueous Methanol by LC–MS/MS and LC–HRMS
Source: Molecules. 2026 Aug 25;31(17):2963. doi: 10.3390/molecules31172963 (PMC13567413; doi:10.3390/molecules31172963)
Supplement: Supplementary file 1 [file molecules-31-02963-s001.zip › SupplementaryFiles/Supplementary Table S2.pdf]

Table S2. Full retrospective LC–HRMS extracted features

Full retrospective LC–HRMS extracted-ion peak areas for salicylate-pathway, phenylpropanoid, caffeic-acid derivative, flavonoid and sugar-related features across *Echinacea* species, tissues and sample IDs. Peak areas are non-calibrated HRMS signal intensities and are intended for relative comparison only.

Index	Name	RT (Min)	RT (Sec)	Start RT	End RT	Base Peak	Peak Area	Peak Height	Baseline Width	SignalToNoise	Scan Filter	TraceId	% Area	% Height	Integration Method	Sample#	Species	Tissue	Putative Feature Assignment	Idnetifcation confidence
1		0.9046995	54.281969	0.8944	0.915016	137.023819	1921.36443	3662.747413	0.02061575	INF	No Filter	Trace1	18.85	24.23	Automated - Genesis	119651	Echinacea purpurea	Roots		
2		1.3736885	82.42131	1.363424	1.402935	137.023834	953.609474	934.6550003	0.0395108	INF	No Filter	Trace1	9.35	6.18	Automated - Genesis	119651	Echinacea purpurea	Roots		
3		3.8456739	230.74043	3.835441	3.857514	137.023788	302.394123	578.6891243	0.0220729	INF	No Filter	Trace1	2.97	3.83	Automated - Genesis	119651	Echinacea purpurea	Roots		
4		5.4336092	326.01655	5.422869	5.445962	136.016174	222.421768	390.2684068	0.0230937	INF	No Filter	Trace1	2.18	2.58	Automated - Genesis	119651	Echinacea purpurea	Roots		
5		10.259712	615.58272	10.24577	10.2745	137.023956	980.490981	1380.124342	0.028731	INF	No Filter	Trace1	9.62	9.13	Automated - Genesis	119651	Echinacea purpurea	Roots		
6		10.341883	620.51298	10.32794	10.35582	137.023834	1166.84057	1645.436027	0.027882	INF	No Filter	Trace1	11.45	10.89	Automated - Genesis	119651	Echinacea purpurea	Roots		
7		10.393846	623.63076	10.37991	10.40779	137.023758	1070.40904	1509.421648	0.027882	INF	No Filter	Trace1	10.5	9.99	Automated - Genesis	119651	Echinacea purpurea	Roots		
8		10.948102	656.88612	10.93332	10.96204	137.023849	1204.11039	1668.927019	0.028728	INF	No Filter	Trace1	11.81	11.04	Automated - Genesis	119651	Echinacea purpurea	Roots		
9		11.206213	672.37278	11.19227	11.22015	137.023773	1099.98334	1551.166047	0.027882	INF	No Filter	Trace1	10.79	10.26	Automated - Genesis	119651	Echinacea purpurea	Roots		
10		13.306897	798.41382	13.29296	13.32084	137.023788	1272.35181	1794.192084	0.027881	INF	No Filter	Trace1	12.48	11.87	Automated - Genesis	119651	Echinacea purpurea	Roots		
1		2.3881617	143.2897	2.377896	2.39843	103.055504	774.486317	1482.999514	0.0205344	INF	No Filter	Trace2	100	100	Automated - Genesis	119651	Echinacea purpurea	Roots		
1		1.8081237	108.48742	1.697811	2.370321	137.023193	1074947.59	91268.31028	0.67250963	36.08434547	No Filter	Trace3	61.5	70.65	Manual	119651	Echinacea purpurea	Roots		
2		2.4229221	145.37533	2.404334	3.018988	137.023102	672799.022	37921.75307	0.6146541	14.38269889	No Filter	Trace3	38.5	29.35	Manual	119651	Echinacea purpurea	Roots		
1		0.9173562	55.041373	0.901891	0.94472	93.0331192	4360.03934	3629.40932	0.04282913	INF	No Filter	Trace4	6.76	14.58	Automated - Genesis	119651	Echinacea purpurea	Roots		
2		1.5526723	93.160338	1.502079	1.678527	0	41138.2187	10113.02149	0.176448	INF	No Filter	Trace4	63.74	40.63	Automated - Genesis	119651	Echinacea purpurea	Roots		
3		4.364987	261.89922	4.300452	4.378799	0	2377.97397	1052.747671	0.0783468	INF	No Filter	Trace4	3.68	4.23	Automated - Genesis	119651	Echinacea purpurea	Roots		
4		4.5098081	270.58849	4.407962	4.572276	93.0334244	5084.9936	1280.302694	0.1643137	INF	No Filter	Trace4	7.88	5.14	Automated - Genesis	119651	Echinacea purpurea	Roots		
5		4.6206398	277.23839	4.575856	4.633213	0	1905.17102	1193.71533	0.057357	INF	No Filter	Trace4	2.95	4.8	Automated - Genesis	119651	Echinacea purpurea	Roots		
6		4.6690246	280.14148	4.643445	4.684373	93.0332336	1451.69658	1308.459735	0.0409282	INF	No Filter	Trace4	2.25	5.26	Automated - Genesis	119651	Echinacea purpurea	Roots		
7		4.827271	289.63626	4.815503	4.863161	0	1523.86728	1150.866637	0.047658	INF	No Filter	Trace4	2.36	4.62	Automated - Genesis	119651	Echinacea purpurea	Roots		
8		4.958472	297.50832	4.93479	4.972285	0	1092.54193	1036.73778	0.0374943	INF	No Filter	Trace4	1.69	4.17	Automated - Genesis	119651	Echinacea purpurea	Roots		
9		5.0368116	302.2087	5.025044	5.065973	0	1178.23627	1301.216258	0.0409296	INF	No Filter	Trace4	1.83	5.23	Automated - Genesis	119651	Echinacea purpurea	Roots		
10		5.1675611	310.05367	5.13409	5.197076	0	2094.6526	1245.892009	0.0629864	INF	No Filter	Trace4	3.25	5.01	Automated - Genesis	119651	Echinacea purpurea	Roots		
11		5.3668036	322.00822	5.352187	5.404968	0	2329.64443	1578.832292	0.0527801	INF	No Filter	Trace4	3.61	6.34	Automated - Genesis	119651	Echinacea purpurea	Roots		
1		0.920166	55.209958	0.909866	0.930464	174.076462	1746.11846	3333.514149	0.02059807	INF	No Filter	Trace6	65.04	64.97	Automated - Genesis	119651	Echinacea purpurea	Roots		
2		1.499738	89.98428	1.489474	1.510003	174.076218	938.360409	1797.435577	0.0205296	INF	No Filter	Trace6	34.96	35.03	Automated - Genesis	119651	Echinacea purpurea	Roots		
1		2.4609403	147.65642	2.446999	2.474881	223.061386	1213.57839	1711.318964	0.0278818	INF	No Filter	Trace9	3.59	7.21	Automated - Genesis	119651	Echinacea purpurea	Roots		
2		2.6531496	159.18898	2.639209	2.667091	223.06041	1041.40589	1468.52695	0.027882	INF	No Filter	Trace9	3.08	6.19	Automated - Genesis	119651	Echinacea purpurea	Roots		
3		2.7329894	163.97936	2.667091	2.765518	223.060135	23461.5548	8766.284385	0.0984275	INF	No Filter	Trace9	69.4	36.93	Automated - Genesis	119651	Echinacea purpurea	Roots	C11H12O5 Z-Sinapic acid	Level 4
4		2.7849534	167.0972	2.770166	2.798895	223.060791	2189.73213	3066.233235	0.028729	INF	No Filter	Trace9	6.48	12.92	Automated - Genesis	119651	Echinacea purpurea	Roots		
5		2.822129	169.32774	2.808188	2.83607	223.060486	1671.35771	2356.892918	0.0278814	INF	No Filter	Trace9	4.94	9.93	Automated - Genesis	119651	Echinacea purpurea	Roots		
6		2.8717669	172.30601	2.857826	2.885708	223.061371	1295.29271	1826.572684	0.0278816	INF	No Filter	Trace9	3.83	7.7	Automated - Genesis	119651	Echinacea purpurea	Roots		
7		2.9701951	178.21171	2.956254	2.984136	223.060196	1032.97918	1456.675194	0.0278818	INF	No Filter	Trace9	3.06	6.14	Automated - Genesis	119651	Echinacea purpurea	Roots		
8		6.142905	368.5743	6.132164	6.153645	223.06105	961.142407	1759.204978	0.0214813	INF	No Filter	Trace9	2.84	7.41	Automated - Genesis	119651	Echinacea purpurea	Roots	C11H12O5 E-Sinapic acid	Level 4
9		9.7295399	583.77239	9.715599	9.743481	223.061813	938.596098	1323.567384	0.0278818	INF	No Filter	Trace9	2.78	5.58	Automated - Genesis	119651	Echinacea purpurea	Roots		
1		0.7164518	42.987109	0.706135	0.726751	0	32767.0849	50546.83051	0.02061608	5.659005073	No Filter	Trace10	2.89	36.17	Automated - Genesis	119651	Echinacea purpurea	Roots		
2		1.5960762	95.764572	1.541167	2.751577	0	1101403.96	89208.68141	1.2104101	INF	No Filter	Trace10	97.11	63.83	Automated - Genesis	119651	Echinacea purpurea	Roots	C16H18O9 chlorogenic acid (5-o-caffeoylquinic acid); C16H18O9 cis 5-o-caffeoylquinic acid; C16H18O9 4-o-caffeoylquinic acid (crytochlorogenic acid)	Level 4
1		12.351059	741.06354	12.33712	12.36585	301.035034	988.1858	1347.194969	0.028728	INF	No Filter	Trace14	100	100	Automated - Genesis	119651	Echinacea purpurea	Roots		
1		0.9046995	54.281969	0.896741	0.913776	137.023819	1922.32024	4572.088084	0.01703543	INF	No Filter	Trace17	19.3	23.45	Automated - Genesis	119651	Echinacea purpurea	Roots		
2		1.3736885	82.42131	1.364664	1.382401	137.023834	487.595652	1166.69934	0.0177374	INF	No Filter	Trace17	4.89	5.98	Automated - Genesis	119651	Echinacea purpurea	Roots		
3		1.3926699	83.560194	1.383957	1.400594	137.023727	465.764789	1112.780317	0.0166368	INF	No Filter	Trace17	4.68	5.71	Automated - Genesis	119651	Echinacea purpurea	Roots		
4		3.8456739	230.74043	3.836978	3.853565	137.023788	301.372341	722.3587518	0.016587	INF	No Filter	Trace17	3.03	3.71	Automated - Genesis	119651	Echinacea purpurea	Roots		
5		10.259712	615.58272	10.2481	10.27133	137.023956	978.74487	1722.764185	0.023235	INF	No Filter	Trace17	9.83	8.84	Automated - Genesis	119651	Echinacea purpurea	Roots		
6		10.341883	620.51298	10.33027	10.3535	137.023834	1166.83782	2053.944104	0.023235	INF	No Filter	Trace17	11.71	10.54	Automated - Genesis	119651	Echinacea purpurea	Roots		
7		10.393846	623.63076	10.38223	10.40546	137.023758	1070.40904	1884.161792	0.023235	INF	No Filter	Trace17	10.75	9.67	Automated - Genesis	119651	Echinacea purpurea	Roots		
8		10.948102	656.88612	10.93564	10.95972	137.023849	1195.92917	2083.267142	0.02408	INF	No Filter	Trace17	12.01	10.69	Automated - Genesis	119651	Echinacea purpurea	Roots		
9		11.206213	672.37278	11.1946	11.21783	137.023773	1099.98469	1936.269963	0.023234	INF	No Filter	Trace17	11.04	9.93	Automated - Genesis	119651	Echinacea purpurea	Roots		
10		13.306897	798.41382	13.29528	13.31851	137.023788	1272.3545	2239.631435	0.023235	INF	No Filter	Trace17	12.77	11.49	Automated - Genesis	119651	Echinacea purpurea	Roots		
1		1.4211246	85.267476	1.414753	1.428598	153.018646	2008.73051	4858.857546	0.0138447	34.44654719	No Filter	Trace18	48.73	52.93	Automated - ICIS	119651	Echinacea purpurea	Roots		
2		1.6071053	96.426318	1.60121	1.615716	153.018616	2113.69598	4320.281827	0.0145066	30.62835048	No Filter	Trace18	51.27	47.07	Automated - ICIS	119651	Echinacea purpurea	Roots		
1		4.3288085	259.72851	4.279978	4.445364	593.152588	125454.693	27147.38437	0.1653865	INF	No Filter	Trace22	27.01	26.46	Automated - ICIS	119651	Echinacea purpurea	Roots	C27H30O15 Kaempferol diglycosides	Level 3
2		4.4847465	269.08479	4.459685	4.502648	0	5656.913	4823.241824	0.0429625	INF	No Filter	Trace22	1.22	4.7	Automated - ICIS	119651	Echinacea purpurea	Roots		
3		4.6513356	279.08014	4.586597	4.795039	593.1521	333443.901	70637.99481	0.2084429	INF	No Filter	Trace22	71.78	68.84	Automated - ICIS	119651	Echinacea purpurea	Roots	C27H30O15 Kaempferol diglycosides	Level 3
1		0.8068589	48.411534	0.796559	0.817162	216.050751	5125.0903	9783.118409	0.0206029	INF	No Filter	Trace25	5.38	20.23	Automated - Genesis	119651	Echinacea purpurea	Roots		
2		0.8738036	52.428216	0.832609	0.930464	216.050629	85309.0335	30148.80712	0.0978551	INF	No Filter	Trace25	89.49	62.34	Automated - Genesis	119651	Echinacea purpurea	Roots	i9G 2-hydroxyacryloyl-N-glutamate C8H11NO6	Level 4
3		0.9407624	56.445744	0.930464	0.952679	216.050827	3223.73933	5239.507778	0.02221463	INF	No Filter	Trace25	3.38	10.83	Automated - Genesis	119651	Echinacea purpurea	Roots		
4		0.9629769	57.778616	0.952679	0.973275	216.051422	1670.31633	3189.142712	0.02059673	INF	No Filter	Trace25	1.75	6.59	Automated - Genesis	119651	Echinacea purpurea	Roots		
1		5.6050909	336.30545	5.499666	6.502512	0	11507447.7	1136402.831	1.0028469	385.6181911	No Filter	Trace26	100	100	Automated - Genesis	119651	Echinacea purpurea	Roots	C10H10O4 Ferulic acid	Level 4
1		1.5360349	92.162094	1.504871	1.604314	0	15900.6509	6209.320005	0.0994433	INF	No Filter	Trace27	1.41	2.83	Automated - ICIS	119651	Echinacea purpurea	Roots	C17H22O10 Sinapoyl-glucose	Level 3
2		2.1031878	126.19127	2.04282	2.278733	0	280612.155	52570.21003	0.235913	INF	No Filter	Trace27	24.85	23.99	Automated - ICIS	119651	Echinacea purpurea	Roots	C17H22O10 Sinapoyl-glucose	Level 3
3		2.3016236	138.09742	2.281074	2.319813	0	4387.80217	2827.213009	0.038739	INF	No Filter	Trace27	0.39	1.29	Automated - ICIS	119651	Echinacea purpurea	Roots		
4		2.5120564	150.72338	2.345476	2.664767	385.11319	211535.503	25210.96918	0.319291	INF	No Filter	Trace27	18.73	11.5	Automated - ICIS	119651	Echinacea purpurea	Roots	C17H22O10 Sinapoyl-glucose	Level 3
5		2.7190489	163.14293	2.688002	2.77566	385.115143	7761.70076	3700.779945	0.087658	INF	No Filter	Trace27	0.69	1.69	Automated - ICIS	119651	Echinacea purpurea	Roots	C17H22O10 Sinapoyl-glucose	Level 3
6		2.9391478	176.34887	2.890355	3.050039	385.113861	69998.2043	16770.01328	0.1596834	INF	No Filter	Trace27	6.2	7.65	Automated - ICIS	119651	Echinacea purpurea	Roots	C17H22O10 Sinapoyl-glucose	Level 3
7		3.1415014	188.49008	3.080244	3.218338	385.114014	47859.4082	16565.68434	0.1380942	INF	No Filter	Trace27	4.24	7.56	Automated - ICIS	119651	Echinacea purpurea	Roots		

8	3.2529005	195.17403	3.239819	3.298703	0	6225.23406	5068.053682	0.0588841	INF	No Filter	Trace27	0.55	2.31	Automated - ICIS	119651	Echinacea purpurea	Roots	$C_{17}H_{22}O_{10}$ Sinapoyl-glucose	Level 3
9	3.6524057	219.14434	3.593517	3.809861	385.114532	280583.808	53798.20655	0.2163438	INF	No Filter	Trace27	24.85	24.55	Automated - ICIS	119651	Echinacea purpurea	Roots	$C_{17}H_{22}O_{10}$ Sinapoyl-glucose	Level 3
10	3.8933267	233.5996	3.811396	4.052804	0	195620.962	33399.72116	0.2414077	INF	No Filter	Trace27	17.32	15.24	Automated - ICIS	119651	Echinacea purpurea	Roots	$C_{17}H_{22}O_{10}$ Sinapoyl-glucose	Level 3
11	4.1248078	247.48847	4.063036	4.14374	0	8708.13783	3044.714743	0.0807041	INF	No Filter	Trace27	0.77	1.39	Automated - ICIS	119651	Echinacea purpurea	Roots		
1	1.2883917	77.303502	1.233108	1.74916	0	1612650.63	289571.0849	0.5160523	INF	No Filter	Trace28	100	100	Automated - Genesis	119651	Echinacea purpurea	Roots	$C_{15}H_{16}O_{9}$ z sinapoyl-malate	Level 4
1	0.7354807	44.12884	0.725182	0.757646	0	48612.8572	60588.62695	0.03246402	INF	No Filter	Trace29	41.69	47.74	Automated - Genesis	119651	Echinacea purpurea	Roots		
2	0.7811089	46.866535	0.771529	0.805619	335.076935	11521.802	17508.90715	0.03408964	INF	No Filter	Trace29	9.88	13.8	Automated - Genesis	119651	Echinacea purpurea	Roots		
3	0.8416678	50.500068	0.809199	0.851966	0	16809.0532	14708.09145	0.04276672	INF	No Filter	Trace29	14.42	11.59	Automated - Genesis	119651	Echinacea purpurea	Roots		
4	0.8995494	53.972966	0.891591	0.918926	335.078003	12745.1611	23971.36135	0.02733483	INF	No Filter	Trace29	10.93	18.89	Automated - Genesis	119651	Echinacea purpurea	Roots		
5	2.6299147	157.79488	2.577958	2.714402	335.077515	26903.8906	10126.40488	0.1364437	INF	No Filter	Trace29	23.08	7.98	Automated - Genesis	119651	Echinacea purpurea	Roots	$C_{16}H_{16}O_{8}$ caffeoyl-shikimate	Level 4
1	1.3262018	79.572108	1.315936	1.336466	137.023895	768.531415	1472.825851	0.0205309	INF	No Filter	Trace1	20.84	22.68	Automated - Genesis	119652	Echinacea purpurea	Stems		
2	1.3576408	81.458448	1.346731	1.367909	137.023804	448.196393	802.6698381	0.0211779	INF	No Filter	Trace1	12.15	12.36	Automated - Genesis	119652	Echinacea purpurea	Stems		
3	1.4192513	85.155078	1.408969	1.429516	137.024231	451.700633	864.7239845	0.020547	INF	No Filter	Trace1	12.25	13.32	Automated - Genesis	119652	Echinacea purpurea	Stems		
4	1.5132726	90.796356	1.503004	1.525164	137.023972	496.39229	946.6017864	0.0221599	INF	No Filter	Trace1	13.46	14.58	Automated - Genesis	119652	Echinacea purpurea	Stems		
5	5.1761246	310.56748	5.165892	5.186357	137.024017	269.104266	517.3685795	0.0204652	INF	No Filter	Trace1	7.3	7.97	Automated - Genesis	119652	Echinacea purpurea	Stems		
6	5.9846052	359.07631	5.974372	5.994836	119.01445	236.688837	454.3591433	0.020464	INF	No Filter	Trace1	6.42	7	Automated - Genesis	119652	Echinacea purpurea	Stems		
7	11.749749	704.98494	11.73581	11.76369	137.023788	1016.99251	1434.100137	0.027881	INF	No Filter	Trace1	27.58	22.09	Automated - Genesis	119652	Echinacea purpurea	Stems		
1	1.2492092	74.952552	1.175573	1.325191	137.023071	70450.8687	20951.55939	0.149618321	9.769946648	No Filter	Trace3	5.88	17.19	Manual	119652	Echinacea purpurea	Stems		
2	1.8091106	108.54664	1.742113	2.297879	137.023209	1128207.62	100938.1395	0.555766195	46.64921696	No Filter	Trace3	94.12	82.81	Manual	119652	Echinacea purpurea	Stems		
1	0.9150262	54.901571	0.891596	0.960187	0	9316.93486	5944.427608	0.06859125	INF	No Filter	Trace4	16.12	21.02	Automated - Genesis	119652	Echinacea purpurea	Stems		
2	1.1075642	66.453852	1.097282	1.117389	93.0330124	392.660207	766.4459528	0.0201068	INF	No Filter	Trace4	0.68	2.71	Automated - Genesis	119652	Echinacea purpurea	Stems		
3	1.5318481	91.910886	1.519958	1.542116	93.0331421	386.51672	726.1193704	0.0221584	INF	No Filter	Trace4	0.67	2.57	Automated - Genesis	119652	Echinacea purpurea	Stems		
4	3.875449	232.52694	3.865202	3.885681	93.0332947	379.284767	728.4889729	0.0204793	INF	No Filter	Trace4	0.66	2.58	Automated - Genesis	119652	Echinacea purpurea	Stems		
5	3.8959148	233.75489	3.885681	3.906147	93.0333252	397.365006	763.8725641	0.0204651	INF	No Filter	Trace4	0.69	2.7	Automated - Genesis	119652	Echinacea purpurea	Stems		
6	3.9667959	238.00775	3.91638	3.97937	0	1642.72801	923.9097569	0.0629901	INF	No Filter	Trace4	2.84	3.27	Automated - Genesis	119652	Echinacea purpurea	Stems		
7	4.038793	242.32758	4.027025	4.052606	0	640.117425	936.1950776	0.0255809	INF	No Filter	Trace4	1.11	3.31	Automated - Genesis	119652	Echinacea purpurea	Stems		
8	4.0833018	244.99811	4.062837	4.093535	93.0330429	645.213795	706.6458854	0.0306979	INF	No Filter	Trace4	1.12	2.5	Automated - Genesis	119652	Echinacea purpurea	Stems		
9	4.1037669	246.22601	4.093535	4.115594	93.0332794	345.291295	609.9878244	0.0220591	INF	No Filter	Trace4	0.6	2.16	Automated - Genesis	119652	Echinacea purpurea	Stems		
10	4.1514095	249.08457	4.130942	4.161641	93.0332718	751.858298	791.2699055	0.0306989	INF	No Filter	Trace4	1.3	2.8	Automated - Genesis	119652	Echinacea purpurea	Stems		
11	4.2451188	254.70713	4.234885	4.255351	93.0332947	378.95666	728.4248518	0.020466	INF	No Filter	Trace4	0.66	2.58	Automated - Genesis	119652	Echinacea purpurea	Stems		
12	4.2774206	256.64524	4.265583	4.287651	93.033226	435.534426	712.8388549	0.022068	INF	No Filter	Trace4	0.75	2.52	Automated - Genesis	119652	Echinacea purpurea	Stems		
13	4.3030164	258.18098	4.287651	4.318365	93.0332947	1092.75543	1300.963416	0.0307139	INF	No Filter	Trace4	1.89	4.6	Automated - Genesis	119652	Echinacea purpurea	Stems		
14	4.3636784	261.8207	4.318365	4.401832	0	2580.97194	1133.568189	0.0834667	INF	No Filter	Trace4	4.47	4.01	Automated - Genesis	119652	Echinacea purpurea	Stems		
15	4.4977555	269.86533	4.405412	4.510837	0	3064.7917	1130.712949	0.105425	INF	No Filter	Trace4	5.3	4	Automated - Genesis	119652	Echinacea purpurea	Stems		
16	4.5304348	271.82609	4.514417	4.613883	93.0330811	4883.6139	1667.629318	0.0994657	INF	No Filter	Trace4	8.45	5.9	Automated - Genesis	119652	Echinacea purpurea	Stems		
17	4.6410181	278.46109	4.624118	4.659947	0	1389.80696	1143.511229	0.0358293	INF	No Filter	Trace4	2.4	4.04	Automated - Genesis	119652	Echinacea purpurea	Stems		
18	4.7823775	286.94265	4.665063	4.85919	0	8490.93969	1446.473977	0.1941268	INF	No Filter	Trace4	14.69	5.11	Automated - Genesis	119652	Echinacea purpurea	Stems		
19	4.8875468	293.25281	4.874538	4.90012	0	521.015654	759.6127639	0.0255818	INF	No Filter	Trace4	0.9	2.69	Automated - Genesis	119652	Echinacea purpurea	Stems		
20	4.9556711	297.34027	4.915475	4.968245	0	1582.18789	1014.172787	0.0527697	INF	No Filter	Trace4	2.74	3.59	Automated - Genesis	119652	Echinacea purpurea	Stems		
21	4.9953612	299.72167	4.983593	5.102892	0	4711.51882	1484.651134	0.1192987	INF	No Filter	Trace4	8.15	5.25	Automated - Genesis	119652	Echinacea purpurea	Stems		
22	5.2048624	312.29174	5.109612	5.228907	0	4997.36813	1255.555251	0.1192943	INF	No Filter	Trace4	8.65	4.44	Automated - Genesis	119652	Echinacea purpurea	Stems		
23	5.3343957	320.06374	5.228907	5.40609	0	8762.82499	1604.557254	0.1771834	INF	No Filter	Trace4	15.16	5.67	Automated - Genesis	119652	Echinacea purpurea	Stems		
1	2.1687223	130.12334	2.158458	2.178987	225.040237	677.592998	1298.021445	0.0205289	INF	No Filter	Trace5	23.25	29.53	Automated - ICIS	119652	Echinacea purpurea	Stems		
2	2.7790069	166.74041	2.764216	2.792947	225.03978	1193.52699	1626.898682	0.0287318	INF	No Filter	Trace5	40.95	37.01	Automated - ICIS	119652	Echinacea purpurea	Stems		
3	13.749873	824.99238	13.73593	13.76381	225.039642	1043.35972	1471.316988	0.027882	INF	No Filter	Trace5	35.8	33.47	Automated - ICIS	119652	Echinacea purpurea	Stems		
1	0.6612172	39.673034	0.650477	0.671958	174.076797	1417.1198	2593.767933	0.0214813	INF	No Filter	Trace6	100	100	Automated - Genesis	119652	Echinacea purpurea	Stems	$C_{15}H_{17}NO_{9}$ Isochorismate-9-glutamate	Level 2
1	0.6254152	37.524909	0.614674	0.636156	223.061127	1468.32198	2687.489044	0.02148124	INF	No Filter	Trace9	16.4	29.08	Automated - Genesis	119652	Echinacea purpurea	Stems		
2	2.7130989	162.78593	2.699158	2.72704	223.060699	1185.59867	1671.859889	0.027882	INF	No Filter	Trace9	13.25	18.09	Automated - Genesis	119652	Echinacea purpurea	Stems	$C_{11}H_{12}O_{5}$ Z-Sinapic acid	Level 4
3	6.1189725	367.13835	6.108232	6.169095	223.060806	5364.52496	3177.41699	0.0608637	INF	No Filter	Trace9	59.94	34.38	Automated - Genesis	119652	Echinacea purpurea	Stems	$C_{11}H_{12}O_{5}$ E-Sinapic acid	Level 4
4	6.253043	375.18258	6.242302	6.263783	223.060684	932.108849	1706.043368	0.021481	INF	No Filter	Trace9	10.41	18.46	Automated - Genesis	119652	Echinacea purpurea	Stems		
1	1.1588371	69.530226	1.149328	1.169431	353.087585	1047.06395	2046.391667	0.0201024	INF	No Filter	Trace10	1.59	6.67	Automated - Genesis	119652	Echinacea purpurea	Stems	$C_{16}H_{18}O_{9}$ neochlorogenic acid (3-o-caffeoylquinic acid)	Level 4
2	1.5985736	95.914416	1.569019	1.770198	0	49516.2415	9190.259956	0.2011786	INF	No Filter	Trace10	75.35	29.96	Automated - Genesis	119652	Echinacea purpurea	Stems	$C_{16}H_{18}O_{9}$ chlorogenic acid (5-o-caffeoylquinic acid); $C_{16}H_{18}O_{9}$ cis 5-o-caffeoylquinic acid	Level 4
3	1.829193	109.75158	1.806318	1.83977	353.086945	2098.42288	2601.952593	0.0334521	INF	No Filter	Trace10	3.19	8.48	Automated - Genesis	119652	Echinacea purpurea	Stems		
4	1.864318	111.85908	1.853139	1.874896	353.086517	1258.62	2351.130846	0.0217562	INF	No Filter	Trace10	1.92	7.66	Automated - Genesis	119652	Echinacea purpurea	Stems		
5	1.9111108	114.66665	1.901633	1.950053	353.087891	4320.27168	2610.265304	0.0484202	INF	No Filter	Trace10	6.57	8.51	Automated - Genesis	119652	Echinacea purpurea	Stems		
6	2.158458	129.50748	2.148191	2.168722	353.087463	907.509544	1738.114306	0.0205309	INF	No Filter	Trace10	1.38	5.67	Automated - Genesis	119652	Echinacea purpurea	Stems		
7	2.2165414	132.99248	2.206277	2.226807	353.088135	833.426174	1596.490479	0.0205294	INF	No Filter	Trace10	1.27	5.2	Automated - Genesis	119652	Echinacea purpurea	Stems		
8	2.3105544	138.63326	2.300289	2.32082	353.088776	959.156707	1837.328909	0.0205303	INF	No Filter	Trace10	1.46	5.99	Automated - Genesis	119652	Echinacea purpurea	Stems		
9	2.5975503	155.85302	2.583609	2.612338	353.087677	1145.21832	1587.201456	0.0287288	INF	No Filter	Trace10	1.74	5.17	Automated - Genesis	119652	Echinacea purpurea	Stems		
10	2.9122944	174.73766	2.898354	2.926235	353.08902	1233.71188	1739.72367	0.0278816	INF	No Filter	Trace10	1.88	5.67	Automated - Genesis	119652	Echinacea purpurea	Stems		
11	3.121627	187.29762	3.107686	3.135568	353.089355	1160.15973	1635.985838	0.0278817	INF	No Filter	Trace10	1.77	5.33	Automated - Genesis	119652	Echinacea purpurea	Stems		
12	9.5668309	574.00985	9.55289	9.580772	353.087585	1236.89723	1744.196599	0.0278818	INF	No Filter	Trace10	1.88	5.69	Automated - Genesis	119652	Echinacea purpurea	Stems		
1	0.6241757	37.45054	0.602294	0.642077	0	71071.3534	65185.33766	0.03978277	INF	No Filter	Trace13	100	100	Automated - Genesis	119652	Echinacea purpurea	Stems	$C_{22}H_{18}O_{12}$ cichoric acid	Level 4
1	1.3262018	79.572108	1.31749	1.334126	137.023895	769.493536	1838.480452	0.016636	INF	No Filter	Trace17	22.25	24.39	Automated - Genesis	119652	Echinacea purpurea	Stems		
2	1.3576408	81.458448	1.348283	1.365569	137.023804	454.85867	1001.946569	0.0172855	INF	No Filter	Trace17	13.15	13.29	Automated - Genesis	119652	Echinacea purpurea	Stems		
3	1.4192513	85.155078	1.410522	1.427176	137.024231	452.217516	1079.406735	0.0166542	INF	No Filter	Trace17	13.08	14.32	Automated - Genesis	119652	Echinacea purpurea	Stems		
4	1.5132726	90.796356	1.504556	1.521197	137.023972	494.693981	1181.612123	0.0166411	INF	No Filter	Trace17	14.31	15.68	Automated - Genesis	119652	Echinacea purpurea	Stems		
5	5.1761246	310.56748	5.167429	5.184016	137.024017	269.436223	645.8143165	0.0165873	INF	No Filter	Trace17	7.79	8.57	Automated - Genesis	119652	Echinacea purpurea	Stems		
6	11.749749	704.98494	11.73813	11.76137	137.023788	1016.99466	1790.140408	0.023235	INF	No Filter	Trace17	29.41	23.75	Automated - Genesis	119652	Echinacea purpurea	Stems		
1	0.9526974	57.161845	0.940772	0.962995	216.982193	5509.58661	10469.41351	0.02222283	INF	No Filter	Trace20	84.84	88.29	Automated - Genesis	119652	Echinacea purpurea	Stems		
2	11.22419	673.4514	11.21025	11.23813	216.981049	984.627299	1388.452412	0.027882	INF	No Filter	Trace20	15.16	11.71	Automated - Genesis	119652	Echinacea purpurea	Stems		

1	6.5228794	391.37276	6.490658	6.62277	431.097992	70593.0448	20699.55801	0.1321123	INF		No Filter	Trace21	100	100	Automated - Genesis	119652	Echinacea purpurea	Stems		
1	4.336496	260.18976	4.265583	4.490595	593.152039	2938119.7	599101.1494	0.2250116	INF		No Filter	Trace22	71.71	68.22	Automated - ICIS	119652	Echinacea purpurea	Stems	C27H30O15 Kaempferol diglycosides	Level 3
2	4.5049159	270.29495	4.492936	4.538835	593.15155	6543.22249	4936.42909	0.0458995	INF		No Filter	Trace22	0.16	0.56	Automated - ICIS	119652	Echinacea purpurea	Stems		
3	4.6422576	278.53546	4.580558	4.780841	593.151794	1035922.14	245865.1241	0.2002834	INF		No Filter	Trace22	25.28	28	Automated - ICIS	119652	Echinacea purpurea	Stems	C27H30O15 Kaempferol diglycosides	Level 3
4	4.85919	291.5514	4.780841	4.882431	0	23132.7958	9499.333129	0.1015899	INF		No Filter	Trace22	0.56	1.08	Automated - ICIS	119652	Echinacea purpurea	Stems		
5	4.9131344	294.78806	4.882431	5.09032	593.151001	74394.0013	10845.10859	0.2078886	INF		No Filter	Trace22	1.82	1.23	Automated - ICIS	119652	Echinacea purpurea	Stems	C27H30O15 Kaempferol diglycosides	Level 3
6	5.1430869	308.58521	5.09032	5.19821	593.150757	15208.6	4323.479532	0.1078903	INF		No Filter	Trace22	0.37	0.49	Automated - ICIS	119652	Echinacea purpurea	Stems		
7	5.2265661	313.59397	5.19821	5.23556	593.151062	3825.57899	3675.781215	0.0373498	INF		No Filter	Trace22	0.09	0.42	Automated - ICIS	119652	Echinacea purpurea	Stems		
1	4.6241181	277.44709	4.574637	4.700143	0	33243.9873	8801.826153	0.125506	INF		No Filter	Trace24	18.07	16.02	Automated - Genesis	119652	Echinacea purpurea	Stems		
2	4.9272457	295.63474	4.861966	5.001717	0	27975.7851	7251.037035	0.1397506	INF		No Filter	Trace24	15.21	13.2	Automated - Genesis	119652	Echinacea purpurea	Stems	C21H20O11 kaempferol-O-glucoside	Level 3
3	5.5596634	333.5798	5.532262	5.630524	0	43117.6417	13513.18563	0.0982623	INF		No Filter	Trace24	23.44	24.6	Automated - Genesis	119652	Echinacea purpurea	Stems		
4	5.6615058	339.69035	5.634104	5.692986	0	7124.10136	4381.874451	0.0588818	INF		No Filter	Trace24	3.87	7.98	Automated - Genesis	119652	Echinacea purpurea	Stems		
5	5.8239916	349.4395	5.81376	5.834224	447.094238	986.446641	1895.767651	0.0204646	INF		No Filter	Trace24	0.54	3.45	Automated - Genesis	119652	Echinacea purpurea	Stems		
6	5.8995965	353.97579	5.844456	6.042177	0	68623.065	13846.46	0.1977214	INF		No Filter	Trace24	37.31	25.21	Automated - Genesis	119652	Echinacea purpurea	Stems		
7	6.0636579	363.81947	6.052918	6.074398	447.093536	1006.50044	1842.249151	0.0214807	INF		No Filter	Trace24	0.55	3.35	Automated - Genesis	119652	Echinacea purpurea	Stems		
8	6.0851391	365.10835	6.074398	6.09588	447.095398	911.075013	1667.550718	0.0214818	INF		No Filter	Trace24	0.5	3.04	Automated - Genesis	119652	Echinacea purpurea	Stems		
9	6.1476141	368.85685	6.136873	6.158355	447.09375	947.033251	1733.380668	0.0214813	INF		No Filter	Trace24	0.51	3.16	Automated - Genesis	119652	Echinacea purpurea	Stems		
1	5.584725	335.0835	5.497202	6.599674	0	61775499.2	5858957.346	1.1024719		846.9683314	No Filter	Trace26	100	100	Automated - Genesis	119652	Echinacea purpurea	Stems	C10H10O4 Ferulic acid	Level 4
1	2.0952442	125.71465	2.033638	2.221674	385.11322	75540.722	15921.22879	0.1880355	INF		No Filter	Trace27	10.71	10.55	Automated - ICIS	119652	Echinacea purpurea	Stems	C17H22O10 Sinapoyl-glucose	Level 3
2	2.412303	144.73818	2.310554	2.492152	385.113495	102280.948	17937.23146	0.1815971	INF		No Filter	Trace27	14.5	11.89	Automated - ICIS	119652	Echinacea purpurea	Stems	C17H22O10 Sinapoyl-glucose	Level 3
3	2.5060922	150.36553	2.492152	2.595227	385.114227	36961.8318	15224.30567	0.103075	INF		No Filter	Trace27	5.24	10.09	Automated - ICIS	119652	Echinacea purpurea	Stems	C17H22O10 Sinapoyl-glucose	Level 3
4	2.9642618	177.85571	2.905324	3.015378	385.11377	33206.8443	9910.300307	0.1100542	INF		No Filter	Trace27	4.71	6.57	Automated - ICIS	119652	Echinacea purpurea	Stems	C17H22O10 Sinapoyl-glucose	Level 3
5	3.1332444	187.99466	3.058043	3.175067	385.113586	28174.4051	8993.04114	0.1170235	INF		No Filter	Trace27	3.99	5.96	Automated - ICIS	119652	Echinacea purpurea	Stems		
6	3.2610953	195.66572	3.208632	3.339132	0	29617.8012	8270.270735	0.1304999	INF		No Filter	Trace27	4.2	5.48	Automated - ICIS	119652	Echinacea purpurea	Stems	C17H22O10 Sinapoyl-glucose	Level 3
7	3.6593622	219.56173	3.587398	3.799856	0	252280.594	41742.8844	0.2124578	INF		No Filter	Trace27	35.77	27.66	Automated - ICIS	119652	Echinacea purpurea	Stems	C17H22O10 Sinapoyl-glucose	Level 3
8	3.8718684	232.3121	3.802196	4.034916	0	134479.732	22914.19985	0.23272	INF		No Filter	Trace27	19.06	15.18	Automated - ICIS	119652	Echinacea purpurea	Stems	C17H22O10 Sinapoyl-glucose	Level 3
9	4.0592569	243.55541	4.034916	4.080961	0	4105.31297	3109.186351	0.0460449	INF		No Filter	Trace27	0.58	2.06	Automated - ICIS	119652	Echinacea purpurea	Stems	C17H22O10 Sinapoyl-glucose	Level 3
10	4.1065418	246.39251	4.086078	4.128601	385.113861	2992.01895	1850.200365	0.042523	INF		No Filter	Trace27	0.42	1.23	Automated - ICIS	119652	Echinacea purpurea	Stems		
11	4.2144209	252.86525	4.190018	4.227428	0	2966.16956	3027.448765	0.0374104	INF		No Filter	Trace27	0.42	2.01	Automated - ICIS	119652	Echinacea purpurea	Stems		
12	5.486461	329.18766	5.469799	5.512762	0	2771.36673	2002.547458	0.0429625	INF		No Filter	Trace27	0.39	1.33	Automated - ICIS	119652	Echinacea purpurea	Stems		
1	1.2866933	77.201598	1.226335	1.848007	0	1924299.06	327600.2102	0.6216725	INF		No Filter	Trace28	100	100	Automated - Genesis	119652	Echinacea purpurea	Stems	C15H16O9 z sinapoyl-malate	Level 4
1	0.7279787	43.67872	0.720021	0.737038	335.078674	23082.4783	54990.67335	0.01701721	INF		No Filter	Trace29	34.46	40.38	Automated - Genesis	119652	Echinacea purpurea	Stems		
2	0.7691786	46.150718	0.761219	0.779878	335.078217	6472.70633	15005.18338	0.01865833	INF		No Filter	Trace29	9.66	11.02	Automated - Genesis	119652	Echinacea purpurea	Stems		
3	0.8004744	48.028462	0.788607	0.821076	0	11757.4957	12003.07173	0.03246892	INF		No Filter	Trace29	17.55	8.81	Automated - Genesis	119652	Echinacea purpurea	Stems		
4	0.8635099	51.810593	0.855552	0.872568	335.078857	17008.1883	40520.73163	0.01701665	INF		No Filter	Trace29	25.39	29.75	Automated - Genesis	119652	Echinacea purpurea	Stems		
5	0.889255	53.355298	0.881298	0.908637	335.078796	8664.70207	13679.40688	0.0273387	INF		No Filter	Trace29	12.94	10.04	Automated - Genesis	119652	Echinacea purpurea	Stems		
1	9.7637096	585.82258	9.749769	9.778495	510.078461	1557.48035	2180.942203	0.0287259	INF		No Filter	Trace30	53.33	53.15	Automated - Genesis	119652	Echinacea purpurea	Stems		
2	13.672347	820.34082	13.65841	13.68629	510.078247	1363.1453	1922.291533	0.027881	INF		No Filter	Trace30	46.67	46.85	Automated - Genesis	119652	Echinacea purpurea	Stems		
1	0.8480796	50.884773	0.837782	0.858378	137.023788	1228.11585	2344.838527	0.0205962	INF		No Filter	Trace1	9.75	12	Automated - Genesis	119653	Echinacea purpurea	Leaves		
2	2.3715786	142.29472	2.361314	2.381846	137.024353	282.791444	541.1436044	0.0205315	INF		No Filter	Trace1	2.24	2.77	Automated - Genesis	119653	Echinacea purpurea	Leaves		
3	3.1485445	188.91267	3.134604	3.162485	137.023758	1280.36373	1805.505049	0.0278815	INF		No Filter	Trace1	10.16	9.24	Automated - Genesis	119653	Echinacea purpurea	Leaves		
4	3.6082668	216.49601	3.595925	3.619007	119.013321	298.529797	468.6493747	0.0230823	INF		No Filter	Trace1	2.37	2.4	Automated - Genesis	119653	Echinacea purpurea	Leaves		
5	4.0206618	241.23971	4.01043	4.032502	137.023956	948.585296	1551.51449	0.0220715	INF		No Filter	Trace1	7.53	7.94	Automated - Genesis	119653	Echinacea purpurea	Leaves		
6	4.0785468	244.71281	4.052966	4.088779	137.023727	1681.60847	1894.880705	0.0358127	INF		No Filter	Trace1	13.35	9.7	Automated - Genesis	119653	Echinacea purpurea	Leaves		
7	4.4920391	269.52235	4.481298	4.50278	93.0345001	262.952745	481.286903	0.0214812	INF		No Filter	Trace1	2.09	2.46	Automated - Genesis	119653	Echinacea purpurea	Leaves		
8	5.2125322	312.75193	5.2023	5.222765	137.024048	280.441855	539.1024787	0.0204642	INF		No Filter	Trace1	2.23	2.76	Automated - Genesis	119653	Echinacea purpurea	Leaves		
9	6.2859153	377.15492	6.275175	6.296656	137.023743	1904.68574	3486.1743	0.0214813	INF		No Filter	Trace1	15.12	17.84	Automated - Genesis	119653	Echinacea purpurea	Leaves		
10	6.8540404	411.24242	6.8433	6.866379	137.023987	917.359718	1440.591059	0.0230788	INF		No Filter	Trace1	7.28	7.37	Automated - Genesis	119653	Echinacea purpurea	Leaves		
11	7.2393556	434.36134	7.22912	7.249587	136.016708	267.262482	512.8519746	0.0204676	INF		No Filter	Trace1	2.12	2.62	Automated - Genesis	119653	Echinacea purpurea	Leaves		
12	10.574631	634.47786	10.56069	10.58857	137.023743	1209.12597	1705.055826	0.027881	INF		No Filter	Trace1	9.6	8.72	Automated - Genesis	119653	Echinacea purpurea	Leaves		
13	10.774672	646.48032	10.75989	10.78861	137.023788	1153.64202	1523.873766	0.028728	INF		No Filter	Trace1	9.16	7.8	Automated - Genesis	119653	Echinacea purpurea	Leaves		
14	11.828089	709.68534	11.81415	11.84203	137.023895	884.284165	1246.960378	0.027882	INF		No Filter	Trace1	7.02	6.38	Automated - Genesis	119653	Echinacea purpurea	Leaves		
1	0.6241768	37.450609	0.609856	0.691502	0	63908.8545	25130.36008	0.08164621		8.493737528	No Filter	Trace3	44.74	63.97	Automated - ICIS	119653	Echinacea purpurea	Leaves		
2	1.8082935	108.49761	1.706774	1.962279	137.023163	78941.1189	14154.24382	0.255505091		6.57603584	No Filter	Trace3	55.26	36.03	Manual	119653	Echinacea purpurea	Leaves		
1	3.8280377	229.68226	3.81503	3.840611	0	602.200121	874.5453338	0.0255812	INF		No Filter	Trace4	2.08	3.3	Automated - Genesis	119653	Echinacea purpurea	Leaves		
2	3.8626715	231.76029	3.840611	3.872903	93.0331879	730.586094	667.2617301	0.0322921	INF		No Filter	Trace4	2.53	2.52	Automated - Genesis	119653	Echinacea purpurea	Leaves		
3	3.9012769	234.07661	3.888255	3.913849	0	652.969259	953.8771122	0.0255949	INF		No Filter	Trace4	2.26	3.6	Automated - Genesis	119653	Echinacea purpurea	Leaves		
4	3.9343242	236.05945	3.924091	3.946241	93.0329285	375.745678	660.7797834	0.0221498	INF		No Filter	Trace4	1.3	2.5	Automated - Genesis	119653	Echinacea purpurea	Leaves		
5	4.0127706	240.76624	4.002539	4.024611	93.0329437	300.277528	574.6114325	0.0220716	INF		No Filter	Trace4	1.04	2.17	Automated - Genesis	119653	Echinacea purpurea	Leaves		
6	4.128537	247.71222	4.118304	4.154117	93.0333633	612.636113	735.9214579	0.035813	INF		No Filter	Trace4	2.12	2.78	Automated - Genesis	119653	Echinacea purpurea	Leaves		
7	4.1643504	249.86102	4.154117	4.184815	93.0331879	714.913664	726.8629068	0.0306976	INF		No Filter	Trace4	2.47	2.75	Automated - Genesis	119653	Echinacea purpurea	Leaves		
8	4.1966504	251.79902	4.184815	4.206884	93.0333405	374.048448	688.1120438	0.0220686	INF		No Filter	Trace4	1.29	2.6	Automated - Genesis	119653	Echinacea purpurea	Leaves		
9	4.2647002	255.88201	4.237582	4.28012	0	970.220601	1046.674073	0.0425376	INF		No Filter	Trace4	3.36	3.95	Automated - Genesis	119653	Echinacea purpurea	Leaves		
10	4.3123514	258.74108	4.285236	4.326164	0	1086.63555	1216.539934	0.0409279	INF		No Filter	Trace4	3.76	4.6	Automated - Genesis	119653	Echinacea purpurea	Leaves		
11	4.4080937	264.48562	4.378931	4.429575	93.0332184	1369.32552	917.7598859	0.050644	INF		No Filter	Trace4	4.74	3.47	Automated - Genesis	119653	Echinacea purpurea	Leaves		
12	4.5271094	271.62656	4.50278	4.590314	0	3889.3541	1371.825448	0.0875343	INF		No Filter	Trace4	13.46	5.18	Automated - Genesis	119653	Echinacea purpurea	Leaves		
13	4.6565899	279.39539	4.595133	4.669162	0	3142.09789	1353.84066	0.0740287	INF		No Filter	Trace4	10.87	5.11	Automated - Genesis	119653	Echinacea purpurea	Leaves		
14	4.7298303	283.78982	4.701474	4.742404	0	1059.39521	1189.926775	0.0409299	INF		No Filter	Trace4	3.67	4.49	Automated - Genesis	119653	Echinacea purpurea	Leaves		
15	4.7679833	286.079	4.757752	4.795167	93.0330887	635.337239	592.7142801	0.0374156	INF		No Filter	Trace4	2.2	2.24	Automated - Genesis	119653	Echinacea purpurea	Leaves		
16	4.8258649	289.55189	4.810517	4.846338	93.0330429	1197.43353	1046.074842	0.0358209	INF		No Filter	Trace4	4.14	3.95	Automated - Genesis	119653	Echinacea purpurea	Leaves		
17	4.8904202	293.42521	4.846338	4.904233	0	2163.44631	1392.834346	0.0578954	INF		No Filter	Trace4	7.48	5.26	Automated - Genesis	119653	Echinacea purpurea	Leaves		
18	4.9569975	297.41985	4.946766	4.977484	93.0331192	774.249804	883.0477525	0.0307178	INF		No Filter	Trace4	2.68	3.34	Automated - Genesis	119653	Echinacea purpurea	Leaves		

19	4.9928327	299.56996	4.982601	5.018415	93.0331802	555.333525	549.1910476	0.0358137	INF	No Filter	Trace4	1.92	2.07	Automated - Genesis	119653	Echinacea purpurea	Leaves		
20	5.0317813	301.90688	5.018415	5.045595	0	723.254263	1039.056419	0.0271807	INF	No Filter	Trace4	2.5	3.92	Automated - Genesis	119653	Echinacea purpurea	Leaves		
21	5.0586033	303.5162	5.045595	5.071175	0	673.428855	987.4224092	0.0255801	INF	No Filter	Trace4	2.33	3.73	Automated - Genesis	119653	Echinacea purpurea	Leaves		
22	5.081408	304.88448	5.071175	5.101872	93.0332108	572.548323	571.0926889	0.030697	INF	No Filter	Trace4	1.98	2.16	Automated - Genesis	119653	Echinacea purpurea	Leaves		
23	5.1290697	307.74418	5.10859	5.154651	93.0334091	1198.85923	1129.997465	0.0460604	INF	No Filter	Trace4	4.15	4.27	Automated - Genesis	119653	Echinacea purpurea	Leaves		
24	5.1699983	310.1999	5.159766	5.192067	93.0329514	649.31993	697.8171337	0.0323004	INF	No Filter	Trace4	2.25	2.64	Automated - Genesis	119653	Echinacea purpurea	Leaves		
25	5.2227646	313.36588	5.197182	5.232996	93.0333099	588.403332	605.4850421	0.0358142	INF	No Filter	Trace4	2.04	2.29	Automated - Genesis	119653	Echinacea purpurea	Leaves		
26	5.2432281	314.59369	5.232996	5.275537	93.0331802	968.822891	664.6566648	0.0425406	INF	No Filter	Trace4	3.35	2.51	Automated - Genesis	119653	Echinacea purpurea	Leaves		
27	5.2908851	317.45311	5.280654	5.301118	93.0332336	396.701529	762.5995003	0.0204641	INF	No Filter	Trace4	1.37	2.88	Automated - Genesis	119653	Echinacea purpurea	Leaves		
28	5.3141255	318.84753	5.301118	5.326698	0	866.066536	1276.442535	0.0255799	INF	No Filter	Trace4	3	4.82	Automated - Genesis	119653	Echinacea purpurea	Leaves		
29	5.3397058	320.38235	5.326698	5.352279	0	525.71203	771.1474862	0.0255816	INF	No Filter	Trace4	1.82	2.91	Automated - Genesis	119653	Echinacea purpurea	Leaves		
30	5.3948251	323.68951	5.369227	5.405056	93.033432	536.339177	527.1719629	0.0358296	INF	No Filter	Trace4	1.86	1.99	Automated - Genesis	119653	Echinacea purpurea	Leaves		
1	4.2045427	252.27256	4.19431	4.214774	163.039627	705.590482	1355.837116	0.0204647	INF	No Filter	Trace8	43.35	44.55	Automated - Genesis	119653	Echinacea purpurea	Leaves		
2	6.9825432	418.95259	6.971803	6.993284	163.039429	922.016192	1687.587807	0.0214813	INF	No Filter	Trace8	56.65	55.45	Automated - Genesis	119653	Echinacea purpurea	Leaves		
1	1.1561301	69.367806	1.130861	1.248526	0	33441.0851	11272.97302	0.1176649	INF	No Filter	Trace10	8.34	20.24	Automated - Genesis	119653	Echinacea purpurea	Leaves	C16H18O9 neochlorogenic acid (3-o-caffeoylquinic acid)	Level 4
2	1.5874931	95.249586	1.544878	2.1575	0	337670.572	36413.12887	0.6126218	INF	No Filter	Trace10	84.23	65.38	Automated - Genesis	119653	Echinacea purpurea	Leaves	C16H18O9 chlorogenic acid (5-o-caffeoylquinic acid); C16H18O9 cis 5-o-caffeoylquinic acid	Level 4
3	2.2333275	133.99965	2.1575	2.330139	0	21072.1998	4356.166825	0.1726392	INF	No Filter	Trace10	5.26	7.82	Automated - Genesis	119653	Echinacea purpurea	Leaves		
4	2.3751588	142.50953	2.362554	2.472615	0	8722.01427	3649.016747	0.1100608	INF	No Filter	Trace10	2.18	6.55	Automated - Genesis	119653	Echinacea purpurea	Leaves	C16H18O9 4-o-caffeoylquinic acid (crytochlorogenic acid)	Level 4
1	4.8128693	288.77216	4.813716	4.956799	0	4.71E-13	-84.27753126	0.143082851	INF	No Filter	Trace11	0	-1.22	Manual	119653	Echinacea purpurea	Leaves	C25H24O12 3,5-dicaffeolquinic acid	Level 4
2	4.8801884	292.8113	4.813293	4.977484	0	18433.165	6986.432971	0.1641911	INF	No Filter	Trace11	100	101.22	Manual	119653	Echinacea purpurea	Leaves	C25H24O12 3,5-dicaffeolquinic acid	Level 4
1	1.5037508	90.225048	1.369507	1.573911	0	543893.333	70739.1546	0.204404073	INF	No Filter	Trace12	100	106.49	Manual	119653	Echinacea purpurea	Leaves	C13H12O9 caftaric acid	Level 4
1	0.6254168	37.525008	0.602292	0.643317	473.072906	93665.3631	91956.99189	0.04102505	INF	No Filter	Trace13	100	100	Automated - Genesis	119653	Echinacea purpurea	Leaves	C22H18O12 cichoric acid	Level 4
1	0.8480796	50.884773	0.840122	0.857138	137.023788	1228.59948	2926.985424	0.01701628	INF	No Filter	Trace17	10.37	12.97	Automated - Genesis	119653	Echinacea purpurea	Leaves		
2	2.3715786	142.29472	2.362554	2.380292	137.024353	282.313478	675.4919044	0.017738	INF	No Filter	Trace17	2.38	2.99	Automated - Genesis	119653	Echinacea purpurea	Leaves		
3	3.1485445	188.91267	3.136927	3.160162	137.023758	1280.36466	2253.753041	0.0232349	INF	No Filter	Trace17	10.81	9.99	Automated - Genesis	119653	Echinacea purpurea	Leaves		
4	4.0206618	241.23971	4.012771	4.031262	137.023956	978.615543	1936.704913	0.0184918	INF	No Filter	Trace17	8.26	8.58	Automated - Genesis	119653	Echinacea purpurea	Leaves		
5	4.0785468	244.71281	4.055307	4.087539	137.023727	1682.38673	2365.317756	0.0322324	INF	No Filter	Trace17	14.2	10.48	Automated - Genesis	119653	Echinacea purpurea	Leaves		
6	5.2125322	312.75193	5.203836	5.220424	137.024048	280.798416	672.9440338	0.0165882	INF	No Filter	Trace17	2.37	2.98	Automated - Genesis	119653	Echinacea purpurea	Leaves		
7	6.2859153	377.15492	6.277515	6.295417	137.023743	1904.68366	4351.677628	0.0179012	INF	No Filter	Trace17	16.08	19.28	Automated - Genesis	119653	Echinacea purpurea	Leaves		
8	6.8540404	411.24242	6.845641	6.865139	137.023987	944.72559	1798.242813	0.0194988	INF	No Filter	Trace17	7.98	7.97	Automated - Genesis	119653	Echinacea purpurea	Leaves		
9	10.574631	634.47786	10.56301	10.58625	137.023743	1209.13152	2128.365553	0.023235	INF	No Filter	Trace17	10.21	9.43	Automated - Genesis	119653	Echinacea purpurea	Leaves		
10	10.774672	646.48032	10.76221	10.78629	137.023788	1168.97012	1902.2019	0.024081	INF	No Filter	Trace17	9.87	8.43	Automated - Genesis	119653	Echinacea purpurea	Leaves		
11	11.828089	709.68534	11.81647	11.83971	137.023895	884.284165	1556.539953	0.023235	INF	No Filter	Trace17	7.47	6.9	Automated - Genesis	119653	Echinacea purpurea	Leaves		
1	0.6218361	37.310167	0.614676	0.638498	153.018555	3157.93741	4455.924464	0.0238219	39.72017966	No Filter	Trace18	100	100	Automated - ICIS	119653	Echinacea purpurea	Leaves		
1	5.3499387	320.99632	5.339706	5.36177	563.138611	1001.64215	1842.159305	0.0220646	INF	No Filter	Trace23	33.39	33.5	Automated - Genesis	119653	Echinacea purpurea	Leaves		
2	8.0050365	480.30219	7.994296	8.015777	563.140747	903.111393	1652.978709	0.0214813	INF	No Filter	Trace23	30.1	30.06	Automated - Genesis	119653	Echinacea purpurea	Leaves		
3	8.7908144	527.44886	8.780073	8.801555	563.141296	1095.15948	2004.511923	0.0214815	INF	No Filter	Trace23	36.51	36.45	Automated - Genesis	119653	Echinacea purpurea	Leaves		
1	3.1555152	189.33091	3.141574	3.169456	447.093201	1539.60097	2171.05718	0.0278816	INF	No Filter	Trace24	100	100	Automated - Genesis	119653	Echinacea purpurea	Leaves		
1	0.8429305	50.575829	0.832632	0.853229	216.051086	1604.47488	3063.452221	0.02059637	INF	No Filter	Trace25	35.5	36.59	Automated - Genesis	119653	Echinacea purpurea	Leaves	i9G 2-hydroxyacryloyl-N-glutamate C8H11NO6	Level 4
2	0.8651472	51.908829	0.853229	0.875445	216.050629	1651.55745	2895.590368	0.02221668	INF	No Filter	Trace25	36.54	34.58	Automated - Genesis	119653	Echinacea purpurea	Leaves	i9G 2-hydroxyacryloyl-N-glutamate C8H11NO6	Level 4
3	0.9320898	55.925385	0.921791	0.942388	216.051041	1264.07509	2413.526092	0.020597	INF	No Filter	Trace25	27.97	28.83	Automated - Genesis	119653	Echinacea purpurea	Leaves		
1	5.5980237	335.88142	5.499747	6.062709	0	6328780.36	742504.8899	0.5629611	145.8706655	No Filter	Trace26	100	100	Automated - Genesis	119653	Echinacea purpurea	Leaves	C10H10O4 Ferulic acid	Level 4
1	1.5346118	92.076708	1.515255	1.557934	0	5155.61596	3321.783132	0.0426792	INF	No Filter	Trace27	1.96	5.36	Automated - ICIS	119653	Echinacea purpurea	Leaves	C17H22O10 Sinapoyl-glucose	Level 3
2	2.1085116	126.5107	2.063489	2.167766	0	15622.1715	4308.086073	0.1042767	INF	No Filter	Trace27	5.95	6.95	Automated - ICIS	119653	Echinacea purpurea	Leaves	C17H22O10 Sinapoyl-glucose	Level 3
3	2.4043859	144.26315	2.319875	2.53155	385.114655	94671.0806	18476.58887	0.2116747	INF	No Filter	Trace27	36.08	29.8	Automated - ICIS	119653	Echinacea purpurea	Leaves	C17H22O10 Sinapoyl-glucose	Level 3
4	2.7702407	166.21444	2.723772	2.862545	385.114838	66560.6074	20138.08207	0.1387729	INF	No Filter	Trace27	25.37	32.48	Automated - ICIS	119653	Echinacea purpurea	Leaves	C17H22O10 Sinapoyl-glucose	Level 3
5	3.6488884	218.9333	3.600745	3.765053	385.114227	80377.042	15756.00827	0.1643081	INF	No Filter	Trace27	30.63	25.41	Automated - ICIS	119653	Echinacea purpurea	Leaves	C17H22O10 Sinapoyl-glucose	Level 3
1	7.5887921	455.32753	7.578052	7.599533	339.071442	1120.57682	2050.99975	0.0214812	INF	No Filter	Trace28	55.62	55.62	Automated - Genesis	119653	Echinacea purpurea	Leaves		
2	9.1695742	550.17445	9.158834	9.180315	339.071014	894.174218	1636.634345	0.0214812	INF	No Filter	Trace28	44.38	44.38	Automated - Genesis	119653	Echinacea purpurea	Leaves		
1	10.011056	600.66336	9.997115	10.02585	510.081848	1190.39832	1675.639197	0.028735	INF	No Filter	Trace30	100	100	Automated - Genesis	119653	Echinacea purpurea	Leaves		
1	2.377508	142.65048	2.36724	2.387773	137.023972	771.625013	1478.113637	0.0205324	INF	No Filter	Trace1	5.18	6.21	Automated - Genesis	119654	Echinacea purpurea	Flowers		
2	4.4951927	269.71156	4.484452	4.505933	137.024292	310.19646	567.7568057	0.0214813	INF	No Filter	Trace1	2.08	2.38	Automated - Genesis	119654	Echinacea purpurea	Flowers		
3	6.3426994	380.56196	6.331959	6.35344	137.023743	1107.5833	2027.229813	0.0214812	INF	No Filter	Trace1	7.43	8.51	Automated - Genesis	119654	Echinacea purpurea	Flowers		
4	7.5776083	454.6565	7.566867	7.588349	137.024017	1068.79257	1956.221408	0.0214812	INF	No Filter	Trace1	7.17	8.22	Automated - Genesis	119654	Echinacea purpurea	Flowers		
5	8.1604231	489.62539	8.147342	8.172403	0	1896.53012	3079.26019	0.025061	INF	No Filter	Trace1	12.72	12.93	Automated - Genesis	119654	Echinacea purpurea	Flowers		
6	8.366948	502.01688	8.354603	8.377689	137.023727	784.505829	1429.591115	0.023086	INF	No Filter	Trace1	5.26	6	Automated - Genesis	119654	Echinacea purpurea	Flowers		
7	9.2832806	556.99684	9.270857	9.294021	137.023727	780.985984	1422.882585	0.0231638	INF	No Filter	Trace1	5.24	5.98	Automated - Genesis	119654	Echinacea purpurea	Flowers		
8	9.392292	563.53752	9.381552	9.403033	136.016434	782.558087	1432.31636	0.0214812	INF	No Filter	Trace1	5.25	6.01	Automated - Genesis	119654	Echinacea purpurea	Flowers		
9	9.7571203	585.42722	9.74318	9.771061	137.023804	1474.17805	2078.809319	0.0278818	INF	No Filter	Trace1	9.89	8.73	Automated - Genesis	119654	Echinacea purpurea	Flowers		
10	10.400588	624.03528	10.38665	10.41453	137.023834	1010.57828	1425.103595	0.027882	INF	No Filter	Trace1	6.78	5.98	Automated - Genesis	119654	Echinacea purpurea	Flowers		
11	11.281315	676.8789	11.26651	11.29526	137.023788	896.466736	1241.977743	0.028747	INF	No Filter	Trace1	6.01	5.22	Automated - Genesis	119654	Echinacea purpurea	Flowers		
12	11.799943	707.99658	11.786	11.81388	137.023727	1232.98202	1738.682373	0.027882	INF	No Filter	Trace1	8.27	7.3	Automated - Genesis	119654	Echinacea purpurea	Flowers		
13	13.09302	785.5812	13.07908	13.10782	137.023773	1364.24559	1920.348317	0.028737	INF	No Filter	Trace1	9.15	8.06	Automated - Genesis	119654	Echinacea purpurea	Flowers		
14	13.17055	790.233	13.15661	13.18449	137.023727	1428.36542	2014.233571	0.027882	INF	No Filter	Trace1	9.58	8.46	Automated - Genesis	119654	Echinacea purpurea	Flowers		
1	5.5797256	334.78354	5.568985	5.590466	89.0395203	220.841047	404.2128592	0.0214809	INF	No Filter	Trace2	100	100	Automated - Genesis	119654	Echinacea purpurea	Flowers		
1	0.6241768	37.450609	0.609856	0.691502	0	63908.8545	25130.36008	0.08164621	8.493737528	No Filter	Trace3	74.43	83.95	Automated - ICIS	119654	Echinacea purpurea	Flowers		
2	1.7583176	105.49906	1.706774	1.962279	137.023193	21957.4031	4806.188117	0.255505091	2.497557525	No Filter	Trace3	25.57	16.05	Manual	119654	Echinacea purpurea	Flowers		

1	1.5510888	93.065328	1.540821	1.561355	93.0333405	461.427386	882.5554211	0.0205339	INF	No Filter	Trace4	2	3.49	Automated - Genesis	119654	Echinacea purpurea	Flowers		
2	3.8300592	229.80355	3.804478	3.840293	93.0330582	686.437989	772.4283209	0.0358142	INF	No Filter	Trace4	2.98	3.05	Automated - Genesis	119654	Echinacea purpurea	Flowers		
3	3.867481	232.04886	3.85564	3.877713	93.0332794	315.297194	579.7162144	0.0220726	INF	No Filter	Trace4	1.37	2.29	Automated - Genesis	119654	Echinacea purpurea	Flowers		
4	3.9237613	235.42568	3.913529	3.94583	93.0333939	731.870344	678.7529009	0.0323011	INF	No Filter	Trace4	3.18	2.68	Automated - Genesis	119654	Echinacea purpurea	Flowers		
5	4.0123389	240.74033	3.991874	4.024175	93.0328903	619.391116	628.4055184	0.0323006	INF	No Filter	Trace4	2.69	2.48	Automated - Genesis	119654	Echinacea purpurea	Flowers		
6	4.0717575	244.30545	4.059989	4.095802	0	867.342101	876.3779804	0.0358134	INF	No Filter	Trace4	3.77	3.46	Automated - Genesis	119654	Echinacea purpurea	Flowers		
7	4.1229769	247.37861	4.112744	4.133209	93.0330963	331.587301	637.4387281	0.0204646	INF	No Filter	Trace4	1.44	2.52	Automated - Genesis	119654	Echinacea purpurea	Flowers		
8	4.2217998	253.30799	4.211568	4.232032	93.0331573	362.964878	697.8413231	0.020464	INF	No Filter	Trace4	1.58	2.76	Automated - Genesis	119654	Echinacea purpurea	Flowers		
9	4.2473801	254.84281	4.237149	4.257612	93.0333328	355.088246	682.632038	0.0204638	INF	No Filter	Trace4	1.54	2.7	Automated - Genesis	119654	Echinacea purpurea	Flowers		
10	4.305254	258.31524	4.279675	4.315489	93.0334244	596.541549	626.5963999	0.0358149	INF	No Filter	Trace4	2.59	2.47	Automated - Genesis	119654	Echinacea purpurea	Flowers		
11	4.3462024	260.77214	4.335956	4.358042	93.0332565	323.213695	618.0166456	0.0220855	INF	No Filter	Trace4	1.4	2.44	Automated - Genesis	119654	Echinacea purpurea	Flowers		
12	4.432728	265.96368	4.404087	4.44865	0	984.957043	684.5439668	0.0445636	INF	No Filter	Trace4	4.28	2.7	Automated - Genesis	119654	Echinacea purpurea	Flowers		
13	4.5302577	271.81546	4.473711	4.543339	0	1751.06469	1020.016998	0.0696275	INF	No Filter	Trace4	7.61	4.03	Automated - Genesis	119654	Echinacea purpurea	Flowers		
14	4.5768001	274.60801	4.546919	4.608876	0	1602.80767	849.6271742	0.0619568	INF	No Filter	Trace4	6.96	3.36	Automated - Genesis	119654	Echinacea purpurea	Flowers		
15	4.6687323	280.12394	4.648268	4.678965	93.0333099	501.53791	529.021637	0.030697	INF	No Filter	Trace4	2.18	2.09	Automated - Genesis	119654	Echinacea purpurea	Flowers		
16	4.6959152	281.75491	4.684081	4.706147	93.0330505	397.917693	732.0206751	0.0220662	INF	No Filter	Trace4	1.73	2.89	Automated - Genesis	119654	Echinacea purpurea	Flowers		
17	4.7498543	284.99126	4.736844	4.762427	0	623.72338	927.0809027	0.0255829	INF	No Filter	Trace4	2.71	3.66	Automated - Genesis	119654	Echinacea purpurea	Flowers		
18	4.8100937	288.60562	4.799862	4.820326	93.0334549	251.026188	482.5882838	0.020464	INF	No Filter	Trace4	1.09	1.91	Automated - Genesis	119654	Echinacea purpurea	Flowers		
19	4.8305582	289.83349	4.820326	4.84079	93.0331879	345.454266	664.1388481	0.0204639	INF	No Filter	Trace4	1.5	2.62	Automated - Genesis	119654	Echinacea purpurea	Flowers		
20	4.8833437	293.00062	4.862864	4.903808	93.0333099	965.904262	775.8239097	0.0409438	INF	No Filter	Trace4	4.2	3.06	Automated - Genesis	119654	Echinacea purpurea	Flowers		
21	4.9309246	295.85548	4.919156	4.946335	0	758.525978	1060.763964	0.0271791	INF	No Filter	Trace4	3.29	4.19	Automated - Genesis	119654	Echinacea purpurea	Flowers		
22	4.9667994	298.00796	4.946335	4.977031	93.0331268	687.060951	671.1968772	0.030696	INF	No Filter	Trace4	2.98	2.65	Automated - Genesis	119654	Echinacea purpurea	Flowers		
23	5.0077285	300.46371	4.987263	5.01796	93.0333023	704.644863	720.7453852	0.0306968	INF	No Filter	Trace4	3.06	2.85	Automated - Genesis	119654	Echinacea purpurea	Flowers		
24	5.06715	304.029	5.055382	5.091195	0	1173.1579	1287.947365	0.0358125	INF	No Filter	Trace4	5.1	5.09	Automated - Genesis	119654	Echinacea purpurea	Flowers		
25	5.1014257	306.08554	5.091195	5.113263	93.033165	409.742157	723.3866867	0.022068	INF	No Filter	Trace4	1.78	2.86	Automated - Genesis	119654	Echinacea purpurea	Flowers		
26	5.1262697	307.57618	5.113263	5.138843	0	644.366983	963.589629	0.0255803	INF	No Filter	Trace4	2.8	3.81	Automated - Genesis	119654	Echinacea purpurea	Flowers		
27	5.1490755	308.94453	5.138843	5.159307	93.0333023	302.436593	581.440142	0.0204642	INF	No Filter	Trace4	1.31	2.3	Automated - Genesis	119654	Echinacea purpurea	Flowers		
28	5.1825557	310.95334	5.159307	5.196728	0	1063.29641	1072.451851	0.0374207	INF	No Filter	Trace4	4.62	4.24	Automated - Genesis	119654	Echinacea purpurea	Flowers		
29	5.2136112	312.81667	5.201843	5.242772	0	960.826551	1023.371305	0.0409284	INF	No Filter	Trace4	4.17	4.04	Automated - Genesis	119654	Echinacea purpurea	Flowers		
30	5.2608961	315.65377	5.242772	5.290414	0	1593.53736	1245.552243	0.0476426	INF	No Filter	Trace4	6.92	4.92	Automated - Genesis	119654	Echinacea purpurea	Flowers		
31	5.303422	318.20532	5.290414	5.331345	0	1402.03517	1148.895973	0.0409303	INF	No Filter	Trace4	6.09	4.54	Automated - Genesis	119654	Echinacea purpurea	Flowers		
32	5.3466964	320.80178	5.336461	5.358548	93.0330048	249.170627	476.6414319	0.0220871	INF	No Filter	Trace4	1.08	1.88	Automated - Genesis	119654	Echinacea purpurea	Flowers		
1	4.8810032	292.86019	4.870755	4.891234	174.075287	766.724284	1473.271819	0.0204793	INF	No Filter	Trace6	100	100	Automated - Genesis	119654	Echinacea purpurea	Flowers		
1	1.0250534	61.503204	1.014752	1.035351	163.039398	906.320947	1730.397499	0.0205989	INF	No Filter	Trace8	8.73	10.02	Automated - Genesis	119654	Echinacea purpurea	Flowers		
2	1.3267328	79.603968	1.316469	1.337007	163.03952	950.830751	1821.28461	0.020538	INF	No Filter	Trace8	9.15	10.55	Automated - Genesis	119654	Echinacea purpurea	Flowers		
3	1.3694266	82.165596	1.357537	1.379691	163.039459	820.274229	1563.974341	0.0221539	INF	No Filter	Trace8	7.9	9.06	Automated - Genesis	119654	Echinacea purpurea	Flowers		
4	2.1313813	127.88288	2.121117	2.141646	163.039368	1283.07282	2457.804428	0.0205292	INF	No Filter	Trace8	12.35	14.23	Automated - Genesis	119654	Echinacea purpurea	Flowers		
5	2.3245655	139.47393	2.314302	2.334834	163.039505	870.088568	1666.203982	0.0205325	INF	No Filter	Trace8	8.38	9.65	Automated - Genesis	119654	Echinacea purpurea	Flowers		
6	2.5093453	150.56072	2.495404	2.524129	163.039322	1230.17251	1731.624248	0.028725	INF	No Filter	Trace8	11.84	10.03	Automated - Genesis	119654	Echinacea purpurea	Flowers		
7	2.5613048	153.67829	2.547364	2.575246	163.039398	1248.29374	1760.303484	0.0278816	INF	No Filter	Trace8	12.02	10.19	Automated - Genesis	119654	Echinacea purpurea	Flowers		
8	3.1054398	186.32639	3.091499	3.120237	163.039658	1027.6067	1356.356325	0.0287384	INF	No Filter	Trace8	9.89	7.86	Automated - Genesis	119654	Echinacea purpurea	Flowers		
9	7.5187245	451.12347	7.507984	7.531066	163.039566	1033.89091	1745.229833	0.0230817	INF	No Filter	Trace8	9.95	10.11	Automated - Genesis	119654	Echinacea purpurea	Flowers		
10	9.649389	578.96334	9.635448	9.66333	163.04007	1016.41804	1433.295551	0.0278818	INF	No Filter	Trace8	9.79	8.3	Automated - Genesis	119654	Echinacea purpurea	Flowers		
1	6.6605691	399.63415	6.649828	6.67131	353.087585	990.15098	1812.300163	0.0214812	INF	No Filter	Trace10	26.49	27.24	Automated - Genesis	119654	Echinacea purpurea	Flowers		
2	6.9856115	419.13669	6.974871	6.996353	353.08844	880.127668	1610.89969	0.0214813	INF	No Filter	Trace10	23.55	24.22	Automated - Genesis	119654	Echinacea purpurea	Flowers		
3	7.0203964	421.22378	7.010165	7.032233	353.089294	977.922763	1599.944957	0.0220683	INF	No Filter	Trace10	26.16	24.05	Automated - Genesis	119654	Echinacea purpurea	Flowers		
4	8.7528592	525.17155	8.742119	8.7636	353.089355	889.86146	1628.719223	0.021481	INF	No Filter	Trace10	23.81	24.49	Automated - Genesis	119654	Echinacea purpurea	Flowers		
1	1.4981379	89.888274	1.369343	1.594161	0	123193.387	17549.65569	0.224817518	INF	No Filter	Trace12	100	100	Manual	119654	Echinacea purpurea	Flowers	C13H12O9 caftaric acid	Level 4
1	0.6471431	38.828583	0.614921	0.657884	473.074585	17152.3881	14370.79793	0.0429625	INF	No Filter	Trace13	20.21	13.19	Automated - Genesis	119654	Echinacea purpurea	Flowers	C22H18O12 cichoric acid	Level 4
2	0.7642914	45.857485	0.753989	0.774591	473.072998	2396.06221	4572.907465	0.02060211	INF	No Filter	Trace13	2.82	4.2	Automated - Genesis	119654	Echinacea purpurea	Flowers		
3	0.7951873	47.711237	0.784889	0.805486	473.074738	2235.70346	4268.663785	0.02059622	INF	No Filter	Trace13	2.63	3.92	Automated - Genesis	119654	Echinacea purpurea	Flowers		
4	5.3392399	320.35439	5.329004	5.349472	473.072723	1000.72473	1922.46969	0.0204673	INF	No Filter	Trace13	1.18	1.76	Automated - Genesis	119654	Echinacea purpurea	Flowers		
5	5.4165725	324.99435	5.405832	5.427313	473.073944	839.089993	1535.801162	0.0214813	INF	No Filter	Trace13	0.99	1.41	Automated - Genesis	119654	Echinacea purpurea	Flowers		
6	5.6219512	337.31707	5.611211	5.632692	473.073059	1057.23452	1935.066999	0.0214813	INF	No Filter	Trace13	1.25	1.78	Automated - Genesis	119654	Echinacea purpurea	Flowers		
7	5.7703448	346.22069	5.759605	5.782681	473.071472	1360.21239	2136.421973	0.0230764	INF	No Filter	Trace13	1.6	1.96	Automated - Genesis	119654	Echinacea purpurea	Flowers		
8	5.8738309	352.42985	5.8636	5.884064	473.071838	919.564606	1767.037932	0.0204646	INF	No Filter	Trace13	1.08	1.62	Automated - Genesis	119654	Echinacea purpurea	Flowers		
9	5.9352251	356.11351	5.924992	5.947055	473.072327	1141.58163	2099.593016	0.022063	INF	No Filter	Trace13	1.35	1.93	Automated - Genesis	119654	Echinacea purpurea	Flowers		
10	6.1978909	371.87345	6.17481	6.208632	473.074249	2263.13729	1995.334152	0.0338219	INF	No Filter	Trace13	2.67	1.83	Automated - Genesis	119654	Echinacea purpurea	Flowers		
11	6.2265326	373.59196	6.215792	6.237273	473.07077	1354.78876	2479.680872	0.0214813	INF	No Filter	Trace13	1.6	2.28	Automated - Genesis	119654	Echinacea purpurea	Flowers		
12	6.3621951	381.73171	6.34986	6.372936	473.072906	928.739078	1630.335691	0.0230759	INF	No Filter	Trace13	1.09	1.5	Automated - Genesis	119654	Echinacea purpurea	Flowers		
13	6.4087378	384.52427	6.397997	6.419478	473.071747	1010.44829	1849.438398	0.0214813	INF	No Filter	Trace13	1.19	1.7	Automated - Genesis	119654	Echinacea purpurea	Flowers		
14	6.5093434	390.5606	6.496262	6.521323	0	1832.42517	2975.159293	0.0250615	INF	No Filter	Trace13	2.16	2.73	Automated - Genesis	119654	Echinacea purpurea	Flowers		
15	6.5825478	394.95287	6.526504	6.594528	0	5360.96085	3590.24144	0.0680241	INF	No Filter	Trace13	6.32	3.3	Automated - Genesis	119654	Echinacea purpurea	Flowers		
16	6.6462482	398.77489	6.635508	6.656989	473.070923	978.621588	1791.179734	0.0214813	INF	No Filter	Trace13	1.15	1.64	Automated - Genesis	119654	Echinacea purpurea	Flowers		
17	6.7926598	407.55959	6.765994	6.803401	473.072998	2110.13303	1814.53058	0.0374062	INF	No Filter	Trace13	2.49	1.67	Automated - Genesis	119654	Echinacea purpurea	Flowers		
18	7.0101645	420.60987	6.999933	7.020396	473.072937	1453.95776	2794.200637	0.0204638	INF	No Filter	Trace13	1.71	2.56	Automated - Genesis	119654	Echinacea purpurea	Flowers		
19	7.0731895	424.39137	7.062942	7.083421	473.073578	995.637428	1912.993584	0.0204797	INF	No Filter	Trace13	1.17	1.76	Automated - Genesis	119654	Echinacea purpurea	Flowers		
20	7.115718	426.94308	7.103887	7.12595	473.072357	966.783759	1849.388675	0.0220627	INF	No Filter	Trace13	1.14	1.7	Automated - Genesis	119654	Echinacea purpurea	Flowers		
21	7.1940662	431.64397	7.171995	7.204298	473.072876	2223.56051	2128.086608	0.032303	INF	No Filter	Trace13	2.62	1.95	Automated - Genesis	119654	Echinacea purpurea	Flowers		
22	7.2401171	434.40703	7.214537	7.250349	473.074768	2361.77598	2324.902612	0.0358125	INF	No Filter	Trace13	2.78	2.13	Automated - Genesis	119654	Echinacea purpurea	Flowers		
23	7.2656978	435.94187	7.255464	7.277537	473.072571	1214.94305	2233.651317	0.0220725	INF	No Filter	Trace13	1.43	2.05	Automated - Genesis	119654	Echinacea purpurea	Flowers		
24	7.2928848	437.57309	7.282653	7.303116	473.074432	864.540902	1661.372819	0.0204637	INF	No Filter	Trace13	1.02	1.53	Automated - Genesis	119654	Echinacea purpurea	Flowers		
25	7.3389319	440.33591	7.3287	7.349181	473.073273	1087.21932	2086.286536	0.0204807	INF	No Filter	Trace13	1.28	1.92	Automated - Genesis	119654	Echinacea purpurea	Flowers		
26	7.4020166	444.121	7.391784	7.412248	473.071075	1222.7214	2349.844776	0.0204635	INF	No Filter	Trace13	1.44	2.16	Automated - Genesis	119654	Echinacea purpurea	Flowers		
27	7.4275965	445.65579	7.417365	7.437834	473.071075	1278.00987	2454.77192	0.0204693	INF	No Filter	Trace13	1.51	2.25	Automated - Genesis	119654	Echinacea purpurea	Flowers		
28	7.4496625	446.97975	7.437834	7.459894	473.072632	849.315288	1624.737377	0.02206	INF	No Filter	Trace13	1	1.49	Automated - Genesis	119654	Echinacea purpurea	Flowers		

29	7.4803594	448.82156	7.470126	7.490591	473.070892	1335.9421	2567.374212	0.0204649	INF	No Filter	Trace13	1.57	2.36	Automated - Genesis	119654	Echinacea purpurea	Flowers		
30	7.5115641	450.69385	7.500824	7.522305	473.072479	1143.89219	2093.687536	0.0214812	INF	No Filter	Trace13	1.35	1.92	Automated - Genesis	119654	Echinacea purpurea	Flowers		
31	7.538226	452.29356	7.527485	7.548967	473.072693	1368.07116	2504.008318	0.0214812	INF	No Filter	Trace13	1.61	2.3	Automated - Genesis	119654	Echinacea purpurea	Flowers		
32	7.5668674	454.01204	7.556127	7.577608	473.071045	1110.13143	2031.89165	0.0214812	INF	No Filter	Trace13	1.31	1.87	Automated - Genesis	119654	Echinacea purpurea	Flowers		
33	7.5906895	455.44137	7.577608	7.60625	0	2310.33031	2759.467598	0.0286413	INF	No Filter	Trace13	2.72	2.53	Automated - Genesis	119654	Echinacea purpurea	Flowers		
34	7.7780846	466.68508	7.745497	7.790065	0	3739.50339	2712.816297	0.0445677	INF	No Filter	Trace13	4.41	2.49	Automated - Genesis	119654	Echinacea purpurea	Flowers		
35	7.8366074	470.19644	7.825867	7.859689	473.072388	2323.78211	2314.720021	0.0338227	INF	No Filter	Trace13	2.74	2.12	Automated - Genesis	119654	Echinacea purpurea	Flowers		
36	7.9098121	474.58873	7.899072	7.920553	473.073547	1467.14892	2685.336378	0.0214813	INF	No Filter	Trace13	1.73	2.46	Automated - Genesis	119654	Echinacea purpurea	Flowers		
37	7.9579633	477.4778	7.947223	7.968704	473.073761	1078.14995	1973.398963	0.0214812	INF	No Filter	Trace13	1.27	1.81	Automated - Genesis	119654	Echinacea purpurea	Flowers		
38	7.9794445	478.76667	7.968704	7.990185	473.07373	824.256633	1508.642691	0.0214813	INF	No Filter	Trace13	0.97	1.38	Automated - Genesis	119654	Echinacea purpurea	Flowers		
39	8.0045059	480.27035	7.993765	8.015247	473.07251	1175.18242	2150.943492	0.0214812	INF	No Filter	Trace13	1.39	1.97	Automated - Genesis	119654	Echinacea purpurea	Flowers		
40	8.0370922	482.22553	8.015247	8.049072	0	3430.33854	3334.105334	0.0338254	INF	No Filter	Trace13	4.04	3.06	Automated - Genesis	119654	Echinacea purpurea	Flowers		
41	8.0705533	484.2332	8.056233	8.099195	473.074219	5083.0492	4149.157208	0.0429624	INF	No Filter	Trace13	5.99	3.81	Automated - Genesis	119654	Echinacea purpurea	Flowers		
1	8.3080596	498.48358	8.297319	8.318801	269.044891	804.504684	1472.495646	0.0214812	INF	No Filter	Trace16	100	100	Automated - Genesis	119654	Echinacea purpurea	Flowers		
1	2.377508	142.65048	2.369581	2.386533	137.023972	771.923824	1845.081024	0.0169521	INF	No Filter	Trace17	5.47	6.66	Automated - Genesis	119654	Echinacea purpurea	Flowers		
2	4.4951927	269.71156	4.485692	4.503593	137.024292	310.196041	708.7122952	0.0179009	INF	No Filter	Trace17	2.2	2.56	Automated - Genesis	119654	Echinacea purpurea	Flowers		
3	6.3426994	380.56196	6.334299	6.3522	137.023743	1107.58004	2530.524829	0.017901	INF	No Filter	Trace17	7.85	9.14	Automated - Genesis	119654	Echinacea purpurea	Flowers		
4	7.5776083	454.6565	7.569208	7.587109	137.024017	1068.7932	2441.887354	0.0179012	INF	No Filter	Trace17	7.57	8.82	Automated - Genesis	119654	Echinacea purpurea	Flowers		
5	8.1604231	489.62539	8.149683	8.171164	0	1896.53167	3593.195366	0.0214813	INF	No Filter	Trace17	13.44	12.98	Automated - Genesis	119654	Echinacea purpurea	Flowers		
6	8.366948	502.01688	8.358548	8.376449	137.023727	781.065343	1784.511943	0.0179011	INF	No Filter	Trace17	5.53	6.45	Automated - Genesis	119654	Echinacea purpurea	Flowers		
7	9.2832806	556.99684	9.274881	9.292782	137.023727	777.393948	1776.137903	0.0179009	INF	No Filter	Trace17	5.51	6.42	Automated - Genesis	119654	Echinacea purpurea	Flowers		
8	9.7571203	585.42722	9.745503	9.768738	137.023804	1474.17709	2594.909844	0.0232345	INF	No Filter	Trace17	10.45	9.37	Automated - Genesis	119654	Echinacea purpurea	Flowers		
9	10.400588	624.03528	10.38897	10.41221	137.023834	1010.56404	1778.910318	0.023235	INF	No Filter	Trace17	7.16	6.43	Automated - Genesis	119654	Echinacea purpurea	Flowers		
10	11.281315	676.8789	11.26883	11.29293	137.023788	890.233049	1550.320293	0.024101	INF	No Filter	Trace17	6.31	5.6	Automated - Genesis	119654	Echinacea purpurea	Flowers		
11	11.799943	707.99658	11.78833	11.81156	137.023727	1232.98643	2170.340475	0.023234	INF	No Filter	Trace17	8.74	7.84	Automated - Genesis	119654	Echinacea purpurea	Flowers		
12	13.09302	785.5812	13.0814	13.10464	137.023773	1361.78441	2397.108145	0.023234	INF	No Filter	Trace17	9.65	8.66	Automated - Genesis	119654	Echinacea purpurea	Flowers		
13	13.17055	790.233	13.15893	13.18217	137.023727	1428.35378	2514.302044	0.023234	INF	No Filter	Trace17	10.12	9.08	Automated - Genesis	119654	Echinacea purpurea	Flowers		
1	11.879787	712.78722	11.86585	11.89373	216.981461	1072.96198	1513.015792	0.027882	INF	No Filter	Trace20	100	100	Automated - Genesis	119654	Echinacea purpurea	Flowers		
1	4.6408119	278.44871	4.597042	4.736844	593.151978	125108.545	33471.93134	0.1398016	404.0500268	No Filter	Trace22	100	100	Automated - ICIS	119654	Echinacea purpurea	Flowers	C27H30O15 Kaempferol diglycosides	Level 3
1	2.556658	153.39948	2.542717	2.570599	563.142395	1232.71312	1738.331243	0.0278813	INF	No Filter	Trace23	55.41	50.94	Automated - Genesis	119654	Echinacea purpurea	Flowers		
2	5.6024462	336.14677	5.591705	5.614791	563.139954	991.834885	1673.920596	0.0230854	INF	No Filter	Trace23	44.59	49.06	Automated - Genesis	119654	Echinacea purpurea	Flowers		
1	4.9219314	295.31588	4.9117	4.932164	447.092865	867.249076	1666.542058	0.0204641	INF	No Filter	Trace24	27.65	34.86	Automated - Genesis	119654	Echinacea purpurea	Flowers	C21H20O11 kaempferol-O-glucoside	Level 3
2	10.297506	617.85036	10.28357	10.31145	447.092316	1095.51064	1544.856066	0.027881	INF	No Filter	Trace24	34.92	32.32	Automated - Genesis	119654	Echinacea purpurea	Flowers		
3	11.768869	706.13214	11.75493	11.78368	447.091766	1174.17773	1568.916564	0.02875	INF	No Filter	Trace24	37.43	32.82	Automated - Genesis	119654	Echinacea purpurea	Flowers		
1	13.935698	836.14188	13.92176	13.95051	216.051987	2080.92076	2779.993581	0.028753	INF	No Filter	Trace25	100	100	Automated - Genesis	119654	Echinacea purpurea	Flowers		
1	0.6364024	38.184141	0.602464	0.706405	193.049911	75035.7867	30177.80316	0.10394089	INF	No Filter	Trace26	11.4	17.29	Automated - Genesis	119654	Echinacea purpurea	Flowers		
2	0.7270033	43.620195	0.712794	0.759138	0	11730.6205	8989.840444	0.04634413	INF	No Filter	Trace26	1.78	5.15	Automated - Genesis	119654	Echinacea purpurea	Flowers		
3	0.7717819	46.306912	0.759138	0.784889	0	4510.81006	6689.432306	0.02575112	INF	No Filter	Trace26	0.69	3.83	Automated - Genesis	119654	Echinacea purpurea	Flowers		
4	0.8248582	49.491493	0.810635	0.836396	0	4571.78657	6706.483253	0.02576137	INF	No Filter	Trace26	0.69	3.84	Automated - Genesis	119654	Echinacea purpurea	Flowers		
5	0.9049763	54.298576	0.894661	0.915276	193.049728	1705.31794	3250.964515	0.02061547	INF	No Filter	Trace26	0.26	1.86	Automated - Genesis	119654	Echinacea purpurea	Flowers		
6	2.6527747	159.16648	2.396799	3.276867	193.049927	239485.293	14597.83005	0.8800671	INF	No Filter	Trace26	36.38	8.36	Automated - Genesis	119654	Echinacea purpurea	Flowers		
7	5.5904658	335.42795	5.499356	5.966292	0	240507.248	30003.66457	0.4669364	8.692045175	No Filter	Trace26	36.54	17.19	Automated - Genesis	119654	Echinacea purpurea	Flowers	C10H10O4 Ferulic acid	Level 4
8	6.3271389	379.62833	6.281836	6.34986	0	6150.85549	3394.558521	0.068024	INF	No Filter	Trace26	0.93	1.94	Automated - Genesis	119654	Echinacea purpurea	Flowers		
9	6.3931773	383.59064	6.372936	6.405158	0	5113.64589	6036.293698	0.0322218	INF	No Filter	Trace26	0.78	3.46	Automated - Genesis	119654	Echinacea purpurea	Flowers		
10	6.421819	385.30914	6.405158	6.442559	0	4619.83644	4468.533155	0.0374013	INF	No Filter	Trace26	0.7	2.56	Automated - Genesis	119654	Echinacea purpurea	Flowers		
11	6.5753874	394.52324	6.555146	6.587368	0	3319.40715	3929.887346	0.032222	INF	No Filter	Trace26	0.5	2.25	Automated - Genesis	119654	Echinacea purpurea	Flowers		
12	6.8522837	411.13702	6.839203	6.865867	0	2501.65567	3580.232253	0.0266645	INF	No Filter	Trace26	0.38	2.05	Automated - Genesis	119654	Echinacea purpurea	Flowers		
13	8.0188267	481.1296	7.968704	8.034752	193.050171	6712.31092	3459.519326	0.0660478	INF	No Filter	Trace26	1.02	1.98	Automated - Genesis	119654	Echinacea purpurea	Flowers		
14	8.4301523	505.80914	8.417071	8.443734	0	2206.49097	3555.578494	0.0266632	INF	No Filter	Trace26	0.34	2.04	Automated - Genesis	119654	Echinacea purpurea	Flowers		
15	8.894443	533.66658	8.867041	8.906423	0	3034.60809	3277.989497	0.0393827	INF	No Filter	Trace26	0.46	1.88	Automated - Genesis	119654	Echinacea purpurea	Flowers		
16	9.4280941	565.68565	9.413773	9.458335	193.049698	3890.13355	4278.814153	0.0445618	INF	No Filter	Trace26	0.59	2.45	Automated - Genesis	119654	Echinacea purpurea	Flowers		
17	9.5012977	570.07786	9.486977	9.522209	193.050385	3942.49657	4452.592761	0.0352318	INF	No Filter	Trace26	0.6	2.55	Automated - Genesis	119654	Echinacea purpurea	Flowers		
18	9.8346483	590.0789	9.795149	9.863374	0	6389.69889	3769.867818	0.068225	INF	No Filter	Trace26	0.97	2.16	Automated - Genesis	119654	Echinacea purpurea	Flowers		
19	9.9075203	594.45122	9.881962	9.973445	193.050369	7554.05386	3769.794762	0.0914823	INF	No Filter	Trace26	1.15	2.16	Automated - Genesis	119654	Echinacea purpurea	Flowers		
20	9.999003	599.94018	9.985062	10.03238	193.050049	4039.78277	3863.047886	0.047316	INF	No Filter	Trace26	0.61	2.21	Automated - Genesis	119654	Echinacea purpurea	Flowers		
21	10.088141	605.28846	10.07188	10.10441	0	2790.88648	3491.100263	0.032528	INF	No Filter	Trace26	0.42	2	Automated - Genesis	119654	Echinacea purpurea	Flowers		
22	10.496716	629.80296	10.48278	10.54403	193.049774	5957.18156	4570.765503	0.061254	INF	No Filter	Trace26	0.91	2.62	Automated - Genesis	119654	Echinacea purpurea	Flowers		
23	12.724808	763.48848	12.70854	12.73875	193.050446	2805.0104	3736.84117	0.030205	INF	No Filter	Trace26	0.43	2.14	Automated - Genesis	119654	Echinacea purpurea	Flowers		
24	12.809308	768.55848	12.79537	12.82557	193.050217	2720.16727	3637.520291	0.030205	INF	No Filter	Trace26	0.41	2.08	Automated - Genesis	119654	Echinacea purpurea	Flowers		
25	13.694681	821.68086	13.65201	13.71095	0	4016.55587	3021.199053	0.058936	INF	No Filter	Trace26	0.61	1.73	Automated - Genesis	119654	Echinacea purpurea	Flowers		
26	13.87064	832.2384	13.85353	13.88458	193.049805	2895.51381	3858.983716	0.031051	INF	No Filter	Trace26	0.44	2.21	Automated - Genesis	119654	Echinacea purpurea	Flowers		
1	2.4907573	149.44544	2.472169	2.549688	0	7535.67792	3857.258354	0.0775182	INF	No Filter	Trace27	9.28	16.38	Automated - ICIS	119654	Echinacea purpurea	Flowers	C17H22O10 Sinapoyl-glucose	Level 3
2	3.6687857	220.12714	3.600401	3.753989	0	56036.48	12857.4723	0.1535881	INF	No Filter	Trace27	69	54.6	Automated - ICIS	119654	Echinacea purpurea	Flowers	C17H22O10 Sinapoyl-glucose	Level 3
3	3.867481	232.04886	3.848183	3.948606	0	17643.5401	6833.339944	0.1004222	INF	No Filter	Trace27	21.72	29.02	Automated - ICIS	119654	Echinacea purpurea	Flowers	C17H22O10 Sinapoyl-glucose	Level 3
1	6.1556695	369.34017	5.993115	7.005049	0	3853830.42	179816.893	1.0119339	28.70410794	No Filter	Trace28	100	100	Automated - Genesis	119654	Echinacea purpurea	Flowers	C15H16O9 E-Sinapoyl-malate	Level 4
1	0.6791139	40.746831	0.668373	0.689854	137.023819	5229.50227	9571.640742	0.02148083	INF	No Filter	Trace1	25.02	28.55	Automated - Genesis	119655	Echinacea purpurea	Roots		
2	3.1135734	186.8144	3.098783	3.127514	137.023773	951.221029	1296.67922	0.0287308	INF	No Filter	Trace1	4.55	3.87	Automated - Genesis	119655	Echinacea purpurea	Roots		
3	5.9985696	359.91418	5.988337	6.009606	137.023727	1347.94634	2590.974823	0.0212687	INF	No Filter	Trace1	6.45	7.73	Automated - Genesis	119655	Echinacea purpurea	Roots		
4	7.0889755	425.33853	7.078743	7.099208	119.013908	703.545447	1352.070671	0.0204646	INF	No Filter	Trace1	3.37	4.03	Automated - Genesis	119655	Echinacea purpurea	Roots		
5	7.6368899	458.21339	7.626149	7.647631	137.023788	1249.06151	2286.149253	0.0214813	INF	No Filter	Trace1	5.98	6.82	Automated - Genesis	119655	Echinacea purpurea	Roots		
6	9.3819432	562.91659	9.371202	9.392683	137.023727	1282.07367	2346.579589	0.0214813	INF	No Filter	Trace1	6.13	7	Automated - Genesis	119655	Echinacea purpurea	Roots		
7	9.528098	571.68588	9.513315	9.542039	137.023727	1022.9426	1369.033209	0.0287238	INF	No Filter	Trace1	4.89	4.08	Automated - Genesis	119655	Echinacea purpurea	Roots		
8	9.5699205	574.19523	9.55598	9.583861	137.023743	2567.61161	3620.761504	0.0278815	INF	No Filter	Trace1	12.28	10.8	Automated - Genesis	119655	Echinacea purpurea	Roots		
9	10.353584	621.21504	10.33964	10.36837	137.023773	1486.75398	1989.536756	0.028725	INF	No Filter	Trace1	7.11	5.93	Automated - Genesis	119655	Echinacea purpurea	Roots		

10	10.809413	648.56478	10.79547	10.82335	137.023743	1856.64677	2618.145101	0.027881	INF	No Filter	Trace1	8.88	7.81	Automated - Genesis	119655	Echinacea purpurea	Roots		
11	12.452746	747.16476	12.43796	12.46669	137.023758	2033.61965	2862.562605	0.028727	INF	No Filter	Trace1	9.73	8.54	Automated - Genesis	119655	Echinacea purpurea	Roots		
12	13.699248	821.95488	13.68447	13.71319	137.023788	1173.02723	1626.089873	0.028719	INF	No Filter	Trace1	5.61	4.85	Automated - Genesis	119655	Echinacea purpurea	Roots		
1	0.6241768	37.450609	0.609856	0.691502	0	63908.8545	25130.36008	0.08164621	8.493737528	No Filter	Trace3	36.52	56.16	Automated - ICIS	119655	Echinacea purpurea	Roots		
2	1.7931377	107.58826	1.706774	1.962279	137.023254	111075.257	19620.80335	0.255505091	9.033989679	No Filter	Trace3	63.48	43.84	Manual	119655	Echinacea purpurea	Roots		
1	0.9137474	54.824841	0.891582	0.965311	0	17156.8986	9182.257636	0.07372956	INF	No Filter	Trace4	37.75	40.03	Automated - Genesis	119655	Echinacea purpurea	Roots		
2	0.9807671	58.846023	0.970463	0.991065	93.0331955	541.70818	1034.684653	0.0206015	INF	No Filter	Trace4	1.19	4.51	Automated - Genesis	119655	Echinacea purpurea	Roots		
3	4.0602124	243.61274	4.049981	4.070445	93.0329437	527.917856	1014.884946	0.020464	INF	No Filter	Trace4	1.16	4.42	Automated - Genesis	119655	Echinacea purpurea	Roots		
4	4.1822716	250.9363	4.169264	4.196442	0	668.330523	942.331125	0.0271785	INF	No Filter	Trace4	1.47	4.11	Automated - Genesis	119655	Echinacea purpurea	Roots		
5	4.4537329	267.22397	4.42583	4.473975	0	1592.78909	1287.515833	0.0481444	INF	No Filter	Trace4	3.5	5.61	Automated - Genesis	119655	Echinacea purpurea	Roots		
6	4.5002758	270.01655	4.477555	4.547181	0	2358.83701	1184.530155	0.0696262	INF	No Filter	Trace4	5.19	5.16	Automated - Genesis	119655	Echinacea purpurea	Roots		
7	4.6843812	281.06287	4.582983	4.701331	93.0332565	4116.10259	1272.812082	0.1183485	INF	No Filter	Trace4	9.06	5.55	Automated - Genesis	119655	Echinacea purpurea	Roots		
8	4.7182159	283.09295	4.706448	4.732028	0	781.994579	1153.788024	0.0255808	INF	No Filter	Trace4	1.72	5.03	Automated - Genesis	119655	Echinacea purpurea	Roots		
9	4.7875684	287.2541	4.774561	4.810375	0	1103.48899	966.478835	0.0358147	INF	No Filter	Trace4	2.43	4.21	Automated - Genesis	119655	Echinacea purpurea	Roots		
10	4.9443487	296.66092	4.863226	4.982501	0	3827.2016	1072.803715	0.119275	INF	No Filter	Trace4	8.42	4.68	Automated - Genesis	119655	Echinacea purpurea	Roots		
11	5.0521556	303.12934	5.02504	5.065968	0	1070.50935	947.1742585	0.0409281	INF	No Filter	Trace4	2.36	4.13	Automated - Genesis	119655	Echinacea purpurea	Roots		
12	5.1458435	308.75061	5.12896	5.222679	0	3341.67525	1301.065564	0.0937196	INF	No Filter	Trace4	7.35	5.67	Automated - Genesis	119655	Echinacea purpurea	Roots		
13	5.258491	315.50946	5.232912	5.399857	93.0333176	8363.68909	1580.750618	0.1669454	INF	No Filter	Trace4	18.4	6.89	Automated - Genesis	119655	Echinacea purpurea	Roots		
1	0.8828525	52.971149	0.85834	0.914987	0	10567.3061	7126.029381	0.05664676	INF	No Filter	Trace6	86.98	70.13	Automated - Genesis	119655	Echinacea purpurea	Roots		
2	2.0838152	125.02891	2.073546	2.09408	174.076691	834.642001	1598.616459	0.0205337	INF	No Filter	Trace6	6.87	15.73	Automated - Genesis	119655	Echinacea purpurea	Roots		
3	4.3082948	258.49769	4.298063	4.318526	174.076584	747.783878	1437.06622	0.0204634	INF	No Filter	Trace6	6.15	14.14	Automated - Genesis	119655	Echinacea purpurea	Roots		
1	0.6254108	37.52465	0.61467	0.636151	163.039413	1461.15087	2674.376846	0.02148125	INF	No Filter	Trace8	26.75	28.02	Automated - Genesis	119655	Echinacea purpurea	Roots		
2	1.7667949	106.00769	1.757319	1.778987	163.039429	824.772545	1627.935371	0.0216678	INF	No Filter	Trace8	15.1	17.05	Automated - Genesis	119655	Echinacea purpurea	Roots		
3	2.5337538	152.02523	2.518964	2.547695	163.039459	1491.36803	2088.306544	0.0287302	INF	No Filter	Trace8	27.3	21.88	Automated - Genesis	119655	Echinacea purpurea	Roots		
4	3.6451957	218.71174	3.634455	3.655936	163.039474	910.033479	1665.647625	0.0214813	INF	No Filter	Trace8	16.66	17.45	Automated - Genesis	119655	Echinacea purpurea	Roots		
5	4.8182658	289.09595	4.808035	4.828499	163.039993	774.847179	1489.091023	0.020464	INF	No Filter	Trace8	14.19	15.6	Automated - Genesis	119655	Echinacea purpurea	Roots		
1	13.532592	811.95552	13.51781	13.54653	223.060501	1343.23976	1861.693407	0.028728	INF	No Filter	Trace9	100	100	Automated - Genesis	119655	Echinacea purpurea	Roots		
1	0.7588775	45.532652	0.750916	0.767936	353.089233	28386.065	54757.70993	0.0170195	7.025161013	No Filter	Trace10	2.88	30.17	Automated - Genesis	119655	Echinacea purpurea	Roots		
2	0.7810917	46.865503	0.771516	0.790151	353.089417	31672.3249	56059.47814	0.0186349	6.975379577	No Filter	Trace10	3.21	30.89	Automated - Genesis	119655	Echinacea purpurea	Roots		
3	1.5960354	95.762124	1.54983	2.737587	0	926808.469	70692.38802	1.1877569	INF	No Filter	Trace10	93.91	38.95	Automated - Genesis	119655	Echinacea purpurea	Roots	C16H18O9 chlorogenic acid (5-o-caffeoylquinic acid); C16H18O9 cis 5-o-caffeoylquinic acid; C16H18O9 4-o-caffeoylquinic acid (crytochlorogenic acid)	Level 4
1	1.5513965	93.08379	1.369343	1.594161	0	4646851.62	550441.1732	0.224817518	366.3166499	No Filter	Trace12	100	100	Manual	119655	Echinacea purpurea	Roots	C13H12O9 caftaric acid	Level 4
1	0.7885822	47.314931	0.774325	0.801689	0	11563.6523	17788.27984	0.02736458	INF	No Filter	Trace13	100	100	Automated - Genesis	119655	Echinacea purpurea	Roots		
1	10.274586	616.47516	10.25979	10.28853	301.035309	1153.59564	1523.297828	0.028733	INF	No Filter	Trace14	100	100	Automated - Genesis	119655	Echinacea purpurea	Roots		
1	5.7264769	343.58861	5.647341	6.025536	0	204673.701	24107.67271	0.3781953	INF	No Filter	Trace16	96.76	84.99	Automated - Genesis	119655	Echinacea purpurea	Roots		
2	6.0828192	364.96915	6.062578	6.128629	0	6844.42505	4257.754902	0.0660507	INF	No Filter	Trace16	3.24	15.01	Automated - Genesis	119655	Echinacea purpurea	Roots		
1	0.6791139	40.746831	0.670714	0.688615	137.023819	5229.51569	11947.96682	0.017901	INF	No Filter	Trace17	25.9	29.75	Automated - Genesis	119655	Echinacea purpurea	Roots		
2	3.1135734	186.8144	3.101107	3.125191	137.023773	946.676419	1618.602361	0.0240838	INF	No Filter	Trace17	4.69	4.03	Automated - Genesis	119655	Echinacea purpurea	Roots		
3	5.9985696	359.91418	5.990678	6.007266	137.023727	1347.36718	3234.229331	0.0165875	INF	No Filter	Trace17	6.67	8.05	Automated - Genesis	119655	Echinacea purpurea	Roots		
4	7.6368899	458.21339	7.62849	7.646391	137.023788	1249.07163	2853.725518	0.0179009	INF	No Filter	Trace17	6.19	7.1	Automated - Genesis	119655	Echinacea purpurea	Roots		
5	9.3819432	562.91659	9.373543	9.391444	137.023727	1282.07021	2929.158735	0.0179012	INF	No Filter	Trace17	6.35	7.29	Automated - Genesis	119655	Echinacea purpurea	Roots		
6	9.528098	571.68588	9.515638	9.539715	137.023727	1027.06084	1708.919484	0.0240771	INF	No Filter	Trace17	5.09	4.25	Automated - Genesis	119655	Echinacea purpurea	Roots		
7	9.5699205	574.19523	9.558303	9.581538	137.023743	2567.60428	4519.678443	0.0232348	INF	No Filter	Trace17	12.71	11.25	Automated - Genesis	119655	Echinacea purpurea	Roots		
8	10.353584	621.21504	10.34197	10.36605	137.023773	1492.73746	2483.473816	0.024079	INF	No Filter	Trace17	7.39	6.18	Automated - Genesis	119655	Echinacea purpurea	Roots		
9	10.809413	648.56478	10.7978	10.82103	137.023743	1856.65734	3268.145102	0.023234	INF	No Filter	Trace17	9.19	8.14	Automated - Genesis	119655	Echinacea purpurea	Roots		
10	12.452746	747.16476	12.44113	12.46436	137.023758	2029.99135	3573.243497	0.023235	INF	No Filter	Trace17	10.05	8.9	Automated - Genesis	119655	Echinacea purpurea	Roots		
11	13.699248	821.95488	13.68679	13.71087	137.023788	1165.1407	2029.79493	0.024071	INF	No Filter	Trace17	5.77	5.05	Automated - Genesis	119655	Echinacea purpurea	Roots		
1	1.2535802	75.214812	1.248448	1.258712	153.018631	1353.96639	3493.754149	0.0102646	26.82595958	No Filter	Trace18	100	100	Automated - ICIS	119655	Echinacea purpurea	Roots	C7H6O4 Gentisic acid (2,5-DHBA)	Level 4
1	0.8840917	53.045503	0.873792	0.89439	253.03891	1351.3853	2579.965377	0.02059836	INF	No Filter	Trace19	26.82	26.8	Automated - Genesis	119655	Echinacea purpurea	Roots		
2	0.9938734	59.632402	0.983575	1.004172	253.039154	1646.80786	3144.347376	0.02059663	INF	No Filter	Trace19	32.69	32.66	Automated - Genesis	119655	Echinacea purpurea	Roots		
3	1.0933321	65.599926	1.083033	1.103943	253.03891	979.010141	1867.790291	0.0209094	INF	No Filter	Trace19	19.43	19.4	Automated - Genesis	119655	Echinacea purpurea	Roots		
4	1.1805202	70.831212	1.17101	1.192746	253.038788	1061.07533	2035.600693	0.021736	INF	No Filter	Trace19	21.06	21.14	Automated - Genesis	119655	Echinacea purpurea	Roots		
1	4.3157513	258.94508	4.290172	4.370068	0	9969.64798	4406.02652	0.0798965	INF	No Filter	Trace22	0.51	0.96	Automated - ICIS	119655	Echinacea purpurea	Roots	C27H30O15 Kaempferol diglycosides	Level 3
2	4.6462287	278.77372	4.580642	4.789909	593.151917	1823548.65	423863.4405	0.2092667	INF	No Filter	Trace22	93.49	92.67	Automated - ICIS	119655	Echinacea purpurea	Roots	C27H30O15 Kaempferol diglycosides	Level 3
3	4.8233819	289.40291	4.815491	4.912048	593.152466	18958.3844	5978.758193	0.0965567	INF	No Filter	Trace22	0.97	1.31	Automated - ICIS	119655	Echinacea purpurea	Roots	C27H30O15 Kaempferol diglycosides	Level 3
4	4.9297351	295.78411	4.912048	4.975045	0	22907.0587	9134.688675	0.062997	INF	No Filter	Trace22	1.17	2	Automated - ICIS	119655	Echinacea purpurea	Roots	C27H30O15 Kaempferol diglycosides	Level 3
5	4.9955122	299.73073	4.975045	5.164776	593.151672	60426.3418	9684.754286	0.1897311	INF	No Filter	Trace22	3.1	2.12	Automated - ICIS	119655	Echinacea purpurea	Roots		
6	5.1750277	310.50166	5.164776	5.282106	0	14781.1895	4332.376631	0.1173299	INF	No Filter	Trace22	0.76	0.95	Automated - ICIS	119655	Echinacea purpurea	Roots		
1	3.5540884	213.2453	3.543348	3.564829	447.093201	1100.296	2013.897415	0.0214812	INF	No Filter	Trace24	1.74	9.7	Automated - Genesis	119655	Echinacea purpurea	Roots	C21H20O11 kaempferol-O-glucoside	Level 3
2	3.5898906	215.39344	3.57915	3.600631	447.093445	1028.11503	1881.789818	0.0214813	INF	No Filter	Trace24	1.63	9.06	Automated - Genesis	119655	Echinacea purpurea	Roots	C21H20O11 kaempferol-O-glucoside	Level 3
3	4.9246194	295.47716	4.854078	5.027815	0	59488.5569	13813.35692	0.1737368	INF	No Filter	Trace24	94.12	66.52	Automated - Genesis	119655	Echinacea purpurea	Roots	C21H20O11 kaempferol-O-glucoside	Level 3
4	5.0380477	302.28286	5.027815	5.04828	447.092651	817.893485	1571.631826	0.0204651	INF	No Filter	Trace24	1.29	7.57	Automated - Genesis	119655	Echinacea purpurea	Roots		
5	5.0636268	303.81761	5.053395	5.07386	447.092865	772.248613	1484.112995	0.0204642	INF	No Filter	Trace24	1.22	7.15	Automated - Genesis	119655	Echinacea purpurea	Roots		

1	0.8761325	52.567947	0.842893	0.904689	0	22607.2557	10836.64277	0.06179585	INF	No Filter	Trace25	87.6	68.56	Automated - Genesis	119655	Echinacea purpurea	Roots	i9G 2-hydroxyacryloyl-N-glutamate C8H11NO6	Level 4
2	0.9201362	55.208174	0.909838	0.930439	216.051239	1210.53016	2310.406435	0.02060182	INF	No Filter	Trace25	4.69	14.62	Automated - Genesis	119655	Echinacea purpurea	Roots		
3	13.935607	836.13642	13.92167	13.95041	216.052383	1989.31036	2659.420686	0.028743	INF	No Filter	Trace25	7.71	16.82	Automated - Genesis	119655	Echinacea purpurea	Roots		
1	5.5907962	335.44777	5.499664	6.409122	0	7877630.57	775419.2384	0.9094577	156.5192895	No Filter	Trace26	100	100	Automated - Genesis	119655	Echinacea purpurea	Roots	C10H10O4 Ferulic acid	Level 4
1	2.1265003	127.59002	2.099212	2.136765	385.115479	4039.58241	3473.335481	0.0375528	INF	No Filter	Trace27	0.87	3.51	Automated - ICIS	119655	Echinacea purpurea	Roots	C17H22O10 Sinapoyl-glucose	Level 3
2	2.5050233	150.3014	2.458554	2.625215	385.113922	107265.341	26652.64334	0.166661	INF	No Filter	Trace27	22.98	26.9	Automated - ICIS	119655	Echinacea purpurea	Roots	C17H22O10 Sinapoyl-glucose	Level 3
3	2.977115	178.6269	2.913532	3.021261	0	19097.8268	5506.337254	0.1077294	INF	No Filter	Trace27	4.09	5.56	Automated - ICIS	119655	Echinacea purpurea	Roots		
4	3.1182202	187.09321	3.09646	3.157719	0	9022.53213	6223.591232	0.0612594	INF	No Filter	Trace27	1.93	6.28	Automated - ICIS	119655	Echinacea purpurea	Roots		
5	3.6654375	219.92625	3.592231	3.814944	0	208682.618	36018.7091	0.2227128	INF	No Filter	Trace27	44.7	36.35	Automated - ICIS	119655	Echinacea purpurea	Roots	C17H22O10 Sinapoyl-glucose	Level 3
6	3.8728245	232.36947	3.814944	4.020462	0	118705.004	21217.02141	0.2055186	INF	No Filter	Trace27	25.43	21.41	Automated - ICIS	119655	Echinacea purpurea	Roots	C17H22O10 Sinapoyl-glucose	Level 3
1	1.291128	77.46768	1.263844	1.327056	339.072052	14570.5229	6345.00353	0.063212	INF	No Filter	Trace28	2.08	10.86	Automated - Genesis	119655	Echinacea purpurea	Roots	C15H16O9 z sinapoyl-malate	Level 4
2	6.1345499	368.07299	5.995794	6.652566	0	651237.69	34351.51861	0.6567721	INF	No Filter	Trace28	92.9	58.81	Automated - Genesis	119655	Echinacea purpurea	Roots	C15H16O9 E-Sinapoyl-malate	Level 4
3	6.73059	403.8354	6.653806	6.744911	339.072968	13505.3433	5339.821568	0.0911047	INF	No Filter	Trace28	1.93	9.14	Automated - Genesis	119655	Echinacea purpurea	Roots		
4	6.8325141	409.95085	6.818193	6.859174	339.072113	4876.87559	4612.073113	0.040981	INF	No Filter	Trace28	0.7	7.9	Automated - Genesis	119655	Echinacea purpurea	Roots		
5	6.9044768	414.26861	6.891395	6.982498	0	11311.7251	4155.212396	0.0911026	INF	No Filter	Trace28	1.61	7.11	Automated - Genesis	119655	Echinacea purpurea	Roots		
6	7.0401559	422.40935	7.027583	7.088976	0	5528.75436	3604.112926	0.061393	INF	No Filter	Trace28	0.79	6.17	Automated - Genesis	119655	Echinacea purpurea	Roots		
1	0.7743249	46.459491	0.766367	0.80045	335.078522	17765.1297	25342.07738	0.03408258	INF	No Filter	Trace29	47.54	50.36	Automated - Genesis	119655	Echinacea purpurea	Roots		
2	0.8325938	49.95563	0.824627	0.841654	335.078674	4979.05107	11851.42669	0.0170267	INF	No Filter	Trace29	13.32	23.55	Automated - Genesis	119655	Echinacea purpurea	Roots		
3	0.9070295	54.421772	0.896731	0.918896	0	3508.4656	5615.143108	0.02216577	INF	No Filter	Trace29	9.39	11.16	Automated - Genesis	119655	Echinacea purpurea	Roots		
4	2.3675558	142.05335	2.359633	2.376601	335.078156	1099.31053	2622.814443	0.0169688	INF	No Filter	Trace29	2.94	5.21	Automated - Genesis	119655	Echinacea purpurea	Roots		
5	2.66239	159.7434	2.60895	2.685625	335.077881	10018.5956	4888.20179	0.0766743	INF	No Filter	Trace29	26.81	9.71	Automated - Genesis	119655	Echinacea purpurea	Roots	C16H16O8 caffeoyl-shikimate	Level 4
1	8.6764641	520.58785	8.665724	8.687205	510.077942	917.26041	1678.867715	0.0214813	INF	No Filter	Trace30	41.95	48.4	Automated - Genesis	119655	Echinacea purpurea	Roots		
2	12.992262	779.53572	12.97832	13.0062	510.081055	1269.48996	1790.156483	0.027881	INF	No Filter	Trace30	58.05	51.6	Automated - Genesis	119655	Echinacea purpurea	Roots		
1	1.4943357	89.660142	1.484058	1.5046	137.023773	598.308657	1144.510825	0.020542	INF	No Filter	Trace1	4.05	4.73	Automated - Genesis	119656	Echinacea purpurea	Stems		
2	1.6427128	98.562768	1.633227	1.653291	137.023895	901.620683	1787.259705	0.0200641	INF	No Filter	Trace1	6.1	7.38	Automated - Genesis	119656	Echinacea purpurea	Stems		
3	3.5588063	213.52838	3.548065	3.569547	137.023758	2050.03639	3752.20946	0.0214813	INF	No Filter	Trace1	13.88	15.5	Automated - Genesis	119656	Echinacea purpurea	Stems		
4	4.2452545	254.71527	4.235021	4.255487	137.02478	243.886884	468.8761985	0.0204663	INF	No Filter	Trace1	1.65	1.94	Automated - Genesis	119656	Echinacea purpurea	Stems		
5	4.64748	278.8488	4.637247	4.657715	137.023727	937.527565	1800.97491	0.0204682	INF	No Filter	Trace1	6.35	7.44	Automated - Genesis	119656	Echinacea purpurea	Stems		
6	7.3846808	443.08085	7.374447	7.394912	137.023727	1132.22583	2175.651825	0.0204647	INF	No Filter	Trace1	7.66	8.99	Automated - Genesis	119656	Echinacea purpurea	Stems		
7	8.350709	501.04254	8.339968	8.36306	137.023727	1958.60333	3304.559807	0.0230917	INF	No Filter	Trace1	13.26	13.65	Automated - Genesis	119656	Echinacea purpurea	Stems		
8	10.390942	623.45652	10.377	10.40488	137.023804	1556.63531	2195.066516	0.027882	INF	No Filter	Trace1	10.54	9.07	Automated - Genesis	119656	Echinacea purpurea	Stems		
9	11.681614	700.89684	11.66767	11.6964	137.023849	1042.50476	1444.882952	0.028728	INF	No Filter	Trace1	7.06	5.97	Automated - Genesis	119656	Echinacea purpurea	Stems		
10	11.834334	710.06004	11.82039	11.84827	136.016785	962.567453	1357.319006	0.027882	INF	No Filter	Trace1	6.52	5.61	Automated - Genesis	119656	Echinacea purpurea	Stems		
11	12.205683	732.34098	12.19174	12.21962	119.014282	1077.07328	1518.854271	0.027882	INF	No Filter	Trace1	7.29	6.27	Automated - Genesis	119656	Echinacea purpurea	Stems		
12	13.29478	797.6868	13.28084	13.30872	137.023819	1382.15846	1949.034657	0.027881	INF	No Filter	Trace1	9.36	8.05	Automated - Genesis	119656	Echinacea purpurea	Stems		
13	13.71028	822.6168	13.69634	13.72422	119.01387	929.245464	1310.364668	0.027881	INF	No Filter	Trace1	6.29	5.41	Automated - Genesis	119656	Echinacea purpurea	Stems		
1	4.9955089	299.73053	4.985277	5.005742	77.0399323	222.121856	426.381064	0.0204648	INF	No Filter	Trace2	46.78	47.97	Automated - Genesis	119656	Echinacea purpurea	Stems		
2	8.050999	483.05994	8.040259	8.06174	103.055229	252.709477	462.5378525	0.0214811	INF	No Filter	Trace2	53.22	52.03	Automated - Genesis	119656	Echinacea purpurea	Stems		
1	0.6241768	37.450609	0.609856	0.691502	0	63908.8545	25130.36008	0.08164621	8.493737528	No Filter	Trace3	7.62	15.21	Automated - ICIS	119656	Echinacea purpurea	Stems		
2	0.9122075	54.732447	0.868673	0.970869	137.023102	45775.0296	22037.50109	0.102196781	10.03704727	No Filter	Trace3	5.46	13.34	Manual	119656	Echinacea purpurea	Stems	C13H16O8 SA 2-O-glucoside	Level 2
3	1.2456456	74.738736	1.195702	1.359217	137.023285	81668.1659	17302.3764	0.16351485	7.933695921	No Filter	Trace3	9.73	10.47	Manual	119656	Echinacea purpurea	Stems		
4	1.5303507	91.821042	1.461414	1.655588	137.023117	106591.479	25644.0146	0.194173884	11.55034168	No Filter	Trace3	12.71	15.52	Manual	119656	Echinacea purpurea	Stems	C13H16O8 SA 5-O-glucoside	Level 2
5	1.8092581	108.55549	1.706774	1.962279	137.023209	541018.221	75068.62965	0.255505091	32.9037374	No Filter	Trace3	64.49	45.45	Manual	119656	Echinacea purpurea	Stems		
1	0.9150158	54.900949	0.891595	0.949885	0	10773.1358	6257.303153	0.05829053	INF	No Filter	Trace4	16.47	20.03	Automated - Genesis	119656	Echinacea purpurea	Stems		
2	1.5331467	91.988802	1.494336	1.608107	0	33398.7633	8399.076471	0.113771	INF	No Filter	Trace4	51.07	26.89	Automated - Genesis	119656	Echinacea purpurea	Stems		
3	1.6262143	97.572858	1.612079	1.638359	0	1076.3735	1455.280426	0.0262809	INF	No Filter	Trace4	1.65	4.66	Automated - Genesis	119656	Echinacea purpurea	Stems		
4	4.0231647	241.38988	3.974343	4.03734	0	1734.42522	1003.680766	0.0629968	INF	No Filter	Trace4	2.65	3.21	Automated - Genesis	119656	Echinacea purpurea	Stems		
5	4.1822327	250.93396	4.156652	4.194075	93.0333252	802.611327	960.881315	0.0374229	INF	No Filter	Trace4	1.23	3.08	Automated - Genesis	119656	Echinacea purpurea	Stems		
6	4.383032	262.98192	4.366148	4.40678	0	1305.68906	1009.497674	0.0406321	INF	No Filter	Trace4	2	3.23	Automated - Genesis	119656	Echinacea purpurea	Stems		
7	4.439002	266.34012	4.409121	4.482419	0	2305.21098	1548.111921	0.0732976	INF	No Filter	Trace4	3.52	4.96	Automated - Genesis	119656	Echinacea purpurea	Stems		
8	4.5389627	272.33776	4.51106	4.552044	0	967.31273	970.1569423	0.040984	INF	No Filter	Trace4	1.48	3.11	Automated - Genesis	119656	Echinacea purpurea	Stems		
9	4.6730635	280.38381	4.619123	4.738415	0	3424.87793	1110.544947	0.1192912	INF	No Filter	Trace4	5.24	3.56	Automated - Genesis	119656	Echinacea purpurea	Stems		
10	4.7565374	285.39224	4.738415	4.769111	0	1079.84883	1169.778571	0.0306964	INF	No Filter	Trace4	1.65	3.75	Automated - Genesis	119656	Echinacea purpurea	Stems		
11	4.8297634	289.7858	4.816752	4.842353	0	745.785699	1105.406201	0.025601	INF	No Filter	Trace4	1.14	3.54	Automated - Genesis	119656	Echinacea purpurea	Stems		
12	4.8553598	291.32159	4.842353	4.869544	0	752.312902	998.4089848	0.0271915	INF	No Filter	Trace4	1.15	3.2	Automated - Genesis	119656	Echinacea purpurea	Stems		
13	4.8797766	292.7866	4.869544	4.890009	93.0331192	454.687595	874.1365457	0.0204651	INF	No Filter	Trace4	0.7	2.8	Automated - Genesis	119656	Echinacea purpurea	Stems		
14	4.9375912	296.25547	4.91559	4.953043	0	1087.1913	1015.04684	0.0374532	INF	No Filter	Trace4	1.66	3.25	Automated - Genesis	119656	Echinacea purpurea	Stems		
15	5.0723176	304.33906	5.046736	5.087665	93.0329971	1206.45429	906.4379277	0.0409292	INF	No Filter	Trace4	1.84	2.9	Automated - Genesis	119656	Echinacea purpurea	Stems		
16	5.2100767	312.6046	5.18648	5.244356	0	1548.83591	1117.301337	0.0578765	INF	No Filter	Trace4	2.37	3.58	Automated - Genesis	119656	Echinacea purpurea	Stems		
17	5.3482976	320.89786	5.327833	5.411047	93.0333252	2735.28323	1333.671985	0.0832137	INF	No Filter	Trace4	4.18	4.27	Automated - Genesis	119656	Echinacea purpurea	Stems		
1	2.6472474	158.83484	2.595286	2.734057	225.039978	13175.6642	4589.542652	0.1387706	INF	No Filter	Trace5	34.26	30.54	Automated - ICIS	119656	Echinacea purpurea	Stems	Chorismate C10H10O6	Level 4
2	3.2913814	197.48288	3.251637	3.369404	0	23061.9204	6640.433594	0.117767	INF	No Filter	Trace5	59.96	44.19	Automated - ICIS	119656	Echinacea purpurea	Stems	Prephenate C10H10O6	Level 4
3	3.3908855	203.45313	3.380145	3.401626	225.040131	1190.51066	2179.027975	0.0214812	INF	No Filter	Trace5	3.1	14.5	Automated - ICIS	119656	Echinacea purpurea	Stems		
4	3.5213789	211.28273	3.510638	3.533745	225.041	1032.37457	1617.207559	0.0231063	INF	No Filter	Trace5	2.68	10.76	Automated - ICIS	119656	Echinacea purpurea	Stems		
1	0.8915946	53.495674	0.863507	0.920165	0	5723.14452	4812.0031	0.05665874	INF	No Filter	Trace6	100	100	Automated - Genesis	119656	Echinacea purpurea	Stems		
1	2.6193656	157.16194	2.350365	2.840876	179.034134	735855.312	128601.6382	0.490510949	INF	No Filter	Trace7	100	100	Manual	119656	Echinacea purpurea	Stems		
1	0.9578428	57.470567	0.947544	0.968141	223.061966	1177.53057	2248.211999	0.02059675	INF	No Filter	Trace9	11.23	24.02	Automated - Genesis	119656	Echinacea purpurea	Stems		
2	4.4800781	268.80469	4.469337	4.490819	223.060532	854.572873	1564.137907	0.0214813	INF	No Filter	Trace9	8.15	16.71	Automated - Genesis	119656	Echinacea purpurea	Stems		
3	6.1464773	368.78864	6.099569	6.169198	0	7502.38545	3801.838516	0.0696288	INF	No Filter	Trace9	71.54	40.63	Automated - Genesis	119656	Echinacea purpurea	Stems	C11H12O5 E-Sinapic acid	Level 4
4	6.217343	373.04058	6.206602	6.228084	223.061462	952.867239	1744.053575	0.0214813	INF	No Filter	Trace9	9.09	18.64	Automated - Genesis	119656	Echinacea purpurea	Stems		
1	0.8120086	48.720517	0.804051	0.821068	353.089325	35193.5578	67888.69372	0.017017	5.626436739	No Filter	Trace10	1.78	19.16	Automated - Genesis	119656	Echinacea purpurea	Stems		
2	1.1671805	70.03083	1.115905	1.492784	353.087952	390057.723	64144.14215	0.3768785	INF	No Filter	Trace10	19.78	18.1	Manual	119656	Echinacea purpurea	Stems	C16H18O9 neochlorogenic acid (3-o-caffeoylquinic acid)	Level 4
3	1.5947372	95.684232	1.532847	1.665693	353.088074	437701.855	124205.4843	0.132846715	INF	No Filter	Trace10	22.2	35.05	Manual	119656	Echinacea purpurea	Stems	C16H18O9 chlorogenic acid (5-o-caffeoylquinic acid)	Level 4

4	1.7102393	102.61436	1.675912	1.982482	353.08783	592963.094	67974.12538	0.306569343	INF	No Filter	Trace10	30.07	19.18	Manual	119656	Echinacea purpurea	Stems	C16H18O9 cis 5-o-caffeoylquinic acid	Level 4
5	2.4263073	145.57844	2.268613	2.922628	353.087708	515754.285	30105.57791	0.654014599	INF	No Filter	Trace10	26.16	8.5	Manual	119656	Echinacea purpurea	Stems	C16H18O9 4-o-caffeoylquinic acid (crytochlorogenic acid)	Level 4
1	4.6781791	280.69075	4.629355	4.792708	515.119995	278398.387	48866.75547	0.163353	INF	No Filter	Trace11	21.79	21.79	Manual	119656	Echinacea purpurea	Stems	C25H24O12 3.4-dicaffeolquinic acid	Level 4
2	4.8723202	292.33921	4.813171	5.105792	515.119934	526958.345	66089.71281	0.2926208	INF	No Filter	Trace11	41.25	29.46	Manual	119656	Echinacea purpurea	Stems	C25H24O12 3,5-dicaffeolquinic acid	Level 4
3	5.1790233	310.7414	5.157318	5.236896	515.120972	25126.4868	8913.711096	0.0795779	INF	No Filter	Trace11	1.97	3.97	Automated - ICIS	119656	Echinacea purpurea	Stems		
4	5.2676116	316.0567	5.236896	5.330608	515.119812	16697.4539	5661.903645	0.0937122	INF	No Filter	Trace11	1.31	2.52	Automated - ICIS	119656	Echinacea purpurea	Stems		
5	5.3510724	321.06434	5.330608	5.35619	515.121643	6546.08061	7768.475577	0.0255812	INF	No Filter	Trace11	0.51	3.46	Automated - ICIS	119656	Echinacea purpurea	Stems		
6	5.4146272	324.87763	5.35619	5.648559	515.120239	347364.602	51931.35089	0.2923693	INF	No Filter	Trace11	27.19	23.15	Automated - ICIS	119656	Echinacea purpurea	Stems	C25H24O12 4,5-dicaffeolquinic acid	Level 4
7	5.7026203	342.15722	5.66646	5.725341	0	17439.4415	6624.974759	0.058881	INF	No Filter	Trace11	1.37	2.95	Automated - ICIS	119656	Echinacea purpurea	Stems		
8	5.7539826	345.23896	5.725341	5.770644	515.120239	10977.8009	6481.290754	0.045303	INF	No Filter	Trace11	0.86	2.89	Automated - ICIS	119656	Echinacea purpurea	Stems		
9	5.7937293	347.62376	5.770644	5.829246	0	13056.4113	7172.927705	0.0586025	INF	No Filter	Trace11	1.02	3.2	Automated - ICIS	119656	Echinacea purpurea	Stems		
10	5.8497102	350.98261	5.829246	5.885892	515.120056	10911.1319	4794.418327	0.0566456	INF	No Filter	Trace11	0.85	2.14	Automated - ICIS	119656	Echinacea purpurea	Stems		
11	5.9859475	359.15685	5.915055	5.998521	515.1203	13547.3381	4117.948166	0.0834659	INF	No Filter	Trace11	1.06	1.84	Automated - ICIS	119656	Echinacea purpurea	Stems		
12	6.0191964	361.15178	5.998521	6.069688	515.11969	10511.7717	5877.420261	0.0711674	INF	No Filter	Trace11	0.82	2.62	Automated - ICIS	119656	Echinacea purpurea	Stems		
1	1.5030481	90.182886	1.369343	1.594161	0	1615186.79	176380.489	0.224817518	81.6049583	No Filter	Trace12	100	100	Manual	119656	Echinacea purpurea	Stems	C13H12O9 caftaric acid	Level 4
1	0.6468861	38.813164	0.607504	0.661207	473.072784	25906.2305	16448.64064	0.05370318	INF	No Filter	Trace13	75.73	65.27	Automated - Genesis	119656	Echinacea purpurea	Stems	C22H18O12 cichoric acid	Level 4
2	0.6950745	41.704471	0.671947	0.706147	473.074738	8301.8624	8750.759588	0.03419959	INF	No Filter	Trace13	24.27	34.73	Automated - Genesis	119656	Echinacea purpurea	Stems		
1	12.045984	722.75904	12.03204	12.05993	269.046783	1114.2923	1571.312756	0.027882	INF	No Filter	Trace16	100	100	Automated - Genesis	119656	Echinacea purpurea	Stems		
1	1.4943357	89.660142	1.485298	1.503048	137.023773	597.220257	1428.655519	0.0177505	INF	No Filter	Trace17	5.07	5.72	Automated - Genesis	119656	Echinacea purpurea	Stems		
2	1.6427128	98.562768	1.634466	1.65095	137.023895	906.929527	2230.977973	0.0164838	INF	No Filter	Trace17	7.7	8.93	Automated - Genesis	119656	Echinacea purpurea	Stems		
3	3.5588063	213.52838	3.550406	3.568307	137.023758	2050.03865	4683.760637	0.0179011	INF	No Filter	Trace17	17.4	18.74	Automated - Genesis	119656	Echinacea purpurea	Stems		
4	4.2452545	254.71527	4.236558	4.253147	137.02478	244.183746	585.2828596	0.0165884	INF	No Filter	Trace17	2.07	2.34	Automated - Genesis	119656	Echinacea purpurea	Stems		
5	4.64748	278.8488	4.639587	4.656475	137.023727	937.986423	2248.098216	0.0168878	INF	No Filter	Trace17	7.96	8.99	Automated - Genesis	119656	Echinacea purpurea	Stems		
6	7.3846808	443.08085	7.376788	7.393673	137.023727	1132.75034	2715.795183	0.0168848	INF	No Filter	Trace17	9.62	10.87	Automated - Genesis	119656	Echinacea purpurea	Stems		
7	8.350709	501.04254	8.342309	8.361821	137.023727	1936.63031	4124.974182	0.0195118	INF	No Filter	Trace17	16.44	16.5	Automated - Genesis	119656	Echinacea purpurea	Stems		
8	10.390942	623.45652	10.37932	10.40256	137.023804	1556.63531	2740.029909	0.023235	INF	No Filter	Trace17	13.21	10.96	Automated - Genesis	119656	Echinacea purpurea	Stems		
9	11.681614	700.89684	11.67	11.69408	137.023849	1035.41802	1803.600243	0.024081	INF	No Filter	Trace17	8.79	7.22	Automated - Genesis	119656	Echinacea purpurea	Stems		
10	13.29478	797.6868	13.28316	13.3064	137.023819	1382.16139	2432.916367	0.023235	INF	No Filter	Trace17	11.73	9.73	Automated - Genesis	119656	Echinacea purpurea	Stems		
1	1.1872847	71.237082	1.180583	1.193989	153.018677	1279.57266	3159.931806	0.0134061	31.54891152	No Filter	Trace18	22.95	22.95	Automated - ICIS	119656	Echinacea purpurea	Stems		
2	1.5007075	90.04245	1.494336	1.50818	153.018616	1548.76595	3745.435989	0.0138444	37.39461352	No Filter	Trace18	27.78	27.2	Automated - ICIS	119656	Echinacea purpurea	Stems		
3	2.216603	132.99618	2.21147	2.221735	153.018661	826.260904	2130.020309	0.0102649	21.26622547	No Filter	Trace18	14.82	15.47	Automated - ICIS	119656	Echinacea purpurea	Stems	C7H6O4 2,3-DHBA	Level 4
4	3.6642383	219.8543	3.659418	3.670159	153.018539	1063.91791	2622.249745	0.0107406	26.1806679	No Filter	Trace18	19.08	19.04	Automated - ICIS	119656	Echinacea purpurea	Stems		
5	3.7855971	227.13583	3.780777	3.791518	153.01857	857.223368	2112.1271	0.0107408	21.08757881	No Filter	Trace18	15.37	15.34	Automated - ICIS	119656	Echinacea purpurea	Stems		
1	4.3314916	259.8895	4.284208	4.417521	593.151794	72526.0674	19843.08581	0.1333133	211.8002531	No Filter	Trace22	15.92	18.71	Automated - ICIS	119656	Echinacea purpurea	Stems	C27H30O15 Kaempferol diglycosides	Level 3
2	4.6372467	278.2348	4.578345	4.799063	593.1521	350962.158	77585.54643	0.2207176	828.1291796	No Filter	Trace22	77.05	73.15	Automated - ICIS	119656	Echinacea purpurea	Stems	C27H30O15 Kaempferol diglycosides	Level 3
3	4.9132494	294.79496	4.865964	4.983741	593.151489	32027.6389	8636.753145	0.1177766	92.18659436	No Filter	Trace22	7.03	8.14	Automated - ICIS	119656	Echinacea purpurea	Stems	C27H30O15 Kaempferol diglycosides	Level 3
1	4.8387725	290.32635	4.824647	4.850243	0	2218.21511	3251.563892	0.0255959	INF	No Filter	Trace23	3.59	15.84	Automated - Genesis	119656	Echinacea purpurea	Stems		
2	5.0392795	302.35677	5.029047	5.049512	563.141357	1099.6206	2113.216071	0.0204649	INF	No Filter	Trace23	1.78	10.3	Automated - Genesis	119656	Echinacea purpurea	Stems		
3	5.0943162	305.65897	5.049512	5.195968	0	57323.2428	13036.53714	0.1464559	INF	No Filter	Trace23	92.84	63.51	Automated - Genesis	119656	Echinacea purpurea	Stems	C26H28O14 apigenin di-glycoside family	Level 3
4	5.3306084	319.8365	5.320377	5.340841	563.141541	1105.3338	2124.003013	0.0204646	INF	No Filter	Trace23	1.79	10.35	Automated - Genesis	119656	Echinacea purpurea	Stems		
1	4.9171255	295.02753	4.887669	4.928597	0	3312.96155	3350.792411	0.0409287	INF	No Filter	Trace24	100	100	Automated - Genesis	119656	Echinacea purpurea	Stems	C21H20O11 kaempferol-O-glucoside	Level 3
1	0.867416	52.044961	0.786261	0.968141	0	65535.5335	15588.58122	0.18188047	INF	No Filter	Trace25	98.46	88.83	Automated - Genesis	119656	Echinacea purpurea	Stems	i9G 2-hydroxyacryloyl-N-glutamate C8H11NO6	Level 4
2	0.9990543	59.943256	0.988755	1.009352	216.051208	1026.89638	1960.655942	0.02059733	INF	No Filter	Trace25	1.54	11.17	Automated - Genesis	119656	Echinacea purpurea	Stems		
1	5.5955976	335.73586	5.491417	6.665809	0	36611702.8	3286660.851	1.1743915	434.4689694	No Filter	Trace26	100	100	Automated - Genesis	119656	Echinacea purpurea	Stems	C10H10O4 Ferulic acid	Level 4
1	2.4077194	144.46316	2.315743	2.45736	385.1138	29221.9909	10726.8901	0.1416173	INF	No Filter	Trace27	6.65	12.03	Automated - ICIS	119656	Echinacea purpurea	Stems	C17H22O10 Sinapoyl-glucose	Level 3
2	2.5015062	150.09037	2.462007	2.530229	385.113983	6779.72682	4096.938004	0.0682218	INF	No Filter	Trace27	1.54	4.6	Automated - ICIS	119656	Echinacea purpurea	Stems	C17H22O10 Sinapoyl-glucose	Level 3
3	2.937895	176.2737	2.893749	2.989859	385.113953	18937.7758	7445.326964	0.0961095	INF	No Filter	Trace27	4.31	8.35	Automated - ICIS	119656	Echinacea purpurea	Stems	C17H22O10 Sinapoyl-glucose	Level 3
4	3.1402604	188.41562	3.099912	3.199191	385.112396	21163.9492	6679.907375	0.0992786	INF	No Filter	Trace27	4.81	7.49	Automated - ICIS	119656	Echinacea purpurea	Stems		
5	3.6558381	219.35029	3.580287	3.817611	0	202244.233	32135.34067	0.2373233	INF	No Filter	Trace27	46	36.05	Automated - ICIS	119656	Echinacea purpurea	Stems	C17H22O10 Sinapoyl-glucose	Level 3
6	3.8755247	232.53148	3.817611	4.029884	0	161335.403	28057.64195	0.212273	INF	No Filter	Trace27	36.69	31.48	Automated - ICIS	119656	Echinacea purpurea	Stems	C17H22O10 Sinapoyl-glucose	Level 3
1	1.2765127	76.590762	1.238956	1.478926	0	136501.159	29077.1462	0.2399695	INF	No Filter	Trace28	3.59	14.6	Automated - Genesis	119656	Echinacea purpurea	Stems	C15H16O9 z sinapoyl-malate	Level 4
2	6.1607981	369.64789	5.991064	7.050785	0	3670972.07	170136.9001	1.0597211	31.49776177	No Filter	Trace28	96.41	85.4	Automated - Genesis	119656	Echinacea purpurea	Stems	C15H16O9 E-Sinapoyl-malate	Level 4
1	0.7045758	42.274547	0.693835	0.716445	0	12484.0458	19611.04374	0.0226102	INF	No Filter	Trace29	16.67	11.66	Automated - Genesis	119656	Echinacea purpurea	Stems		
2	0.753734	45.22404	0.745771	0.762794	335.078766	62405.4414	148601.8117	0.01702293	INF	No Filter	Trace29	83.33	88.34	Automated - Genesis	119656	Echinacea purpurea	Stems		
1	1.1044035	66.26421	1.094105	1.114874	137.023972	446.627944	847.0420998	0.020769	INF	No Filter	Trace1	7.23	8.4	Automated - Genesis	119657	Echinacea purpurea	Leaves		
2	3.9976195	239.85717	3.987388	4.007853	137.023788	1388.60015	2668.230816	0.0204647	INF	No Filter	Trace1	22.49	26.46	Automated - Genesis	119657	Echinacea purpurea	Leaves		
3	4.1822634	250.9358	4.172032	4.194107	137.023773	272.064825	520.4606487	0.0220744	INF	No Filter	Trace1	4.41	5.16	Automated - Genesis	119657	Echinacea purpurea	Leaves		
4	4.5193919	271.16351	4.508651	4.531733	137.023743	827.476381	1396.772418	0.0230814	INF	No Filter	Trace1	13.4	13.85	Automated - Genesis	119657	Echinacea purpurea	Leaves		
5	8.37963	502.7778	8.368889	8.390371	65.0389404	198.468546	363.2600398	0.0214813	INF	No Filter	Trace1	3.21	3.6	Automated - Genesis	119657	Echinacea purpurea	Leaves		
6	9.623354	577.40124	9.609413	9.637295	137.023743	1020.46169	1438.99187	0.0278816	INF	No Filter	Trace1	16.53	14.27	Automated - Genesis	119657	Echinacea purpurea	Leaves		
7	11.292345	677.5407	11.2784	11.30629	119.013962	817.404379	1152.691646	0.027882	INF	No Filter	Trace1	13.24	11.43	Automated - Genesis	119657	Echinacea purpurea	Leaves		
8	11.548175	692.8905	11.53423	11.56212	137.023727	1203.82877	1697.561524	0.027882	INF	No Filter	Trace1	19.5	16.83	Automated - Genesis	119657	Echinacea purpurea	Leaves		
1	8.1361854	488.17112	8.125444	8.146926	103.055214	808.60521	1480.013178	0.0214813	INF	No Filter	Trace2	44.07	49.16	Automated - Genesis	119657	Echinacea purpurea	Leaves		
2	8.37963	502.7778	8.368889	8.390371	65.0389404	198.468546	363.2600398	0.0214813	INF	No Filter	Trace2	10.82	12.07	Automated - Genesis	119657	Echinacea purpurea	Leaves		
3	11.301638	678.09828	11.2877	11.31558	103.054924	827.820896	1167.329312	0.027881	INF	No Filter	Trace2	45.12	38.77	Automated - Genesis	119657	Echinacea purpurea	Leaves		

1	1.8293315	109.75989	1.706774	1.962279	137.023331	189814.296	24134.32965	0.255505091	9.619393996	No Filter	Trace3	19.84	26.4	Manual	119657	Echinacea purpurea	Leaves		
2	5.654369	339.26214	5.528467	5.988321	0	766805.516	67300.70347	0.459854015	26.2890072	No Filter	Trace3	80.16	73.6	Manual	119657	Echinacea purpurea	Leaves		
1	0.9098914	54.593484	0.896785	0.922535	0	818.391241	1193.052157	0.0257506	INF	No Filter	Trace4	2.86	4.2	Automated - Genesis	119657	Echinacea purpurea	Leaves		
2	3.8731514	232.38908	3.860144	3.885724	0	685.817717	1007.628946	0.0255801	INF	No Filter	Trace4	2.39	3.55	Automated - Genesis	119657	Echinacea purpurea	Leaves		
3	3.9384201	236.30521	3.895956	3.953913	0	1257.76779	921.5187979	0.0579574	INF	No Filter	Trace4	4.39	3.24	Automated - Genesis	119657	Echinacea purpurea	Leaves		
4	3.979495	238.7697	3.969262	3.989729	93.0333176	390.515975	750.7463041	0.0204664	INF	No Filter	Trace4	1.36	2.64	Automated - Genesis	119657	Echinacea purpurea	Leaves		
5	4.0629812	243.77887	4.052749	4.073214	93.0331039	322.472649	619.9450793	0.0204651	INF	No Filter	Trace4	1.13	2.18	Automated - Genesis	119657	Echinacea purpurea	Leaves		
6	4.1236459	247.41875	4.110639	4.136219	0	671.762201	988.7064465	0.0255806	INF	No Filter	Trace4	2.35	3.48	Automated - Genesis	119657	Echinacea purpurea	Leaves		
7	4.151568	249.09408	4.141335	4.187383	93.0328674	1069.3846	791.3551444	0.0460473	INF	No Filter	Trace4	3.73	2.79	Automated - Genesis	119657	Echinacea purpurea	Leaves		
8	4.2416864	254.50118	4.229919	4.2555	0	659.205103	983.752374	0.0255806	INF	No Filter	Trace4	2.3	3.46	Automated - Genesis	119657	Echinacea purpurea	Leaves		
9	4.285455	257.1273	4.270848	4.298028	0	757.127153	1093.602505	0.02718	INF	No Filter	Trace4	2.64	3.85	Automated - Genesis	119657	Echinacea purpurea	Leaves		
10	4.3404979	260.42987	4.313376	4.35431	0	947.979416	973.169807	0.0409334	INF	No Filter	Trace4	3.31	3.43	Automated - Genesis	119657	Echinacea purpurea	Leaves		
11	4.4091417	264.5485	4.398401	4.419882	93.0333023	282.714636	517.4555462	0.0214812	INF	No Filter	Trace4	0.99	1.82	Automated - Genesis	119657	Echinacea purpurea	Leaves		
12	4.4501284	267.0077	4.437783	4.460869	93.0330582	407.150964	732.4997119	0.0230854	INF	No Filter	Trace4	1.42	2.58	Automated - Genesis	119657	Echinacea purpurea	Leaves		
13	4.4800097	268.80058	4.468029	4.534074	0	2006.99628	1092.905772	0.0660443	INF	No Filter	Trace4	7.01	3.85	Automated - Genesis	119657	Echinacea purpurea	Leaves		
14	4.5841966	275.0518	4.562715	4.613929	93.0330734	1202.08514	802.8992056	0.0512133	INF	No Filter	Trace4	4.2	2.83	Automated - Genesis	119657	Echinacea purpurea	Leaves		
15	4.6395091	278.37055	4.619044	4.649741	93.0327148	574.202593	554.1063938	0.0306964	INF	No Filter	Trace4	2.01	1.95	Automated - Genesis	119657	Echinacea purpurea	Leaves		
16	4.6989249	281.93549	4.649741	4.712738	0	1747.74758	925.0954877	0.0629973	INF	No Filter	Trace4	6.1	3.26	Automated - Genesis	119657	Echinacea purpurea	Leaves		
17	4.7434561	284.60737	4.733205	4.753688	93.0328369	273.629136	525.4982379	0.0204825	INF	No Filter	Trace4	0.96	1.85	Automated - Genesis	119657	Echinacea purpurea	Leaves		
18	4.7757567	286.5454	4.76392	4.785988	93.0329208	291.412545	476.976628	0.0220674	INF	No Filter	Trace4	1.02	1.68	Automated - Genesis	119657	Echinacea purpurea	Leaves		
19	4.7962207	287.77324	4.785988	4.806454	93.0332184	309.024755	594.0728228	0.0204659	INF	No Filter	Trace4	1.08	2.09	Automated - Genesis	119657	Echinacea purpurea	Leaves		
20	4.8269175	289.61505	4.806454	4.837149	93.033165	597.995342	589.4139611	0.0306954	INF	No Filter	Trace4	2.09	2.08	Automated - Genesis	119657	Echinacea purpurea	Leaves		
21	4.8592202	291.55321	4.847382	4.869456	93.0334015	359.851093	588.863892	0.0220738	INF	No Filter	Trace4	1.26	2.07	Automated - Genesis	119657	Echinacea purpurea	Leaves		
22	4.8899359	293.39615	4.879687	4.900167	93.0333786	404.987248	778.5220371	0.0204799	INF	No Filter	Trace4	1.41	2.74	Automated - Genesis	119657	Echinacea purpurea	Leaves		
23	4.9155166	294.931	4.905285	4.925748	93.0333023	501.070994	963.3040958	0.0204631	INF	No Filter	Trace4	1.75	3.39	Automated - Genesis	119657	Echinacea purpurea	Leaves		
24	4.9785136	298.71082	4.930865	4.993861	93.0334244	1986.60029	1015.353675	0.0629968	INF	No Filter	Trace4	6.94	3.58	Automated - Genesis	119657	Echinacea purpurea	Leaves		
25	5.027699	301.66194	4.998979	5.06198	0	2403.27187	1328.056793	0.0630011	INF	No Filter	Trace4	8.39	4.68	Automated - Genesis	119657	Echinacea purpurea	Leaves		
26	5.0801038	304.80623	5.067095	5.114741	0	1761.14231	1207.273034	0.0476453	INF	No Filter	Trace4	6.15	4.25	Automated - Genesis	119657	Echinacea purpurea	Leaves		
27	5.1300895	307.80537	5.119856	5.140323	93.0333023	377.147579	725.0362222	0.0204664	INF	No Filter	Trace4	1.32	2.55	Automated - Genesis	119657	Echinacea purpurea	Leaves		
28	5.1725606	310.35364	5.145439	5.186372	0	971.665374	906.0973683	0.0409337	INF	No Filter	Trace4	3.39	3.19	Automated - Genesis	119657	Echinacea purpurea	Leaves		
29	5.1982324	311.89394	5.186372	5.208465	93.0333405	444.012198	816.6858269	0.0220929	INF	No Filter	Trace4	1.55	2.88	Automated - Genesis	119657	Echinacea purpurea	Leaves		
30	5.2238134	313.4288	5.213581	5.249393	93.0332031	646.388621	712.0091677	0.0358126	INF	No Filter	Trace4	2.26	2.51	Automated - Genesis	119657	Echinacea purpurea	Leaves		
31	5.2647422	315.88453	5.254509	5.276579	93.0333176	311.749898	596.5838662	0.0220697	INF	No Filter	Trace4	1.09	2.1	Automated - Genesis	119657	Echinacea purpurea	Leaves		
32	5.3344119	320.06471	5.286813	5.348224	0	2478.23204	1346.72705	0.0614111	INF	No Filter	Trace4	8.65	4.74	Automated - Genesis	119657	Echinacea purpurea	Leaves		
33	5.3600646	321.60388	5.348224	5.370297	93.0329132	332.930574	544.7292048	0.0220727	INF	No Filter	Trace4	1.16	1.92	Automated - Genesis	119657	Echinacea purpurea	Leaves		
34	5.3805282	322.83169	5.370297	5.390761	93.0330811	383.441457	737.0937805	0.0204642	INF	No Filter	Trace4	1.34	2.6	Automated - Genesis	119657	Echinacea purpurea	Leaves		
1	0.9578659	57.471952	0.947564	0.968164	225.039673	1755.10338	3350.343453	0.02059997	INF	No Filter	Trace5	0.75	3.93	Automated - ICIS	119657	Echinacea purpurea	Leaves		
2	1.0683597	64.101582	1.014512	1.169731	0	45778.9599	13594.79033	0.155219	INF	No Filter	Trace5	19.63	15.93	Automated - ICIS	119657	Echinacea purpurea	Leaves		
3	1.2186871	73.121226	1.169731	1.234084	225.039932	14643.5603	5969.005058	0.0643526	INF	No Filter	Trace5	6.28	6.99	Automated - ICIS	119657	Echinacea purpurea	Leaves		
4	1.2672367	76.034202	1.234084	1.31793	0	21408.9006	7873.077169	0.0838466	INF	No Filter	Trace5	9.18	9.23	Automated - ICIS	119657	Echinacea purpurea	Leaves		
5	1.3305398	79.832388	1.31793	1.353862	0	4611.66277	3906.646101	0.0359316	INF	No Filter	Trace5	1.98	4.58	Automated - ICIS	119657	Echinacea purpurea	Leaves		
6	1.3696441	82.178646	1.353862	1.391414	0	3622.89668	3308.570958	0.0375518	INF	No Filter	Trace5	1.55	3.88	Automated - ICIS	119657	Echinacea purpurea	Leaves		
7	1.4222263	85.333578	1.411945	1.432492	225.040268	1213.53139	2320.875075	0.0205479	INF	No Filter	Trace5	0.52	2.72	Automated - ICIS	119657	Echinacea purpurea	Leaves		
8	1.4495165	86.97099	1.437626	1.485449	225.039932	3235.97834	2384.049181	0.0478231	INF	No Filter	Trace5	1.39	2.79	Automated - ICIS	119657	Echinacea purpurea	Leaves		
9	1.5486633	92.919798	1.538395	1.558927	225.040359	923.028736	1768.160628	0.0205322	INF	No Filter	Trace5	0.4	2.07	Automated - ICIS	119657	Echinacea purpurea	Leaves		
10	1.5920633	95.523798	1.579458	1.605432	0	1685.65373	2440.127753	0.0259736	INF	No Filter	Trace5	0.72	2.86	Automated - ICIS	119657	Echinacea purpurea	Leaves		
11	1.7432863	104.59718	1.733809	1.753864	225.03949	909.930054	1804.611577	0.0200548	INF	No Filter	Trace5	0.39	2.11	Automated - ICIS	119657	Echinacea purpurea	Leaves		
12	1.7762516	106.5751	1.766769	1.788452	225.040421	1124.60888	1886.00895	0.0216831	INF	No Filter	Trace5	0.48	2.21	Automated - ICIS	119657	Echinacea purpurea	Leaves		
13	1.808032	108.48192	1.793109	1.819856	0	1849.63773	2349.3759	0.026747	INF	No Filter	Trace5	0.79	2.75	Automated - ICIS	119657	Echinacea purpurea	Leaves		
14	1.8643749	111.86249	1.853279	1.874953	225.04068	999.078224	1870.568426	0.0216743	INF	No Filter	Trace5	0.43	2.19	Automated - ICIS	119657	Echinacea purpurea	Leaves		
15	1.9044925	114.26955	1.895016	1.928437	225.040375	2638.17465	3226.153473	0.0334208	INF	No Filter	Trace5	1.13	3.78	Automated - ICIS	119657	Echinacea purpurea	Leaves		
16	1.9418832	116.51299	1.928437	1.956827	0	1975.76434	2243.013902	0.0283896	INF	No Filter	Trace5	0.85	2.63	Automated - ICIS	119657	Echinacea purpurea	Leaves		
17	1.9997344	119.98406	1.990258	2.010312	225.040192	774.924523	1518.015897	0.020054	INF	No Filter	Trace5	0.33	1.78	Automated - ICIS	119657	Echinacea purpurea	Leaves		
18	2.0593719	123.56231	2.049104	2.085036	225.040161	2000.94815	1938.595965	0.0359316	INF	No Filter	Trace5	0.86	2.27	Automated - ICIS	119657	Echinacea purpurea	Leaves	Isochorismate C10H10O6	Level 4
19	2.2268682	133.61209	2.216604	2.237133	225.039841	980.993511	1879.1908	0.0205292	INF	No Filter	Trace5	0.42	2.2	Automated - ICIS	119657	Echinacea purpurea	Leaves		
20	2.3568428	141.41057	2.346579	2.368728	225.040024	1227.11492	1999.423699	0.0221496	INF	No Filter	Trace5	0.53	2.34	Automated - ICIS	119657	Echinacea purpurea	Leaves		
21	2.6473016	158.8381	2.567453	2.762006	225.039993	119908.09	17703.70104	0.194553	INF	No Filter	Trace5	51.4	20.75	Automated - ICIS	119657	Echinacea purpurea	Leaves	Chorismate C10H10O6	Level 4
1	0.6814684	40.888103	0.624185	1.105958	174.07666	5015726.87	1417932.368	0.48177225	240.9428265	No Filter	Trace6	100	100	Automated - Genesis	119657	Echinacea purpurea	Leaves	C15H17NO9 Isochorismate-9-glutamate	Level 2
1	2.4309845	145.85907	2.411679	2.881752	179.034164	258211.845	36577.82879	0.470072993	INF	No Filter	Trace7	100	100	Manual	119657	Echinacea purpurea	Leaves		
1	3.6570673	219.42404	3.632006	3.667808	163.039322	10843.0286	10164.50942	0.0358021	5.958409153	No Filter	Trace8	69.06	53.34	Automated - Genesis	119657	Echinacea purpurea	Leaves		
2	3.7052132	222.31279	3.694473	3.715954	163.039429	4857.51484	8890.754085	0.0214813	5.211737067	No Filter	Trace8	30.94	46.66	Automated - Genesis	119657	Echinacea purpurea	Leaves		
1	1.5525557	93.153342	1.505979	1.653379	0	33773.7327	7965.851882	0.1473996	INF	No Filter	Trace9	11.25	14.44	Automated - Genesis	119657	Echinacea purpurea	Leaves		
2	2.8822204	172.93322	2.80003	3.079094	223.060532	220941.62	31966.64996	0.2790642	INF	No Filter	Trace9	73.58	57.94	Automated - Genesis	119657	Echinacea purpurea	Leaves		
3	3.1635055	189.81033	3.090712	3.212264	223.060638	24907.7991	8819.179843	0.1215523	INF	No Filter	Trace9	8.29	15.98	Automated - Genesis	119657	Echinacea purpurea	Leaves		
4	4.2877956	257.26774	4.227579	4.331502	0	20655.9357	6421.899599	0.103923	INF	No Filter	Trace9	6.88	11.64	Automated - Genesis	119657	Echinacea purpurea	Leaves		
1	0.8120086	48.720517	0.804051	0.821068	353.089325	35193.5578	67888.69372	0.017017	5.626436739	No Filter	Trace10	7.47	45.94	Automated - Genesis	119657	Echinacea purpurea	Leaves		
2	1.1697309	70.183854	1.115905	1.492784	353.087982	90434.278	17941.28981	0.3768785	INF	No Filter	Trace10	19.2	12.14	Manual	119657	Echinacea purpurea	Leaves	C16H18O9 neochlorogenic acid (3-o-caffeoylquinic acid)	Level 4
3	1.5987474	95.924844	1.532847	1.665693	0	118570.999	31963.98781	0.132846715	INF	No Filter	Trace10	25.18	21.63	Manual	119657	Echinacea purpurea	Leaves	C16H18O9 chlorogenic acid (5-o-caffeoylquinic acid)	Level 4
4	1.6757489	100.54493	1.675912	1.982482	353.087433	188689.74	20589.23833	0.306569343	INF	No Filter	Trace10	40.06	13.93	Manual	119657	Echinacea purpurea	Leaves	C16H18O9 cis 5-o-caffeoylquinic acid	Level 4
5	2.7294781	163.76869	2.268613	2.922628	353.087097	38093.1072	9400.619312	0.654014599	INF	No Filter	Trace10	8.09	6.36	Manual	119657	Echinacea purpurea	Leaves	C16H18O9 4-o-caffeoylquinic acid (crytochlorogenic acid)	Level 4

1	4.6781005	280.68603	4.629355	4.792708	515.118896	78086.4918	17840.85378	0.163353	INF	No Filter	Trace11	10.68	11.58	Manual	119657	Echinacea purpurea	Leaves	C25H24O12 3.4-dicaffeolquinic acid	Level 4
2	4.8671151	292.02691	4.813171	5.105792	515.119019	180919.537	26847.56948	0.2926208	INF	No Filter	Trace11	24.74	17.43	Manual	119657	Echinacea purpurea	Leaves	C25H24O12 3,5-dicaffeolquinic acid	Level 4
3	5.1790233	310.7414	5.157318	5.236896	515.120972	25126.4868	8913.711096	0.0795779	INF	No Filter	Trace11	3.44	5.79	Automated - ICIS	119657	Echinacea purpurea	Leaves		
4	5.2676116	316.0567	5.236896	5.330608	515.119812	16697.4539	5661.903645	0.0937122	INF	No Filter	Trace11	2.28	3.68	Automated - ICIS	119657	Echinacea purpurea	Leaves		
5	5.3510724	321.06434	5.330608	5.35619	515.121643	6546.08061	7768.475577	0.0255812	INF	No Filter	Trace11	0.9	5.04	Automated - ICIS	119657	Echinacea purpurea	Leaves		
6	5.4146272	324.87763	5.35619	5.648559	515.120239	347364.602	51931.35089	0.2923693	INF	No Filter	Trace11	47.51	33.71	Automated - ICIS	119657	Echinacea purpurea	Leaves	C25H24O12 4,5-dicaffeolquinic acid	Level 4
7	5.7026203	342.15722	5.66646	5.725341	0	17439.4415	6624.974759	0.058881	INF	No Filter	Trace11	2.39	4.3	Automated - ICIS	119657	Echinacea purpurea	Leaves		
8	5.7539826	345.23896	5.725341	5.770644	515.120239	10977.8009	6481.290754	0.045303	INF	No Filter	Trace11	1.5	4.21	Automated - ICIS	119657	Echinacea purpurea	Leaves		
9	5.7937293	347.62376	5.770644	5.829246	0	13056.4113	7172.927705	0.0586025	INF	No Filter	Trace11	1.79	4.66	Automated - ICIS	119657	Echinacea purpurea	Leaves		
10	5.8497102	350.98261	5.829246	5.885892	515.120056	10911.1319	4794.418327	0.0566456	INF	No Filter	Trace11	1.49	3.11	Automated - ICIS	119657	Echinacea purpurea	Leaves		
11	5.9859475	359.15685	5.915055	5.998521	515.1203	13547.3381	4117.948166	0.0834659	INF	No Filter	Trace11	1.85	2.67	Automated - ICIS	119657	Echinacea purpurea	Leaves		
12	6.0191964	361.15178	5.998521	6.069688	515.11969	10511.7717	5877.420261	0.0711674	INF	No Filter	Trace11	1.44	3.82	Automated - ICIS	119657	Echinacea purpurea	Leaves		
1	0.646906	38.814358	0.625425	0.657647	473.074829	18231.4346	24280.49377	0.032222	INF	No Filter	Trace13	28.62	33.13	Automated - Genesis	119657	Echinacea purpurea	Leaves	C22H18O12 cichoric acid	Level 4
2	2.8162938	168.97763	2.802353	2.830235	473.072327	1895.48617	2672.892845	0.0278817	INF	No Filter	Trace13	2.98	3.65	Automated - Genesis	119657	Echinacea purpurea	Leaves		
3	3.5766972	214.60183	3.565957	3.587438	473.074585	1409.93799	2580.62713	0.0214813	INF	No Filter	Trace13	2.21	3.52	Automated - Genesis	119657	Echinacea purpurea	Leaves		
4	6.2053379	372.32027	6.192257	6.217318	0	2021.96795	3282.944363	0.0250614	INF	No Filter	Trace13	3.17	4.48	Automated - Genesis	119657	Echinacea purpurea	Leaves		
5	6.5037387	390.22432	6.490657	6.515719	0	2310.25834	3751.032173	0.0250612	INF	No Filter	Trace13	3.63	5.12	Automated - Genesis	119657	Echinacea purpurea	Leaves		
6	6.5626232	393.75739	6.549542	6.574603	0	1796.55498	2916.925777	0.0250615	INF	No Filter	Trace13	2.82	3.98	Automated - Genesis	119657	Echinacea purpurea	Leaves		
7	7.9237132	475.42279	7.895072	7.953964	473.072205	3666.85618	2776.601688	0.0588916	INF	No Filter	Trace13	5.76	3.79	Automated - Genesis	119657	Echinacea purpurea	Leaves		
8	8.0593943	483.56366	8.034333	8.070135	473.072876	2751.5978	2585.88756	0.0358016	INF	No Filter	Trace13	4.32	3.53	Automated - Genesis	119657	Echinacea purpurea	Leaves		
9	8.105937	486.35622	8.095196	8.136185	473.072266	2912.46602	3025.233141	0.040989	INF	No Filter	Trace13	4.57	4.13	Automated - Genesis	119657	Echinacea purpurea	Leaves		
10	8.2690147	496.14088	8.230872	8.3005	0	5926.80278	4121.185137	0.069628	INF	No Filter	Trace13	9.31	5.62	Automated - Genesis	119657	Echinacea purpurea	Leaves		
11	8.3243216	499.4593	8.31124	8.377289	0	5790.19743	3958.489929	0.0660489	INF	No Filter	Trace13	9.09	5.4	Automated - Genesis	119657	Echinacea purpurea	Leaves		
12	8.5523513	513.14108	8.522099	8.580993	473.070587	3408.26617	2567.034216	0.0588943	INF	No Filter	Trace13	5.35	3.5	Automated - Genesis	119657	Echinacea purpurea	Leaves		
13	8.8006179	528.03707	8.789877	8.811359	473.07254	1657.3232	3033.414154	0.0214813	INF	No Filter	Trace13	2.6	4.14	Automated - Genesis	119657	Echinacea purpurea	Leaves		
14	8.869003	532.14018	8.854321	8.884563	0	2577.74536	3221.552248	0.030242	INF	No Filter	Trace13	4.05	4.4	Automated - Genesis	119657	Echinacea purpurea	Leaves		
15	8.9298665	535.79199	8.888143	8.943449	0	4083.16192	2993.755751	0.0553057	INF	No Filter	Trace13	6.41	4.08	Automated - Genesis	119657	Echinacea purpurea	Leaves		
16	8.9541897	537.25138	8.943449	8.96493	473.073639	1606.18773	2939.829924	0.0214809	INF	No Filter	Trace13	2.52	4.01	Automated - Genesis	119657	Echinacea purpurea	Leaves		
17	9.0222133	541.3328	9.011473	9.03456	473.07373	1646.7193	2584.031694	0.0230871	INF	No Filter	Trace13	2.59	3.53	Automated - Genesis	119657	Echinacea purpurea	Leaves		
1	5.9437482	356.62489	5.928035	5.955219	0	1972.26958	2557.21249	0.0271837	INF	No Filter	Trace14	0.57	5.75	Automated - Genesis	119657	Echinacea purpurea	Leaves		
2	6.7763185	406.57911	6.760398	6.790639	301.035492	3262.72085	3999.497002	0.0302411	INF	No Filter	Trace14	0.95	9	Automated - Genesis	119657	Echinacea purpurea	Leaves		
3	7.118811	427.12866	7.003026	7.843343	301.035553	326472.812	24880.52714	0.8403168	INF	No Filter	Trace14	94.66	55.96	Automated - Genesis	119657	Echinacea purpurea	Leaves	C15H10O7 quercetin	Level 4
4	7.8651907	471.91144	7.850504	7.877171	0	2130.88354	3382.556675	0.0266674	INF	No Filter	Trace14	0.62	7.61	Automated - Genesis	119657	Echinacea purpurea	Leaves		
5	7.9296344	475.77806	7.877171	7.943223	0	6066.90937	3385.328811	0.0660521	INF	No Filter	Trace14	1.76	7.61	Automated - Genesis	119657	Echinacea purpurea	Leaves		
6	7.9813657	478.88194	7.968284	8.000506	0	3537.97849	3631.217348	0.0322223	INF	No Filter	Trace14	1.03	8.17	Automated - Genesis	119657	Echinacea purpurea	Leaves		
7	8.2093912	492.56347	8.19865	8.220132	301.034882	1433.32889	2623.421657	0.0214812	INF	No Filter	Trace14	0.42	5.9	Automated - Genesis	119657	Echinacea purpurea	Leaves		
1	7.1057358	426.34415	7.017155	7.796801	0	2636723.14	221906.631	0.7796454	INF	No Filter	Trace15	78.44	61.33	Manual	119657	Echinacea purpurea	Leaves	C15H10O6 Luteolin	Level 4
2	7.922474	475.34844	7.86161	7.993346	0	33409.2741	6862.85209	0.1317357	INF	No Filter	Trace15	0.99	1.9	Automated - ICIS	119657	Echinacea purpurea	Leaves		
3	8.0100073	480.60044	7.993346	8.150506	0	32513.721	7504.819856	0.1571597	INF	No Filter	Trace15	0.97	2.07	Automated - ICIS	119657	Echinacea purpurea	Leaves		
4	8.2511136	495.06682	8.150506	8.263094	0	29541.9254	7313.547517	0.1125883	INF	No Filter	Trace15	0.88	2.02	Automated - ICIS	119657	Echinacea purpurea	Leaves		
5	8.3665486	501.99292	8.263094	8.408272	285.040253	44378.4009	7341.053761	0.1451776	INF	No Filter	Trace15	1.32	2.03	Automated - ICIS	119657	Echinacea purpurea	Leaves		
6	8.4309923	505.85954	8.408272	8.548771	285.040253	53492.7349	10083.63853	0.1404995	INF	No Filter	Trace15	1.59	2.79	Automated - ICIS	119657	Echinacea purpurea	Leaves		
7	8.6398772	518.39263	8.548771	8.865423	285.040649	165845.265	19826.46008	0.3166516	INF	No Filter	Trace15	4.93	5.48	Automated - ICIS	119657	Echinacea purpurea	Leaves	C15H10O6 Kaempferol	Level 4
8	8.9470293	536.82176	8.865423	9.004312	285.040497	26010.5938	5784.541068	0.1388894	INF	No Filter	Trace15	0.77	1.6	Automated - ICIS	119657	Echinacea purpurea	Leaves		
9	9.0798632	544.79179	9.004312	9.178131	0	46207.6998	6903.713581	0.1738184	INF	No Filter	Trace15	1.37	1.91	Automated - ICIS	119657	Echinacea purpurea	Leaves		
10	9.2298508	553.79105	9.178131	9.252571	0	17008.8988	7643.37768	0.0744408	INF	No Filter	Trace15	0.51	2.11	Automated - ICIS	119657	Echinacea purpurea	Leaves		
11	9.3918238	563.50943	9.252571	9.412066	285.040405	23951.4184	5880.562752	0.1594943	INF	No Filter	Trace15	0.71	1.63	Automated - ICIS	119657	Echinacea purpurea	Leaves		
12	9.5388609	572.33165	9.412066	9.693916	285.040802	66680.2361	9222.955102	0.2818499	INF	No Filter	Trace15	1.98	2.55	Automated - ICIS	119657	Echinacea purpurea	Leaves		
13	9.867568	592.05408	9.693916	10.0296	0	65229.6723	8525.412833	0.3356795	INF	No Filter	Trace15	1.94	2.36	Automated - ICIS	119657	Echinacea purpurea	Leaves		
14	10.048183	602.89098	10.0296	10.09698	285.03949	13922.2477	8342.766929	0.067381	INF	No Filter	Trace15	0.41	2.31	Automated - ICIS	119657	Echinacea purpurea	Leaves		
15	10.172169	610.33014	10.1257	10.31792	285.039612	27378.3495	5134.435225	0.192219	INF	No Filter	Trace15	0.81	1.42	Automated - ICIS	119657	Echinacea purpurea	Leaves		
16	10.348123	620.88738	10.31792	10.51246	285.040131	33176.5761	6676.237929	0.194539	INF	No Filter	Trace15	0.99	1.85	Automated - ICIS	119657	Echinacea purpurea	Leaves		
17	10.583007	634.98042	10.56442	10.64427	285.040741	11713.7528	4684.665264	0.079848	INF	No Filter	Trace15	0.35	1.29	Automated - ICIS	119657	Echinacea purpurea	Leaves		
18	10.822547	649.35282	10.76361	10.8931	285.039581	19137.0128	4317.754649	0.129488	INF	No Filter	Trace15	0.57	1.19	Automated - ICIS	119657	Echinacea purpurea	Leaves		
19	10.959002	657.54012	10.8931	10.98456	285.040588	8273.88284	4069.289055	0.091461	INF	No Filter	Trace15	0.25	1.12	Automated - ICIS	119657	Echinacea purpurea	Leaves		
20	11.691627	701.49762	11.63267	11.70789	0	6993.9397	3784.642158	0.075225	INF	No Filter	Trace15	0.21	1.05	Automated - ICIS	119657	Echinacea purpurea	Leaves		
1	8.3832101	502.99261	8.277779	9.869892	0	2835308.03	220326.4305	1.5921127	INF	No Filter	Trace16	100	100	Automated - Genesis	119657	Echinacea purpurea	Leaves	C15H10O5 apigenin	Level 4
1	1.1044035	66.26421	1.095674	1.113306	137.023972	444.826934	1057.335015	0.0176315	INF	No Filter	Trace17	8.64	9.88	Automated - Genesis	119657	Echinacea purpurea	Leaves		
2	3.9976195	239.85717	3.989729	4.006613	137.023788	1389.26006	3330.665465	0.0168843	INF	No Filter	Trace17	26.99	31.14	Automated - Genesis	119657	Echinacea purpurea	Leaves		
3	4.1822634	250.9358	4.173568	4.190158	137.023773	271.168616	649.6740457	0.0165902	INF	No Filter	Trace17	5.27	6.07	Automated - Genesis	119657	Echinacea purpurea	Leaves		
4	4.5193919	271.16351	4.510992	4.530494	137.023743	818.250979	1743.545434	0.0195021	INF	No Filter	Trace17	15.9	16.3	Automated - Genesis	119657	Echinacea purpurea	Leaves		
5	9.623354	577.40124	9.611736	9.634971	137.023743	1020.46522	1796.246598	0.0232351	INF	No Filter	Trace17	19.82	16.79	Automated - Genesis	119657	Echinacea purpurea	Leaves		
6	11.548175	692.8905	11.53656	11.55979	137.023727	1203.82877	2119.01066	0.023235	INF	No Filter	Trace17	23.39	19.81	Automated - Genesis	119657	Echinacea purpurea	Leaves		
1	1.6592758	99.556548	1.653379	1.666272	153.018555	3819.74808	9530.465465	0.0128934	27.30039941	No Filter	Trace18	100	100	Automated - ICIS	119657	Echinacea purpurea	Leaves		
1	3.9707991	238.24795	3.575457	5.80355	0	274625268	25273434.65	2.2280923	INF	No Filter	Trace21	100	100	Automated - Genesis	119657	Echinacea purpurea	Leaves	C21H20O10 apigenin-o-glucoside	Level 3
1	1.5971957	95.831742	1.561268	1.626328	0	48492.96	22433.36437	0.0650597	INF	No Filter	Trace22	0.04	0.09	Automated - ICIS	119657	Echinacea purpurea	Leaves		
2	1.6345656	98.073936	1.626328	1.692238	593.154053	18962.9212	11597.60203	0.0659104	INF	No Filter	Trace22	0.02	0.05	Automated - ICIS	119657	Echinacea purpurea	Leaves		
3	2.0850357	125.10214	1.986365	2.210232	593.152344	149922.19	22272.46003	0.2238668	INF	No Filter	Trace22	0.12	0.09	Automated - ICIS	119657	Echinacea purpurea	Leaves		
4	2.3157833	138.947	2.260021	2.581394	593.151733	46733982.5	6962970.639	0.3213734	INF	No Filter	Trace22	38.12	28.9	Automated - ICIS	119657	Echinacea purpurea	Leaves	C27H30O15 luteolin diglycosides	Level 3
5	2.8682793	172.09676	2.665889	3.034948	593.151733	4857576.54	734572.2519	0.369059	INF	No Filter	Trace22	3.96	3.05	Automated - ICIS	119657	Echinacea purpurea	Leaves		
6	3.1611818	189.67091	3.055859	3.236086	593.151306	2248615.24	440123.7794	0.1802262	INF	No Filter	Trace22	1.83	1.83	Automated - ICIS	119657	Echinacea purpurea	Leaves		

7	3.2854731	197.12839	3.236086	3.430282	593.151672	654673.603	185540.6911	0.1941968	INF	No Filter	Trace22	0.53	0.77	Automated - ICIS	119657	Echinacea purpurea	Leaves		
8	3.5444753	212.66852	3.480769	3.703974	593.151611	65380147	15026068.81	0.2232052	INF	No Filter	Trace22	53.33	62.37	Automated - ICIS	119657	Echinacea purpurea	Leaves	C27H30O15 luteolin diglycosides	Level 3
9	3.7950811	227.70487	3.729035	3.805023	0	459732.702	154653.6139	0.0759874	INF	No Filter	Trace22	0.38	0.64	Automated - ICIS	119657	Echinacea purpurea	Leaves		
10	3.8459681	230.75809	3.839613	3.892376	593.1521	37139.6599	33643.37676	0.0527626	INF	No Filter	Trace22	0.03	0.14	Automated - ICIS	119657	Echinacea purpurea	Leaves		
11	3.9243117	235.4587	3.892376	3.945218	593.151794	44853.5194	30334.90355	0.0528418	INF	No Filter	Trace22	0.04	0.13	Automated - ICIS	119657	Echinacea purpurea	Leaves		
12	3.9669216	238.0153	3.959031	4.001498	593.151428	20642.7238	17796.51445	0.0424668	INF	No Filter	Trace22	0.02	0.07	Automated - ICIS	119657	Echinacea purpurea	Leaves		
13	4.0913377	245.48026	4.001498	4.110639	593.152649	88763.0301	22934.33976	0.1091411	INF	No Filter	Trace22	0.07	0.1	Automated - ICIS	119657	Echinacea purpurea	Leaves		
14	4.1850421	251.10253	4.110639	4.333842	593.15155	986303.177	229753.7933	0.2232032	INF	No Filter	Trace22	0.8	0.95	Automated - ICIS	119657	Echinacea purpurea	Leaves	C27H30O15 Kaempferol diglycosides	Level 3
15	4.3689442	262.13665	4.333842	4.394525	593.15155	13319.7409	7694.485326	0.0606825	INF	No Filter	Trace22	0.01	0.03	Automated - ICIS	119657	Echinacea purpurea	Leaves	C27H30O15 Kaempferol diglycosides	Level 3
16	4.4055613	264.33368	4.394525	4.528153	0	117445.387	24447.01377	0.1336284	INF	No Filter	Trace22	0.1	0.1	Automated - ICIS	119657	Echinacea purpurea	Leaves		
17	4.5806167	274.837	4.528153	4.603633	0	54536.0443	18058.3944	0.0754804	INF	No Filter	Trace22	0.04	0.07	Automated - ICIS	119657	Echinacea purpurea	Leaves		
18	4.6753209	280.51925	4.603633	4.73598	0	91873.0317	16236.81025	0.1323467	INF	No Filter	Trace22	0.07	0.07	Automated - ICIS	119657	Echinacea purpurea	Leaves	C27H30O15 Kaempferol diglycosides	Level 3
19	4.7449916	284.6995	4.73598	4.783647	0	27970.7138	13591.00909	0.0476673	INF	No Filter	Trace22	0.02	0.06	Automated - ICIS	119657	Echinacea purpurea	Leaves		
20	4.8950522	293.70313	4.783647	4.950593	0	79460.3802	12420.62504	0.1669453	INF	No Filter	Trace22	0.06	0.05	Automated - ICIS	119657	Echinacea purpurea	Leaves	C27H30O15 Kaempferol diglycosides	Level 3
21	4.9800494	298.80296	4.950593	5.059639	0	43777.2943	9575.188889	0.1090464	INF	No Filter	Trace22	0.04	0.04	Automated - ICIS	119657	Echinacea purpurea	Leaves		
22	5.1789163	310.73498	5.059639	5.21124	593.152649	46666.3876	8511.480866	0.1516012	INF	No Filter	Trace22	0.04	0.04	Automated - ICIS	119657	Echinacea purpurea	Leaves		
23	5.2726339	316.35803	5.21124	5.390761	593.152039	72940.5041	10823.3629	0.1795208	INF	No Filter	Trace22	0.06	0.04	Automated - ICIS	119657	Echinacea purpurea	Leaves		
24	5.4395709	326.37425	5.392296	5.643628	593.152649	96839.0155	12645.03497	0.2513319	INF	No Filter	Trace22	0.08	0.05	Automated - ICIS	119657	Echinacea purpurea	Leaves		
25	5.7109105	342.65463	5.643628	5.871755	593.151184	133557.174	28350.21135	0.228127	INF	No Filter	Trace22	0.11	0.12	Automated - ICIS	119657	Echinacea purpurea	Leaves		
26	5.8973377	353.84026	5.871755	5.943748	593.151306	7560.75274	4291.132777	0.0719929	INF	No Filter	Trace22	0.01	0.02	Automated - ICIS	119657	Echinacea purpurea	Leaves		
27	6.0048496	360.29098	5.943748	6.073236	593.151184	18956.2317	6622.597362	0.1294879	INF	No Filter	Trace22	0.02	0.03	Automated - ICIS	119657	Echinacea purpurea	Leaves		
28	6.1154651	366.92791	6.080397	6.259041	593.151489	31830.0226	6927.460163	0.1786444	INF	No Filter	Trace22	0.03	0.03	Automated - ICIS	119657	Echinacea purpurea	Leaves		
29	6.3084394	378.50636	6.269782	6.353742	593.153076	13494.7206	6935.905085	0.0839608	INF	No Filter	Trace22	0.01	0.03	Automated - ICIS	119657	Echinacea purpurea	Leaves		
30	6.3852253	383.11352	6.353742	6.394726	593.152466	4361.82702	3858.690542	0.0409839	INF	No Filter	Trace22	0	0.02	Automated - ICIS	119657	Echinacea purpurea	Leaves		
31	6.4174476	385.04686	6.398307	6.457196	593.148743	4530.01499	3057.62442	0.0588896	INF	No Filter	Trace22	0	0.01	Automated - ICIS	119657	Echinacea purpurea	Leaves		
32	6.5889238	395.33543	6.580524	6.628669	593.153687	3401.79128	2704.057533	0.0481453	INF	No Filter	Trace22	0	0.01	Automated - ICIS	119657	Echinacea purpurea	Leaves		
1	2.5178058	151.06835	2.391241	2.646715	563.141052	21586072.9	4320323.821	0.255474453	INF	No Filter	Trace23	4.63	6.38	Manual	119657	Echinacea purpurea	Leaves		
2	2.8534694	171.20816	2.80003	3.003885	563.14093	52000116	7585303.042	0.2038557	INF	No Filter	Trace23	11.15	11.2	Manual	119657	Echinacea purpurea	Leaves		
3	3.0674767	184.0486	3.00438	3.208759	563.140625	333245096	43967894.81	0.204379562	INF	No Filter	Trace23	71.48	64.91	Manual	119657	Echinacea purpurea	Leaves	C26H28O14 apigenin di-glycoside family	Level 3
4	3.3307761	199.84657	3.290511	3.423358	0	39565739.1	7382699.39	0.132846715	INF	No Filter	Trace23	8.49	10.9	Manual	119657	Echinacea purpurea	Leaves		
5	3.4771882	208.63129	3.454015	3.576642	0	19831409.5	4483938.62	0.122627737	INF	No Filter	Trace23	4.25	6.62	Manual	119657	Echinacea purpurea	Leaves		
1	3.1046521	186.27913	3.004595	3.208989	447.093597	103514268	16839952.87	0.204394204	2594.359066	No Filter	Trace24	68.48	79.31	Manual	119657	Echinacea purpurea	Leaves	C21H20O11 Luteolin-O-glucoside	Level 3
2	3.2878136	197.26882	3.219209	3.699535	0	47654011	4393321.032	0.48032638	688.7037234	No Filter	Trace24	31.52	20.69	Manual	119657	Echinacea purpurea	Leaves	C21H20O11 Luteolin-O-glucoside; C21H20O11 kaempferol-O-glucoside	Level 3
1	0.7228652	43.371912	0.712563	0.733165	216.050476	7118.53713	13586.01659	0.02060206	INF	No Filter	Trace25	4.03	21.47	Automated - Genesis	119657	Echinacea purpurea	Leaves		
2	0.7589111	45.534664	0.748613	0.769209	216.050598	8683.2625	16578.99949	0.02059662	INF	No Filter	Trace25	4.92	26.2	Automated - Genesis	119657	Echinacea purpurea	Leaves		
3	0.860719	51.643142	0.801733	1.102063	0	148251.885	19672.30092	0.3003298	INF	No Filter	Trace25	83.97	31.09	Automated - Genesis	119657	Echinacea purpurea	Leaves	i9G 2-hydroxyacryloyl-N-glutamate C8H11NO6	Level 4
4	1.1282795	67.69677	1.105958	1.15352	0	5530.16844	3752.470543	0.0475623	INF	No Filter	Trace25	3.13	5.93	Automated - Genesis	119657	Echinacea purpurea	Leaves		
5	1.1898373	71.390238	1.180329	1.202049	216.050659	1294.72928	2146.171458	0.0217208	INF	No Filter	Trace25	0.73	3.39	Automated - Genesis	119657	Echinacea purpurea	Leaves		
6	1.2186871	73.121226	1.208421	1.239217	216.051071	2493.80356	2402.608412	0.0307964	INF	No Filter	Trace25	1.41	3.8	Automated - Genesis	119657	Echinacea purpurea	Leaves		
7	1.2597468	75.584808	1.249483	1.270029	216.050476	1062.9604	2033.301547	0.0205464	INF	No Filter	Trace25	0.6	3.21	Automated - Genesis	119657	Echinacea purpurea	Leaves		
8	1.2894768	77.368608	1.276872	1.302533	0	2125.68389	3100.696026	0.0256613	INF	No Filter	Trace25	1.2	4.9	Automated - Genesis	119657	Echinacea purpurea	Leaves		
1	0.6277657	37.665943	0.602283	0.654066	0	54533.8975	36359.01615	0.05178337	INF	No Filter	Trace26	10.95	20.44	Automated - Genesis	119657	Echinacea purpurea	Leaves		
2	0.7177126	43.062756	0.707415	0.728015	193.049683	8667.70054	16544.14062	0.02060075	INF	No Filter	Trace26	1.74	9.3	Automated - Genesis	119657	Echinacea purpurea	Leaves		
3	0.9527148	57.162885	0.940792	0.963015	193.049988	3374.63482	6411.520909	0.02222315	INF	No Filter	Trace26	0.68	3.6	Automated - Genesis	119657	Echinacea purpurea	Leaves		
4	0.9784626	58.707757	0.968164	0.988761	193.04982	2373.48312	4531.716351	0.02059676	INF	No Filter	Trace26	0.48	2.55	Automated - Genesis	119657	Echinacea purpurea	Leaves		
5	1.3048742	78.292452	1.270029	1.31793	0	5084.76601	4013.518208	0.0479011	INF	No Filter	Trace26	1.02	2.26	Automated - Genesis	119657	Echinacea purpurea	Leaves		
6	2.4411309	146.46785	2.421691	2.459719	193.049911	4979.38881	5182.512623	0.0380281	INF	No Filter	Trace26	1	2.91	Automated - Genesis	119657	Echinacea purpurea	Leaves		
7	2.8752499	172.51499	2.839529	2.919396	193.05011	8296.21182	3873.438646	0.0798669	INF	No Filter	Trace26	1.67	2.18	Automated - Genesis	119657	Echinacea purpurea	Leaves		
8	3.4039814	204.23888	3.362259	3.426702	0	6546.8384	3725.119057	0.0644433	INF	No Filter	Trace26	1.31	2.09	Automated - Genesis	119657	Echinacea purpurea	Leaves		
9	3.7915008	227.49005	3.698053	3.82549	0	29026.3646	7648.640566	0.1274375	INF	No Filter	Trace26	5.83	4.3	Automated - Genesis	119657	Echinacea purpurea	Leaves		
10	3.838077	230.28462	3.82549	3.934544	0	15835.0793	5888.380003	0.1090532	INF	No Filter	Trace26	3.18	3.31	Automated - Genesis	119657	Echinacea purpurea	Leaves		
11	4.4477877	266.86726	4.196882	4.845042	193.049866	126923.026	6592.117042	0.6481596	INF	No Filter	Trace26	25.48	3.71	Automated - Genesis	119657	Echinacea purpurea	Leaves		
12	5.6198063	337.18838	5.451916	5.822775	193.04985	161504.957	21656.49464	0.3708597	5.273926625	No Filter	Trace26	32.43	12.17	Automated - Genesis	119657	Echinacea purpurea	Leaves	C10H10O4 Ferulic acid	Level 4
13	6.628669	397.72014	6.592504	6.66213	0	6877.07357	4041.148296	0.0696259	INF	No Filter	Trace26	1.38	2.27	Automated - Genesis	119657	Echinacea purpurea	Leaves		
14	6.7484181	404.90509	6.735337	6.787059	0	4815.1848	3807.290469	0.0517218	INF	No Filter	Trace26	0.97	2.14	Automated - Genesis	119657	Echinacea purpurea	Leaves		
15	6.8121206	408.72724	6.787059	6.847923	193.049652	7971.98822	4216.072861	0.0608637	INF	No Filter	Trace26	1.6	2.37	Automated - Genesis	119657	Echinacea purpurea	Leaves		
16	6.9032274	414.19364	6.888907	6.917548	193.050034	2943.10501	4170.514366	0.0286417	INF	No Filter	Trace26	0.59	2.34	Automated - Genesis	119657	Echinacea purpurea	Leaves		
17	9.6256776	577.54066	9.564419	9.655883	193.050095	7158.7365	4315.384157	0.0914635	INF	No Filter	Trace26	1.44	2.43	Automated - Genesis	119657	Echinacea purpurea	Leaves		
18	9.9799506	598.79704	9.945099	9.996215	0	4096.02429	3789.070458	0.0511167	INF	No Filter	Trace26	0.82	2.13	Automated - Genesis	119657	Echinacea purpurea	Leaves		
19	10.027271	601.63626	10.00086	10.04586	0	4813.04686	3856.130418	0.044997	INF	No Filter	Trace26	0.97	2.17	Automated - Genesis	119657	Echinacea purpurea	Leaves		
20	10.261306	615.67836	10.24504	10.32024	193.05011	5215.90612	3938.407655	0.0752	INF	No Filter	Trace26	1.05	2.21	Automated - Genesis	119657	Echinacea purpurea	Leaves		
21	10.500839	630.05034	10.48225	10.53189	193.049774	4652.02784	3661.081757	0.049638	INF	No Filter	Trace26	0.93	2.06	Automated - Genesis	119657	Echinacea purpurea	Leaves		
22	11.162851	669.77106	11.13729	11.18144	193.049957	4870.74269	4257.982402	0.044146	INF	No Filter	Trace26	0.98	2.39	Automated - Genesis	119657	Echinacea purpurea	Leaves		
23	12.719567	763.17402	12.69633	12.73583	193.050415	4148.49799	4073.33295	0.0395	INF	No Filter	Trace26	0.83	2.29	Automated - Genesis	119657	Echinacea purpurea	Leaves		
24	13.097072	785.82432	13.08081	13.1258	193.051285	4836.28173	3893.795873	0.044987	INF	No Filter	Trace26	0.97	2.19	Automated - Genesis	119657	Echinacea purpurea	Leaves		

25	13.76378	825.8268	13.7359	13.78089	0	4110.11143	3663.22009	0.044988	INF		No Filter	Trace26	0.83	2.06	Automated - Genesis	119657	Echinacea purpurea	Leaves		
26	13.811091	828.66546	13.79483	13.84362	0	4398.73081	3806.785871	0.048793	INF		No Filter	Trace26	0.88	2.14	Automated - Genesis	119657	Echinacea purpurea	Leaves		
1	1.5561358	93.368148	1.533262	1.605432	0	26531.6219	12439.68061	0.0721692	INF		No Filter	Trace27	4.56	9.08	Automated - ICIS	119657	Echinacea purpurea	Leaves	C17H22O10 Sinapoyl-glucose	Level 3
2	1.7020997	102.12598	1.690999	1.729152	385.114563	3112.63697	3191.027951	0.0381531	INF		No Filter	Trace27	0.53	2.33	Automated - ICIS	119657	Echinacea purpurea	Leaves		
3	2.5061884	150.3713	2.448102	2.677507	385.113983	471570.086	105835.2178	0.2294051	INF		No Filter	Trace27	80.99	77.26	Automated - ICIS	119657	Echinacea purpurea	Leaves	C17H22O10 Sinapoyl-glucose	Level 3
4	2.7318012	163.90807	2.717861	2.790736	385.11438	5561.07014	2662.342806	0.0728754	INF		No Filter	Trace27	0.96	1.94	Automated - ICIS	119657	Echinacea purpurea	Leaves	C17H22O10 Sinapoyl-glucose	Level 3
5	2.93566	176.1396	2.804677	3.041918	385.115082	75471.7909	12849.00305	0.2372417	INF		No Filter	Trace27	12.96	9.38	Automated - ICIS	119657	Echinacea purpurea	Leaves	C17H22O10 Sinapoyl-glucose	Level 3
1	1.1898373	71.390238	1.180329	1.202049	339.072723	1290.36567	2138.938248	0.0217208	INF		No Filter	Trace28	5.44	5.96	Automated - Genesis	119657	Echinacea purpurea	Leaves		
2	1.2871364	77.228184	1.276872	1.297401	339.071991	1176.19424	2253.147023	0.0205289	INF		No Filter	Trace28	4.96	6.28	Automated - Genesis	119657	Echinacea purpurea	Leaves	C15H16O9 z sinapoyl-malate	Level 4
3	1.5435311	92.611866	1.533262	1.553795	339.072632	1061.84597	2033.467993	0.0205327	INF		No Filter	Trace28	4.48	5.67	Automated - Genesis	119657	Echinacea purpurea	Leaves		
4	2.2320009	133.92005	2.221736	2.242283	339.073181	971.580129	1860.758851	0.0205465	INF		No Filter	Trace28	4.1	5.19	Automated - Genesis	119657	Echinacea purpurea	Leaves		
5	2.6635658	159.81395	2.649625	2.677507	339.07373	1227.92051	1731.555328	0.0278816	INF		No Filter	Trace28	5.18	4.83	Automated - Genesis	119657	Echinacea purpurea	Leaves		
6	2.7271543	163.62926	2.713214	2.741095	339.071411	1260.34985	1777.294051	0.0278813	INF		No Filter	Trace28	5.31	4.95	Automated - Genesis	119657	Echinacea purpurea	Leaves		
7	2.9286897	175.72138	2.914749	2.943475	339.073273	1126.55684	1577.511869	0.028726	INF		No Filter	Trace28	4.75	4.4	Automated - Genesis	119657	Echinacea purpurea	Leaves		
8	3.5838577	215.03146	3.573117	3.594598	339.071716	1274.6465	2332.986483	0.0214813	INF		No Filter	Trace28	5.37	6.5	Automated - Genesis	119657	Echinacea purpurea	Leaves		
9	3.6892893	221.35736	3.678549	3.701633	339.072998	1147.33907	1800.844172	0.0230845	INF		No Filter	Trace28	4.84	5.02	Automated - Genesis	119657	Echinacea purpurea	Leaves		
10	4.1389945	248.33967	4.128762	4.149227	339.071411	1014.3622	1949.353343	0.0204655	INF		No Filter	Trace28	4.28	5.43	Automated - Genesis	119657	Echinacea purpurea	Leaves		
11	4.2826799	256.96079	4.268508	4.30592	0	3203.43227	3399.07774	0.0374119	INF		No Filter	Trace28	13.5	9.48	Automated - Genesis	119657	Echinacea purpurea	Leaves		
12	4.3263858	259.58315	4.316152	4.336617	339.07251	1046.19909	2010.106012	0.0204655	INF		No Filter	Trace28	4.41	5.6	Automated - Genesis	119657	Echinacea purpurea	Leaves		
13	4.3791763	262.75058	4.368944	4.389409	339.072876	1188.29901	2283.669646	0.0204645	INF		No Filter	Trace28	5.01	6.37	Automated - Genesis	119657	Echinacea purpurea	Leaves		
14	4.4318624	265.91174	4.421122	4.444208	339.072083	1089.77199	1960.595276	0.023086	INF		No Filter	Trace28	4.59	5.47	Automated - Genesis	119657	Echinacea purpurea	Leaves		
15	4.5281529	271.68917	4.505071	4.538893	339.072174	3829.37328	3439.782846	0.0338222	INF		No Filter	Trace28	16.14	9.59	Automated - Genesis	119657	Echinacea purpurea	Leaves		
16	4.5603745	273.62247	4.549634	4.571116	339.071472	965.060766	1766.3685	0.0214814	INF		No Filter	Trace28	4.07	4.92	Automated - Genesis	119657	Echinacea purpurea	Leaves		
17	8.2487732	494.92639	8.238033	8.259514	339.071381	851.322295	1558.20651	0.0214812	INF		No Filter	Trace28	3.59	4.34	Automated - Genesis	119657	Echinacea purpurea	Leaves		
1	0.7228652	43.371912	0.714904	0.731926	335.078766	6117.25368	14567.55545	0.01702215	INF		No Filter	Trace29	12.73	41.34	Automated - Genesis	119657	Echinacea purpurea	Leaves		
2	2.3943905	143.66343	2.386466	2.403103	335.076843	906.048085	2168.130978	0.016637	INF		No Filter	Trace29	1.89	6.15	Automated - Genesis	119657	Echinacea purpurea	Leaves		
3	2.4379551	146.27731	2.426338	2.450425	335.075806	1368.49099	2225.537148	0.0240876	INF		No Filter	Trace29	2.85	6.32	Automated - Genesis	119657	Echinacea purpurea	Leaves		
4	2.6170963	157.02578	2.576747	2.692302	335.077881	36257.4594	10704.36396	0.1155552	INF		No Filter	Trace29	75.44	30.38	Automated - Genesis	119657	Echinacea purpurea	Leaves	C16H16O8 caffeoyl-shikimate	Level 4
5	2.7225075	163.35045	2.71089	2.736448	335.076996	3411.24327	5572.982807	0.0255579	INF		No Filter	Trace29	7.1	15.82	Automated - Genesis	119657	Echinacea purpurea	Leaves		
1	0.7640887	45.845321	0.753789	0.774387	137.023743	4063.21577	7757.354936	0.0205979	INF		No Filter	Trace1	20.9	26.16	Automated - Genesis	119658	Echinacea purpurea	Flowers		
2	0.8944613	53.667676	0.884148	0.904762	137.023773	2361.76891	4503.090293	0.02061382	INF		No Filter	Trace1	12.15	15.18	Automated - Genesis	119658	Echinacea purpurea	Flowers		
3	5.4473714	326.84228	5.43503	5.458112	137.023773	859.399922	1508.398398	0.0230817	INF		No Filter	Trace1	4.42	5.09	Automated - Genesis	119658	Echinacea purpurea	Flowers		
4	8.0639397	483.83638	8.053199	8.074681	137.023743	1795.66435	3286.633148	0.0214813	INF		No Filter	Trace1	9.24	11.08	Automated - Genesis	119658	Echinacea purpurea	Flowers		
5	9.8616494	591.69896	9.846865	9.87559	137.023727	2671.34827	3574.804308	0.0287256	INF		No Filter	Trace1	13.74	12.05	Automated - Genesis	119658	Echinacea purpurea	Flowers		
6	9.9484702	596.90821	9.933677	9.962411	137.023788	2601.04074	3604.519273	0.0287339	INF		No Filter	Trace1	13.38	12.15	Automated - Genesis	119658	Echinacea purpurea	Flowers		
7	11.008867	660.53202	10.99493	11.02281	137.023849	1098.84606	1549.577609	0.027882	INF		No Filter	Trace1	5.65	5.22	Automated - Genesis	119658	Echinacea purpurea	Flowers		
8	11.433651	686.01906	11.41971	11.44844	137.024017	1315.81907	1793.937903	0.028728	INF		No Filter	Trace1	6.77	6.05	Automated - Genesis	119658	Echinacea purpurea	Flowers		
9	12.83054	769.8324	12.8166	12.86857	137.023727	2672.16599	2079.552856	0.051968	INF		No Filter	Trace1	13.75	7.01	Automated - Genesis	119658	Echinacea purpurea	Flowers		
1	4.6180343	277.08206	4.607802	4.628266	89.039917	244.215215	469.488373	0.0204638	INF		No Filter	Trace2	100	100	Automated - Genesis	119658	Echinacea purpurea	Flowers		
1	1.9101302	114.60781	1.706774	1.962279	137.023285	160805.135	17963.65596	0.255505091		7.146510084	No Filter	Trace3	17.76	22.4	Manual	119658	Echinacea purpurea	Flowers		
2	5.6820544	340.92326	5.528467	5.988321	0	744600.573	62216.76714	0.459854015		24.34921719	No Filter	Trace3	82.24	77.6	Manual	119658	Echinacea purpurea	Flowers		
1	0.9174008	55.044047	0.907103	0.937997	93.0331116	1258.97259	1416.127499	0.03089447	INF		No Filter	Trace4	3.91	4.79	Automated - Genesis	119658	Echinacea purpurea	Flowers		
2	1.1777914	70.667484	1.167198	1.187301	93.0327225	340.234585	665.9478226	0.0201031	INF		No Filter	Trace4	1.06	2.25	Automated - Genesis	119658	Echinacea purpurea	Flowers		
3	3.8356061	230.13637	3.825372	3.845838	93.0330124	263.420971	506.3555815	0.0204659	INF		No Filter	Trace4	0.82	1.71	Automated - Genesis	119658	Echinacea purpurea	Flowers		
4	3.8934898	233.60939	3.883258	3.903722	93.033165	339.99272	653.6356036	0.0204636	INF		No Filter	Trace4	1.05	2.21	Automated - Genesis	119658	Echinacea purpurea	Flowers		
5	3.9190701	235.14421	3.908837	3.941134	93.0332489	652.480771	649.3153937	0.0322963	INF		No Filter	Trace4	2.02	2.2	Automated - Genesis	119658	Echinacea purpurea	Flowers		
6	3.9513649	237.08189	3.941134	3.961597	93.0334015	290.791089	559.0262036	0.0204636	INF		No Filter	Trace4	0.9	1.89	Automated - Genesis	119658	Echinacea purpurea	Flowers		
7	3.987176	239.23056	3.976945	3.997412	93.0334473	273.313149	525.2052292	0.0204673	INF		No Filter	Trace4	0.85	1.78	Automated - Genesis	119658	Echinacea purpurea	Flowers		
8	4.0246104	241.47662	4.012775	4.034842	93.0329666	317.907968	520.3606274	0.0220671	INF		No Filter	Trace4	0.99	1.76	Automated - Genesis	119658	Echinacea purpurea	Flowers		
9	4.0553072	243.31843	4.045074	4.065539	93.0334244	246.069879	473.0705178	0.0204646	INF		No Filter	Trace4	0.76	1.6	Automated - Genesis	119658	Echinacea purpurea	Flowers		
10	4.1147677	246.88606	4.10135	4.128582	0	602.889447	859.435348	0.0272318	INF		No Filter	Trace4	1.87	2.91	Automated - Genesis	119658	Echinacea purpurea	Flowers		
11	4.1388143	248.32886	4.128582	4.149046	93.0329666	273.271228	525.3677398	0.0204636	INF		No Filter	Trace4	0.85	1.78	Automated - Genesis	119658	Echinacea purpurea	Flowers		
12	4.1797419	250.78451	4.16951	4.191581	93.0330353	305.776114	585.1444818	0.0220704	INF		No Filter	Trace4	0.95	1.98	Automated - Genesis	119658	Echinacea purpurea	Flowers		
13	4.3057437	258.34462	4.280164	4.315976	93.0330505	626.84177	637.5316462	0.0358125	INF		No Filter	Trace4	1.94	2.16	Automated - Genesis	119658	Echinacea purpurea	Flowers		
14	4.3687365	262.12419	4.346673	4.39432	93.0332642	1979.07643	1337.442029	0.047647	INF		No Filter	Trace4	6.14	4.52	Automated - Genesis	119658	Echinacea purpurea	Flowers		
15	4.4188723	265.13234	4.408132	4.429613	93.0333633	381.418389	698.1282844	0.0214806	INF		No Filter	Trace4	1.18	2.36	Automated - Genesis	119658	Echinacea purpurea	Flowers		
16	4.5112182	270.67309	4.452696	4.543799	0	2893.57165	1042.428478	0.0911032	INF		No Filter	Trace4	8.98	3.53	Automated - Genesis	119658	Echinacea purpurea	Flowers		
17	4.5545394	273.27236	4.543799	4.56528	93.0333328	302.788161	554.1967201	0.0214813	INF		No Filter	Trace4	0.94	1.87	Automated - Genesis	119658	Echinacea purpurea	Flowers		
18	4.5760206	274.56124	4.56528	4.618034	93.0331497	1656.36542	1012.711451	0.0527542	INF		No Filter	Trace4	5.14	3.43	Automated - Genesis	119658	Echinacea purpurea	Flowers		
19	4.6310406	277.86244	4.618034	4.643614	0	683.752776	1014.444122	0.0255793	INF		No Filter	Trace4	2.12	3.43	Automated - Genesis	119658	Echinacea purpurea	Flowers		
20	4.6691937	280.15162	4.658962	4.679425	93.0332489	255.634147	491.4696378	0.0204635	INF		No Filter	Trace4	0.79	1.66	Automated - Genesis	119658	Echinacea purpurea	Flowers		
21	4.6963822	281.78293	4.684542	4.706614	93.033371	330.771738	608.38374	0.0220715	INF		No Filter	Trace4	1.03	2.06	Automated - Genesis	119658	Echinacea purpurea	Flowers		
22	4.7322004	283.93202	4.706614	4.742432	93.0332031	741.250754	909.7748456	0.0358186	INF		No Filter	Trace4	2.3	3.08	Automated - Genesis	119658	Echinacea purpurea	Flowers		
23	4.7554395	285.32637	4.742432	4.768012	0	564.380366	825.2669512	0.0255798	INF		No Filter	Trace4	1.75	2.79	Automated - Genesis	119658	Echinacea purpurea	Flowers		
24	4.8105468	288.63281	4.779851	4.825894	93.0332108	1783.99842	1256.287984	0.0460431	INF		No Filter	Trace4	5.53	4.25	Automated - Genesis	119658	Echinacea purpurea	Flowers		
25	4.8389041	290.33425	4.825894	4.868435	0	1551.48946	1223.591538	0.0425407	INF		No Filter	Trace4	4.81	4.14	Automated - Genesis	119658	Echinacea purpurea	Flowers		
26	4.946789	296.80734	4.878667	4.96214	93.0331726	4128.90592	1705.793933	0.0834729	INF		No Filter	Trace4	12.81	5.77	Automated - Genesis	119658	Echinacea purpurea	Flowers		
27	5.0046234	300.2774	4.992856	5.018435	0	677.22316	1004.518695	0.0255796	INF		No Filter	Trace4	2.1	3.4	Automated - Genesis	119658	Echinacea purpurea	Flowers		
28	5.0404991	302.42995	5.030267	5.060963	93.033226	716.313172	691.9371387	0.0306963	INF		No Filter	Trace4	2.22	2.34	Automated - Genesis	119658	Echinacea purpurea	Flowers		
29	5.0865438	305.19263	5.076312	5.108607	93.03302	625.944069	613.7095863	0.0322952	INF		No Filter	Trace4	1.94	2.08	Automated - Genesis	119658	Echinacea purpurea	Flowers		
30	5.1188418	307.13051	5.108607	5.129091	93.0332947	358.869395	689.6212904	0.0204838	INF		No Filter	Trace4	1.11	2.33	Automated - Genesis	119658	Echinacea purpurea	Flowers		
31	5.1393224	308.35934	5.129091	5.149555	93.0334244	380.7166	731.9409557	0.0204646	INF		No Filter	Trace4	1.18	2.48	Automated - Genesis	119658	Echinacea purpurea	Flowers		
32	5.2050979	312.30587	5.175135	5.28587	0	3461.37195	1202.26466	0.1107352	INF		No Filter	Trace4	10.74	4.07	Automated - Genesis	119658	Echinacea purpurea	Flowers		
33	5.314225	318.8535	5.28587	5.326798	0	1004.30091	1037.449377	0.0409277	INF		No Filter	Trace4	3.12	3.51	Automated - Genesis	119658	Echinacea purpurea	Flowers		
34	5.3551526	321.30916	5.342145	5.369336	0	734.092828	941.8808231	0.0271909	INF		No Filter	Trace4	2.28	3.19	Automated - Genesis	119658	Echinacea purpurea	Flowers		
35	5.3913367	323.4802	5.369336	5.405148	0	1231.46515	1390.512777	0.0358125	INF		No Filter	Trace4	3.82	4.7	Automated - Genesis	119658	Echinacea purpurea	Flowers		

1	0.9408054	56.448324	0.930507	0.952726	225.04155	2586.60774	4201.725153	0.0222191	INF	No Filter	Trace5	0.92	4.78	Automated - ICIS	119658	Echinacea purpurea	Flowers		
2	1.0854439	65.126634	1.036756	1.140391	0	29415.478	11293.69437	0.1036349	INF	No Filter	Trace5	10.42	12.85	Automated - ICIS	119658	Echinacea purpurea	Flowers		
3	1.1604967	69.629802	1.140391	1.207463	225.039658	13973.0533	5885.477537	0.0670719	INF	No Filter	Trace5	4.95	6.7	Automated - ICIS	119658	Echinacea purpurea	Flowers		
4	1.2354821	74.128926	1.207463	1.296352	0	23418.8344	6381.576617	0.088889	INF	No Filter	Trace5	8.3	7.26	Automated - ICIS	119658	Echinacea purpurea	Flowers		
5	1.3243547	79.461282	1.296352	1.35281	0	9159.81237	4183.810556	0.0564578	INF	No Filter	Trace5	3.25	4.76	Automated - ICIS	119658	Echinacea purpurea	Flowers		
6	1.3721671	82.330026	1.357943	1.410886	0	5405.59982	4040.956226	0.0529429	INF	No Filter	Trace5	1.92	4.6	Automated - ICIS	119658	Echinacea purpurea	Flowers		
7	1.4211679	85.270074	1.410886	1.431434	225.040283	1393.34968	2664.718743	0.0205479	INF	No Filter	Trace5	0.49	3.03	Automated - ICIS	119658	Echinacea purpurea	Flowers		
8	1.4587187	87.523122	1.436566	1.468984	225.039795	2531.26871	3025.942697	0.0324183	INF	No Filter	Trace5	0.9	3.44	Automated - ICIS	119658	Echinacea purpurea	Flowers		
9	1.4792493	88.754958	1.468984	1.489514	225.039566	1247.92933	2390.506603	0.0205296	INF	No Filter	Trace5	0.44	2.72	Automated - ICIS	119658	Echinacea purpurea	Flowers		
10	1.5049143	90.294858	1.494647	1.515178	225.040482	826.293312	1582.873279	0.0205316	INF	No Filter	Trace5	0.29	1.8	Automated - ICIS	119658	Echinacea purpurea	Flowers		
11	1.6170415	97.02249	1.597707	1.627618	225.040207	1788.66128	1785.772757	0.0299104	INF	No Filter	Trace5	0.63	2.03	Automated - ICIS	119658	Echinacea purpurea	Flowers		
12	1.8656739	111.94043	1.852234	1.89397	0	3081.18842	2443.842468	0.041736	INF	No Filter	Trace5	1.09	2.78	Automated - ICIS	119658	Echinacea purpurea	Flowers		
13	1.9585656	117.51394	1.949088	1.969142	225.040497	713.971771	1398.683093	0.0200541	INF	No Filter	Trace5	0.25	1.59	Automated - ICIS	119658	Echinacea purpurea	Flowers		
14	2.0326415	121.95849	2.02076	2.042906	225.04068	956.461624	1823.767672	0.0221458	INF	No Filter	Trace5	0.34	2.08	Automated - ICIS	119658	Echinacea purpurea	Flowers	Isochorismate C10H10O6	Level 4
15	2.5454693	152.72816	2.531529	2.568704	225.039993	2148.67301	1820.187192	0.0371757	INF	No Filter	Trace5	0.76	2.07	Automated - ICIS	119658	Echinacea purpurea	Flowers		
16	2.6624922	159.74953	2.568704	2.869511	225.039948	160002.678	18159.35095	0.3008071	INF	No Filter	Trace5	56.7	20.67	Automated - ICIS	119658	Echinacea purpurea	Flowers	Chorismate C10H10O6	Level 4
17	2.9020398	174.12239	2.869511	2.963297	225.039917	14621.955	5553.693969	0.0937854	INF	No Filter	Trace5	5.18	6.32	Automated - ICIS	119658	Echinacea purpurea	Flowers		
18	3.0004721	180.02833	2.986532	3.014413	225.039551	1237.94067	1745.667738	0.0278816	INF	No Filter	Trace5	0.44	1.99	Automated - ICIS	119658	Echinacea purpurea	Flowers		
19	3.0501409	183.00845	3.01906	3.078023	225.040024	4785.6185	2303.957268	0.0589625	INF	No Filter	Trace5	1.7	2.62	Automated - ICIS	119658	Echinacea purpurea	Flowers		
20	3.3059303	198.35582	3.29519	3.316671	225.040665	924.244209	1691.663147	0.0214813	INF	No Filter	Trace5	0.33	1.93	Automated - ICIS	119658	Echinacea purpurea	Flowers	Prephenate C10H10O6	Level 4
21	3.7186205	223.11723	3.70788	3.729361	225.039719	1058.59891	1937.572331	0.0214812	INF	No Filter	Trace5	0.38	2.21	Automated - ICIS	119658	Echinacea purpurea	Flowers		
22	6.1020861	366.12517	6.091345	6.114439	225.04155	921.18477	1554.017422	0.0230943	INF	No Filter	Trace5	0.33	1.77	Automated - ICIS	119658	Echinacea purpurea	Flowers		
1	0.677943	40.676577	0.6219	1.093401	174.076584	5135349.12	1451712.754	0.47150173	187.0853585	No Filter	Trace6	100	100	Automated - Genesis	119658	Echinacea purpurea	Flowers	C15H17NO9 Isochorismate-9-glutamate	Level 2
1	2.4981536	149.88922	2.411679	2.881752	179.034149	267818.335	37276.47652	0.470072993	INF	No Filter	Trace7	100	100	Manual	119658	Echinacea purpurea	Flowers		
1	0.6218996	37.313974	0.602291	0.6398	223.060822	15759.3604	15149.89264	0.03750878	INF	No Filter	Trace9	4.95	17.86	Automated - Genesis	119658	Echinacea purpurea	Flowers		
2	0.9784894	58.709366	0.968173	0.988789	223.061356	1741.47808	3319.884375	0.02061555	INF	No Filter	Trace9	0.55	3.91	Automated - Genesis	119658	Echinacea purpurea	Flowers		
3	1.2421675	74.53005	1.227992	1.253671	0	2499.39157	3619.335612	0.0256792	INF	No Filter	Trace9	0.79	4.27	Automated - Genesis	119658	Echinacea purpurea	Flowers		
4	1.5515119	93.090714	1.494647	1.644091	0	37232.1303	9993.336758	0.1494441	INF	No Filter	Trace9	11.7	11.78	Automated - Genesis	119658	Echinacea purpurea	Flowers		
5	2.4865364	149.19218	2.470272	2.524558	0	4226.15436	3095.093351	0.0542863	INF	No Filter	Trace9	1.33	3.65	Automated - Genesis	119658	Echinacea purpurea	Flowers		
6	2.6694627	160.16776	2.611376	2.685727	0	6387.37214	3324.439559	0.0743513	INF	No Filter	Trace9	2.01	3.92	Automated - Genesis	119658	Echinacea purpurea	Flowers	C11H12O5 Z-Sinapic acid	Level 4
7	2.8857756	173.14654	2.801277	3.068729	223.060562	197290.339	30323.41698	0.2674521	INF	No Filter	Trace9	62.02	35.76	Automated - Genesis	119658	Echinacea purpurea	Flowers		
8	3.174093	190.44558	3.109036	3.211234	223.061325	16955.913	8726.99437	0.1021984	INF	No Filter	Trace9	5.33	10.29	Automated - Genesis	119658	Echinacea purpurea	Flowers		
9	4.2955118	257.73071	4.225052	4.371513	0	36013.7749	7251.989566	0.1464612	INF	No Filter	Trace9	11.32	8.55	Automated - Genesis	119658	Echinacea purpurea	Flowers		
1	0.8120086	48.720517	0.804051	0.821068	353.089325	35193.5578	67888.69372	0.017017	5.626436739	No Filter	Trace10	8.25	46.7	Automated - Genesis	119658	Echinacea purpurea	Flowers		
2	1.1805997	70.835982	1.115905	1.492784	353.086823	78041.9584	16548.38884	0.3768785	INF	No Filter	Trace10	18.29	11.38	Manual	119658	Echinacea purpurea	Flowers	C16H18O9 neochlorogenic acid (3-o-caffeoylquinic acid)	Level 4
3	1.5977072	95.862432	1.532847	1.665693	0	121753.075	33642.00877	0.132846715	INF	No Filter	Trace10	28.53	23.14	Manual	119658	Echinacea purpurea	Flowers	C16H18O9 chlorogenic acid (5-o-caffeoylquinic acid)	Level 4
4	1.7257655	103.54593	1.675912	1.982482	353.087189	173457.137	21015.28097	0.306569343	INF	No Filter	Trace10	40.64	14.45	Manual	119658	Echinacea purpurea	Flowers	C16H18O9 cis 5-o-caffeoylquinic acid	Level 4
5	2.7850125	167.10075	2.268613	2.922628	353.086823	18332.6633	6293.0499	0.654014599	INF	No Filter	Trace10	4.3	4.33	Manual	119658	Echinacea purpurea	Flowers	C16H18O9 4-o-caffeoylquinic acid (crytochlorogenic acid)	Level 4
1	4.6822016	280.9321	4.629355	4.792708	515.118896	76328.5165	14885.50558	0.163353	INF	No Filter	Trace11	11.7	10.69	Manual	119658	Echinacea purpurea	Flowers	C25H24O12 3.4-dicaffeolquinic acid	Level 4
2	4.8542656	291.25594	4.813171	5.105792	515.118164	104024.066	14957.75657	0.2926208	INF	No Filter	Trace11	15.94	10.75	Manual	119658	Echinacea purpurea	Flowers	C25H24O12 3,5-dicaffeolquinic acid	Level 4
3	5.1790233	310.7414	5.157318	5.236896	515.120972	25126.4868	8913.711096	0.0795779	INF	No Filter	Trace11	3.85	6.4	Automated - ICIS	119658	Echinacea purpurea	Flowers		
4	5.2676116	316.0567	5.236896	5.330608	515.119812	16697.4539	5661.903645	0.0937122	INF	No Filter	Trace11	2.56	4.07	Automated - ICIS	119658	Echinacea purpurea	Flowers		
5	5.3510724	321.06434	5.330608	5.35619	515.121643	6546.08061	7768.475577	0.0255812	INF	No Filter	Trace11	1	5.58	Automated - ICIS	119658	Echinacea purpurea	Flowers		
6	5.4146272	324.87763	5.35619	5.648559	515.120239	347364.602	51931.35089	0.2923693	INF	No Filter	Trace11	53.23	37.31	Automated - ICIS	119658	Echinacea purpurea	Flowers	C25H24O12 4,5-dicaffeolquinic acid	Level 4
7	5.7026203	342.15722	5.66646	5.725341	0	17439.4415	6624.974759	0.058881	INF	No Filter	Trace11	2.67	4.76	Automated - ICIS	119658	Echinacea purpurea	Flowers		
8	5.7539826	345.23896	5.725341	5.770644	515.120239	10977.8009	6481.290754	0.045303	INF	No Filter	Trace11	1.68	4.66	Automated - ICIS	119658	Echinacea purpurea	Flowers		
9	5.7937293	347.62376	5.770644	5.829246	0	13056.4113	7172.927705	0.0586025	INF	No Filter	Trace11	2	5.15	Automated - ICIS	119658	Echinacea purpurea	Flowers		
10	5.8497102	350.98261	5.829246	5.885892	515.120056	10911.1319	4794.418327	0.0566456	INF	No Filter	Trace11	1.67	3.44	Automated - ICIS	119658	Echinacea purpurea	Flowers		
11	5.9859475	359.15685	5.915055	5.998521	515.1203	13547.3381	4117.948166	0.0834659	INF	No Filter	Trace11	2.08	2.96	Automated - ICIS	119658	Echinacea purpurea	Flowers		
12	6.0191964	361.15178	5.998521	6.069688	515.11969	10511.7717	5877.420261	0.0711674	INF	No Filter	Trace11	1.61	4.22	Automated - ICIS	119658	Echinacea purpurea	Flowers		
1	2.4392193	146.35316	2.425279	2.454008	311.040924	1165.35806	1539.194112	0.0287292	INF	No Filter	Trace12	7.69	10.1	Automated - Genesis	119658	Echinacea purpurea	Flowers		
2	3.9167297	235.00378	3.906497	3.926962	311.04007	1782.49902	3425.599403	0.0204652	INF	No Filter	Trace12	11.76	22.47	Automated - Genesis	119658	Echinacea purpurea	Flowers		
3	3.9733642	238.40185	3.932078	3.984835	0	10205.9189	6423.502872	0.0527576	INF	No Filter	Trace12	67.32	42.14	Automated - Genesis	119658	Echinacea purpurea	Flowers		
4	4.073431	244.40586	4.063198	4.083662	311.040314	2005.81421	3854.772185	0.0204641	INF	No Filter	Trace12	13.23	25.29	Automated - Genesis	119658	Echinacea purpurea	Flowers		
1	0.6398002	38.388014	0.62906	0.650541	473.074249	5983.42258	10951.7289	0.02148123	INF	No Filter	Trace13	6.59	9.06	Automated - Genesis	119658	Echinacea purpurea	Flowers	C22H18O12 cichoric acid	Level 4
2	2.5454693	152.72816	2.531529	2.559411	473.073456	1374.15293	1937.748184	0.027882	INF	No Filter	Trace13	1.51	1.6	Automated - Genesis	119658	Echinacea purpurea	Flowers		
3	2.5989095	155.93457	2.584969	2.6137	473.073792	1296.34216	1796.607377	0.0287308	INF	No Filter	Trace13	1.43	1.49	Automated - Genesis	119658	Echinacea purpurea	Flowers		
4	2.840776	170.44656	2.810571	2.854717	473.073914	2501.04718	1807.344597	0.044146	INF	No Filter	Trace13	2.76	1.5	Automated - Genesis	119658	Echinacea purpurea	Flowers		
5	2.8997167	173.983	2.885776	2.913658	473.07312	1661.158	2342.513464	0.0278819	INF	No Filter	Trace13	1.83	1.94	Automated - Genesis	119658	Echinacea purpurea	Flowers		
6	3.1531816	189.1909	3.139241	3.167123	473.071228	1939.35922	2734.776274	0.0278817	INF	No Filter	Trace13	2.14	2.26	Automated - Genesis	119658	Echinacea purpurea	Flowers		
7	4.9377192	296.26315	4.927487	4.949564	473.072723	1068.35734	1746.424137	0.0220776	INF	No Filter	Trace13	1.18	1.44	Automated - Genesis	119658	Echinacea purpurea	Flowers		
8	6.3109905	378.65943	6.30025	6.321731	473.074829	1560.72935	2856.659812	0.0214813	INF	No Filter	Trace13	1.72	2.36	Automated - Genesis	119658	Echinacea purpurea	Flowers		
9	6.3591451	381.54871	6.346793	6.369886	473.073975	1449.55096	2341.016408	0.023093	INF	No Filter	Trace13	1.6	1.94	Automated - Genesis	119658	Echinacea purpurea	Flowers		
10	6.4967919	389.80751	6.486051	6.507533	473.074554	1029.77186	1884.819062	0.0214812	INF	No Filter	Trace13	1.13	1.56	Automated - Genesis	119658	Echinacea purpurea	Flowers		
11	6.5628409	393.77045	6.5521	6.573582	473.071533	1082.87771	1982.00329	0.0214813	INF	No Filter	Trace13	1.19	1.64	Automated - Genesis	119658	Echinacea purpurea	Flowers		
12	6.9503503	417.02102	6.93801	6.961091	473.07193	1072.86469	1955.093721	0.0230805	INF	No Filter	Trace13	1.18	1.62	Automated - Genesis	119658	Echinacea purpurea	Flowers		
13	7.1043967	426.2638	7.094165	7.116233	473.071716	1305.01566	2135.080612	0.0220683	INF	No Filter	Trace13	1.44	1.77	Automated - Genesis	119658	Echinacea purpurea	Flowers		
14	7.1878595	431.27157	7.177626	7.199776	473.074524	1042.6233	1693.041559	0.02215	INF	No Filter	Trace13	1.15	1.4	Automated - Genesis	119658	Echinacea purpurea	Flowers		

15	7.735295	464.1177	7.724555	7.746036	473.073792	763.999199	1398.350969	0.0214812	INF	No Filter	Trace13	0.84	1.16	Automated - Genesis	119658	Echinacea purpurea	Flowers		
16	7.8120867	468.7252	7.801346	7.822827	473.072968	852.743797	1560.784602	0.0214812	INF	No Filter	Trace13	0.94	1.29	Automated - Genesis	119658	Echinacea purpurea	Flowers		
17	8.0639397	483.83638	8.053199	8.074681	473.072906	966.259783	1768.560713	0.0214813	INF	No Filter	Trace13	1.06	1.46	Automated - Genesis	119658	Echinacea purpurea	Flowers		
18	8.0890013	485.34008	8.078261	8.099742	473.071899	973.223008	1781.312757	0.0214812	INF	No Filter	Trace13	1.07	1.47	Automated - Genesis	119658	Echinacea purpurea	Flowers		
19	8.2871563	497.22938	8.276416	8.297897	473.07251	895.744244	1639.497143	0.0214813	INF	No Filter	Trace13	0.99	1.36	Automated - Genesis	119658	Echinacea purpurea	Flowers		
20	8.4622143	507.73286	8.451474	8.472955	473.074738	914.42625	1673.684462	0.0214813	INF	No Filter	Trace13	1.01	1.38	Automated - Genesis	119658	Echinacea purpurea	Flowers		
21	8.5159173	510.95504	8.505177	8.52827	473.07312	969.721122	1635.948018	0.0230935	INF	No Filter	Trace13	1.07	1.35	Automated - Genesis	119658	Echinacea purpurea	Flowers		
22	8.8195096	529.17058	8.808769	8.83025	473.070465	1014.05619	1856.040764	0.0214812	INF	No Filter	Trace13	1.12	1.54	Automated - Genesis	119658	Echinacea purpurea	Flowers		
23	8.860494	531.62964	8.848151	8.871235	473.07196	1175.67697	1899.968161	0.0230841	INF	No Filter	Trace13	1.3	1.57	Automated - Genesis	119658	Echinacea purpurea	Flowers		
24	8.9070369	534.42221	8.896296	8.917777	473.070374	1540.06716	2818.812981	0.0214813	INF	No Filter	Trace13	1.7	2.33	Automated - Genesis	119658	Echinacea purpurea	Flowers		
25	8.9838226	539.02936	8.973083	8.994564	473.074249	960.181043	1757.442223	0.0214813	INF	No Filter	Trace13	1.06	1.45	Automated - Genesis	119658	Echinacea purpurea	Flowers		
26	9.1088515	546.53109	9.096415	9.119592	473.074585	979.957435	1572.970211	0.023177	INF	No Filter	Trace13	1.08	1.3	Automated - Genesis	119658	Echinacea purpurea	Flowers		
27	9.5840951	575.04571	9.570154	9.598036	473.072235	1444.70233	2037.263235	0.0278819	INF	No Filter	Trace13	1.59	1.69	Automated - Genesis	119658	Echinacea purpurea	Flowers		
28	9.8445412	590.67247	9.830601	9.859326	473.071625	1523.46051	2144.492406	0.0287254	INF	No Filter	Trace13	1.68	1.77	Automated - Genesis	119658	Echinacea purpurea	Flowers		
29	10.049229	602.95374	10.03529	10.06317	473.07486	1936.44913	2730.699398	0.027882	INF	No Filter	Trace13	2.13	2.26	Automated - Genesis	119658	Echinacea purpurea	Flowers		
30	10.175555	610.5333	10.16161	10.19267	473.072815	2977.14019	3992.182356	0.031057	INF	No Filter	Trace13	3.28	3.3	Automated - Genesis	119658	Echinacea purpurea	Flowers		
31	10.750741	645.04446	10.7368	10.76468	473.071289	1712.42497	2414.814274	0.027882	INF	No Filter	Trace13	1.89	2	Automated - Genesis	119658	Echinacea purpurea	Flowers		
32	10.80968	648.5808	10.79574	10.82362	473.070892	1305.26642	1840.602351	0.027882	INF	No Filter	Trace13	1.44	1.52	Automated - Genesis	119658	Echinacea purpurea	Flowers		
33	10.875584	652.53504	10.84918	10.91508	473.073334	5943.77505	2704.86714	0.065904	INF	No Filter	Trace13	6.55	2.24	Automated - Genesis	119658	Echinacea purpurea	Flowers		
34	11.015837	660.95022	11.0019	11.03062	473.073761	1216.10725	1685.55532	0.028725	INF	No Filter	Trace13	1.34	1.39	Automated - Genesis	119658	Echinacea purpurea	Flowers		
35	11.046886	662.81316	11.03062	11.0678	0	2460.31597	2253.313277	0.037175	INF	No Filter	Trace13	2.71	1.86	Automated - Genesis	119658	Echinacea purpurea	Flowers		
36	11.140672	668.44032	11.12673	11.15461	473.073486	1377.18524	1942.017615	0.027882	INF	No Filter	Trace13	1.52	1.61	Automated - Genesis	119658	Echinacea purpurea	Flowers		
37	11.239106	674.34636	11.22517	11.25305	473.071838	1634.86724	2305.398458	0.027882	INF	No Filter	Trace13	1.8	1.91	Automated - Genesis	119658	Echinacea purpurea	Flowers		
38	11.513496	690.80976	11.49955	11.52828	473.071228	1271.40701	1780.425044	0.028724	INF	No Filter	Trace13	1.4	1.47	Automated - Genesis	119658	Echinacea purpurea	Flowers		
39	11.788724	707.32344	11.77478	11.80267	473.074463	1113.45286	1570.122579	0.027881	INF	No Filter	Trace13	1.23	1.3	Automated - Genesis	119658	Echinacea purpurea	Flowers		
40	11.851458	711.08748	11.83752	11.86625	473.071259	1609.16516	2193.733219	0.028729	INF	No Filter	Trace13	1.77	1.81	Automated - Genesis	119658	Echinacea purpurea	Flowers		
41	11.948412	716.90472	11.93363	11.96235	473.074341	1274.7	1766.805445	0.028726	INF	No Filter	Trace13	1.4	1.46	Automated - Genesis	119658	Echinacea purpurea	Flowers		
42	12.022763	721.36578	12.00882	12.03755	473.073456	1271.13392	1679.138214	0.028728	INF	No Filter	Trace13	1.4	1.39	Automated - Genesis	119658	Echinacea purpurea	Flowers		
43	12.349124	740.94744	12.33518	12.36392	473.072449	1241.07446	1720.007763	0.028732	INF	No Filter	Trace13	1.37	1.42	Automated - Genesis	119658	Echinacea purpurea	Flowers		
44	12.581707	754.90242	12.56777	12.59565	473.071594	1384.58321	1952.511213	0.027881	INF	No Filter	Trace13	1.53	1.62	Automated - Genesis	119658	Echinacea purpurea	Flowers		
45	12.622059	757.32354	12.60494	12.66621	0	4068.04154	2397.331824	0.061263	INF	No Filter	Trace13	4.48	1.98	Automated - Genesis	119658	Echinacea purpurea	Flowers		
46	12.680146	760.80876	12.66621	12.69493	473.070251	1364.98122	1911.375389	0.028726	INF	No Filter	Trace13	1.5	1.58	Automated - Genesis	119658	Echinacea purpurea	Flowers		
47	12.774776	766.48656	12.75999	12.78872	473.07019	1293.0134	1707.845096	0.028729	INF	No Filter	Trace13	1.42	1.41	Automated - Genesis	119658	Echinacea purpurea	Flowers		
48	12.856097	771.36582	12.84216	12.87089	473.070557	1326.34278	1751.431537	0.028732	INF	No Filter	Trace13	1.46	1.45	Automated - Genesis	119658	Echinacea purpurea	Flowers		
49	12.953052	777.18312	12.93595	12.96932	0	2653.33871	3308.444852	0.03337	INF	No Filter	Trace13	2.92	2.74	Automated - Genesis	119658	Echinacea purpurea	Flowers		
50	13.081691	784.90146	13.06775	13.09563	473.074524	1227.61545	1731.107631	0.027881	INF	No Filter	Trace13	1.35	1.43	Automated - Genesis	119658	Echinacea purpurea	Flowers		
51	13.20189	792.1134	13.1871	13.21583	473.073883	1152.58661	1613.830256	0.028735	INF	No Filter	Trace13	1.27	1.33	Automated - Genesis	119658	Echinacea purpurea	Flowers		
52	13.424317	805.45902	13.40108	13.4484	0	5363.35044	3672.389221	0.047315	INF	No Filter	Trace13	5.91	3.04	Automated - Genesis	119658	Echinacea purpurea	Flowers		
53	13.659201	819.55206	13.64526	13.67314	473.072052	1785.22564	2517.390246	0.027881	INF	No Filter	Trace13	1.97	2.08	Automated - Genesis	119658	Echinacea purpurea	Flowers		
54	13.905702	834.34212	13.89176	13.91964	473.072388	1445.57935	2038.491904	0.027881	INF	No Filter	Trace13	1.59	1.69	Automated - Genesis	119658	Echinacea purpurea	Flowers		
1	6.0256618	361.53971	6.01098	6.037642	0	2671.00865	3781.461813	0.0266615	INF	No Filter	Trace14	0.85	6.2	Automated - Genesis	119658	Echinacea purpurea	Flowers		
2	6.5146931	390.88159	6.503952	6.527039	301.036224	1268.32353	2281.797271	0.0230865	INF	No Filter	Trace14	0.4	3.74	Automated - Genesis	119658	Echinacea purpurea	Flowers		
3	6.719994	403.19964	6.689751	6.730735	301.036469	2098.17086	2058.699662	0.0409843	INF	No Filter	Trace14	0.67	3.37	Automated - Genesis	119658	Echinacea purpurea	Flowers		
4	7.1288068	427.72841	7.015821	7.608387	0	271339.788	20298.08504	0.5925655	INF	No Filter	Trace14	86.46	33.26	Automated - Genesis	119658	Echinacea purpurea	Flowers	C15H10O7 quercetin	Level 4
5	7.6429489	458.57693	7.611967	7.65851	0	7017.20396	6122.559679	0.0465428	INF	No Filter	Trace14	2.24	10.03	Automated - Genesis	119658	Echinacea purpurea	Flowers		
6	7.6715906	460.29544	7.65851	7.731715	0	7921.17335	3761.374296	0.0732055	INF	No Filter	Trace14	2.52	6.16	Automated - Genesis	119658	Echinacea purpurea	Flowers		
7	7.7750448	466.50269	7.760357	7.847889	0	7845.74208	3478.940447	0.0875321	INF	No Filter	Trace14	2.5	5.7	Automated - Genesis	119658	Echinacea purpurea	Flowers		
8	7.9055376	474.33226	7.863815	7.917518	0	4474.63849	3773.989067	0.053703	INF	No Filter	Trace14	1.43	6.18	Automated - Genesis	119658	Echinacea purpurea	Flowers		
9	7.960849	477.65094	7.947768	7.972829	0	1962.97723	3187.163111	0.0250611	INF	No Filter	Trace14	0.63	5.22	Automated - Genesis	119658	Echinacea purpurea	Flowers		
10	8.0662802	483.97681	8.053199	8.078261	0	1992.49368	3235.048969	0.0250614	INF	No Filter	Trace14	0.63	5.3	Automated - Genesis	119658	Echinacea purpurea	Flowers		
11	8.0997418	485.98451	8.089001	8.112089	301.035522	1278.26199	2157.100465	0.0230875	INF	No Filter	Trace14	0.41	3.53	Automated - Genesis	119658	Echinacea purpurea	Flowers		
12	8.1228293	487.36976	8.112089	8.13357	301.034393	1170.08039	2141.623656	0.0214812	INF	No Filter	Trace14	0.37	3.51	Automated - Genesis	119658	Echinacea purpurea	Flowers		
13	8.2712227	496.27336	8.260482	8.283576	301.034302	1373.79784	2154.480329	0.0230937	INF	No Filter	Trace14	0.44	3.53	Automated - Genesis	119658	Echinacea purpurea	Flowers		
14	8.4212262	505.27357	8.410485	8.431967	301.036621	1419.88402	2598.830047	0.0214815	INF	No Filter	Trace14	0.45	4.26	Automated - Genesis	119658	Echinacea purpurea	Flowers		
1	7.1083425	426.50055	7.017155	7.796801	0	913771.887	100809.2659	0.7796454	INF	No Filter	Trace15	55.76	41.88	Manual	119658	Echinacea purpurea	Flowers	C15H10O6 Luteolin	Level 4
2	7.922474	475.34844	7.86161	7.993346	0	33409.2741	6862.85209	0.1317357	INF	No Filter	Trace15	2.04	2.85	Automated - ICIS	119658	Echinacea purpurea	Flowers		
3	8.0100073	480.60044	7.993346	8.150506	0	32513.721	7504.819856	0.1571597	INF	No Filter	Trace15	1.98	3.12	Automated - ICIS	119658	Echinacea purpurea	Flowers		
4	8.2511136	495.06682	8.150506	8.263094	0	29541.9254	7313.547517	0.1125883	INF	No Filter	Trace15	1.8	3.04	Automated - ICIS	119658	Echinacea purpurea	Flowers		
5	8.3665486	501.99292	8.263094	8.408272	285.040253	44378.4009	7341.053761	0.1451776	INF	No Filter	Trace15	2.71	3.05	Automated - ICIS	119658	Echinacea purpurea	Flowers		
6	8.4309923	505.85954	8.408272	8.548771	285.040253	53492.7349	10083.63853	0.1404995	INF	No Filter	Trace15	3.26	4.19	Automated - ICIS	119658	Echinacea purpurea	Flowers		
7	8.6398772	518.39263	8.548771	8.865423	285.040649	165845.265	19826.46008	0.3166516	INF	No Filter	Trace15	10.12	8.24	Automated - ICIS	119658	Echinacea purpurea	Flowers	C15H10O6 Kaempferol	Level 4
8	8.9470293	536.82176	8.865423	9.004312	285.040497	26010.5938	5784.541068	0.1388894	INF	No Filter	Trace15	1.59	2.4	Automated - ICIS	119658	Echinacea purpurea	Flowers		
9	9.0798632	544.79179	9.004312	9.178131	0	46207.6998	6903.713581	0.1738184	INF	No Filter	Trace15	2.82	2.87	Automated - ICIS	119658	Echinacea purpurea	Flowers		
10	9.2298508	553.79105	9.178131	9.252571	0	17008.8988	7643.37768	0.0744408	INF	No Filter	Trace15	1.04	3.18	Automated - ICIS	119658	Echinacea purpurea	Flowers		
11	9.3918238	563.50943	9.252571	9.412066	285.040405	23951.4184	5880.562752	0.1594943	INF	No Filter	Trace15	1.46	2.44	Automated - ICIS	119658	Echinacea purpurea	Flowers		
12	9.5388609	572.33165	9.412066	9.693916	285.040802	66680.2361	9222.955102	0.2818499	INF	No Filter	Trace15	4.07	3.83	Automated - ICIS	119658	Echinacea purpurea	Flowers		
13	9.867568	592.05408	9.693916	10.0296	0	65229.6723	8525.412833	0.3356795	INF	No Filter	Trace15	3.98	3.54	Automated - ICIS	119658	Echinacea purpurea	Flowers		
14	10.048183	602.89098	10.0296	10.09698	285.03949	13922.2477	8342.766929	0.067381	INF	No Filter	Trace15	0.85	3.47	Automated - ICIS	119658	Echinacea purpurea	Flowers		
15	10.172169	610.33014	10.1257	10.31792	285.039612	27378.3495	5134.435225	0.192219	INF	No Filter	Trace15	1.67	2.13	Automated - ICIS	119658	Echinacea purpurea	Flowers		
16	10.348123	620.88738	10.31792	10.51246	285.040131	33176.5761	6676.237929	0.194539	INF	No Filter	Trace15	2.02	2.77	Automated - ICIS	119658	Echinacea purpurea	Flowers		
17	10.583007	634.98042	10.56442	10.64427	285.040741	11713.7528	4684.665264	0.079848	INF	No Filter	Trace15	0.71	1.95	Automated - ICIS	119658	Echinacea purpurea	Flowers		
18	10.822547	649.35282	10.76361	10.8931	285.039581	19137.0128	4317.754649	0.129488	INF	No Filter	Trace15	1.17	1.79	Automated - ICIS	119658	Echinacea purpurea	Flowers		
19	10.959002	657.54012	10.8931	10.98456	285.040588	8273.88284	4069.289055	0.091461	INF	No Filter	Trace15	0.5	1.69	Automated - ICIS	119658	Echinacea purpurea	Flowers		
20	11.691627	701.49762	11.63267	11.70789	0	6993.9397	3784.642158	0.075225	INF	No Filter	Trace15	0.43	1.57	Automated - ICIS	119658	Echinacea purpurea	Flowers		
1	8.3877648	503.26589	8.276416	9.196384	0	1444674.56	121525.52	0.9199684	INF	No Filter	Trace16	100	100	Automated - Genesis	119658	Echinacea purpurea	Flowers	C15H10O5 apigenin	Level 4
1	0.7640887	45.845321	0.75613	0.773147	137.023743	4064.81379	9683.253051	0.01701765	INF	No Filter	Trace17	20.93	24.82	Automated - Genesis	119658	Echinacea purpurea	Flowers		

2	0.8944613	53.667676	0.886488	0.903522	137.023773	2362.9257	5621.060682	0.01703378	INF	No Filter	Trace17	12.17	14.41	Automated - Genesis	119658	Echinacea purpurea	Flowers		
3	5.4473714	326.84228	5.43737	5.456872	137.023773	845.397464	1882.884502	0.0195017	INF	No Filter	Trace17	4.35	4.83	Automated - Genesis	119658	Echinacea purpurea	Flowers		
4	8.0639397	483.83638	8.05554	8.073441	137.023743	1795.65837	4102.596919	0.017901	INF	No Filter	Trace17	9.25	10.51	Automated - Genesis	119658	Echinacea purpurea	Flowers		
5	9.8616494	591.69896	9.849188	9.873267	137.023727	2682.12383	4462.311575	0.0240786	INF	No Filter	Trace17	13.81	11.44	Automated - Genesis	119658	Echinacea purpurea	Flowers		
6	9.9484702	596.90821	9.936001	9.960088	137.023788	2583.23774	4499.403797	0.0240869	INF	No Filter	Trace17	13.3	11.53	Automated - Genesis	119658	Echinacea purpurea	Flowers		
7	11.008867	660.53202	10.99725	11.02049	137.023849	1098.83058	1934.287168	0.023235	INF	No Filter	Trace17	5.66	4.96	Automated - Genesis	119658	Echinacea purpurea	Flowers		
8	11.433651	686.01906	11.42203	11.44611	137.024017	1309.55598	2239.31415	0.02408	INF	No Filter	Trace17	6.74	5.74	Automated - Genesis	119658	Echinacea purpurea	Flowers		
9	12.83054	769.8324	12.81892	12.84216	137.023727	1474.73304	2595.837976	0.023235	INF	No Filter	Trace17	7.59	6.65	Automated - Genesis	119658	Echinacea purpurea	Flowers		
10	12.853774	771.22644	12.84216	12.86624	137.023743	1202.27857	1999.456411	0.024085	INF	No Filter	Trace17	6.19	5.12	Automated - Genesis	119658	Echinacea purpurea	Flowers		
1	1.8216019	109.29611	1.814917	1.828286	153.018539	4261.37506	10544.86554	0.0133687	27.32766208	No Filter	Trace18	59.52	66.81	Automated - ICIS	119658	Echinacea purpurea	Flowers		
2	4.3482083	260.8925	4.341556	4.354564	0	2898.15639	5239.096737	0.0130075	13.57743868	No Filter	Trace18	40.48	33.19	Automated - ICIS	119658	Echinacea purpurea	Flowers		
1	3.9682482	238.09489	3.842258	4.89679	0	225338613	24345275.46	1.0545327	166.9839232	No Filter	Trace21	100	100	Automated - Genesis	119658	Echinacea purpurea	Flowers	C21H20O10 apigenin-o-glucoside	Level 3
1	1.5784146	94.704876	1.544827	1.649986	593.151611	75633.6894	25876.28747	0.1051599	INF	No Filter	Trace22	0.07	0.12	Automated - ICIS	119658	Echinacea purpurea	Flowers		
2	2.0207598	121.24559	1.957326	2.037773	593.152527	31072.4604	13051.64132	0.0804474	INF	No Filter	Trace22	0.03	0.06	Automated - ICIS	119658	Echinacea purpurea	Flowers		
3	2.1215422	127.29253	2.037773	2.230946	593.15271	162958.681	20935.0232	0.1931724	INF	No Filter	Trace22	0.15	0.1	Automated - ICIS	119658	Echinacea purpurea	Flowers		
4	2.3301115	139.80669	2.26923	2.557087	593.151917	42477408.7	6056222.565	0.2878568	INF	No Filter	Trace22	38.68	29.19	Automated - ICIS	119658	Echinacea purpurea	Flowers	C27H30O15 luteolin diglycosides	Level 3
5	2.6183464	157.10078	2.571028	2.632288	593.151978	103271.321	50395.85868	0.0612598	INF	No Filter	Trace22	0.09	0.24	Automated - ICIS	119658	Echinacea purpurea	Flowers		
6	2.8671877	172.03126	2.653199	3.01209	593.151917	5880518.61	796666.1543	0.3588913	INF	No Filter	Trace22	5.35	3.84	Automated - ICIS	119658	Echinacea purpurea	Flowers		
7	3.1555052	189.33031	3.057111	3.393564	593.151794	3549033.22	498656.9323	0.3364527	INF	No Filter	Trace22	3.23	2.4	Automated - ICIS	119658	Echinacea purpurea	Flowers		
8	3.4488788	206.93273	3.393564	3.45962	593.151611	35626.3525	19610.74441	0.0660557	INF	No Filter	Trace22	0.03	0.09	Automated - ICIS	119658	Echinacea purpurea	Flowers		
9	3.554308	213.25848	3.487022	3.69948	593.151733	55346051.1	12634668	0.2124582	INF	No Filter	Trace22	50.4	60.9	Automated - ICIS	119658	Echinacea purpurea	Flowers	C27H30O15 luteolin diglycosides	Level 3
10	3.7977478	227.86487	3.758003	3.807684	0	120721.347	89200.62145	0.0496811	INF	No Filter	Trace22	0.11	0.43	Automated - ICIS	119658	Echinacea purpurea	Flowers		
11	3.8486171	230.91703	3.826908	3.883258	593.152161	55308.2554	29610.65559	0.0563506	INF	No Filter	Trace22	0.05	0.14	Automated - ICIS	119658	Echinacea purpurea	Flowers		
12	3.9064967	234.3898	3.883258	3.956482	593.151978	56556.3319	34561.11189	0.0732233	INF	No Filter	Trace22	0.05	0.17	Automated - ICIS	119658	Echinacea purpurea	Flowers		
13	3.9848352	239.09011	3.973364	4.031262	593.151489	37287.679	24865.44729	0.0578973	INF	No Filter	Trace22	0.03	0.12	Automated - ICIS	119658	Echinacea purpurea	Flowers		
14	4.0478503	242.87102	4.032501	4.118348	593.152283	58364.4334	19853.35966	0.0858465	INF	No Filter	Trace22	0.05	0.1	Automated - ICIS	119658	Echinacea purpurea	Flowers		
15	4.1774013	250.64408	4.118348	4.336441	593.152039	1057632.5	250588.6509	0.2180928	INF	No Filter	Trace22	0.96	1.21	Automated - ICIS	119658	Echinacea purpurea	Flowers	C27H30O15 Kaempferol diglycosides	Level 3
16	4.3766285	262.59771	4.337976	4.38686	593.15155	8126.9496	6011.622503	0.0488838	INF	No Filter	Trace22	0.01	0.03	Automated - ICIS	119658	Echinacea purpurea	Flowers	C27H30O15 Kaempferol diglycosides	Level 3
17	4.4081322	264.48793	4.399436	4.601082	0	130125.746	16774.4485	0.2016463	INF	No Filter	Trace22	0.12	0.08	Automated - ICIS	119658	Echinacea purpurea	Flowers		
18	4.6617368	279.70421	4.601082	4.740092	593.152344	86770.1324	16789.1119	0.1390095	INF	No Filter	Trace22	0.08	0.08	Automated - ICIS	119658	Echinacea purpurea	Flowers	C27H30O15 Kaempferol diglycosides	Level 3
19	4.768012	286.08072	4.740092	4.782626	0	20263.6197	11671.40757	0.0425344	INF	No Filter	Trace22	0.02	0.06	Automated - ICIS	119658	Echinacea purpurea	Flowers		
20	4.8337885	290.02731	4.782626	4.84915	593.151123	38284.5097	14102.99984	0.0665239	INF	No Filter	Trace22	0.03	0.07	Automated - ICIS	119658	Echinacea purpurea	Flowers		
21	4.9377192	296.26315	4.84915	5.116501	593.150574	103069.392	11189.70522	0.2673512	INF	No Filter	Trace22	0.09	0.05	Automated - ICIS	119658	Echinacea purpurea	Flowers	C27H30O15 Kaempferol diglycosides	Level 3
22	5.2784133	316.7048	5.116501	5.29376	593.153137	62140.9409	9539.90384	0.1772593	INF	No Filter	Trace22	0.06	0.05	Automated - ICIS	119658	Echinacea purpurea	Flowers		
23	5.3398047	320.38828	5.29376	5.350037	593.152344	13548.6764	6312.628008	0.0562761	INF	No Filter	Trace22	0.01	0.03	Automated - ICIS	119658	Echinacea purpurea	Flowers		
24	5.3823434	322.9406	5.350037	5.392576	593.152222	7246.54218	6088.818123	0.0425396	INF	No Filter	Trace22	0.01	0.03	Automated - ICIS	119658	Echinacea purpurea	Flowers		
25	5.4158892	324.95335	5.392576	5.573046	0	76932.9671	12663.56819	0.1804703	INF	No Filter	Trace22	0.07	0.06	Automated - ICIS	119658	Echinacea purpurea	Flowers		
26	5.605268	336.31608	5.573046	5.636751	0	19728.8637	9177.157334	0.0637048	INF	No Filter	Trace22	0.02	0.04	Automated - ICIS	119658	Echinacea purpurea	Flowers		
27	5.719458	343.16748	5.658232	5.825402	0	148833.452	33023.04109	0.1671694	INF	No Filter	Trace22	0.14	0.16	Automated - ICIS	119658	Echinacea purpurea	Flowers		
28	5.8573384	351.4403	5.828982	5.929347	593.152039	20050.3726	6588.696974	0.1003641	INF	No Filter	Trace22	0.02	0.03	Automated - ICIS	119658	Echinacea purpurea	Flowers		
29	5.9680075	358.08045	5.932927	5.977001	593.151611	7526.69146	5952.438665	0.0440739	INF	No Filter	Trace22	0.01	0.03	Automated - ICIS	119658	Echinacea purpurea	Flowers		
30	6.0269013	361.61408	5.998704	6.068624	593.15155	13182.5559	7676.571786	0.0699201	INF	No Filter	Trace22	0.01	0.04	Automated - ICIS	119658	Echinacea purpurea	Flowers		
31	6.1788829	370.73297	6.159742	6.200744	593.153748	4462.55451	3527.907601	0.0410016	INF	No Filter	Trace22	0	0.02	Automated - ICIS	119658	Echinacea purpurea	Flowers		
32	6.2114847	372.68908	6.200744	6.247287	0	6429.04081	5393.363056	0.0465429	INF	No Filter	Trace22	0.01	0.03	Automated - ICIS	119658	Echinacea purpurea	Flowers		
1	2.5268818	151.61291	2.391241	2.646715	563.141113	22847172.2	4662368.651	0.255474453	INF	No Filter	Trace23	5.4	7.74	Manual	119658	Echinacea purpurea	Flowers		
2	2.8477465	170.86479	2.80003	3.003885	563.140747	51124188.8	6965581.371	0.2038557	INF	No Filter	Trace23	12.08	11.57	Manual	119658	Echinacea purpurea	Flowers		
3	3.0942867	185.6572	3.00438	3.208759	563.140564	292967056	37267873.52	0.204379562	INF	No Filter	Trace23	69.22	61.9	Manual	119658	Echinacea purpurea	Flowers	C26H28O14 apigenin di-glycoside family	Level 3
4	3.3333321	199.99993	3.290511	3.423358	0	38592275.3	7417507.445	0.132846715	INF	No Filter	Trace23	9.12	12.32	Manual	119658	Echinacea purpurea	Flowers		
5	3.4834414	209.00648	3.454015	3.576642	0	17721123.8	3896869.927	0.122627737	INF	No Filter	Trace23	4.19	6.47	Manual	119658	Echinacea purpurea	Flowers		
1	3.1090357	186.54214	3.004595	3.208989	447.093536	92420340.3	15309938.7	0.204394204	2588.762903	No Filter	Trace24	68.91	79.87	Manual	119658	Echinacea purpurea	Flowers	C21H20O11 Luteolin-O-glucoside	Level 3
2	3.2867897	197.20738	3.219209	3.699535	0	41698979.2	3859298.736	0.48032638	634.4255101	No Filter	Trace24	31.09	20.13	Manual	119658	Echinacea purpurea	Flowers	C21H20O11 Luteolin-O-glucoside; C21H20O11 kaempferol-O-glucoside	Level 3
1	0.8726106	52.356634	0.769238	1.177791	0	233247.175	27945.04651	0.40855365	INF	No Filter	Trace25	100	100	Automated - Genesis	119658	Echinacea purpurea	Flowers	i9G 2-hydroxyacryloyl-N-glutamate C8H11NO6	Level 4
1	0.6326402	37.958413	0.602291	0.712588	193.04982	104132.941	42154.01283	0.1102965	INF	No Filter	Trace26	62.35	53.78	Automated - Genesis	119658	Echinacea purpurea	Flowers		
2	0.7228898	43.373388	0.712588	0.733188	193.049911	5066.85097	9673.91281	0.02060019	INF	No Filter	Trace26	3.03	12.34	Automated - Genesis	119658	Echinacea purpurea	Flowers		
3	2.8811287	172.86772	2.8036	2.939216	193.049652	24374.3368	6756.723771	0.1356153	INF	No Filter	Trace26	14.6	8.62	Automated - Genesis	119658	Echinacea purpurea	Flowers		
4	3.045494	182.72964	2.979561	3.061758	0	7204.44101	5179.874941	0.0821974	INF	No Filter	Trace26	4.31	6.61	Automated - Genesis	119658	Echinacea purpurea	Flowers		
5	3.7905873	227.43524	3.772323	3.865569	0	11738.3799	5313.220298	0.0932459	INF	No Filter	Trace26	7.03	6.78	Automated - Genesis	119658	Echinacea purpurea	Flowers		
6	10.68948	641.3688	10.6523	10.72054	193.050262	6513.73328	4340.466099	0.068232	INF	No Filter	Trace26	3.9	5.54	Automated - Genesis	119658	Echinacea purpurea	Flowers		
7	10.748418	644.90508	10.72983	10.812	193.050034	7970.63947	4966.755157	0.082173	INF	No Filter	Trace26	4.77	6.34	Automated - Genesis	119658	Echinacea purpurea	Flowers		
1	1.5476192	92.857152	1.515178	1.604392	385.115173	23190.6787	10581.14351	0.0892138	INF	No Filter	Trace27	4.3	9.73	Automated - ICIS	119658	Echinacea purpurea	Flowers	C17H22O10 Sinapoyl-glucose	Level 3
2	2.5120943	150.72566	2.456331	2.751636	385.113892	449406.201	85341.7215	0.2953042	INF	No Filter	Trace27	83.31	78.49	Automated - ICIS	119658	Echinacea purpurea	Flowers	C17H22O10 Sinapoyl-glucose	Level 3
3	2.911334	174.68004	2.758606	2.981885	385.114075	61963.4119	10156.85284	0.2232782	INF	No Filter	Trace27	11.49	9.34	Automated - ICIS	119658	Echinacea purpurea	Flowers	C17H22O10 Sinapoyl-glucose	Level 3
4	2.9958253	179.74952	2.981885	3.040847	385.112671	4849.75157	2646.621991	0.0589626	INF	No Filter	Trace27	0.9	2.43	Automated - ICIS	119658	Echinacea purpurea	Flowers		

1	1.6829604	100.97762	1.673475	1.695159	339.072205	1020.32186	2013.333379	0.0216839	INF	No Filter	Trace28	12.9	14.91	Automated - Genesis	119658	Echinacea purpurea	Flowers		
2	2.5315286	151.89172	2.516742	2.545469	339.071075	1250.93181	1733.77557	0.0287276	INF	No Filter	Trace28	15.81	12.84	Automated - Genesis	119658	Echinacea purpurea	Flowers		
3	4.3919795	263.51877	4.381744	4.402211	339.073547	805.955966	1548.42554	0.0204666	INF	No Filter	Trace28	10.19	11.47	Automated - Genesis	119658	Echinacea purpurea	Flowers		
4	4.5307177	271.84306	4.518379	4.541458	339.071442	1033.5492	1814.19116	0.0230796	INF	No Filter	Trace28	13.06	13.44	Automated - Genesis	119658	Echinacea purpurea	Flowers		
5	4.555779	273.34674	4.545038	4.566519	339.07132	762.57801	1395.761166	0.0214812	INF	No Filter	Trace28	9.64	10.34	Automated - Genesis	119658	Echinacea purpurea	Flowers		
6	4.5772603	274.63562	4.566519	4.588001	339.071869	1016.2774	1860.100362	0.0214813	INF	No Filter	Trace28	12.85	13.78	Automated - Genesis	119658	Echinacea purpurea	Flowers		
7	4.6770844	280.62506	4.666853	4.687317	339.073547	738.957404	1419.988782	0.0204642	INF	No Filter	Trace28	9.34	10.52	Automated - Genesis	119658	Echinacea purpurea	Flowers		
8	9.7771603	586.62962	9.762372	9.791101	339.071869	1282.2993	1715.597035	0.0287291	INF	No Filter	Trace28	16.21	12.71	Automated - Genesis	119658	Echinacea purpurea	Flowers		
1	2.6578453	159.47072	2.596586	2.723754	335.077667	26044.3497	8741.306277	0.1271682	INF	No Filter	Trace29	88.05	56.33	Automated - Genesis	119658	Echinacea purpurea	Flowers	C16H16O8 caffeoyl-shikimate	Level 4
2	2.9818845	178.91307	2.970267	2.993502	335.076416	1190.98447	2096.434734	0.0232346	INF	No Filter	Trace29	4.03	13.51	Automated - Genesis	119658	Echinacea purpurea	Flowers		
3	5.1216167	307.297	5.113726	5.130627	335.077332	872.78035	2089.43528	0.0169006	INF	No Filter	Trace29	2.95	13.46	Automated - Genesis	119658	Echinacea purpurea	Flowers		
4	10.870937	652.25622	10.85932	10.88256	335.076996	1472.31736	2591.699364	0.023235	INF	No Filter	Trace29	4.98	16.7	Automated - Genesis	119658	Echinacea purpurea	Flowers		
1	0.6647838	39.887028	0.654043	0.675525	137.023727	6314.5996	11557.69134	0.02148124	INF	No Filter	Trace1	39.75	44.1	Automated - Genesis	119659	Echinacea tennesseensis	Roots		
2	2.4019716	144.1183	2.391708	2.415912	136.016129	840.454305	1417.200665	0.0242048	INF	No Filter	Trace1	5.29	5.41	Automated - Genesis	119659	Echinacea tennesseensis	Roots		
3	3.1414	188.484	3.127459	3.155341	136.016663	1299.49541	1832.509073	0.0278818	INF	No Filter	Trace1	8.18	6.99	Automated - Genesis	119659	Echinacea tennesseensis	Roots		
4	3.7807955	226.84773	3.768457	3.791536	137.023788	1878.84486	3297.959169	0.023079	INF	No Filter	Trace1	11.83	12.58	Automated - Genesis	119659	Echinacea tennesseensis	Roots		
5	8.8121683	528.7301	8.801428	8.822909	137.023727	1141.33779	2089.033186	0.0214813	INF	No Filter	Trace1	7.18	7.97	Automated - Genesis	119659	Echinacea tennesseensis	Roots		
6	11.18464	671.0784	11.1707	11.19943	137.023804	1582.84786	2157.85552	0.028729	INF	No Filter	Trace1	9.96	8.23	Automated - Genesis	119659	Echinacea tennesseensis	Roots		
7	11.774619	706.47714	11.75983	11.78856	137.023773	1475.91991	1948.897583	0.028733	INF	No Filter	Trace1	9.29	7.44	Automated - Genesis	119659	Echinacea tennesseensis	Roots		
8	13.588584	815.31504	13.57464	13.60253	137.023819	1353.18126	1908.168756	0.027882	INF	No Filter	Trace1	8.52	7.28	Automated - Genesis	119659	Echinacea tennesseensis	Roots		
1	4.2864962	257.18977	4.276264	4.296728	65.0388336	250.002022	479.9264885	0.0204642	INF	No Filter	Trace2	47.42	47.38	Automated - Genesis	119659	Echinacea tennesseensis	Roots		
2	5.3895099	323.37059	5.379278	5.399742	77.0396576	277.234498	532.9730063	0.0204637	INF	No Filter	Trace2	52.58	52.62	Automated - Genesis	119659	Echinacea tennesseensis	Roots		
1	1.8348861	110.09317	1.706774	1.962279	137.023315	217914.442	23725.89922	0.255505091	11.08532327	No Filter	Trace3	11.38	14.56	Manual	119659	Echinacea tennesseensis	Roots		
2	5.6579542	339.47725	5.528467	5.988321	137.023285	1696209.1	139266.3603	0.459854015	64.5858627	No Filter	Trace3	88.62	85.44	Manual	119659	Echinacea tennesseensis	Roots		
1	0.9150034	54.900201	0.901896	0.927643	0	1354.03812	1967.254262	0.0257475	INF	No Filter	Trace4	4.32	7.53	Automated - Genesis	119659	Echinacea tennesseensis	Roots		
2	0.9498626	56.991753	0.937946	0.960164	93.0334091	455.200222	831.8491329	0.02221855	INF	No Filter	Trace4	1.45	3.18	Automated - Genesis	119659	Echinacea tennesseensis	Roots		
3	3.770798	226.24788	3.760057	3.783136	93.0326385	325.421194	526.1062848	0.0230788	INF	No Filter	Trace4	1.04	2.01	Automated - Genesis	119659	Echinacea tennesseensis	Roots		
4	3.8097329	228.58397	3.798696	3.819966	93.0327377	361.766169	693.9027684	0.0212693	INF	No Filter	Trace4	1.15	2.66	Automated - Genesis	119659	Echinacea tennesseensis	Roots		
5	3.8506628	231.03977	3.840431	3.862495	93.0334625	396.798993	700.7842244	0.0220645	INF	No Filter	Trace4	1.26	2.68	Automated - Genesis	119659	Echinacea tennesseensis	Roots		
6	3.8778448	232.67069	3.867612	3.888076	93.0325775	253.673954	487.6599973	0.0204647	INF	No Filter	Trace4	0.81	1.87	Automated - Genesis	119659	Echinacea tennesseensis	Roots		
7	3.9715565	238.29339	3.961325	3.981789	93.0333023	267.025902	513.3122934	0.0204638	INF	No Filter	Trace4	0.85	1.97	Automated - Genesis	119659	Echinacea tennesseensis	Roots		
8	4.085734	245.14404	4.075502	4.095966	93.0330124	266.319078	511.9707409	0.0204641	INF	No Filter	Trace4	0.85	1.96	Automated - Genesis	119659	Echinacea tennesseensis	Roots		
9	4.1282605	247.69563	4.118028	4.164073	93.0331726	965.779313	685.5909215	0.0460447	INF	No Filter	Trace4	3.08	2.62	Automated - Genesis	119659	Echinacea tennesseensis	Roots		
10	4.2452053	254.71232	4.191256	4.257778	0	1605.00985	977.9637878	0.0665219	INF	No Filter	Trace4	5.12	3.74	Automated - Genesis	119659	Echinacea tennesseensis	Roots		
11	4.3003087	258.01852	4.290076	4.310541	93.0327682	231.607222	445.2709314	0.0204644	INF	No Filter	Trace4	0.74	1.7	Automated - Genesis	119659	Echinacea tennesseensis	Roots		
12	4.3514858	261.08915	4.34124	4.363317	93.0331955	345.728967	610.6260122	0.0220772	INF	No Filter	Trace4	1.1	2.34	Automated - Genesis	119659	Echinacea tennesseensis	Roots		
13	4.3735496	262.41298	4.363317	4.383782	93.0332794	379.277356	729.1010034	0.0204647	INF	No Filter	Trace4	1.21	2.79	Automated - Genesis	119659	Echinacea tennesseensis	Roots		
14	4.4233857	265.40314	4.396789	4.436467	0	956.818019	1110.734562	0.0396784	INF	No Filter	Trace4	3.05	4.25	Automated - Genesis	119659	Echinacea tennesseensis	Roots		
15	4.466719	268.00314	4.455978	4.477459	93.0333633	354.321831	648.5267421	0.0214809	INF	No Filter	Trace4	1.13	2.48	Automated - Genesis	119659	Echinacea tennesseensis	Roots		
16	4.5268461	271.61077	4.477459	4.564989	0	3370.66112	1611.542256	0.0875298	INF	No Filter	Trace4	10.74	6.17	Automated - Genesis	119659	Echinacea tennesseensis	Roots		
17	4.59487	275.6922	4.57573	4.622858	0	1724.49254	1045.572841	0.0471279	INF	No Filter	Trace4	5.5	4	Automated - Genesis	119659	Echinacea tennesseensis	Roots		
18	4.6791419	280.74851	4.66891	4.690978	93.0334244	436.079548	834.513232	0.0220678	INF	No Filter	Trace4	1.39	3.2	Automated - Genesis	119659	Echinacea tennesseensis	Roots		
19	4.7142184	282.8531	4.690978	4.737023	0	1747.62362	1395.468132	0.0460451	INF	No Filter	Trace4	5.57	5.34	Automated - Genesis	119659	Echinacea tennesseensis	Roots		
20	4.7641571	285.84943	4.737023	4.779656	0	1057.43034	1146.375695	0.042633	INF	No Filter	Trace4	3.37	4.39	Automated - Genesis	119659	Echinacea tennesseensis	Roots		
21	4.8221213	289.32728	4.800121	4.858007	0	1752.01643	1013.776498	0.0578865	INF	No Filter	Trace4	5.58	3.88	Automated - Genesis	119659	Echinacea tennesseensis	Roots		
22	4.8761305	292.56783	4.863123	4.888703	0	761.106114	1115.367901	0.0255798	INF	No Filter	Trace4	2.43	4.27	Automated - Genesis	119659	Echinacea tennesseensis	Roots		
23	4.9193997	295.16398	4.893819	4.929631	93.0333481	609.100821	634.8922165	0.035812	INF	No Filter	Trace4	1.94	2.43	Automated - Genesis	119659	Echinacea tennesseensis	Roots		
24	4.9516931	297.10159	4.94146	4.961926	93.0332718	459.079858	882.5831622	0.0204657	INF	No Filter	Trace4	1.46	3.38	Automated - Genesis	119659	Echinacea tennesseensis	Roots		
25	4.9992795	299.95677	4.987509	5.108409	0	3627.00541	1053.435796	0.1209001	INF	No Filter	Trace4	11.56	4.03	Automated - Genesis	119659	Echinacea tennesseensis	Roots		
26	5.1662373	309.97424	5.108409	5.243045	0	5379.34469	1245.276172	0.1346362	INF	No Filter	Trace4	17.14	4.77	Automated - Genesis	119659	Echinacea tennesseensis	Roots		
27	5.2686255	316.11753	5.258394	5.280465	93.033226	350.736335	619.1001297	0.022071	INF	No Filter	Trace4	1.12	2.37	Automated - Genesis	119659	Echinacea tennesseensis	Roots		
28	5.3111623	318.66974	5.300931	5.321394	93.0329132	231.931642	445.873315	0.0204636	INF	No Filter	Trace4	0.74	1.71	Automated - Genesis	119659	Echinacea tennesseensis	Roots		
29	5.3367427	320.20456	5.32651	5.346974	93.0329742	254.63522	489.5447557	0.0204637	INF	No Filter	Trace4	0.81	1.87	Automated - Genesis	119659	Echinacea tennesseensis	Roots		
30	5.3792784	322.7567	5.36393	5.394626	93.0326004	1097.15526	1145.110304	0.0306962	INF	No Filter	Trace4	3.5	4.38	Automated - Genesis	119659	Echinacea tennesseensis	Roots		
1	1.0956787	65.740722	1.02478	1.140337	0	27384.7761	7714.647715	0.1155572	INF	No Filter	Trace5	8.47	8.55	Automated - ICIS	119659	Echinacea tennesseensis	Roots		
2	1.179311	70.75866	1.140337	1.204618	0	13003.3992	5451.82584	0.0642812	INF	No Filter	Trace5	4.02	6.04	Automated - ICIS	119659	Echinacea tennesseensis	Roots		
3	1.2677626	76.065756	1.204618	1.380038	0	32036.0138	5512.032974	0.1754197	INF	No Filter	Trace5	9.91	6.11	Automated - ICIS	119659	Echinacea tennesseensis	Roots		
4	1.3954535	83.72721	1.385188	1.415984	225.039627	2324.49367	2594.356542	0.0307955	INF	No Filter	Trace5	0.72	2.87	Automated - ICIS	119659	Echinacea tennesseensis	Roots		
5	1.4416493	86.498958	1.431381	1.453537	225.040436	1137.01839	1851.898039	0.0221563	INF	No Filter	Trace5	0.35	2.05	Automated - ICIS	119659	Echinacea tennesseensis	Roots		
6	1.4689377	88.136262	1.45867	1.479202	225.040344	807.753923	1547.316667	0.0205323	INF	No Filter	Trace5	0.25	1.71	Automated - ICIS	119659	Echinacea tennesseensis	Roots		
7	1.4946039	89.676234	1.484336	1.504868	225.041138	1018.49769	1950.969234	0.0205326	INF	No Filter	Trace5	0.31	2.16	Automated - ICIS	119659	Echinacea tennesseensis	Roots		
8	1.5475451	92.852706	1.537281	1.557815	225.040085	1246.82214	2388.143527	0.020534	INF	No Filter	Trace5	0.39	2.65	Automated - ICIS	119659	Echinacea tennesseensis	Roots		
9	1.7339226	104.03536	1.724448	1.744501	225.039673	1317.09335	2612.368823	0.0200533	INF	No Filter	Trace5	0.41	2.89	Automated - ICIS	119659	Echinacea tennesseensis	Roots		
10	1.8749929	112.49957	1.865517	1.885571	225.0401	804.667441	1576.504361	0.0200532	INF	No Filter	Trace5	0.25	1.75	Automated - ICIS	119659	Echinacea tennesseensis	Roots		
11	1.8973892	113.84335	1.885571	1.912312	0	1720.22501	2116.398532	0.0267418	INF	No Filter	Trace5	0.53	2.34	Automated - ICIS	119659	Echinacea tennesseensis	Roots		
12	1.9418431	116.51059	1.932365	1.954052	225.040237	1096.50765	1818.650075	0.0216875	INF	No Filter	Trace5	0.34	2.01	Automated - ICIS	119659	Echinacea tennesseensis	Roots		
13	1.9635295	117.81177	1.954052	1.974108	225.040405	782.223362	1532.342844	0.0200562	INF	No Filter	Trace5	0.24	1.7	Automated - ICIS	119659	Echinacea tennesseensis	Roots		
14	2.0463202	122.77921	2.036052	2.056585	225.040192	1005.7604	1926.58861	0.0205327	INF	No Filter	Trace5	0.31	2.13	Automated - ICIS	119659	Echinacea tennesseensis	Roots	Isochorismate C10H10O6	Level 4
15	2.0668499	124.01099	2.056585	2.077115	225.039734	909.992368	1742.989816	0.0205307	INF	No Filter	Trace5	0.28	1.93	Automated - ICIS	119659	Echinacea tennesseensis	Roots	Isochorismate C10H10O6	Level 4
16	2.1751461	130.50877	2.160988	2.18665	0	1945.07055	2826.04058	0.0256616	INF	No Filter	Trace5	0.6	3.13	Automated - ICIS	119659	Echinacea tennesseensis	Roots		
17	2.2550034	135.3002	2.244735	2.265268	225.04155	740.771037	1419.030901	0.0205322	INF	No Filter	Trace5	0.23	1.57	Automated - ICIS	119659	Echinacea tennesseensis	Roots		
18	2.3490161	140.94097	2.338743	2.360896	225.040543	926.10101	1696.835073	0.022153	INF	No Filter	Trace5	0.29	1.88	Automated - ICIS	119659	Echinacea tennesseensis	Roots		
19	2.4321769	145.93061	2.418236	2.446959	225.040817	1437.48562	1992.518965	0.0287232	INF	No Filter	Trace5	0.44	2.21	Automated - ICIS	119659	Echinacea tennesseensis	Roots		
20	2.518987	151.13922	2.505046	2.533775	225.040771	1293.08675	1730.051168	0.0287288	INF	No Filter	Trace5	0.4	1.92	Automated - ICIS	119659	Echinacea tennesseensis	Roots		
21	2.6461463	158.76878	2.550039	2.845323	225.040268	200172.358	19541.96283	0.2952843	INF	No Filter	Trace5	61.9	21.65	Automated - ICIS	119659	Echinacea tennesseensis	Roots	Chorismate C10H10O6	Level 4
22	2.8949621	173.69773	2.847647	2.949257	0	15124.6659	5321.430533	0.1016101	INF	No Filter	Trace5	4.68	5.9	Automated - ICIS	119659	Echinacea tennesseensis	Roots		

23	2.9631975	177.79185	2.949257	3.009667	225.040466	8957.01131	5706.027153	0.0604102	INF	No Filter	Trace5	2.77	6.32	Automated - ICIS	119659	Echinacea tennesseensis	Roots		
24	3.0500121	183.00073	3.036071	3.063953	225.040604	2190.79805	3089.359274	0.0278818	INF	No Filter	Trace5	0.68	3.42	Automated - ICIS	119659	Echinacea tennesseensis	Roots		
25	3.1623114	189.73868	3.146047	3.183223	0	2944.74399	2652.686461	0.0371757	INF	No Filter	Trace5	0.91	2.94	Automated - ICIS	119659	Echinacea tennesseensis	Roots		
26	3.6200709	217.20425	3.60933	3.630811	225.039597	1063.04796	1945.71322	0.0214812	INF	No Filter	Trace5	0.33	2.16	Automated - ICIS	119659	Echinacea tennesseensis	Roots		
1	0.6707047	40.242284	0.613422	1.100843	174.075943	5503401.3	1450072.217	0.48742151	173.1386173	No Filter	Trace6	100	100	Automated - Genesis	119659	Echinacea tennesseensis	Roots	C15H17NO9 Isochorismate-9-glutamate	Level 2
1	2.5120165	150.72099	2.411679	2.881752	0	315111.836	33889.80804	0.470072993	INF	No Filter	Trace7	100	100	Manual	119659	Echinacea tennesseensis	Roots		
1	0.6576234	39.457402	0.646883	0.668364	163.039322	9228.7391	16891.60042	0.02148126	7.351406558	No Filter	Trace8	100	100	Automated - Genesis	119659	Echinacea tennesseensis	Roots		
1	0.6170017	37.020102	0.602289	0.664784	0	50255.9315	23535.08087	0.06249473	INF	No Filter	Trace9	9.94	22.69	Automated - Genesis	119659	Echinacea tennesseensis	Roots		
2	1.0675918	64.055508	1.004168	1.098502	223.060928	9945.6452	3588.649099	0.0943344	INF	No Filter	Trace9	1.97	3.46	Automated - Genesis	119659	Echinacea tennesseensis	Roots		
3	1.2266997	73.601982	1.164336	1.248473	0	10019.9696	3883.552312	0.0841367	INF	No Filter	Trace9	1.98	3.74	Automated - Genesis	119659	Echinacea tennesseensis	Roots		
4	1.4471651	86.829906	1.415984	1.45867	0	6200.66968	4951.460408	0.0426863	INF	No Filter	Trace9	1.23	4.77	Automated - Genesis	119659	Echinacea tennesseensis	Roots		
5	1.4728303	88.369818	1.45867	1.499736	0	4303.11503	3852.254266	0.0410661	INF	No Filter	Trace9	0.85	3.71	Automated - Genesis	119659	Echinacea tennesseensis	Roots		
6	1.5344894	92.069364	1.499736	1.78731	0	57814.7242	10073.69048	0.2875743	INF	No Filter	Trace9	11.43	9.71	Automated - Genesis	119659	Echinacea tennesseensis	Roots		
7	2.4632237	147.79342	2.446959	2.500399	0	4137.14	3490.006854	0.0534402	INF	No Filter	Trace9	0.82	3.36	Automated - Genesis	119659	Echinacea tennesseensis	Roots		
8	2.8763744	172.58246	2.646146	3.080217	223.060974	282090.808	32726.89497	0.4340709	INF	No Filter	Trace9	55.78	31.55	Automated - Genesis	119659	Echinacea tennesseensis	Roots	C11H12O5 Z-Sinapic acid	Level 4
9	3.1437234	188.6234	3.113518	3.225389	0	32649.506	9315.748458	0.1118709	INF	No Filter	Trace9	6.46	8.98	Automated - Genesis	119659	Echinacea tennesseensis	Roots		
10	4.2628934	255.7736	4.21451	4.391673	0	48297.559	8298.495041	0.1771633	INF	No Filter	Trace9	9.55	8	Automated - Genesis	119659	Echinacea tennesseensis	Roots		
1	0.8120086	48.720517	0.804051	0.821068	353.089325	35193.5578	67888.69372	0.017017	5.626436739	No Filter	Trace10	6.46	41.01	Automated - Genesis	119659	Echinacea tennesseensis	Roots		
2	1.1671481	70.028886	1.115905	1.492784	353.087677	95747.2222	18968.60178	0.3768785	INF	No Filter	Trace10	17.59	11.46	Manual	119659	Echinacea tennesseensis	Roots	C16H18O9 neochlorogenic acid (3-o-caffeoylquinic acid)	Level 4
3	1.5960821	95.764926	1.532847	1.665693	0	158355.336	41715.37203	0.132846715	INF	No Filter	Trace10	29.08	25.2	Manual	119659	Echinacea tennesseensis	Roots	C16H18O9 chlorogenic acid (5-o-caffeoylquinic acid)	Level 4
4	1.7256868	103.54121	1.675912	1.982482	353.088013	218751.697	25692.688	0.306569343	INF	No Filter	Trace10	40.18	15.52	Manual	119659	Echinacea tennesseensis	Roots	C16H18O9 cis 5-o-caffeoylquinic acid	Level 4
5	2.7399228	164.39537	2.268613	2.922628	353.087921	36428.3565	11285.27195	0.654014599	INF	No Filter	Trace10	6.69	6.82	Manual	119659	Echinacea tennesseensis	Roots	C16H18O9 4-o-caffeoylquinic acid (crytochlorogenic acid)	Level 4
1	4.6870336	281.22202	4.629355	4.792708	515.119934	89788.4491	17382.49563	0.163353	INF	No Filter	Trace11	13.29	12.17	Manual	119659	Echinacea tennesseensis	Roots	C25H24O12 3.4-dicaffeolquinic acid	Level 4
2	4.8658988	291.95393	4.813171	5.105792	515.118774	113608.784	16123.20282	0.2926208	INF	No Filter	Trace11	16.82	11.29	Manual	119659	Echinacea tennesseensis	Roots	C25H24O12 3,5-dicaffeolquinic acid	Level 4
3	5.1790233	310.7414	5.157318	5.236896	515.120972	25126.4868	8913.711096	0.0795779	INF	No Filter	Trace11	3.72	6.24	Automated - ICIS	119659	Echinacea tennesseensis	Roots		
4	5.2676116	316.0567	5.236896	5.330608	515.119812	16697.4539	5661.903645	0.0937122	INF	No Filter	Trace11	2.47	3.96	Automated - ICIS	119659	Echinacea tennesseensis	Roots		
5	5.3510724	321.06434	5.330608	5.35619	515.121643	6546.08061	7768.475577	0.0255812	INF	No Filter	Trace11	0.97	5.44	Automated - ICIS	119659	Echinacea tennesseensis	Roots		
6	5.4146272	324.87763	5.35619	5.648559	515.120239	347364.602	51931.35089	0.2923693	INF	No Filter	Trace11	51.42	36.35	Automated - ICIS	119659	Echinacea tennesseensis	Roots	C25H24O12 4,5-dicaffeolquinic acid	Level 4
7	5.7026203	342.15722	5.66646	5.725341	0	17439.4415	6624.974759	0.058881	INF	No Filter	Trace11	2.58	4.64	Automated - ICIS	119659	Echinacea tennesseensis	Roots		
8	5.7539826	345.23896	5.725341	5.770644	515.120239	10977.8009	6481.290754	0.045303	INF	No Filter	Trace11	1.62	4.54	Automated - ICIS	119659	Echinacea tennesseensis	Roots		
9	5.7937293	347.62376	5.770644	5.829246	0	13056.4113	7172.927705	0.0586025	INF	No Filter	Trace11	1.93	5.02	Automated - ICIS	119659	Echinacea tennesseensis	Roots		
10	5.8497102	350.98261	5.829246	5.885892	515.120056	10911.1319	4794.418327	0.0566456	INF	No Filter	Trace11	1.62	3.36	Automated - ICIS	119659	Echinacea tennesseensis	Roots		
11	5.9859475	359.15685	5.915055	5.998521	515.1203	13547.3381	4117.948166	0.0834659	INF	No Filter	Trace11	2.01	2.88	Automated - ICIS	119659	Echinacea tennesseensis	Roots		
12	6.0191964	361.15178	5.998521	6.069688	515.11969	10511.7717	5877.420261	0.0711674	INF	No Filter	Trace11	1.56	4.11	Automated - ICIS	119659	Echinacea tennesseensis	Roots		
1	3.4075994	204.45596	3.396859	3.41834	311.042297	947.828001	1734.822626	0.0214812	INF	No Filter	Trace12	7.66	10.58	Automated - Genesis	119659	Echinacea tennesseensis	Roots		
2	3.9692163	238.15298	3.943636	3.979449	311.040375	6179.90709	5981.156703	0.0358125	INF	No Filter	Trace12	49.94	36.49	Automated - Genesis	119659	Echinacea tennesseensis	Roots		
3	3.9896808	239.38085	3.979449	3.999912	311.039886	2108.72306	4052.304848	0.0204636	INF	No Filter	Trace12	17.04	24.72	Automated - Genesis	119659	Echinacea tennesseensis	Roots		
4	4.270785	256.2471	4.260553	4.28262	311.040222	998.175629	1633.423872	0.0220671	INF	No Filter	Trace12	8.07	9.97	Automated - Genesis	119659	Echinacea tennesseensis	Roots		
5	11.015652	660.93912	11.00171	11.03044	311.042114	1135.54565	1573.866947	0.028727	INF	No Filter	Trace12	9.18	9.6	Automated - Genesis	119659	Echinacea tennesseensis	Roots		
6	11.816441	708.98646	11.8025	11.83038	311.040314	1003.54036	1415.148116	0.027881	INF	No Filter	Trace12	8.11	8.63	Automated - Genesis	119659	Echinacea tennesseensis	Roots		
1	0.6826849	40.961096	0.671944	0.695061	473.074493	16734.1196	30096.32889	0.02311663	INF	No Filter	Trace13	80.73	90.35	Automated - Genesis	119659	Echinacea tennesseensis	Roots		
2	3.1228121	187.36873	3.108871	3.157664	473.07486	3993.56653	3215.980865	0.0487928	INF	No Filter	Trace13	19.27	9.65	Automated - Genesis	119659	Echinacea tennesseensis	Roots		
1	6.0058948	360.35369	5.993323	6.017875	0	1827.93738	3038.262355	0.0245521	INF	No Filter	Trace14	0.5	5.15	Automated - Genesis	119659	Echinacea tennesseensis	Roots		
2	6.6774829	400.64897	6.664402	6.689463	0	1987.8726	3227.575883	0.0250612	INF	No Filter	Trace14	0.55	5.47	Automated - Genesis	119659	Echinacea tennesseensis	Roots		
3	6.7269462	403.61677	6.705465	6.748427	301.035889	5641.27401	4878.548627	0.0429625	INF	No Filter	Trace14	1.55	8.27	Automated - Genesis	119659	Echinacea tennesseensis	Roots		
4	7.1262838	427.57703	6.989529	7.628405	301.035889	320960.775	25261.83909	0.6388767	INF	No Filter	Trace14	88.01	42.81	Automated - Genesis	119659	Echinacea tennesseensis	Roots	C15H10O7 quercetin	Level 4
5	7.7051889	462.31133	7.629645	7.767292	0	17566.3429	4036.221275	0.1376475	INF	No Filter	Trace14	4.82	6.84	Automated - Genesis	119659	Echinacea tennesseensis	Roots		
6	7.7927128	467.56277	7.776052	7.822594	0	4674.21164	3139.661446	0.0465428	INF	No Filter	Trace14	1.28	5.32	Automated - Genesis	119659	Echinacea tennesseensis	Roots		
7	7.8404953	470.42972	7.829755	7.851236	301.035431	1428.65172	2614.867628	0.021481	INF	No Filter	Trace14	0.39	4.43	Automated - Genesis	119659	Echinacea tennesseensis	Roots		
8	7.8766577	472.59946	7.863577	7.888638	0	2107.55734	3421.893664	0.0250615	INF	No Filter	Trace14	0.58	5.8	Automated - Genesis	119659	Echinacea tennesseensis	Roots		
9	7.90888	474.5328	7.895798	7.92086	0	1838.06713	2984.32439	0.0250615	INF	No Filter	Trace14	0.5	5.06	Automated - Genesis	119659	Echinacea tennesseensis	Roots		
10	7.97493	478.4958	7.961849	8.004811	0	3674.76952	3020.244225	0.0429625	INF	No Filter	Trace14	1.01	5.12	Automated - Genesis	119659	Echinacea tennesseensis	Roots		
11	8.2820691	496.92415	8.267389	8.30479	0	2971.54892	3380.550205	0.0374015	INF	No Filter	Trace14	0.81	5.73	Automated - Genesis	119659	Echinacea tennesseensis	Roots		
1	7.1081475	426.48885	7.017155	7.796801	0	1253305.27	138548.1142	0.7796454	INF	No Filter	Trace15	63.36	49.76	Manual	119659	Echinacea tennesseensis	Roots	C15H10O6 Luteolin	Level 4
2	7.922474	475.34844	7.86161	7.993346	0	33409.2741	6862.85209	0.1317357	INF	No Filter	Trace15	1.69	2.46	Automated - ICIS	119659	Echinacea tennesseensis	Roots		
3	8.0100073	480.60044	7.993346	8.150506	0	32513.721	7504.819856	0.1571597	INF	No Filter	Trace15	1.64	2.7	Automated - ICIS	119659	Echinacea tennesseensis	Roots		
4	8.2511136	495.06682	8.150506	8.263094	0	29541.9254	7313.547517	0.1125883	INF	No Filter	Trace15	1.49	2.63	Automated - ICIS	119659	Echinacea tennesseensis	Roots		
5	8.3665486	501.99292	8.263094	8.408272	285.040253	44378.4009	7341.053761	0.1451776	INF	No Filter	Trace15	2.24	2.64	Automated - ICIS	119659	Echinacea tennesseensis	Roots		
6	8.4309923	505.85954	8.408272	8.548771	285.040253	53492.7349	10083.63853	0.1404995	INF	No Filter	Trace15	2.7	3.62	Automated - ICIS	119659	Echinacea tennesseensis	Roots		
7	8.6398772	518.39263	8.548771	8.865423	285.040649	165845.265	19826.46008	0.3166516	INF	No Filter	Trace15	8.38	7.12	Automated - ICIS	119659	Echinacea tennesseensis	Roots	C15H10O6 Kaempferol	Level 4
8	8.9470293	536.82176	8.865423	9.004312	285.040497	26010.5938	5784.541068	0.1388894	INF	No Filter	Trace15	1.31	2.08	Automated - ICIS	119659	Echinacea tennesseensis	Roots		
9	9.0798632	544.79179	9.004312	9.178131	0	46207.6998	6903.713581	0.1738184	INF	No Filter	Trace15	2.34	2.48	Automated - ICIS	119659	Echinacea tennesseensis	Roots		
10	9.2298508	553.79105	9.178131	9.252571	0	17008.8988	7643.37768	0.0744408	INF	No Filter	Trace15	0.86	2.74	Automated - ICIS	119659	Echinacea tennesseensis	Roots		
11	9.3918238	563.50943	9.252571	9.412066	285.040405	23951.4184	5880.562752	0.1594943	INF	No Filter	Trace15	1.21	2.11	Automated - ICIS	119659	Echinacea tennesseensis	Roots		
12	9.5388609	572.33165	9.412066	9.693916	285.040802	66680.2361	9222.955102	0.2818499	INF	No Filter	Trace15	3.37	3.31	Automated - ICIS	119659	Echinacea tennesseensis	Roots		
13	9.867568	592.05408	9.693916	10.0296	0	65229.6723	8525.412833	0.3356795	INF	No Filter	Trace15	3.3	3.06	Automated - ICIS	119659	Echinacea tennesseensis	Roots		
14	10.048183	602.89098	10.0296	10.09698	285.03949	13922.2477	8342.766929	0.067381	INF	No Filter	Trace15	0.7	3	Automated - ICIS	119659	Echinacea tennesseensis	Roots		
15	10.172169	610.33014	10.1257	10.31792	285.039612	27378.3495	5134.435225	0.192219	INF	No Filter	Trace15	1.38	1.84	Automated - ICIS	119659	Echinacea tennesseensis	Roots		

16	10.348123	620.88738	10.31792	10.51246	285.040131	33176.5761	6676.237929	0.194539	INF	No Filter	Trace15	1.68	2.4	Automated - ICIS	119659	Echinacea tennesseensis	Roots		
17	10.583007	634.98042	10.56442	10.64427	285.040741	11713.7528	4684.665264	0.079848	INF	No Filter	Trace15	0.59	1.68	Automated - ICIS	119659	Echinacea tennesseensis	Roots		
18	10.822547	649.35282	10.76361	10.8931	285.039581	19137.0128	4317.754649	0.129488	INF	No Filter	Trace15	0.97	1.55	Automated - ICIS	119659	Echinacea tennesseensis	Roots		
19	10.959002	657.54012	10.8931	10.98456	285.040588	8273.88284	4069.289055	0.091461	INF	No Filter	Trace15	0.42	1.46	Automated - ICIS	119659	Echinacea tennesseensis	Roots		
20	11.691627	701.49762	11.63267	11.70789	0	6993.9397	3784.642158	0.075225	INF	No Filter	Trace15	0.35	1.36	Automated - ICIS	119659	Echinacea tennesseensis	Roots		
1	8.3839137	503.03482	8.270969	9.321785	0	1652524.82	144411.3015	1.050816	INF	No Filter	Trace16	100	100	Automated - Genesis	119659	Echinacea tennesseensis	Roots	C15H10O5 apigenin	Level 4
1	0.6647838	39.887028	0.656384	0.674285	137.023727	6314.59994	14427.08899	0.01790113	INF	No Filter	Trace17	46	50.34	Automated - Genesis	119659	Echinacea tennesseensis	Roots		
2	3.7807955	226.84773	3.770798	3.790296	137.023788	1848.30352	4116.734821	0.0194981	INF	No Filter	Trace17	13.46	14.36	Automated - Genesis	119659	Echinacea tennesseensis	Roots		
3	8.8121683	528.7301	8.803768	8.821669	137.023727	1141.33451	2607.671963	0.0179009	INF	No Filter	Trace17	8.31	9.1	Automated - Genesis	119659	Echinacea tennesseensis	Roots		
4	11.18464	671.0784	11.17302	11.1971	137.023804	1575.30346	2693.580637	0.024082	INF	No Filter	Trace17	11.47	9.4	Automated - Genesis	119659	Echinacea tennesseensis	Roots		
5	11.774619	706.47714	11.76215	11.78624	137.023773	1495.64072	2432.745263	0.024085	INF	No Filter	Trace17	10.89	8.49	Automated - Genesis	119659	Echinacea tennesseensis	Roots		
6	13.588584	815.31504	13.57697	13.6002	137.023819	1353.18126	2381.904796	0.023235	INF	No Filter	Trace17	9.86	8.31	Automated - Genesis	119659	Echinacea tennesseensis	Roots		
1	1.4946039	89.676234	1.488232	1.502077	153.01857	6783.62146	16404.93986	0.0138447	30.55058796	No Filter	Trace18	56.08	55.63	Automated - ICIS	119659	Echinacea tennesseensis	Roots		
2	3.5396897	212.38138	3.53487	3.545611	153.018646	5312.08296	13084.70618	0.0107407	24.36738388	No Filter	Trace18	43.92	44.37	Automated - ICIS	119659	Echinacea tennesseensis	Roots		
1	3.978209	238.69254	3.647473	5.805903	0	266842458	24988301.57	2.1584299	INF	No Filter	Trace21	100	100	Automated - Genesis	119659	Echinacea tennesseensis	Roots	C21H20O10 apigenin-o-glucoside	Level 3
1	1.5834767	95.008602	1.549886	1.658162	593.151672	91187.7763	29039.13904	0.1082758	INF	No Filter	Trace22	0.07	0.12	Automated - ICIS	119659	Echinacea tennesseensis	Roots		
2	2.097645	125.8587	1.982345	2.211149	593.151611	201233.208	25668.94756	0.2288042	INF	No Filter	Trace22	0.16	0.11	Automated - ICIS	119659	Echinacea tennesseensis	Roots		
3	2.3182127	139.09276	2.252212	2.584891	593.151489	50328260.1	6824144.186	0.3326794	INF	No Filter	Trace22	39.49	28.87	Automated - ICIS	119659	Echinacea tennesseensis	Roots	C27H30O15 luteolin diglycosides	Level 3
4	2.8647568	171.88541	2.678675	3.031424	593.152039	6914257.77	928553.5634	0.3527495	INF	No Filter	Trace22	5.42	3.93	Automated - ICIS	119659	Echinacea tennesseensis	Roots		
5	3.1623114	189.73868	3.033748	3.381298	593.151367	4149096.59	604554.1197	0.3475509	INF	No Filter	Trace22	3.26	2.56	Automated - ICIS	119659	Echinacea tennesseensis	Roots		
6	3.4290807	205.74484	3.40994	3.461663	593.151611	21570.7701	12017.25401	0.0517225	INF	No Filter	Trace22	0.02	0.05	Automated - ICIS	119659	Echinacea tennesseensis	Roots		
7	3.5361099	212.16659	3.475984	3.695614	593.151733	63298776.6	14511464.73	0.2196301	INF	No Filter	Trace22	49.66	61.4	Automated - ICIS	119659	Echinacea tennesseensis	Roots	C27H30O15 luteolin diglycosides	Level 3
8	3.7986964	227.92178	3.729075	3.807392	593.1521	264083.368	109587.403	0.0783175	INF	No Filter	Trace22	0.21	0.46	Automated - ICIS	119659	Echinacea tennesseensis	Roots		
9	3.8534379	231.20627	3.807392	3.875504	593.151489	67567.4344	28864.32513	0.0681116	INF	No Filter	Trace22	0.05	0.12	Automated - ICIS	119659	Echinacea tennesseensis	Roots		
10	3.9266892	235.60135	3.877845	3.940861	593.1521	47749.5936	29646.9782	0.0630157	INF	No Filter	Trace22	0.04	0.13	Automated - ICIS	119659	Echinacea tennesseensis	Roots		
11	3.9845643	239.07386	3.961325	4.049914	593.151978	42324.0669	20964.05774	0.0885883	INF	No Filter	Trace22	0.03	0.09	Automated - ICIS	119659	Echinacea tennesseensis	Roots		
12	4.0731612	244.38967	4.049914	4.114448	593.15155	29465.1972	18140.2764	0.0645346	INF	No Filter	Trace22	0.02	0.08	Automated - ICIS	119659	Echinacea tennesseensis	Roots		
13	4.1821982	250.93189	4.114448	4.325888	593.1521	1248161.34	302853.373	0.2114397	INF	No Filter	Trace22	0.98	1.28	Automated - ICIS	119659	Echinacea tennesseensis	Roots		
14	4.3337832	260.02699	4.325888	4.388897	593.151672	21807.5583	10174.47054	0.0630092	INF	No Filter	Trace22	0.02	0.04	Automated - ICIS	119659	Echinacea tennesseensis	Roots	C27H30O15 Kaempferol diglycosides	Level 3
15	4.4090648	264.54389	4.388897	4.614159	593.151611	150593.576	28191.27047	0.2252617	INF	No Filter	Trace22	0.12	0.12	Automated - ICIS	119659	Echinacea tennesseensis	Roots		
16	4.64333	278.5998	4.614159	4.726791	0	45813.4369	13095.54642	0.1126317	INF	No Filter	Trace22	0.04	0.06	Automated - ICIS	119659	Echinacea tennesseensis	Roots	C27H30O15 Kaempferol diglycosides	Level 3
17	4.7824312	286.94587	4.726791	4.84383	593.151184	34320.8035	8849.228553	0.117039	INF	No Filter	Trace22	0.03	0.04	Automated - ICIS	119659	Echinacea tennesseensis	Roots		
18	4.8902388	293.41433	4.84383	4.922175	0	30861.5913	11009.30821	0.0783455	INF	No Filter	Trace22	0.02	0.05	Automated - ICIS	119659	Echinacea tennesseensis	Roots	C27H30O15 Kaempferol diglycosides	Level 3
19	4.9324072	295.94443	4.922175	4.954469	593.153442	12907.9378	9587.888741	0.0322935	INF	No Filter	Trace22	0.01	0.04	Automated - ICIS	119659	Echinacea tennesseensis	Roots	C27H30O15 Kaempferol diglycosides	Level 3
20	5.0533034	303.1982	4.954469	5.162361	593.151978	72147.3664	9837.246609	0.2078929	INF	No Filter	Trace22	0.06	0.04	Automated - ICIS	119659	Echinacea tennesseensis	Roots		
21	5.2560535	315.36321	5.162361	5.333162	593.151611	61751.4709	8345.062053	0.170801	INF	No Filter	Trace22	0.05	0.04	Automated - ICIS	119659	Echinacea tennesseensis	Roots		
22	5.3446334	320.678	5.333162	5.397401	593.152771	17939.1116	8590.519147	0.064239	INF	No Filter	Trace22	0.01	0.04	Automated - ICIS	119659	Echinacea tennesseensis	Roots		
23	5.4542501	327.25501	5.397401	5.536961	593.149719	53287.1232	12219.31857	0.13956	INF	No Filter	Trace22	0.04	0.05	Automated - ICIS	119659	Echinacea tennesseensis	Roots		
24	5.7061001	342.36601	5.536961	5.893257	593.152649	193723.133	34974.55858	0.3562956	INF	No Filter	Trace22	0.15	0.15	Automated - ICIS	119659	Echinacea tennesseensis	Roots		
25	6.0214553	361.28732	5.944493	6.050463	593.153381	17070.5859	5879.758028	0.1059699	INF	No Filter	Trace22	0.01	0.02	Automated - ICIS	119659	Echinacea tennesseensis	Roots		
26	6.1463898	368.78339	6.082685	6.159471	593.154053	13748.4468	6960.34659	0.0767858	INF	No Filter	Trace22	0.01	0.03	Automated - ICIS	119659	Echinacea tennesseensis	Roots		
27	6.1932959	371.59775	6.159471	6.204037	0	6809.1632	4959.290535	0.0445658	INF	No Filter	Trace22	0.01	0.02	Automated - ICIS	119659	Echinacea tennesseensis	Roots		
28	6.2577396	375.46438	6.204037	6.26848	0	10505.2259	4920.456096	0.0644437	INF	No Filter	Trace22	0.01	0.02	Automated - ICIS	119659	Echinacea tennesseensis	Roots		
29	6.2915653	377.49392	6.26848	6.313047	0	5610.40123	5661.596112	0.0445663	INF	No Filter	Trace22	0	0.02	Automated - ICIS	119659	Echinacea tennesseensis	Roots		
30	6.4089891	384.53935	6.400589	6.445147	593.153381	3692.05232	3224.433018	0.0445585	INF	No Filter	Trace22	0	0.01	Automated - ICIS	119659	Echinacea tennesseensis	Roots		
31	6.6309402	397.85641	6.600697	6.641681	0	4314.80852	3930.693403	0.0409839	INF	No Filter	Trace22	0	0.02	Automated - ICIS	119659	Echinacea tennesseensis	Roots		
1	2.51434	150.8604	2.391241	2.646715	563.141174	28265877.5	5705083.089	0.255474453	INF	No Filter	Trace23	5.66	7.96	Manual	119659	Echinacea tennesseensis	Roots		
2	2.8476467	170.8588	2.80003	3.003885	563.140869	59119504	7850419.517	0.2038557	INF	No Filter	Trace23	11.83	10.95	Manual	119659	Echinacea tennesseensis	Roots		
3	3.063953	183.83718	3.00438	3.208759	563.141052	348162923	45025985.25	0.204379562	INF	No Filter	Trace23	69.66	62.79	Manual	119659	Echinacea tennesseensis	Roots	C26H28O14 apigenin di-glycoside family	Level 3
4	3.3224178	199.34507	3.290511	3.423358	0	44186493.9	8351001.018	0.132846715	INF	No Filter	Trace23	8.84	11.65	Manual	119659	Echinacea tennesseensis	Roots		
5	3.4688231	208.12939	3.454015	3.576642	0	20047684.1	4778902.015	0.122627737	INF	No Filter	Trace23	4.01	6.66	Manual	119659	Echinacea tennesseensis	Roots		
1	3.0988052	185.92831	3.004595	3.208989	447.093323	112871231	17857427.5	0.204394204	2826.88726	No Filter	Trace24	70.32	80.06	Manual	119659	Echinacea tennesseensis	Roots	C21H20O11 Luteolin-O-glucoside	Level 3
2	3.2758752	196.55251	3.219209	3.699535	0	47639830.5	4448338.61	0.48032638	692.4251978	No Filter	Trace24	29.68	19.94	Manual	119659	Echinacea tennesseensis	Roots	C21H20O11 Luteolin-O-glucoside; C21H20O11 kaempferol-O-glucoside	Level 3
1	0.8738093	52.42856	0.753732	1.150934	216.050735	274466.374	25767.65707	0.39720156	INF	No Filter	Trace25	96.1	83.55	Automated - Genesis	119659	Echinacea tennesseensis	Roots	i9G 2-hydroxyacryloyl-N-glutamate C8H11NO6	Level 4
2	1.1805505	70.83303	1.164336	1.233072	216.050674	11143.2419	5072.920556	0.068736	INF	No Filter	Trace25	3.9	16.45	Automated - Genesis	119659	Echinacea tennesseensis	Roots		
1	0.643303	38.598182	0.628982	0.675525	193.04982	42126.0376	28441.91072	0.04654229	INF	No Filter	Trace26	17.31	26.64	Automated - Genesis	119659	Echinacea tennesseensis	Roots		
2	0.952671	57.160258	0.940754	0.962973	193.049774	2490.03684	4731.001403	0.02221877	INF	No Filter	Trace26	1.02	4.43	Automated - Genesis	119659	Echinacea tennesseensis	Roots		
3	1.0144805	60.86883	1.004168	1.02478	193.049728	2436.0639	4645.090976	0.0206121	INF	No Filter	Trace26	1	4.35	Automated - Genesis	119659	Echinacea tennesseensis	Roots		
4	1.102396	66.14376	1.031546	1.117425	0	9316.02523	3604.868139	0.0858787	INF	No Filter	Trace26	3.83	3.38	Automated - Genesis	119659	Echinacea tennesseensis	Roots		
5	1.1375277	68.251662	1.124125	1.150934	0	3814.20958	4615.061611	0.0268087	INF	No Filter	Trace26	1.57	4.32	Automated - Genesis	119659	Echinacea tennesseensis	Roots		
6	1.2899126	77.394756	1.274136	1.301416	0	3221.58405	4749.635419	0.0272803	INF	No Filter	Trace26	1.32	4.45	Automated - Genesis	119659	Echinacea tennesseensis	Roots		
7	1.3448303	80.689818	1.301416	1.357886	0	5902.88887	3938.667879	0.0564698	INF	No Filter	Trace26	2.43	3.69	Automated - Genesis	119659	Echinacea tennesseensis	Roots		
8	2.8833442	173.00065	2.843	2.901932	193.049866	13359.0866	9288.097119	0.0589322	INF	No Filter	Trace26	5.49	8.7	Automated - Genesis	119659	Echinacea tennesseensis	Roots		

9	2.9655208	177.93125	2.904256	3.0686	193.049881	18695.3874	5134.430493	0.1643438	INF		No Filter	Trace26	7.68	4.81	Automated - Genesis	119659	Echinacea tennesseensis	Roots		
10	3.1553411	189.32047	3.139077	3.171605	0	3616.14977	4523.39867	0.0325285	INF		No Filter	Trace26	1.49	4.24	Automated - Genesis	119659	Echinacea tennesseensis	Roots		
11	3.2361298	194.16779	3.203908	3.24687	193.049789	3759.10272	2870.809275	0.0429625	INF		No Filter	Trace26	1.54	2.69	Automated - Genesis	119659	Echinacea tennesseensis	Roots		
12	3.4099402	204.59641	3.396859	3.459322	0	6886.55363	3182.867474	0.0624631	INF		No Filter	Trace26	2.83	2.98	Automated - Genesis	119659	Echinacea tennesseensis	Roots		
13	5.6101724	336.61034	5.518964	5.815352	0	105345.087	14017.2631	0.296388785	INF		No Filter	Trace26	43.28	13.13	Manual	119659	Echinacea tennesseensis	Roots	C10H10O4 Ferulic acid	Level 4
14	10.694775	641.6865	10.67766	10.73195	193.049744	5434.08502	3615.724998	0.054295	INF		No Filter	Trace26	2.23	3.39	Automated - Genesis	119659	Echinacea tennesseensis	Roots		
15	13.553732	813.22392	13.48551	13.58394	193.049927	11672.2854	3892.647285	0.098431	INF		No Filter	Trace26	4.8	3.65	Automated - Genesis	119659	Echinacea tennesseensis	Roots		
16	13.839777	830.38662	13.80725	13.85372	193.049774	5301.25172	5517.490584	0.04647	INF		No Filter	Trace26	2.18	5.17	Automated - Genesis	119659	Echinacea tennesseensis	Roots		
1	0.7537322	45.223934	0.743434	0.781097	385.115814	34623.3615	37083.07903	0.03766318	INF		No Filter	Trace27	5.64	22.45	Automated - ICIS	119659	Echinacea tennesseensis	Roots		
2	1.5372809	92.236854	1.520266	1.573212	385.114044	23542.2361	13267.00202	0.0529456	INF		No Filter	Trace27	3.84	8.03	Automated - ICIS	119659	Echinacea tennesseensis	Roots	C17H22O10 Sinapoyl-glucose	Level 3
3	2.5027224	150.16334	2.446959	2.687969	385.114014	490352.189	95675.21028	0.2410096	INF		No Filter	Trace27	79.88	57.93	Automated - ICIS	119659	Echinacea tennesseensis	Roots	C17H22O10 Sinapoyl-glucose	Level 3
4	2.8127947	168.76768	2.78956	2.826736	385.114655	7657.55175	8406.77438	0.0371756	INF		No Filter	Trace27	1.25	5.09	Automated - ICIS	119659	Echinacea tennesseensis	Roots	C17H22O10 Sinapoyl-glucose	Level 3
5	2.9367843	176.20706	2.826736	3.00502	385.113068	57689.2966	10715.82721	0.1782846	INF		No Filter	Trace27	9.4	6.49	Automated - ICIS	119659	Echinacea tennesseensis	Roots	C17H22O10 Sinapoyl-glucose	Level 3
1	0.7382844	44.297061	0.717668	0.748583	339.0737	31609.9549	35455.52627	0.0309146	INF		No Filter	Trace28	80.7	79.66	Automated - Genesis	119659	Echinacea tennesseensis	Roots		
2	4.4917799	269.50679	4.478699	4.50376	0	2595.74161	4214.49107	0.0250615	INF		No Filter	Trace28	6.63	9.47	Automated - Genesis	119659	Echinacea tennesseensis	Roots		
3	4.5399275	272.39565	4.510921	4.566228	0	4965.24401	4840.892264	0.0553076	INF		No Filter	Trace28	12.68	10.88	Automated - Genesis	119659	Echinacea tennesseensis	Roots		
1	2.6368519	158.21111	2.580244	2.719012	335.077148	33575.2472	13722.64367	0.1387678	INF		No Filter	Trace29	92.29	74.14	Automated - Genesis	119659	Echinacea tennesseensis	Roots	C16H16O8 caffeoyl-shikimate	Level 4
2	2.8267356	169.60414	2.815118	2.838353	335.075806	1261.27684	2220.129394	0.0232347	INF		No Filter	Trace29	3.47	12	Automated - Genesis	119659	Echinacea tennesseensis	Roots		
3	13.935884	836.15304	13.92427	13.94838	335.076233	1544.86413	2565.264488	0.024108	INF		No Filter	Trace29	4.25	13.86	Automated - Genesis	119659	Echinacea tennesseensis	Roots		
1	9.2283383	553.7003	9.217598	9.239079	510.082367	995.520124	1822.121256	0.0214812	INF		No Filter	Trace30	100	100	Automated - Genesis	119659	Echinacea tennesseensis	Roots		
1	2.7642267	165.8536	2.750286	2.779023	137.023911	865.144004	1198.859156	0.0287369	INF		No Filter	Trace1	3.56	3.6	Automated - Genesis	119660	Echinacea tennesseensis	Stems		
2	4.1065516	246.3931	4.096319	4.118387	137.023849	1496.22787	2448.287046	0.0220684	INF		No Filter	Trace1	6.16	7.35	Automated - Genesis	119660	Echinacea tennesseensis	Stems		
3	4.5567089	273.40253	4.545968	4.56745	137.023727	866.013827	1585.083539	0.0214813	INF		No Filter	Trace1	3.57	4.76	Automated - Genesis	119660	Echinacea tennesseensis	Stems		
4	6.3083794	378.50276	6.297639	6.31912	136.016098	803.863996	1471.314204	0.0214812	INF		No Filter	Trace1	3.31	4.42	Automated - Genesis	119660	Echinacea tennesseensis	Stems		
5	6.9942688	419.65613	6.983528	7.005306	137.023788	815.918097	1494.834664	0.0217774	INF		No Filter	Trace1	3.36	4.49	Automated - Genesis	119660	Echinacea tennesseensis	Stems		
6	7.1801365	430.80819	7.169904	7.190368	137.023743	1705.38855	3277.062972	0.0204638	INF		No Filter	Trace1	7.02	9.84	Automated - Genesis	119660	Echinacea tennesseensis	Stems		
7	8.3397342	500.38405	8.328994	8.350475	137.023743	1022.14216	1870.863882	0.0214813	INF		No Filter	Trace1	4.21	5.62	Automated - Genesis	119660	Echinacea tennesseensis	Stems		
8	9.6673319	580.03991	9.653391	9.681273	137.023819	1397.21533	1970.259714	0.0278817	INF		No Filter	Trace1	5.76	5.92	Automated - Genesis	119660	Echinacea tennesseensis	Stems		
9	9.7216141	583.29685	9.70535	9.749496	137.023773	4946.08598	5089.730232	0.0441459	INF		No Filter	Trace1	20.37	15.28	Automated - Genesis	119660	Echinacea tennesseensis	Stems		
10	10.031693	601.90158	10.01691	10.04563	137.023788	1099.35829	1523.772977	0.028725	INF		No Filter	Trace1	4.53	4.58	Automated - Genesis	119660	Echinacea tennesseensis	Stems		
11	10.299119	617.94714	10.28518	10.31306	137.023727	2242.10475	3161.671219	0.027882	INF		No Filter	Trace1	9.24	9.49	Automated - Genesis	119660	Echinacea tennesseensis	Stems		
12	11.34789	680.8734	11.33395	11.38359	137.023773	3636.22291	3498.025192	0.049639	INF		No Filter	Trace1	14.98	10.5	Automated - Genesis	119660	Echinacea tennesseensis	Stems		
13	12.432336	745.94016	12.4184	12.44713	137.023743	1393.60108	1931.340869	0.028732	INF		No Filter	Trace1	5.74	5.8	Automated - Genesis	119660	Echinacea tennesseensis	Stems		
14	12.681163	760.86978	12.66722	12.69595	137.023911	1048.35977	1453.067708	0.028726	INF		No Filter	Trace1	4.32	4.36	Automated - Genesis	119660	Echinacea tennesseensis	Stems		
15	13.371884	802.31304	13.35794	13.38583	137.023849	939.683942	1325.116274	0.027882	INF		No Filter	Trace1	3.87	3.98	Automated - Genesis	119660	Echinacea tennesseensis	Stems		
1	1.8092553	108.55532	1.707545	2.226821	137.023376	532828.176	60965.44656	0.5192756		29.19690517	No Filter	Trace3	99.95	98.77	Manual	119660	Echinacea tennesseensis	Stems		
2	5.5453177	332.71906	5.528467	5.988321	0	275.543485	757.6860214	0.459854015		0.361273973	No Filter	Trace3	0.05	1.23	Manual	119660	Echinacea tennesseensis	Stems		
1	3.8431153	230.58692	3.832884	3.853348	93.0333633	415.964183	799.5965829	0.0204646	INF		No Filter	Trace4	1.75	2.98	Automated - Genesis	119660	Echinacea tennesseensis	Stems		
2	3.8754096	232.52458	3.865178	3.895894	93.0330582	601.982109	653.6190934	0.0307157	INF		No Filter	Trace4	2.53	2.43	Automated - Genesis	119660	Echinacea tennesseensis	Stems		
3	3.921477	235.28862	3.895894	3.931716	93.0329514	542.393941	591.6061746	0.0358222	INF		No Filter	Trace4	2.28	2.2	Automated - Genesis	119660	Echinacea tennesseensis	Stems		
4	3.9565627	237.39376	3.943553	3.984484	0	892.067596	855.5171046	0.0409303	INF		No Filter	Trace4	3.76	3.19	Automated - Genesis	119660	Echinacea tennesseensis	Stems		
5	4.015182	240.91092	4.00495	4.027019	93.033226	312.103623	597.2782559	0.0220692	INF		No Filter	Trace4	1.31	2.22	Automated - Genesis	119660	Echinacea tennesseensis	Stems		
6	4.0986593	245.91956	4.088426	4.110495	93.0333328	397.198728	760.1090467	0.0220684	INF		No Filter	Trace4	1.67	2.83	Automated - Genesis	119660	Echinacea tennesseensis	Stems		
7	4.1258437	247.55062	4.11561	4.136077	93.0331573	306.376973	589.0004373	0.0204667	INF		No Filter	Trace4	1.29	2.19	Automated - Genesis	119660	Echinacea tennesseensis	Stems		
8	4.1770108	250.62065	4.166773	4.187247	93.032959	263.050166	505.6666152	0.0204738	INF		No Filter	Trace4	1.11	1.88	Automated - Genesis	119660	Echinacea tennesseensis	Stems		
9	4.2655917	255.9355	4.255358	4.277496	93.0332336	419.921509	803.3780504	0.0221373	INF		No Filter	Trace4	1.77	2.99	Automated - Genesis	119660	Echinacea tennesseensis	Stems		
10	4.3967776	263.80666	4.386546	4.406713	93.0328522	416.915047	800.745004	0.0201671	INF		No Filter	Trace4	1.76	2.98	Automated - Genesis	119660	Echinacea tennesseensis	Stems		
11	4.4233744	265.40246	4.412634	4.434115	93.0332108	408.437016	747.5659243	0.0214812	INF		No Filter	Trace4	1.72	2.78	Automated - Genesis	119660	Echinacea tennesseensis	Stems		
12	4.4799229	268.79537	4.437695	4.493004	0	1589.72329	1106.046641	0.0553089	INF		No Filter	Trace4	6.69	4.12	Automated - Genesis	119660	Echinacea tennesseensis	Stems		
13	4.5144853	270.86912	4.503745	4.541148	93.0331573	553.679129	518.8681311	0.0374038	INF		No Filter	Trace4	2.33	1.93	Automated - Genesis	119660	Echinacea tennesseensis	Stems		
14	4.5817704	274.90622	4.555469	4.613853	0	1297.94841	1056.890178	0.0583839	INF		No Filter	Trace4	5.47	3.94	Automated - Genesis	119660	Echinacea tennesseensis	Stems		
15	4.659899	279.59394	4.649666	4.67013	93.0333252	365.638243	702.9722623	0.0204646	INF		No Filter	Trace4	1.54	2.62	Automated - Genesis	119660	Echinacea tennesseensis	Stems		
16	4.6803619	280.82171	4.67013	4.722907	93.0331345	1493.7126	746.138269	0.0527765	INF		No Filter	Trace4	6.29	2.78	Automated - Genesis	119660	Echinacea tennesseensis	Stems		
17	4.7449064	284.69438	4.722907	4.775673	0	1108.56184	833.4782665	0.0527667	INF		No Filter	Trace4	4.67	3.1	Automated - Genesis	119660	Echinacea tennesseensis	Stems		
18	4.8027897	288.16738	4.785906	4.816605	0	964.020487	1036.586732	0.0306987	INF		No Filter	Trace4	4.06	3.86	Automated - Genesis	119660	Echinacea tennesseensis	Stems		
19	4.8370839	290.22503	4.821721	4.86427	93.0330353	1287.99216	1035.256109	0.0425492	INF		No Filter	Trace4	5.42	3.86	Automated - Genesis	119660	Echinacea tennesseensis	Stems		
20	4.8745022	292.47013	4.86427	4.884734	93.0332642	264.587052	508.6563114	0.020464	INF		No Filter	Trace4	1.11	1.89	Automated - Genesis	119660	Echinacea tennesseensis	Stems		
21	4.925663	295.53978	4.905199	4.947734	93.0332336	1133.98424	783.1249847	0.0425349	INF		No Filter	Trace4	4.77	2.92	Automated - Genesis	119660	Echinacea tennesseensis	Stems		
22	4.9784512	298.70707	4.95285	4.988683	93.0334244	587.372552	660.6360388	0.0358326	INF		No Filter	Trace4	2.47	2.46	Automated - Genesis	119660	Echinacea tennesseensis	Stems		
23	5.0040315	300.24189	4.9938	5.014264	93.0333862	418.803206	805.129431	0.0204634	INF		No Filter	Trace4	1.76	3	Automated - Genesis	119660	Echinacea tennesseensis	Stems		
24	5.0261043	301.56626	5.014264	5.036336	93.032959	367.505954	601.2588541	0.022072	INF		No Filter	Trace4	1.55	2.24	Automated - Genesis	119660	Echinacea tennesseensis	Stems		
25	5.0465682	302.79409	5.036336	5.0568	93.0331955	365.725456	703.1209984	0.0204646	INF		No Filter	Trace4	1.54	2.62	Automated - Genesis	119660	Echinacea tennesseensis	Stems		
26	5.0670319	304.02191	5.0568	5.077265	93.032814	275.987951	530.5290118	0.020465	INF		No Filter	Trace4	1.16	1.98	Automated - Genesis	119660	Echinacea tennesseensis	Stems		
27	5.094151	305.64906	5.082381	5.109576	0	772.062125	1139.878489	0.0271955	INF		No Filter	Trace4	3.25	4.25	Automated - Genesis	119660	Echinacea tennesseensis	Stems		
28	5.1469245	308.81547	5.119809	5.160737	0	1085.94158	1223.818285	0.0409284	INF		No Filter	Trace4	4.57	4.56	Automated - Genesis	119660	Echinacea tennesseensis	Stems		
29	5.194575	311.6745	5.17097	5.233972	0	1874.46776	944.7113009	0.0630016	INF		No Filter	Trace4	7.89	3.52	Automated - Genesis	119660	Echinacea tennesseensis	Stems		
30	5.2442031	314.65219	5.233972	5.254453	93.0330276	338.468142	649.4455558	0.0204812	INF		No Filter	Trace4	1.43	2.42	Automated - Genesis	119660	Echinacea tennesseensis	Stems		
31	5.2765211	316.59127	5.264684	5.286754	93.0332947	398.889667	652.8056284	0.0220702	INF		No Filter	Trace4	1.68	2.43	Automated - Genesis	119660	Echinacea tennesseensis	Stems		
32	5.3151105	318.90663	5.302103	5.327683	0	563.31527	840.3812697	0.0255804	INF		No Filter	Trace4	2.37	3.13	Automated - Genesis	119660	Echinacea tennesseensis	Stems		
33	5.3394521	320.36713	5.327683	5.353264	0	646.145274	950.7443019	0.0255807	INF		No Filter	Trace4	2.72	3.54	Automated - Genesis	119660	Echinacea tennesseensis	Stems		
34	5.3804495	322.82697	5.365098	5.395816	93.0332184	1018.98963	1115.245865	0.0307182	INF		No Filter	Trace4	4.29	4.15	Automated - Genesis	119660	Echinacea tennesseensis	Stems		
1	1.1630159	69.780954	1.153508	1.173612	225.040024	778.846316	1522.088165	0.0201048	INF		No Filter	Trace5	27.96	32.09	Automated - ICIS	119660	Echinacea tennesseensis	Stems		
2	2.4735677	148.41406	2.459627	2.487508	225.040298	1072.60886	1512.54765	0.0278817	INF		No Filter	Trace5	38.51	31.89	Automated - ICIS	119660	Echinacea tennesseensis	Stems		
3	7.8145063	468.87038	7.803765	7.825246	225.039581	933.762969	1709.116	0.0214812	INF		No Filter	Trace5	33.53	36.03	Automated - ICIS	119660	Echinacea tennesseensis	Stems		
1	0.613433	36.805981	0.602293	0.650475	0	20183.7946	15855.88545	0.04818108	INF		No Filter	Trace6	84.42	69.37	Automated - Genesis	119660	Echinacea tennesseensis	Stems		

2	0.6612153	39.672918	0.650475	0.671956	174.076477	1364.28358	2497.071716	0.02148131	INF	No Filter	Trace6	5.71	10.93	Automated - Genesis	119660	Echinacea tennesseensis	Stems	C15H17NO9 Isochorismate-9-glutamate	Level 2
3	0.820693	49.241582	0.81039	0.830992	174.076065	1190.13447	2271.34898	0.02060214	INF	No Filter	Trace6	4.98	9.94	Automated - Genesis	119660	Echinacea tennesseensis	Stems		
4	0.9150044	54.900261	0.904703	0.925303	174.075562	1169.26446	2232.102234	0.02059962	INF	No Filter	Trace6	4.89	9.77	Automated - Genesis	119660	Echinacea tennesseensis	Stems		
1	2.5177135	151.06281	2.411679	2.881752	179.034149	638960.761	69423.55377	0.470072993	INF	No Filter	Trace7	100	100	Manual	119660	Echinacea tennesseensis	Stems		
1	0.6170132	37.020789	0.602293	0.888011	0	183592.481	35627.19989	0.28571707	INF	No Filter	Trace9	84.69	78.62	Automated - Genesis	119660	Echinacea tennesseensis	Stems		
2	0.9070441	54.422644	0.894399	1.049545	0	29506.2522	5956.685992	0.15514579	INF	No Filter	Trace9	13.61	13.14	Automated - Genesis	119660	Echinacea tennesseensis	Stems		
3	2.696846	161.81076	2.679733	2.717757	223.060379	3681.23552	3734.208088	0.0380244	INF	No Filter	Trace9	1.7	8.24	Automated - Genesis	119660	Echinacea tennesseensis	Stems	C11H12O5 Z-Sinapic acid	Level 4
1	1.5947665	95.68599	1.532847	1.829197	353.087708	76353.4386	11819.80563	0.296350365	INF	No Filter	Trace10	100	100	Manual	119660	Echinacea tennesseensis	Stems	C16H18O9 chlorogenic acid (5-o-caffeoylquinic acid); C16H18O9 cis 5-o-caffeoylquinic acid	Level 4
1	1.5370608	92.223648	1.369343	1.573723	0	5525524.16	746403.1943	0.204379562	261.1745315	No Filter	Trace12	100	100	Manual	119660	Echinacea tennesseensis	Stems	C13H12O9 caftaric acid	Level 4
1	10.461139	627.66834	10.4472	10.47508	301.034302	1035.45634	1460.167397	0.027882	INF	No Filter	Trace14	50.1	50.14	Automated - Genesis	119660	Echinacea tennesseensis	Stems		
2	12.036287	722.17722	12.0215	12.05023	301.035034	1031.43369	1451.914391	0.028728	INF	No Filter	Trace14	49.9	49.86	Automated - Genesis	119660	Echinacea tennesseensis	Stems		
1	2.7642267	165.8536	2.752609	2.776699	137.023911	859.197929	1496.496766	0.0240898	INF	No Filter	Trace17	3.66	3.78	Automated - Genesis	119660	Echinacea tennesseensis	Stems		
2	4.1065516	246.3931	4.098659	4.117148	137.023849	1543.44807	3056.116834	0.0184884	INF	No Filter	Trace17	6.58	7.72	Automated - Genesis	119660	Echinacea tennesseensis	Stems		
3	4.5567089	273.40253	4.548309	4.56621	137.023727	866.012673	1978.608062	0.0179009	INF	No Filter	Trace17	3.69	5	Automated - Genesis	119660	Echinacea tennesseensis	Stems		
4	6.9942688	419.65613	6.985869	7.002965	137.023788	817.526231	1865.953337	0.0170956	INF	No Filter	Trace17	3.48	4.71	Automated - Genesis	119660	Echinacea tennesseensis	Stems		
5	7.1801365	430.80819	7.172245	7.189129	137.023743	1706.18598	4090.65078	0.016884	INF	No Filter	Trace17	7.27	10.33	Automated - Genesis	119660	Echinacea tennesseensis	Stems		
6	8.3397342	500.38405	8.331335	8.349236	137.023743	1022.13922	2335.33834	0.0179009	INF	No Filter	Trace17	4.35	5.9	Automated - Genesis	119660	Echinacea tennesseensis	Stems		
7	9.6673319	580.03991	9.655714	9.678949	137.023819	1397.21665	2459.410914	0.023235	INF	No Filter	Trace17	5.95	6.21	Automated - Genesis	119660	Echinacea tennesseensis	Stems		
8	9.7216141	583.29685	9.707673	9.747172	137.023773	4946.08892	6221.527243	0.0394991	INF	No Filter	Trace17	21.07	15.71	Automated - Genesis	119660	Echinacea tennesseensis	Stems		
9	10.031693	601.90158	10.01923	10.04331	137.023788	1091.91113	1902.076088	0.024078	INF	No Filter	Trace17	4.65	4.8	Automated - Genesis	119660	Echinacea tennesseensis	Stems		
10	10.299119	617.94714	10.2875	10.31074	137.023727	2242.10475	3946.61102	0.023235	INF	No Filter	Trace17	9.55	9.97	Automated - Genesis	119660	Echinacea tennesseensis	Stems		
11	11.34789	680.8734	11.33627	11.38127	137.023773	3617.09729	4366.470711	0.044992	INF	No Filter	Trace17	15.41	11.03	Automated - Genesis	119660	Echinacea tennesseensis	Stems		
12	12.432336	745.94016	12.42072	12.4448	137.023743	1384.10799	2410.82979	0.024084	INF	No Filter	Trace17	5.9	6.09	Automated - Genesis	119660	Echinacea tennesseensis	Stems		
13	12.681163	760.86978	12.66955	12.69363	137.023911	1041.24476	1813.817008	0.024079	INF	No Filter	Trace17	4.44	4.58	Automated - Genesis	119660	Echinacea tennesseensis	Stems		
14	13.371884	802.31304	13.36027	13.3835	137.023849	939.685092	1654.099408	0.023234	INF	No Filter	Trace17	4	4.18	Automated - Genesis	119660	Echinacea tennesseensis	Stems		
1	13.036248	782.17488	13.02146	13.05019	216.98082	1371.02966	1929.904095	0.028734	INF	No Filter	Trace20	100	100	Automated - Genesis	119660	Echinacea tennesseensis	Stems		
1	11.712257	702.73542	11.69832	11.7262	431.098328	1118.24491	1576.876705	0.027882	INF	No Filter	Trace21	100	100	Automated - Genesis	119660	Echinacea tennesseensis	Stems		
1	3.8203098	229.21859	3.810078	3.830543	447.093353	915.757777	1759.900328	0.0204657	INF	No Filter	Trace24	44.22	53.25	Automated - Genesis	119660	Echinacea tennesseensis	Stems		
2	12.103668	726.22008	12.08973	12.11846	447.09433	1154.95125	1545.258167	0.02873	INF	No Filter	Trace24	55.78	46.75	Automated - Genesis	119660	Echinacea tennesseensis	Stems		
1	0.8567373	51.404239	0.846439	0.868653	216.051315	2490.28054	4047.155574	0.02221408	INF	No Filter	Trace25	50.77	47.41	Automated - Genesis	119660	Echinacea tennesseensis	Stems	i9G 2-hydroxyacryloyl-N-glutamate C8H11NO6	Level 4
2	4.6370933	278.2256	4.626861	4.647325	216.050674	844.135743	1622.183667	0.0204637	INF	No Filter	Trace25	17.21	19	Automated - Genesis	119660	Echinacea tennesseensis	Stems		
3	5.7216127	343.29676	5.710872	5.732353	216.050446	778.835505	1425.509344	0.0214812	INF	No Filter	Trace25	15.88	16.7	Automated - Genesis	119660	Echinacea tennesseensis	Stems		
4	8.3664045	501.98427	8.354055	8.377145	216.051895	791.562804	1442.436749	0.02309	INF	No Filter	Trace25	16.14	16.9	Automated - Genesis	119660	Echinacea tennesseensis	Stems		
1	0.643303	38.598182	0.628982	0.675525	193.04982	42126.0376	28441.91072	0.04654229	INF	No Filter	Trace26	0.62	3.12	Automated - Genesis	119660	Echinacea tennesseensis	Stems		
2	0.952671	57.160258	0.940754	0.962973	193.049774	2490.03684	4731.001403	0.02221877	INF	No Filter	Trace26	0.04	0.52	Automated - Genesis	119660	Echinacea tennesseensis	Stems		
3	1.0144805	60.86883	1.004168	1.02478	193.049728	2436.0639	4645.090976	0.0206121	INF	No Filter	Trace26	0.04	0.51	Automated - Genesis	119660	Echinacea tennesseensis	Stems		
4	1.102396	66.14376	1.031546	1.117425	0	9316.02523	3604.868139	0.0858787	INF	No Filter	Trace26	0.14	0.4	Automated - Genesis	119660	Echinacea tennesseensis	Stems		
5	1.1375277	68.251662	1.124125	1.150934	0	3814.20958	4615.061611	0.0268087	INF	No Filter	Trace26	0.06	0.51	Automated - Genesis	119660	Echinacea tennesseensis	Stems		
6	1.2899126	77.394756	1.274136	1.301416	0	3221.58405	4749.635419	0.0272803	INF	No Filter	Trace26	0.05	0.52	Automated - Genesis	119660	Echinacea tennesseensis	Stems		
7	1.3448303	80.689818	1.301416	1.357886	0	5902.88887	3938.667879	0.0564698	INF	No Filter	Trace26	0.09	0.43	Automated - Genesis	119660	Echinacea tennesseensis	Stems		
8	2.8833442	173.00065	2.843	2.901932	193.049866	13359.0866	9288.097119	0.0589322	INF	No Filter	Trace26	0.2	1.02	Automated - Genesis	119660	Echinacea tennesseensis	Stems		
9	2.9655208	177.93125	2.904256	3.0686	193.049881	18695.3874	5134.430493	0.1643438	INF	No Filter	Trace26	0.27	0.56	Automated - Genesis	119660	Echinacea tennesseensis	Stems		
10	3.1553411	189.32047	3.139077	3.171605	0	3616.14977	4523.39867	0.0325285	INF	No Filter	Trace26	0.05	0.5	Automated - Genesis	119660	Echinacea tennesseensis	Stems		
11	3.2361298	194.16779	3.203908	3.24687	193.049789	3759.10272	2870.809275	0.0429625	INF	No Filter	Trace26	0.05	0.31	Automated - Genesis	119660	Echinacea tennesseensis	Stems		
12	3.4099402	204.59641	3.396859	3.459322	0	6886.55363	3182.867474	0.0624631	INF	No Filter	Trace26	0.1	0.35	Automated - Genesis	119660	Echinacea tennesseensis	Stems		
13	5.6026007	336.15604	5.518964	5.815352	0	6704393.83	819050.777	0.296388785	864.442684	No Filter	Trace26	97.98	89.83	Manual	119660	Echinacea tennesseensis	Stems	C10H10O4 Ferulic acid	Level 4
14	10.694775	641.6865	10.67766	10.73195	193.049744	5434.08502	3615.724998	0.054295	INF	No Filter	Trace26	0.08	0.4	Automated - Genesis	119660	Echinacea tennesseensis	Stems		
15	13.553732	813.22392	13.48551	13.58394	193.049927	11672.2854	3892.647285	0.098431	INF	No Filter	Trace26	0.17	0.43	Automated - Genesis	119660	Echinacea tennesseensis	Stems		
16	13.839777	830.38662	13.80725	13.85372	193.049774	5301.25172	5517.490584	0.04647	INF	No Filter	Trace26	0.08	0.48	Automated - Genesis	119660	Echinacea tennesseensis	Stems		
1	1.5588321	93.529926	1.521283	1.620445	385.114319	18708.693	8827.076166	0.0991626	INF	No Filter	Trace27	14.97	25.06	Automated - ICIS	119660	Echinacea tennesseensis	Stems	C17H22O10 Sinapoyl-glucose	Level 3
2	2.1110358	126.66215	2.095264	2.153338	0	6155.48996	3353.021388	0.0580738	INF	No Filter	Trace27	4.93	9.52	Automated - ICIS	119660	Echinacea tennesseensis	Stems	C17H22O10 Sinapoyl-glucose	Level 3
3	2.4030183	144.1811	2.356755	2.450333	385.113495	14446.3919	5009.667681	0.0935771	INF	No Filter	Trace27	11.56	14.22	Automated - ICIS	119660	Echinacea tennesseensis	Stems	C17H22O10 Sinapoyl-glucose	Level 3
4	3.6378628	218.27177	3.59454	3.785525	0	80361.2811	15130.61355	0.1909857	INF	No Filter	Trace27	64.3	42.96	Automated - ICIS	119660	Echinacea tennesseensis	Stems	C17H22O10 Sinapoyl-glucose	Level 3
5	3.8458917	230.7535	3.835659	3.898669	385.114777	5300.57974	2900.132986	0.0630099	INF	No Filter	Trace27	4.24	8.23	Automated - ICIS	119660	Echinacea tennesseensis	Stems	C17H22O10 Sinapoyl-glucose	Level 3
1	4.8670459	292.02275	4.855207	4.877278	339.071808	835.032512	1597.293049	0.0220705	INF	No Filter	Trace28	8.06	32.15	Automated - Genesis	119660	Echinacea tennesseensis	Stems		
2	5.2969876	317.81926	5.221898	5.456934	0	9523.01078	3371.358377	0.235036496	INF	No Filter	Trace28	91.94	67.85	Manual	119660	Echinacea tennesseensis	Stems		
1	0.8789517	52.737103	0.870994	0.888011	335.077942	1608.43879	3831.93959	0.0170165	INF	No Filter	Trace29	100	100	Automated - Genesis	119660	Echinacea tennesseensis	Stems		
1	0.92414	55.448402	0.913822	0.934441	137.024185	453.11929	865.2187156	0.02061918	INF	No Filter	Trace1	1.34	1.68	Automated - Genesis	119661	Echinacea tennesseensis	Leaves		
2	0.9835992	59.015951	0.9733	0.993898	137.023727	2280.71656	4354.650235	0.02059773	INF	No Filter	Trace1	6.74	8.46	Automated - Genesis	119661	Echinacea tennesseensis	Leaves		
3	1.2782357	76.694142	1.266352	1.2885	137.024124	466.216846	760.0597559	0.0221473	INF	No Filter	Trace1	1.38	1.48	Automated - Genesis	119661	Echinacea tennesseensis	Leaves		
4	1.5124061	90.744366	1.50214	1.52267	137.023849	529.873905	1013.824164	0.0205304	INF	No Filter	Trace1	1.56	1.97	Automated - Genesis	119661	Echinacea tennesseensis	Leaves		
5	1.5946137	95.676822	1.584347	1.619396	137.024109	1089.35663	1076.135611	0.035049	INF	No Filter	Trace1	3.22	2.09	Automated - Genesis	119661	Echinacea tennesseensis	Leaves		
6	1.6425593	98.553558	1.631979	1.652348	137.024506	382.526007	756.6368101	0.0203691	INF	No Filter	Trace1	1.13	1.47	Automated - Genesis	119661	Echinacea tennesseensis	Leaves		
7	2.7803986	166.82392	2.765605	2.794339	137.023773	1406.81432	1917.502414	0.0287337	INF	No Filter	Trace1	4.15	3.72	Automated - Genesis	119661	Echinacea tennesseensis	Leaves		
8	4.7299035	283.79421	4.719672	4.740137	137.023743	903.498608	1736.072028	0.0204655	INF	No Filter	Trace1	2.67	3.37	Automated - Genesis	119661	Echinacea tennesseensis	Leaves		
9	7.2304875	433.82925	7.220254	7.24072	137.023758	1003.56157	1928.613609	0.0204661	INF	No Filter	Trace1	2.96	3.74	Automated - Genesis	119661	Echinacea tennesseensis	Leaves		
10	7.7175061	463.05037	7.706766	7.728247	137.023743	1802.24649	3298.701361	0.0214813	INF	No Filter	Trace1	5.32	6.41	Automated - Genesis	119661	Echinacea tennesseensis	Leaves		
11	8.9874515	539.24709	8.976711	8.998192	137.023788	1898.58984	3475.044483	0.0214808	INF	No Filter	Trace1	5.61	6.75	Automated - Genesis	119661	Echinacea tennesseensis	Leaves		
12	9.0629977	543.77986	9.052257	9.073738	137.024719	253.123107	463.2942996	0.0214813	INF	No Filter	Trace1	0.75	0.9	Automated - Genesis	119661	Echinacea tennesseensis	Leaves	C8H8O3 Methyl salicylate	Level 2
13	9.6027609	576.16565	9.58882	9.617552	137.023849	1026.07933	1372.574366	0.0287316	INF	No Filter	Trace1	3.03	2.67	Automated - Genesis	119661	Echinacea tennesseensis	Leaves		
14	9.9485725	596.91435	9.933788	9.962514	137.02388	1041.36366	1443.343948	0.028726	INF	No Filter	Trace1	3.08	2.8	Automated - Genesis	119661	Echinacea tennesseensis	Leaves		

15	10.129175	607.7505	10.11523	10.14312	137.023758	2455.35218	3462.459077	0.027882	INF	No Filter	Trace1	7.25	6.72	Automated - Genesis	119661	Echinacea tennesseensis	Leaves		
16	10.309776	618.58656	10.29584	10.32372	137.023773	2426.92872	3422.347271	0.027881	INF	No Filter	Trace1	7.17	6.65	Automated - Genesis	119661	Echinacea tennesseensis	Leaves		
17	11.020551	661.23306	11.00661	11.03535	137.023727	1349.5502	1804.945237	0.028734	INF	No Filter	Trace1	3.99	3.5	Automated - Genesis	119661	Echinacea tennesseensis	Leaves		
18	11.321355	679.2813	11.30741	11.3353	137.023895	925.074496	1304.527341	0.027882	INF	No Filter	Trace1	2.73	2.53	Automated - Genesis	119661	Echinacea tennesseensis	Leaves		
19	11.542313	692.53878	11.52837	11.55625	137.023727	2140.79299	3018.814307	0.027881	INF	No Filter	Trace1	6.32	5.86	Automated - Genesis	119661	Echinacea tennesseensis	Leaves		
20	11.926769	715.60614	11.91283	11.94155	137.023849	949.738583	1336.876648	0.028723	INF	No Filter	Trace1	2.8	2.6	Automated - Genesis	119661	Echinacea tennesseensis	Leaves		
21	12.31905	739.143	12.30511	12.33299	137.023773	2293.98857	3234.909273	0.027882	INF	No Filter	Trace1	6.77	6.28	Automated - Genesis	119661	Echinacea tennesseensis	Leaves		
22	12.504316	750.25896	12.49038	12.51826	137.023727	1341.59977	1891.864516	0.027881	INF	No Filter	Trace1	3.96	3.67	Automated - Genesis	119661	Echinacea tennesseensis	Leaves		
23	13.067884	784.07304	13.05394	13.08182	137.023727	1117.82469	1576.306814	0.027881	INF	No Filter	Trace1	3.3	3.06	Automated - Genesis	119661	Echinacea tennesseensis	Leaves		
24	13.112877	786.77262	13.09809	13.12682	137.023773	2410.9907	3286.886945	0.028728	INF	No Filter	Trace1	7.12	6.38	Automated - Genesis	119661	Echinacea tennesseensis	Leaves		
25	13.647718	818.86308	13.63378	13.66166	137.023743	1912.35716	2696.77797	0.027882	INF	No Filter	Trace1	5.65	5.24	Automated - Genesis	119661	Echinacea tennesseensis	Leaves		
1	1.8200494	109.20296	1.707545	2.226821	137.023315	362689.098	39172.17393	0.5192756	16.76560649	No Filter	Trace3	96.42	92.06	Manual	119661	Echinacea tennesseensis	Leaves		
2	5.6403309	338.41985	5.528467	5.988321	137.023376	13470.6392	3380.242181	0.459854015	1.456791073	No Filter	Trace3	3.58	7.94	Manual	119661	Echinacea tennesseensis	Leaves		
1	0.9154092	54.924549	0.893226	0.934441	0	2003.20928	1549.148791	0.0412152	INF	No Filter	Trace4	7	5.72	Automated - Genesis	119661	Echinacea tennesseensis	Leaves		
2	1.5294265	91.76559	1.500588	1.604404	93.0333557	7314.33501	2513.55842	0.1038155	INF	No Filter	Trace4	25.56	9.28	Automated - Genesis	119661	Echinacea tennesseensis	Leaves		
3	3.830532	229.83192	3.820301	3.840765	93.0330658	373.795452	718.5347417	0.0204646	INF	No Filter	Trace4	1.31	2.65	Automated - Genesis	119661	Echinacea tennesseensis	Leaves		
4	3.9037665	234.22599	3.893534	3.914	93.0330811	327.379915	629.371703	0.0204667	INF	No Filter	Trace4	1.14	2.32	Automated - Genesis	119661	Echinacea tennesseensis	Leaves		
5	4.065574	243.93444	4.055343	4.07581	93.0330353	291.72698	560.7774882	0.020467	INF	No Filter	Trace4	1.02	2.07	Automated - Genesis	119661	Echinacea tennesseensis	Leaves		
6	4.086041	245.16246	4.07581	4.096287	93.0331726	272.905215	523.8407352	0.0204776	INF	No Filter	Trace4	0.95	1.93	Automated - Genesis	119661	Echinacea tennesseensis	Leaves		
7	4.1439406	248.63644	4.118359	4.154174	93.0334396	552.575754	609.1387952	0.0358146	INF	No Filter	Trace4	1.93	2.25	Automated - Genesis	119661	Echinacea tennesseensis	Leaves		
8	4.3211352	259.26811	4.30067	4.331368	93.0333023	739.202714	730.4123192	0.0306978	INF	No Filter	Trace4	2.58	2.7	Automated - Genesis	119661	Echinacea tennesseensis	Leaves		
9	4.3652016	261.9121	4.346715	4.379015	0	1188.67809	1176.230017	0.0323	INF	No Filter	Trace4	4.15	4.34	Automated - Genesis	119661	Echinacea tennesseensis	Leaves		
10	4.4224962	265.34977	4.411755	4.433237	93.0332642	323.232527	591.6174054	0.0214812	INF	No Filter	Trace4	1.13	2.18	Automated - Genesis	119661	Echinacea tennesseensis	Leaves		
11	4.4455766	266.7346	4.433237	4.470638	93.0329742	679.779446	651.9153716	0.0374013	INF	No Filter	Trace4	2.38	2.41	Automated - Genesis	119661	Echinacea tennesseensis	Leaves		
12	4.5271832	271.63099	4.481378	4.540264	0	1319.71428	957.4290923	0.0588862	INF	No Filter	Trace4	4.61	3.54	Automated - Genesis	119661	Echinacea tennesseensis	Leaves		
13	4.5617455	273.70473	4.551005	4.572486	93.0325546	367.199088	672.086654	0.0214813	INF	No Filter	Trace4	1.28	2.48	Automated - Genesis	119661	Echinacea tennesseensis	Leaves		
14	4.5939676	275.63806	4.583227	4.603903	93.0329819	348.750112	638.2072699	0.0206764	INF	No Filter	Trace4	1.22	2.36	Automated - Genesis	119661	Echinacea tennesseensis	Leaves		
15	4.6452036	278.71222	4.612956	4.65902	0	1200.96348	1032.607404	0.046064	INF	No Filter	Trace4	4.2	3.81	Automated - Genesis	119661	Echinacea tennesseensis	Leaves		
16	4.6845998	281.07599	4.674368	4.696432	93.0331726	310.552015	548.4444394	0.0220635	INF	No Filter	Trace4	1.09	2.03	Automated - Genesis	119661	Echinacea tennesseensis	Leaves		
17	4.7554854	285.32912	4.727128	4.76806	0	816.773657	710.8145499	0.0409316	INF	No Filter	Trace4	2.85	2.62	Automated - Genesis	119661	Echinacea tennesseensis	Leaves		
18	4.8003679	288.02207	4.790135	4.8106	93.0334473	311.851994	599.5172164	0.0204648	INF	No Filter	Trace4	1.09	2.21	Automated - Genesis	119661	Echinacea tennesseensis	Leaves		
19	4.8310651	289.86391	4.820833	4.841297	93.0333328	425.706398	818.395404	0.0204643	INF	No Filter	Trace4	1.49	3.02	Automated - Genesis	119661	Echinacea tennesseensis	Leaves		
20	4.8838353	293.03012	4.873603	4.894068	93.0334244	329.599202	633.6375347	0.0204657	INF	No Filter	Trace4	1.15	2.34	Automated - Genesis	119661	Echinacea tennesseensis	Leaves		
21	4.9094156	294.56494	4.899184	4.919651	93.0332642	282.202622	542.3427561	0.0204667	INF	No Filter	Trace4	0.99	2	Automated - Genesis	119661	Echinacea tennesseensis	Leaves		
22	4.9299005	295.79403	4.919651	4.941742	93.0333786	323.215475	617.9187361	0.0220917	INF	No Filter	Trace4	1.13	2.28	Automated - Genesis	119661	Echinacea tennesseensis	Leaves		
23	4.9700975	298.20585	4.957089	4.982671	0	793.269166	1175.953203	0.025582	INF	No Filter	Trace4	2.77	4.34	Automated - Genesis	119661	Echinacea tennesseensis	Leaves		
24	5.0184829	301.10897	5.008251	5.055919	93.033287	1065.79988	713.3411692	0.0476675	INF	No Filter	Trace4	3.73	2.63	Automated - Genesis	119661	Echinacea tennesseensis	Leaves		
25	5.0661501	303.96901	5.055919	5.076395	93.0333557	326.608245	627.0649738	0.0204761	INF	No Filter	Trace4	1.14	2.32	Automated - Genesis	119661	Echinacea tennesseensis	Leaves		
26	5.10475	306.285	5.091743	5.124037	0	1045.11333	1038.215538	0.0322934	INF	No Filter	Trace4	3.65	3.83	Automated - Genesis	119661	Echinacea tennesseensis	Leaves		
27	5.1496172	308.97703	5.139386	5.159851	93.0332336	525.701996	1010.526149	0.0204649	INF	No Filter	Trace4	1.84	3.73	Automated - Genesis	119661	Echinacea tennesseensis	Leaves		
28	5.1728583	310.3715	5.159851	5.222853	0	2028.69724	1199.065467	0.0630021	INF	No Filter	Trace4	7.09	4.43	Automated - Genesis	119661	Echinacea tennesseensis	Leaves		
29	5.2433172	314.59903	5.222853	5.268899	93.0333405	1087.74687	944.2842103	0.0460459	INF	No Filter	Trace4	3.8	3.49	Automated - Genesis	119661	Echinacea tennesseensis	Leaves		
30	5.2976184	317.8571	5.28585	5.316547	0	986.826009	1104.796297	0.0306969	INF	No Filter	Trace4	3.45	4.08	Automated - Genesis	119661	Echinacea tennesseensis	Leaves		
31	5.3267788	319.60673	5.316547	5.337014	93.033226	335.63547	645.0459624	0.0204665	INF	No Filter	Trace4	1.17	2.38	Automated - Genesis	119661	Echinacea tennesseensis	Leaves		
32	5.3472621	320.83573	5.337014	5.359094	93.0332718	312.232	596.9720487	0.0220804	INF	No Filter	Trace4	1.09	2.2	Automated - Genesis	119661	Echinacea tennesseensis	Leaves		
1	0.8793481	52.760887	0.865137	0.890885	0	5239.75534	7582.924686	0.025748	INF	No Filter	Trace5	62.37	55.62	Automated - ICIS	119661	Echinacea tennesseensis	Leaves		
2	1.0574247	63.445482	1.047125	1.067723	225.040649	1161.596	2217.816274	0.0205982	INF	No Filter	Trace5	13.83	16.27	Automated - ICIS	119661	Echinacea tennesseensis	Leaves		
3	1.1806926	70.841556	1.17116	1.192908	225.039642	1010.90952	1937.96992	0.0217484	INF	No Filter	Trace5	12.03	14.21	Automated - ICIS	119661	Echinacea tennesseensis	Leaves		
4	1.2280796	73.684776	1.217815	1.238344	225.039719	989.470467	1895.448581	0.0205295	INF	No Filter	Trace5	11.78	13.9	Automated - ICIS	119661	Echinacea tennesseensis	Leaves		
1	0.6313487	37.880922	0.618267	0.643329	0	2512.831	4079.882365	0.02506138	INF	No Filter	Trace6	100	100	Automated - Genesis	119661	Echinacea tennesseensis	Leaves		
1	2.4136777	144.82066	2.411679	2.881752	179.034119	361859.476	44371.19675	0.470072993	INF	No Filter	Trace7	100	100	Manual	119661	Echinacea tennesseensis	Leaves		
1	0.7640437	45.842619	0.753746	0.774342	163.039368	5434.26197	10375.80202	0.02059605	6.097806179	No Filter	Trace8	100	100	Automated - Genesis	119661	Echinacea tennesseensis	Leaves		
1	0.6241883	37.451297	0.602302	0.675551	0	73219.2938	33964.63194	0.0732487	INF	No Filter	Trace9	78.45	66.6	Automated - Genesis	119661	Echinacea tennesseensis	Leaves		
2	0.7731025	46.386148	0.758895	0.796561	0	9703.72809	8902.070835	0.0376667	INF	No Filter	Trace9	10.4	17.45	Automated - Genesis	119661	Echinacea tennesseensis	Leaves		
3	0.8857362	53.144174	0.875438	0.896035	223.060394	2036.90853	3889.12499	0.02059638	INF	No Filter	Trace9	2.18	7.63	Automated - Genesis	119661	Echinacea tennesseensis	Leaves		
4	0.9321005	55.926028	0.911482	0.983599	223.060913	8370.71603	4244.81648	0.07211766	INF	No Filter	Trace9	8.97	8.32	Automated - Genesis	119661	Echinacea tennesseensis	Leaves		
1	1.5946137	95.676822	1.532847	1.829197	0	149304.546	23593.39751	0.296350365	INF	No Filter	Trace10	100	100	Manual	119661	Echinacea tennesseensis	Leaves	C16H18O9 chlorogenic acid (5-o-caffeoylquinic acid); C16H18O9 cis 5-o-caffeoylquinic acid	Level 4
1	1.5124061	90.744366	1.359316	1.584165	0	2383233.97	300854.0002	0.224849218	205.0154023	No Filter	Trace12	100	100	Manual	119661	Echinacea tennesseensis	Leaves	C13H12O9 caftaric acid	Level 4
1	0.6218478	37.310865	0.602302	0.643329	473.072144	36263.9152	39311.03	0.04102691	INF	No Filter	Trace13	100	100	Automated - Genesis	119661	Echinacea tennesseensis	Leaves	C22H18O12 cichoric acid	Level 4
1	0.92414	55.448402	0.915409	0.9321	137.024185	453.675991	1080.024291	0.01669132	INF	No Filter	Trace17	1.34	1.68	Automated - Genesis	119661	Echinacea tennesseensis	Leaves		
2	0.9835992	59.015951	0.975641	0.992658	137.023727	2281.59151	5435.767801	0.01701685	INF	No Filter	Trace17	6.73	8.46	Automated - Genesis	119661	Echinacea tennesseensis	Leaves		
3	1.2782357	76.694142	1.267905	1.286159	137.024124	481.331711	948.7577933	0.0182544	INF	No Filter	Trace17	1.42	1.48	Automated - Genesis	119661	Echinacea tennesseensis	Leaves		
4	1.5124061	90.744366	1.50338	1.521119	137.023849	528.999534	1265.523624	0.017739	INF	No Filter	Trace17	1.56	1.97	Automated - Genesis	119661	Echinacea tennesseensis	Leaves		
5	1.5946137	95.676822	1.585901	1.617841	137.024109	1111.99445	1343.30497	0.0319397	INF	No Filter	Trace17	3.28	2.09	Automated - Genesis	119661	Echinacea tennesseensis	Leaves		
6	1.6425593	98.553558	1.63432	1.650796	137.024506	386.831962	944.4850417	0.0164765	INF	No Filter	Trace17	1.14	1.47	Automated - Genesis	119661	Echinacea tennesseensis	Leaves		
7	2.7803986	166.82392	2.767929	2.792016	137.023773	1400.06494	2393.555697	0.0240871	INF	No Filter	Trace17	4.13	3.72	Automated - Genesis	119661	Echinacea tennesseensis	Leaves		
8	4.7299035	283.79421	4.722012	4.738898	137.023743	903.916765	2167.082067	0.0168855	INF	No Filter	Trace17	2.67	3.37	Automated - Genesis	119661	Echinacea tennesseensis	Leaves		
9	7.2304875	433.82925	7.222595	7.239481	137.023758	1003.9962	2407.42544	0.0168856	INF	No Filter	Trace17	2.96	3.74	Automated - Genesis	119661	Echinacea tennesseensis	Leaves		
10	7.7175061	463.05037	7.709106	7.727007	137.023743	1802.23955	4117.661276	0.0179009	INF	No Filter	Trace17	5.32	6.41	Automated - Genesis	119661	Echinacea tennesseensis	Leaves		
11	8.9874515	539.24709	8.979052	8.996953	137.023788	1898.59206	4337.78464	0.017901	INF	No Filter	Trace17	5.6	6.75	Automated - Genesis	119661	Echinacea tennesseensis	Leaves		
12	9.0629977	543.77986	9.053497	9.071398	137.024719	253.123423	578.3151573	0.017901	INF	No Filter	Trace17	0.75	0.9	Automated - Genesis	119661	Echinacea tennesseensis	Leaves		
13	9.6027609	576.16565	9.591144	9.615229	137.023849	1030.24987	1713.339795	0.024085	INF	No Filter	Trace17	3.04	2.67	Automated - Genesis	119661	Echinacea tennesseensis	Leaves		
14	9.9485725	596.91435	9.936111	9.96019	137.02388	1034.29889	1801.679156	0.0240792	INF	No Filter	Trace17	3.05	2.8	Automated - Genesis	119661	Echinacea tennesseensis	Leaves		

15	10.129175	607.7505	10.11756	10.14079	137.023758	2455.32552	4322.074689	0.023235	INF	No Filter	Trace17	7.25	6.72	Automated - Genesis	119661	Echinacea tennesseensis	Leaves			
16	10.309776	618.58656	10.29816	10.32139	137.023773	2426.93988	4272.004431	0.023235	INF	No Filter	Trace17	7.16	6.65	Automated - Genesis	119661	Echinacea tennesseensis	Leaves			
17	11.020551	661.23306	11.00893	11.03302	137.023727	1355.05477	2253.054246	0.024088	INF	No Filter	Trace17	4	3.5	Automated - Genesis	119661	Echinacea tennesseensis	Leaves			
18	11.321355	679.2813	11.30974	11.33297	137.023895	925.061469	1628.398914	0.023235	INF	No Filter	Trace17	2.73	2.53	Automated - Genesis	119661	Echinacea tennesseensis	Leaves			
19	11.542313	692.53878	11.5307	11.55393	137.023727	2140.79752	3768.287399	0.023235	INF	No Filter	Trace17	6.32	5.86	Automated - Genesis	119661	Echinacea tennesseensis	Leaves			
20	11.926769	715.60614	11.91515	11.93839	137.023849	948.043228	1668.779499	0.023235	INF	No Filter	Trace17	2.8	2.6	Automated - Genesis	119661	Echinacea tennesseensis	Leaves			
21	12.31905	739.143	12.30743	12.33067	137.023773	2293.96366	4038.031693	0.023235	INF	No Filter	Trace17	6.77	6.28	Automated - Genesis	119661	Echinacea tennesseensis	Leaves			
22	12.504316	750.25896	12.4927	12.51593	137.023727	1341.60594	2361.552746	0.023235	INF	No Filter	Trace17	3.96	3.67	Automated - Genesis	119661	Echinacea tennesseensis	Leaves			
23	13.067884	784.07304	13.05627	13.0795	137.023727	1117.82983	1967.652362	0.023235	INF	No Filter	Trace17	3.3	3.06	Automated - Genesis	119661	Echinacea tennesseensis	Leaves			
24	13.112877	786.77262	13.10041	13.12449	137.023773	2399.50964	4102.913725	0.024082	INF	No Filter	Trace17	7.08	6.38	Automated - Genesis	119661	Echinacea tennesseensis	Leaves			
25	13.647718	818.86308	13.6361	13.65934	137.023743	1912.34114	3366.299947	0.023235	INF	No Filter	Trace17	5.64	5.24	Automated - Genesis	119661	Echinacea tennesseensis	Leaves			
1	0.691505	41.4903	0.685052	0.697426	153.018738	4206.1724	8977.932234	0.01237408	22.96112217	No Filter	Trace18	100	100	Automated - ICIS	119661	Echinacea tennesseensis	Leaves			
1	8.9874515	539.24709	8.976711	8.998192	431.098389	828.999777	1517.342529	0.0214808	INF	No Filter	Trace21	6.15	8.14	Automated - Genesis	119661	Echinacea tennesseensis	Leaves			
2	9.1612628	549.67577	9.137441	9.176823	0	3052.87708	2361.72886	0.0393818	INF	No Filter	Trace21	22.65	12.67	Automated - Genesis	119661	Echinacea tennesseensis	Leaves			
3	9.2106483	552.6389	9.199908	9.221389	431.097168	953.312361	1744.853745	0.0214812	INF	No Filter	Trace21	7.07	9.36	Automated - Genesis	119661	Echinacea tennesseensis	Leaves			
4	9.3124946	558.74968	9.301754	9.323235	431.099579	1337.77922	2448.557929	0.0214813	INF	No Filter	Trace21	9.92	13.14	Automated - Genesis	119661	Echinacea tennesseensis	Leaves			
5	9.3892795	563.35677	9.378539	9.40002	431.098175	904.094845	1654.772757	0.0214813	INF	No Filter	Trace21	6.71	8.88	Automated - Genesis	119661	Echinacea tennesseensis	Leaves			
6	9.5423509	572.54105	9.52841	9.556292	431.10025	1341.87132	1892.220638	0.0278817	INF	No Filter	Trace21	9.95	10.15	Automated - Genesis	119661	Echinacea tennesseensis	Leaves			
7	9.9384343	596.30606	9.924494	9.95322	431.096802	1371.64012	1812.090329	0.028726	INF	No Filter	Trace21	10.17	9.72	Automated - Genesis	119661	Echinacea tennesseensis	Leaves			
8	10.46947	628.1682	10.45553	10.48341	431.098694	1091.56319	1539.316978	0.027882	INF	No Filter	Trace21	8.1	8.26	Automated - Genesis	119661	Echinacea tennesseensis	Leaves			
9	10.793493	647.60958	10.77955	10.80743	431.099548	1347.64301	1900.397043	0.027882	INF	No Filter	Trace21	10	10.2	Automated - Genesis	119661	Echinacea tennesseensis	Leaves			
10	11.579489	694.76934	11.56555	11.59343	431.097595	1251.30798	1764.559861	0.027882	INF	No Filter	Trace21	9.28	9.47	Automated - Genesis	119661	Echinacea tennesseensis	Leaves			
1	10.887274	653.23644	10.87333	10.90121	447.092743	970.435197	1368.47666	0.027882	INF	No Filter	Trace24	100	100	Automated - Genesis	119661	Echinacea tennesseensis	Leaves			
1	13.936071	836.16426	13.92213	13.95086	216.052383	1440.08263	1926.977368	0.028727	INF	No Filter	Trace25	100	100	Automated - Genesis	119661	Echinacea tennesseensis	Leaves			
1	0.6146874	36.881241	0.602302	0.625427	193.050095	25279.8453	46061.63607	0.02312556	INF	No Filter	Trace26	0.96	13.3	Automated - Genesis	119661	Echinacea tennesseensis	Leaves			
2	5.6052678	336.31607	5.501074	5.959395	0	2597006.92	300214.5579	0.4583209	42.82229482	No Filter	Trace26	99.04	86.7	Automated - Genesis	119661	Echinacea tennesseensis	Leaves	C10H10O4	Ferulic acid	Level 4
1	2.0863115	125.17869	2.041359	2.193482	0	44711.0849	10836.75654	0.1521224	INF	No Filter	Trace27	10.61	11.8	Automated - ICIS	119661	Echinacea tennesseensis	Leaves	C17H22O10	Sinapoyl-glucose	Level 3
2	2.4252949	145.51769	2.354218	2.444737	385.113953	13803.3581	6812.489872	0.0905192	INF	No Filter	Trace27	3.28	7.42	Automated - ICIS	119661	Echinacea tennesseensis	Leaves	C17H22O10	Sinapoyl-glucose	Level 3
3	2.5005002	150.03001	2.451707	2.603582	385.114929	52869.9253	13891.83572	0.1518745	INF	No Filter	Trace27	12.55	15.13	Automated - ICIS	119661	Echinacea tennesseensis	Leaves	C17H22O10	Sinapoyl-glucose	Level 3
4	2.9392454	176.35472	2.904393	3.007471	0	18871.6632	7631.445381	0.1030777	INF	No Filter	Trace27	4.48	8.31	Automated - ICIS	119661	Echinacea tennesseensis	Leaves	C17H22O10	Sinapoyl-glucose	Level 3
5	3.1369642	188.21785	3.103591	3.16717	0	6555.79519	3266.262157	0.0635782	INF	No Filter	Trace27	1.56	3.56	Automated - ICIS	119661	Echinacea tennesseensis	Leaves			
6	3.644121	218.64726	3.582898	3.802598	0	168676.098	28139.02682	0.2197008	INF	No Filter	Trace27	40.03	30.65	Automated - ICIS	119661	Echinacea tennesseensis	Leaves	C17H22O10	Sinapoyl-glucose	Level 3
7	3.873069	232.38414	3.807714	4.055343	0	115892.055	21239.592	0.2476284	INF	No Filter	Trace27	27.5	23.13	Automated - ICIS	119661	Echinacea tennesseensis	Leaves	C17H22O10	Sinapoyl-glucose	Level 3
1	1.2936488	77.618928	1.246891	1.379757	0	48044.5402	16796.52983	0.132865447	INF	No Filter	Trace28	97.83	85.02	Manual	119661	Echinacea tennesseensis	Leaves	C15H16O9	z sinapoyl-malate	Level 4
2	4.8670459	292.02275	4.855207	4.877278	339.071808	835.032512	1597.293049	0.0220705	INF	No Filter	Trace28	1.7	8.08	Automated - Genesis	119661	Echinacea tennesseensis	Leaves			
3	5.4568721	327.41233	5.221898	5.456934	0	230.828196	1363.227456	0.235036496	INF	No Filter	Trace28	0.47	6.9	Manual	119661	Echinacea tennesseensis	Leaves			
1	0.7176936	43.061618	0.709736	0.726752	335.078796	17759.0092	42309.8429	0.01701643	INF	No Filter	Trace29	48.9	48.9	Automated - Genesis	119661	Echinacea tennesseensis	Leaves			
2	0.8017107	48.102642	0.793753	0.810769	335.078644	18556.527	44209.21619	0.01701633	INF	No Filter	Trace29	51.1	51.1	Automated - Genesis	119661	Echinacea tennesseensis	Leaves			
1	0.9086921	54.521527	0.898394	0.929293	137.024246	1445.81012	1411.995141	0.03089957	INF	No Filter	Trace1	6.8	4.31	Automated - Genesis	119662	Echinacea tennesseensis	Flowers			
2	3.1238437	187.43062	3.109903	3.140108	137.023727	2946.20552	3988.320549	0.0302053	INF	No Filter	Trace1	13.86	12.18	Automated - Genesis	119662	Echinacea tennesseensis	Flowers			
3	3.3139568	198.83741	3.303216	3.324698	137.023849	931.190331	1704.373531	0.0214812	INF	No Filter	Trace1	4.38	5.2	Automated - Genesis	119662	Echinacea tennesseensis	Flowers			
4	3.4374018	206.24411	3.426661	3.44974	137.023727	1090.40894	1712.340295	0.023079	INF	No Filter	Trace1	5.13	5.23	Automated - Genesis	119662	Echinacea tennesseensis	Flowers			
5	4.096288	245.77728	4.086055	4.10652	137.023758	1323.10493	2542.717713	0.0204642	INF	No Filter	Trace1	6.22	7.76	Automated - Genesis	119662	Echinacea tennesseensis	Flowers			
6	4.2529731	255.17839	4.242741	4.263205	137.023758	1644.77208	3160.956287	0.020464	INF	No Filter	Trace1	7.74	9.65	Automated - Genesis	119662	Echinacea tennesseensis	Flowers			
7	4.541057	272.46342	4.530317	4.551798	137.024628	255.951348	468.4718417	0.0214813	INF	No Filter	Trace1	1.2	1.43	Automated - Genesis	119662	Echinacea tennesseensis	Flowers			
8	4.7102186	282.61312	4.699987	4.72045	137.023743	1826.87155	3510.70966	0.0204637	INF	No Filter	Trace1	8.59	10.72	Automated - Genesis	119662	Echinacea tennesseensis	Flowers			
9	6.4387048	386.32229	6.427964	6.451047	137.02388	1124.50492	1765.229678	0.0230829	INF	No Filter	Trace1	5.29	5.39	Automated - Genesis	119662	Echinacea tennesseensis	Flowers			
10	8.635988	518.15928	8.625247	8.646728	137.023773	853.656819	1562.460199	0.0214809	INF	No Filter	Trace1	4.02	4.77	Automated - Genesis	119662	Echinacea tennesseensis	Flowers			
11	11.358059	681.48354	11.34327	11.372	137.023727	1044.30056	1378.754089	0.028735	INF	No Filter	Trace1	4.91	4.21	Automated - Genesis	119662	Echinacea tennesseensis	Flowers			
12	11.913822	714.82932	11.89988	11.92776	137.023773	2261.9728	3189.761654	0.027882	INF	No Filter	Trace1	10.64	9.74	Automated - Genesis	119662	Echinacea tennesseensis	Flowers			
13	12.810823	768.64938	12.79688	12.82476	137.023773	2490.8388	3512.314992	0.027882	INF	No Filter	Trace1	11.72	10.72	Automated - Genesis	119662	Echinacea tennesseensis	Flowers			
14	12.993763	779.62578	12.97982	13.0077	137.023773	2018.43421	2846.265468	0.027882	INF	No Filter	Trace1	9.49	8.69	Automated - Genesis	119662	Echinacea tennesseensis	Flowers			
1	5.6375594	338.25356	5.626819	5.6483	103.055176	861.160784	1576.194314	0.0214813	INF	No Filter	Trace2	63.08	62.52	Automated - Genesis	119662	Echinacea tennesseensis	Flowers			
2	7.4666281	447.99769	7.456396	7.47686	89.0398788	251.035379	481.9159717	0.020464	INF	No Filter	Trace2	18.39	19.11	Automated - Genesis	119662	Echinacea tennesseensis	Flowers			
3	8.3311687	499.87012	8.320428	8.34191	77.0400391	252.995038	463.0593647	0.0214812	INF	No Filter	Trace2	18.53	18.37	Automated - Genesis	119662	Echinacea tennesseensis	Flowers			
1	1.8047892	108.28735	1.707545	2.226821	0	1497724.52	157248.0577	0.5192756	79.55431637	No Filter	Trace3	99.99	99.56	Manual	119662	Echinacea tennesseensis	Flowers			
2	5.5308873	331.85324	5.528467	5.988321	0	146.607459	695.5890022	0.459854015	0.343373943	No Filter	Trace3	0.01	0.44	Manual	119662	Echinacea tennesseensis	Flowers			
1	0.9063512	54.381074	0.893245	0.934443	0	3293.28306	2732.237698	0.0411985	INF	No Filter	Trace4	13.92	10.19	Automated - Genesis	119662	Echinacea tennesseensis	Flowers			
2	1.552415	93.1449	1.54215	1.562679	93.033432	340.034629	650.6512828	0.0205291	INF	No Filter	Trace4	1.44	2.43	Automated - Genesis	119662	Echinacea tennesseensis	Flowers			
3	3.8254122	229.52473	3.812404	3.837986	0	686.672121	1026.697112	0.0255815	INF	No Filter	Trace4	2.9	3.83	Automated - Genesis	119662	Echinacea tennesseensis	Flowers			
4	4.0269882	241.61929	4.015153	4.037219	93.0334625	302.589596	495.3237695	0.0220661	INF	No Filter	Trace4	1.28	1.85	Automated - Genesis	119662	Echinacea tennesseensis	Flowers			
5	4.0628153	243.76892	4.052584	4.073048	93.032692	252.737514	485.885867	0.020464	INF	No Filter	Trace4	1.07	1.81	Automated - Genesis	119662	Echinacea tennesseensis	Flowers			
6	4.1155715	246.93429	4.103744	4.125804	93.0328751	273.109868	502.5146003	0.0220593	INF	No Filter	Trace4	1.15	1.87	Automated - Genesis	119662	Echinacea tennesseensis	Flowers			
7	4.1643919	249.86351	4.151384	4.176965	0	769.213824	1151.515492	0.0255811	INF	No Filter	Trace4	3.25	4.29	Automated - Genesis	119662	Echinacea tennesseensis	Flowers			
8	4.1939162	251.63497	4.182081	4.204149	93.0330887	473.443477	774.8876739	0.0220685	INF	No Filter	Trace4	2	2.89	Automated - Genesis	119662	Echinacea tennesseensis	Flowers			
9	4.2978454	257.87072	4.287613	4.308077	93.0334244	278.812338	536.0254798	0.020464	INF	No Filter	Trace4	1.18	2	Automated - Genesis	119662	Echinacea tennesseensis	Flowers			
10	4.423268	265.39608	4.386439	4.434009	93.0329514	994.966677	754.4955671	0.0475701	INF	No Filter	Trace4	4.2	2.81	Automated - Genesis	119662	Echinacea tennesseensis	Flowers			
11	4.4476121	266.85673	4.434009	4.460693	0	635.169395	979.7104082	0.0266846	INF	No Filter	Trace4	2.68	3.65	Automated - Genesis	119662	Echinacea tennesseensis	Flowers			
12	4.4857546	269.14528	4.475014	4.496495	93.033371	525.25288	961.3723984	0.0214812	INF	No Filter	Trace4	2.22	3.58	Automated - Genesis	119662	Echinacea tennesseensis	Flowers			
13	4.541057	272.46342	4.530317	4.59834	93.0333405	1112.44216	552.8604951	0.0680235	INF	No Filter	Trace4	4.7	2.06	Automated - Genesis	119662	Echinacea tennesseensis	Flowers			
14	4.6188731	277.13239	4.608641	4.629105	93.0331192	338.035536	649.8520243	0.0204637	INF	No Filter	Trace4	1.43	2.42	Automated - Genesis	119662	Echinacea tennesseensis	Flowers			
15	4.651105	279.0663	4.639337	4.692095	0	1467.8345	1080.594289	0.0527577	INF	No Filter	Trace4	6.2	4.03	Automated - Genesis	119662	Echinacea tennesseensis	Flowers			
16	4.7499118	284.99471	4.707443	4.76374	0	1798.74842	1093.91983	0.056297	INF	No Filter	Trace4	7.6	4.08	Automated - Genesis	119662	Echinacea tennesseensis	Flowers			
17	4.7755692	286.53415	4.76374	4.785801	93.0334396	302.560808	495.5678714	0.0220608	INF	No Filter	Trace4	1.28	1.85	Automated - Genesis	119662	Echinacea tennesseensis	Flowers			
18	4.8346208	290.07725	4.821613	4.847194	0	761.662321	1172.170599	0.0255802	INF	No Filter	Trace4	3.22	4.37	Automated - Genesis	119662	Echinacea tennesseensis	Flowers			
19	4.8641455	291.84873	4.852309	4.884623	93.0333023	654.938092	640.3301788	0.0323141	INF	No Filter	Trace4	2.77	2.39	Automated - Genesis	119662	Echinacea tennesseensis	Flowers			

20	4.9078768	294.47261	4.894859	4.920449	0	848.195615	1310.221869	0.02559	INF	No Filter	Trace4	3.58	4.88	Automated - Genesis	119662	Echinacea tennesseensis	Flowers		
21	4.9425113	296.55068	4.930681	4.952743	93.0332184	401.775406	657.9996808	0.0220621	INF	No Filter	Trace4	1.7	2.45	Automated - Genesis	119662	Echinacea tennesseensis	Flowers		
22	5.0515602	303.09361	5.041324	5.061792	93.0334167	412.234114	792.5164762	0.0204681	INF	No Filter	Trace4	1.74	2.95	Automated - Genesis	119662	Echinacea tennesseensis	Flowers		
23	5.0837925	305.02755	5.072024	5.097604	0	1014.82806	1510.791317	0.0255802	INF	No Filter	Trace4	4.29	5.63	Automated - Genesis	119662	Echinacea tennesseensis	Flowers		
24	5.1429146	308.57488	5.129907	5.155486	0	586.323904	852.4765744	0.0255792	INF	No Filter	Trace4	2.48	3.18	Automated - Genesis	119662	Echinacea tennesseensis	Flowers		
25	5.205925	312.3555	5.192918	5.233845	0	1337.95077	1444.842558	0.040927	INF	No Filter	Trace4	5.65	5.39	Automated - Genesis	119662	Echinacea tennesseensis	Flowers		
26	5.2543092	315.25855	5.233845	5.264541	93.0329361	673.869487	840.122008	0.0306968	INF	No Filter	Trace4	2.85	3.13	Automated - Genesis	119662	Echinacea tennesseensis	Flowers		
27	5.2945802	317.67481	5.281572	5.307152	0	804.722748	1177.587396	0.0255801	INF	No Filter	Trace4	3.4	4.39	Automated - Genesis	119662	Echinacea tennesseensis	Flowers		
28	5.3614422	321.68653	5.307152	5.375256	0	2009.64148	903.9215306	0.0681032	INF	No Filter	Trace4	8.49	3.37	Automated - Genesis	119662	Echinacea tennesseensis	Flowers		
29	5.3854869	323.12921	5.375256	5.39572	93.0328674	310.536317	596.9666732	0.0204641	INF	No Filter	Trace4	1.31	2.23	Automated - Genesis	119662	Echinacea tennesseensis	Flowers		
1	1.5133136	90.798816	1.503043	1.525201	174.076691	395.688295	754.6915781	0.0221575	INF	No Filter	Trace6	100	100	Automated - Genesis	119662	Echinacea tennesseensis	Flowers		
1	2.4827515	148.96509	2.411679	2.881752	179.03418	745136.554	104985.3767	0.470072993	INF	No Filter	Trace7	100	100	Manual	119662	Echinacea tennesseensis	Flowers		
1	0.6862887	41.17732	0.675548	0.69866	163.039383	8688.17894	13603.08587	0.02311237	7.081087244	No Filter	Trace8	100	100	Automated - Genesis	119662	Echinacea tennesseensis	Flowers		
1	0.6182647	37.09588	0.602289	0.712539	223.060593	80326.4681	28623.79437	0.11025004	INF	No Filter	Trace9	79.65	59.73	Automated - Genesis	119662	Echinacea tennesseensis	Flowers		
2	0.767959	46.077537	0.727986	0.781128	0	12548.085	7499.711964	0.05314117	INF	No Filter	Trace9	12.44	15.65	Automated - Genesis	119662	Echinacea tennesseensis	Flowers		
3	0.8120234	48.721405	0.801725	0.832626	223.061356	3643.04413	3518.080519	0.03090073	INF	No Filter	Trace9	3.61	7.34	Automated - Genesis	119662	Echinacea tennesseensis	Flowers		
4	0.8429292	50.575754	0.832626	0.853227	223.061768	2355.51045	4496.927368	0.02060095	INF	No Filter	Trace9	2.34	9.38	Automated - Genesis	119662	Echinacea tennesseensis	Flowers		
5	0.9543409	57.260452	0.944043	0.96464	223.061234	1981.98161	3784.243153	0.02059684	INF	No Filter	Trace9	1.97	7.9	Automated - Genesis	119662	Echinacea tennesseensis	Flowers		
1	1.5947131	95.682786	1.532847	1.829197	353.087799	161720.705	26728.54078	0.296350365	INF	No Filter	Trace10	100	100	Manual	119662	Echinacea tennesseensis	Flowers	C16H18O9 chlorogenic acid (5-o-caffeoylquinic acid); C16H18O9 cis 5-o-caffeoylquinic acid	Level 4
1	1.5421502	92.529012	1.359316	1.584165	0	8598527.31	1033005.68	0.224849218	237.9737616	No Filter	Trace12	100	100	Manual	119662	Echinacea tennesseensis	Flowers	C13H12O9 caftaric acid	Level 4
1	0.9086921	54.521527	0.899963	0.926953	137.024246	1447.69193	1762.547463	0.02699005	INF	No Filter	Trace17	6.78	4.32	Automated - Genesis	119662	Echinacea tennesseensis	Flowers		
2	3.1238437	187.43062	3.112227	3.137785	137.023727	2946.20957	4872.235112	0.0255583	INF	No Filter	Trace17	13.8	11.95	Automated - Genesis	119662	Echinacea tennesseensis	Flowers		
3	3.3139568	198.83741	3.305557	3.323458	137.023849	931.189631	2127.513867	0.0179009	INF	No Filter	Trace17	4.36	5.22	Automated - Genesis	119662	Echinacea tennesseensis	Flowers		
4	3.4374018	206.24411	3.429002	3.448501	137.023727	1122.92741	2137.458518	0.0194985	INF	No Filter	Trace17	5.26	5.24	Automated - Genesis	119662	Echinacea tennesseensis	Flowers		
5	4.096288	245.77728	4.088396	4.10528	137.023758	1323.7004	3173.991555	0.0168838	INF	No Filter	Trace17	6.2	7.78	Automated - Genesis	119662	Echinacea tennesseensis	Flowers		
6	4.2529731	255.17839	4.245081	4.261965	137.023758	1645.50169	3945.718594	0.0168835	INF	No Filter	Trace17	7.71	9.68	Automated - Genesis	119662	Echinacea tennesseensis	Flowers		
7	4.541057	272.46342	4.531556	4.549457	137.024628	255.951278	584.7781143	0.017901	INF	No Filter	Trace17	1.2	1.43	Automated - Genesis	119662	Echinacea tennesseensis	Flowers		
8	4.7102186	282.61312	4.702327	4.719211	137.023743	1827.70972	4382.304317	0.0168836	INF	No Filter	Trace17	8.56	10.75	Automated - Genesis	119662	Echinacea tennesseensis	Flowers		
9	6.4387048	386.32229	6.430305	6.449807	137.02388	1158.12018	2203.478609	0.0195025	INF	No Filter	Trace17	5.43	5.4	Automated - Genesis	119662	Echinacea tennesseensis	Flowers		
10	8.635988	518.15928	8.627588	8.645489	137.023773	853.65934	1950.368085	0.0179008	INF	No Filter	Trace17	4	4.78	Automated - Genesis	119662	Echinacea tennesseensis	Flowers		
11	11.358059	681.48354	11.34559	11.36968	137.023727	1058.2849	1721.053742	0.024087	INF	No Filter	Trace17	4.96	4.22	Automated - Genesis	119662	Echinacea tennesseensis	Flowers		
12	11.913822	714.82932	11.90221	11.92544	137.023773	2261.94823	3981.675394	0.023235	INF	No Filter	Trace17	10.6	9.76	Automated - Genesis	119662	Echinacea tennesseensis	Flowers		
13	12.810823	768.64938	12.79921	12.82244	137.023773	2490.88278	4384.308201	0.023234	INF	No Filter	Trace17	11.67	10.75	Automated - Genesis	119662	Echinacea tennesseensis	Flowers		
14	12.993763	779.62578	12.98215	13.00538	137.023773	2018.43421	3552.900313	0.023235	INF	No Filter	Trace17	9.46	8.71	Automated - Genesis	119662	Echinacea tennesseensis	Flowers		
1	0.6146845	36.881071	0.607524	0.621845	153.018738	5049.50927	11853.92765	0.01432083	31.99235289	No Filter	Trace18	35.75	38.33	Automated - ICIS	119662	Echinacea tennesseensis	Flowers		
2	0.7125387	42.75232	0.70615	0.720029	153.018555	4298.5036	10451.98181	0.01387858	28.20866639	No Filter	Trace18	30.43	33.8	Automated - ICIS	119662	Echinacea tennesseensis	Flowers		
3	1.010988	60.65928	1.000689	1.016138	153.018631	4778.01813	8621.4992	0.0154491	23.26840967	No Filter	Trace18	33.82	27.88	Automated - ICIS	119662	Echinacea tennesseensis	Flowers		
1	12.575893	754.55358	12.56195	12.58983	216.981293	1049.53734	1480.022777	0.027882	INF	No Filter	Trace20	100	100	Automated - Genesis	119662	Echinacea tennesseensis	Flowers		
1	9.5758763	574.55258	8.818228	10.30381	431.099396	579476.644	15057.48623	1.4855824	INF	No Filter	Trace21	100	100	Automated - Genesis	119662	Echinacea tennesseensis	Flowers		
1	5.5989114	335.93468	5.500639	6.24576	0	6864475.84	732581.1216	0.7451214	91.61718481	No Filter	Trace26	100	100	Automated - Genesis	119662	Echinacea tennesseensis	Flowers	C10H10O4 Ferulic acid	Level 4
1	1.5508623	93.051738	1.500703	1.645021	0	75894.3029	19181.27236	0.144318	INF	No Filter	Trace27	9.54	11.29	Automated - ICIS	119662	Echinacea tennesseensis	Flowers	C17H22O10 Sinapoyl-glucose	Level 3
2	2.0872179	125.23307	2.028416	2.226712	0	87243.7873	17138.24763	0.1982953	INF	No Filter	Trace27	10.96	10.09	Automated - ICIS	119662	Echinacea tennesseensis	Flowers	C17H22O10 Sinapoyl-glucose	Level 3
3	2.4098827	144.59296	2.307663	2.59745	385.114258	251871.634	36163.19065	0.2897861	INF	No Filter	Trace27	31.65	21.29	Automated - ICIS	119662	Echinacea tennesseensis	Flowers	C17H22O10 Sinapoyl-glucose	Level 3
4	2.9214874	175.28924	2.884312	2.943243	385.113647	8483.14464	7264.791633	0.0589312	INF	No Filter	Trace27	1.07	4.28	Automated - ICIS	119662	Echinacea tennesseensis	Flowers	C17H22O10 Sinapoyl-glucose	Level 3
5	2.9734484	178.4069	2.950214	3.00133	0	5391.22444	3801.820405	0.0511162	INF	No Filter	Trace27	0.68	2.24	Automated - ICIS	119662	Echinacea tennesseensis	Flowers		
6	3.128491	187.70946	3.090463	3.170313	385.114136	10791.0006	7319.04671	0.07985	INF	No Filter	Trace27	1.36	4.31	Automated - ICIS	119662	Echinacea tennesseensis	Flowers		
7	3.6521881	219.13129	3.598128	3.78193	0	125147.128	26110.31782	0.1838025	INF	No Filter	Trace27	15.73	15.37	Automated - ICIS	119662	Echinacea tennesseensis	Flowers	C17H22O10 Sinapoyl-glucose	Level 3
8	3.8702834	232.217	3.820296	3.971884	0	95720.8181	19653.16834	0.1515876	INF	No Filter	Trace27	12.03	11.57	Automated - ICIS	119662	Echinacea tennesseensis	Flowers	C17H22O10 Sinapoyl-glucose	Level 3
9	3.9872319	239.23391	3.971884	4.055358	385.114258	17372.5081	6520.725049	0.0834746	INF	No Filter	Trace27	2.18	3.84	Automated - ICIS	119662	Echinacea tennesseensis	Flowers	C17H22O10 Sinapoyl-glucose	Level 3
10	5.4719968	328.31981	5.428672	5.58583	0	113413.578	21896.65215	0.1571583	INF	No Filter	Trace27	14.25	12.89	Automated - ICIS	119662	Echinacea tennesseensis	Flowers		
11	5.6024916	336.1495	5.589411	5.630399	0	4379.18951	4807.52666	0.0409884	INF	No Filter	Trace27	0.55	2.23	Automated - ICIS	119662	Echinacea tennesseensis	Flowers		
1	0.7176876	43.061255	0.70973	0.726747	335.078003	4075.59721	9709.183242	0.0170167	INF	No Filter	Trace29	13.79	24.59	Automated - Genesis	119662	Echinacea tennesseensis	Flowers		
2	0.8519875	51.119252	0.834967	0.863917	0	25484.9219	29771.63568	0.02895032	INF	No Filter	Trace29	86.21	75.41	Automated - Genesis	119662	Echinacea tennesseensis	Flowers		
1	1.5723102	94.338612	1.558466	1.584128	0	1459.17849	2147.275433	0.0256622	INF	No Filter	Trace1	4.96	4.96	Automated - Genesis	119663	Echinacea pallida	Roots		
2	3.4991921	209.95153	3.488451	3.509933	137.024078	864.185075	1581.730426	0.0214812	INF	No Filter	Trace1	2.94	3.65	Automated - Genesis	119663	Echinacea pallida	Roots		
3	4.151929	249.11574	4.141697	4.162161	137.023773	837.624766	1609.632433	0.020464	INF	No Filter	Trace1	2.85	3.72	Automated - Genesis	119663	Echinacea pallida	Roots		
4	4.2751553	256.50932	4.263328	4.285388	137.023972	360.790056	590.9477331	0.0220598	INF	No Filter	Trace1	1.23	1.36	Automated - Genesis	119663	Echinacea pallida	Roots		
5	4.481465	268.8879	4.470725	4.492206	137.024323	299.19768	547.626127	0.0214813	INF	No Filter	Trace1	1.02	1.26	Automated - Genesis	119663	Echinacea pallida	Roots		
6	4.5618267	273.7096	4.551086	4.572568	137.023987	296.997679	543.587599	0.0214813	INF	No Filter	Trace1	1.01	1.26	Automated - Genesis	119663	Echinacea pallida	Roots		
7	4.6414032	278.48419	4.631172	4.651645	137.023788	1014.20933	1948.94577	0.0204733	INF	No Filter	Trace1	3.45	4.5	Automated - Genesis	119663	Echinacea pallida	Roots		
8	6.7093905	402.56343	6.69865	6.720131	137.023727	1723.79394	3155.105905	0.0214812	INF	No Filter	Trace1	5.86	7.28	Automated - Genesis	119663	Echinacea pallida	Roots		
9	8.6339119	518.03471	8.623171	8.644652	137.023773	1815.84461	3323.556175	0.0214814	INF	No Filter	Trace1	6.17	7.67	Automated - Genesis	119663	Echinacea pallida	Roots		
10	8.8786701	532.72021	8.867929	8.88941	137.023743	780.660747	1428.866969	0.0214809	INF	No Filter	Trace1	2.65	3.3	Automated - Genesis	119663	Echinacea pallida	Roots		
11	10.340092	620.40552	10.32615	10.35403	137.023788	1260.09073	1776.916427	0.027882	INF	No Filter	Trace1	4.28	4.1	Automated - Genesis	119663	Echinacea pallida	Roots		
12	10.412969	624.77814	10.39903	10.42691	137.023819	1227.77944	1731.408463	0.027882	INF	No Filter	Trace1	4.17	4	Automated - Genesis	119663	Echinacea pallida	Roots		
13	10.551748	633.10488	10.53781	10.56569	137.024323	1105.02193	1558.240202	0.027882	INF	No Filter	Trace1	3.76	3.6	Automated - Genesis	119663	Echinacea pallida	Roots		
14	10.650175	639.0105	10.63623	10.66412	137.024017	980.764217	1383.018913	0.027882	INF	No Filter	Trace1	3.33	3.19	Automated - Genesis	119663	Echinacea pallida	Roots		
15	11.154813	669.28878	11.14087	11.16875	137.023773	1544.92017	2178.601589	0.027881	INF	No Filter	Trace1	5.25	5.03	Automated - Genesis	119663	Echinacea pallida	Roots		
16	11.61766	697.0596	11.60287	11.6316	137.023972	1182.67441	1656.006225	0.028733	INF	No Filter	Trace1	4.02	3.82	Automated - Genesis	119663	Echinacea pallida	Roots		
17	11.919948	715.19688	11.90601	11.93389	137.023788	1330.46483	1876.139478	0.027881	INF	No Filter	Trace1	4.52	4.33	Automated - Genesis	119663	Echinacea pallida	Roots		
18	12.202189	732.13134	12.18739	12.21613	137.024063	1261.90145	1776.270993	0.028741	INF	No Filter	Trace1	4.29	4.1	Automated - Genesis	119663	Echinacea pallida	Roots		
19	12.368864	742.13184	12.35407	12.38281	137.023727	1956.25588	2753.666093	0.028739	INF	No Filter	Trace1	6.65	6.36	Automated - Genesis	119663	Echinacea pallida	Roots		
20	12.575044	754.50264	12.5611	12.58898	137.023834	1123.97856	1584.96528	0.027881	INF	No Filter	Trace1	3.82	3.66	Automated - Genesis	119663	Echinacea pallida	Roots		

21	12.627017	757.62102	12.61308	12.64096	137.023788	1310.31556	1847.733812	0.027882	INF	No Filter	Trace1	4.46	4.27	Automated - Genesis	119663	Echinacea pallida	Roots		
22	12.779756	766.78536	12.76496	12.7937	137.023773	1248.45005	1701.455827	0.028737	INF	No Filter	Trace1	4.24	3.93	Automated - Genesis	119663	Echinacea pallida	Roots		
23	13.518497	811.10982	13.50456	13.53329	136.01622	906.443046	1212.097292	0.028738	INF	No Filter	Trace1	3.08	2.8	Automated - Genesis	119663	Echinacea pallida	Roots		
24	13.645675	818.7405	13.61547	13.65962	137.023758	2396.51915	1812.665692	0.044146	INF	No Filter	Trace1	8.15	4.19	Automated - Genesis	119663	Echinacea pallida	Roots		
25	13.706942	822.41652	13.693	13.72088	137.023758	1123.15524	1583.800924	0.027882	INF	No Filter	Trace1	3.82	3.66	Automated - Genesis	119663	Echinacea pallida	Roots		
1	0.9189247	55.135484	0.86861	1.001456	0	69348.3104	26199.8636	0.132846174	13.99395936	No Filter	Trace3	6.6	17.91	Manual	119663	Echinacea pallida	Roots	C13H16O8 SA 2-O-glucoside	Level 2
2	1.249465	74.9679	1.195616	1.399994	137.023315	109762.394	27933.83376	0.20437873	15.07156044	No Filter	Trace3	10.45	19.09	Manual	119663	Echinacea pallida	Roots		
3	1.5340406	92.042436	1.491965	1.655468	137.023087	45343.3342	12213.49817	0.163502984	6.473357951	No Filter	Trace3	4.32	8.35	Manual	119663	Echinacea pallida	Roots	C13H16O8 SA 5-O-glucoside	Level 2
4	1.8100688	108.60413	1.716781	2.391231	137.023209	825839.171	79971.7133	0.674449809	43.10524947	No Filter	Trace3	78.63	54.66	Manual	119663	Echinacea pallida	Roots		
1	0.9150155	54.90093	0.886459	0.983588	0	21607.3823	9587.851463	0.09712985	INF	No Filter	Trace4	58.04	53.96	Automated - Genesis	119663	Echinacea pallida	Roots		
2	1.0065393	60.392358	0.985929	1.027136	93.0332108	1229.34447	1026.327786	0.0412068	INF	No Filter	Trace4	3.3	5.78	Automated - Genesis	119663	Echinacea pallida	Roots		
3	1.5276686	91.660116	1.499805	1.616097	93.0332184	11551.5799	4558.648828	0.1162912	INF	No Filter	Trace4	31.03	25.66	Automated - Genesis	119663	Echinacea pallida	Roots		
4	4.4381444	266.28866	4.390859	4.452824	0	1634.24418	1411.468294	0.0619652	INF	No Filter	Trace4	4.39	7.94	Automated - Genesis	119663	Echinacea pallida	Roots		
5	4.603985	276.2391	4.572568	4.618165	0	1207.50848	1184.13215	0.045597	INF	No Filter	Trace4	3.24	6.66	Automated - Genesis	119663	Echinacea pallida	Roots		
1	0.9784383	58.706295	0.968139	0.988751	225.040146	942.7107	1799.313712	0.0206121	INF	No Filter	Trace5	18.9	24.03	Automated - ICIS	119663	Echinacea pallida	Roots		
2	1.2135354	72.812124	1.20327	1.2238	225.041107	1043.94148	1999.823208	0.0205299	INF	No Filter	Trace5	20.93	26.71	Automated - ICIS	119663	Echinacea pallida	Roots		
3	2.0637193	123.82316	2.053455	2.073988	225.040482	889.487374	1703.468732	0.0205325	INF	No Filter	Trace5	17.83	22.75	Automated - ICIS	119663	Echinacea pallida	Roots	Isochorismate C10H10O6	Level 4
4	2.1166666	127	2.094518	2.126932	225.039886	2112.58073	1985.37061	0.0324145	INF	No Filter	Trace5	42.35	26.51	Automated - ICIS	119663	Echinacea pallida	Roots	Isochorismate C10H10O6	Level 4
1	0.6182404	37.094426	0.6075	0.628981	174.076675	2741.22181	5017.288092	0.0214812	INF	No Filter	Trace6	100	100	Automated - Genesis	119663	Echinacea pallida	Roots		
1	2.4209423	145.25654	2.411679	2.881752	179.03418	740803.034	90610.05369	0.470072993	79.9231831	No Filter	Trace7	100	100	Manual	119663	Echinacea pallida	Roots		
1	0.6683634	40.101803	0.657623	0.679104	163.039322	9527.64291	17438.49001	0.02148121	9.67271322	No Filter	Trace8	100	100	Automated - Genesis	119663	Echinacea pallida	Roots		
1	0.6277413	37.664479	0.602288	0.712525	0	50523.1453	18369.06666	0.11023712	INF	No Filter	Trace9	68.26	35.22	Automated - Genesis	119663	Echinacea pallida	Roots		
2	0.7914263	47.485578	0.774335	0.806875	223.061691	6476.3504	7120.987803	0.03254021	INF	No Filter	Trace9	8.75	13.65	Automated - Genesis	119663	Echinacea pallida	Roots		
3	0.8274721	49.648327	0.817173	0.83777	223.061249	1631.37125	3114.786378	0.02059658	INF	No Filter	Trace9	2.2	5.97	Automated - Genesis	119663	Echinacea pallida	Roots		
4	0.8995675	53.974051	0.889267	0.909866	223.061478	1404.61224	2681.474903	0.0205994	INF	No Filter	Trace9	1.9	5.14	Automated - Genesis	119663	Echinacea pallida	Roots		
5	0.9629899	57.779394	0.952692	0.973288	223.061157	1070.26313	2043.527141	0.02059622	INF	No Filter	Trace9	1.45	3.92	Automated - Genesis	119663	Echinacea pallida	Roots		
6	1.0093479	60.560874	0.99905	1.019646	223.060745	1207.69648	2305.892997	0.02059585	INF	No Filter	Trace9	1.63	4.42	Automated - Genesis	119663	Echinacea pallida	Roots		
7	1.050543	63.03258	1.029944	1.060841	223.060989	2524.44587	2466.483436	0.0308973	INF	No Filter	Trace9	3.41	4.73	Automated - Genesis	119663	Echinacea pallida	Roots		
8	1.0711396	64.268376	1.060841	1.091742	223.06102	2081.57931	2134.301752	0.0309006	INF	No Filter	Trace9	2.81	4.09	Automated - Genesis	119663	Echinacea pallida	Roots		
9	1.2699956	76.199736	1.259731	1.280261	223.060669	1133.53103	2171.443635	0.0205296	INF	No Filter	Trace9	1.53	4.16	Automated - Genesis	119663	Echinacea pallida	Roots		
10	1.6848015	101.08809	1.675307	1.696998	223.060776	1030.31055	2030.050951	0.0216905	INF	No Filter	Trace9	1.39	3.89	Automated - Genesis	119663	Echinacea pallida	Roots		
11	1.7193677	103.16206	1.709892	1.738183	223.060974	1834.59056	1939.325727	0.0282912	INF	No Filter	Trace9	2.48	3.72	Automated - Genesis	119663	Echinacea pallida	Roots		
12	2.1474687	128.84812	2.137197	2.157733	223.061157	1038.00817	1987.701817	0.0205354	INF	No Filter	Trace9	1.4	3.81	Automated - Genesis	119663	Echinacea pallida	Roots		
13	4.0157087	240.94252	4.005477	4.027535	223.060379	1022.32119	1880.490117	0.0220582	INF	No Filter	Trace9	1.38	3.61	Automated - Genesis	119663	Echinacea pallida	Roots		
14	6.1432239	368.59343	6.132484	6.153964	223.060562	1041.33304	1905.994013	0.0214808	INF	No Filter	Trace9	1.41	3.65	Automated - Genesis	119663	Echinacea pallida	Roots	C11H12O5 E-Sinapic acid	Level 4
1	1.1563051	69.378306	1.146791	1.166899	353.088898	985.102257	1924.77114	0.0201074	INF	No Filter	Trace10	7.35	18.74	Automated - Genesis	119663	Echinacea pallida	Roots	C16H18O9 neochlorogenic acid (3-o-caffeoylquinic acid)	Level 4
2	1.5789957	94.739742	1.564838	1.645938	0	7123.46441	3386.339489	0.0811005	INF	No Filter	Trace10	53.16	32.98	Automated - Genesis	119663	Echinacea pallida	Roots	C16H18O9 chlorogenic acid (5-o-caffeoylquinic acid)	Level 4
3	1.7028942	102.17365	1.691801	1.738183	353.08844	4051.21335	2502.908458	0.0463823	INF	No Filter	Trace10	30.23	24.37	Automated - Genesis	119663	Echinacea pallida	Roots	C16H18O9 cis 5-o-caffeoylquinic acid	Level 4
4	1.8033847	108.20308	1.793908	1.813962	353.088013	1239.33996	2454.455154	0.0200536	INF	No Filter	Trace10	9.25	23.9	Automated - Genesis	119663	Echinacea pallida	Roots		
1	1.5167557	91.005342	1.359316	1.584165	0	7060576.66	831492.0107	0.224849218	293.2233528	No Filter	Trace12	100	100	Manual	119663	Echinacea pallida	Roots	C13H12O9 caftaric acid	Level 4
1	0.6254008	37.52405	0.602288	0.652803	473.072937	82286.0286	86835.37953	0.05051512	INF	No Filter	Trace13	100	100	Automated - Genesis	119663	Echinacea pallida	Roots	C22H18O12 cichoric acid	Level 4
1	1.5276686	91.660116	1.516756	1.537934	269.046631	440.050337	797.4218875	0.021178	INF	No Filter	Trace16	100	100	Automated - Genesis	119663	Echinacea pallida	Roots		
1	1.5723102	94.338612	1.559706	1.582576	0	1456.73742	2342.921215	0.0228705	INF	No Filter	Trace17	5.11	4.49	Automated - Genesis	119663	Echinacea pallida	Roots		
2	3.4991921	209.95153	3.490792	3.508693	137.024078	864.185323	1974.42248	0.0179012	INF	No Filter	Trace17	3.03	3.78	Automated - Genesis	119663	Echinacea pallida	Roots		
3	4.151929	249.11574	4.144037	4.160921	137.023773	838.014182	2009.251645	0.0168838	INF	No Filter	Trace17	2.94	3.85	Automated - Genesis	119663	Echinacea pallida	Roots		
4	4.2751553	256.50932	4.264864	4.283047	137.023972	372.392724	737.6607731	0.0181834	INF	No Filter	Trace17	1.31	1.41	Automated - Genesis	119663	Echinacea pallida	Roots		
5	4.481465	268.8879	4.471964	4.489865	137.024323	299.197823	683.5838258	0.0179011	INF	No Filter	Trace17	1.05	1.31	Automated - Genesis	119663	Echinacea pallida	Roots		
6	4.5618267	273.7096	4.552326	4.570227	137.023987	296.999181	678.542663	0.0179013	INF	No Filter	Trace17	1.04	1.3	Automated - Genesis	119663	Echinacea pallida	Roots		
7	4.6414032	278.48419	4.633512	4.650405	137.023788	1014.57923	2432.805413	0.0168934	INF	No Filter	Trace17	3.56	4.66	Automated - Genesis	119663	Echinacea pallida	Roots		
8	6.7093905	402.56343	6.70099	6.718891	137.023727	1723.7933	3938.415753	0.0179008	INF	No Filter	Trace17	6.05	7.54	Automated - Genesis	119663	Echinacea pallida	Roots		
9	8.6339119	518.03471	8.625512	8.643413	137.023773	1815.84469	4148.686728	0.0179012	INF	No Filter	Trace17	6.37	7.95	Automated - Genesis	119663	Echinacea pallida	Roots		
10	8.8786701	532.72021	8.87027	8.888171	137.023743	780.659523	1783.608015	0.0179007	INF	No Filter	Trace17	2.74	3.42	Automated - Genesis	119663	Echinacea pallida	Roots		
11	10.340092	620.40552	10.32848	10.35171	137.023788	1260.10705	2218.066797	0.023234	INF	No Filter	Trace17	4.42	4.25	Automated - Genesis	119663	Echinacea pallida	Roots		
12	10.412969	624.77814	10.40135	10.42459	137.023819	1227.76655	2161.260691	0.023234	INF	No Filter	Trace17	4.31	4.14	Automated - Genesis	119663	Echinacea pallida	Roots		
13	10.551748	633.10488	10.54013	10.56337	137.024323	1105.02589	1945.100401	0.023234	INF	No Filter	Trace17	3.88	3.73	Automated - Genesis	119663	Echinacea pallida	Roots		
14	10.650175	639.0105	10.63856	10.66179	137.024017	980.767726	1726.377382	0.023234	INF	No Filter	Trace17	3.44	3.31	Automated - Genesis	119663	Echinacea pallida	Roots		
15	11.154813	669.28878	11.1432	11.16643	137.023773	1544.90666	2719.477278	0.023235	INF	No Filter	Trace17	5.42	5.21	Automated - Genesis	119663	Echinacea pallida	Roots		
16	11.61766	697.0596	11.60519	11.62928	137.023972	1176.98696	2067.138536	0.024086	INF	No Filter	Trace17	4.13	3.96	Automated - Genesis	119663	Echinacea pallida	Roots		
17	11.919948	715.19688	11.90833	11.93157	137.023788	1330.46764	2341.923694	0.023235	INF	No Filter	Trace17	4.67	4.49	Automated - Genesis	119663	Echinacea pallida	Roots		
18	12.202189	732.13134	12.19057	12.21381	137.024063	1259.61427	2217.261122	0.023234	INF	No Filter	Trace17	4.42	4.25	Automated - Genesis	119663	Echinacea pallida	Roots		
19	12.368864	742.13184	12.35725	12.38048	137.023727	1952.71843	3437.311535	0.023234	INF	No Filter	Trace17	6.85	6.58	Automated - Genesis	119663	Echinacea pallida	Roots		
20	12.575044	754.50264	12.56343	12.58666	137.023834	1123.98094	1978.460443	0.023235	INF	No Filter	Trace17	3.94	3.79	Automated - Genesis	119663	Echinacea pallida	Roots		
21	12.627017	757.62102	12.6154	12.63863	137.023788	1310.32025	2306.465828	0.023234	INF	No Filter	Trace17	4.6	4.42	Automated - Genesis	119663	Echinacea pallida	Roots		
22	12.779756	766.78536	12.76728	12.79137	137.023773	1242.44947	2123.871792	0.024089	INF	No Filter	Trace17	4.36	4.07	Automated - Genesis	119663	Echinacea pallida	Roots		
23	13.645675	818.7405	13.61779	13.65729	137.023758	2396.52308	2262.691437	0.039499	INF	No Filter	Trace17	8.41	4.33	Automated - Genesis	119663	Echinacea pallida	Roots		
24	13.706942	822.41652	13.69532	13.71856	137.023758	1123.15524	1977.007016	0.023235	INF	No Filter	Trace17	3.94	3.79	Automated - Genesis	119663	Echinacea pallida	Roots		
1	1.2289331	73.735986	1.2238	1.234068	153.018539	4061.60839	10475.01672	0.010268	22.55933241	No Filter	Trace18	100	100	Automated - ICIS	119663	Echinacea pallida	Roots	C7H6O4 Gentisic acid (2,5-DHBA)	Level 4
1	6.5402482	392.41489	6.4826	6.566549	0	13538.5054	6150.604494	0.0839492	INF	No Filter	Trace21	100	100	Automated - Genesis	119663	Echinacea pallida	Roots		
1	4.3188599	259.13159	4.268444	4.489865	593.152283	960806.034	196037.069	0.2214211	INF	No Filter	Trace22	76.94	73.35	Automated - ICIS	119663	Echinacea pallida	Roots	C27H30O15 Kaempferol diglycosides	Level 3
2	4.5006058	270.03635	4.492206	4.576148	593.150635	20553.8363	6448.481216	0.0839422	INF	No Filter	Trace22	1.65	2.41	Automated - ICIS	119663	Echinacea pallida	Roots		

3	4.6414032	278.48419	4.576148	4.770937	593.152039	247459.961	55183.07628	0.1947885	INF	No Filter	Trace22	19.82	20.65	Automated - ICIS	119663	Echinacea pallida	Roots	C27H30O15 Kaempferol diglycosides	Level 3
4	4.803257	288.19542	4.770937	4.885476	593.153625	15407.1693	5589.416096	0.1145395	INF	No Filter	Trace22	1.23	2.09	Automated - ICIS	119663	Echinacea pallida	Roots		
5	4.96391	297.8346	4.952142	4.999722	0	4550.5723	4003.283673	0.0475804	INF	No Filter	Trace22	0.36	1.5	Automated - ICIS	119663	Echinacea pallida	Roots		
1	4.6094679	276.56807	4.588128	4.646519	0	7027.15472	4966.694788	0.0583906	INF	No Filter	Trace24	40.18	25.02	Automated - Genesis	119663	Echinacea pallida	Roots		
2	4.8918324	293.50994	4.8816	4.917412	447.09491	2413.33179	2877.893509	0.0358121	INF	No Filter	Trace24	13.8	14.5	Automated - Genesis	119663	Echinacea pallida	Roots	C21H20O11 kaempferol-O-glucoside	Level 3
3	4.9299876	295.79926	4.917412	4.944685	0	2127.4879	3109.297294	0.0272737	INF	No Filter	Trace24	12.17	15.67	Automated - Genesis	119663	Echinacea pallida	Roots	C21H20O11 kaempferol-O-glucoside	Level 3
4	4.9600329	297.60197	4.949801	4.970265	447.094086	1183.31864	2273.973148	0.0204641	INF	No Filter	Trace24	6.77	11.46	Automated - Genesis	119663	Echinacea pallida	Roots	C21H20O11 kaempferol-O-glucoside	Level 3
5	5.5708471	334.25083	5.560107	5.581588	447.093262	1202.45014	2200.864488	0.0214807	INF	No Filter	Trace24	6.88	11.09	Automated - Genesis	119663	Echinacea pallida	Roots		
6	5.9066919	354.40151	5.886227	5.916925	447.092773	2495.73326	2425.11666	0.0306979	INF	No Filter	Trace24	14.27	12.22	Automated - Genesis	119663	Echinacea pallida	Roots		
7	5.9271564	355.62938	5.916925	5.937389	447.094696	1037.9323	1994.681422	0.0204646	INF	No Filter	Trace24	5.94	10.05	Automated - Genesis	119663	Echinacea pallida	Roots		
1	0.8738191	52.429145	0.863521	0.884118	216.051178	2020.59052	3857.92564	0.02059678	INF	No Filter	Trace25	100	100	Automated - Genesis	119663	Echinacea pallida	Roots	i9G 2-hydroxyacryloyl-N-glutamate C8H11NO6	Level 4
1	5.5910889	335.46533	5.501221	6.277662	0	5936013.25	621163.7532	0.7764414	93.50973567	No Filter	Trace26	100	100	Automated - Genesis	119663	Echinacea pallida	Roots	C10H10O4 Ferulic acid	Level 4
1	2.0865944	125.19566	2.021042	2.267138	0	146731.913	26016.35426	0.2460951	INF	No Filter	Trace27	21.41	18.09	Automated - ICIS	119663	Echinacea pallida	Roots	C17H22O10 Sinapoyl-glucose	Level 3
2	2.4116482	144.69889	2.33042	2.445025	385.114288	57224.4814	18173.03127	0.1146042	INF	No Filter	Trace27	8.35	12.64	Automated - ICIS	119663	Echinacea pallida	Roots	C17H22O10 Sinapoyl-glucose	Level 3
3	2.4984647	149.90788	2.445025	2.614009	385.114807	84755.7107	18950.28296	0.1689845	INF	No Filter	Trace27	12.37	13.18	Automated - ICIS	119663	Echinacea pallida	Roots	C17H22O10 Sinapoyl-glucose	Level 3
4	2.9659222	177.95533	2.888402	3.017039	385.114075	28494.2795	7092.125727	0.1286371	INF	No Filter	Trace27	4.16	4.93	Automated - ICIS	119663	Echinacea pallida	Roots	C17H22O10 Sinapoyl-glucose	Level 3
5	3.0387987	182.32792	3.021686	3.094562	385.114532	8645.25365	3453.887685	0.072876	INF	No Filter	Trace27	1.26	2.4	Automated - ICIS	119663	Echinacea pallida	Roots		
6	3.2580925	195.48555	3.20797	3.327721	385.113922	48650.209	11982.32324	0.119751	INF	No Filter	Trace27	7.1	8.33	Automated - ICIS	119663	Echinacea pallida	Roots	C17H22O10 Sinapoyl-glucose	Level 3
7	3.6622633	219.7358	3.586715	3.807864	0	212637.718	34084.21031	0.2211494	INF	No Filter	Trace27	31.03	23.7	Automated - ICIS	119663	Echinacea pallida	Roots	C17H22O10 Sinapoyl-glucose	Level 3
8	3.88343	233.0058	3.81298	3.990128	0	87945.7888	18322.05056	0.1771476	INF	No Filter	Trace27	12.83	12.74	Automated - ICIS	119663	Echinacea pallida	Roots	C17H22O10 Sinapoyl-glucose	Level 3
9	4.2121428	252.72857	4.194454	4.260987	0	7288.49299	3709.752078	0.0665335	INF	No Filter	Trace27	1.06	2.58	Automated - ICIS	119663	Echinacea pallida	Roots		
10	4.2712194	256.27316	4.260987	4.303511	385.114777	2894.48374	2004.112989	0.0425244	INF	No Filter	Trace27	0.42	1.39	Automated - ICIS	119663	Echinacea pallida	Roots		
1	1.2877339	77.264034	1.234068	1.835598	0	1652128.91	282426.9652	0.6015304	INF	No Filter	Trace28	100	100	Automated - Genesis	119663	Echinacea pallida	Roots	C15H16O9 z sinapoyl-malate	Level 4
1	0.7457796	44.746775	0.735463	0.757648	0	26682.2321	43097.57941	0.02218542	INF	No Filter	Trace29	46.07	51.02	Automated - Genesis	119663	Echinacea pallida	Roots		
2	0.7862592	47.17555	0.761228	0.795336	335.078278	21520.7795	26002.2941	0.03410793	INF	No Filter	Trace29	37.16	30.78	Automated - Genesis	119663	Echinacea pallida	Roots		
3	0.9019086	54.114517	0.891608	0.913776	0	6272.56241	10299.94683	0.02216815	INF	No Filter	Trace29	10.83	12.19	Automated - Genesis	119663	Echinacea pallida	Roots		
4	0.9253134	55.518803	0.917356	0.951452	335.075684	3441.09299	5077.096065	0.03409642	INF	No Filter	Trace29	5.94	6.01	Automated - Genesis	119663	Echinacea pallida	Roots		
1	5.2135298	312.81179	5.203296	5.223761	93.0344849	226.486412	434.7022219	0.020465	INF	No Filter	Trace1	1.34	1.84	Automated - Genesis	119664	Echinacea pallida	Stems		
2	8.3634784	501.8087	8.351055	8.374219	137.023804	1156.01401	2024.743103	0.0231646	INF	No Filter	Trace1	6.84	8.55	Automated - Genesis	119664	Echinacea pallida	Stems		
3	9.476278	568.57668	9.465537	9.487019	137.023758	1768.44053	3236.790671	0.0214812	INF	No Filter	Trace1	10.46	13.67	Automated - Genesis	119664	Echinacea pallida	Stems		
4	9.5059864	570.35918	9.49418	9.519928	137.023727	1218.47571	1813.688942	0.0257481	INF	No Filter	Trace1	7.2	7.66	Automated - Genesis	119664	Echinacea pallida	Stems		
5	9.9191963	595.15178	9.905255	9.933137	137.023758	1226.11943	1728.987218	0.0278816	INF	No Filter	Trace1	7.25	7.3	Automated - Genesis	119664	Echinacea pallida	Stems		
6	10.332367	619.94202	10.31843	10.34631	137.023743	970.478586	1368.542029	0.027882	INF	No Filter	Trace1	5.74	5.78	Automated - Genesis	119664	Echinacea pallida	Stems		
7	10.640125	638.4075	10.62618	10.65407	137.023849	1465.92656	2067.162885	0.027882	INF	No Filter	Trace1	8.67	8.73	Automated - Genesis	119664	Echinacea pallida	Stems		
8	10.699055	641.9433	10.68427	10.713	137.023727	1297.80173	1798.826852	0.028725	INF	No Filter	Trace1	7.67	7.6	Automated - Genesis	119664	Echinacea pallida	Stems		
9	12.599793	755.98758	12.58585	12.61458	137.023895	1075.64819	1466.635953	0.028726	INF	No Filter	Trace1	6.36	6.19	Automated - Genesis	119664	Echinacea pallida	Stems		
10	12.78588	767.1528	12.77109	12.79982	137.023758	2211.44465	3112.87215	0.02873	INF	No Filter	Trace1	13.08	13.14	Automated - Genesis	119664	Echinacea pallida	Stems		
11	13.224584	793.47504	13.21064	13.23853	137.023834	1083.52134	1527.951794	0.027882	INF	No Filter	Trace1	6.41	6.45	Automated - Genesis	119664	Echinacea pallida	Stems		
12	13.316049	798.96294	13.28584	13.32999	137.023788	2157.85536	1614.017244	0.044146	INF	No Filter	Trace1	12.76	6.82	Automated - Genesis	119664	Echinacea pallida	Stems		
13	13.740836	824.45016	13.7269	13.75478	137.023972	1054.09257	1486.416365	0.027881	INF	No Filter	Trace1	6.23	6.28	Automated - Genesis	119664	Echinacea pallida	Stems		
1	0.9086279	54.517671	0.86861	1.001456	0	20282.1877	8487.685335	0.132846174	4.022584967	No Filter	Trace3	7.11	21.54	Manual	119664	Echinacea pallida	Stems	C13H16O8 SA 2-O-glucoside	Level 2
2	1.2494842	74.969052	1.195616	1.399994	137.023041	11484.14	3192.388011	0.20437873	1.637465835	No Filter	Trace3	4.03	8.1	Manual	119664	Echinacea pallida	Stems		
3	1.5247645	91.48587	1.491965	1.655468	137.023315	7185.79456	2881.00676	0.163502984	1.438989046	No Filter	Trace3	2.52	7.31	Manual	119664	Echinacea pallida	Stems	C13H16O8 SA 5-O-glucoside	Level 2
4	1.8141807	108.85084	1.716781	2.391231	137.023422	246129.404	24844.55089	0.674449809	12.55164368	No Filter	Trace3	86.34	63.05	Manual	119664	Echinacea pallida	Stems		
1	0.9534222	57.205329	0.943124	0.963721	93.0328064	399.888182	763.8404075	0.02059639	INF	No Filter	Trace4	2.58	3.93	Automated - Genesis	119664	Echinacea pallida	Stems		
2	0.9894728	59.368366	0.979169	0.999773	93.032547	345.580877	659.9312822	0.0206038	INF	No Filter	Trace4	2.23	3.4	Automated - Genesis	119664	Echinacea pallida	Stems		
3	3.819481	229.16886	3.809249	3.829713	93.0332489	444.911988	855.3294694	0.020464	INF	No Filter	Trace4	2.87	4.4	Automated - Genesis	119664	Echinacea pallida	Stems		
4	3.9132087	234.79252	3.902969	3.933674	93.0333939	581.622713	641.0227448	0.0307058	INF	No Filter	Trace4	3.75	3.3	Automated - Genesis	119664	Echinacea pallida	Stems		
5	4.023866	241.43196	4.012028	4.034099	93.0331039	439.774036	719.7587067	0.0220715	INF	No Filter	Trace4	2.84	3.71	Automated - Genesis	119664	Echinacea pallida	Stems		
6	4.0954952	245.72971	4.085262	4.107328	93.0334015	309.697744	592.6232818	0.0220663	INF	No Filter	Trace4	2	3.05	Automated - Genesis	119664	Echinacea pallida	Stems		
7	4.1380269	248.28161	4.127794	4.148258	93.0334473	293.226384	563.7096866	0.0204645	INF	No Filter	Trace4	1.89	2.9	Automated - Genesis	119664	Echinacea pallida	Stems		
8	4.2112834	252.677	4.201051	4.221516	93.0333023	401.920785	772.7018917	0.0204651	INF	No Filter	Trace4	2.59	3.98	Automated - Genesis	119664	Echinacea pallida	Stems		
9	4.3050035	258.30021	4.294752	4.315235	93.0333557	273.053723	524.9068931	0.0204831	INF	No Filter	Trace4	1.76	2.7	Automated - Genesis	119664	Echinacea pallida	Stems		
10	4.3408172	260.44903	4.330584	4.378234	93.0330658	1056.4975	693.1004383	0.0476501	INF	No Filter	Trace4	6.81	3.57	Automated - Genesis	119664	Echinacea pallida	Stems		
11	4.3935823	263.61494	4.383351	4.403815	93.0332336	311.920131	599.6514101	0.0204636	INF	No Filter	Trace4	2.01	3.09	Automated - Genesis	119664	Echinacea pallida	Stems		
12	4.4448011	266.68807	4.432456	4.455542	93.0331955	367.606064	620.3908077	0.0230856	INF	No Filter	Trace4	2.37	3.19	Automated - Genesis	119664	Echinacea pallida	Stems		
13	4.4913439	269.48063	4.480603	4.502084	93.0329514	341.399403	624.8669529	0.0214812	INF	No Filter	Trace4	2.2	3.22	Automated - Genesis	119664	Echinacea pallida	Stems		
14	4.5252752	271.51651	4.512825	4.536016	93.0334625	362.97166	564.388094	0.0231906	INF	No Filter	Trace4	2.34	2.91	Automated - Genesis	119664	Echinacea pallida	Stems		
15	4.5825585	274.95351	4.571818	4.593299	93.0332794	317.791337	581.655351	0.0214812	INF	No Filter	Trace4	2.05	3	Automated - Genesis	119664	Echinacea pallida	Stems		
16	4.6532541	279.19525	4.643003	4.663487	93.0331879	323.483712	621.1252731	0.0204844	INF	No Filter	Trace4	2.09	3.2	Automated - Genesis	119664	Echinacea pallida	Stems		
17	4.7264854	283.58912	4.716253	4.736719	93.0331421	348.485978	669.954962	0.0204663	INF	No Filter	Trace4	2.25	3.45	Automated - Genesis	119664	Echinacea pallida	Stems		
18	4.7469506	284.81704	4.736719	4.757183	93.0333023	283.287687	544.5614628	0.0204645	INF	No Filter	Trace4	1.83	2.8	Automated - Genesis	119664	Echinacea pallida	Stems		
19	4.7894902	287.36941	4.762299	4.799724	93.0329895	711.708604	717.8798037	0.0374244	INF	No Filter	Trace4	4.59	3.7	Automated - Genesis	119664	Echinacea pallida	Stems		
20	4.837072	290.22432	4.825304	4.850885	0	582.873768	849.4824826	0.0255813	INF	No Filter	Trace4	3.76	4.37	Automated - Genesis	119664	Echinacea pallida	Stems		
21	4.9784366	298.7062	4.913886	4.99225	0	2171.18442	1196.313698	0.0783638	INF	No Filter	Trace4	14	6.16	Automated - Genesis	119664	Echinacea pallida	Stems		
22	5.0631623	303.78974	5.05015	5.085976	0	1064.42569	1094.003639	0.0358254	INF	No Filter	Trace4	6.86	5.63	Automated - Genesis	119664	Echinacea pallida	Stems		
23	5.1147003	306.88202	5.085976	5.128513	0	1023.15595	992.2415234	0.0425375	INF	No Filter	Trace4	6.6	5.11	Automated - Genesis	119664	Echinacea pallida	Stems		
24	5.1694427	310.16656	5.143862	5.179675	93.0333252	538.819766	522.6604454	0.0358125	INF	No Filter	Trace4	3.48	2.69	Automated - Genesis	119664	Echinacea pallida	Stems		
25	5.2171099	313.02659	5.206877	5.227342	93.0334091	296.899631	570.7459739	0.0204652	INF	No Filter	Trace4	1.91	2.94	Automated - Genesis	119664	Echinacea pallida	Stems		
26	5.2529236	315.17542	5.242691	5.263155	93.0333099	230.82825	443.7554455	0.020464	INF	No Filter	Trace4	1.49	2.29	Automated - Genesis	119664	Echinacea pallida	Stems		
27	5.3312899	319.87739	5.321054	5.358471	93.0333481	537.170927	518.5751224	0.0374174	INF	No Filter	Trace4	3.46	2.67	Automated - Genesis	119664	Echinacea pallida	Stems		

28	5.3738211	322.42927	5.358471	5.399402	93.0328674	1144.97376	899.9142134	0.0409312	INF	No Filter	Trace4	7.38	4.63	Automated - Genesis	119664	Echinacea pallida	Stems		
1	1.2340857	74.045142	1.22382	1.24435	225.040741	1193.48568	2286.19648	0.0205305	INF	No Filter	Trace5	27.4	32.81	Automated - ICIS	119664	Echinacea pallida	Stems		
2	2.2662433	135.9746	2.255979	2.27813	225.040283	802.803731	1470.670017	0.0221515	INF	No Filter	Trace5	18.43	21.11	Automated - ICIS	119664	Echinacea pallida	Stems		
3	2.4417354	146.50412	2.426949	2.455676	225.040634	1312.51672	1733.777106	0.0287276	INF	No Filter	Trace5	30.13	24.88	Automated - ICIS	119664	Echinacea pallida	Stems		
4	12.231588	733.89528	12.21765	12.24553	225.040024	1047.26927	1476.820943	0.027882	INF	No Filter	Trace5	24.04	21.2	Automated - ICIS	119664	Echinacea pallida	Stems		
1	0.636153	38.169182	0.625413	0.646894	174.076279	1873.43081	3428.969795	0.0214809	INF	No Filter	Trace6	41.04	43.38	Automated - Genesis	119664	Echinacea pallida	Stems		
2	0.6898561	41.391365	0.679116	0.702229	174.075653	1951.93305	3055.846954	0.02311349	INF	No Filter	Trace6	42.76	38.66	Automated - Genesis	119664	Echinacea pallida	Stems	C15H17NO9 Isochorismate-9-glutamate	Level 2
3	4.6611464	279.66878	4.650913	4.671379	174.076813	739.057877	1420.256786	0.0204661	INF	No Filter	Trace6	16.19	17.97	Automated - Genesis	119664	Echinacea pallida	Stems		
1	2.5067928	150.40757	2.411679	2.881752	179.034241	219767.554	24584.34054	0.470072993	25.36588541	No Filter	Trace7	100	100	Manual	119664	Echinacea pallida	Stems		
1	0.7331426	43.988558	0.697409	0.743443	163.039444	20108.1867	14876.51484	0.0460336	7.577296062	No Filter	Trace8	100	100	Automated - Genesis	119664	Echinacea pallida	Stems		
1	0.6289926	37.739558	0.602291	0.668375	223.060806	22320.457	10453.23573	0.06608432	INF	No Filter	Trace9	64.78	33.46	Automated - Genesis	119664	Echinacea pallida	Stems		
2	0.7434426	44.606558	0.733143	0.753744	223.060181	2170.08036	4142.297142	0.02060094	INF	No Filter	Trace9	6.3	13.26	Automated - Genesis	119664	Echinacea pallida	Stems		
3	0.7640415	45.842488	0.753744	0.77434	223.06105	1473.55848	2813.510124	0.0205962	INF	No Filter	Trace9	4.28	9.01	Automated - Genesis	119664	Echinacea pallida	Stems		
4	0.9613799	57.682796	0.951081	0.971678	223.061234	1198.11458	2287.54456	0.02059665	INF	No Filter	Trace9	3.48	7.32	Automated - Genesis	119664	Echinacea pallida	Stems		
5	1.0341231	62.047386	1.023178	1.044422	223.06192	951.319707	1812.792398	0.0212439	INF	No Filter	Trace9	2.76	5.8	Automated - Genesis	119664	Echinacea pallida	Stems		
6	2.4603231	147.61939	2.446382	2.474264	223.061447	1131.92842	1596.193487	0.0278814	INF	No Filter	Trace9	3.29	5.11	Automated - Genesis	119664	Echinacea pallida	Stems		
7	2.6595097	159.57058	2.645569	2.673451	223.061172	1531.24544	2159.307461	0.0278816	INF	No Filter	Trace9	4.44	6.91	Automated - Genesis	119664	Echinacea pallida	Stems		
8	2.9199628	175.19777	2.906022	2.933904	223.060104	1001.49629	1412.266024	0.0278813	INF	No Filter	Trace9	2.91	4.52	Automated - Genesis	119664	Echinacea pallida	Stems		
9	4.6201948	277.21169	4.609957	4.630427	223.061279	810.258918	1556.349109	0.0204696	INF	No Filter	Trace9	2.35	4.98	Automated - Genesis	119664	Echinacea pallida	Stems		
10	9.0386436	542.31862	9.027903	9.049384	223.061188	893.572628	1635.515296	0.0214812	INF	No Filter	Trace9	2.59	5.23	Automated - Genesis	119664	Echinacea pallida	Stems		
11	11.071874	664.31244	11.05793	11.08582	223.061722	973.656189	1372.995569	0.027882	INF	No Filter	Trace9	2.83	4.39	Automated - Genesis	119664	Echinacea pallida	Stems		
1	1.1160941	66.965646	1.090772	1.12669	353.088928	5186.17822	7516.69255	0.0359179	INF	No Filter	Trace10	14.87	19.7	Automated - Genesis	119664	Echinacea pallida	Stems		
2	1.1385585	68.31351	1.12669	1.195344	0	16012.6838	10520.32924	0.0686546	INF	No Filter	Trace10	45.9	27.57	Automated - Genesis	119664	Echinacea pallida	Stems	C16H18O9 neochlorogenic acid (3-o-caffeoylquinic acid)	Level 4
3	1.2084219	72.505314	1.198154	1.234086	353.087952	2117.48509	2029.484995	0.0359318	INF	No Filter	Trace10	6.07	5.32	Automated - Genesis	119664	Echinacea pallida	Stems	C16H18O9 neochlorogenic acid (3-o-caffeoylquinic acid)	Level 4
4	1.2443503	74.661018	1.234086	1.254617	353.088318	1057.90487	2026.248606	0.0205309	INF	No Filter	Trace10	3.03	5.31	Automated - Genesis	119664	Echinacea pallida	Stems		
5	1.2818961	76.913766	1.270013	1.292162	353.088745	957.628631	1825.923217	0.0221481	INF	No Filter	Trace10	2.75	4.78	Automated - Genesis	119664	Echinacea pallida	Stems		
6	1.317827	79.06962	1.307562	1.328092	353.08847	1096.42197	2099.988878	0.0205305	INF	No Filter	Trace10	3.14	5.5	Automated - Genesis	119664	Echinacea pallida	Stems		
7	1.4477696	86.866176	1.435884	1.458034	353.088104	1206.7465	2122.1028	0.02215	INF	No Filter	Trace10	3.46	5.56	Automated - Genesis	119664	Echinacea pallida	Stems		
8	2.0936073	125.61644	2.083343	2.103874	353.088867	813.935063	1558.920481	0.0205314	INF	No Filter	Trace10	2.33	4.09	Automated - Genesis	119664	Echinacea pallida	Stems		
9	2.3448583	140.6915	2.334593	2.355123	353.088593	980.332633	1877.93199	0.0205298	INF	No Filter	Trace10	2.81	4.92	Automated - Genesis	119664	Echinacea pallida	Stems		
10	2.8145532	168.87319	2.800612	2.828494	353.087341	1241.07697	1750.11763	0.0278814	INF	No Filter	Trace10	3.56	4.59	Automated - Genesis	119664	Echinacea pallida	Stems		
11	3.1501973	189.01184	3.136256	3.164138	353.088715	1415.09609	1995.500407	0.027882	INF	No Filter	Trace10	4.06	5.23	Automated - Genesis	119664	Echinacea pallida	Stems		
12	3.1873727	191.24236	3.171109	3.205759	0	2797.21848	2838.618966	0.0346506	INF	No Filter	Trace10	8.02	7.44	Automated - Genesis	119664	Echinacea pallida	Stems		
1	0.6241731	37.450383	0.606272	0.639733	0	46548.2174	44970.89521	0.03346087	2.398811421	No Filter	Trace13	100	100	Manual	119664	Echinacea pallida	Stems	C22H18O12 cichoric acid	Level 4
1	4.5981187	275.88712	4.587378	4.609957	269.045013	878.352207	1560.550099	0.0225788	INF	No Filter	Trace16	100	100	Automated - Genesis	119664	Echinacea pallida	Stems		
1	8.3634784	501.8087	8.353396	8.372979	137.023804	1136.25311	2527.42075	0.0195838	INF	No Filter	Trace17	6.82	8.71	Automated - Genesis	119664	Echinacea pallida	Stems		
2	9.476278	568.57668	9.467878	9.485779	137.023758	1768.43835	4040.380182	0.017901	INF	No Filter	Trace17	10.61	13.92	Automated - Genesis	119664	Echinacea pallida	Stems		
3	9.5059864	570.35918	9.49652	9.517604	137.023727	1234.30436	2263.968728	0.0210842	INF	No Filter	Trace17	7.41	7.8	Automated - Genesis	119664	Echinacea pallida	Stems		
4	9.9191963	595.15178	9.907579	9.930814	137.023758	1226.12248	2158.238329	0.0232349	INF	No Filter	Trace17	7.36	7.44	Automated - Genesis	119664	Echinacea pallida	Stems		
5	10.332367	619.94202	10.32075	10.34398	137.023743	970.479774	1708.306361	0.023234	INF	No Filter	Trace17	5.82	5.89	Automated - Genesis	119664	Echinacea pallida	Stems		
6	10.640125	638.4075	10.62851	10.65174	137.023849	1465.9403	2580.37198	0.023235	INF	No Filter	Trace17	8.8	8.89	Automated - Genesis	119664	Echinacea pallida	Stems		
7	10.699055	641.9433	10.68659	10.71067	137.023727	1289.01029	2245.416865	0.024078	INF	No Filter	Trace17	7.74	7.74	Automated - Genesis	119664	Echinacea pallida	Stems		
8	12.599793	755.98758	12.58818	12.61225	137.023895	1070.52771	1830.753805	0.024078	INF	No Filter	Trace17	6.42	6.31	Automated - Genesis	119664	Echinacea pallida	Stems		
9	12.78588	767.1528	12.77426	12.7975	137.023758	2207.49298	3885.696735	0.023234	INF	No Filter	Trace17	13.25	13.39	Automated - Genesis	119664	Echinacea pallida	Stems		
10	13.224584	793.47504	13.21297	13.2362	137.023834	1083.52267	1907.292369	0.023234	INF	No Filter	Trace17	6.5	6.57	Automated - Genesis	119664	Echinacea pallida	Stems		
11	13.316049	798.96294	13.28817	13.32767	137.023788	2157.84687	2014.725062	0.039499	INF	No Filter	Trace17	12.95	6.94	Automated - Genesis	119664	Echinacea pallida	Stems		
12	13.740836	824.45016	13.72922	13.75245	137.023972	1054.0948	1855.445048	0.023235	INF	No Filter	Trace17	6.33	6.39	Automated - Genesis	119664	Echinacea pallida	Stems		
1	0.6110918	36.665508	0.606272	0.617013	153.018616	3148.38813	8188.916794	0.01074034	20.58444483	No Filter	Trace18	31.39	33.55	Automated - ICIS	119664	Echinacea pallida	Stems		
2	0.6325728	37.954365	0.625413	0.639733	153.018585	6881.68779	16217.33134	0.01432047	40.76543586	No Filter	Trace18	68.61	66.45	Automated - ICIS	119664	Echinacea pallida	Stems		
1	12.515282	750.91692	12.50134	12.53009	431.098328	1139.37146	1578.542127	0.028748	INF	No Filter	Trace21	100	100	Automated - Genesis	119664	Echinacea pallida	Stems		
1	2.645569	158.73414	2.631628	2.65951	563.1427	1619.97275	2284.428349	0.0278815	INF	No Filter	Trace23	100	100	Automated - Genesis	119664	Echinacea pallida	Stems		
1	3.95341	237.2046	3.943179	3.963643	447.092804	839.519657	1613.391583	0.0204644	INF	No Filter	Trace24	100	100	Automated - Genesis	119664	Echinacea pallida	Stems		
1	0.860714	51.64284	0.815539	0.89957	0	24141.2767	11322.57108	0.08403068	INF	No Filter	Trace25	92.89	76.07	Automated - Genesis	119664	Echinacea pallida	Stems	i9G 2-hydroxyacryloyl-N-glutamate C8H11NO6	Level 4
2	0.9407832	56.44699	0.930483	0.951081	216.050888	1059.5716	2022.804302	0.02059884	INF	No Filter	Trace25	4.08	13.59	Automated - Genesis	119664	Echinacea pallida	Stems		
3	1.1429186	68.575116	1.13341	1.153512	216.051605	787.472565	1539.068173	0.0201021	INF	No Filter	Trace25	3.03	10.34	Automated - Genesis	119664	Echinacea pallida	Stems		
1	0.6134325	36.805951	0.602291	0.639733	0	49347.2712	53817.38799	0.03744267	INF	No Filter	Trace26	1.54	13.54	Automated - Genesis	119664	Echinacea pallida	Stems		
2	0.6886165	41.316987	0.668375	0.758893	0	56692.1469	22818.90073	0.09051783	INF	No Filter	Trace26	1.76	5.74	Automated - Genesis	119664	Echinacea pallida	Stems		
3	2.9440419	176.64251	2.685068	3.691547	193.049698	657581.855	29119.39946	1.0064786	22.85900534	No Filter	Trace26	20.47	7.32	Automated - Genesis	119664	Echinacea pallida	Stems		
4	5.4116783	324.7007	5.203296	5.499206	0	607636.75	82338.4262	0.2959103	68.08760985	No Filter	Trace26	18.92	20.71	Manual	119664	Echinacea pallida	Stems		
5	5.575999	334.55994	5.518757	5.978654	0	1840996.82	209444.8937	0.459896453	165.1898581	No Filter	Trace26	57.31	52.69	Manual	119664	Echinacea pallida	Stems	C10H10O4 Ferulic acid	Level 4
1	1.5417844	92.507064	1.50423	1.618676	385.114716	26930.2009	8638.784109	0.1144455	INF	No Filter	Trace27	27.53	21.85	Automated - ICIS	119664	Echinacea pallida	Stems	C17H22O10 Sinapoyl-glucose	Level 3
2	1.6822394	100.93436	1.67276	1.71091	385.114777	2165.49292	2355.983319	0.0381504	INF	No Filter	Trace27	2.21	5.96	Automated - ICIS	119664	Echinacea pallida	Stems		
3	1.902815	114.1689	1.893339	1.940158	385.112518	4011.97533	3838.13678	0.0468188	INF	No Filter	Trace27	4.1	9.71	Automated - ICIS	119664	Echinacea pallida	Stems		
4	2.0098709	120.59225	1.961828	2.020136	385.113983	5686.28827	3217.645327	0.0583075	INF	No Filter	Trace27	5.81	8.14	Automated - ICIS	119664	Echinacea pallida	Stems		
5	2.0821031	124.92619	2.052548	2.11576	0	8683.3006	4443.173222	0.0632129	INF	No Filter	Trace27	8.88	11.24	Automated - ICIS	119664	Echinacea pallida	Stems	C17H22O10 Sinapoyl-glucose	Level 3
6	3.6625409	219.75245	3.600076	3.710687	385.114899	30027.0939	7562.170024	0.1106107	INF	No Filter	Trace27	30.69	19.12	Automated - ICIS	119664	Echinacea pallida	Stems	C17H22O10 Sinapoyl-glucose	Level 3
7	3.7429091	224.57455	3.728588	3.76797	0	3025.98843	2430.113123	0.0393824	INF	No Filter	Trace27	3.09	6.15	Automated - ICIS	119664	Echinacea pallida	Stems		
8	3.8955108	233.73065	3.837606	3.943179	385.114349	15701.6457	5401.035567	0.105573	INF	No Filter	Trace27	16.05	13.66	Automated - ICIS	119664	Echinacea pallida	Stems	C17H22O10 Sinapoyl-glucose	Level 3
9	3.999455	239.9673	3.989223	4.026643	385.11438	1605.03819	1653.794429	0.0374201	INF	No Filter	Trace27	1.64	4.18	Automated - ICIS	119664	Echinacea pallida	Stems	C17H22O10 Sinapoyl-glucose	Level 3
1	1.2972973	77.837838	1.276764	1.307562	339.073029	2334.34233	2484.014232	0.0307975	INF	No Filter	Trace28	5.31	7.98	Automated - Genesis	119664	Echinacea pallida	Stems	C15H16O9 z sinapoyl-malate	Level 4
2	1.3383571	80.301426	1.328092	1.348624	339.07193	1050.8295	2012.625551	0.020532	INF	No Filter	Trace28	2.39	6.46	Automated - Genesis	119664	Echinacea pallida	Stems	C15H16O9 z sinapoyl-malate	Level 4
3	2.5680501	154.08301	2.55411	2.581991	339.072998	1189.90912	1677.968291	0.0278812	INF	No Filter	Trace28	2.7	5.39	Automated - Genesis	119664	Echinacea pallida	Stems		

4	2.7750538	166.50323	2.760262	2.788995	339.071838	1179.07294	1556.913528	0.0287327	INF	No Filter	Trace28	2.68	5	Automated - Genesis	119664	Echinacea pallida	Stems		
5	4.7701938	286.21163	4.759958	4.782034	339.071594	1480.51351	2421.279183	0.0220752	INF	No Filter	Trace28	3.37	7.77	Automated - Genesis	119664	Echinacea pallida	Stems		
6	4.9847924	299.08754	4.974561	4.995025	339.071228	1107.18067	2127.617795	0.0204642	INF	No Filter	Trace28	2.52	6.83	Automated - Genesis	119664	Echinacea pallida	Stems		
7	5.0734037	304.40422	5.063162	5.083635	339.07193	1046.63924	2011.045944	0.0204729	INF	No Filter	Trace28	2.38	6.46	Automated - Genesis	119664	Echinacea pallida	Stems		
8	5.2352344	314.11406	5.225001	5.245466	339.07312	1024.1763	1967.974391	0.020465	INF	No Filter	Trace28	2.33	6.32	Automated - Genesis	119664	Echinacea pallida	Stems		
9	5.3159378	318.95627	5.255699	5.408098	0	29050.4908	7130.452202	0.1523997	INF	No Filter	Trace28	66.03	22.9	Automated - Genesis	119664	Echinacea pallida	Stems		
10	5.4200783	325.2047	5.409338	5.430819	339.072083	1211.78742	2217.963331	0.0214813	INF	No Filter	Trace28	2.75	7.12	Automated - Genesis	119664	Echinacea pallida	Stems		
11	5.4753841	328.52305	5.464644	5.486125	339.07193	1081.96933	1980.346124	0.0214812	INF	No Filter	Trace28	2.46	6.36	Automated - Genesis	119664	Echinacea pallida	Stems		
12	6.0405861	362.43517	6.029846	6.051327	339.070831	1149.39568	2103.793719	0.0214812	INF	No Filter	Trace28	2.61	6.76	Automated - Genesis	119664	Echinacea pallida	Stems		
13	12.353251	741.19506	12.33931	12.36806	339.070648	1086.60155	1452.065863	0.028749	INF	No Filter	Trace28	2.47	4.66	Automated - Genesis	119664	Echinacea pallida	Stems		
1	2.5726972	154.36183	2.56108	2.584315	335.076477	1071.85872	1886.730751	0.0232349	INF	No Filter	Trace29	34.5	31.61	Automated - Genesis	119664	Echinacea pallida	Stems	C16H16O8 caffeoyl-shikimate	Level 4
2	2.6525392	159.15235	2.640922	2.664157	335.077454	1078.66864	1898.723261	0.0232343	INF	No Filter	Trace29	34.72	31.81	Automated - Genesis	119664	Echinacea pallida	Stems	C16H16O8 caffeoyl-shikimate	Level 4
3	3.6195784	217.1747	3.611178	3.629079	335.078522	955.984327	2184.166843	0.0179008	INF	No Filter	Trace29	30.77	36.59	Automated - Genesis	119664	Echinacea pallida	Stems		
1	4.6304265	277.82559	4.620195	4.640662	510.078156	879.466649	1689.522	0.0204674	INF	No Filter	Trace30	100	100	Automated - Genesis	119664	Echinacea pallida	Stems		
1	0.9122198	54.733185	0.86861	1.001456	137.023575	26440.6438	9939.157467	0.132846174	4.63687255	No Filter	Trace3	7.84	18.64	Manual	119665	Echinacea pallida	Leaves	C13H16O8 SA 2-O-glucoside	Level 2
2	1.2456547	74.739282	1.195616	1.399994	137.022934	29595.366	7633.471182	0.20437873	3.827830836	No Filter	Trace3	8.77	14.32	Manual	119665	Echinacea pallida	Leaves		
3	1.5620298	93.721788	1.491965	1.655468	137.023453	40357.9825	11528.02445	0.163502984	5.245038395	No Filter	Trace3	11.97	21.63	Manual	119665	Echinacea pallida	Leaves	C13H16O8 SA 5-O-glucoside	Level 2
4	1.8024763	108.14858	1.716781	2.391231	137.02327	240880.013	24207.17726	0.674449809	12.15390703	No Filter	Trace3	71.42	45.41	Manual	119665	Echinacea pallida	Leaves		
1	0.91737	55.042198	0.896754	0.948265	93.0333099	4326.7779	2644.001531	0.05151101	INF	No Filter	Trace4	14.29	9.02	Automated - Genesis	119665	Echinacea pallida	Leaves		
2	1.5379199	92.275194	1.517389	1.607763	93.0333328	9266.55268	3965.881434	0.0903743	INF	No Filter	Trace4	30.61	13.53	Automated - Genesis	119665	Echinacea pallida	Leaves		
3	3.8021876	228.13126	3.791447	3.812419	93.0334396	377.98914	708.7961514	0.0209725	INF	No Filter	Trace4	1.25	2.42	Automated - Genesis	119665	Echinacea pallida	Leaves		
4	3.8754106	232.52464	3.865179	3.885643	93.0333557	309.983053	595.9096343	0.0204635	INF	No Filter	Trace4	1.02	2.03	Automated - Genesis	119665	Echinacea pallida	Leaves		
5	3.9998215	239.98929	3.989589	4.020286	93.0333786	578.766281	650.1908201	0.0306966	INF	No Filter	Trace4	1.91	2.22	Automated - Genesis	119665	Echinacea pallida	Leaves		
6	4.0321225	241.92735	4.020286	4.042357	93.0334015	297.480528	547.0498046	0.0220715	INF	No Filter	Trace4	0.98	1.87	Automated - Genesis	119665	Echinacea pallida	Leaves		
7	4.0730706	244.38424	4.062838	4.083302	93.033371	353.45944	679.5139075	0.0204642	INF	No Filter	Trace4	1.17	2.32	Automated - Genesis	119665	Echinacea pallida	Leaves		
8	4.1104835	246.62901	4.09865	4.120715	93.0334625	314.683858	515.2175936	0.0220655	INF	No Filter	Trace4	1.04	1.76	Automated - Genesis	119665	Echinacea pallida	Leaves		
9	4.1718752	250.31251	4.161643	4.182107	93.0334167	271.358354	521.6795744	0.0204642	INF	No Filter	Trace4	0.9	1.78	Automated - Genesis	119665	Echinacea pallida	Leaves		
10	4.1990603	251.94362	4.187227	4.209292	93.0330658	306.008696	562.9841962	0.0220648	INF	No Filter	Trace4	1.01	1.92	Automated - Genesis	119665	Echinacea pallida	Leaves		
11	4.2399883	254.3993	4.229756	4.25022	93.0334473	253.660584	487.6892741	0.0204635	INF	No Filter	Trace4	0.84	1.66	Automated - Genesis	119665	Echinacea pallida	Leaves		
12	4.4246075	265.47645	4.412627	4.45361	0	1205.85839	1227.440013	0.040983	INF	No Filter	Trace4	3.98	4.19	Automated - Genesis	119665	Echinacea pallida	Leaves		
13	4.5157128	270.94277	4.500152	4.548294	0	1006.79762	855.9043851	0.0481421	INF	No Filter	Trace4	3.33	2.92	Automated - Genesis	119665	Echinacea pallida	Leaves		
14	4.5840965	275.04579	4.562615	4.594837	93.0330429	739.730916	684.5278832	0.0322218	INF	No Filter	Trace4	2.44	2.34	Automated - Genesis	119665	Echinacea pallida	Leaves		
15	4.6473001	278.83801	4.613828	4.675221	0	1732.84538	1034.519539	0.0613928	INF	No Filter	Trace4	5.72	3.53	Automated - Genesis	119665	Echinacea pallida	Leaves		
16	4.697294	281.83764	4.685453	4.707525	93.0325699	270.758235	498.0012111	0.0220719	INF	No Filter	Trace4	0.89	1.7	Automated - Genesis	119665	Echinacea pallida	Leaves		
17	4.733117	283.98702	4.722885	4.743349	93.0334244	396.954088	763.1435221	0.020464	INF	No Filter	Trace4	1.31	2.6	Automated - Genesis	119665	Echinacea pallida	Leaves		
18	4.7807658	286.84595	4.76893	4.790997	93.0328293	301.766012	555.1413551	0.0220672	INF	No Filter	Trace4	1	1.89	Automated - Genesis	119665	Echinacea pallida	Leaves		
19	4.8334622	290.00773	4.821695	4.847278	0	668.201396	968.6984565	0.025583	INF	No Filter	Trace4	2.21	3.31	Automated - Genesis	119665	Echinacea pallida	Leaves		
20	4.8708774	292.25264	4.85911	4.884689	0	691.208115	1026.710952	0.0255786	INF	No Filter	Trace4	2.28	3.5	Automated - Genesis	119665	Echinacea pallida	Leaves		
21	4.9051534	294.3092	4.894922	4.925619	93.0333405	671.782816	771.6491723	0.0306968	INF	No Filter	Trace4	2.22	2.63	Automated - Genesis	119665	Echinacea pallida	Leaves		
22	4.9594521	297.56713	4.947684	4.973265	0	692.762617	1030.424493	0.0255804	INF	No Filter	Trace4	2.29	3.52	Automated - Genesis	119665	Echinacea pallida	Leaves		
23	4.9834962	299.00977	4.973265	4.993732	93.0333252	370.25922	711.5536004	0.0204671	INF	No Filter	Trace4	1.22	2.43	Automated - Genesis	119665	Echinacea pallida	Leaves		
24	5.0141986	300.85192	5.003967	5.026053	93.0331421	457.02735	873.1995899	0.0220868	INF	No Filter	Trace4	1.51	2.98	Automated - Genesis	119665	Echinacea pallida	Leaves		
25	5.0720977	304.32586	5.046517	5.08233	93.0332413	564.141713	543.194857	0.035813	INF	No Filter	Trace4	1.86	1.85	Automated - Genesis	119665	Echinacea pallida	Leaves		
26	5.1315379	307.89227	5.11975	5.145349	0	823.897481	1255.899558	0.0255989	INF	No Filter	Trace4	2.72	4.29	Automated - Genesis	119665	Echinacea pallida	Leaves		
27	5.1622343	309.73406	5.150466	5.176046	0	622.421091	907.0427182	0.02558	INF	No Filter	Trace4	2.06	3.1	Automated - Genesis	119665	Echinacea pallida	Leaves		
28	5.2098847	312.59308	5.198117	5.239045	0	937.38027	917.6711276	0.040928	INF	No Filter	Trace4	3.1	3.13	Automated - Genesis	119665	Echinacea pallida	Leaves		
29	5.264625	315.8775	5.254393	5.276463	93.0331955	333.109934	637.4439116	0.0220703	INF	No Filter	Trace4	1.1	2.18	Automated - Genesis	119665	Echinacea pallida	Leaves		
30	5.2866942	317.20165	5.276463	5.296926	93.0333939	353.701327	679.9703387	0.0204634	INF	No Filter	Trace4	1.17	2.32	Automated - Genesis	119665	Echinacea pallida	Leaves		
31	5.3225076	319.35046	5.312275	5.332739	93.0334244	253.325136	487.0273769	0.020464	INF	No Filter	Trace4	0.84	1.66	Automated - Genesis	119665	Echinacea pallida	Leaves		
32	5.3480875	320.88525	5.337855	5.359921	93.0329895	231.981432	443.9466091	0.0220657	INF	No Filter	Trace4	0.77	1.51	Automated - Genesis	119665	Echinacea pallida	Leaves		
33	5.3906167	323.437	5.380384	5.400849	93.0327377	287.762682	553.2296236	0.0204652	INF	No Filter	Trace4	0.95	1.89	Automated - Genesis	119665	Echinacea pallida	Leaves		
1	2.5789482	154.73689	2.411679	2.881752	179.034332	113691.8	11251.69786	0.470072993	INF	No Filter	Trace7	100	100	Manual	119665	Echinacea pallida	Leaves		
1	0.634913	38.094777	0.602957	0.674494	0	18475.4466	9017.145347	0.071537292	INF	No Filter	Trace9	100	100	Manual	119665	Echinacea pallida	Leaves		
1	1.5620298	93.721788	1.400087	1.604479	0	1359178.69	156255.4635	0.204392263	INF	No Filter	Trace12	100	100	Manual	119665	Echinacea pallida	Leaves	C13H12O9 caftaric acid	Level 4
1	0.6277525	37.665152	0.606272	0.639733	0	4974.08859	9016.113807	0.03346087	2.230343078	No Filter	Trace13	100	100	Manual	119665	Echinacea pallida	Leaves	C22H18O12 cichoric acid	Level 4
1	4.6445249	278.67149	4.601998	4.76535	0	104560.258	30120.58608	0.1633521	INF	No Filter	Trace22	100	100	Automated - ICIS	119665	Echinacea pallida	Leaves	C27H30O15 Kaempferol diglycosides	Level 3
1	4.9220378	295.32227	4.877233	4.970924	0	18807.4489	5677.679866	0.0936915	INF	No Filter	Trace24	89.87	58.78	Automated - Genesis	119665	Echinacea pallida	Leaves	C21H20O11 kaempferol-O-glucoside	Level 3
2	5.0118582	300.71149	5.001626	5.022105	447.094055	1124.21484	2157.709232	0.0204786	INF	No Filter	Trace24	5.37	22.34	Automated - Genesis	119665	Echinacea pallida	Leaves		
3	8.9058637	534.35182	8.895123	8.916604	447.091827	996.754381	1824.357433	0.0214813	INF	No Filter	Trace24	4.76	18.89	Automated - Genesis	119665	Echinacea pallida	Leaves		
1	0.6218317	37.309903	0.607511	0.702233	193.04982	112272.329	71911.99639	0.09472237	INF	No Filter	Trace26	17.15	52.33	Automated - Genesis	119665	Echinacea pallida	Leaves		
2	0.712538	42.752277	0.702233	0.722839	193.049774	4931.37663	9412.581916	0.02060529	INF	No Filter	Trace26	0.75	6.85	Automated - Genesis	119665	Echinacea pallida	Leaves		
3	0.9019031	54.114187	0.841296	0.951074	0	28633.9793	10381.54601	0.10977787	INF	No Filter	Trace26	4.37	7.55	Automated - Genesis	119665	Echinacea pallida	Leaves		
4	3.0719849	184.31909	2.421579	3.31407	193.049652	257383.637	10430.59233	0.8924915	8.270024507	No Filter	Trace26	39.31	7.59	Automated - Genesis	119665	Echinacea pallida	Leaves		
5	5.5965735	335.79441	5.53089	5.776798	193.050018	251610.283	35283.39903	0.2459079	6.007784377	No Filter	Trace26	38.42	25.68	Automated - Genesis	119665	Echinacea pallida	Leaves	C10H10O4 Ferulic acid	Level 4
1	2.0872681	125.23609	2.038729	2.221618	0	95552.7165	18927.69134	0.182889	INF	No Filter	Trace27	16.1	14.5	Automated - ICIS	119665	Echinacea pallida	Leaves	C17H22O10 Sinapoyl-glucose	Level 3
2	2.2445004	134.67002	2.221618	2.257559	0	2921.49484	3019.668019	0.0359402	INF	No Filter	Trace27	0.49	2.31	Automated - ICIS	119665	Echinacea pallida	Leaves		
3	2.2729561	136.37737	2.257559	2.300245	385.115143	4464.89886	3632.528741	0.0426861	INF	No Filter	Trace27	0.75	2.78	Automated - ICIS	119665	Echinacea pallida	Leaves		
4	2.503741	150.22446	2.461919	2.567331	385.114288	12966.4142	5298.216013	0.1054119	INF	No Filter	Trace27	2.18	4.06	Automated - ICIS	119665	Echinacea pallida	Leaves	C17H22O10 Sinapoyl-glucose	Level 3
5	2.7107604	162.64562	2.665766	2.727025	0	3603.22812	2704.537432	0.0612589	INF	No Filter	Trace27	0.61	2.07	Automated - ICIS	119665	Echinacea pallida	Leaves		
6	2.952639	177.15834	2.898343	3.030163	385.113342	18628.3982	4513.141901	0.1318193	INF	No Filter	Trace27	3.14	3.46	Automated - ICIS	119665	Echinacea pallida	Leaves	C17H22O10 Sinapoyl-glucose	Level 3
7	3.1286042	187.71625	3.076632	3.144868	385.114075	5547.57714	3190.254259	0.0682361	INF	No Filter	Trace27	0.93	2.44	Automated - ICIS	119665	Echinacea pallida	Leaves		
8	3.2623449	195.74069	3.201482	3.398018	385.114624	137878.551	29099.81012	0.1965366	INF	No Filter	Trace27	23.23	22.29	Automated - ICIS	119665	Echinacea pallida	Leaves	C17H22O10 Sinapoyl-glucose	Level 3
9	3.6665135	219.99081	3.590971	3.799847	0	159990.641	27580.58704	0.2088761	INF	No Filter	Trace27	26.96	21.13	Automated - ICIS	119665	Echinacea pallida	Leaves	C17H22O10 Sinapoyl-glucose	Level 3
10	3.8974143	233.84486	3.815196	3.939599	0	41579.4834	9246.154694	0.1244032	INF	No Filter	Trace27	7.01	7.08	Automated - ICIS	119665	Echinacea pallida	Leaves	C17H22O10 Sinapoyl-glucose	Level 3

11	4.0115897	240.69538	3.939599	4.113258	0	104453.443	19930.27102	0.1736591	INF	No Filter	Trace27	17.6	15.27	Automated - ICIS	119665	Echinacea pallida	Leaves	C17H22O10 Sinapoyl-glucose	Level 3
12	4.1490709	248.94425	4.113258	4.174651	385.115417	5890.40282	3385.219595	0.0613923	INF	No Filter	Trace27	0.99	2.59	Automated - ICIS	119665	Echinacea pallida	Leaves		
1	1.2962631	77.775786	1.243314	1.363008	339.072205	55914.3922	15872.27782	0.1196942	INF	No Filter	Trace28	97.16	83.52	Automated - Genesis	119665	Echinacea pallida	Leaves	C15H16O9 z sinapoyl-malate	Level 4
2	1.3886709	83.320254	1.378405	1.398935	339.072083	1635.38513	3132.735114	0.0205301	INF	No Filter	Trace28	2.84	16.48	Automated - Genesis	119665	Echinacea pallida	Leaves		
1	1.4482033	86.892198	1.436324	1.458469	65.0392914	304.459866	558.1044142	0.0221446	INF	No Filter	Trace2	56.24	55.1	Automated - Genesis	119667	Echinacea pallida	Flowers		
2	7.3959976	443.75986	7.385766	7.406229	89.0398331	236.898418	454.792297	0.0204631	INF	No Filter	Trace2	43.76	44.9	Automated - Genesis	119668	Echinacea pallida	Flowers		
1	0.9343723	56.062337	0.86861	1.001456	0	8183.28977	5826.980905	0.132846174	3.104120912	No Filter	Trace3	0.97	5.5	Manual	119669	Echinacea pallida	Flowers	C13H16O8 SA 2-O-glucoside	Level 2
2	1.2457407	74.744442	1.195616	1.399994	137.023071	46728.2712	14685.77751	0.20437873	7.910118215	No Filter	Trace3	5.55	13.86	Manual	119670	Echinacea pallida	Flowers		
3	1.5331372	91.988232	1.491965	1.655468	137.02301	1040.86329	1770.375481	0.163502984	0.929322915	No Filter	Trace3	0.12	1.67	Manual	119671	Echinacea pallida	Flowers	C13H16O8 SA 5-O-glucoside	Level 2
4	1.7916377	107.49826	1.716781	2.391231	137.023346	785397.776	83671.10693	0.674449809	44.40136313	No Filter	Trace3	93.35	78.97	Manual	119672	Echinacea pallida	Flowers		
1	0.9173514	55.041085	0.907052	0.927655	93.0330734	431.320306	823.4545429	0.02060247	INF	No Filter	Trace4	3.09	4.01	Automated - Genesis	119673	Echinacea pallida	Flowers		
2	3.8405719	230.43431	3.83034	3.86776	93.0331192	572.559415	547.9770654	0.0374198	INF	No Filter	Trace4	4.1	2.67	Automated - Genesis	119674	Echinacea pallida	Flowers		
3	3.898459	233.90754	3.888223	3.908709	93.0331879	324.727367	623.9736724	0.0204857	INF	No Filter	Trace4	2.33	3.03	Automated - Genesis	119675	Echinacea pallida	Flowers		
4	3.9512401	237.07441	3.941008	3.961473	93.0331039	274.985247	528.6108009	0.0204642	INF	No Filter	Trace4	1.97	2.57	Automated - Genesis	119676	Echinacea pallida	Flowers		
5	3.997284	239.83704	3.987052	4.007516	93.0331497	354.993694	682.4786944	0.0204642	INF	No Filter	Trace4	2.54	3.32	Automated - Genesis	119677	Echinacea pallida	Flowers		
6	4.0705371	244.23223	4.060304	4.080769	93.0328522	242.596862	466.346821	0.0204655	INF	No Filter	Trace4	1.74	2.27	Automated - Genesis	119678	Echinacea pallida	Flowers		
7	4.1336365	248.01819	4.123404	4.143871	93.0328293	346.888414	666.8740276	0.0204666	INF	No Filter	Trace4	2.49	3.24	Automated - Genesis	119679	Echinacea pallida	Flowers		
8	4.1915151	251.49091	4.179683	4.201746	93.0327835	290.602684	475.8257111	0.022063	INF	No Filter	Trace4	2.08	2.31	Automated - Genesis	119680	Echinacea pallida	Flowers		
9	4.2852153	257.11292	4.274983	4.295447	93.0334549	264.096833	507.7293385	0.0204633	INF	No Filter	Trace4	1.89	2.47	Automated - Genesis	119681	Echinacea pallida	Flowers		
10	4.3107945	258.64767	4.300563	4.321027	93.033371	305.878264	588.0540746	0.0204634	INF	No Filter	Trace4	2.19	2.86	Automated - Genesis	119682	Echinacea pallida	Flowers		
11	4.336375	260.1825	4.326143	4.346606	93.0331345	275.015397	528.7301642	0.0204638	INF	No Filter	Trace4	1.97	2.57	Automated - Genesis	119683	Echinacea pallida	Flowers		
12	4.3942508	263.65505	4.384019	4.404486	93.0332642	328.578196	631.4522325	0.0204669	INF	No Filter	Trace4	2.35	3.07	Automated - Genesis	119684	Echinacea pallida	Flowers		
13	4.4932573	269.59544	4.470537	4.506338	0	1123.20357	1266.523469	0.0358017	INF	No Filter	Trace4	8.05	6.16	Automated - Genesis	119685	Echinacea pallida	Flowers		
14	4.5557272	273.34363	4.543747	4.583129	0	978.313322	1032.924386	0.0393819	INF	No Filter	Trace4	7.01	5.02	Automated - Genesis	119686	Echinacea pallida	Flowers		
15	4.6384376	278.30626	4.628206	4.648671	93.0330887	252.230959	484.8735593	0.0204642	INF	No Filter	Trace4	1.81	2.36	Automated - Genesis	119687	Echinacea pallida	Flowers		
16	4.6809027	280.85416	4.669135	4.696322	0	754.588088	1083.564322	0.0271866	INF	No Filter	Trace4	5.41	5.27	Automated - Genesis	119688	Echinacea pallida	Flowers		
17	4.7321376	283.92826	4.721906	4.74237	93.0332642	326.136986	626.9608729	0.020464	INF	No Filter	Trace4	2.34	3.05	Automated - Genesis	119689	Echinacea pallida	Flowers		
18	4.7577185	285.46311	4.747486	4.76795	93.0333328	355.320906	683.1477908	0.0204634	INF	No Filter	Trace4	2.55	3.32	Automated - Genesis	119690	Echinacea pallida	Flowers		
19	4.7951361	287.70817	4.784904	4.805368	93.0332642	352.571118	677.7859484	0.020464	INF	No Filter	Trace4	2.53	3.3	Automated - Genesis	119691	Echinacea pallida	Flowers		
20	4.8207164	289.24298	4.810484	4.830948	93.0327682	280.140159	538.603956	0.0204637	INF	No Filter	Trace4	2.01	2.62	Automated - Genesis	119692	Echinacea pallida	Flowers		
21	4.899078	293.94468	4.873498	4.909312	93.0330811	639.274137	654.697778	0.0358146	INF	No Filter	Trace4	4.58	3.18	Automated - Genesis	119693	Echinacea pallida	Flowers		
22	4.9195445	295.17267	4.909312	4.941613	93.0334244	509.130335	503.2571769	0.0323006	INF	No Filter	Trace4	3.65	2.45	Automated - Genesis	119694	Echinacea pallida	Flowers		
23	4.962081	297.72486	4.951846	4.972313	93.0333633	316.036166	607.4920914	0.0204667	INF	No Filter	Trace4	2.27	2.95	Automated - Genesis	119695	Echinacea pallida	Flowers		
24	4.9876679	299.26007	4.977433	4.997919	93.0332794	449.61924	862.5340747	0.0204869	INF	No Filter	Trace4	3.22	4.2	Automated - Genesis	119696	Echinacea pallida	Flowers		
25	5.1341486	308.04892	5.113672	5.144381	93.0332565	653.125096	645.0649204	0.0307091	INF	No Filter	Trace4	4.68	3.14	Automated - Genesis	119697	Echinacea pallida	Flowers		
26	5.2124951	312.74971	5.202264	5.222729	93.0334091	288.140258	553.8758745	0.0204647	INF	No Filter	Trace4	2.07	2.69	Automated - Genesis	119698	Echinacea pallida	Flowers		
27	5.2754924	316.52954	5.263656	5.285724	93.0331268	315.150831	516.1629484	0.0220682	INF	No Filter	Trace4	2.26	2.51	Automated - Genesis	119699	Echinacea pallida	Flowers		
28	5.3010726	318.06436	5.29084	5.321536	93.0333557	660.849797	707.1340411	0.0306961	INF	No Filter	Trace4	4.74	3.44	Automated - Genesis	119700	Echinacea pallida	Flowers		
29	5.3384197	320.30518	5.326652	5.352233	0	664.111221	974.5891497	0.0255807	INF	No Filter	Trace4	4.76	4.74	Automated - Genesis	119701	Echinacea pallida	Flowers		
30	5.3821893	322.93136	5.352233	5.394763	0	1022.64866	1069.933905	0.0425301	INF	No Filter	Trace4	7.33	5.2	Automated - Genesis	119702	Echinacea pallida	Flowers		
1	2.7965357	167.79214	2.782595	2.810476	174.075699	1214.55592	1712.716767	0.0278816	INF	No Filter	Trace6	100	100	Automated - Genesis	119703	Echinacea pallida	Flowers		
1	2.7779479	166.67687	2.411679	2.881752	179.03421	336514.146	29083.35081	0.470072993	INF	No Filter	Trace7	100	100	Manual	119704	Echinacea pallida	Flowers		
1	0.6170125	37.020752	0.602293	0.639733	0	18195.3619	19143.07584	0.03743993	INF	No Filter	Trace9	24	36.75	Automated - Genesis	119705	Echinacea pallida	Flowers		
2	0.6576342	39.458053	0.643313	0.671955	223.061264	6521.67926	9072.62923	0.02864169	INF	No Filter	Trace9	8.6	17.42	Automated - Genesis	119706	Echinacea pallida	Flowers		
3	0.7022314	42.133881	0.689856	0.71253	223.061249	4613.03846	8487.326134	0.02267348	INF	No Filter	Trace9	6.08	16.29	Automated - Genesis	119707	Echinacea pallida	Flowers		
4	2.7050721	162.30433	2.636834	2.808153	223.060638	39157.8174	9051.352619	0.1713193	INF	No Filter	Trace9	51.65	17.38	Automated - Genesis	119708	Echinacea pallida	Flowers	C11H12O5 Z-Sinapic acid	Level 4
5	2.8244174	169.46504	2.808153	2.874073	223.060974	6220.61765	4316.556714	0.0659199	INF	No Filter	Trace9	8.21	8.29	Automated - Genesis	119709	Echinacea pallida	Flowers		
6	3.2434229	194.60537	3.232683	3.254164	223.061707	1103.17262	2019.171667	0.0214809	INF	No Filter	Trace9	1.46	3.88	Automated - Genesis	119710	Echinacea pallida	Flowers		
1	0.7485808	44.91485	0.738283	0.758879	353.089447	18531.2758	35381.64643	0.02059609	INF	No Filter	Trace10	17.03	70.21	Automated - Genesis	119711	Echinacea pallida	Flowers		
2	1.5970822	95.824932	1.5588	1.810456	0	90288.4027	15011.41928	0.2516561	INF	No Filter	Trace10	82.97	29.79	Automated - Genesis	119712	Echinacea pallida	Flowers	C16H18O9 chlorogenic acid (5-o-caffeoylquinic acid); C16H18O9 cis 5-o-caffeoylquinic acid	Level 4
1	1.4944136	89.664816	1.400385	1.624985	0	3837320.71	394820.6538	0.2245995	247.0924332	No Filter	Trace12	100	100	Manual	119713	Echinacea pallida	Flowers	C13H12O9 caftaric acid	Level 4
1	0.6062718	36.376309	0.606272	0.639733	0	0.00090262	0	0.03346087	0	No Filter	Trace13	100	NaN	Manual	119714	Echinacea pallida	Flowers	C22H18O12 cichoric acid	Level 4
1	5.7670744	346.02446	5.756334	5.779426	301.036041	1082.29542	1826.019975	0.0230916	INF	No Filter	Trace14	100	100	Automated - Genesis	119715	Echinacea pallida	Flowers		
1	0.7073808	42.44285	0.698652	0.716439	137.023788	5500.42478	13068.27069	0.01778782	INF	No Filter	Trace17	9.92	11.9	Automated - Genesis	119716	Echinacea pallida	Flowers		
2	0.769177	46.150621	0.761219	0.778253	137.023773	4864.85218	11588.33173	0.01703369	INF	No Filter	Trace17	8.78	10.55	Automated - Genesis	119717	Echinacea pallida	Flowers		
3	1.1345959	68.075754	1.126656	1.143637	137.023773	2454.94253	5978.17187	0.0169818	INF	No Filter	Trace17	4.43	5.44	Automated - Genesis	119718	Echinacea pallida	Flowers		
4	1.2896108	77.376648	1.281687	1.298636	137.023758	2231.94007	5334.843656	0.0169486	INF	No Filter	Trace17	4.03	4.86	Automated - Genesis	119719	Echinacea pallida	Flowers		
5	3.08025	184.815	3.068633	3.091867	137.023956	1639.37929	2885.728709	0.0232345	INF	No Filter	Trace17	2.96	2.63	Automated - Genesis	119720	Echinacea pallida	Flowers		
6	3.160101	189.60606	3.148484	3.171719	137.023849	1050.91748	1849.900698	0.023235	INF	No Filter	Trace17	1.9	1.68	Automated - Genesis	119721	Echinacea pallida	Flowers		
7	3.5971219	215.82731	3.588722	3.608222	137.023773	831.764308	1890.004976	0.0195002	INF	No Filter	Trace17	1.5	1.72	Automated - Genesis	119722	Echinacea pallida	Flowers		
8	3.9795958	238.77575	3.971705	3.988588	137.023743	837.816341	2008.946701	0.0168835	INF	No Filter	Trace17	1.51	1.83	Automated - Genesis	119723	Echinacea pallida	Flowers		
9	4.2045219	252.27131	4.19663	4.213514	137.023773	1133.54636	2717.91098	0.0168833	INF	No Filter	Trace17	2.04	2.47	Automated - Genesis	119724	Echinacea pallida	Flowers		
10	4.2608	255.648	4.252906	4.269797	137.023773	945.108668	2266.149946	0.0168909	INF	No Filter	Trace17	1.71	2.06	Automated - Genesis	119725	Echinacea pallida	Flowers		
11	4.7825632	286.95379	4.774672	4.791555	137.023727	979.909695	2349.501164	0.0168833	INF	No Filter	Trace17	1.77	2.14	Automated - Genesis	119726	Echinacea pallida	Flowers		
12	5.6437435	338.62461	5.635344	5.653245	137.023743	726.625554	1660.149283	0.0179009	INF	No Filter	Trace17	1.31	1.51	Automated - Genesis	119727	Echinacea pallida	Flowers	C7H6O3 p-hydroxybenzoic acid	Level 4
13	6.661009	399.66054	6.652609	6.67051	137.023743	1254.13237	2865.384087	0.0179009	INF	No Filter	Trace17	2.26	2.61	Automated - Genesis	119728	Echinacea pallida	Flowers		
14	7.1110209	426.66125	7.101531	7.120028	137.023743	1231.68005	2745.364812	0.0184975	INF	No Filter	Trace17	2.22	2.5	Automated - Genesis	119729	Echinacea pallida	Flowers		
15	7.4177027	445.06216	7.409809	7.42671	137.023926	1186.00712	2843.49737	0.0169011	INF	No Filter	Trace17	2.14	2.59	Automated - Genesis	119730	Echinacea pallida	Flowers		
16	7.6583342	459.50005	7.649935	7.667835	137.023727	1457.78771	3330.641022	0.0179006	INF	No Filter	Trace17	2.63	3.03	Automated - Genesis	119731	Echinacea pallida	Flowers		
17	8.1263437	487.58062	8.107203	8.135845	137.023758	2293.54821	3120.513592	0.0286416	INF	No Filter	Trace17	4.14	2.84	Automated - Genesis	119732	Echinacea pallida	Flowers		
18	8.2210343	493.26206	8.212634	8.230535	137.023743	894.592598	2043.886102	0.0179012	INF	No Filter	Trace17	1.61	1.86	Automated - Genesis	119733	Echinacea pallida	Flowers		
19	9.417761	565.06566	9.409361	9.427262	137.023911	653.947404	1494.095077	0.017901	INF	No Filter	Trace17	1.18	1.36	Automated - Genesis	119734	Echinacea pallida	Flowers		

20	9.6474595	578.84757	9.635842	9.659076	137.023727	1342.00771	2362.242914	0.0232345	INF		No Filter	Trace17	2.42	2.15	Automated - Genesis	119735	Echinacea pallida	Flowers		
21	9.7040646	582.24388	9.692447	9.715682	137.023819	1015.22986	1787.025904	0.0232346	INF		No Filter	Trace17	1.83	1.63	Automated - Genesis	119736	Echinacea pallida	Flowers		
22	10.208679	612.52074	10.19706	10.2203	137.023743	1468.99532	2585.851627	0.023235	INF		No Filter	Trace17	2.65	2.35	Automated - Genesis	119737	Echinacea pallida	Flowers		
23	10.330348	619.82088	10.31873	10.34197	137.023788	1216.10175	2140.61388	0.023235	INF		No Filter	Trace17	2.19	1.95	Automated - Genesis	119738	Echinacea pallida	Flowers		
24	10.393925	623.6355	10.38231	10.40554	137.023743	1040.62503	1831.815418	0.023235	INF		No Filter	Trace17	1.88	1.67	Automated - Genesis	119739	Echinacea pallida	Flowers		
25	10.473774	628.42644	10.46216	10.48539	137.023987	1242.94267	2187.914599	0.023234	INF		No Filter	Trace17	2.24	1.99	Automated - Genesis	119740	Echinacea pallida	Flowers		
26	11.163657	669.81942	11.15204	11.17528	137.023895	1049.11845	1846.685545	0.023235	INF		No Filter	Trace17	1.89	1.68	Automated - Genesis	119741	Echinacea pallida	Flowers		
27	11.359038	681.54228	11.34658	11.37066	137.024109	1281.19698	2084.827689	0.02408	INF		No Filter	Trace17	2.31	1.9	Automated - Genesis	119742	Echinacea pallida	Flowers		
28	11.403184	684.19104	11.39157	11.4148	137.023758	1298.63383	2285.888994	0.023235	INF		No Filter	Trace17	2.34	2.08	Automated - Genesis	119743	Echinacea pallida	Flowers		
29	11.537322	692.23932	11.5257	11.54894	137.023819	1042.28042	1834.69004	0.023235	INF		No Filter	Trace17	1.88	1.67	Automated - Genesis	119744	Echinacea pallida	Flowers		
30	11.604703	696.28218	11.59309	11.61717	137.023727	2295.42313	3733.251543	0.024087	INF		No Filter	Trace17	4.14	3.4	Automated - Genesis	119745	Echinacea pallida	Flowers		
31	11.686876	701.21256	11.67526	11.7156	137.023743	3154.29955	2900.061919	0.040341	INF		No Filter	Trace17	5.69	2.64	Automated - Genesis	119746	Echinacea pallida	Flowers		
32	12.386892	743.21352	12.37528	12.39851	137.023972	972.17124	1711.299246	0.023235	INF		No Filter	Trace17	1.75	1.56	Automated - Genesis	119747	Echinacea pallida	Flowers		
33	13.003916	780.23496	12.9923	13.01553	137.023743	1451.68802	2555.356022	0.023235	INF		No Filter	Trace17	2.62	2.33	Automated - Genesis	119748	Echinacea pallida	Flowers		
34	13.581428	814.88568	13.56981	13.59305	137.023773	1309.32754	2304.821886	0.023236	INF		No Filter	Trace17	2.36	2.1	Automated - Genesis	119749	Echinacea pallida	Flowers		
35	13.921708	835.30248	13.91009	13.93333	137.023788	2081.21073	3663.410844	0.023234	INF		No Filter	Trace17	3.75	3.33	Automated - Genesis	119750	Echinacea pallida	Flowers		
1	7.5851217	455.1073	7.574381	7.595862	253.040878	818.050626	1497.285954	0.0214813	INF		No Filter	Trace19	100	100	Automated - Genesis	119751	Echinacea pallida	Flowers		
1	12.692344	761.54064	12.67755	12.70628	431.097137	1671.5134	2207.68571	0.02873	INF		No Filter	Trace21	100	100	Automated - Genesis	119752	Echinacea pallida	Flowers		
1	4.332795	259.9677	4.290331	4.375324	0	23197.3618	10788.41919	0.0849926	INF		No Filter	Trace22	18.91	29.18	Automated - ICIS	119753	Echinacea pallida	Flowers	C27H30O15 Kaempferol diglycosides	Level 3
2	4.6309813	277.85888	4.590289	4.754138	593.152344	99501.2121	26180.65366	0.1638491	INF		No Filter	Trace22	81.09	70.82	Automated - ICIS	119754	Echinacea pallida	Flowers	C27H30O15 Kaempferol diglycosides	Level 3
1	5.5979425	335.87655	5.471039	6.507439	0	7851809.82	745104.491	1.0364001		114.7240615	No Filter	Trace26	100	100	Automated - Genesis	119755	Echinacea pallida	Flowers	C10H10O4 Ferulic acid	Level 4
1	1.5485352	92.912112	1.512604	1.584477	385.115356	8980.26699	5035.403934	0.0718736	INF		No Filter	Trace27	0.68	2.11	Automated - ICIS	119756	Echinacea pallida	Flowers	C17H22O10 Sinapoyl-glucose	Level 3
2	2.11194	126.7164	2.030986	2.254944	0	110662.499	18517.6071	0.2239578	INF		No Filter	Trace27	8.4	7.75	Automated - ICIS	119757	Echinacea pallida	Flowers	C17H22O10 Sinapoyl-glucose	Level 3
3	2.2743049	136.45829	2.254944	2.302771	0	5552.10039	4655.346142	0.0478267	INF		No Filter	Trace27	0.42	1.95	Automated - ICIS	119758	Echinacea pallida	Flowers		
4	2.5026794	150.16076	2.318169	2.62754	385.11377	214017.828	27549.41663	0.3093713	INF		No Filter	Trace27	16.25	11.52	Automated - ICIS	119759	Echinacea pallida	Flowers	C17H22O10 Sinapoyl-glucose	Level 3
5	2.9344833	176.069	2.888014	3.068633	385.114532	174853.929	30296.09354	0.1806188	INF		No Filter	Trace27	13.28	12.67	Automated - ICIS	119760	Echinacea pallida	Flowers	C17H22O10 Sinapoyl-glucose	Level 3
6	3.1298962	187.79377	3.068633	3.214781	385.114258	30961.4842	12049.46884	0.1461487	INF		No Filter	Trace27	2.35	5.04	Automated - ICIS	119761	Echinacea pallida	Flowers		
7	3.6511849	219.07109	3.570821	3.806293	0	665019.903	115136.8647	0.235472	INF		No Filter	Trace27	50.5	48.16	Automated - ICIS	119762	Echinacea pallida	Flowers	C17H22O10 Sinapoyl-glucose	Level 3
8	3.86776	232.0656	3.822882	4.052847	0	97240.5261	18016.52046	0.2299656	INF		No Filter	Trace27	7.38	7.54	Automated - ICIS	119763	Echinacea pallida	Flowers	C17H22O10 Sinapoyl-glucose	Level 3
9	4.0654199	243.92519	4.052847	4.092537	0	4538.17039	3329.536531	0.0396897	INF		No Filter	Trace27	0.34	1.39	Automated - ICIS	119764	Echinacea pallida	Flowers		
10	4.1415304	248.49182	4.12618	4.161994	385.113586	4928.36443	4481.87631	0.0358136	INF		No Filter	Trace27	0.37	1.87	Automated - ICIS	119765	Echinacea pallida	Flowers		
1	1.2986356	77.918136	1.237028	1.606872	0	552504.304	93001.55081	0.3698442	INF		No Filter	Trace28	100	100	Automated - Genesis	119766	Echinacea pallida	Flowers	C15H16O9 z sinapoyl-malate	Level 4
1	0.7743275	46.459648	0.766369	0.783403	335.078827	6705.34371	15948.54923	0.0170343	INF		No Filter	Trace29	44.9	44.85	Automated - Genesis	119767	Echinacea pallida	Flowers		
2	0.8789655	52.737928	0.871007	0.888024	335.078857	8230.20654	19607.64125	0.01701672	INF		No Filter	Trace29	55.1	55.15	Automated - Genesis	119768	Echinacea pallida	Flowers		
