# Supplementary material for: Organ-Resolved Salicylate–Phenylpropanoid Metabolite Profiling of Echinacea purpurea, E. pallida, and E. tennesseensis Tissues Extracted with 50% Aqueous Methanol by LC–MS/MS and LC–HRMS
Source: Molecules. 2026 Aug 25;31(17):2963. doi: 10.3390/molecules31172963 (PMC13567413; doi:10.3390/molecules31172963)
Supplement: Supplementary file 1 [file molecules-31-02963-s001.zip › SupplementaryFiles/Supplementary Table S3.pdf]

Table S3. Salicylic acid concentrations in 80 *Echinacea purpurea* flowers

Sample Description	Analyte	µg/g
120696251007-1 Echinacea Flower Head	salicylic_acid	0.742
120697251007-1 Echinacea Flower Head	salicylic_acid	7.14
120698251007-1 Echinacea Flower Head	salicylic_acid	2.01
120699251007-1 Echinacea Flower Head	salicylic_acid	4.654
120700251007-1 Echinacea Flower Head	salicylic_acid	0.491
120701251007-1 Echinacea Flower Head	salicylic_acid	7.61
120702251007-1 Echinacea Flower Head	salicylic_acid	1.01
120703251007-1 Echinacea Flower Head	salicylic_acid	2.012
120704251007-1 Echinacea Flower Head	salicylic_acid	0.578
120705251007-1 Echinacea Flower Head	salicylic_acid	1.871
120706251007-1 Echinacea Flower Head	salicylic_acid	5.618
120707251007-1 Echinacea Flower Head	salicylic_acid	9.263
120708251007-1 Echinacea Flower Head	salicylic_acid	0.845
120709251007-1 Echinacea Flower Head	salicylic_acid	0.461
120710251007-1 Echinacea Flower Head	salicylic_acid	7.313
120711251007-1 Echinacea Flower Head	salicylic_acid	11.582
120712251007-1 Echinacea Flower Head	salicylic_acid	1.34
120713251007-1 Echinacea Flower Head	salicylic_acid	7.535
120714251007-1 Echinacea Flower Head	salicylic_acid	1.841
120715251007-1 Echinacea Flower Head	salicylic_acid	5.645
120716251007-1 Echinacea Flower Head	salicylic_acid	7.071
120717251007-1 Echinacea Flower Head	salicylic_acid	4.294
120718251007-1 Echinacea Flower Head	salicylic_acid	9.829
120719251007-1 Echinacea Flower Head	salicylic_acid	1.713
120720251007-1 Echinacea Flower Head	salicylic_acid	26.29
120721251007-1 Echinacea Flower Head	salicylic_acid	8.082
120722251007-1 Echinacea Flower Head	salicylic_acid	2.266
120723251007-1 Echinacea Flower Head	salicylic_acid	2.398
120724251007-1 Echinacea Flower Head	salicylic_acid	7.895
120725251007-1 Echinacea Flower Head	salicylic_acid	3.653
120726251007-1 Echinacea Flower Head	salicylic_acid	1.687
120727251007-1 Echinacea Flower Head	salicylic_acid	1.744
120728251007-1 Echinacea Flower Head	salicylic_acid	7.745
120729251007-1 Echinacea Flower Head	salicylic_acid	1.543
120730251007-1 Echinacea Flower Head	salicylic_acid	0.311
120731251007-1 Echinacea Flower Head	salicylic_acid	0.557
120732251007-1 Echinacea Flower Head	salicylic_acid	0.734
120733251007-1 Echinacea Flower Head	salicylic_acid	0.947
120734251007-1 Echinacea Flower Head	salicylic_acid	4.801
120735251007-1 Echinacea Flower Head	salicylic_acid	2.165
120736251007-1 Echinacea Flower Head	salicylic_acid	0.979

120737251007-1 Echinacea Flower Head	salicylic_acid	0.628
120738251007-1 Echinacea Flower Head	salicylic_acid	0.934
120739251007-1 Echinacea Flower Head	salicylic_acid	1.843
120740251007-1 Echinacea Flower Head	salicylic_acid	1.762
120741251007-1 Echinacea Flower Head	salicylic_acid	0.288
120742251007-1 Echinacea Flower Head	salicylic_acid	121.476
120743251007-1 Echinacea Flower Head	salicylic_acid	6.066
120744251007-1 Echinacea Flower Head	salicylic_acid	5.763
120745251007-1 Echinacea Flower Head	salicylic_acid	0.925
120746251007-1 Echinacea Flower Head	salicylic_acid	0.642
120747251007-1 Echinacea Flower Head	salicylic_acid	3.192
120748251007-1 Echinacea Flower Head	salicylic_acid	1.261
120749251007-1 Echinacea Flower Head	salicylic_acid	0.231
120750251007-1 Echinacea Flower Head	salicylic_acid	0.521
120751251007-1 Echinacea Flower Head	salicylic_acid	1.328
120752251007-1 Echinacea Flower Head	salicylic_acid	6.17
120753251007-1 Echinacea Flower Head	salicylic_acid	3.734
120754251007-1 Echinacea Flower Head	salicylic_acid	10.476
120755251007-1 Echinacea Flower Head	salicylic_acid	0.216
120756251007-1 Echinacea Flower Head	salicylic_acid	0.599
120757251007-1 Echinacea Flower Head	salicylic_acid	0.721
120758251007-1 Echinacea Flower Head	salicylic_acid	5.944
120759251007-1 Echinacea Flower Head	salicylic_acid	3.204
120760251007-1 Echinacea Flower Head	salicylic_acid	0.74
120761251007-1 Echinacea Flower Head	salicylic_acid	0.613
120762251007-1 Echinacea Flower Head	salicylic_acid	1.352
120763251007-1 Echinacea Flower Head	salicylic_acid	5.065
120764251007-1 Echinacea Flower Head	salicylic_acid	0.953
120765251007-1 Echinacea Flower Head	salicylic_acid	1.437
120766251007-1 Echinacea Flower Head	salicylic_acid	0.363
120767251007-1 Echinacea Flower Head	salicylic_acid	2.795
120768251007-1 Echinacea Flower Head	salicylic_acid	2.245
120769251007-1 Echinacea Flower Head	salicylic_acid	1.164
120770251007-1 Echinacea Flower Head	salicylic_acid	3.462
120771251007-1 Echinacea Flower Head	salicylic_acid	0
120772251007-1 Echinacea Flower Head	salicylic_acid	1.18
120773251007-1 Echinacea Flower Head	salicylic_acid	0.668
120774251007-1 Echinacea Flower Head	salicylic_acid	0.587
120775251007-1 Echinacea Flower Head	salicylic_acid	0.998
