# Supplementary material for: Organ-Resolved Salicylate–Phenylpropanoid Metabolite Profiling of Echinacea purpurea, E. pallida, and E. tennesseensis Tissues Extracted with 50% Aqueous Methanol by LC–MS/MS and LC–HRMS
Source: Molecules. 2026 Aug 25;31(17):2963. doi: 10.3390/molecules31172963 (PMC13567413; doi:10.3390/molecules31172963)
Supplement: Supplementary file 1 [file molecules-31-02963-s001.zip › SupplementaryFiles/Supplementary Table S4.pdf]

Table S4. Analytical Replication Summary

**Study component**	**Biological material**	**Extraction replicates**	**Instrumental replication**
Organ-level LC–MS/MS	2 *E. purpurea* plants; 1 *E. pallida*; 1 *E. tennesseensis*	One extraction per tissue lot	One periodic duplicate after every 20 samples
Organ-level LC–HRMS	Same organ extracts	One extraction per tissue lot	Every extract analyzed twice
80-flower LC–MS/MS	80 individual flower heads	One extraction per flower head	One periodic duplicate after every 20 samples
