# Supplementary material for: Organ-Resolved Salicylate–Phenylpropanoid Metabolite Profiling of Echinacea purpurea, E. pallida, and E. tennesseensis Tissues Extracted with 50% Aqueous Methanol by LC–MS/MS and LC–HRMS
Source: Molecules. 2026 Aug 25;31(17):2963. doi: 10.3390/molecules31172963 (PMC13567413; doi:10.3390/molecules31172963)
Supplement: Supplementary file 1 [file molecules-31-02963-s001.zip › SupplementaryFiles/Supplementary Table S5.pdf]

Table S5. Analytical performance characteristics of the LC–MS/MS method for quantification of salicylic acid (SA), p-hydroxybenzoic acid (p-HBA), benzoic acid (BA), dehydroacetic acid (DhAA), and parabens.

Analyte	SA	p-HBA	BA	DhAA	MeHB	EtHB	PrHB	iPrHB	BuHB	iBuHB	sBuHB
Calibration range (µg/g)	0.010 - 1.000	0.010 - 1.000	0.050-25.0	0.050-1.000	0.010-1.000	0.0020-1.000	0.010-1.000	0.010-1.000	0.010-1.000	0.010-1.000	0.010-1.000
R2	0.9999	>0.9999	0.9971	0.9992	0.9986	>0.9999	>0.9999	>0.9999	0.9999	0.9999	>0.9999
Accuracy	95.9%	98.7%	85.2%	92.1%	87.9%	101.2%	100.3%	98.3%	96.7%	102.2%	99.1%
LOD (µg/g)	<0.010*	<0.010*	0.015	< 0.05*	<0.010*	< 0.0020*	< 0.010*	< 0.010*	< 0.010*	< 0.010*	< 0.010*
LOQ (µg/g)	<0.010*	<0.010*	0.049	< 0.05*	<0.010*	< 0.0020*	< 0.010*	< 0.010*	< 0.010*	< 0.010*	< 0.010*
Injection Precision (%RSD)	2.2%	4.0%	10.4%	1.6%	2.7%	1.3%	3.6%	4.4%	1.7%	1.2%	3.1%
Surrogate Recovery	95.2%	79.5%	105.8%	105.8%	83.3%	74.0%	82.2%	82.2%	82.2%	82.2%	82.2%
Instrument Repeatability (%RSD, ISTD Response)	11.7%	5.4%	10.4%	10.4%	7.9%	4.2%	7.5%	7.5%	7.5%	7.5%	7.5%

* The lowest calibration standard produced peaks with S/N > 10 and blank injections showed no detectable analyte signal; therefore, LOD and LOQ were below the lowest calibration level but were not experimentally determined.
